# Functionalization of remote C(sp^3^)-H bonds enabled by copper-catalyzed coupling of O-acyloximes with terminal alkynes

**DOI:** 10.1038/s41467-020-14292-2

**Published:** 2020-01-21

**Authors:** Zhaodong Li, Rubén O. Torres-Ochoa, Qian Wang, Jieping Zhu

**Affiliations:** 1 Department of Applied Chemistry, College of Material and Energy, South China Agricultural University, 510642 Guangzhou, China; 2 Laboratory of Synthesis and Natural Products, Institute of Chemical Sciences and Engineering, Ecole Polytechnique Fédérale de Lausanne, EPFL-SB-ISIC-LSPN, BCH5304, CH-1015 Lausanne, Switzerland

**Keywords:** Homogeneous catalysis, Reaction mechanisms, Synthetic chemistry methodology

## Abstract

Transition metal catalyzed Sonogashira cross-coupling of terminal alkynes with aryl(vinyl) (pseudo)halides has been successfully extended to alkyl halides for the synthesis of functionalized internal alkynes. The direct alkynylation of remote unfunctionalized sp^3^ carbon by terminal alkynes remains difficult to realize. We report herein an approach to this synthetic challenge by developing two catalytic remote sp^3^ carbon alkynylation protocols. In the presence of a catalytic amount of Cu(I) salt and a tridentate ligand (*t*Bu_3_-terpyridine), *O*-acyloximes derived from cycloalkanones and acyclic ketones are efficiently coupled with terminal alkynes to afford a variety of γ- and δ-alkynyl nitriles and γ-alkynyl ketones, respectively. These reactions proceed through a domino sequence involving copper-catalyzed reductive generation of iminyl radical followed by radical translocation via either β-scission or 1,5-hydrogen atom transfer (1,5-HAT) and copper-catalyzed alkynylation of the resulting translocated carbon radicals. The protocols are applicable to complex natural products.

## Abstract (web summary)

Sonogashira cross-coupling is a powerful strategy to prepare functionalized internal alkynes. Here, the authors report a domino sequence for the generation of γ-/δ-alkynyl nitriles and γ-alkynyl ketones from the coupling of terminal alkynes with O-acyloximes derived from cycloalkanones and acylic ketones, respectively.

## Introduction

**Fig. 1:**
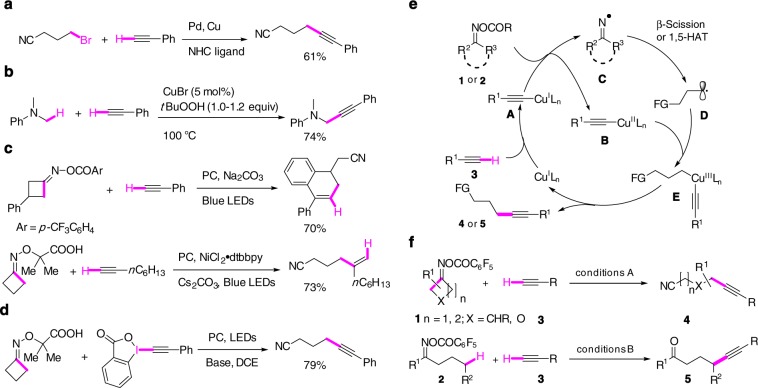
Synthesis of internal alkynes. **a** Sonogashira coupling of unactivated alkyl halides with terminal alkynes (Fu^3^); **b** Cu-catalyzed alkynylation of C(sp^3^)-H adjacent to a nitrogen atom (Li^7^); **c** terminal alkynes as radical acceptors (Chen and Xiao^19^, Leonori^28^); **d** alkynylation of C-radicals with Waser’s EBX reagent^25^; **e** reaction design: working hypothesis; **f** Cu-catalyzed C(sp^3^)–C(sp) coupling of oxime esters with terminal alkynes. NHC *N*-heterocyclic carbene, dtbbp 4,4′-di-*tert*-butyl-2,2′-dipyridyl.

Alkyne is an important functional group in organic synthesis and is also found in natural products and pharmaceuticals^1^. Among many available synthetic methodologies, the Sonogashira reaction is one of the most reliable transformations for the synthesis of internal alkynes^2^. Initially developed for coupling of terminal alkynes with aryl/vinyl (pseudo)halides in the presence of Pd/Cu^3^ or Cu catalyst alone^4,5^, the reaction has subsequently been extended to alkyl bromides/iodides (Fig. 1a) using Pd/Cu^6^, Ni/Cu^7^, and Cu/hν^8^ catalytic systems^9^. In a different approach, in situ oxidation of *N*,*N*-dimethylaniline derivatives to the corresponding iminiums followed by nucleophilic addition of copper acetylides has been developed for the synthesis of propargylamines (Fig. 1b)^10^. The direct alkynylation of remote unfunctionalized sp^3^ carbon by terminal alkynes remains, to the best of our knowledge, unknown.

Using nitrogen-centered radicals (NCRs) as precursors of carbon-centered radicals has become the focus of recent intense research efforts^11–14^. In this context, redox-active acyclic^15^ and cyclic^16^ oxime derivatives, pioneered by Forrester and Zard, respectively, have been demonstrated to be versatile precursors of iminyl radicals under either oxidative or reductive conditions. Depending on the structure of oximes, the iminyl radicals can evolve to a carbon radical through either β-scission^17–32^ or 1,5-hydrogen atom transfer (1,5-HAT) process^33–39^. The resulting carbon radicals can then be trapped by radical acceptors, affording remote C(sp^3^) functionalized alkylnitriles and ketones. However, in spite of a great amount of dedicated efforts, synthetic transformations involving translocated carbon radicals were limited mainly to the radical addition/homolytic substitution (S_H_2) and oxidation reactions. For example, terminal alkynes have been used as radical acceptors by Chen and Xiao for the synthesis of functionalized dihydronaphthalenes (Fig. 1c)^22^. Recent report from Leonori’s group showed that even in the presence of a Ni catalyst, radical addition to terminal alkyne occurred at the expense of the cross-coupling reaction to afford 1,2-disubstituted alkenes (Fig. 1c)^31^. To avoid this radical addition problem, Chen has very recently devised a clever three-component process, in which the primary radical resulting from the β-scission was trapped by styrene to generate a more stable benzylic radical, which can then undergo the Cu-catalyzed cross-coupling with terminal alkynes^32^. To the best of our knowledge, only the Waser’s hypovalent EBX reagent was capable of trapping the primary alkyl radical to afford the γ-alkynyl nitriles (Fig. 1d)^28,40,41^.

Stimulated by the challenges associated with the direct alkynylation of unfunctionalized remote sp^3^ carbon, we became interested in alkynylation of oxime esters with terminal alkynes. The underline principle is outlined in Fig. 1e. Reduction of *O*-acyloximes **1** (cyclic) or **2** (acyclic) by copper acetylide **A**, formed in situ from terminal alkyne **3** and Cu(I) species, would afford Cu(II) intermediate **B** and iminyl radical **C**. β-Scission or 1,5-HAT of the latter would generate the carbon-centered radical **D** which, upon radical oxidative addition to **B**, would afford Cu(III) species **E**. Facile reductive elimination from **E** would furnish the alkynylated product with concurrent regeneration of the Cu(I) catalytic species. In this catalytic cycle, copper went through three oxidation states and the high-valent Cu(III) species, difficult to access by classic oxidative addition, would be formed via a SET process. Although most of radical–Cu(II) rebound processes involved activated secondary or tertiary benzylic carbons^42–44^, we have very recently shown that it is also possible to functionalize the primary radical in the presence of copper under photocatalysis conditions^45,46^. While dual photocatalyst/Cu catalytic system has emerged as a powerful tool for cross-coupling reactions^47^, the catalytic cycle depicted in Fig. 1e using copper as the only catalyst remained uncommon^48–51^. We report herein the successful realization of this endeavor by developing synthesis of γ- and δ-alkynyl nitriles **4** and γ-alkynyl ketones **5** from simple oxime esters **1** or **2** and terminal alkynes **3** (Fig. 1f).

## Results

### Cu-catalyzed alkynylation of cycloalkanone oxime esters

We began our studies by investigating the reductive alkynylation of cyclobutanone oxime esters with phenylacetylene (**3a**). After systematic survey of the ester groups, the copper sources, the ligands, the bases, the temperature, and the solvents with or without Blue LEDs irradiation (Supplementary Methods, Tables 1–7), the optimum conditions found consisted of performing the reaction of **1a** with **3a** in acetonitrile (*c* 0.2 M) in the presence of CuI (0.1 equiv), 4,4,’4”-tri-*tert*-butyl-2,2′:6′,2″-terpyridine (*t*Bu_3_-TERPY, 0.2 equiv) and potassium carbonate (2.0 equiv) at 60 °C. Under these conditions, **4a** was isolated in 76% yield. We stress that the use of *t*Bu_3_-TERPY as a ligand is determinant to the success of the reaction.

**Fig. 2:**
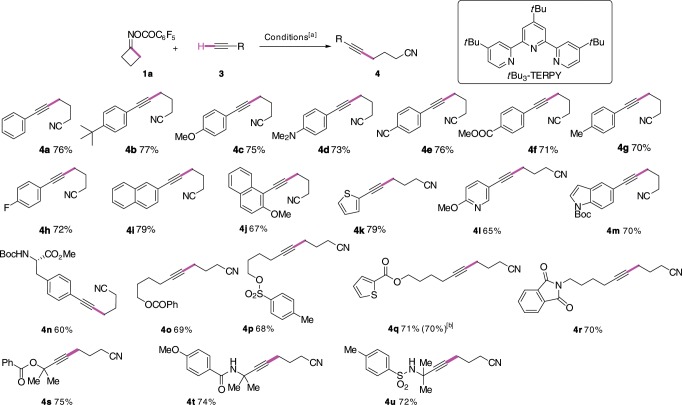
Cu-catalyzed reductive coupling of cyclobutanone oxime ester with terminal alkynes. [a] **1a** (0.2 mmol), **3** (0.4 mmol), CuI (0.1 equiv), *t*Bu_3_-TERPY (0.2 equiv), K_2_CO_3_ (2.0 equiv), CH_3_CN (1.0 mL, *c* 0.2 M), 60 °C, under nitrogen atmosphere. Yields refer to the isolated products. [b] Reaction performed at 2.0 mmol scale.

As shown in Fig. 2, a range of aryl acetylenes bearing electron-donating and electron-withdrawing groups at different positions underwent the C(sp^3^)–C(sp) coupling with *O*-acyloxime **1a** to afford the γ-alkynyl alkylnitriles in good to high yields (**4a**–**4j**). Alkynes attached to an heteroarene such as pyridine, indole, and even thiophene were compatible to afford alkynylated nitriles **4k**–**4m** in satisfactory yields. (*S*)-Methyl 4-ethynyl-*N*-Boc-phenylalanate took part in the reaction to give **4n** in 60% yield. Aliphatic alkynes participated in the reaction to deliver the products **4o**–**4u** in good yields. A range of functional groups, such as ester, amide, carbamate, sulfonamide bearing an acidic proton, were well tolerated. However, reaction of unprotected 4-ethynylaniline and 3-ethynylphenol with **1a** afforded the desired product in low yields (<30%). Performing the reaction of **1a** with **3q** at 2.0 mmol scale under standard conditions provided **4q** in similar isolated yield (70%).

**Fig. 3:**
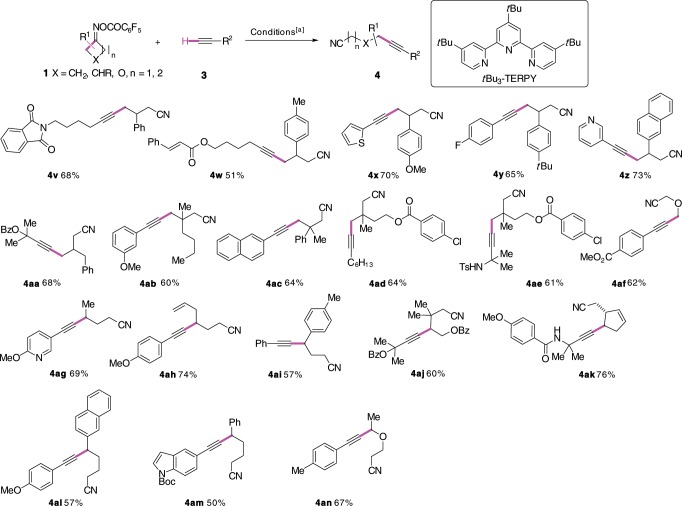
Cu-catalyzed reductive coupling of oxime esters derived from cycloalkanone derivatives. [a] **1** (0.2 mmol), **3** (0.4 mmol), CuI (0.1 equiv), *t*Bu_3_-TERPY (0.2 equiv), K_2_CO_3_ (2.0 equiv), CH_3_CN (1.0 mL, *c* 0.2 M), 60 °C, under nitrogen atmosphere. Yields refer to the isolated products.

The alkynylation protocol was next applied to a diverse set of oxime esters (Fig. 3). Oxime esters derived from C-3 mono- and disubstituted cyclobutanones underwent alkynylation smoothly to afford the corresponding γ-alkynylated nitriles (**4v**–**4af**). Nonsymmetrical C-2 substituted cyclobutanone derivatives underwent β-scission at the more substituted position to deliver the alkynylation products (**4ag**–**4ai)** in good yields. Ring-opening alkynylation of oxetan-3-one oxime ester proceeded well to provide the coupling product **4af** in 62% yield. 2,3,3-Trisubstituted oxime ester was alkynylated without event to afford the highly functionalized alkyne **4aj**. Bicyclo[3.2.0]hept-2-en-6-one-derived oxime ester was converted to *trans*-3,4-disubstituted cyclopentene derivative **4ak** in 76% yield. Gratefully, oxime esters derived from less strained cyclopentanones and dihydrofuran-3(*2H*)-one underwent similar transformation to afford δ-alkynylated nitriles (**4al**, **4am**, **4an**) in good yields. It is nevertheless important to note that the presence of a substituent alpha to the oxime function is needed to drive the fragmentation and that the oxime esters derived from cyclohexanone failed to produce the ω-alkynylated alkylnitriles. We stress that aryl chloride (**4ad**, **4ae**) and alkenes (**4ah**, **4ak**), including α,β-unsaturated ester (**4w**), which are excellent radical acceptors of the transient nucleophilic alkyl radicals, remained unaltered.

### Cu-catalyzed γ-C(sp^3^)-H alkynylation of linear oxime esters

**Fig. 4:**
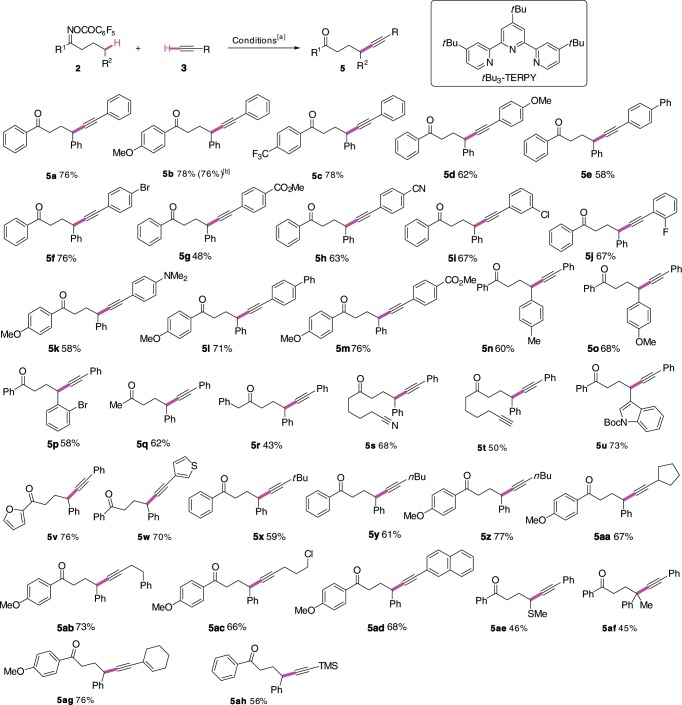
Cu-catalyzed γ-C(sp^3^)-H alkynylation of linear oxime esters. [a] Conditions: **2** (0.1 mmol), **3** (0.2 mmol, 2.0 equiv), (CuOTf)_2_·C_6_H_6_ (0.05 equiv), *t*Bu_3_-TERPY (0.1 equiv), K_2_CO_3_ (2.0 equiv), DCE (2.0 mL, *c* 0.05 M), 45 °C, under nitrogen atmosphere. Yields refer to the isolated products. [b] Reaction performed at 1.0 mmol scale.

Oxime esters derived from linear ketones were next examined for the synthesis of γ-alkynylated ketones by a domino sequence involving reductive generation of iminyl radicals followed by 1,5-HAT and alkynylation of the resulting carbon-centered radicals^52–56^. Reaction of **2a** (R^1^ = R^2^ = Ph) with **3a** (R = Ph) under aforementioned standard conditions afforded only a trace amount of **5a**, with the unfunctionalized ketone being isolated as the major product. This result was not unexpected considering the reversibility of 1,5-HAT of iminyl radicals to benzylic radicals^35^. After an exhaustive optimization of reaction conditions (see Supplementary Methods, Tables 8–14), we found that stirring a DCE solution of **2a** (0.1 mmol) with **3a** (0.2 mmol) at 45 °C in the presence of (CuOTf)_2_·C_6_H_6_ (0.05 equiv), *t*Bu_3_-TERPY (0.1 equiv), and K_2_CO_3_ (2 equiv) provided the desired internal alkyne **5a** in 76%. The generality of this protocol is shown in Fig. 4. Regardless of the electronic nature of the oxime esters and the acetylenes, the γ-C(sp^3^)-H alkynylation proceeded smoothly to afford the corresponding γ-alkynylated ketones (**5a**–**5p**) in good yields. Oxime esters derived from aliphatic ketones (**5q**–**5t**) were selectively alkynylated at the benzylic position. Functional groups such as terminal alkyne (**5t**), nitrile (**5s**), thioether (**5ae**), enyne (**5ag**), alkyl chloride (**5ac**), and heteroarenes (**5u**–**5w**) were well tolerated. Alkynylation on a tertiary carbon was also feasible (**5af**), albeit with reduced yield. An experiment performed at 1.0 mmol scale between *O*-acyloxime **2b** and phenylacetylene (**3a**) gave **5b** in 76% isolated yield. However, the presence of an aryl (R^2^ = Aryl group) or a heteroatom substituent (R^2^ = SMe, **5ae**) in oxime **2** is needed in order for the domino alkynylation process to occur. In fact, the bond dissociation energy (BDE) of iminyl NH bonds (93 kcal/mol) is lower than most of the C(sp^3^)-H bond (96-105 kcal/mol), which makes the 1,5-HAT of iminyl radical to carbon radical thermodynamically unfavorable. One solution to this problem is to perform the reaction under acidic conditions^15,33,39^, which are unfortunately incompatible with the present alkynylation conditions.

**Fig. 5:**
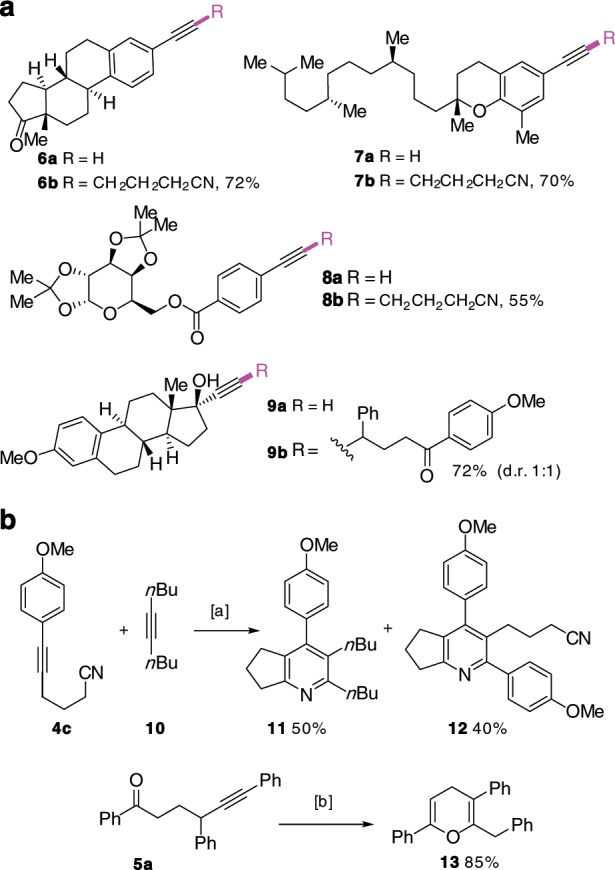
Synthetic application. **a** Functionalization of natural products, **b** post-functionalization of γ-alkynyl nitrile and γ-alkynyl ketone. [a] Ni(COD)_2_ (5 mol%), PBu_3_ (10 mol%), BPh_3_ (20 mol%), toluene, 50 °C, 24 h. [b] *t*BuOK, THF, 0 °C.

Application of these protocols to the late-stage functionalization of natural product-derived alkynes was examined. As shown in Fig. 5a, estrone-derived alkyne **6a**, γ-tocopherol-derived alkyne **7a** and glucose derivative **8a** were successfully engaged in the reaction with cyclic oxime ester **1a** to produce the internal alkynes **6b**, **7b**, and **8b**, respectively, in synthetically useful yields. Reaction of mestranol derivative **9a** with oxime ester **2b** afforded the expected γ-alkynylated ketone **9b** in 72% yield. Finally, post-functionalization of γ-alkynylated alkylnitriles and ketones were performed to demonstrate the synthetic potential of these building blocks. Thus, Ni/BPh_3_-catalyzed [2 + 2 + 2] cycloaddition of nitrile **4c** with dec-5-yne (**10**) afforded the fused pyridines **11** and **12** in 50 and 40% yields, respectively^57^. On the other hand, base-promoted cyclization of the alkynyl ketone **5a** afforded the trisubstituted 4*H*-pyran **13** in 85% yield (Fig. 5b)^58^.

**Fig. 6:**
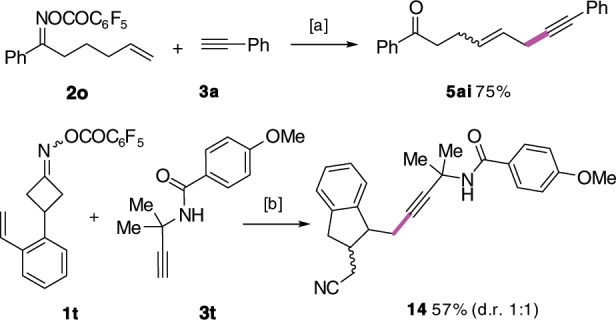
Control experiments. [a] **2o** (0.1 mmol), **3a** (0.2 mmol, 2.0 equiv), (CuOTf)_2_·C_6_H_6_ (0.05 equiv), *t*Bu_3_-TERPY (0.1 equiv), K_2_CO_3_ (2.0 equiv), DCE (2.0 mL, *c* 0.05 M), 45 °C, under nitrogen atmosphere. [b] **1t** (0.2 mmol), **3t** (0.4 mmol), CuI (0.1 equiv), *t*Bu_3_-TERPY (0.2 equiv), K_2_CO_3_ (2.0 equiv), CH_3_CN (1.0 mL, *c* 0.2 M), 60 °C, under nitrogen atmosphere.

Control experiments were conducted to gain insights on the possible reaction mechanism. Addition of radical inhibitors such as TEMPO or TBHP to the reaction mixture suppressed or substantially reduced the product formation (Supplementary Methods, S283). The reaction of **2o** with phenylacetylene **3a** afforded enyne **5ah** in 75% yield, while reaction of **1t** with **3t** provided indane **14** (57%, d.r. 1:1) involving a 5-*exo*-trig radical cyclization before the final Cu-catalyzed cross-coupling reaction (Fig. 6). The results of these control experiments indicated clearly the existence of the radical intermediates and the feasibility of the reaction pathway depicted in Fig. 1e.

## Discussion

Generation of heteroatom-centered radicals followed by β-scission or 1,5-HAT and functionalization of the resulting translocated carbon radicals have been an active research area for the past few years. However, the reported transformations involved mainly the addition of the C-radicals to multiple bonds including alkynes, atom transfer, and reduction/oxidation reaction. To address this limitation, we proposed to combine this radical chemistry with the powerful transition metal-catalyzed cross-coupling reaction, and demonstrated that capture of the C radical by organocopper salts followed by reductive elimination of the resulting Cu(III) intermediate is a highly efficient way to functionalize the translocated C radical. Indeed, we developed efficient and functional group-tolerant Cu-catalyzed syntheses of γ- and δ-alkynyl nitriles and γ-alkynyl ketones, respectively, from readily accessible *O*-acyloximes and terminal alkynes. The reaction proceeded through a domino sequence involving reductive generation of iminyl radical followed by its translocation to a carbon-centered radical via either β-scission or 1,5-HAT and copper-catalyzed coupling of the resulting C(sp^3^) radical with the terminal alkynes. The catalytic amount of copper played a triple roles: it reacted with terminal alkyne to form the copper (I) acetylide, which in turn served as a reductant to reduce the oxime ester to generate the iminyl radical and the Cu(II) species. Finally, Cu(II) intermediate underwent radical rebound with the translocated carbon radical to produce the Cu(III) species. Reductive elimination of the latter afforded the remote alkynylated alkylnitriles or ketones with the concurrent regeneration of the Cu(I) species.

## Methods

### Cu-catalyzed alkynylation of cycloalkanone oxime esters

*O*-acyloximes **1** (0.2 mmol), terminal alkynes **3** (0.4 mmol, 2.0 equiv), K_2_CO_3_ (0.4 mmol, 2.0 equiv), CuI (0.02 mmol, 0.1 equiv), and *t*Bu_3_-TERPY (0.04 mmol, 0.2 equiv) were placed in a dry Schlenk tube. The reaction vessel was evacuated and filled up with nitrogen three times, then CH_3_CN (1.0 mL) was added at rt. After being stirred at 60 °C for 12 h, the reaction mixture was diluted with water and extracted with DCM. The combined organic layers were washed with aqueous NH_4_Cl solution and dried over anhydrous MgSO_4_. After removal of the solvent under reduced pressure, the crude product was purified by column chromatography (silica gel, eluent: ether/petroleum ether) to give the corresponding γ- or δ-alkynyl nitrile **4**.

### Cu-catalyzed γ-C(sp^3^)-H alkynylation of linear oxime esters

A suspension of *O*-acyloximes **2** (0.1 mmol), K_2_CO_3_ (0.2 mmol, 2.0 equiv), *t*Bu_3_-TERPY (0.01 mol, 0.1 equiv), and (CuOTf)_2_·C_6_H_6_ (0.005 mmol, 0.05 equiv) in DCE (*c* 0.05 M) was deoxygenated by freeze–pump–thaw cycles. Alkyne **3** (0.2 mmol, 2.0 equiv) was introduced and the reaction mixture was stirred at 45 °C until the complete consumption of the starting materials (monitored by TLC). The reaction mixture was poured into a saturated NaHCO_3_ solution and extracted with EtOAc. The combined organic layers were washed with aqueous NH_4_Cl solution, dried over Na_2_SO_4_, and evaporated to dryness under reduced pressure. The residue was purified by flash column chromatography on silica gel to afford the corresponding γ-alkynylated ketone **5**.

## Supplementary information

Supplementary Information

## Data Availability

The authors declare that the data supporting the findings of this study are available within the paper and the Supplementary Information, as well as from the authors upon request.
