# Supplementary material for: Functionalization of remote C(sp3)-H bonds enabled by copper-catalyzed coupling of O-acyloximes with terminal alkynes
Source: Nat Commun. 2020 Jan 21;11:403. doi: 10.1038/s41467-020-14292-2 (PMC6972843; doi:10.1038/s41467-020-14292-2)
Supplement: Supplementary file 1 — Supplementary Information [file 41467_2020_14292_MOESM1_ESM.pdf]

Supplementary Information

Functionalization of Remote Unactivated sp^3 Carbon Enabled by Copper-Catalyzed Coupling of *O*-Acyloximes with Terminal Alkynes

Zhaodong Li,[1]† Rubén O. Torres-Ochoa,[2]† Qian Wang[2] and Jieping Zhu[2]*

Affiliations:

1. Department of Applied Chemistry, College of Material and Energy, South China Agricultural University, 510642 Guangzhou (China)

2. Laboratory of Synthesis and Natural Products, Institute of Chemical Sciences and Engineering, Ecole Polytechnique Fédérale de Lausanne, EPFL-SB-ISIC-LSPN, BCH5304, CH-1015 Lausanne (Switzerland)

E-mail: jieping.zhu@epfl.ch

Supplementary Figures

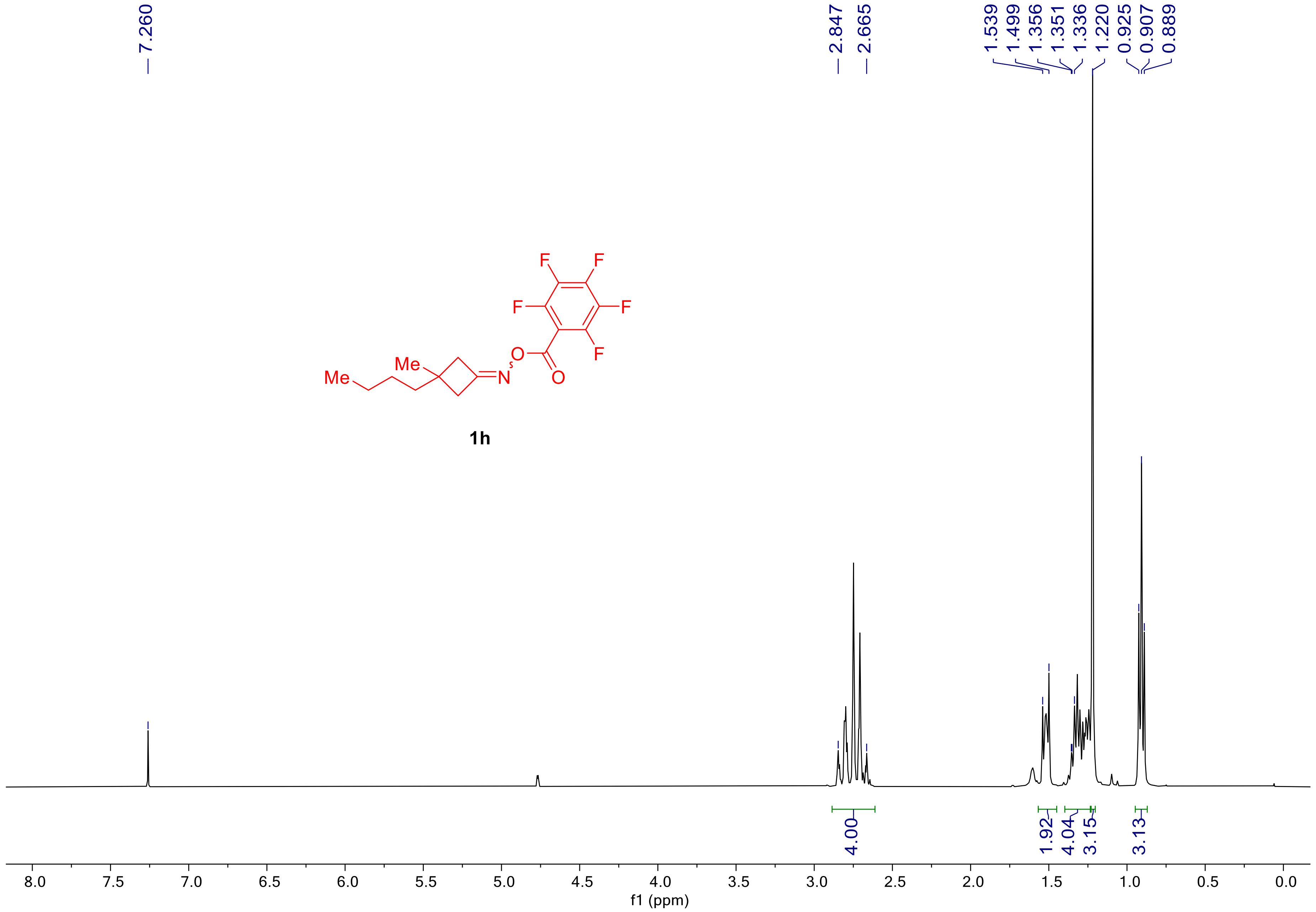

Supplementary Figure 1. ^{1}H NMR spectrum of 1h

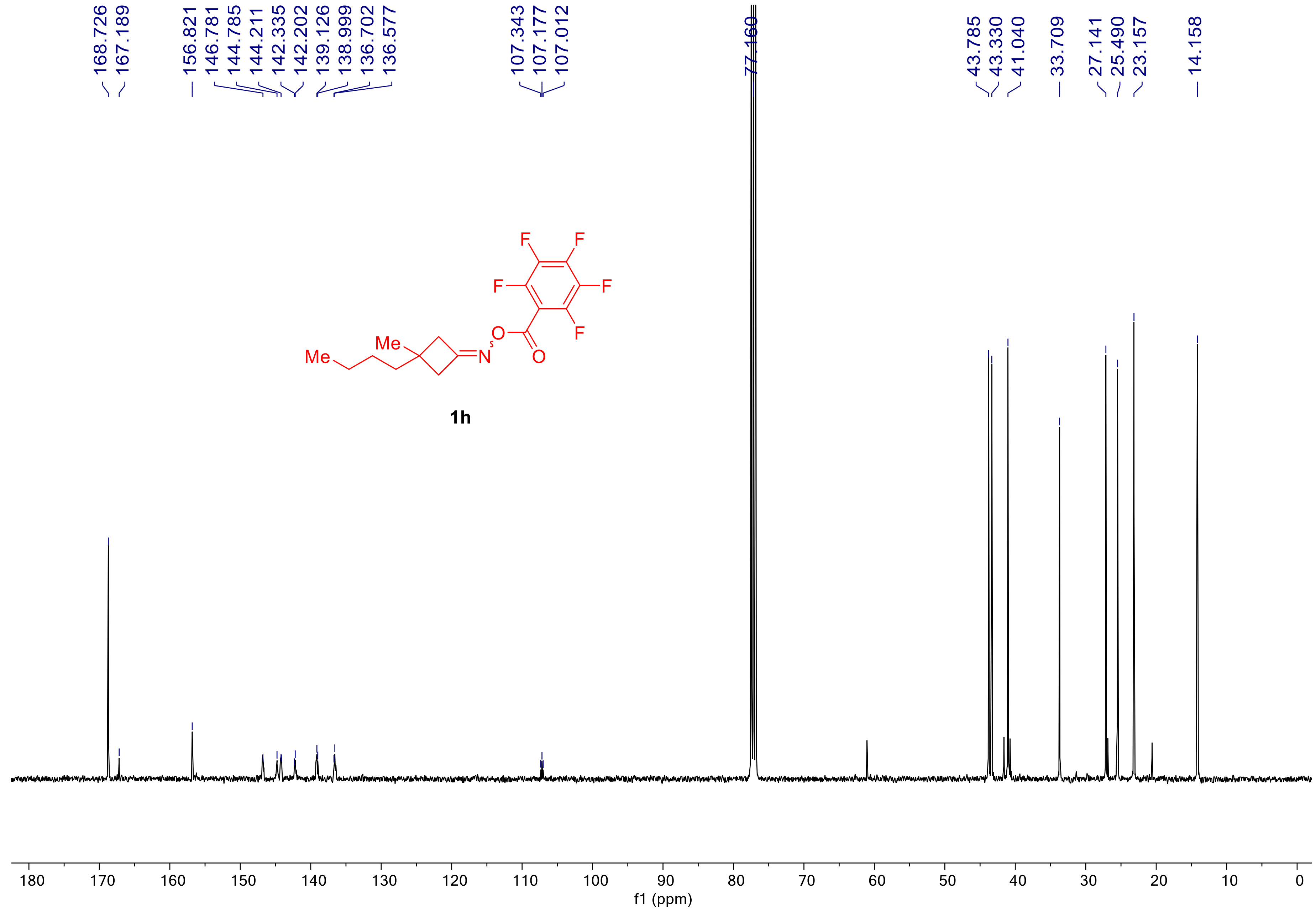

Supplementary Figure 2. ^{13}C NMR spectrum of 1h

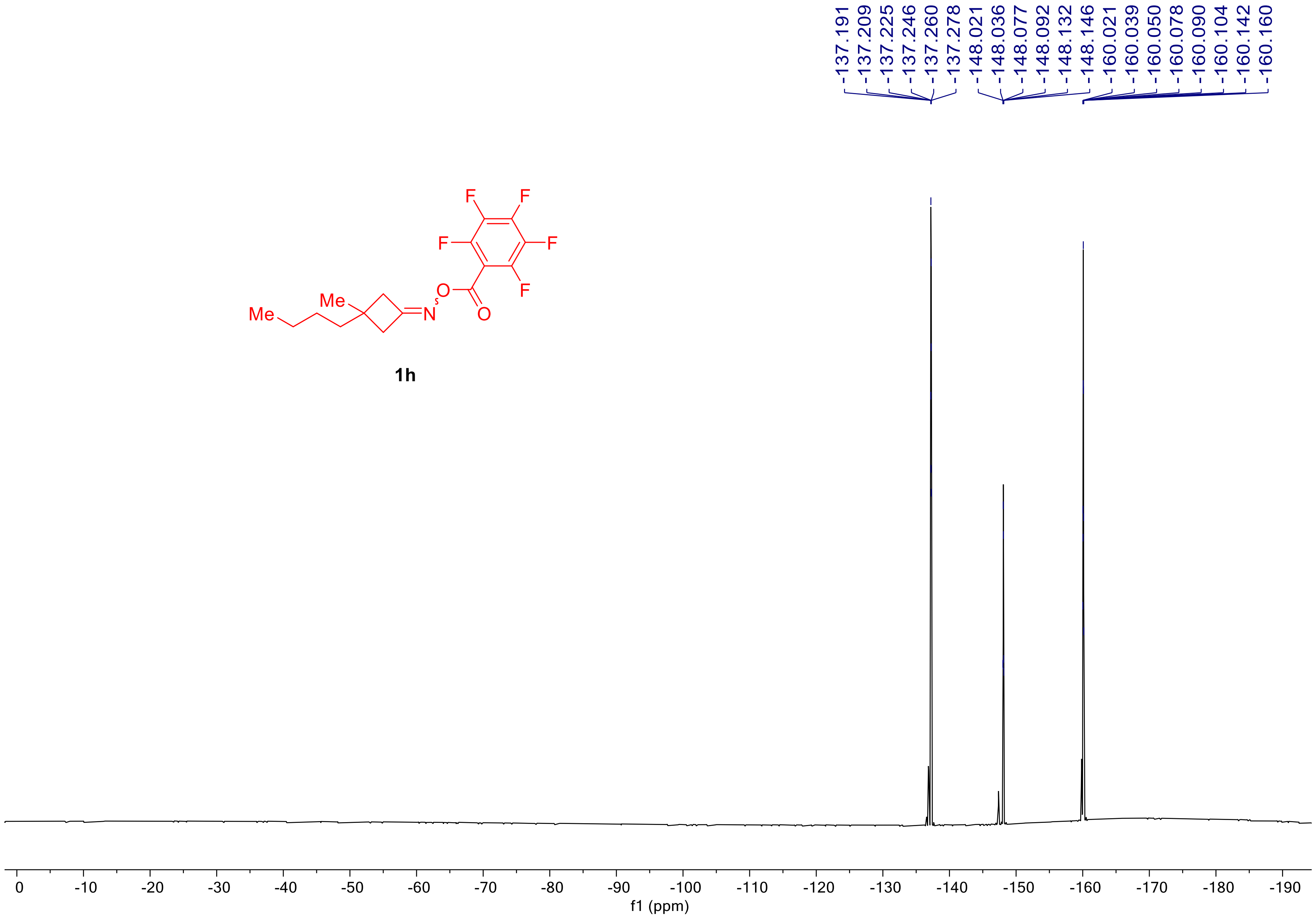

Supplementary Figure 3. ^{19}F NMR spectrum of 1h

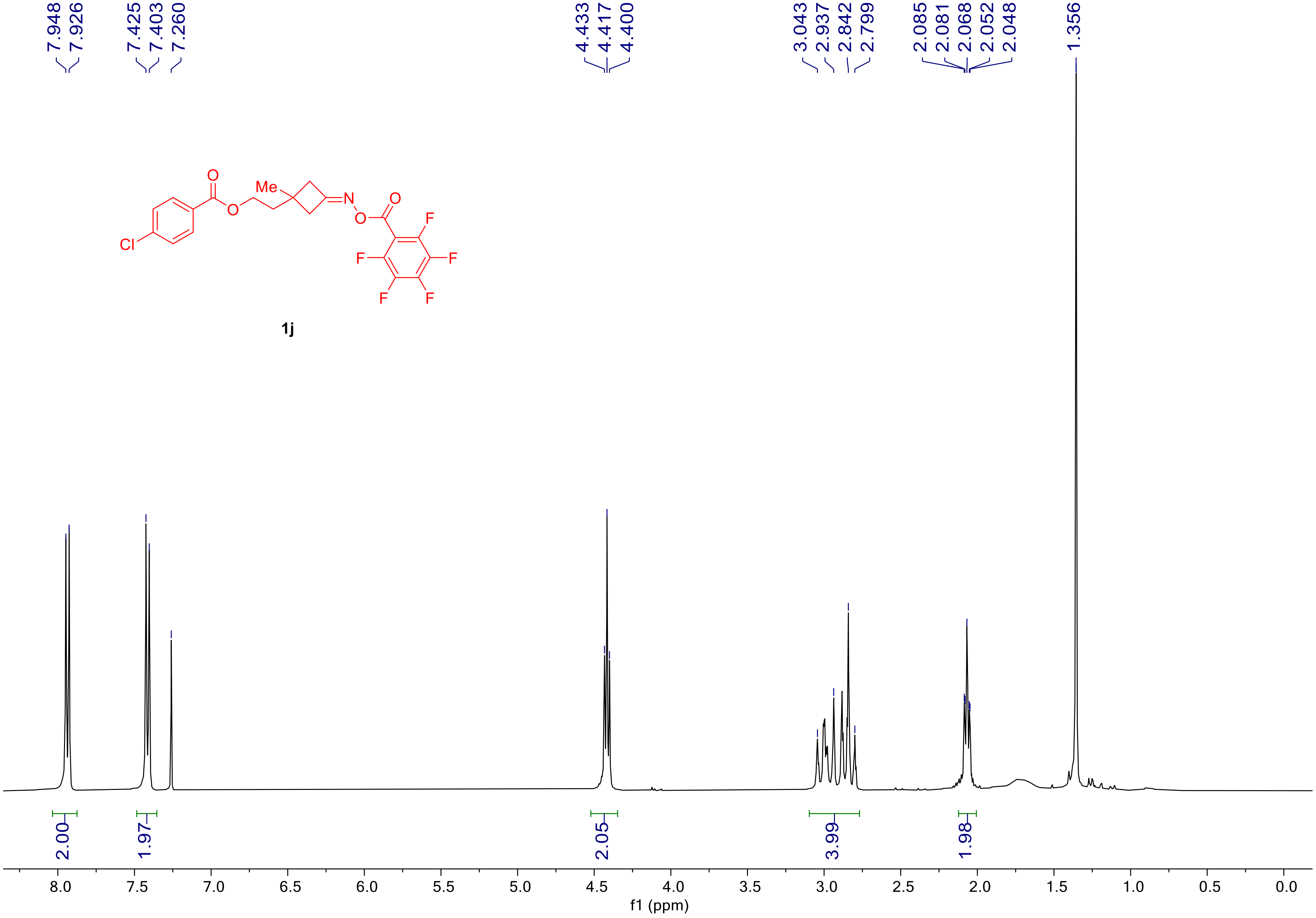

Supplementary Figure 4. ^{1}H NMR spectrum of 1j

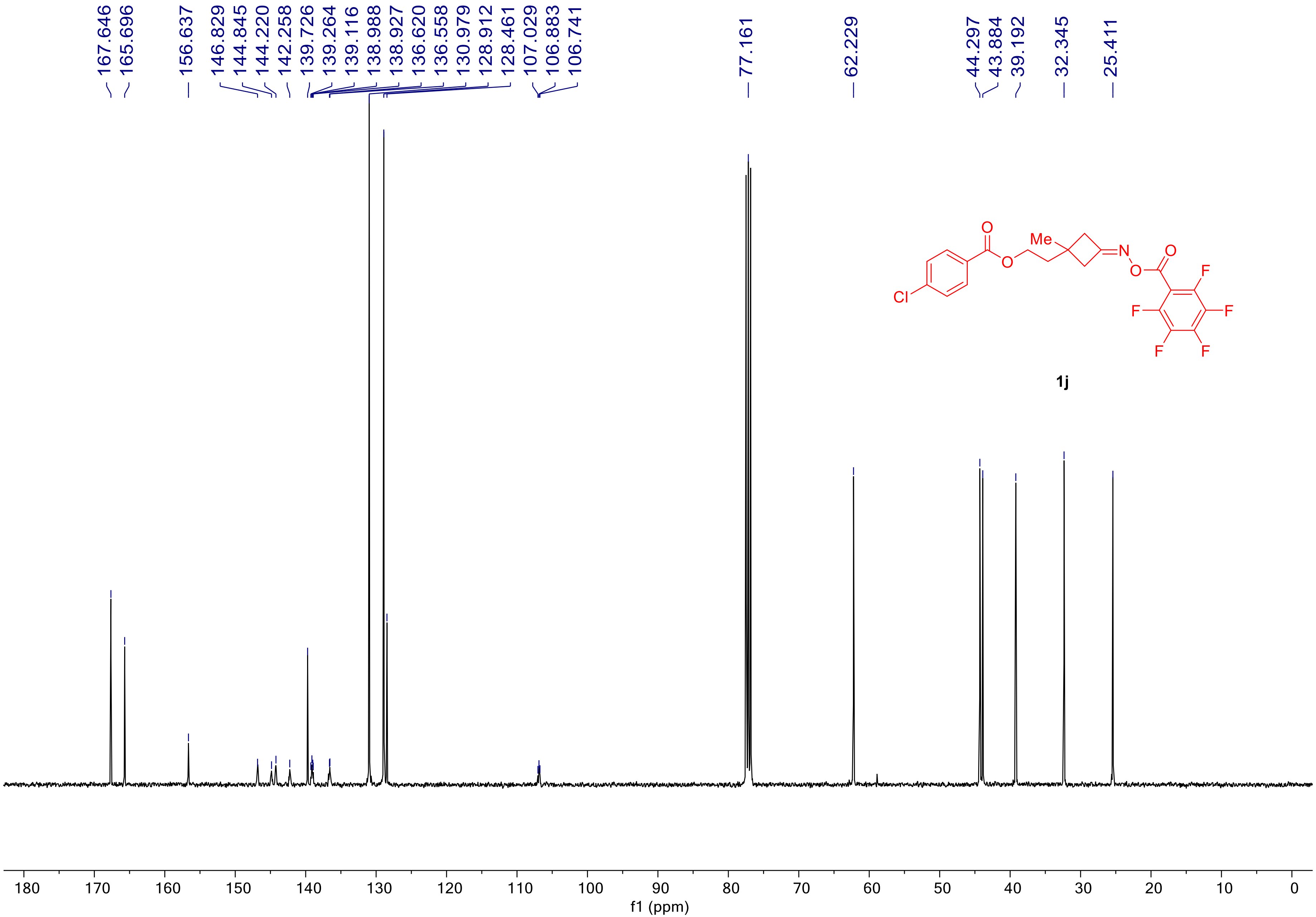

Supplementary Figure 5. ^{13}C NMR spectrum of 1j

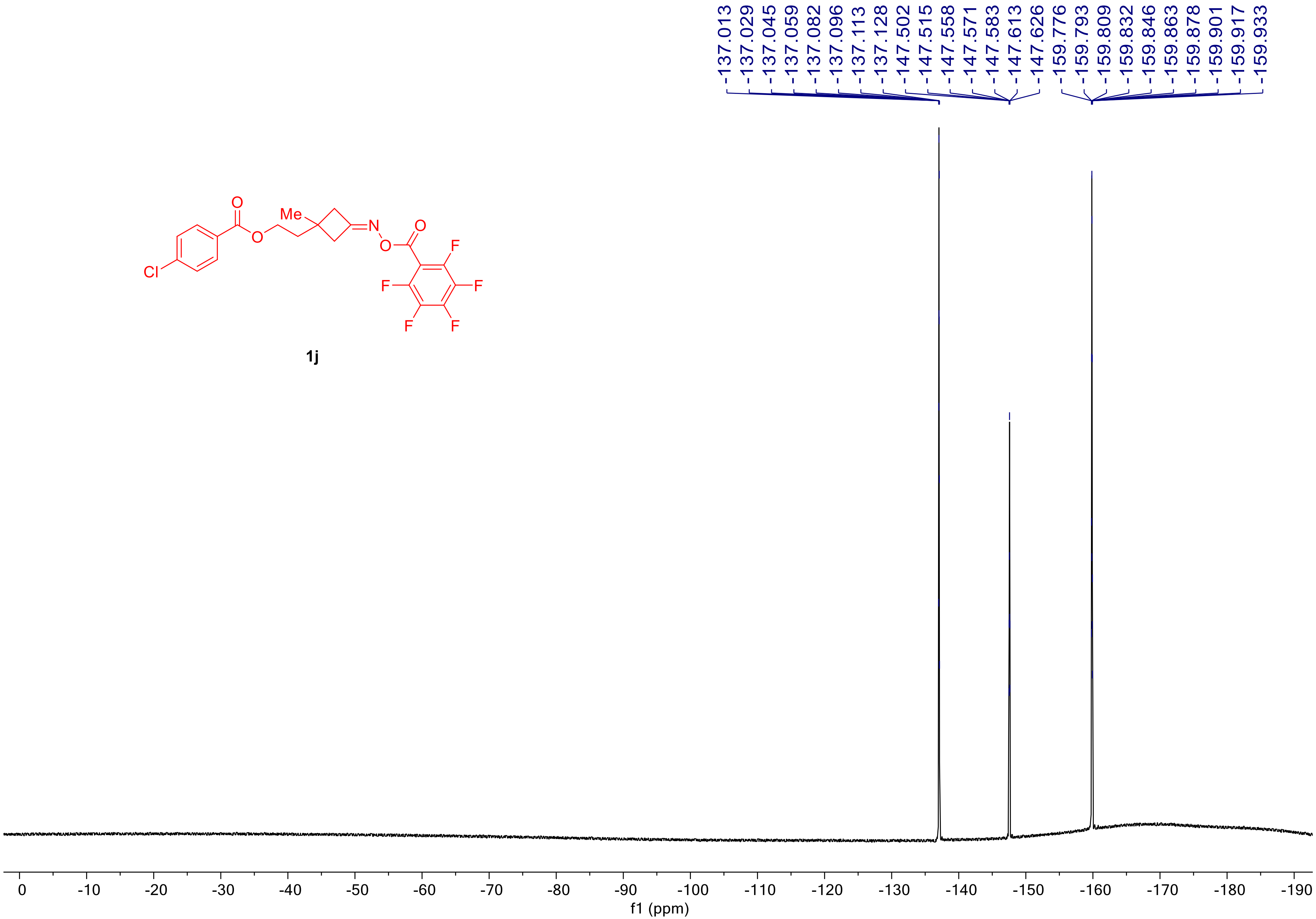

Supplementary Figure 6. ^{19}F NMR spectrum of 1j

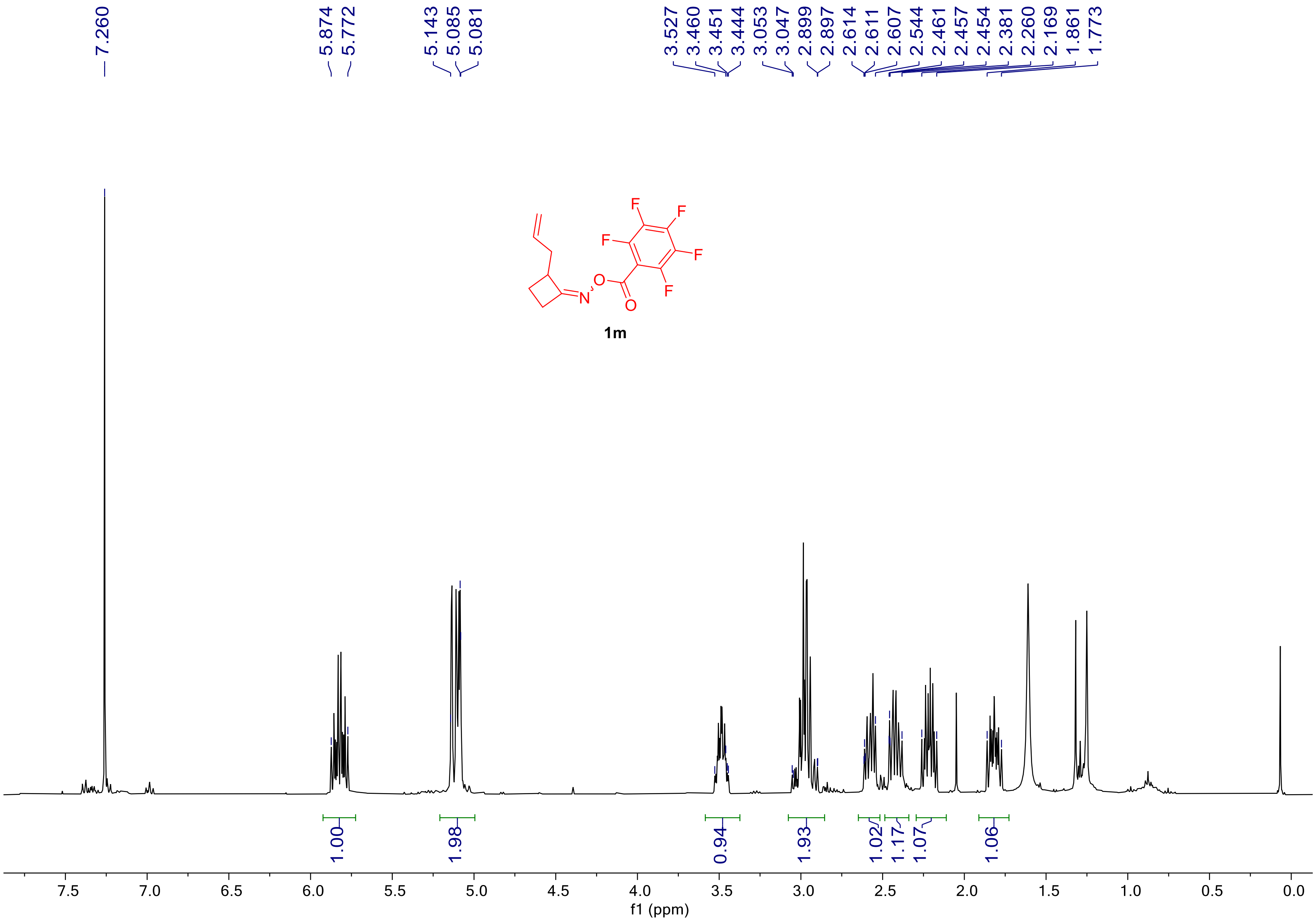

Supplementary Figure 7. ^{1}H NMR spectrum of 1m

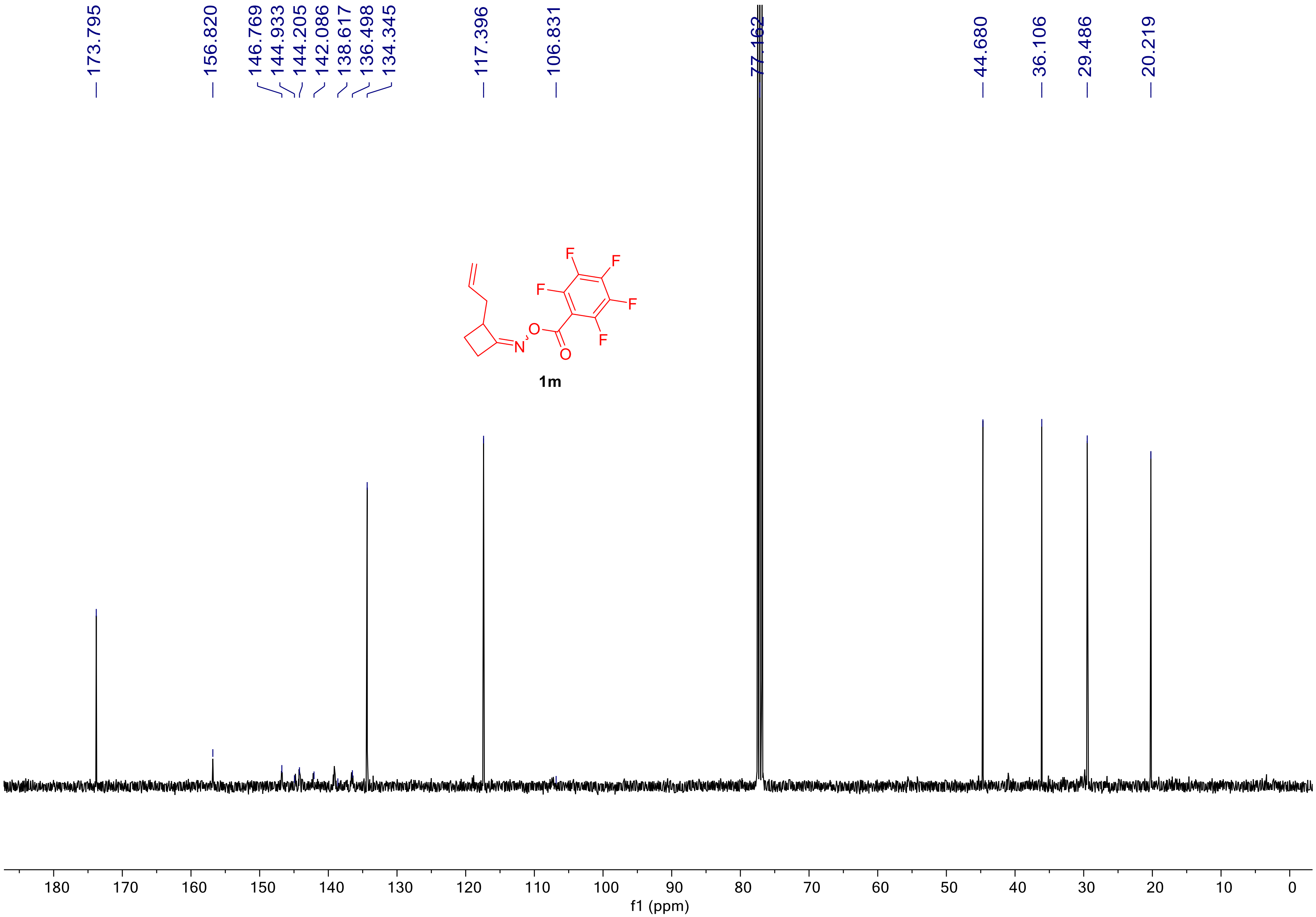

Supplementary Figure 8. ^{13}C NMR spectrum of 1m

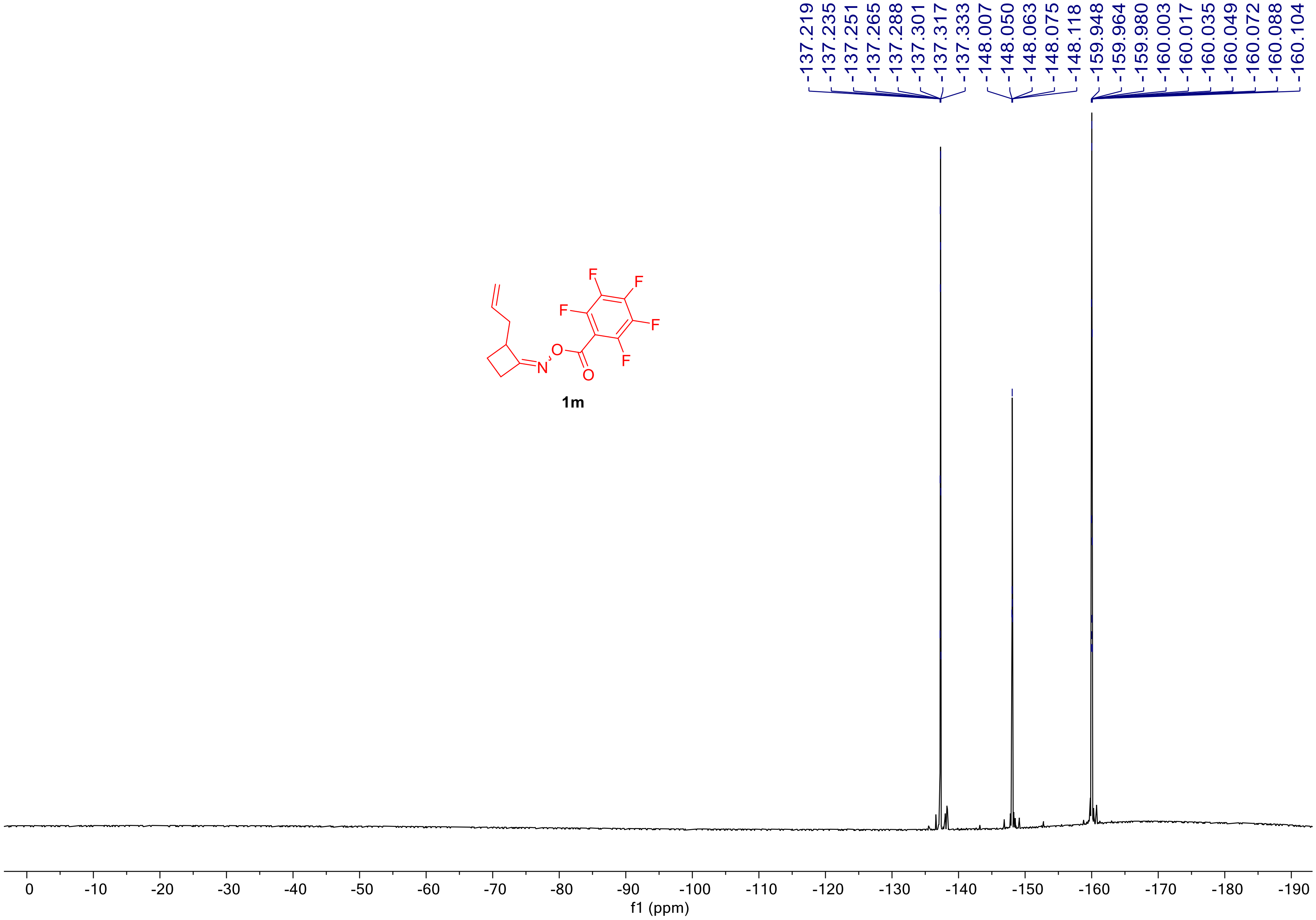

Supplementary Figure 9. ^{19}F NMR spectrum of 1m

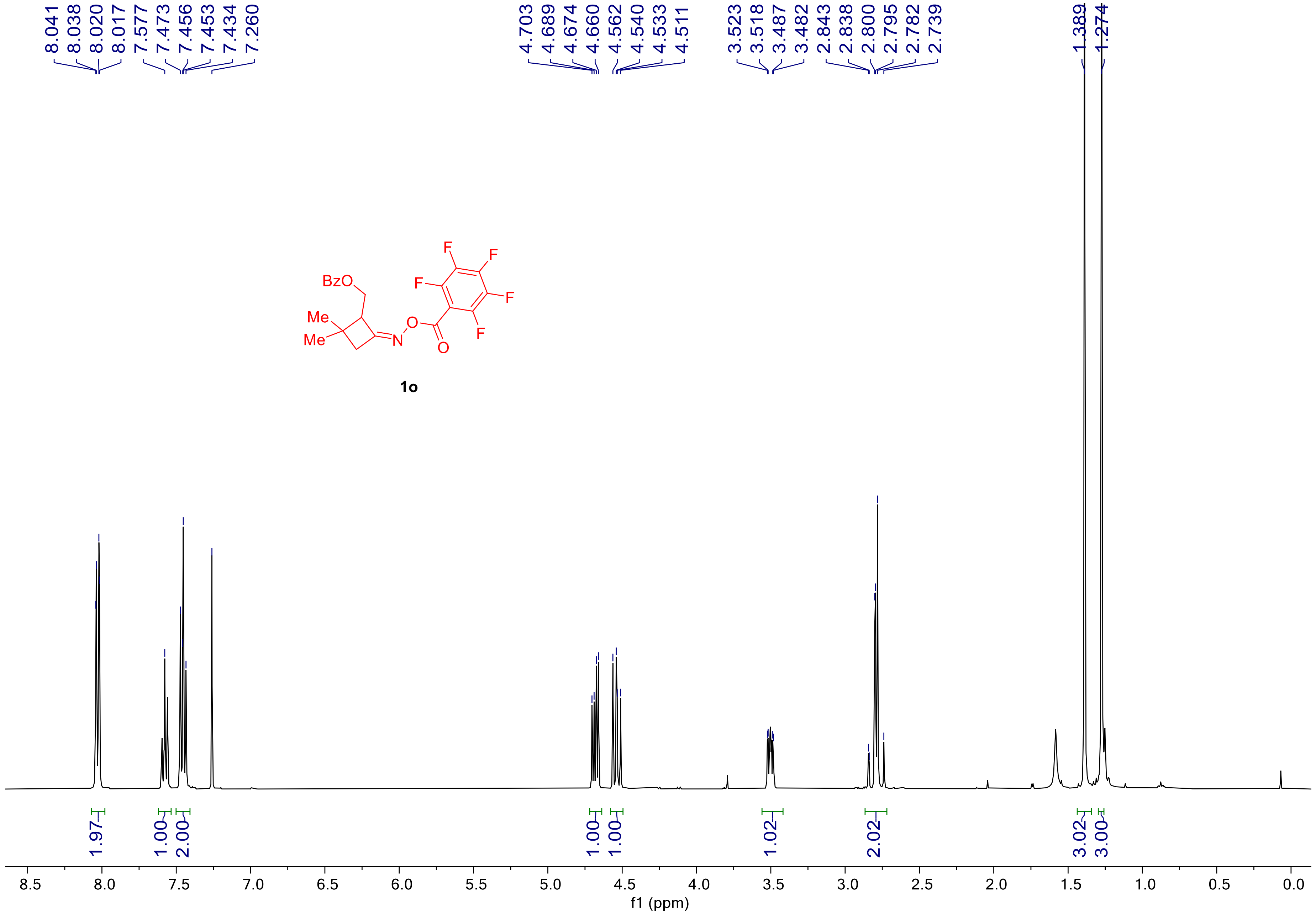

Supplementary Figure 10. ^{1}**H NMR spectrum of 1o**

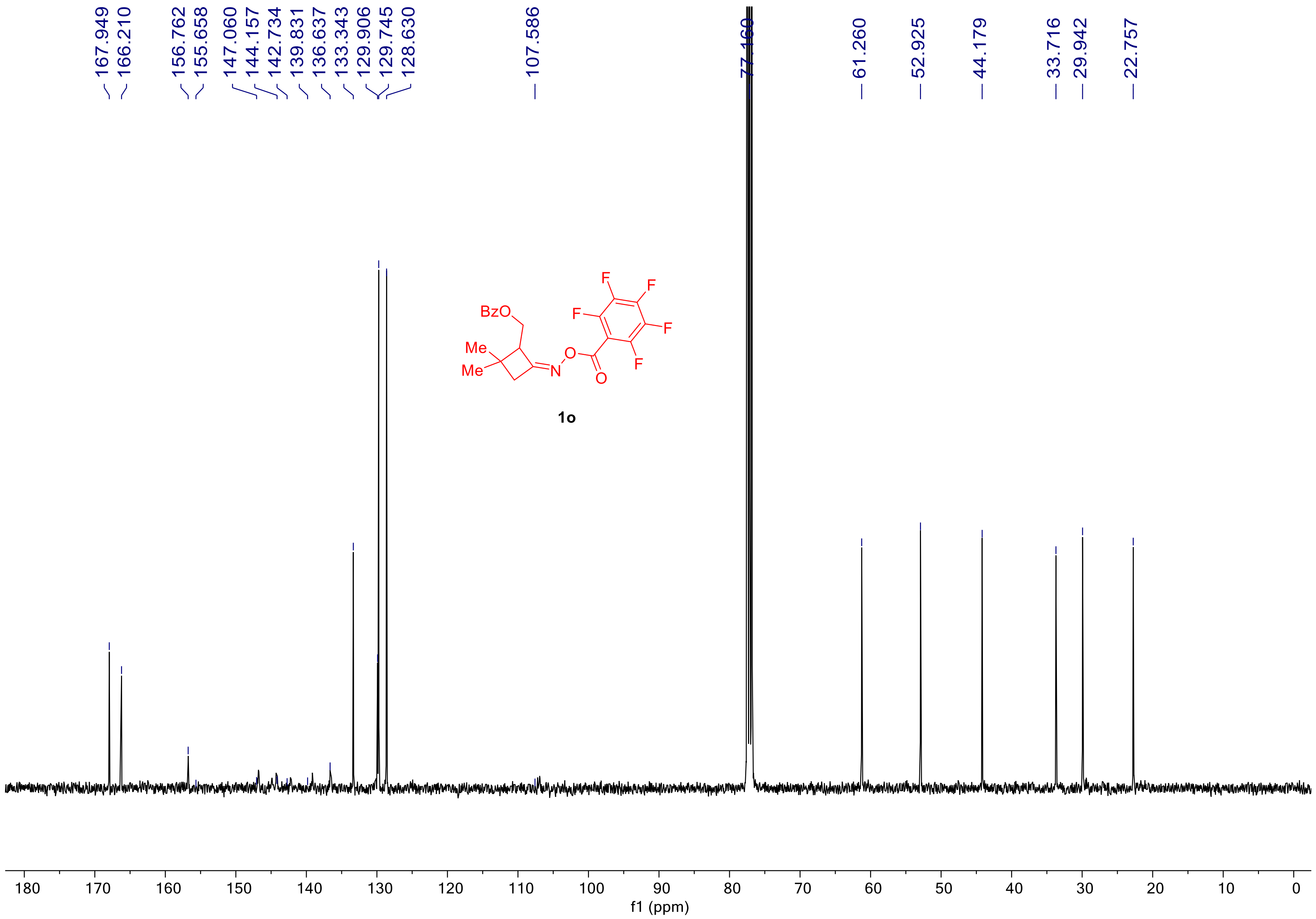

Supplementary Figure 11. ^{13}C **NMR spectrum of 1o**

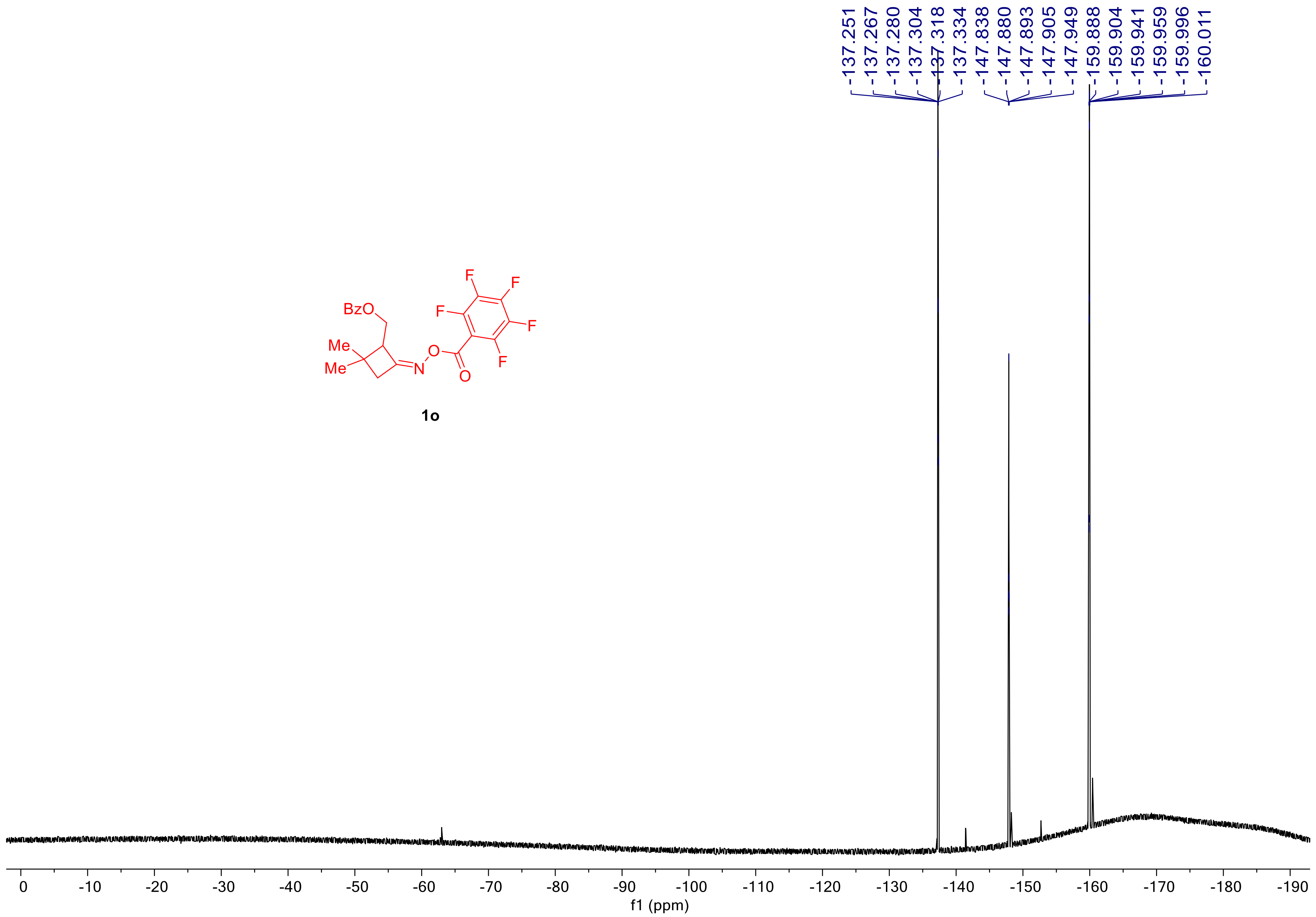

Supplementary Figure 12. ^{19}**F NMR spectrum of 1o**

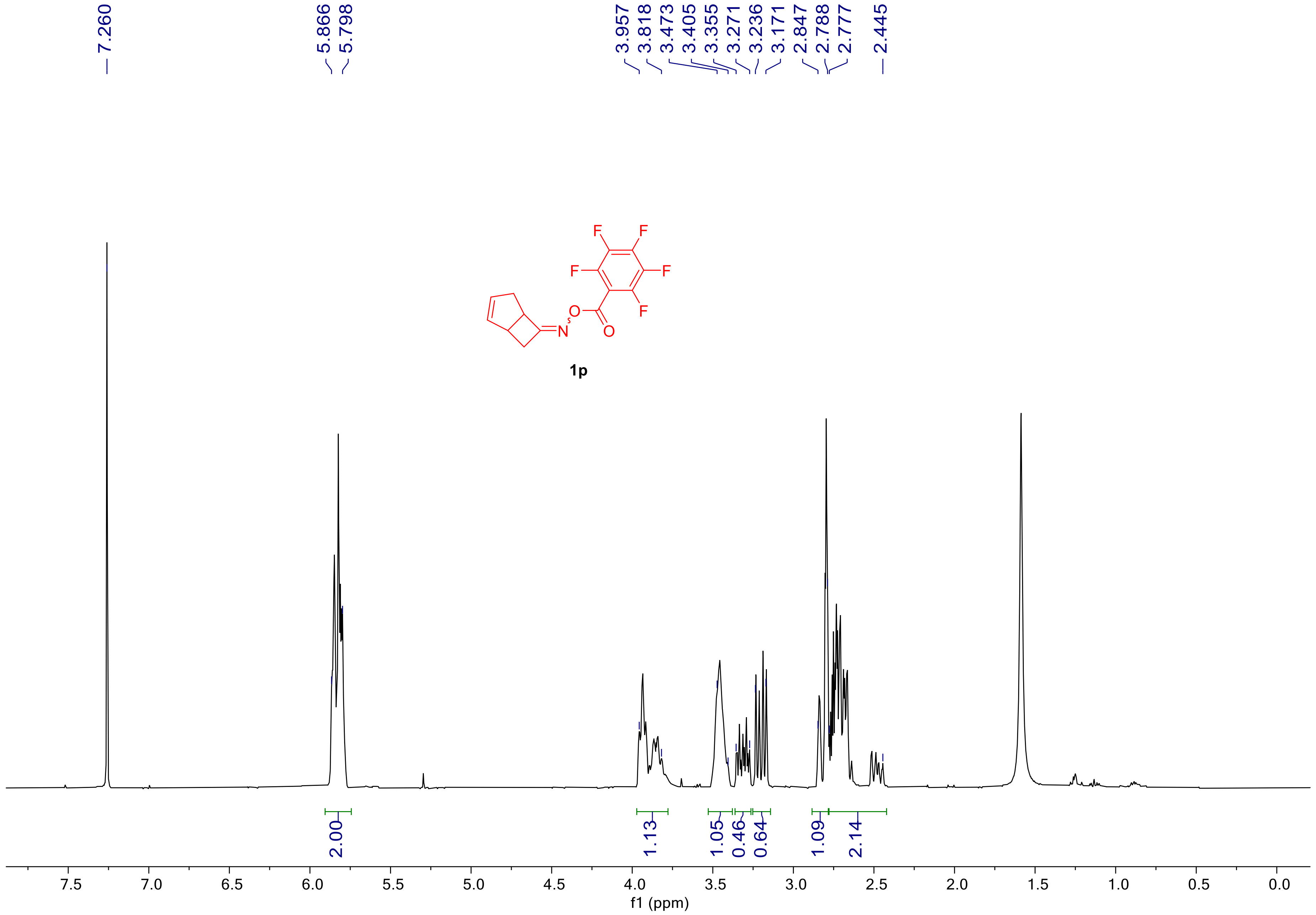

Supplementary Figure 13. ^{1}H NMR spectrum of 1p

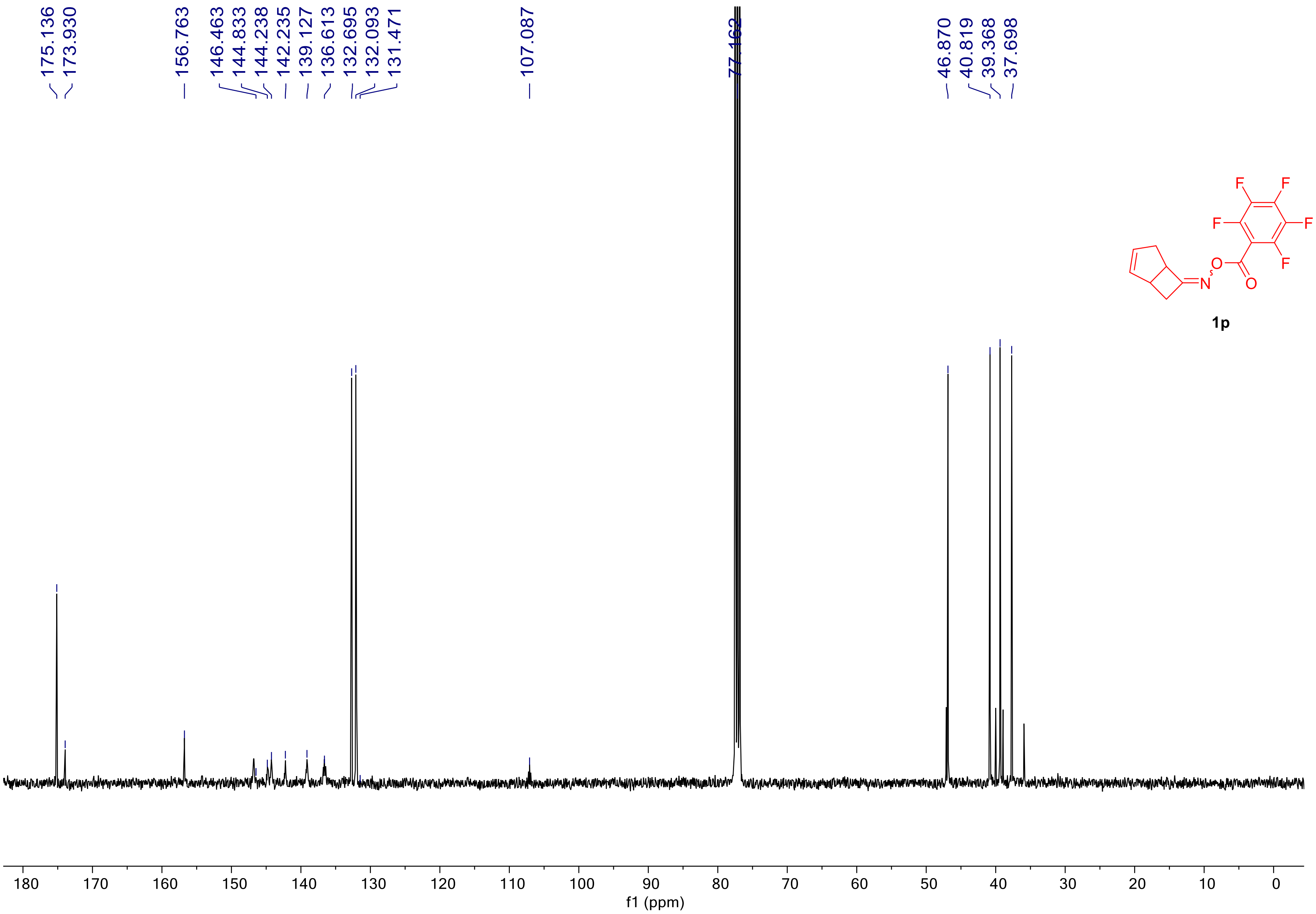

Supplementary Figure 14. ^{13}C NMR spectrum of 1p

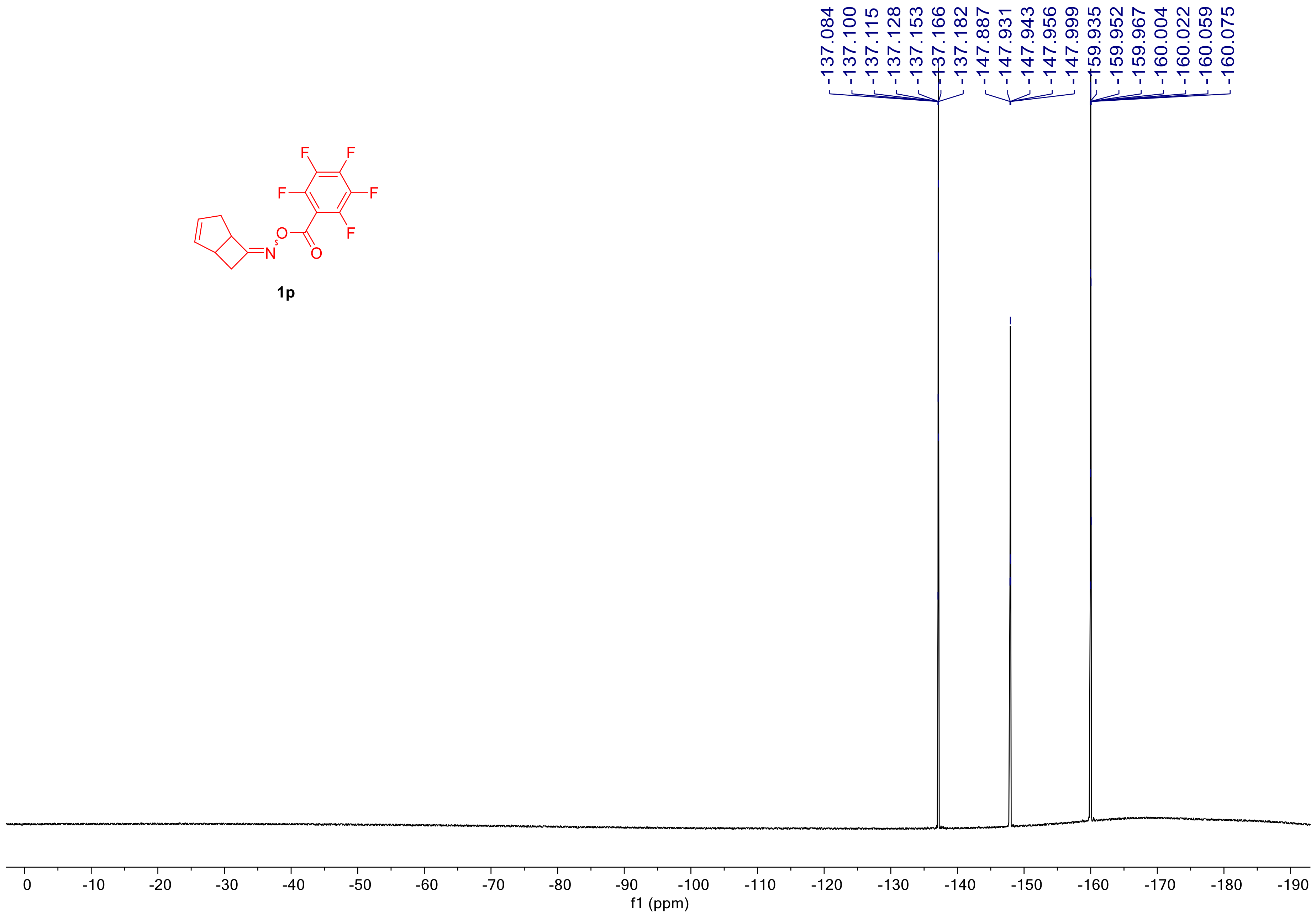

Supplementary Figure 15. ^{19}F NMR spectrum of 1p

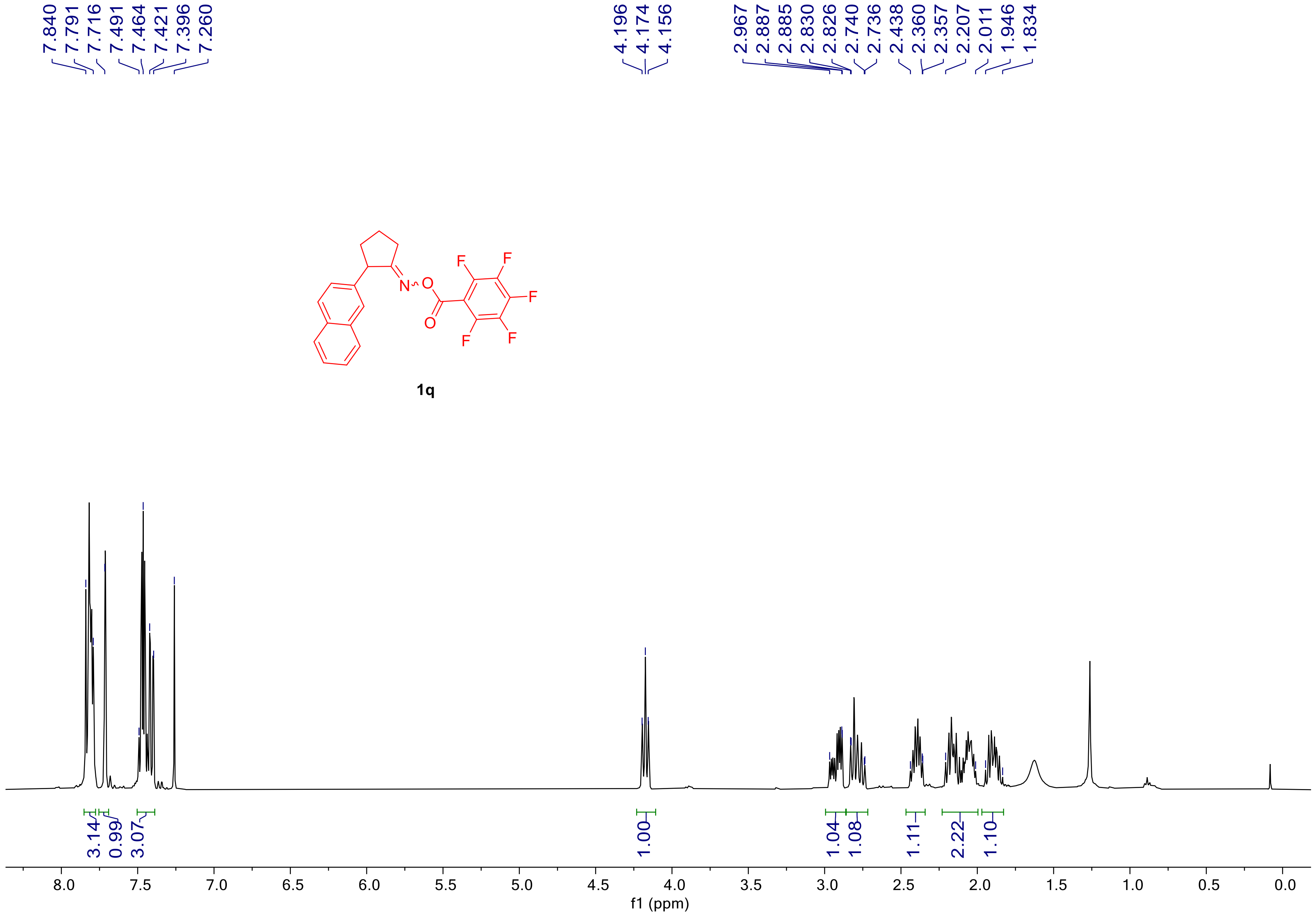

Supplementary Figure 16. ^{1}H **NMR spectrum of 1q**

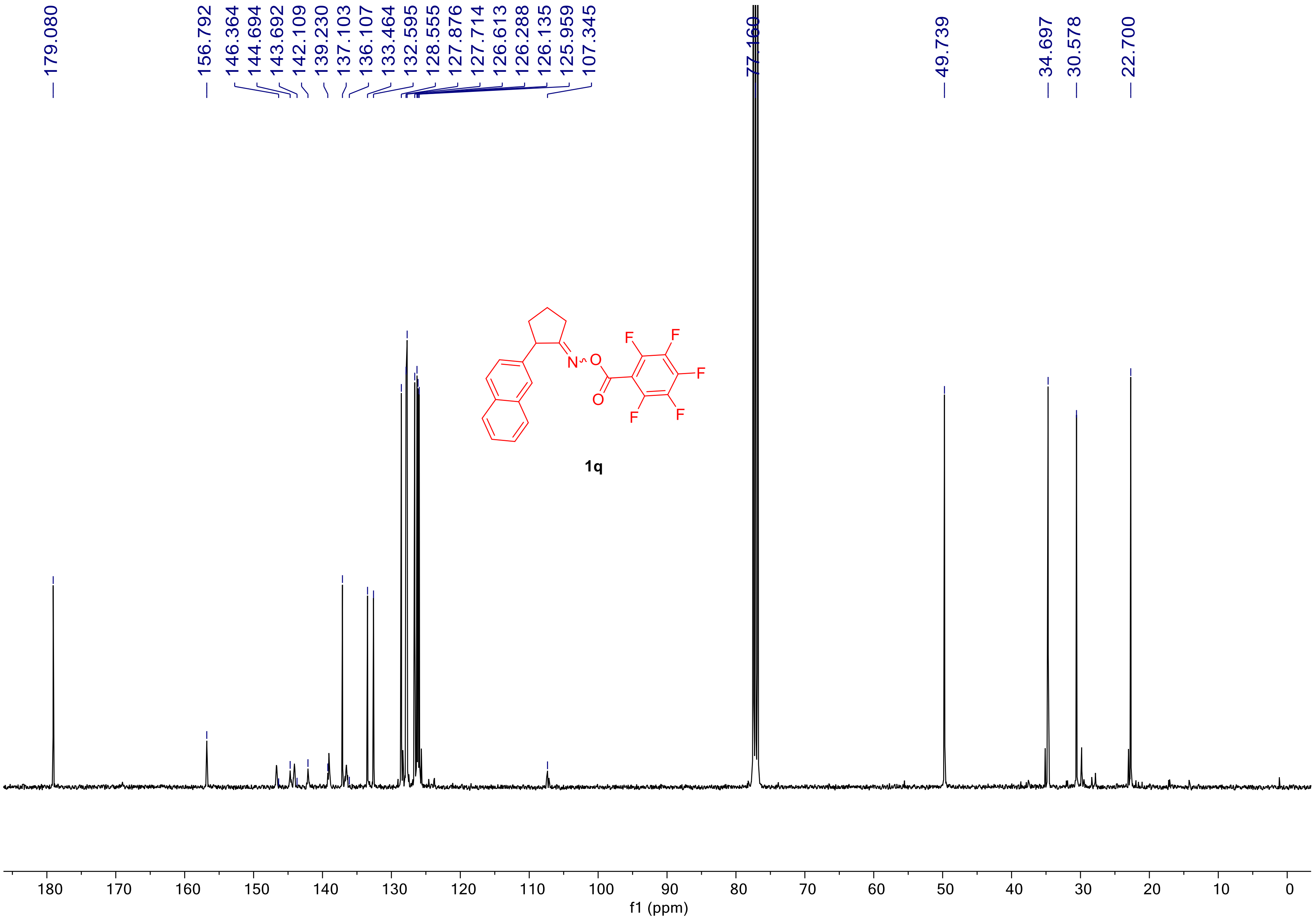

Supplementary Figure 17. ^{13}C NMR spectrum of 1q

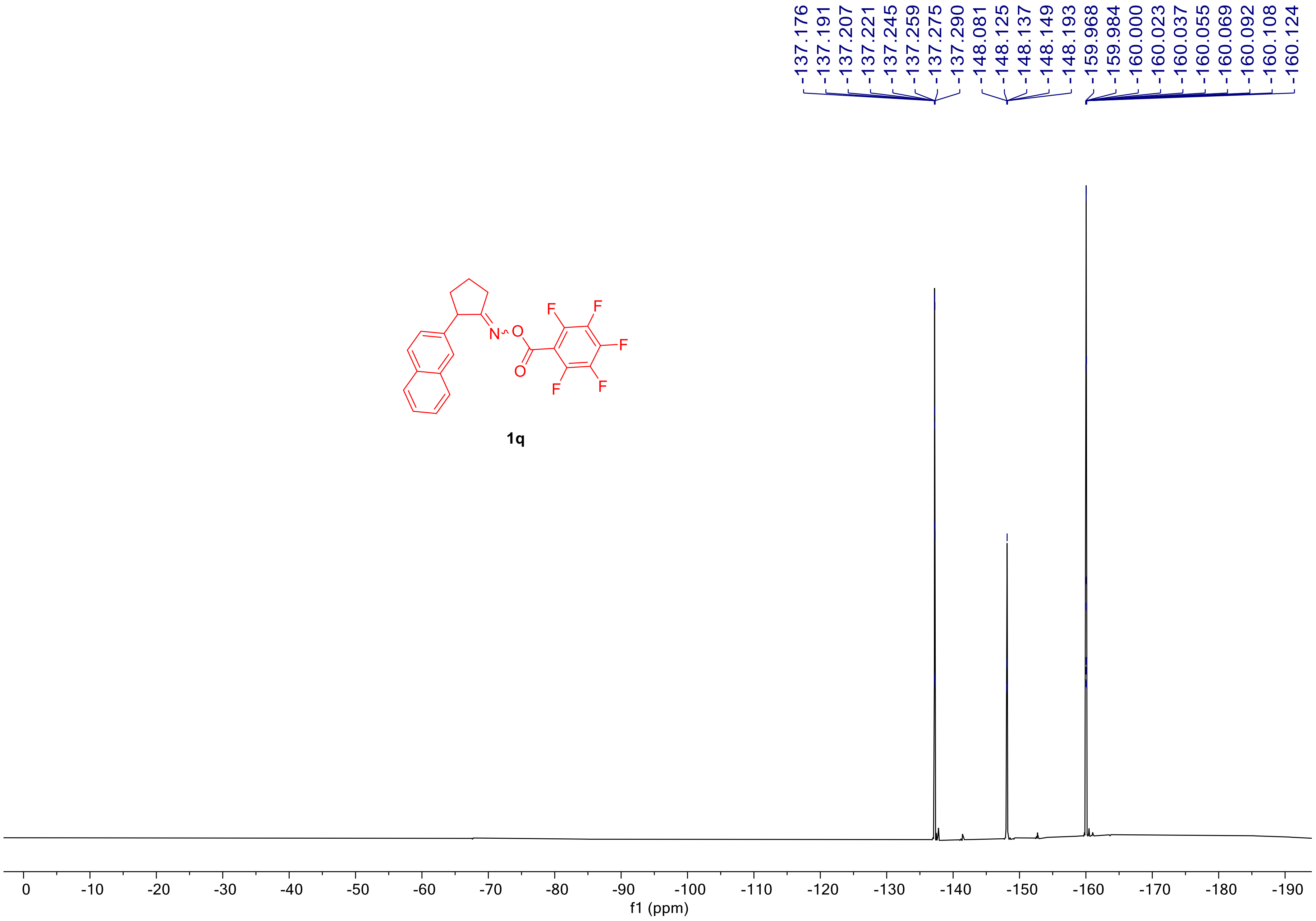

Supplementary Figure 18. ^{19}**F NMR spectrum of 1q**

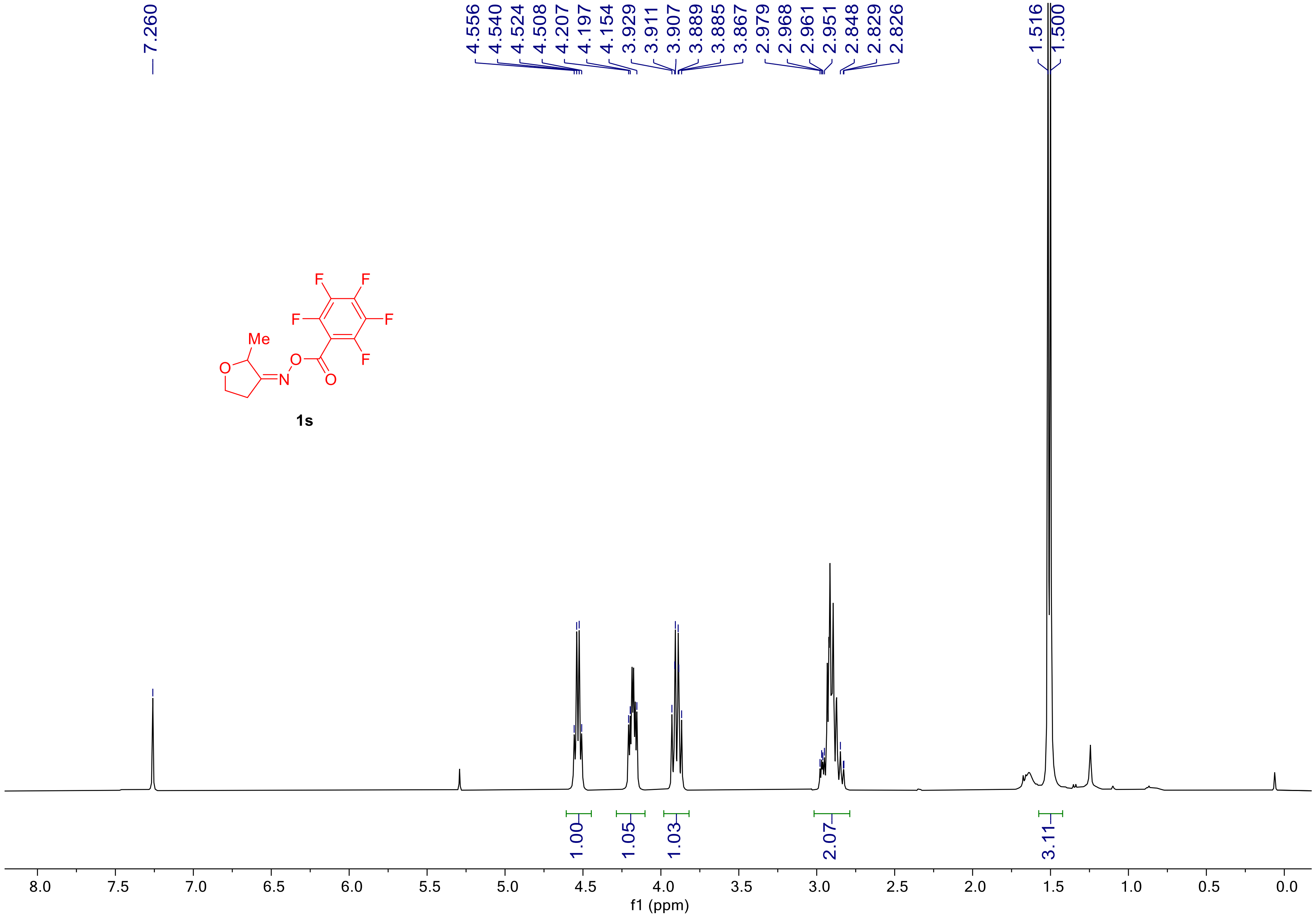

Supplementary Figure 19. ^{1}**H NMR spectrum of 1s**

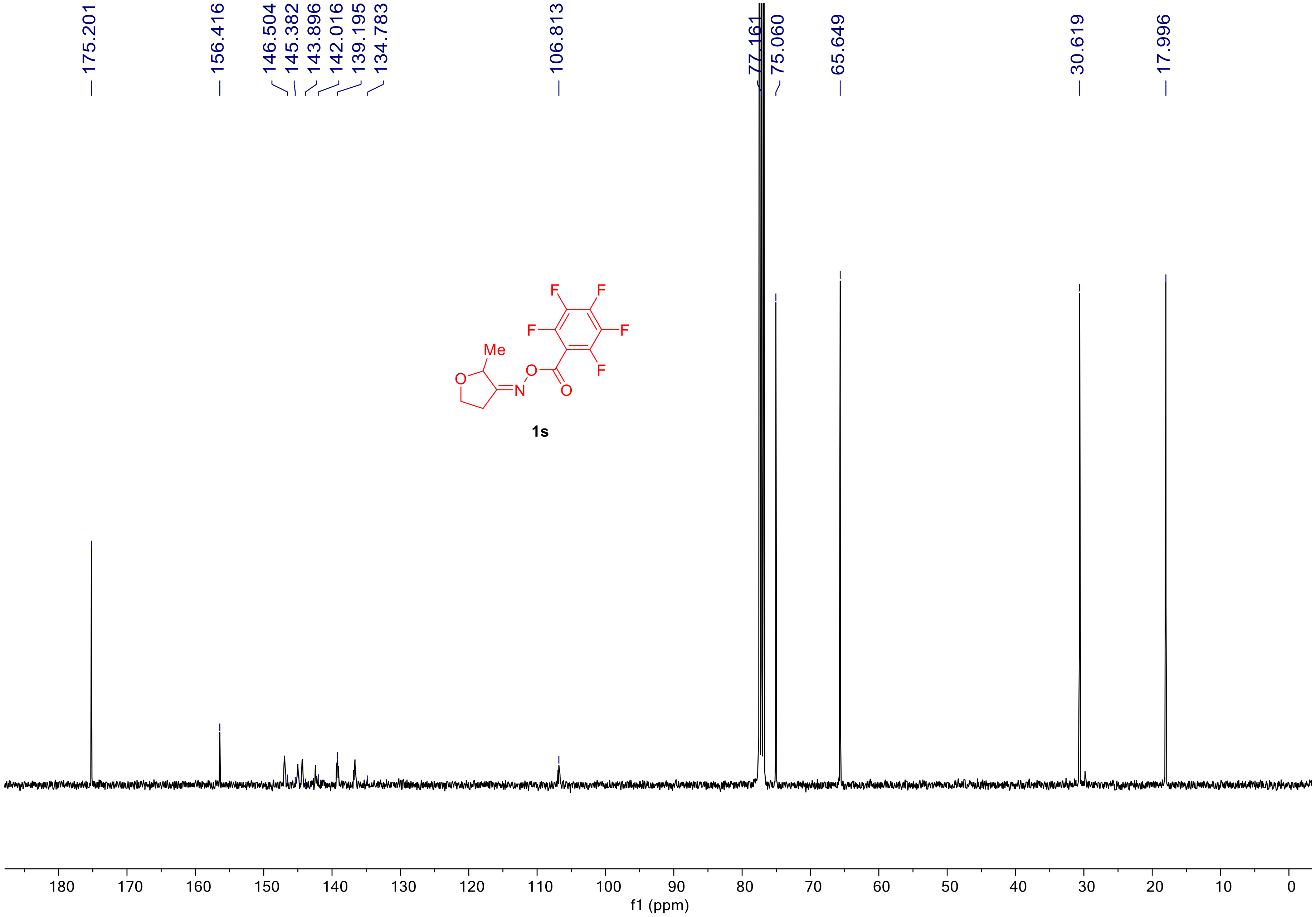

Supplementary Figure 20. ^{13}C NMR spectrum of 1s

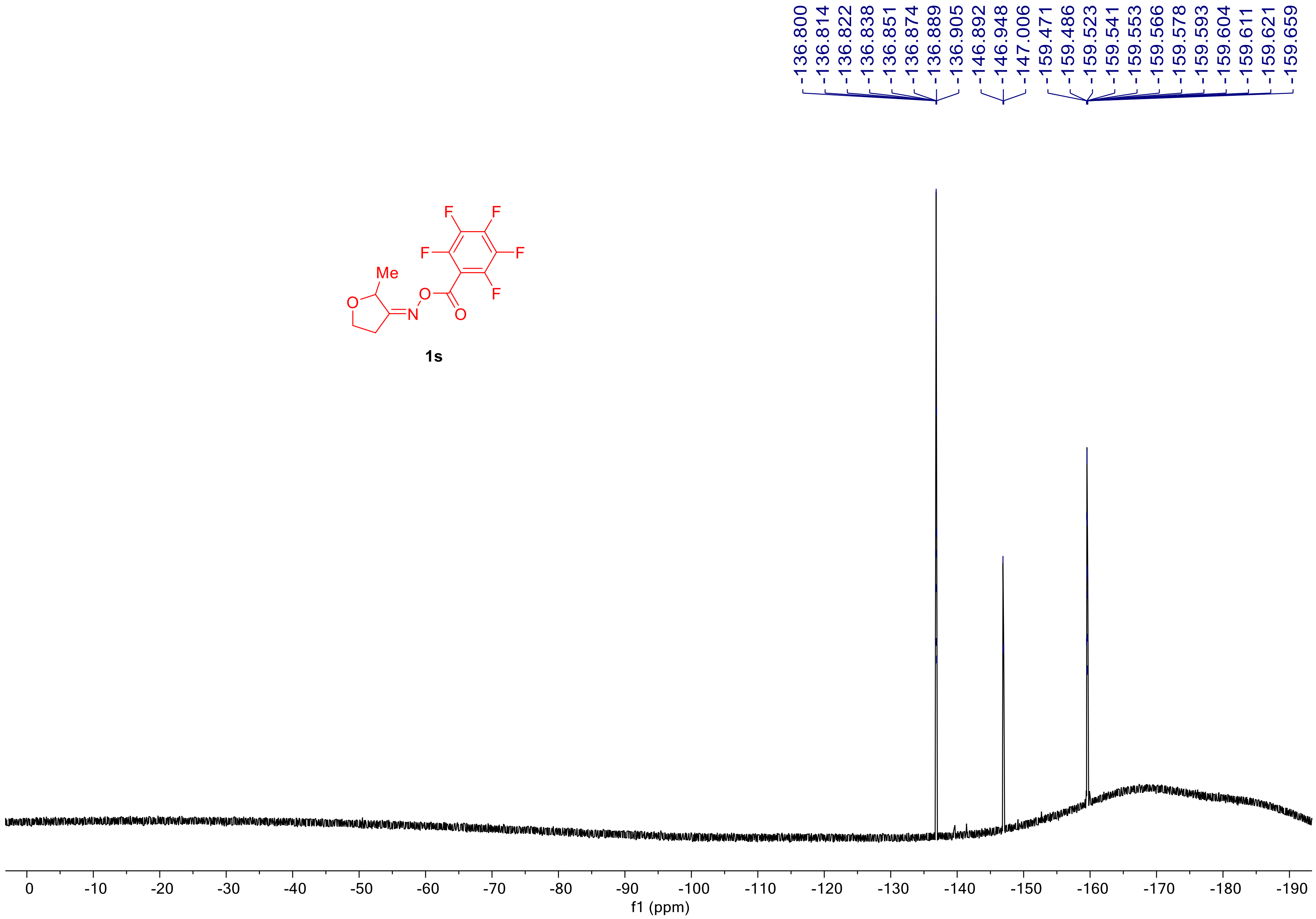

Supplementary Figure 21. ^{19}F NMR spectrum of 1s

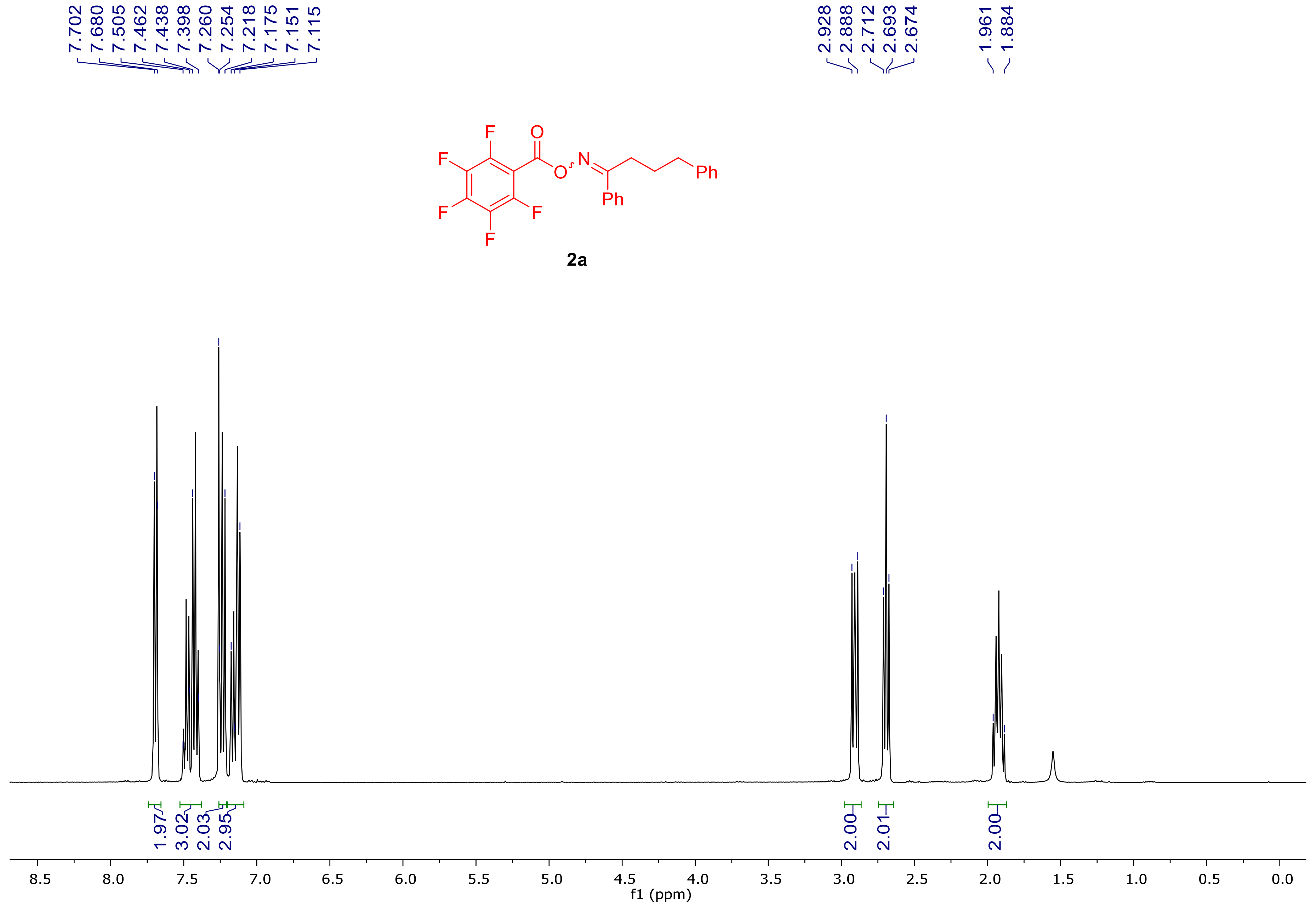

Supplementary Figure 22. ^{1}H NMR spectrum of 2a

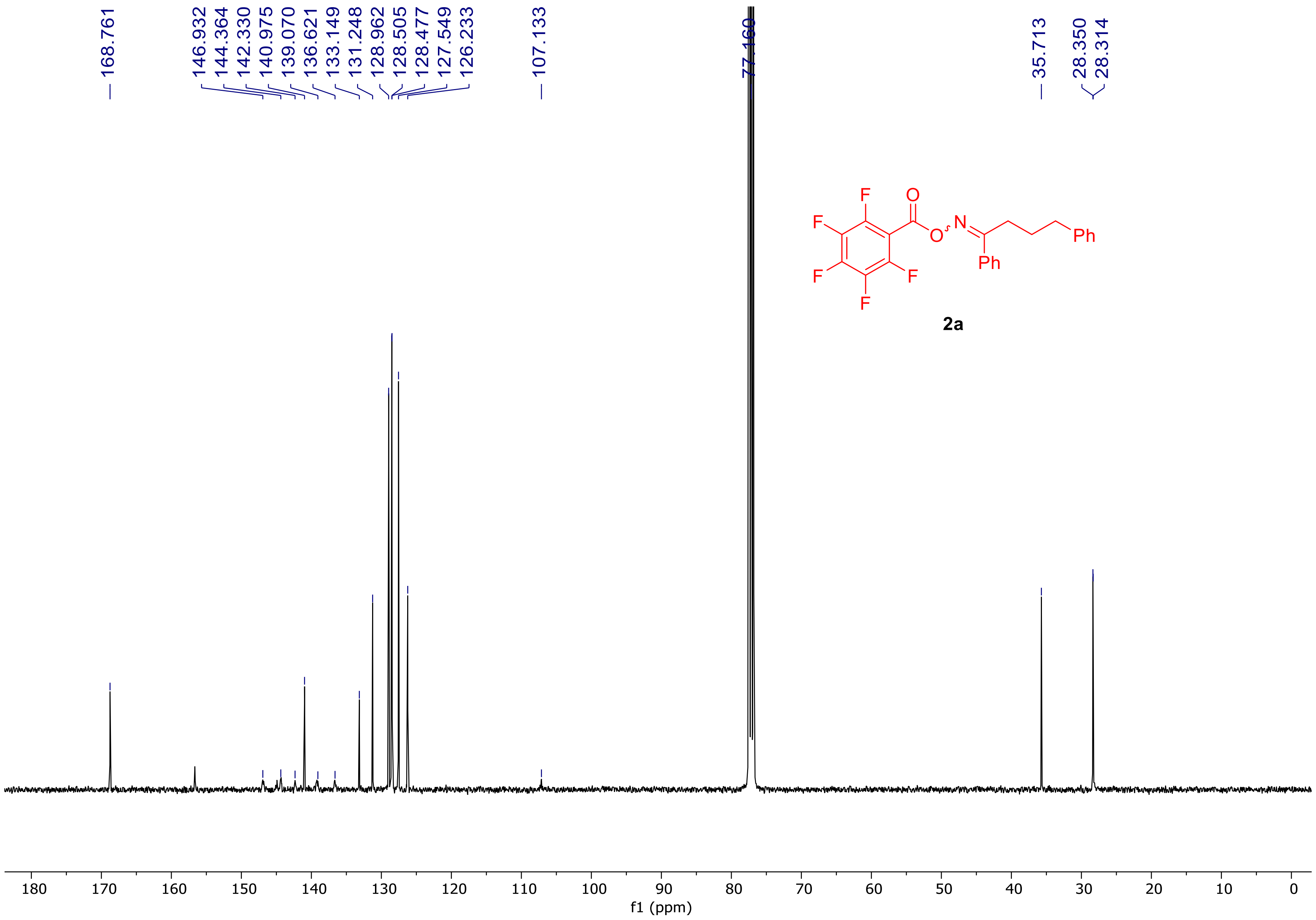

Supplementary Figure 23. ^{13}**C NMR spectrum of 2a**

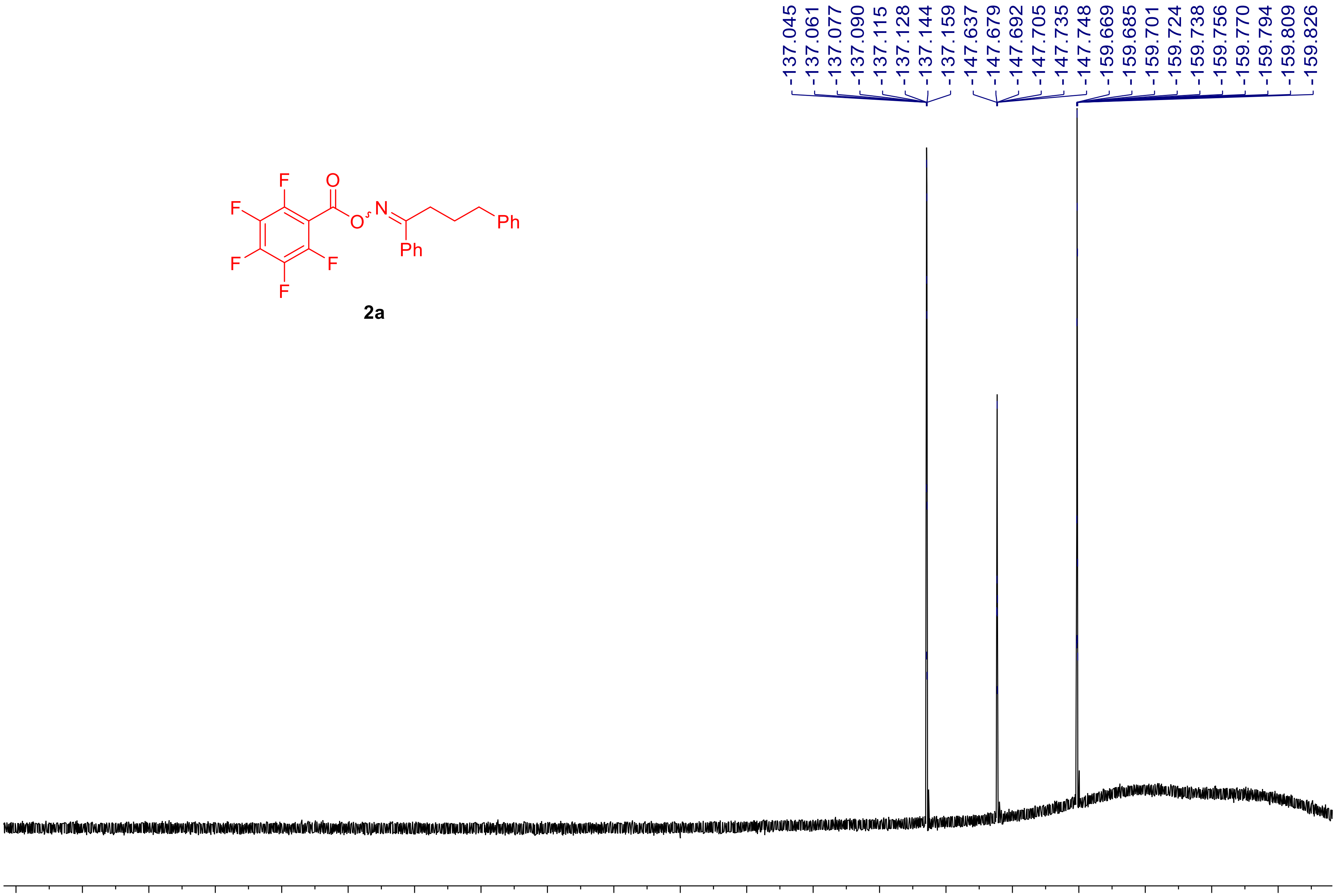

Supplementary Figure 24. ^{19}F NMR spectrum of 2a

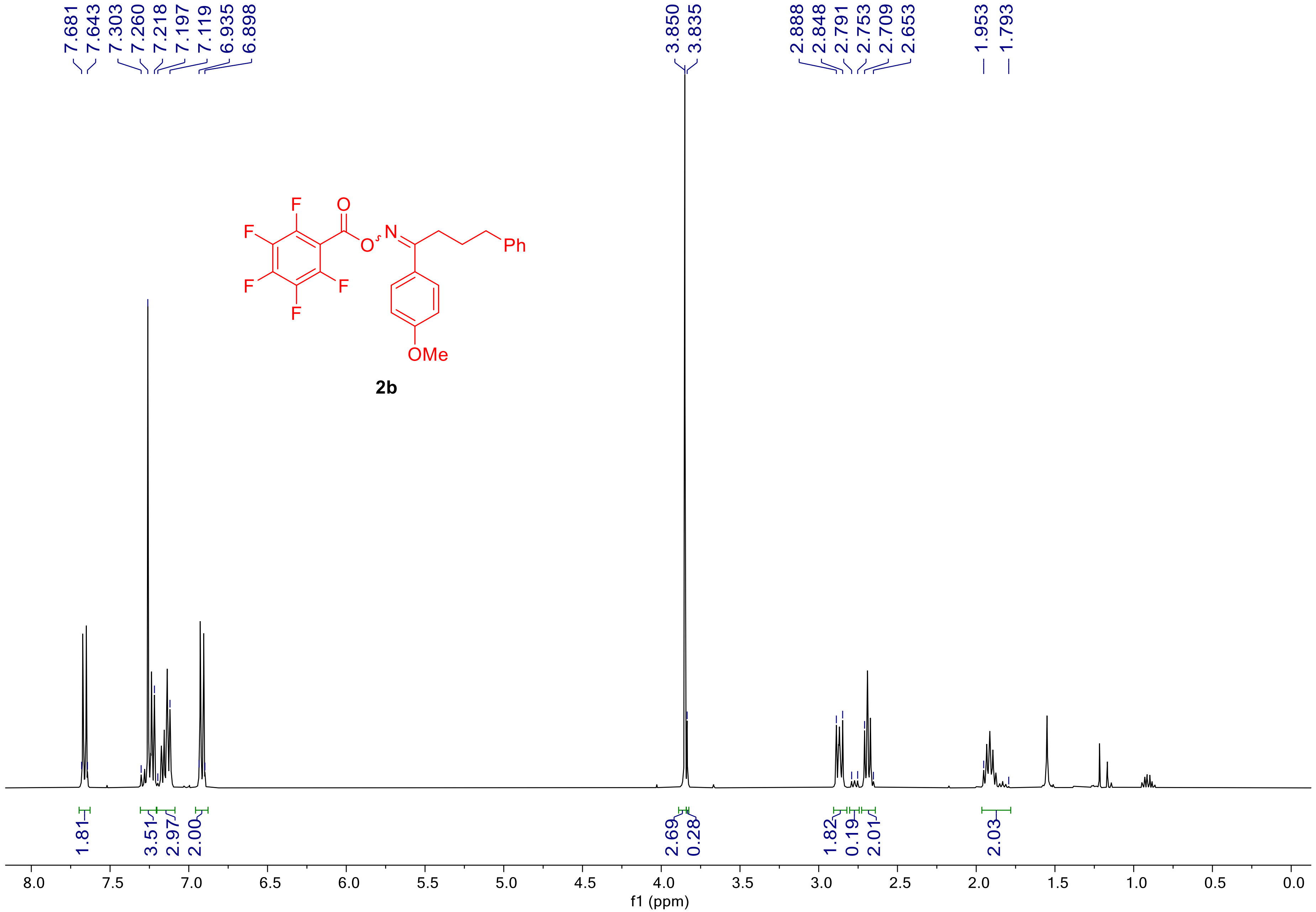

Supplementary Figure 25. ^{1}H NMR spectrum of 2b

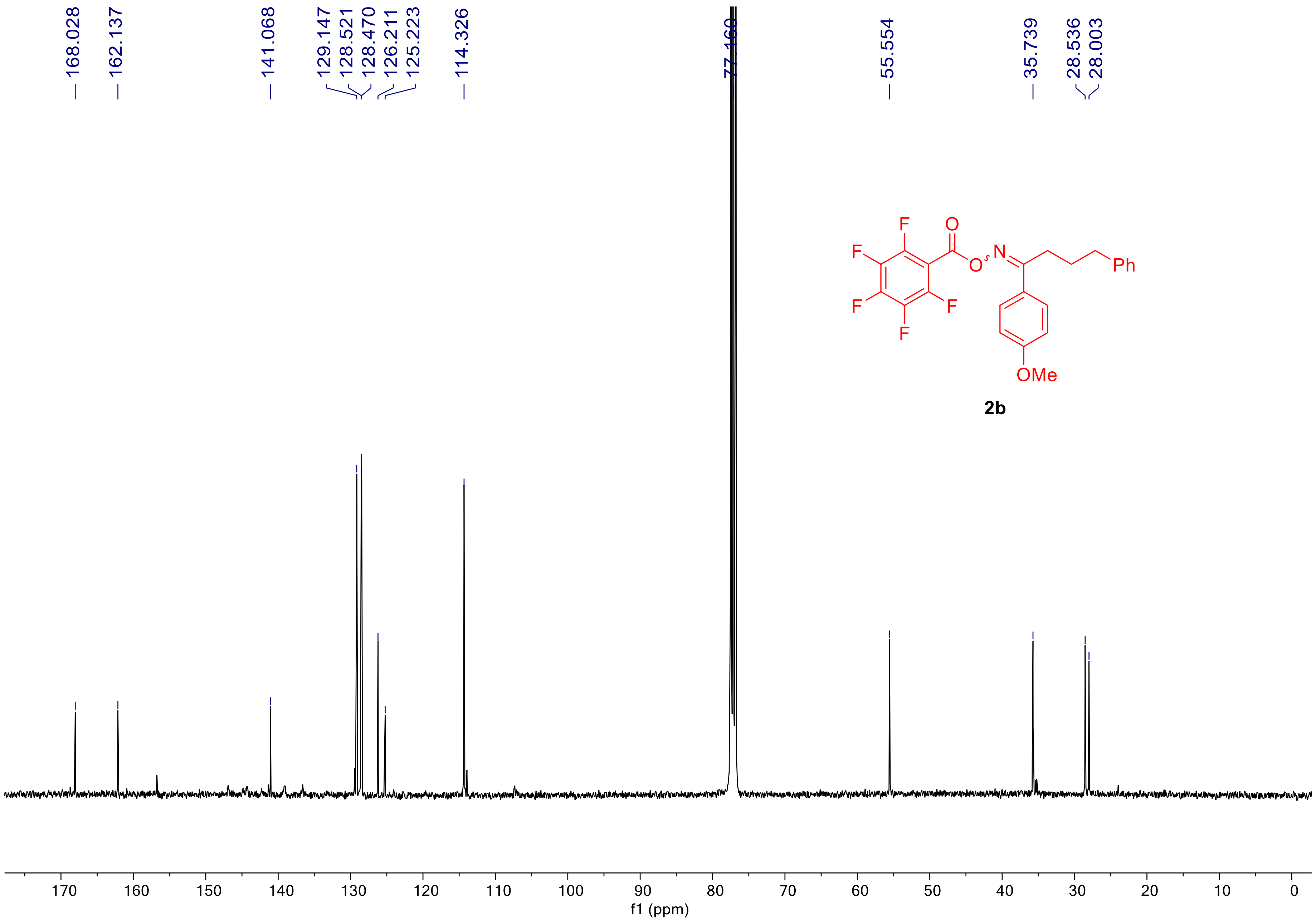

Supplementary Figure 26. ^{13}C NMR spectrum of 2b

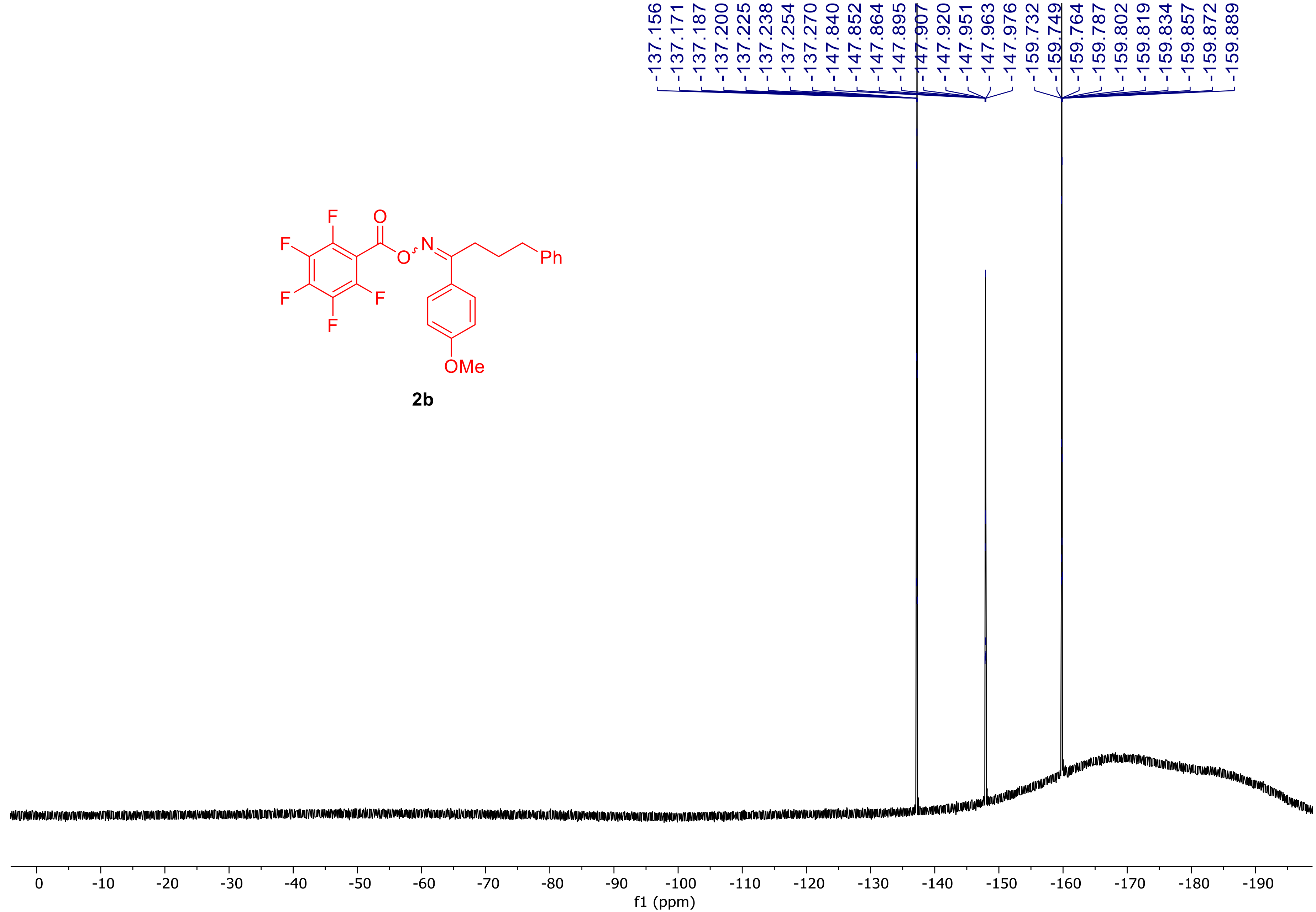

Supplementary Figure 27. ^{19}F NMR spectrum of 2b

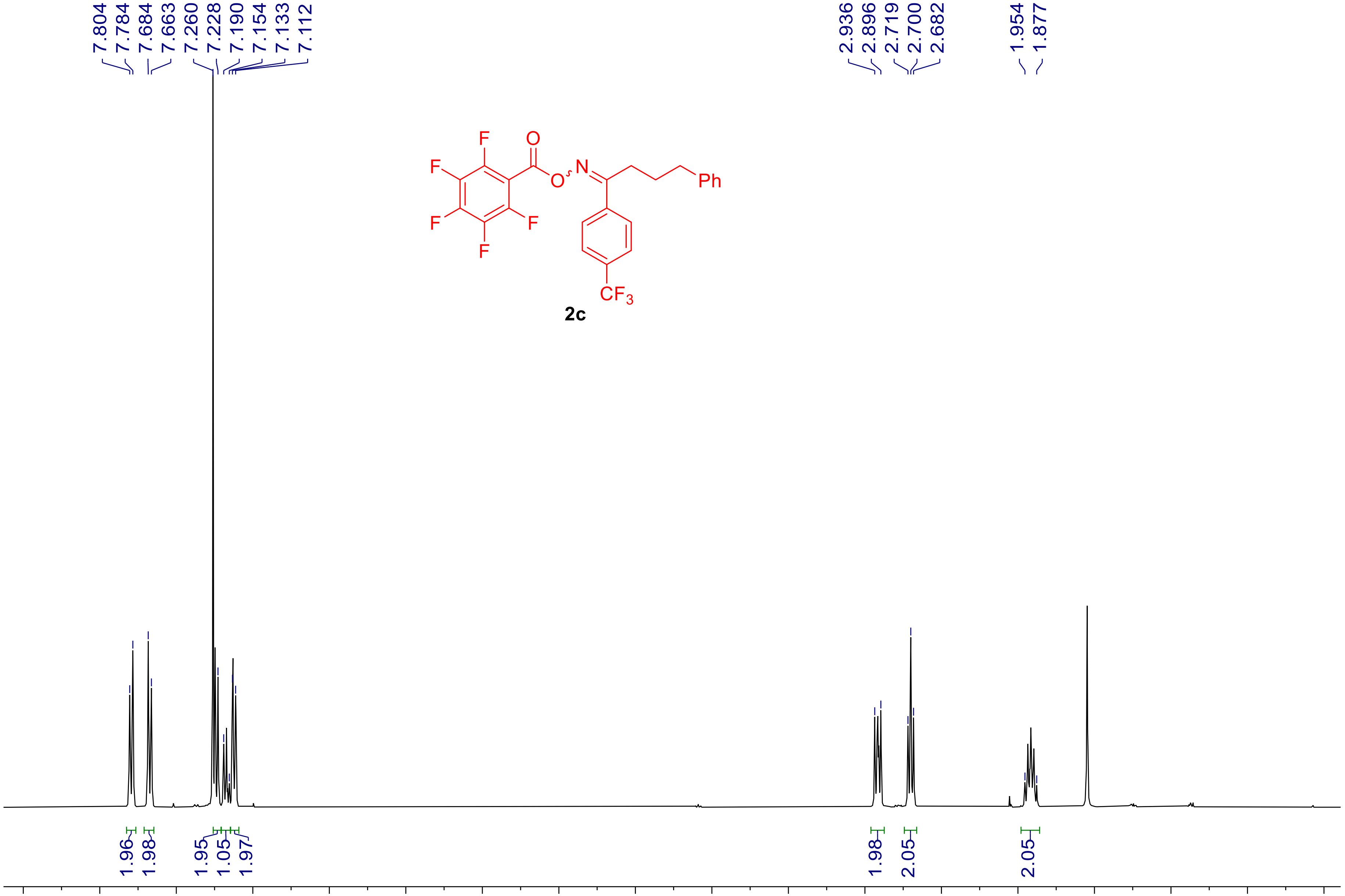

Supplementary Figure 28. ^{1}H NMR spectrum of 2c

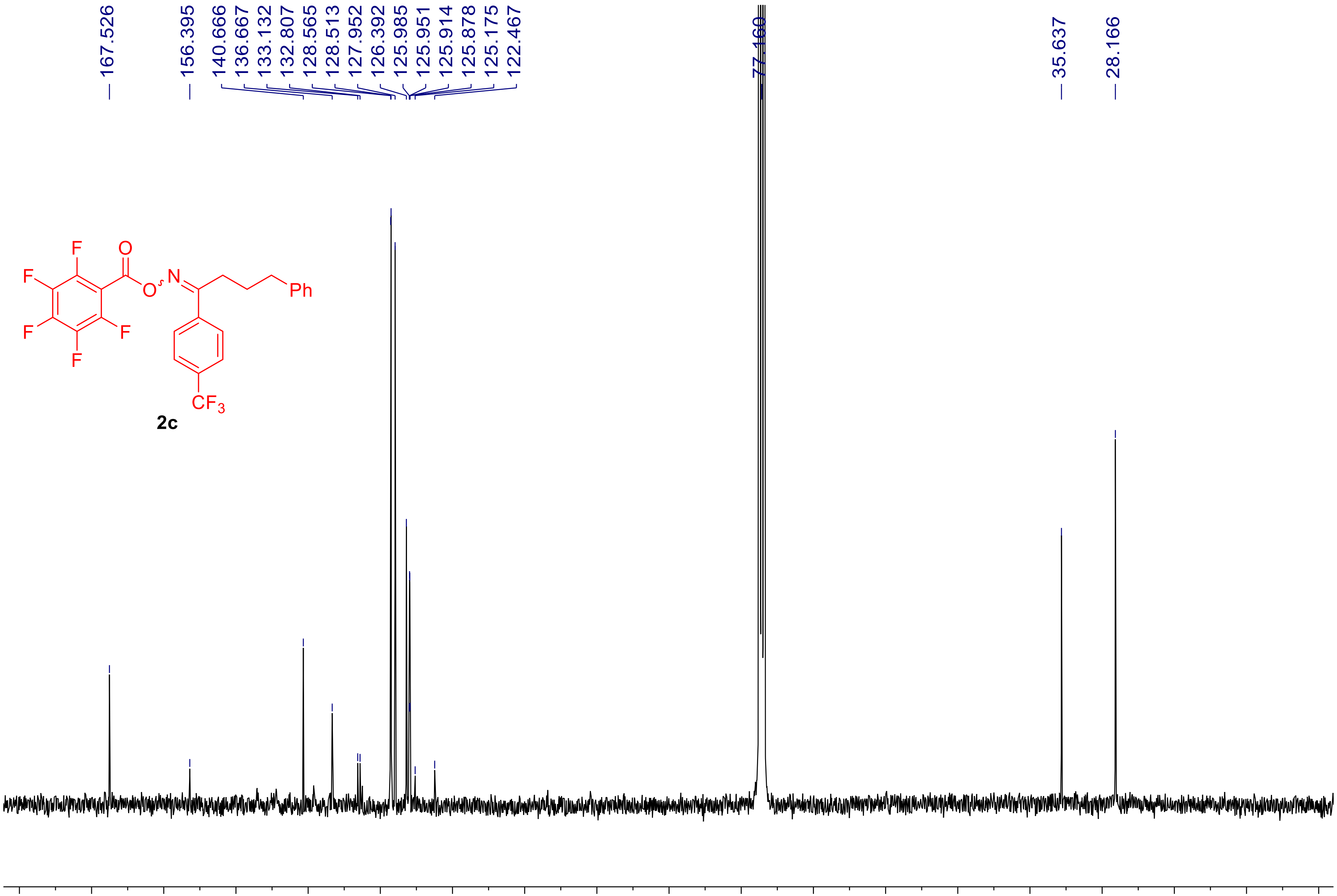

Supplementary Figure 29. ^{13}**C NMR spectrum of 2c**

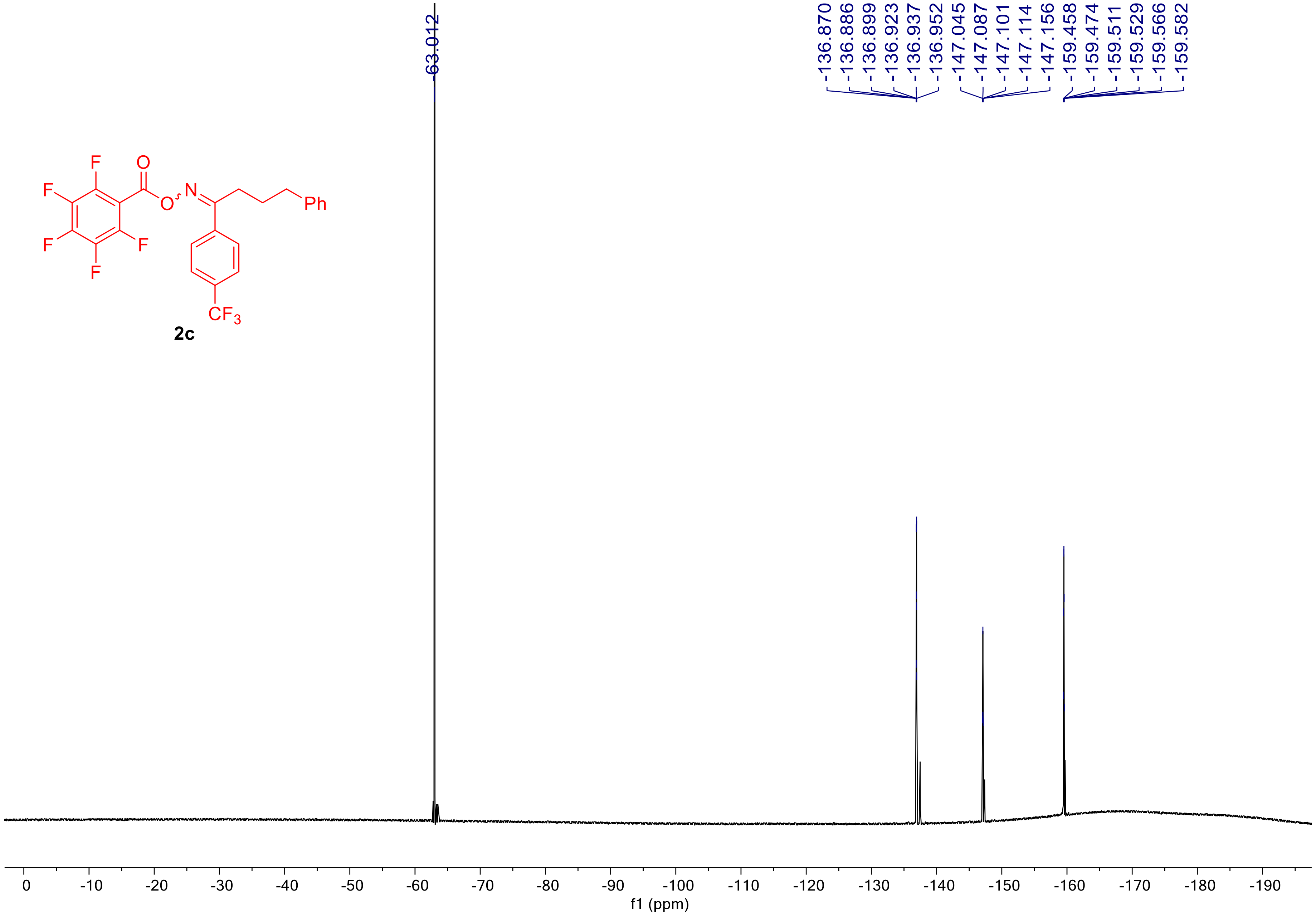

Supplementary Figure 30. ^{19}F NMR spectrum of 2c

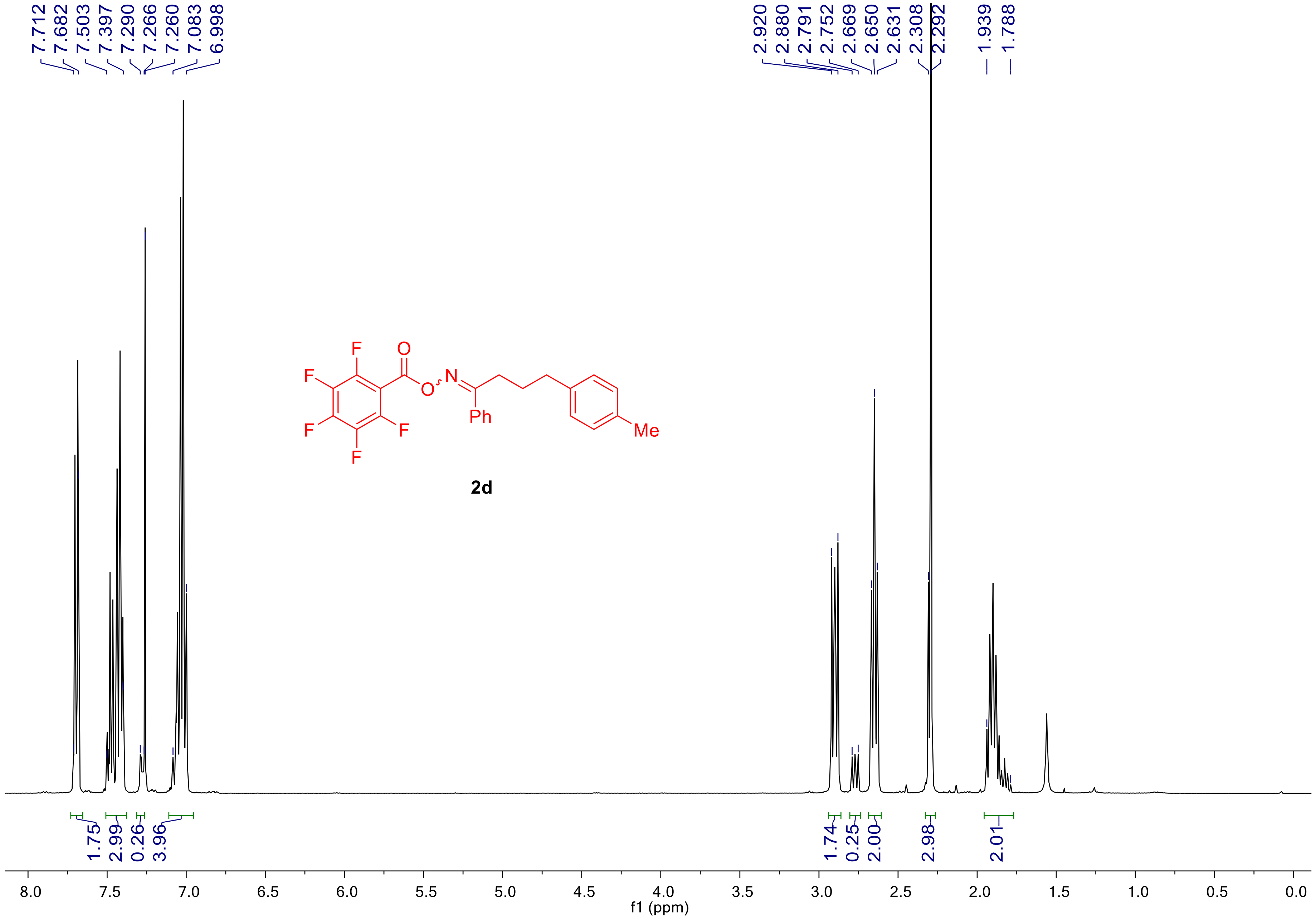

Supplementary Figure 31. ^{1}H NMR spectrum of 2d

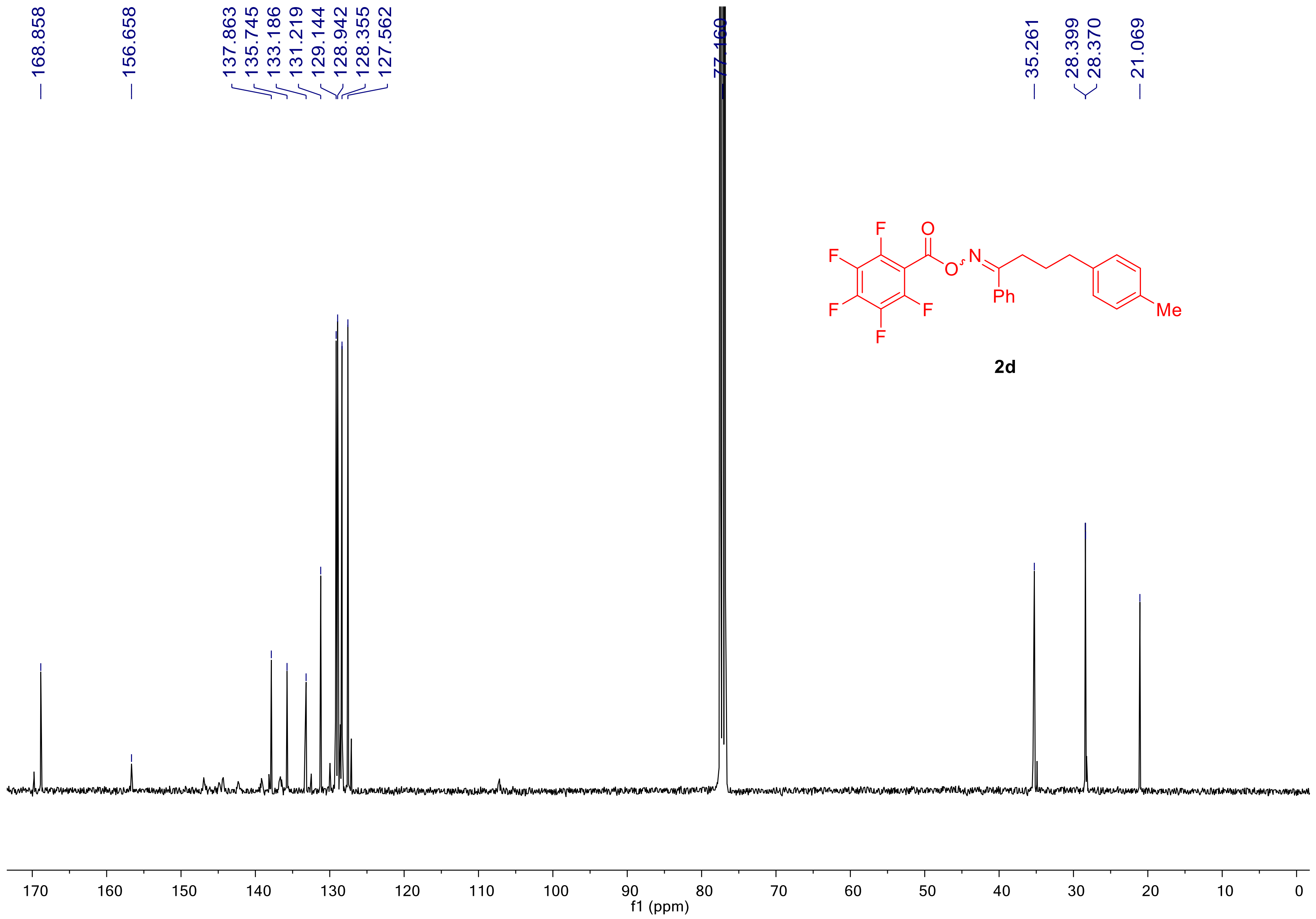

Supplementary Figure 32. ^{13}C NMR spectrum of 2d

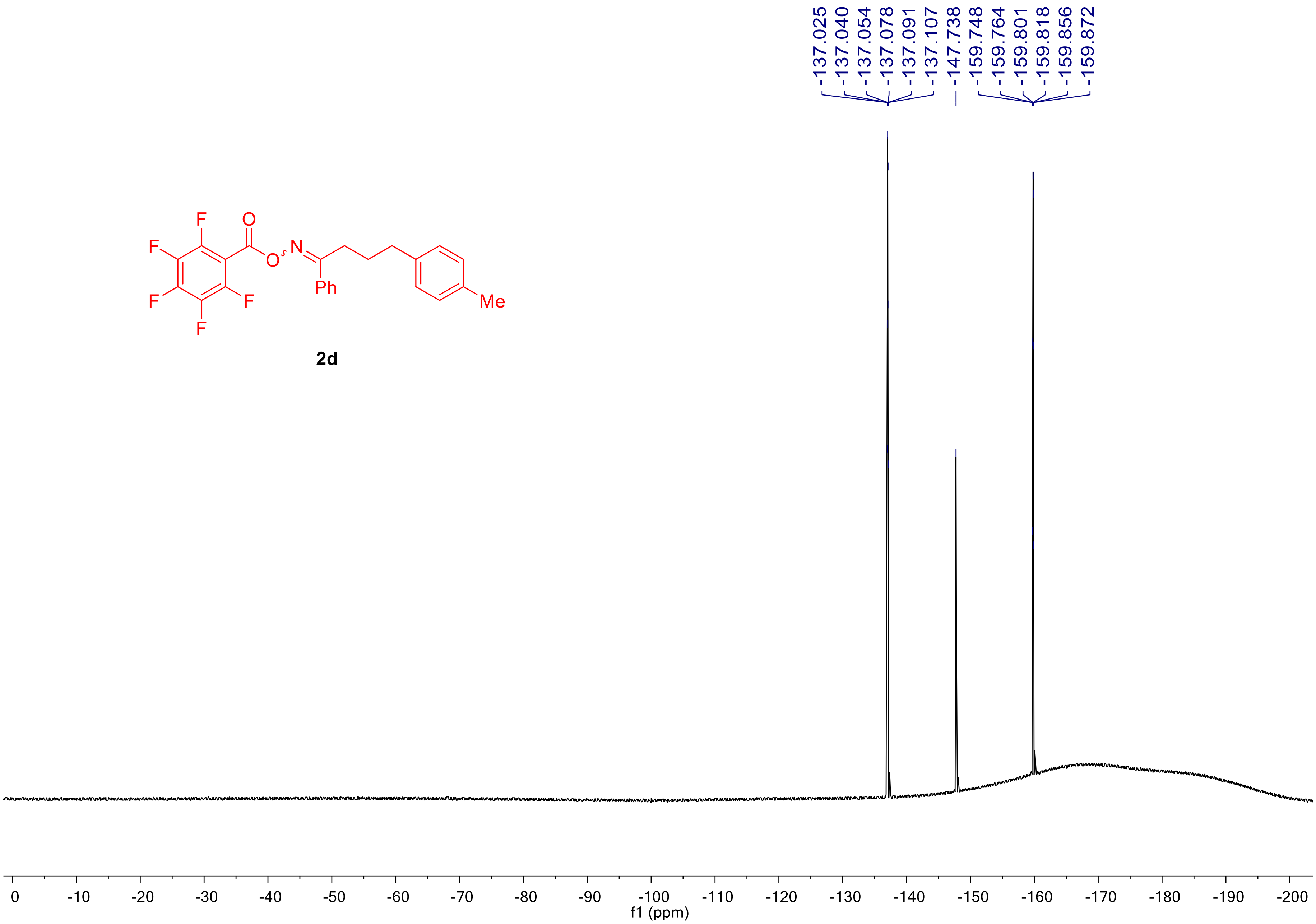

Supplementary Figure 33. ^{19}F NMR spectrum of 2d

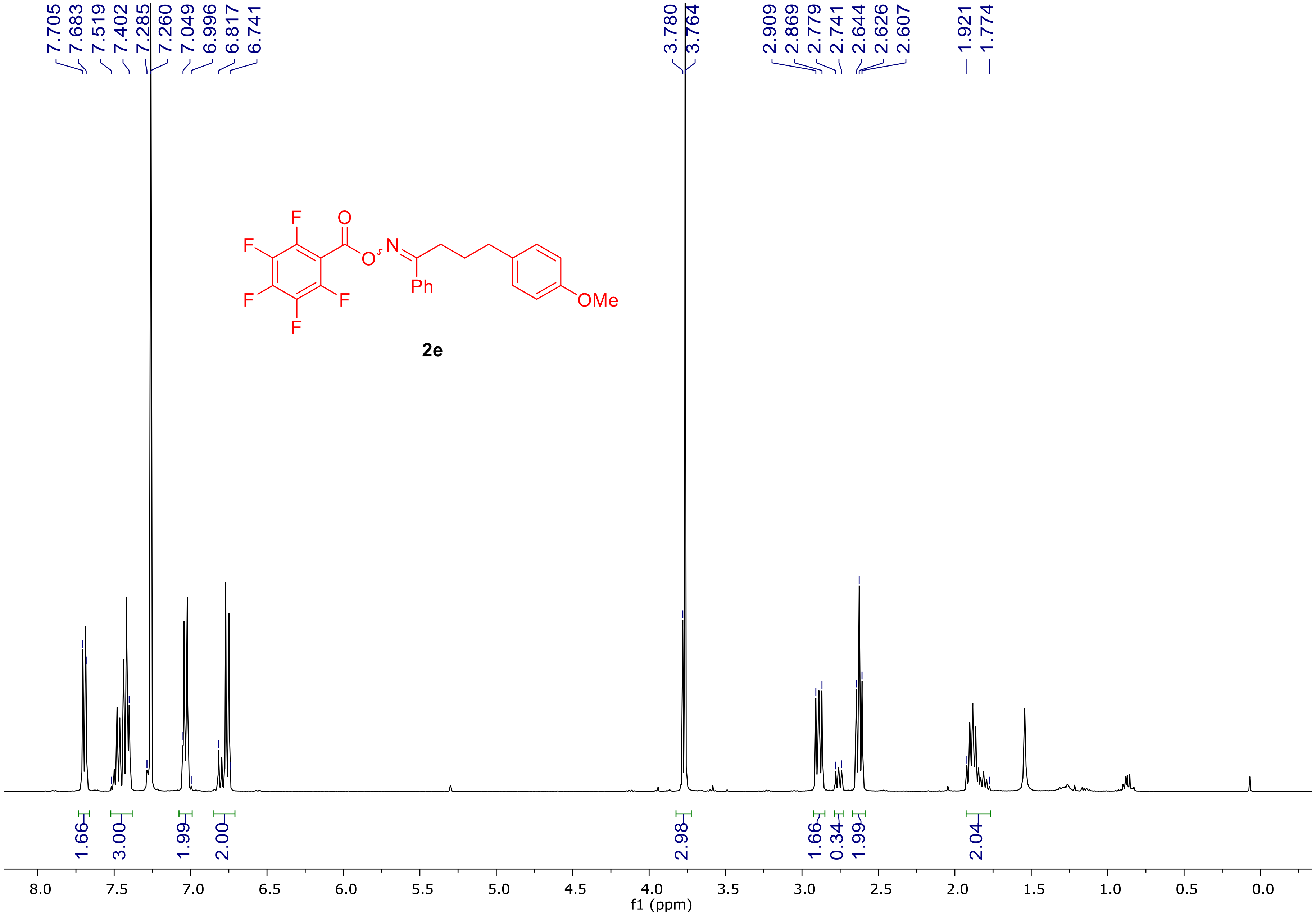

Supplementary Figure 34. ^{1}H NMR spectrum of 2e

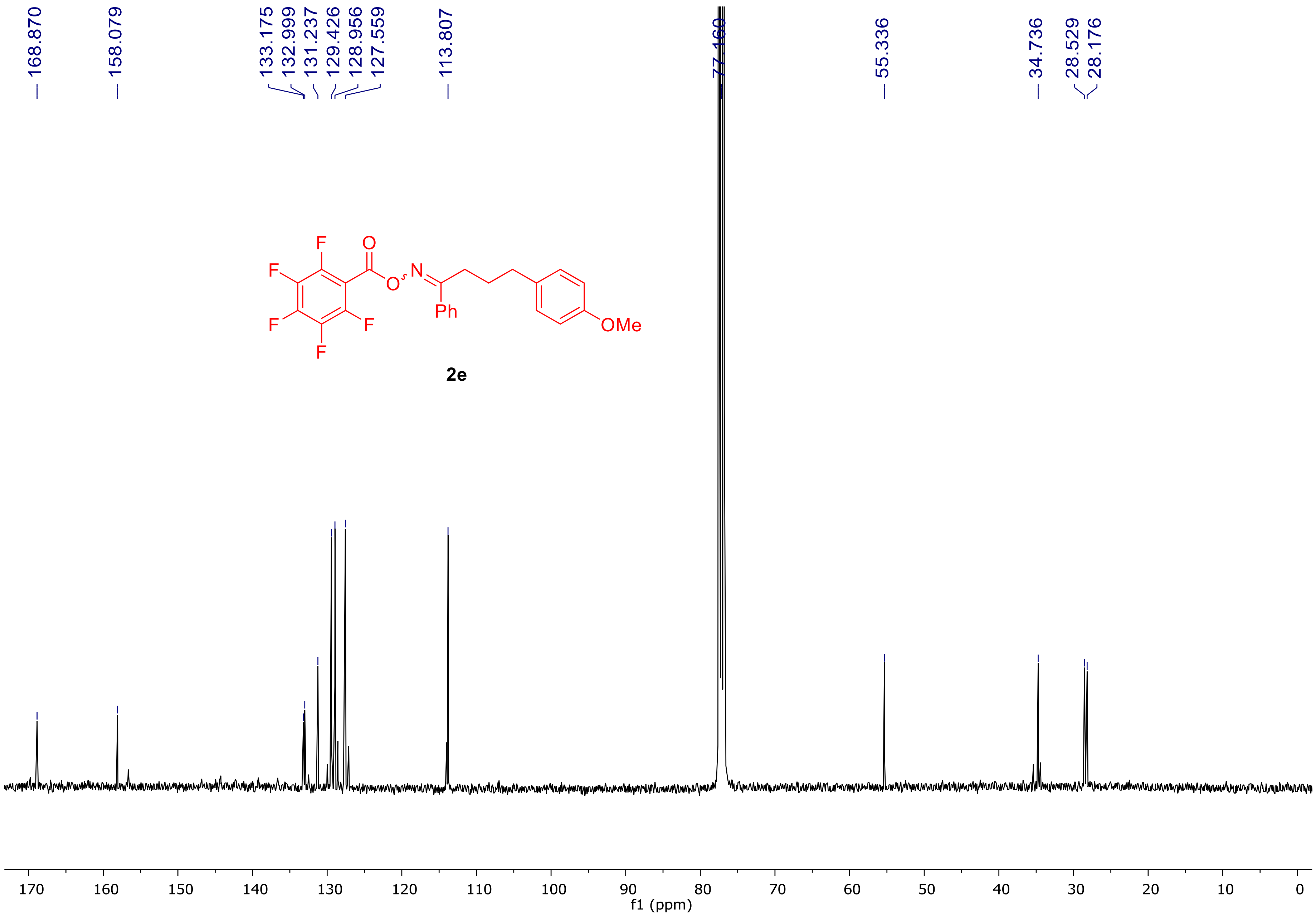

Supplementary Figure 35. ^{13}C NMR spectrum of 2e

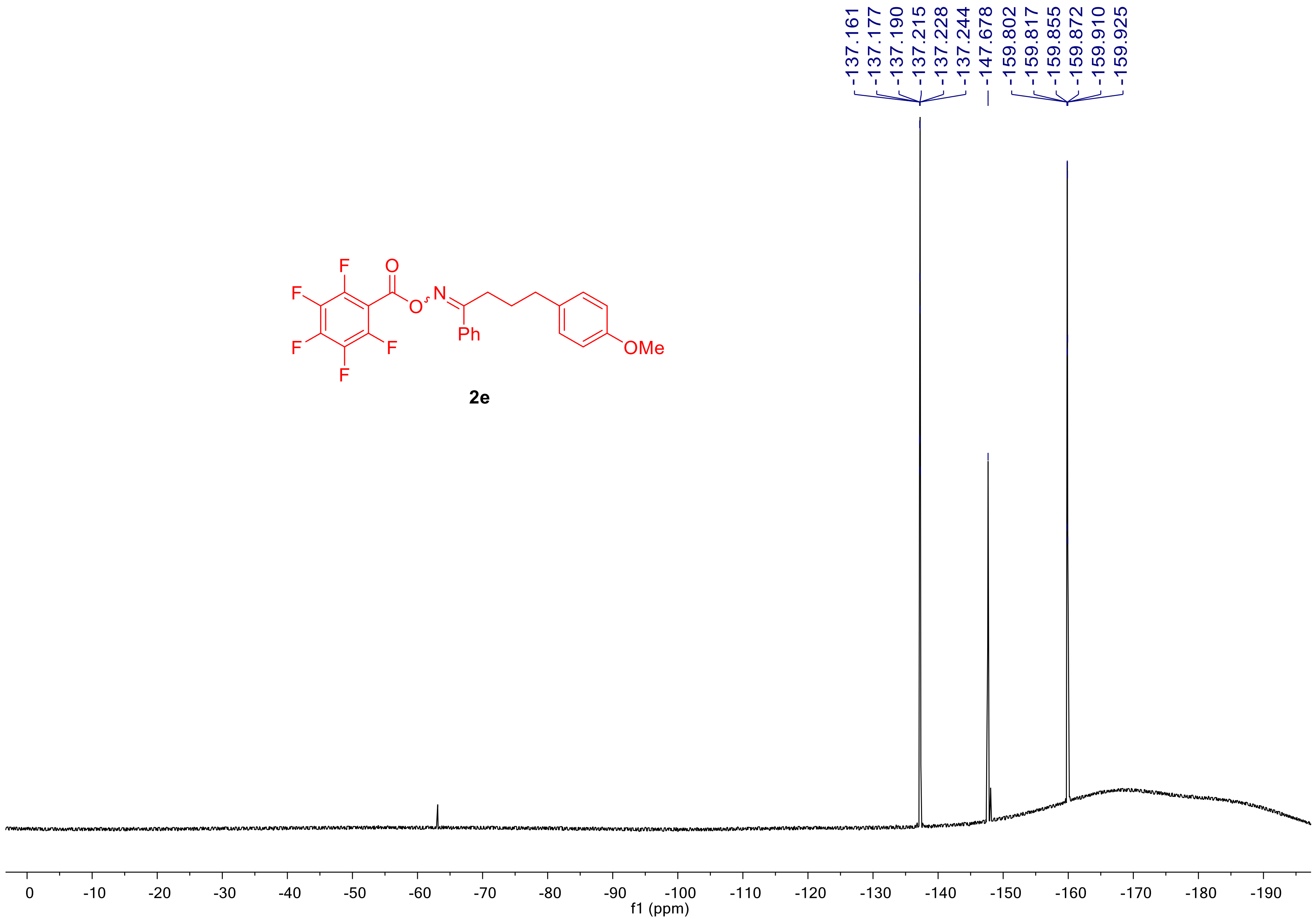

Supplementary Figure 36. ^{19}F NMR spectrum of 2e

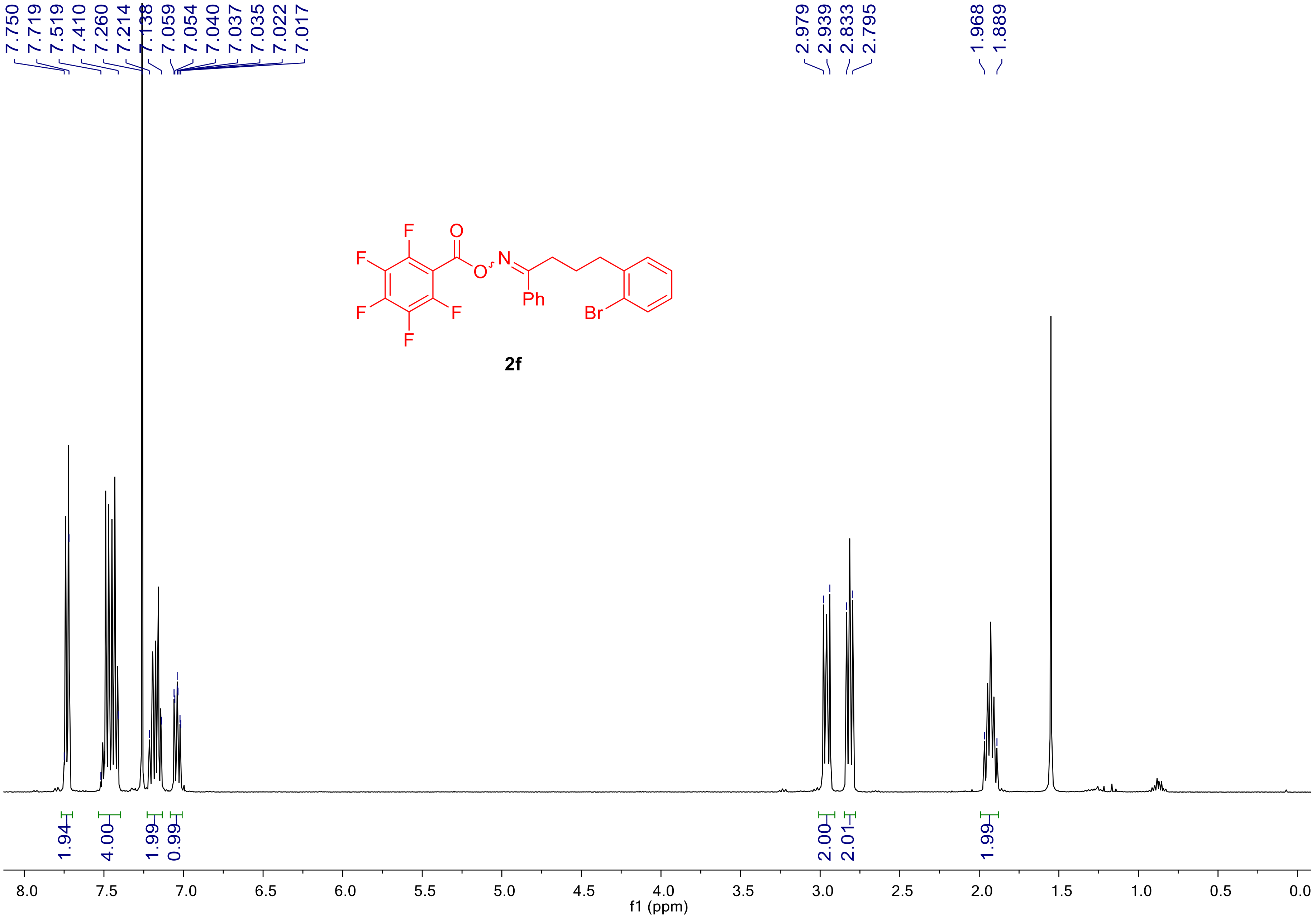

Supplementary Figure 37. ^{1}H NMR spectrum of 2f

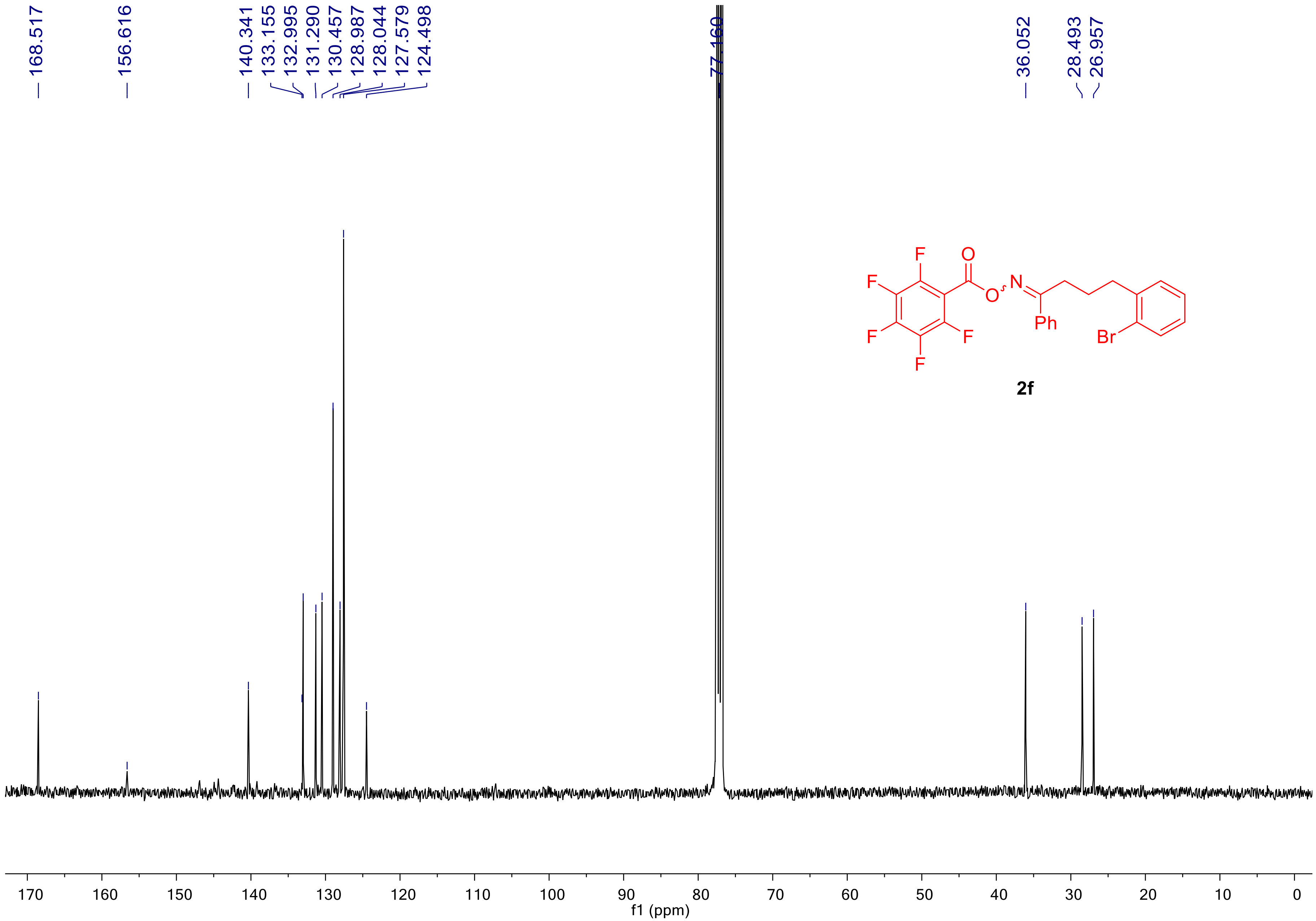

Supplementary Figure 38. ^{13}C NMR spectrum of 2f

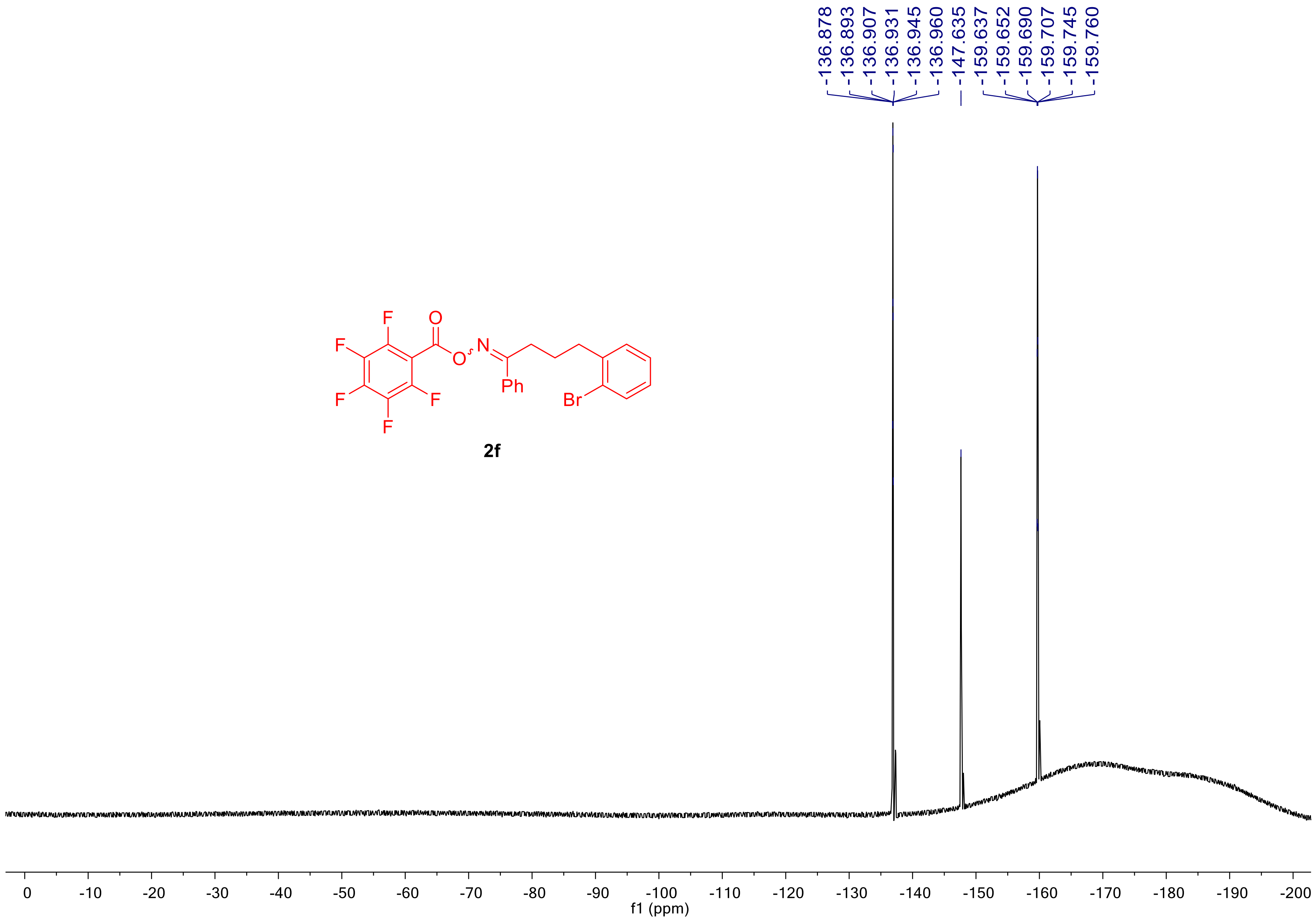

Supplementary Figure 39. ^{19}F NMR spectrum of 2f

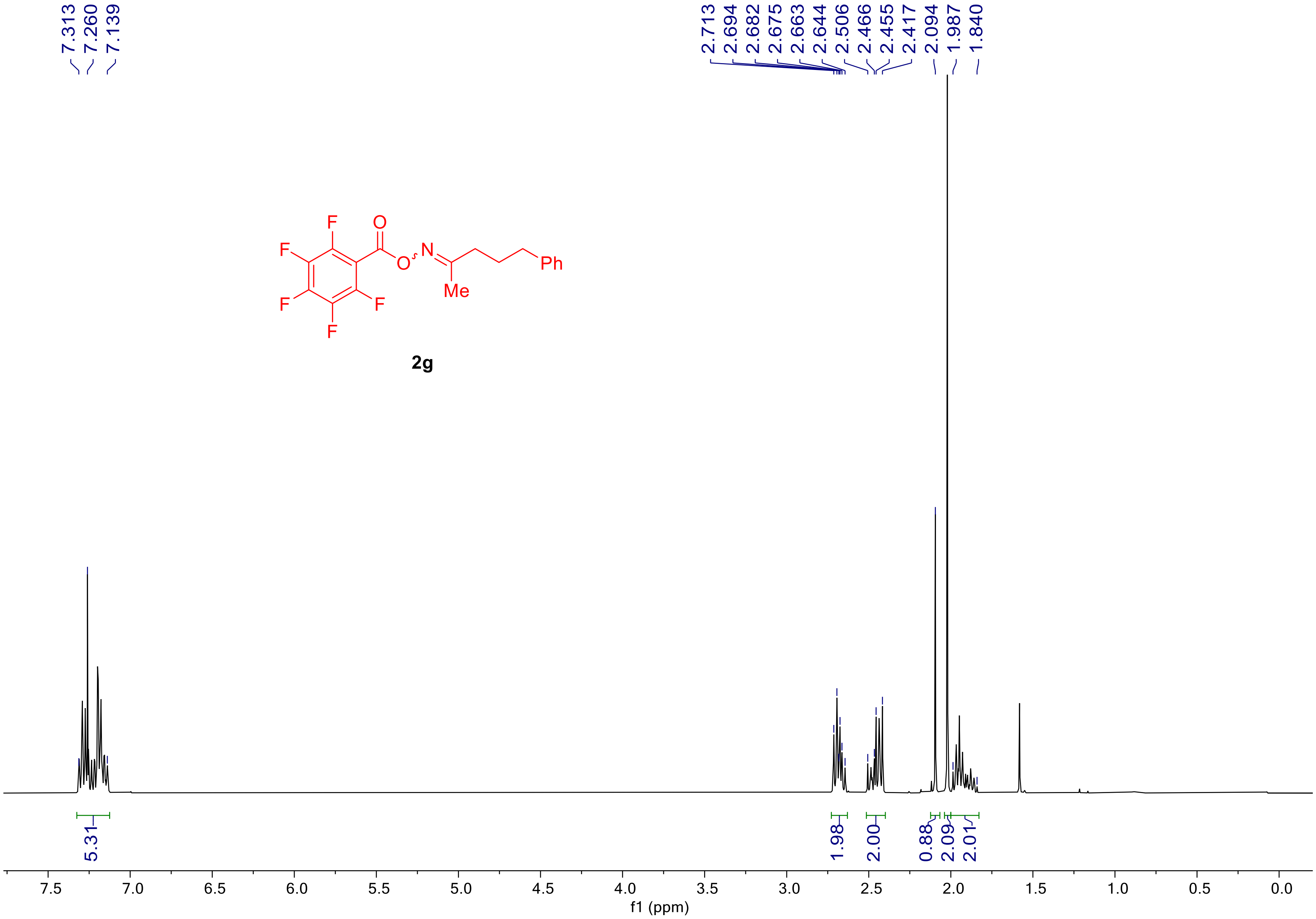

Supplementary Figure 40. ^{1}H NMR spectrum of 2g

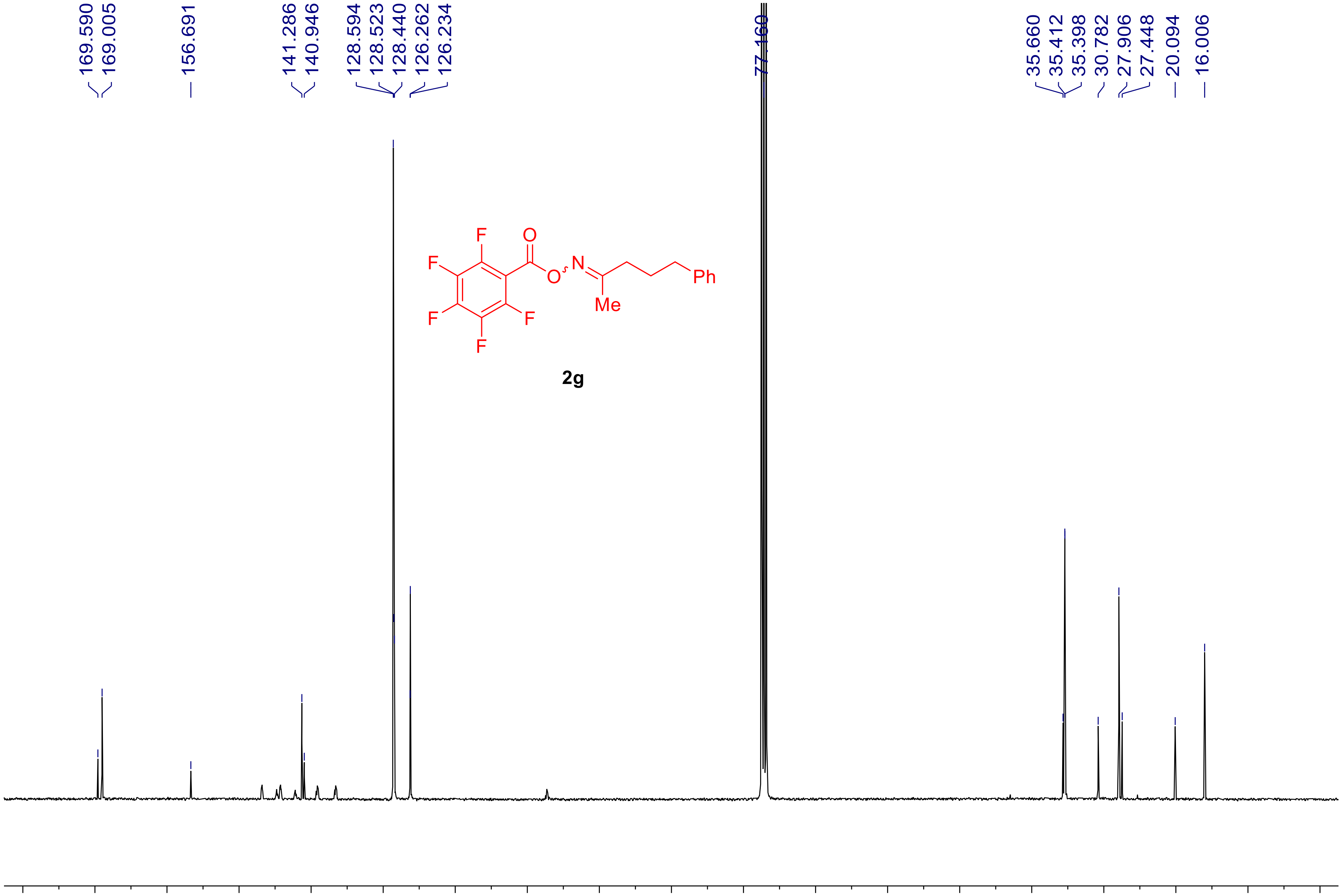

Supplementary Figure 41. ^{13}C NMR spectrum of 2g

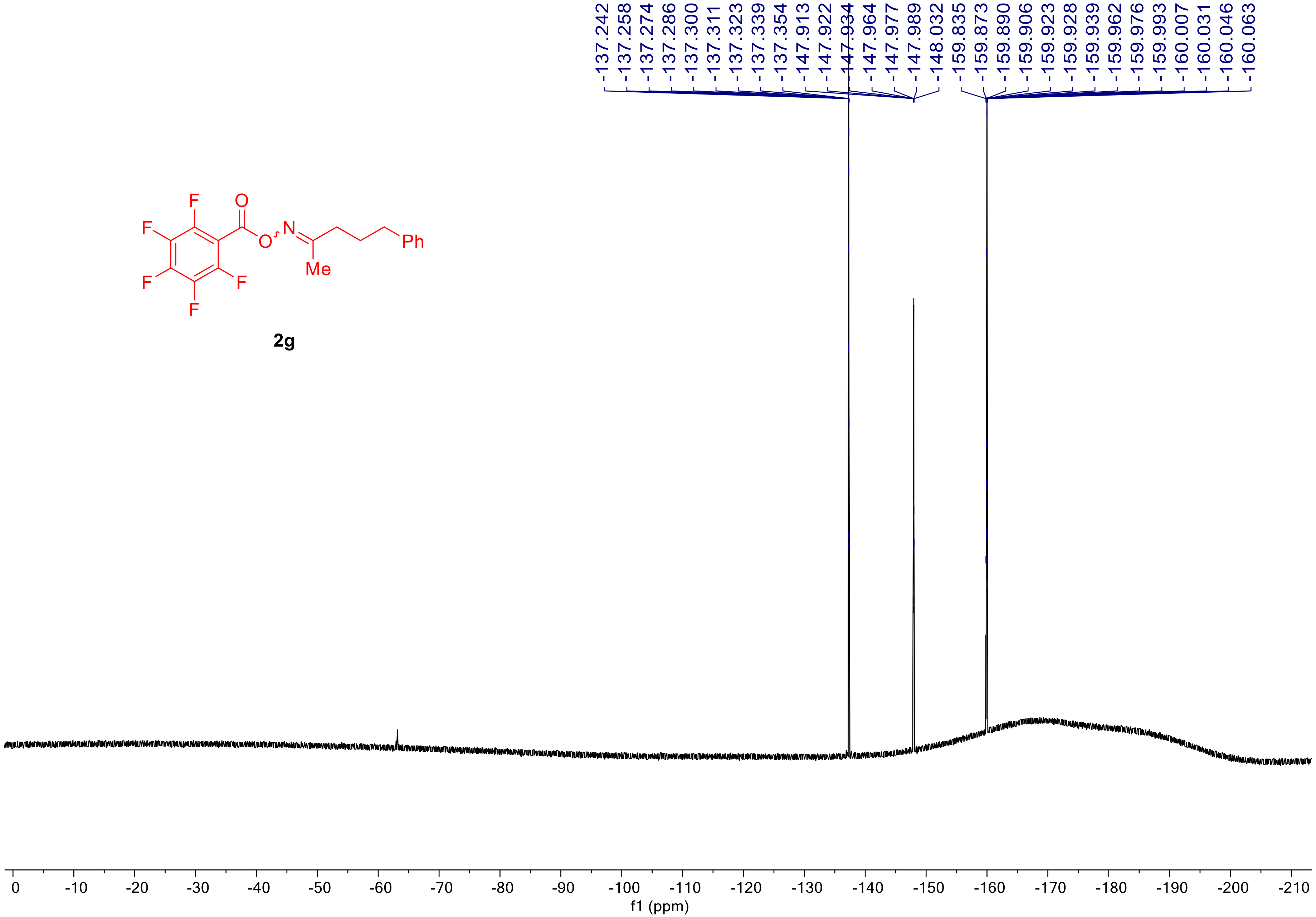

Supplementary Figure 42. ^{19}F NMR spectrum of 2g

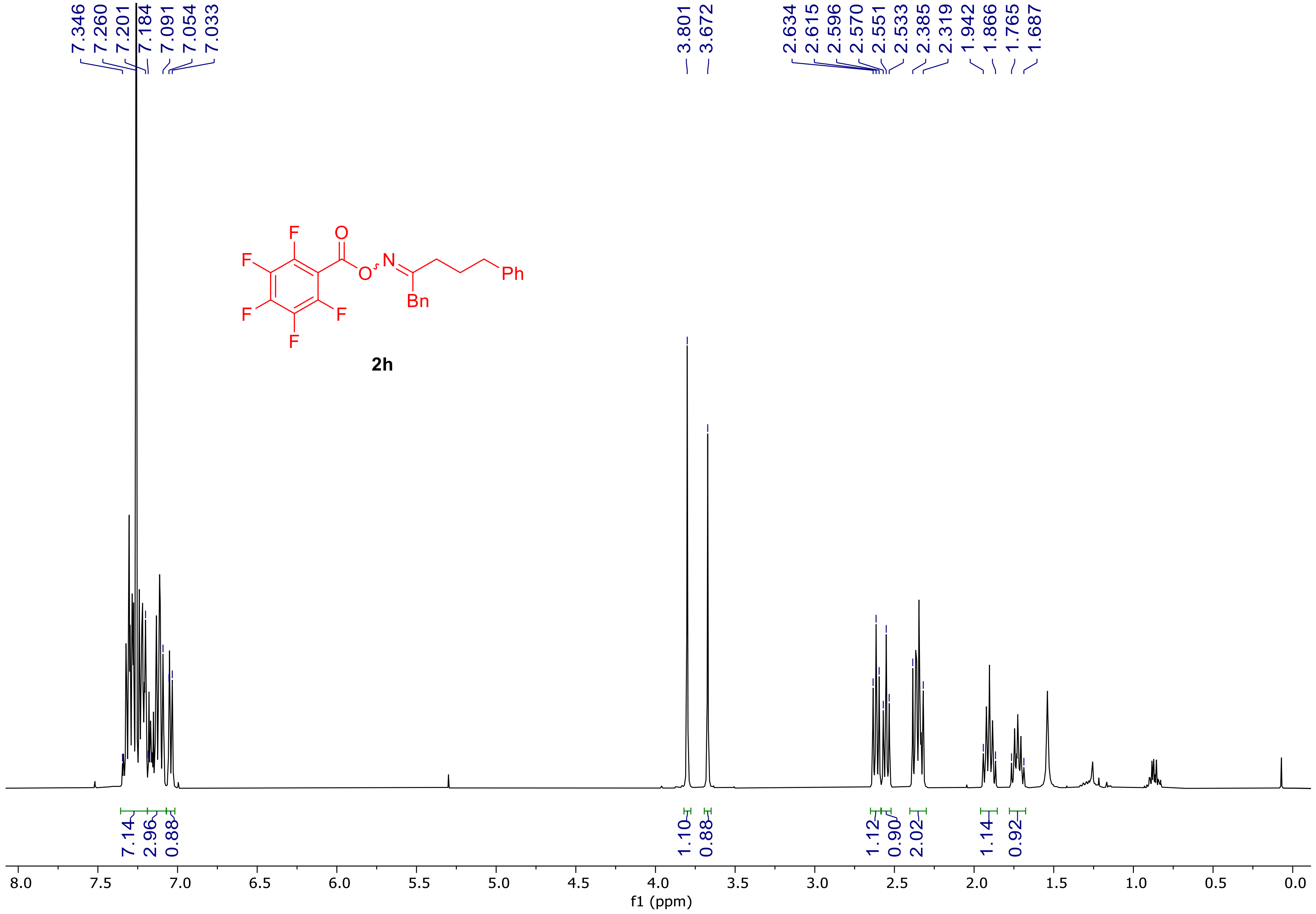

Supplementary Figure 43. ^{1}**H NMR spectrum of 2h**

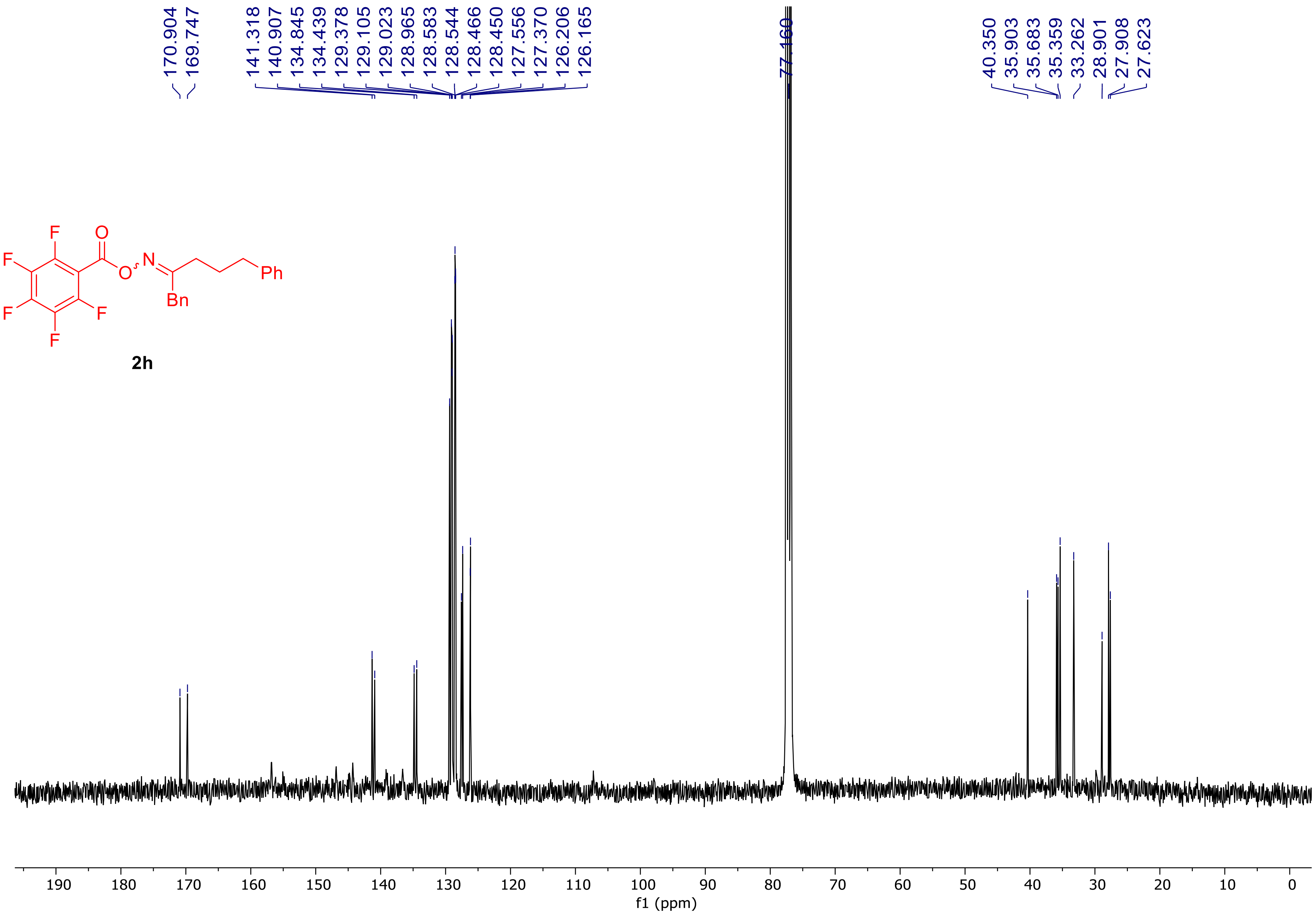

Supplementary Figure 44. ^{13}C NMR spectrum of 2h

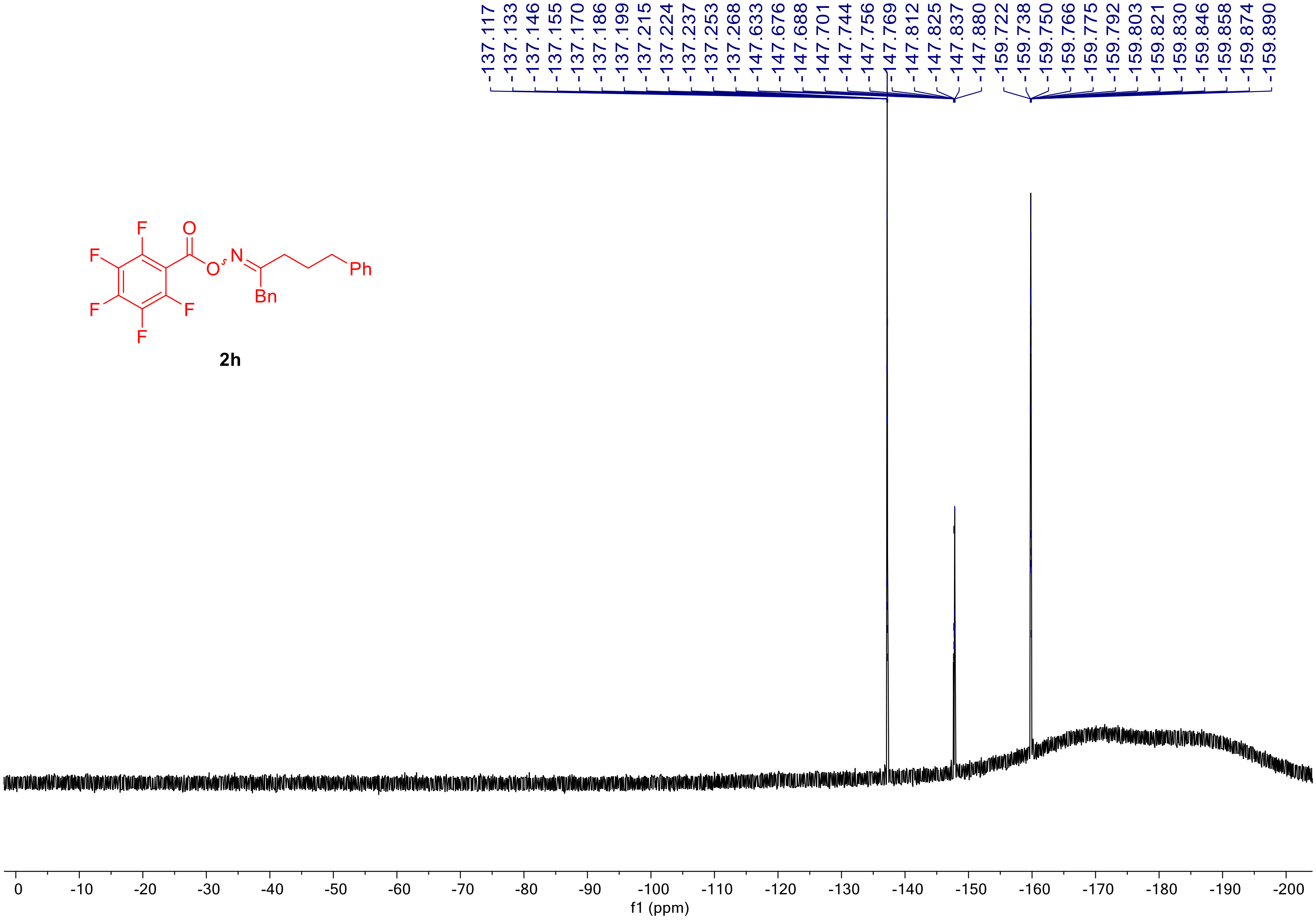

Supplementary Figure 45. ^{19}F NMR spectrum of 2h

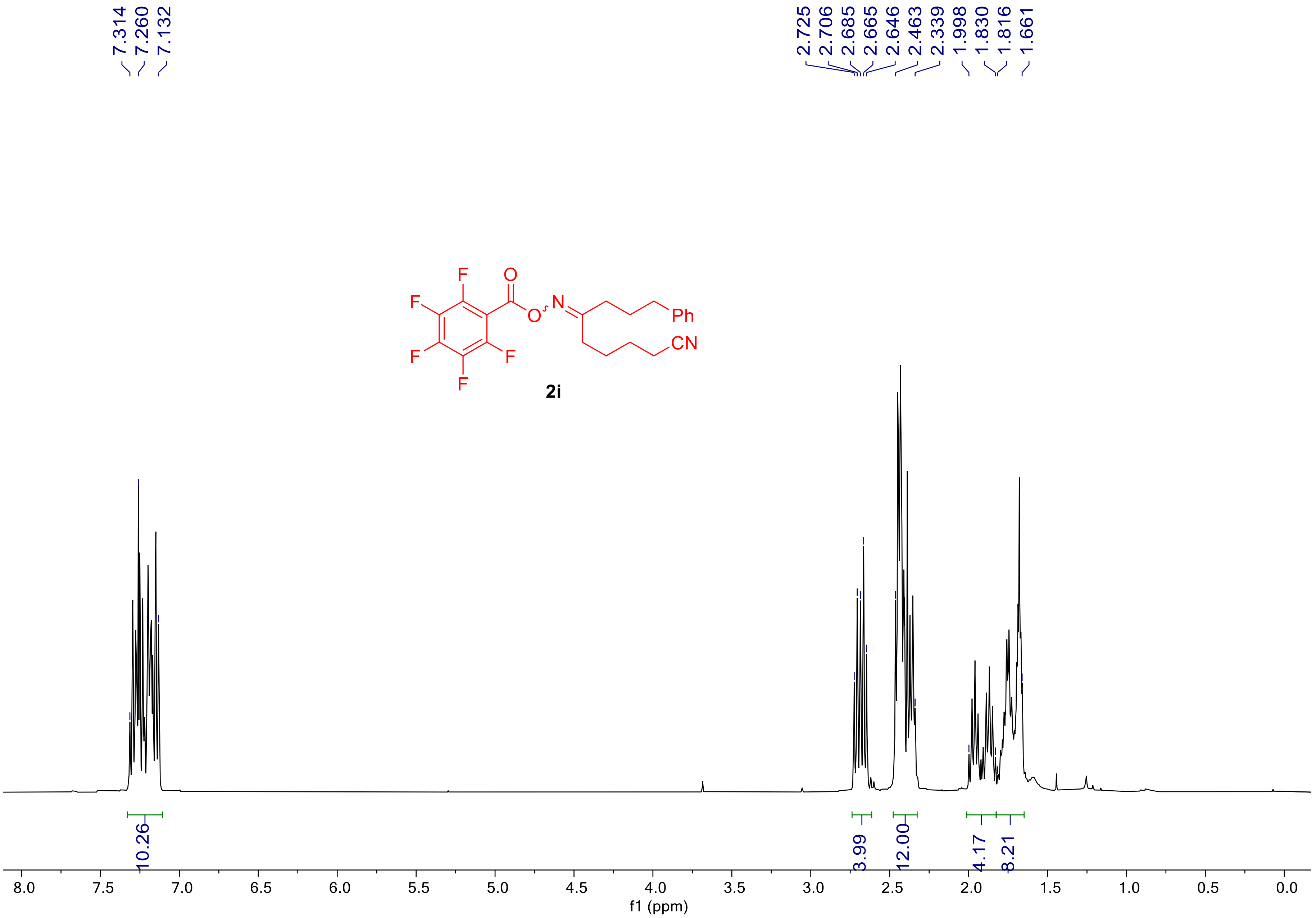

Supplementary Figure 46. ^{1}H NMR spectrum of 2i

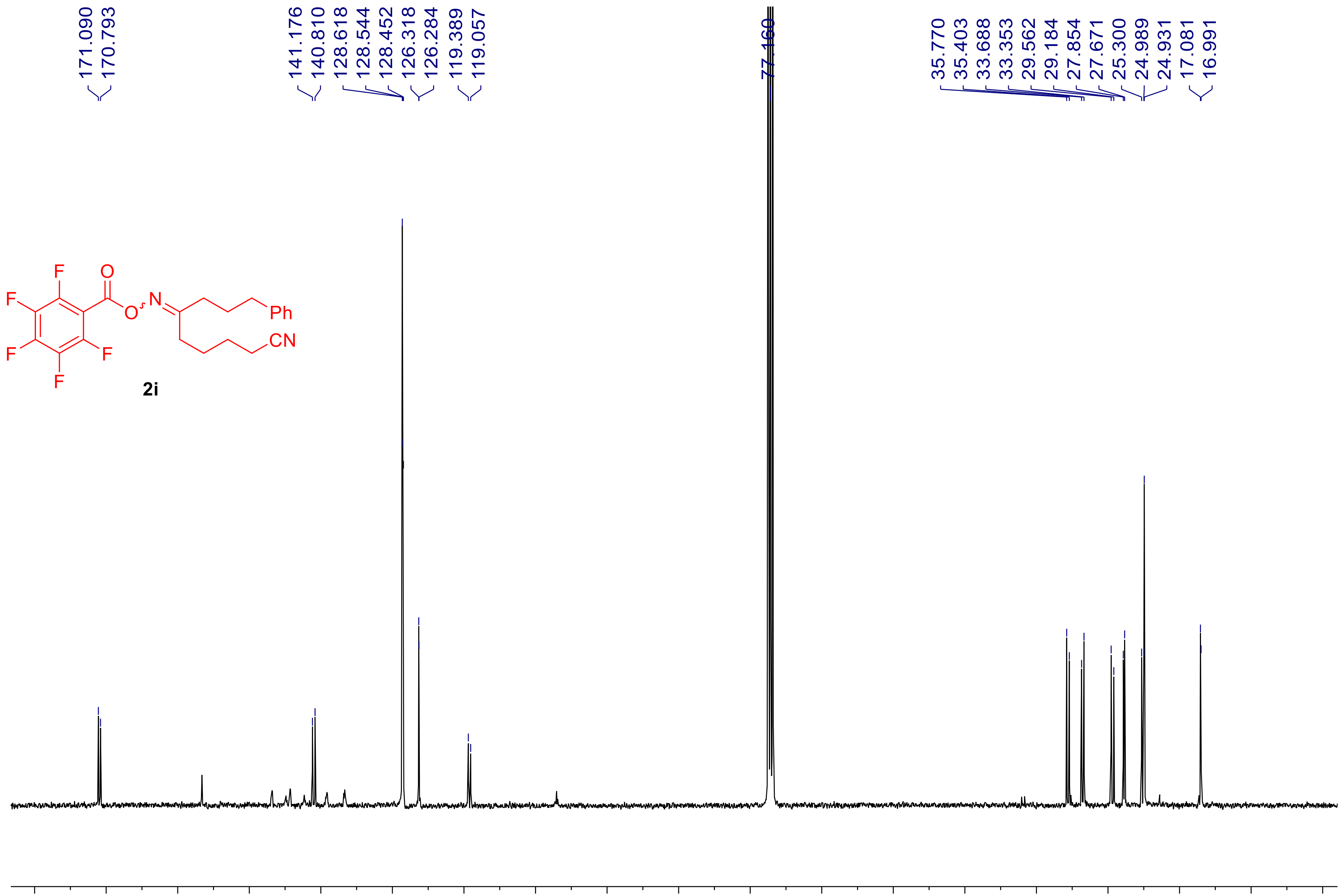

Supplementary Figure 47. ^{13}C NMR spectrum of 2i

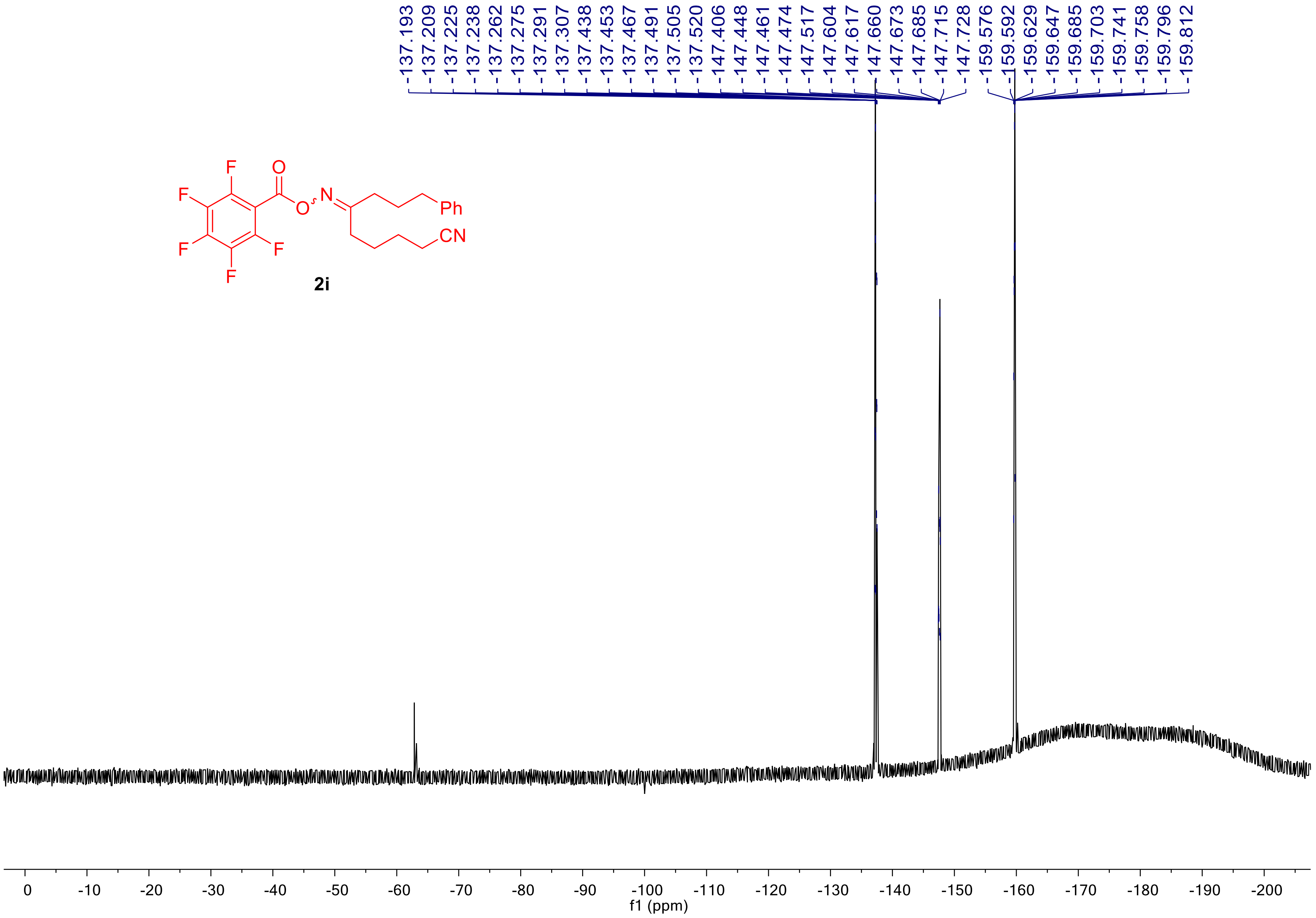

Supplementary Figure 48. ^{19}F NMR spectrum of 2i

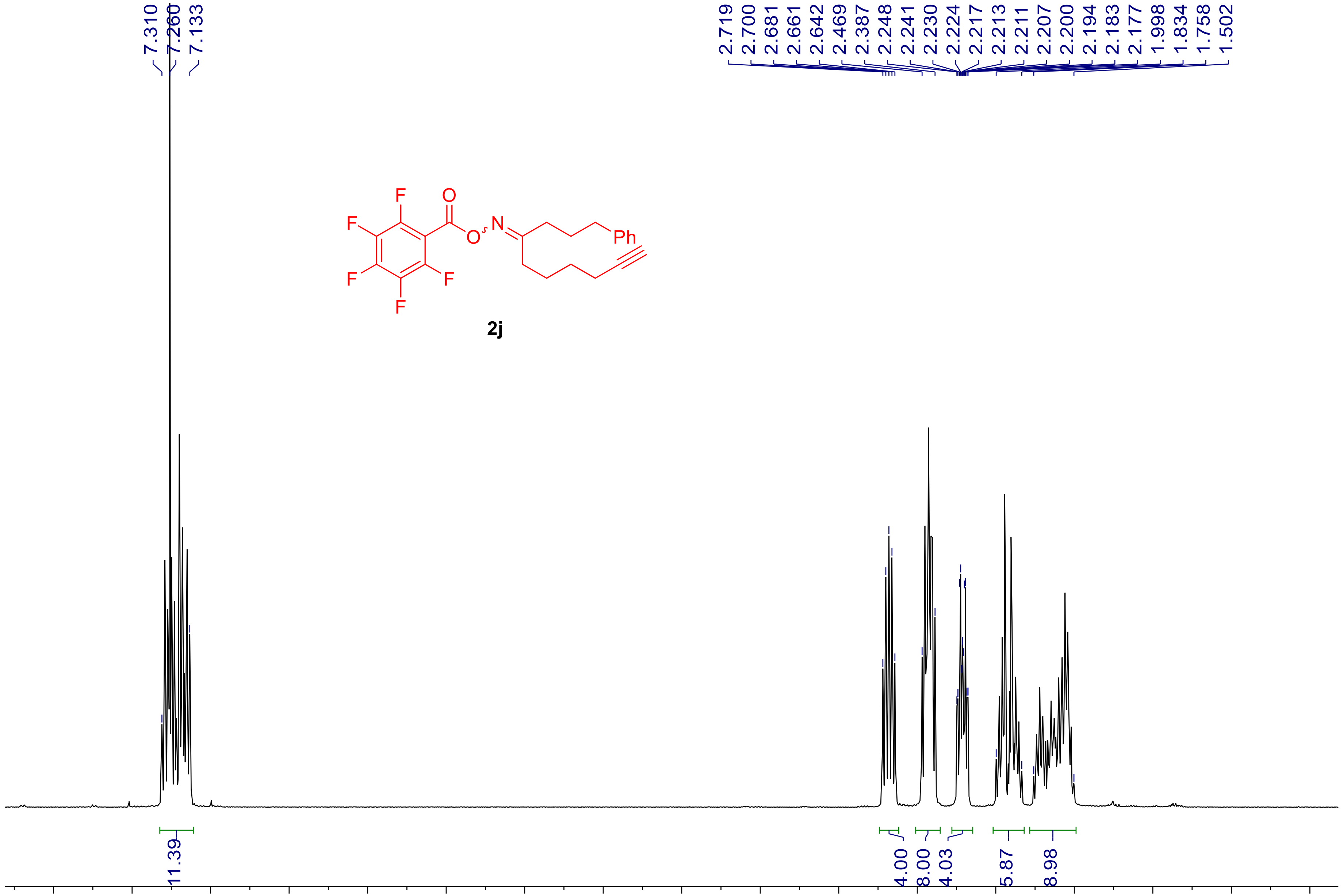

Supplementary Figure 49. ^{1}H NMR spectrum of 2j

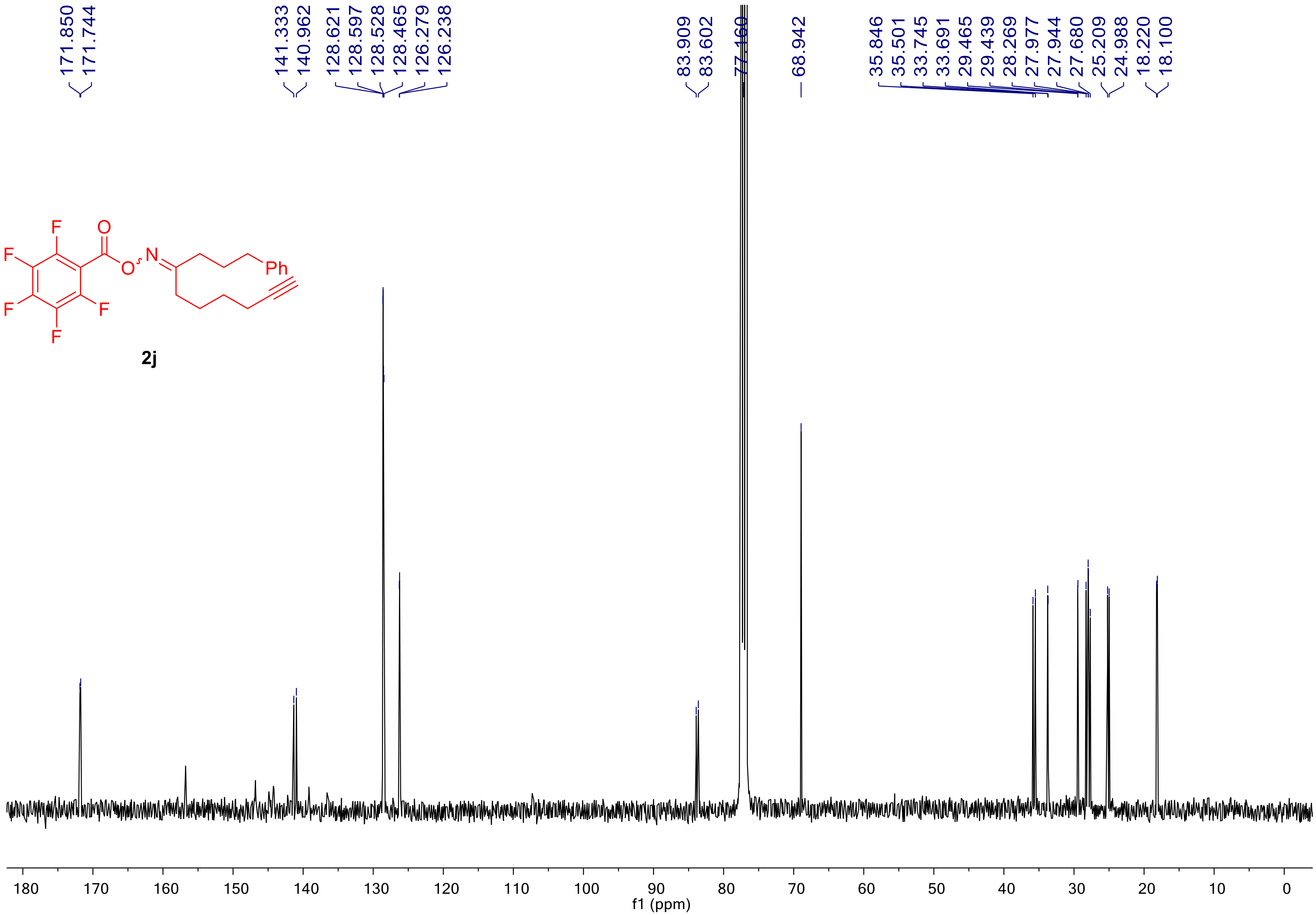

Supplementary Figure 50. ^{13}**C NMR spectrum of 2j**

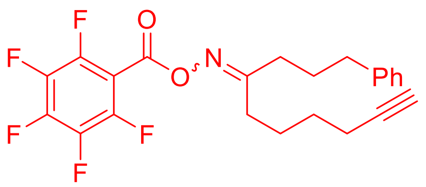

2j

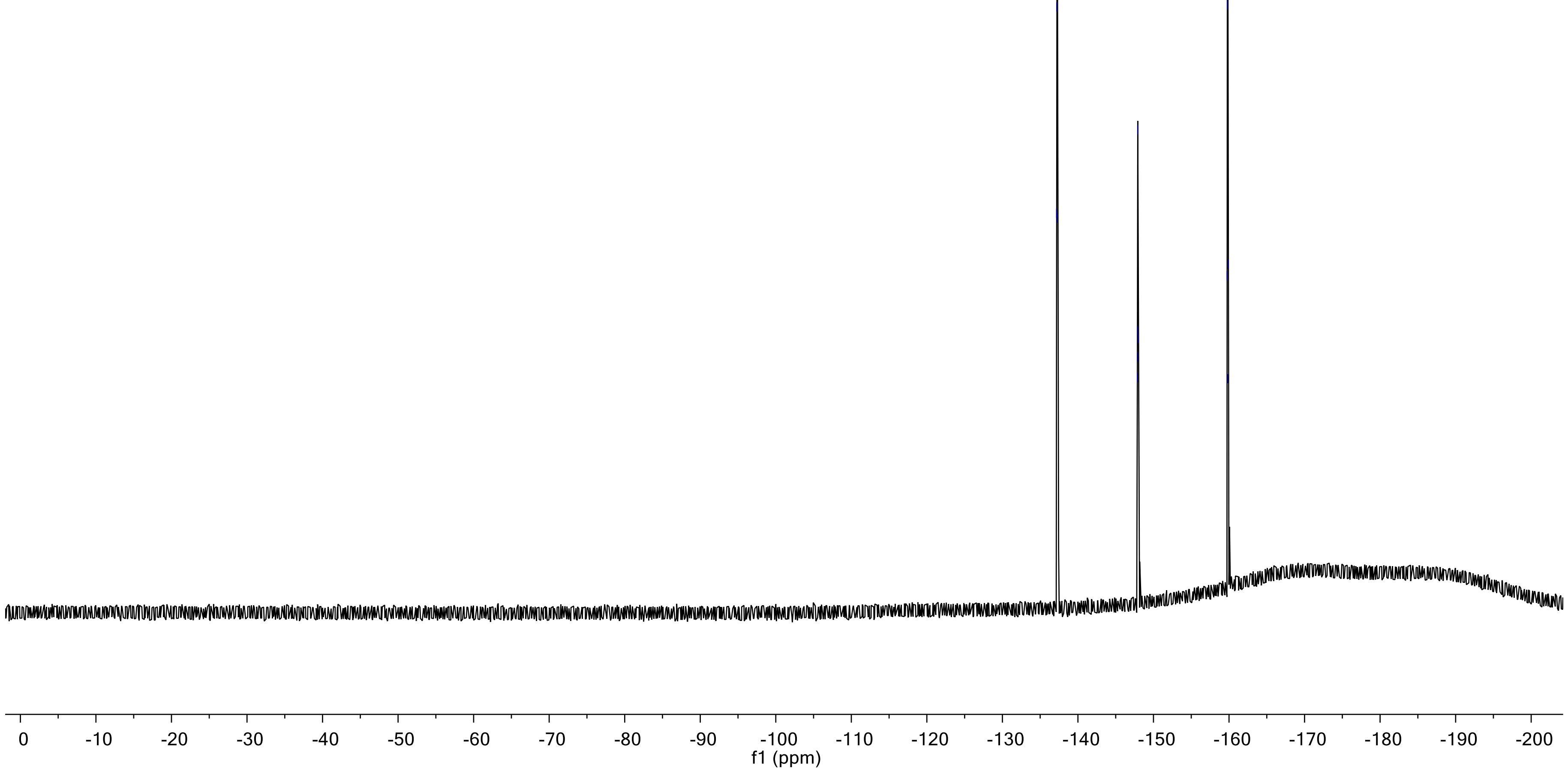

Supplementary Figure 51. ^{19}F NMR spectrum of 2j

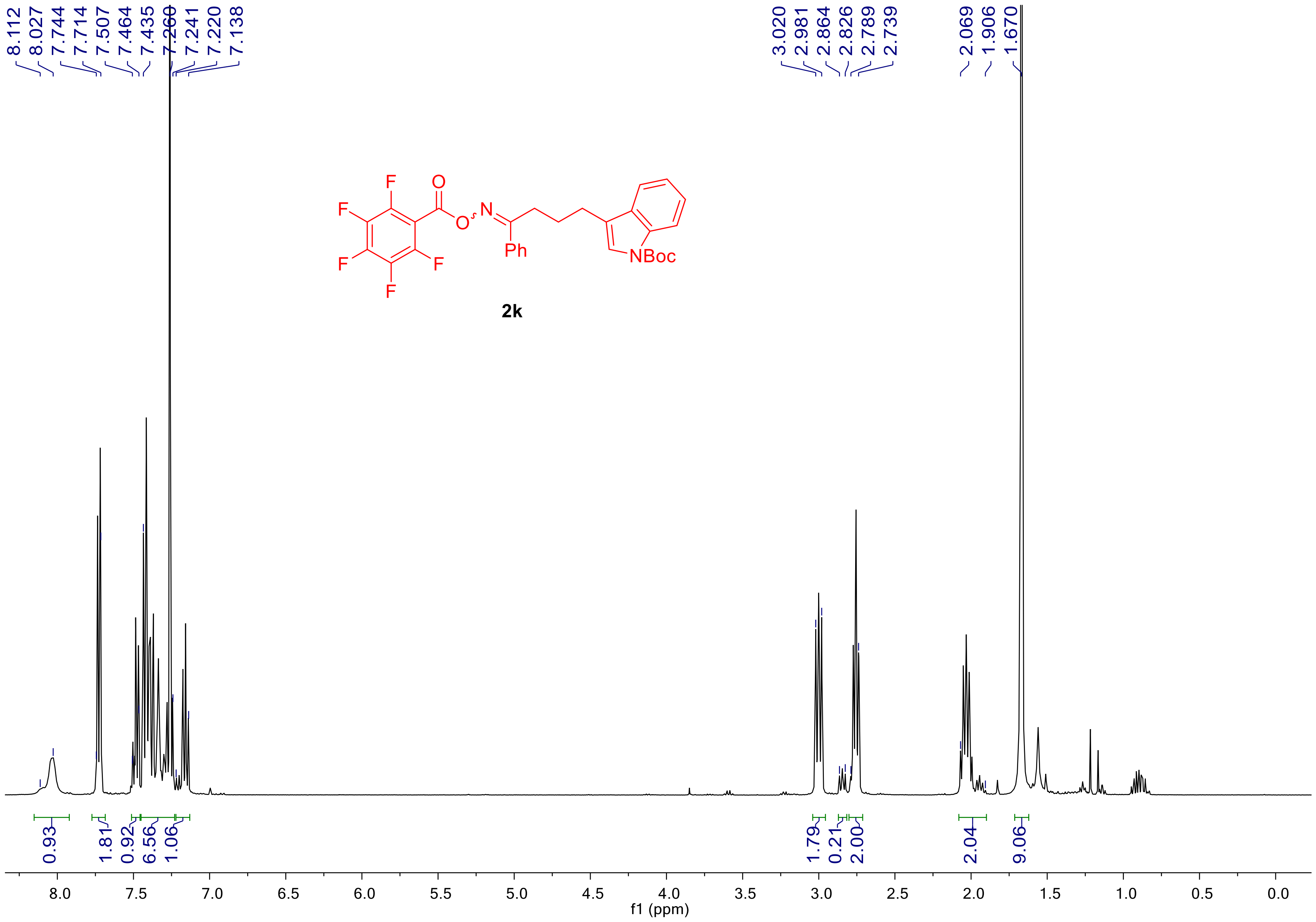

Supplementary Figure 52. ^{1}H NMR spectrum of 2k

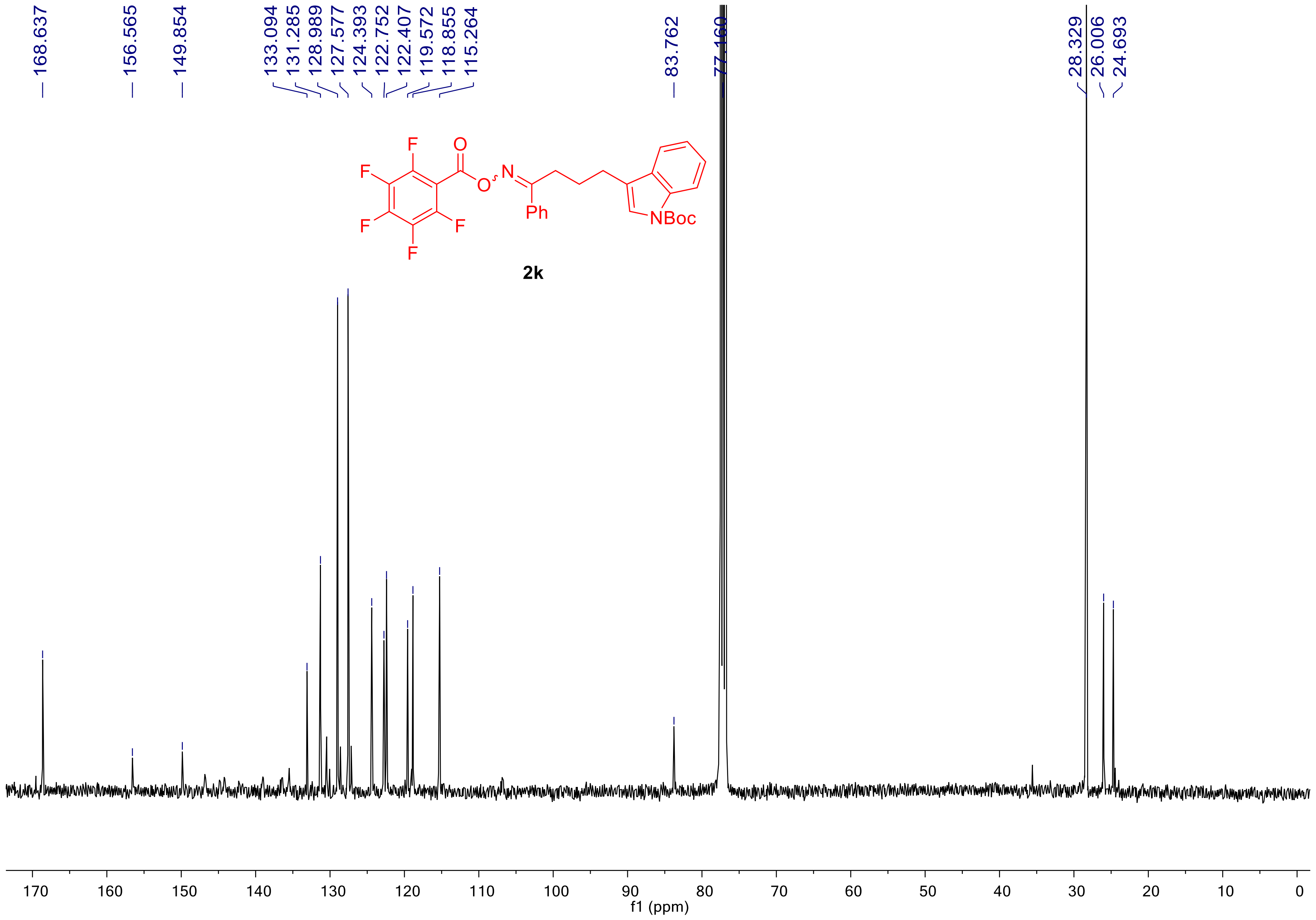

Supplementary Figure 53. ^{13}C NMR spectrum of 2k

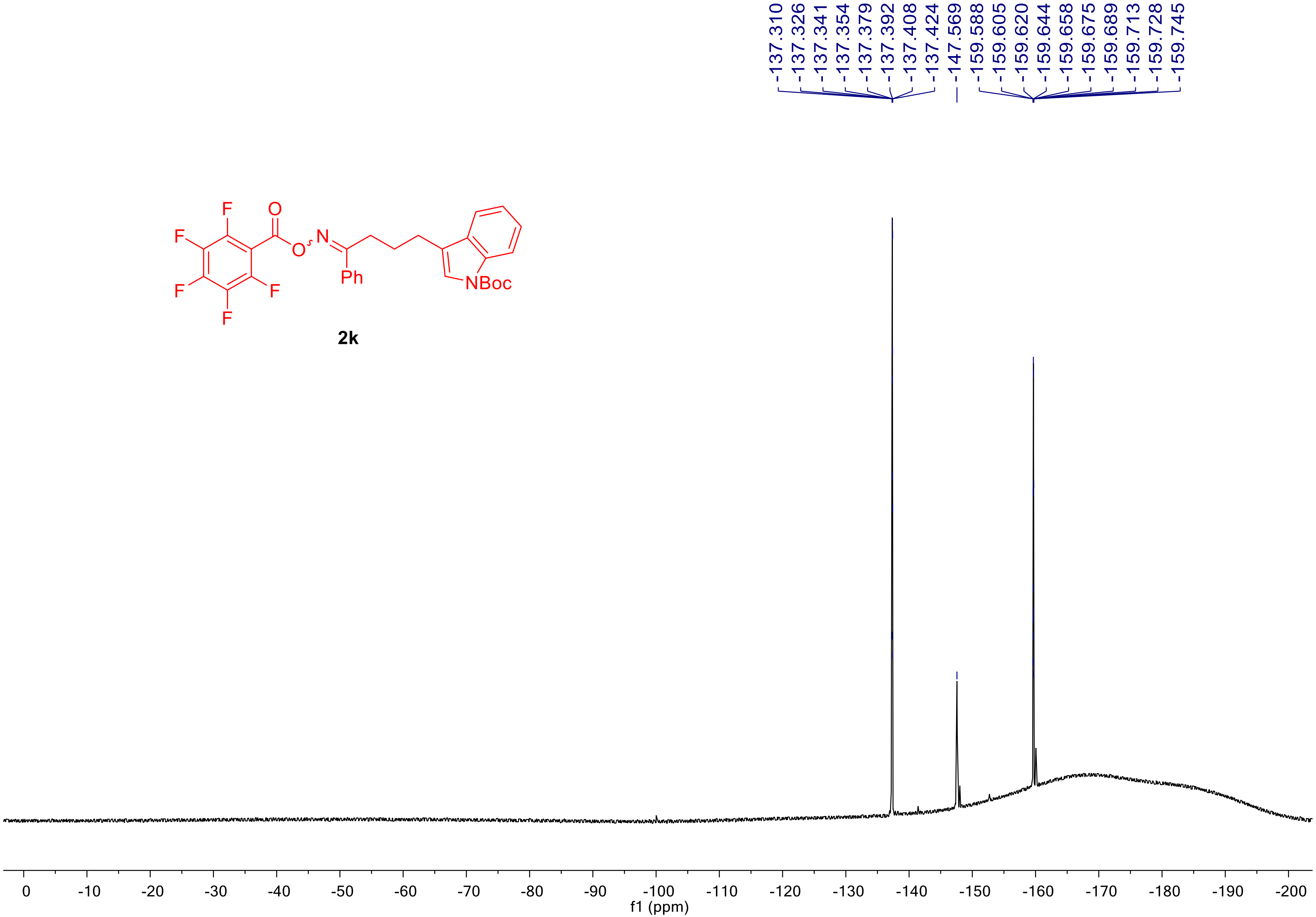

Supplementary Figure 54. ^{19}F NMR spectrum of 2k

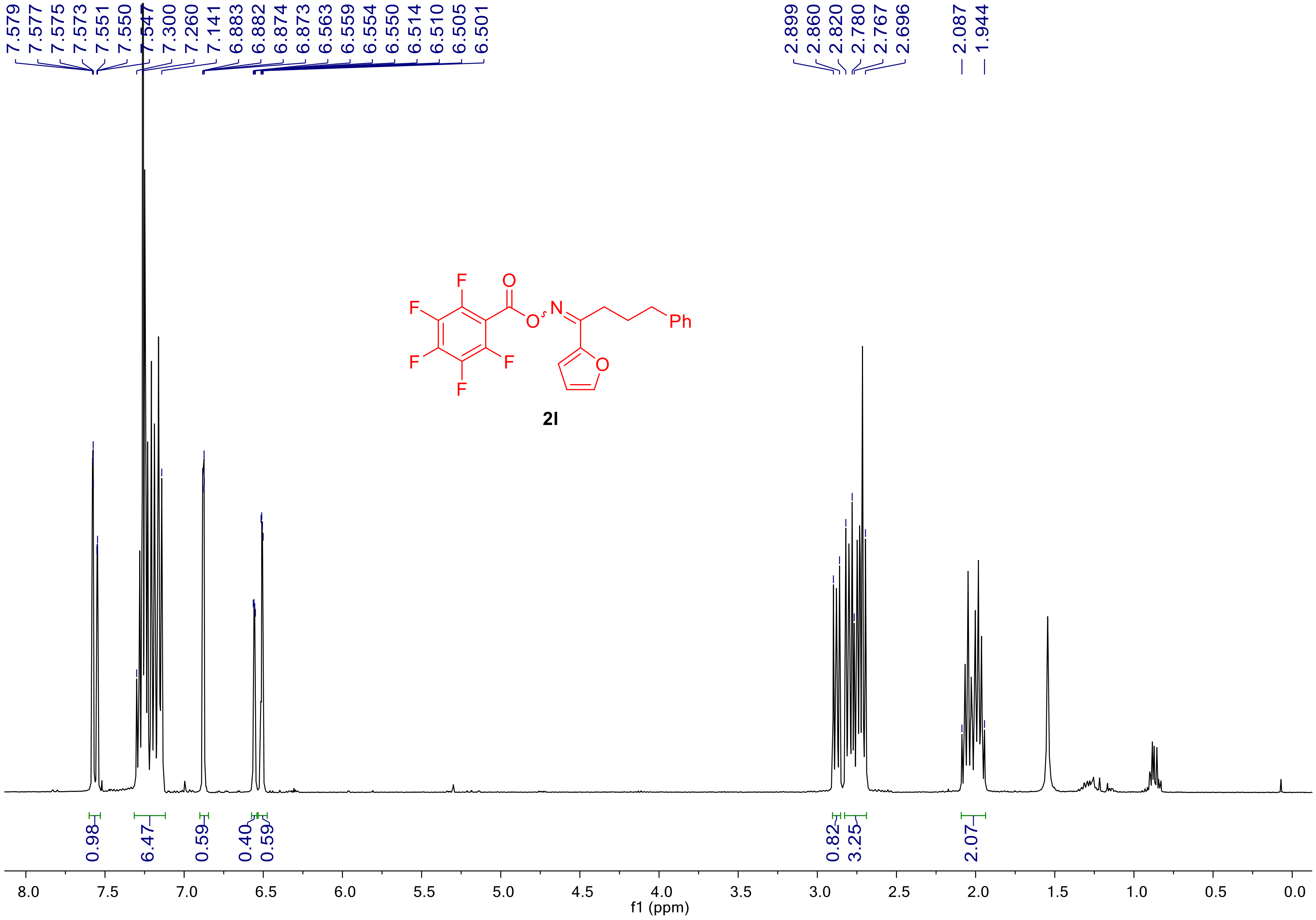

Supplementary Figure 55. ^{1}H NMR spectrum of 2l

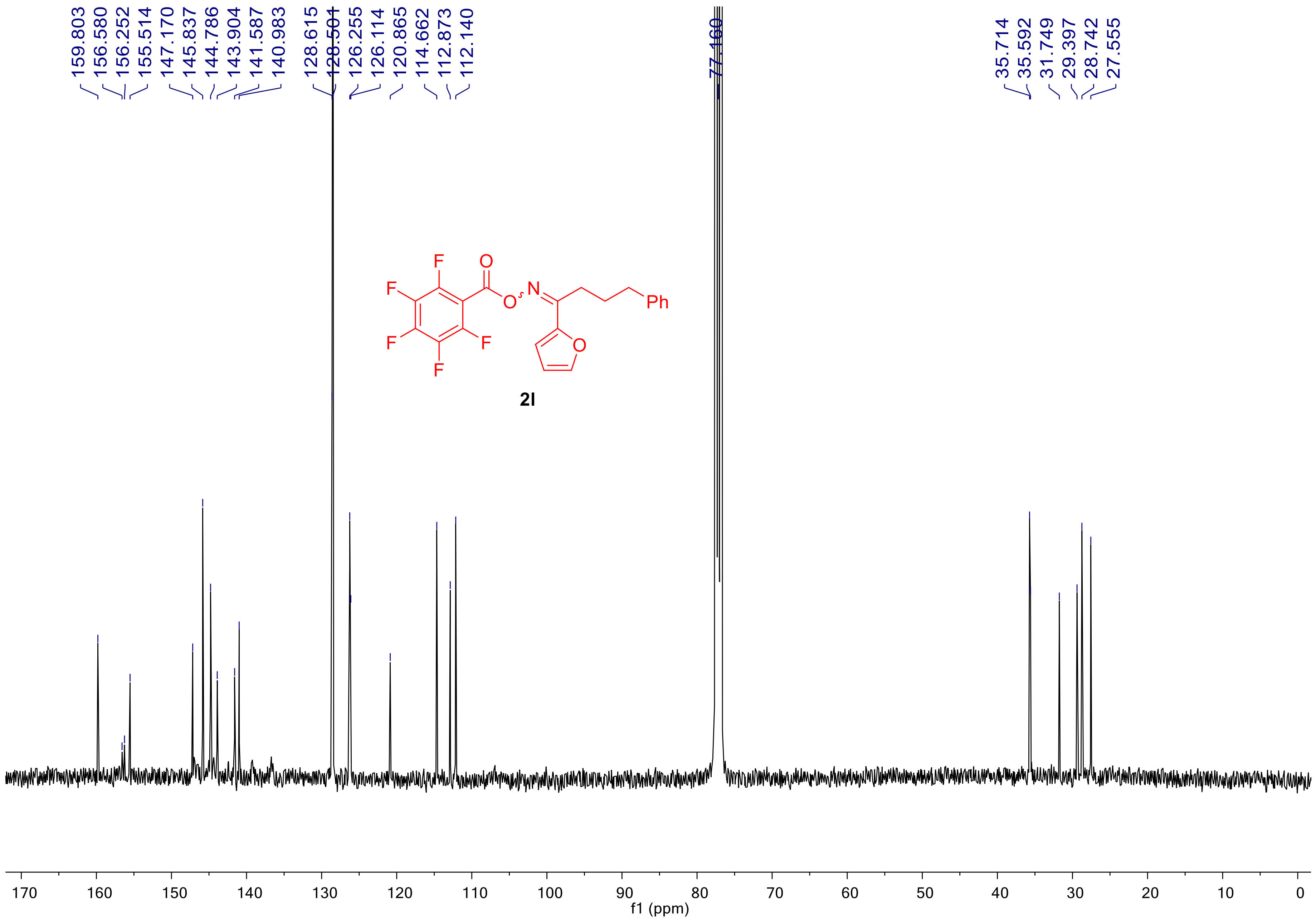

Supplementary Figure 56. ^{13}C NMR spectrum of 2l

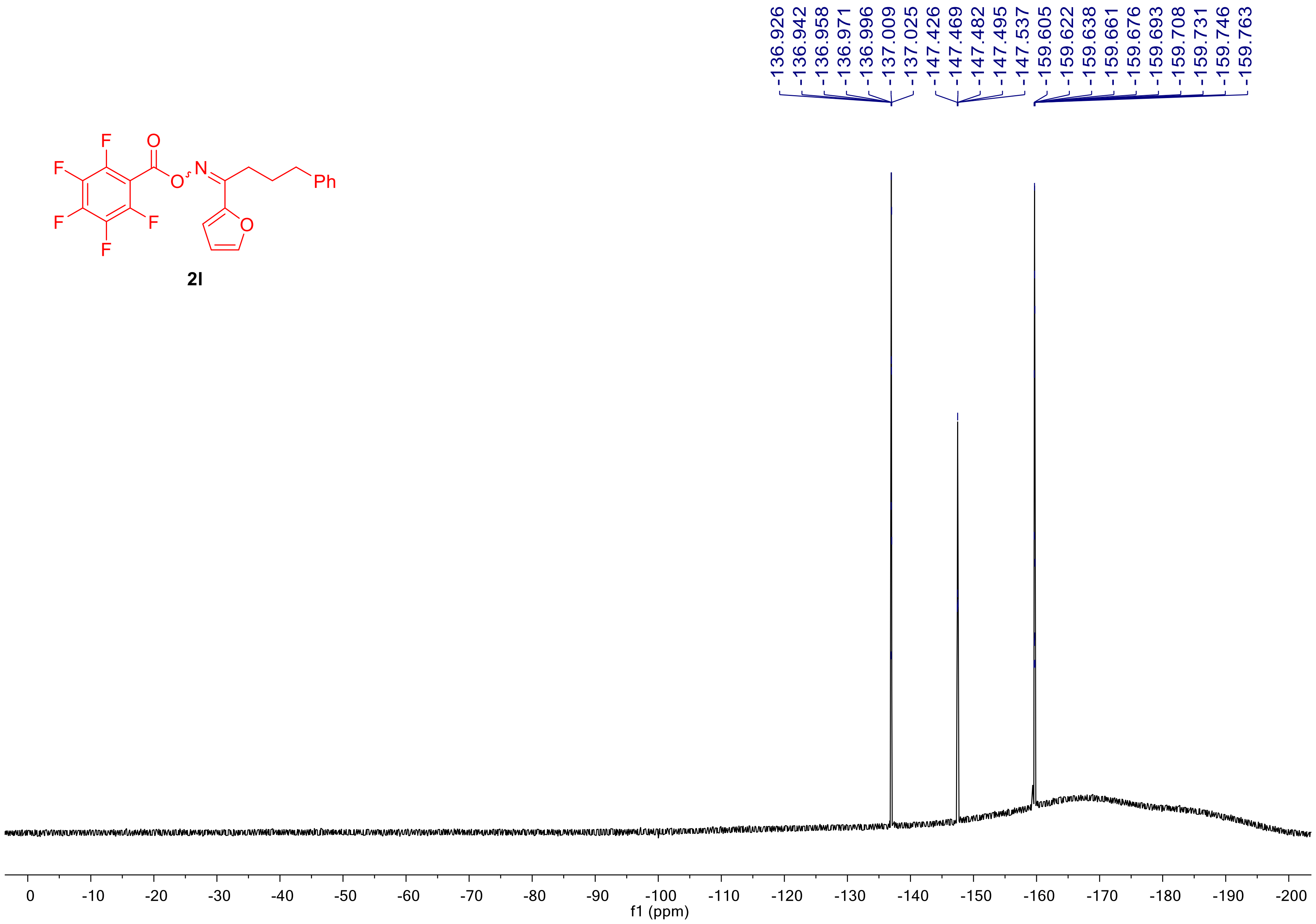

Supplementary Figure 57. ^{19}F NMR spectrum of 2l

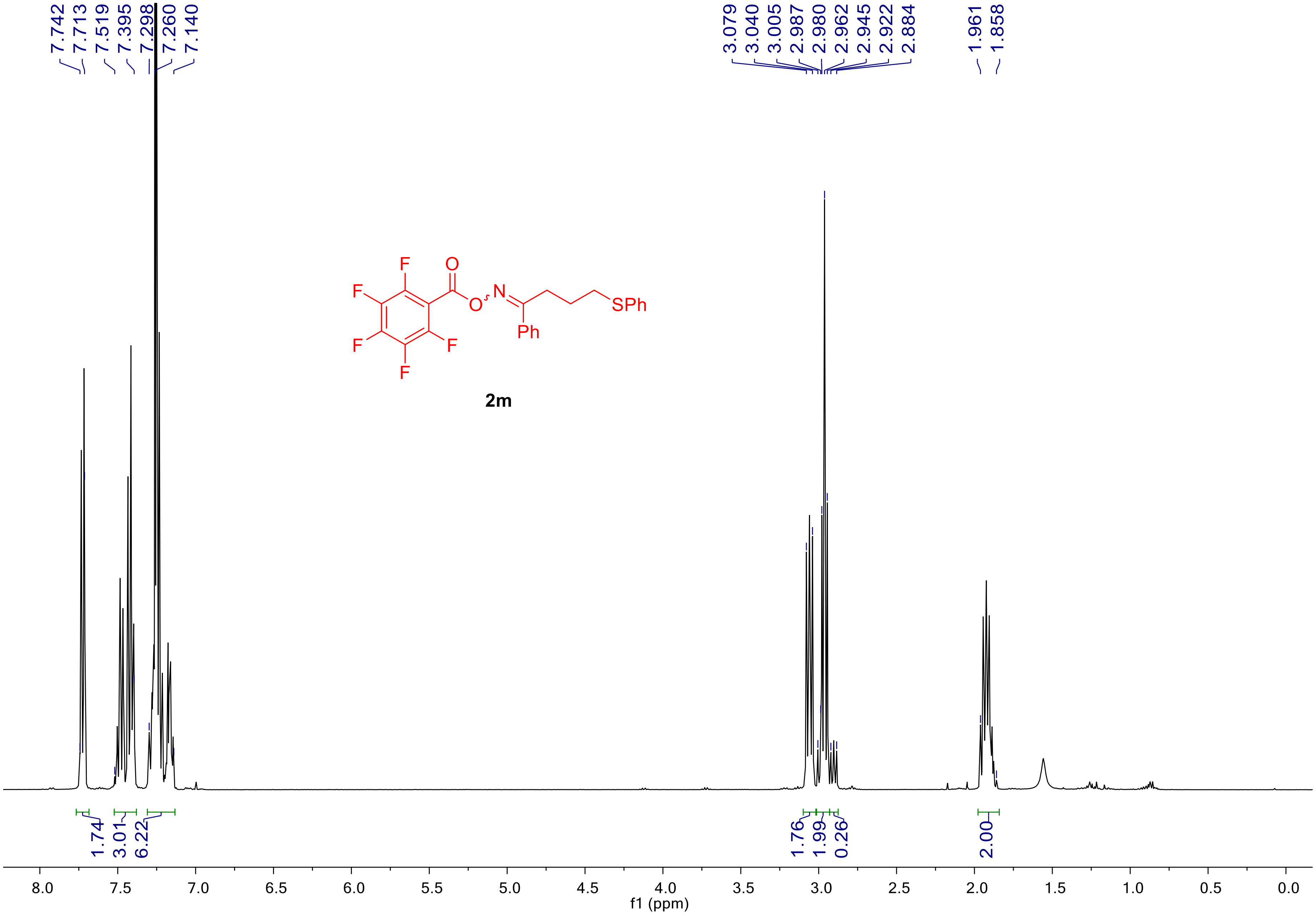

Supplementary Figure 58. ^{1}H NMR spectrum of 2m

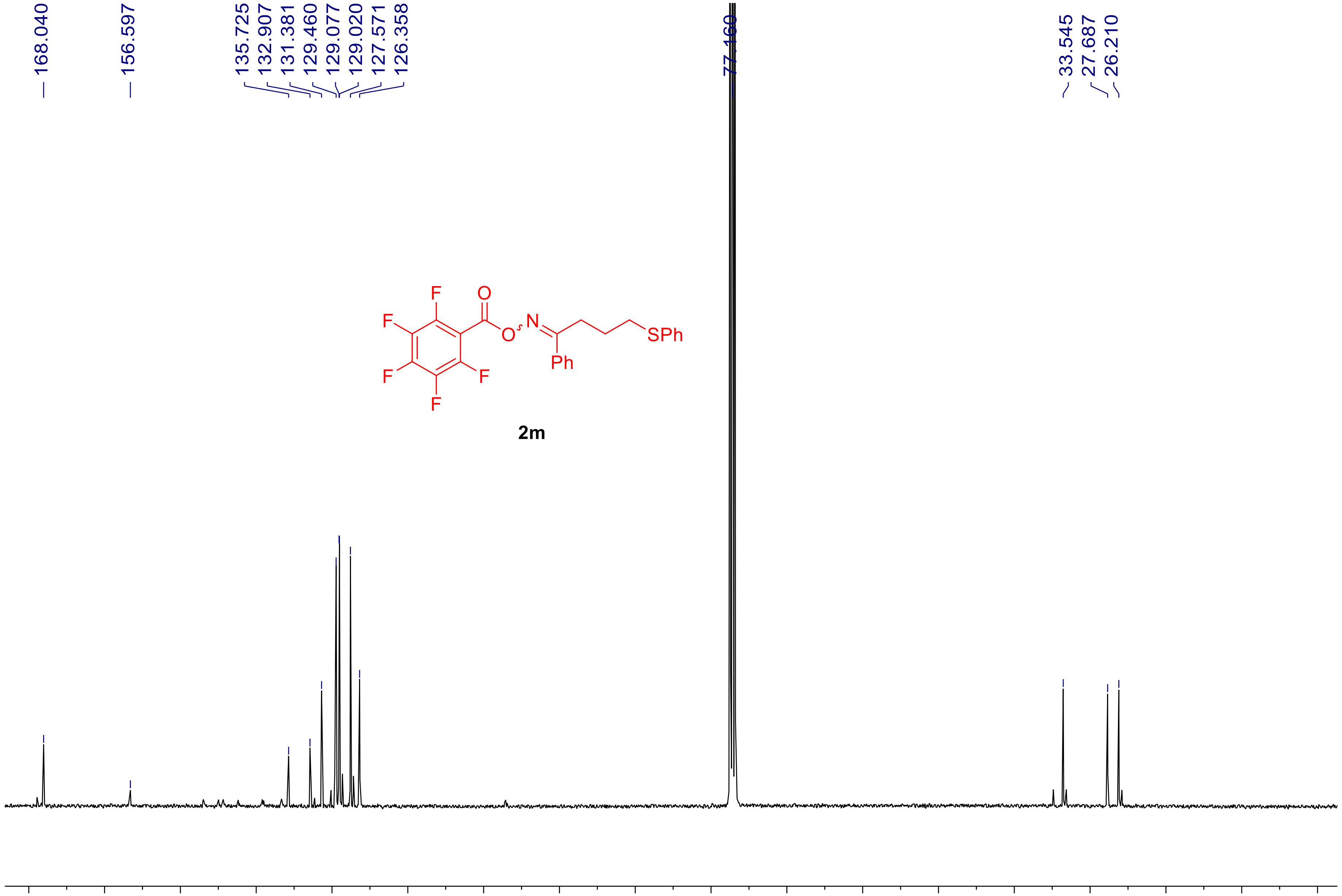

Supplementary Figure 59. ^{13}**C NMR spectrum of 2m**

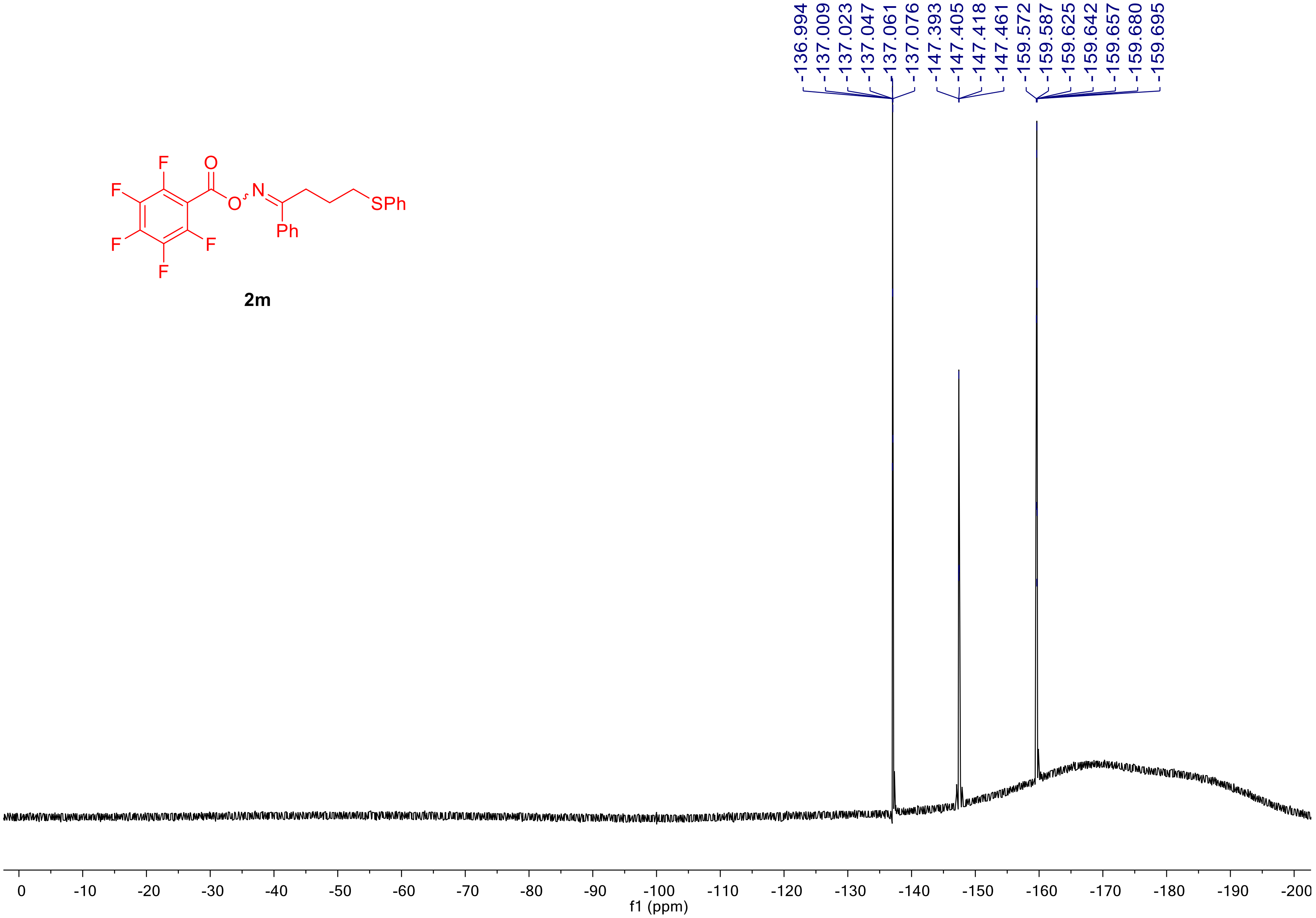

Supplementary Figure 60. ^{19}F NMR spectrum of 2m

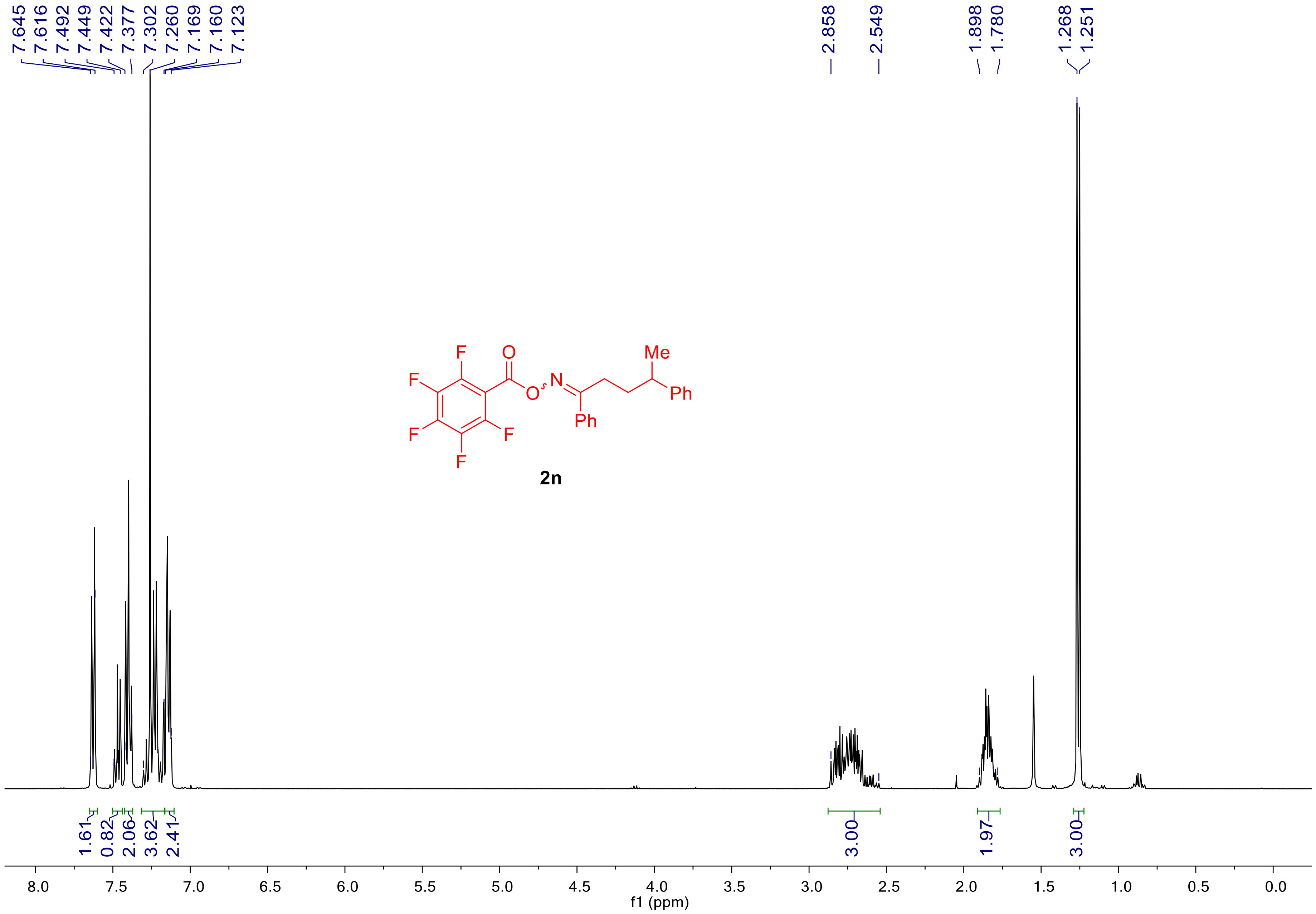

Supplementary Figure 61. ^{1}H NMR spectrum of 2n

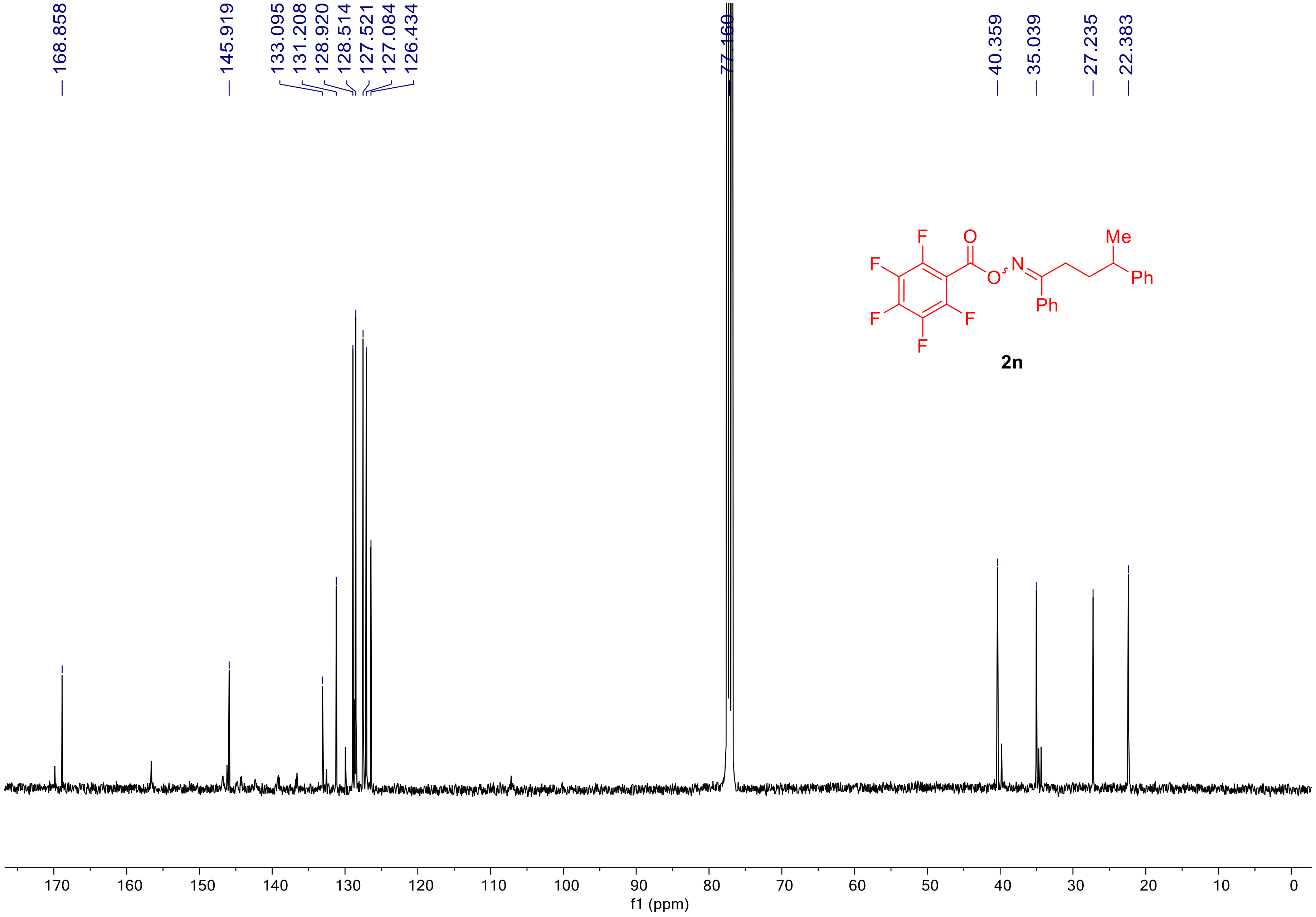

Supplementary Figure 62. ^{13}C NMR spectrum of 2n

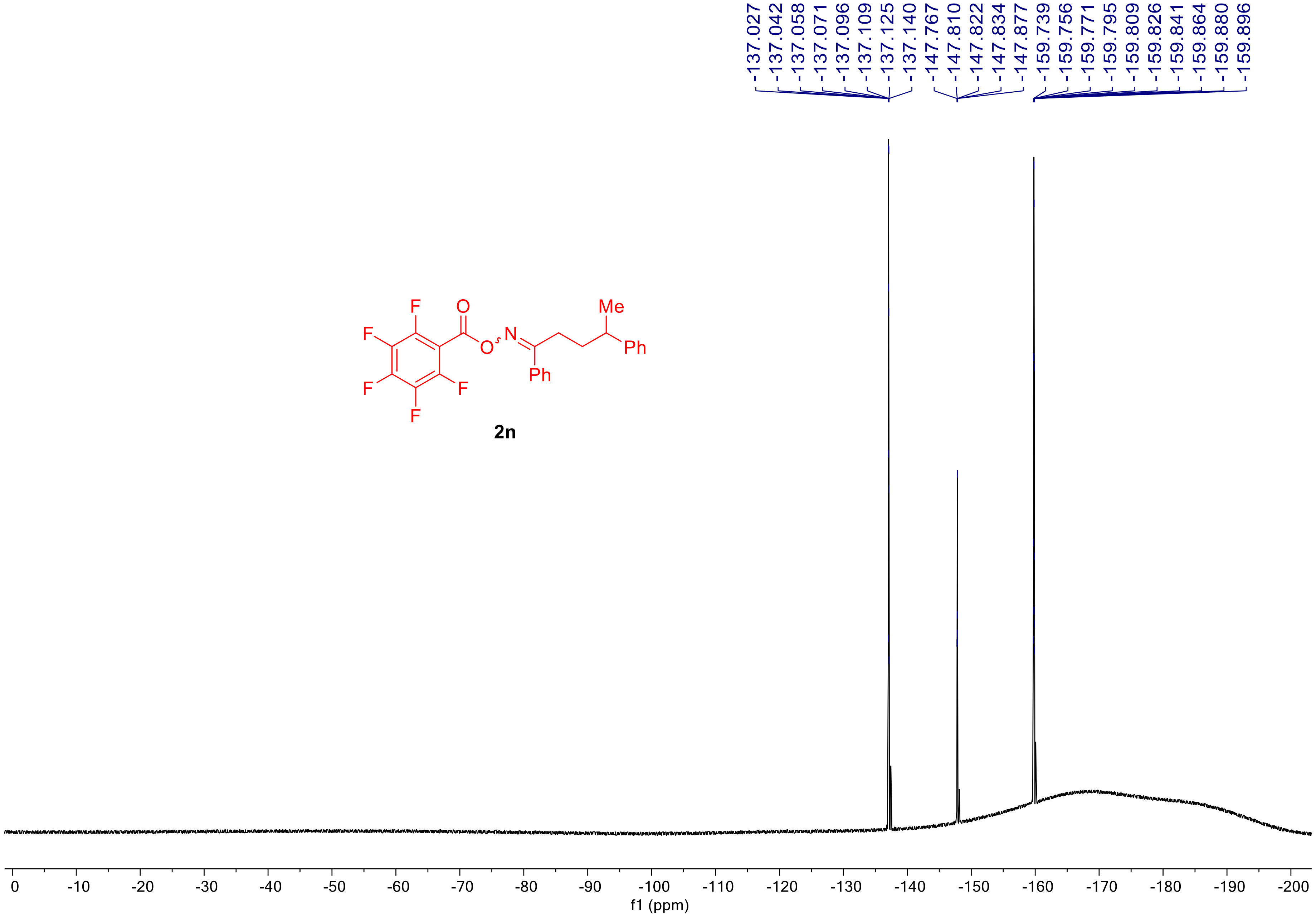

Supplementary Figure 63. ^{19}F NMR spectrum of 2n

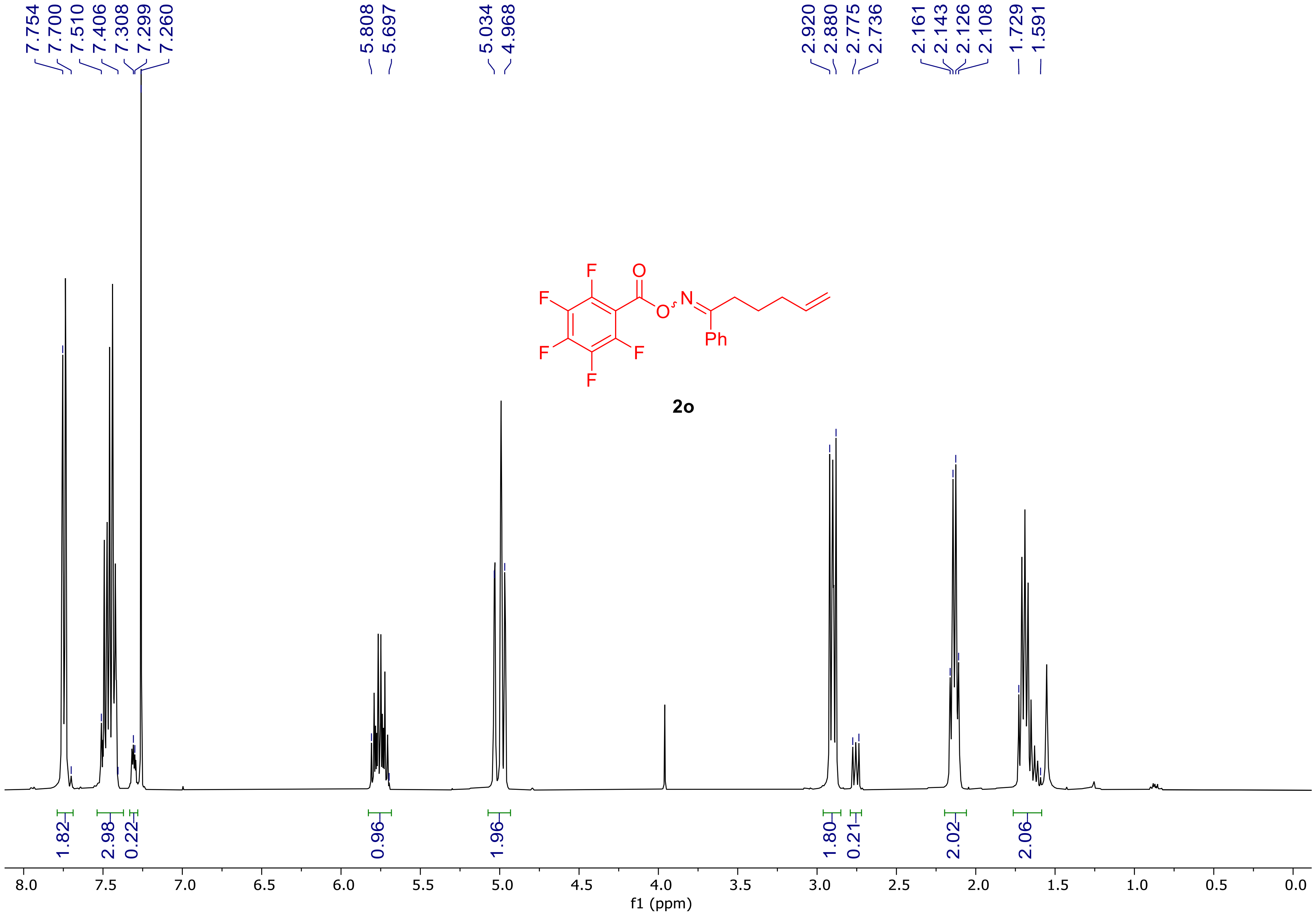

Supplementary Figure 64. ^{1}H NMR spectrum of 2o

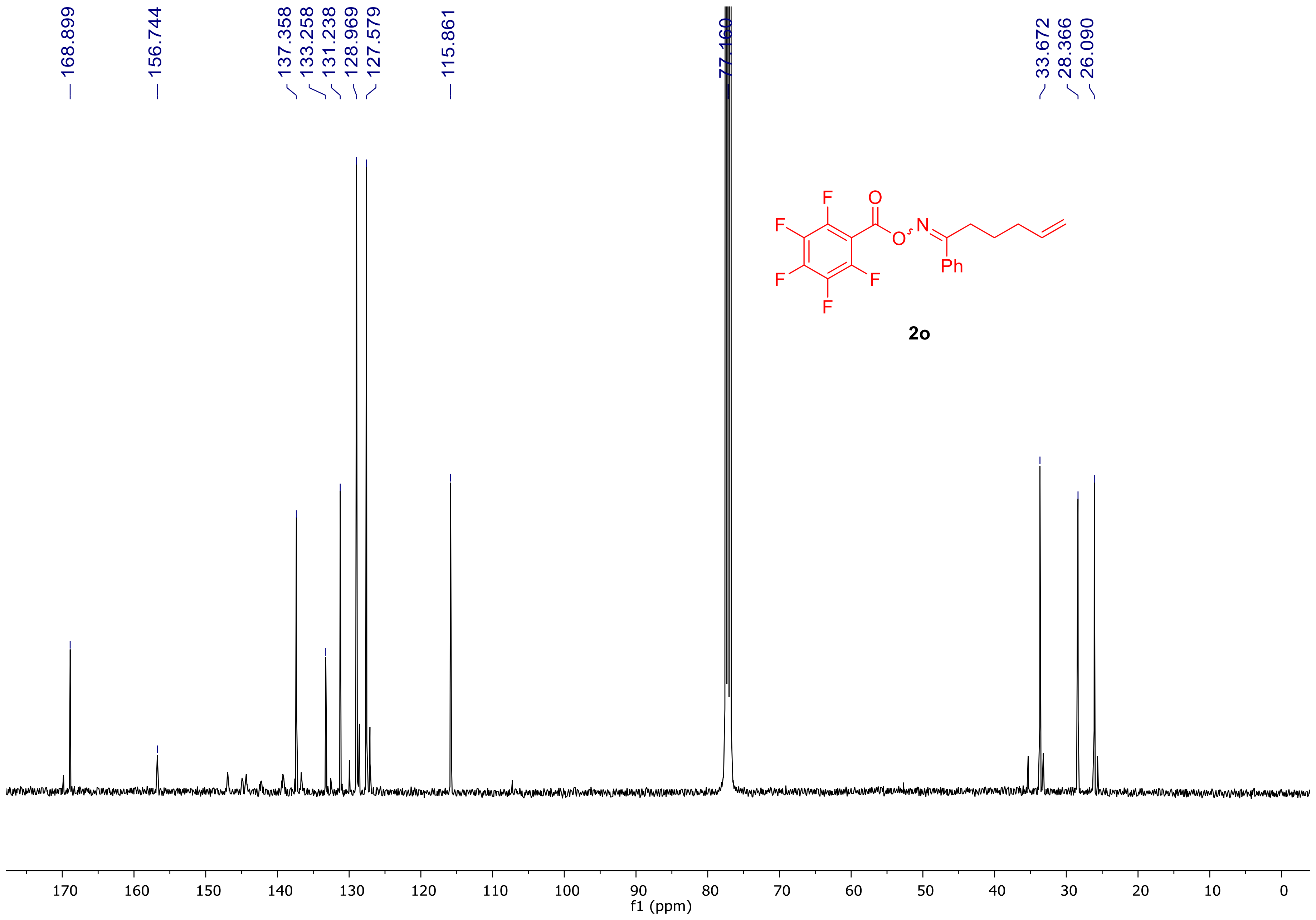

Supplementary Figure 65. ^{13}C NMR spectrum of 2o

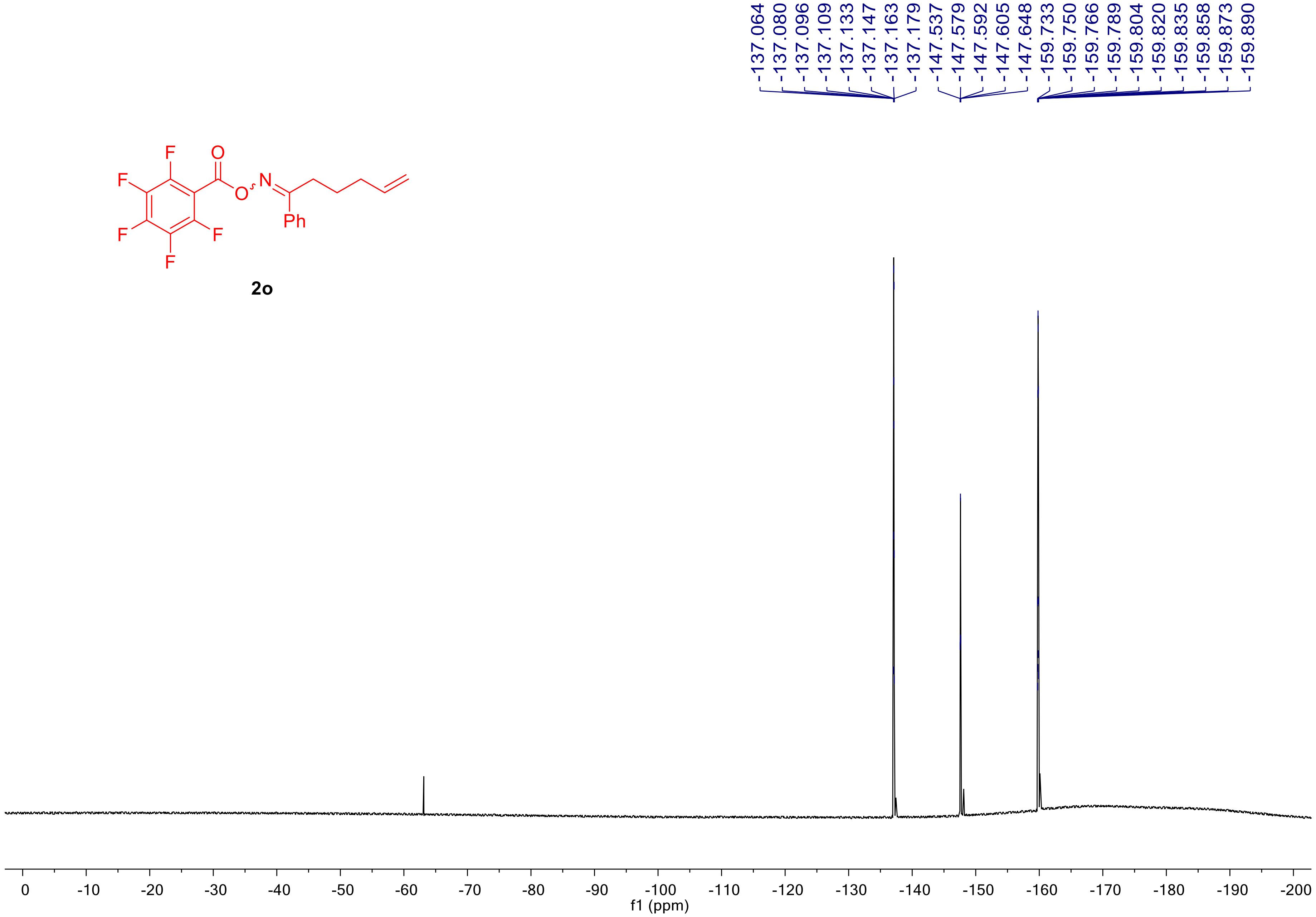

Supplementary Figure 66. ^{19}F NMR spectrum of 2o

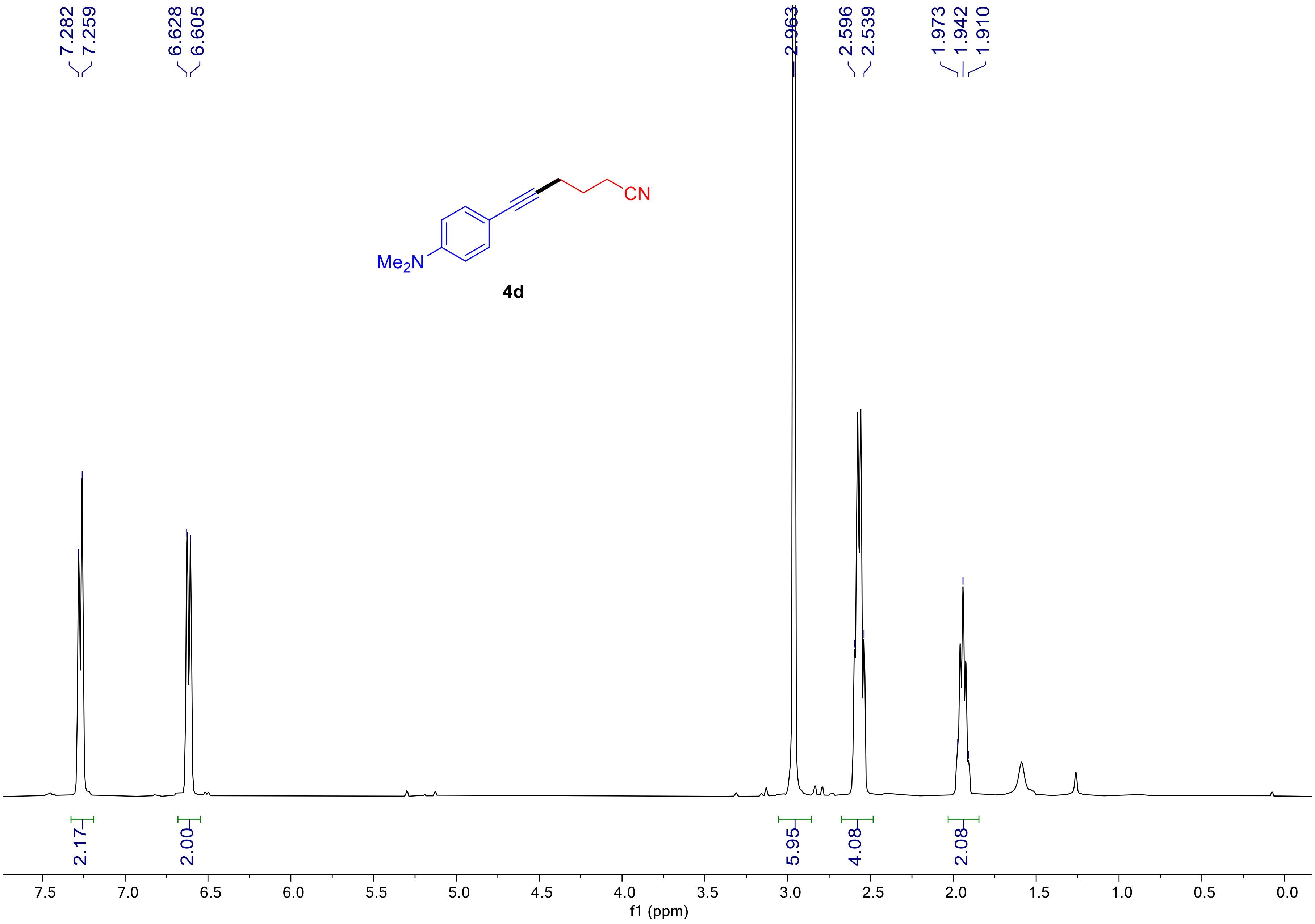

Supplementary Figure 67. ^{1}H NMR spectrum of 4d

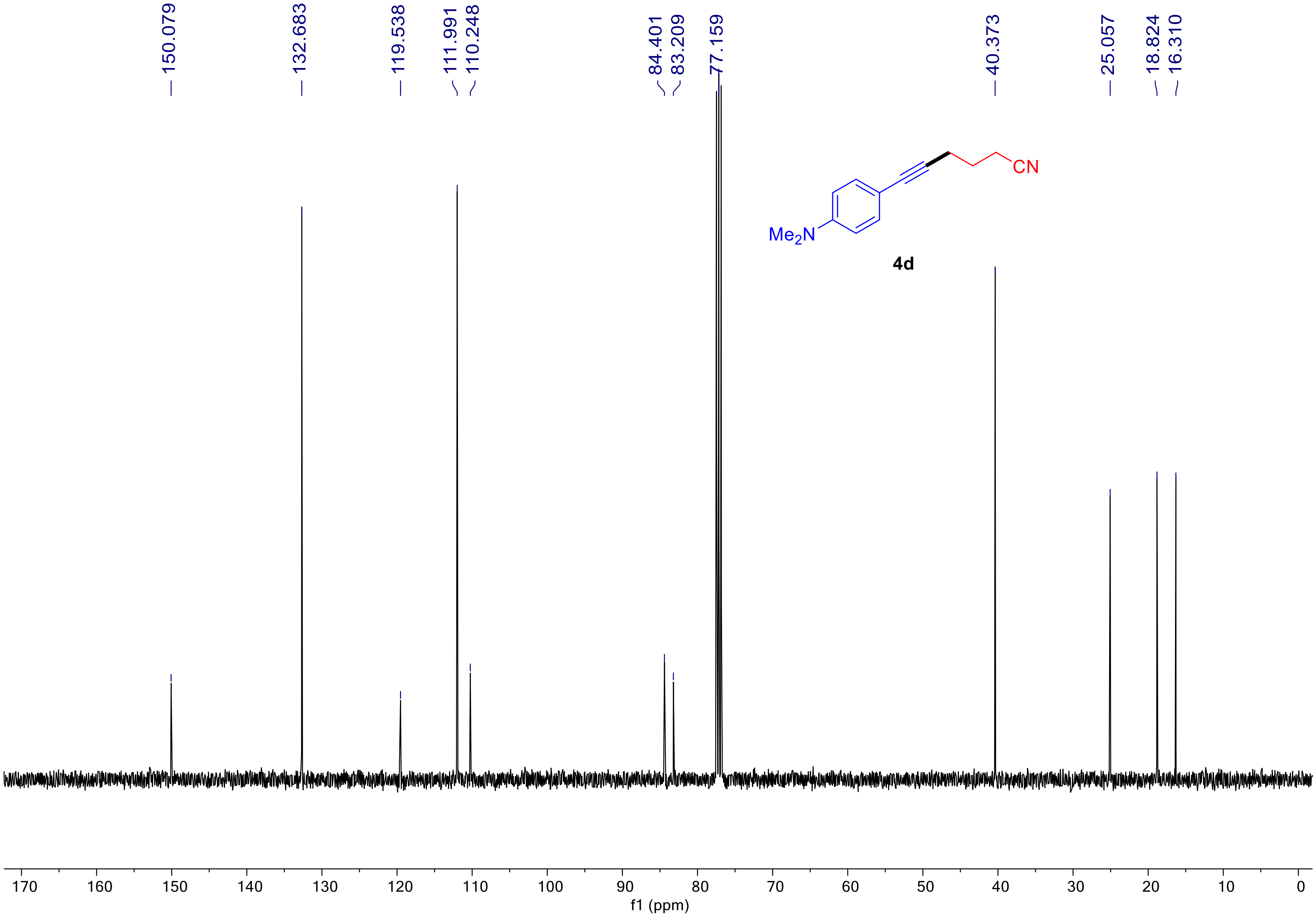

Supplementary Figure 68. ^{13}C NMR spectrum of 4d

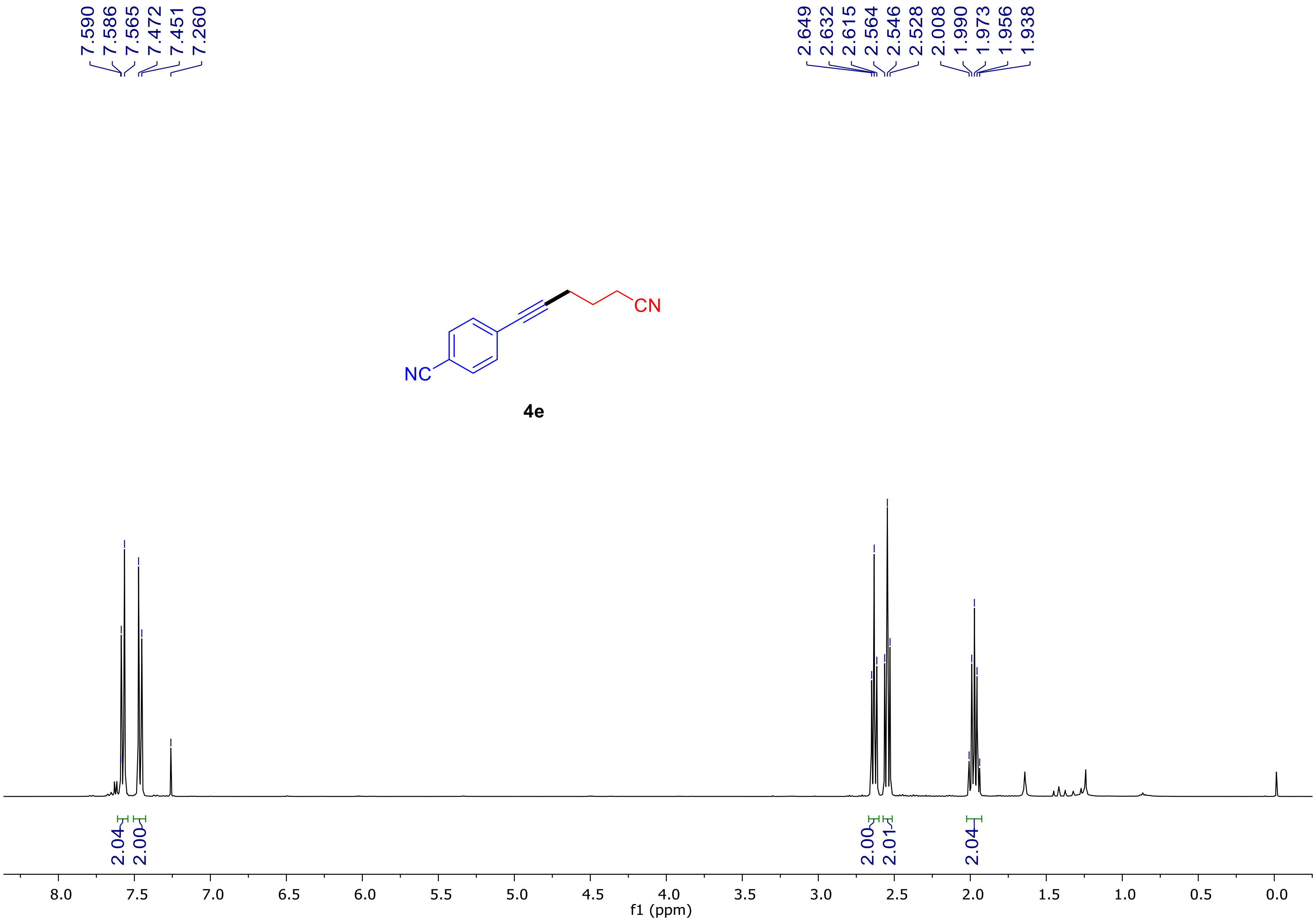

Supplementary Figure 69. ^{1}H NMR spectrum of 4e

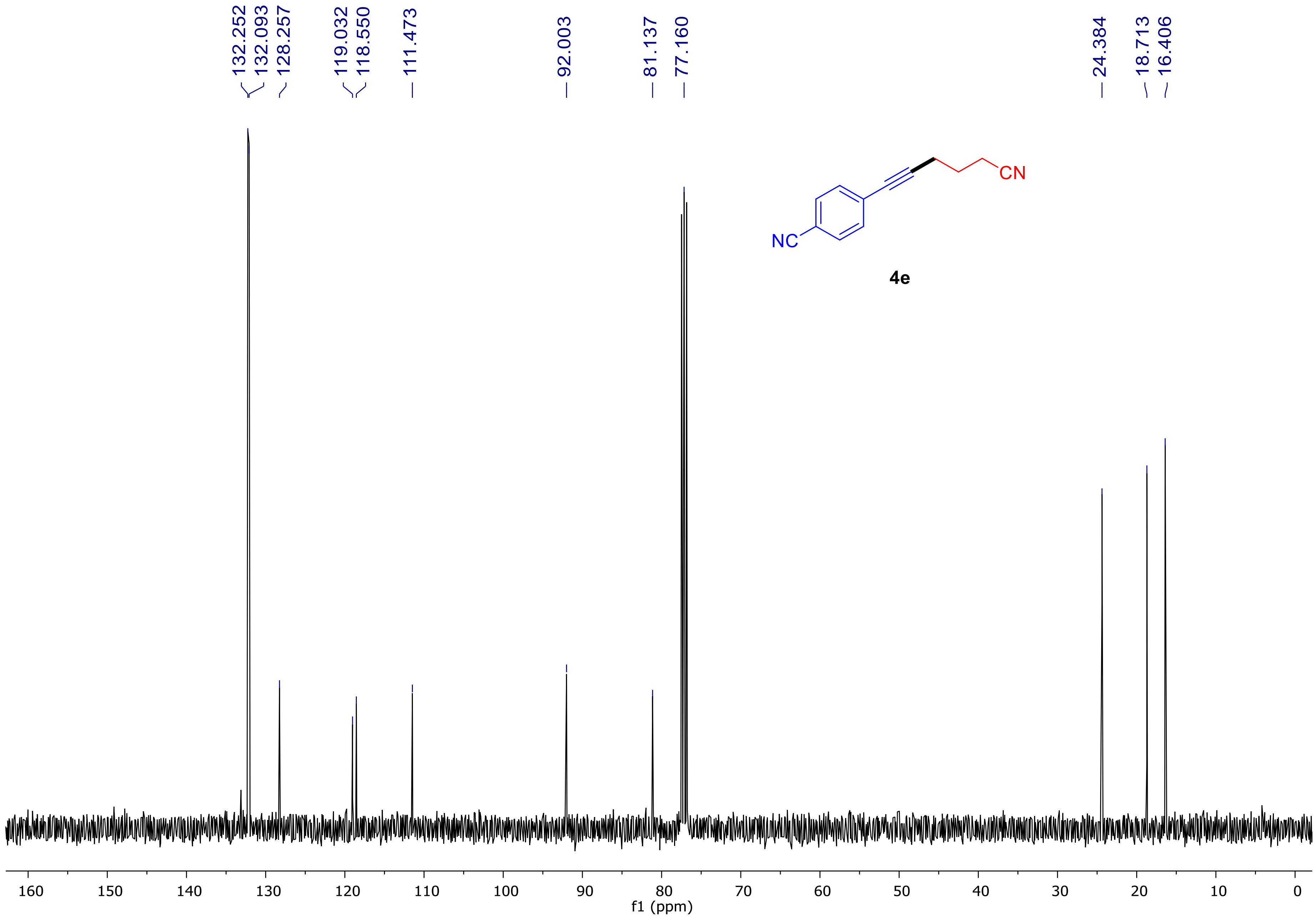

Supplementary Figure 70. ^{13}C NMR spectrum of 4e

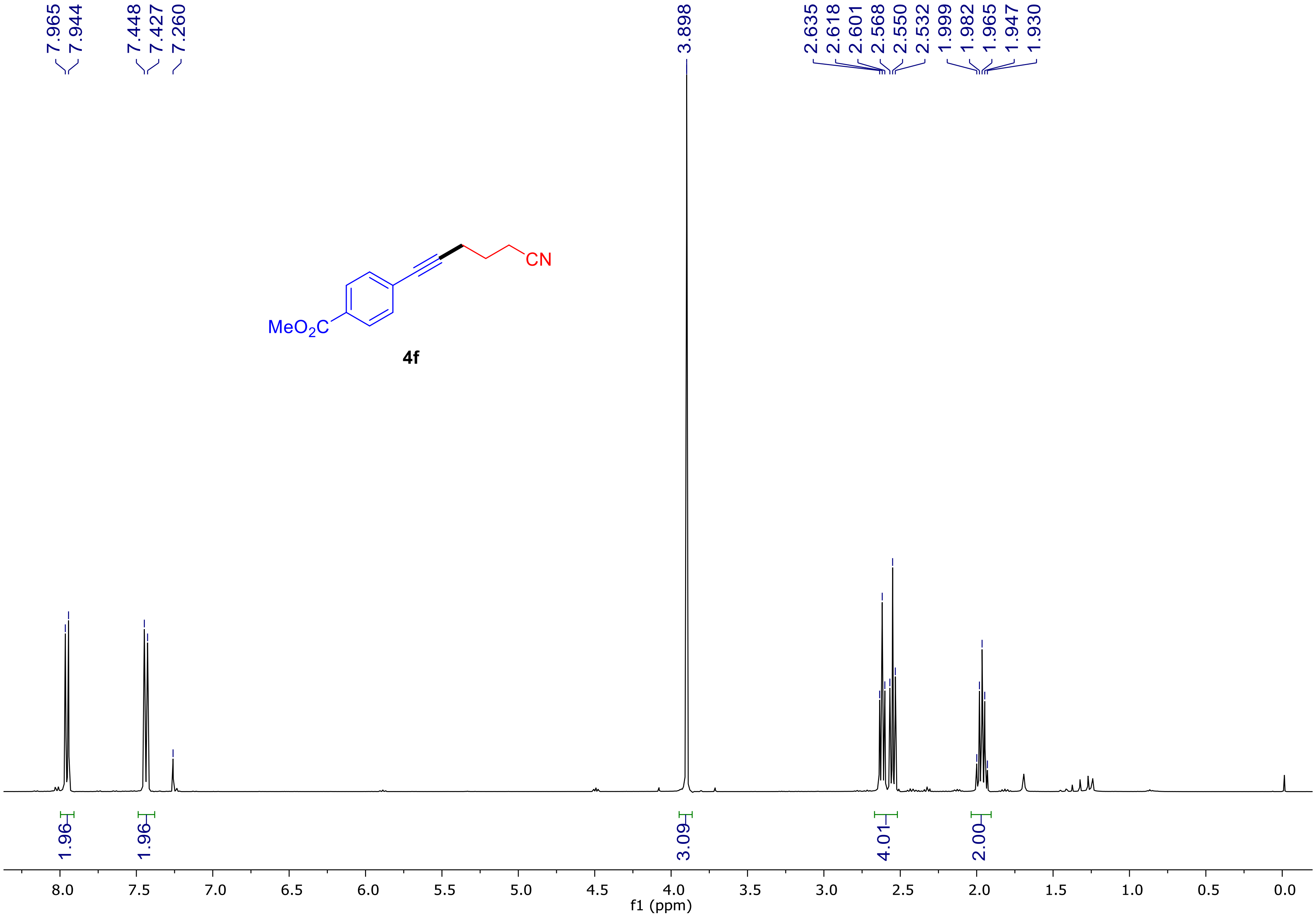

Supplementary Figure 71. ^{1}**H NMR spectrum of 4f**

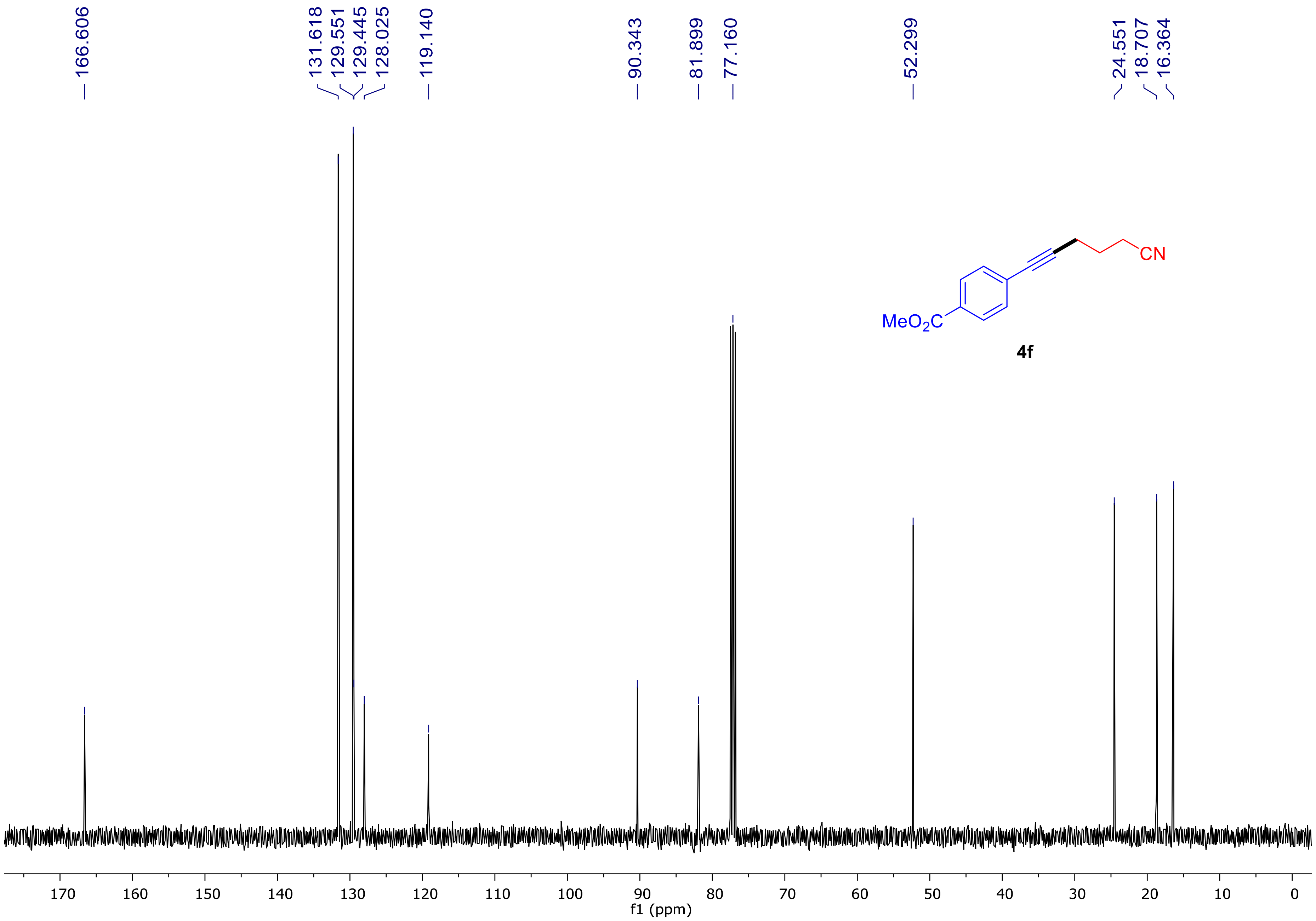

Supplementary Figure 72. ^{13}C NMR spectrum of 4f

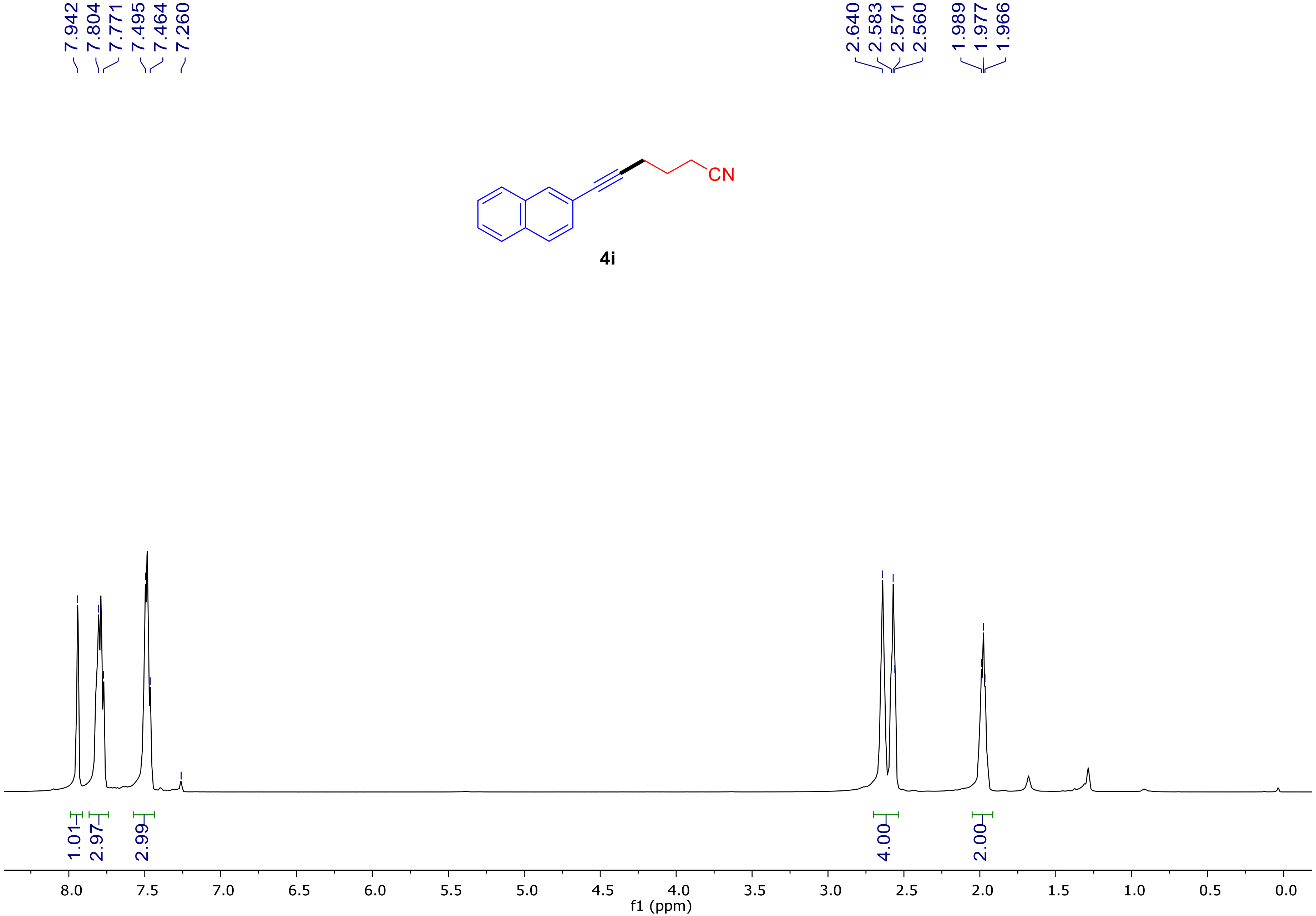

Supplementary Figure 73. ^{1}H NMR spectrum of 4i

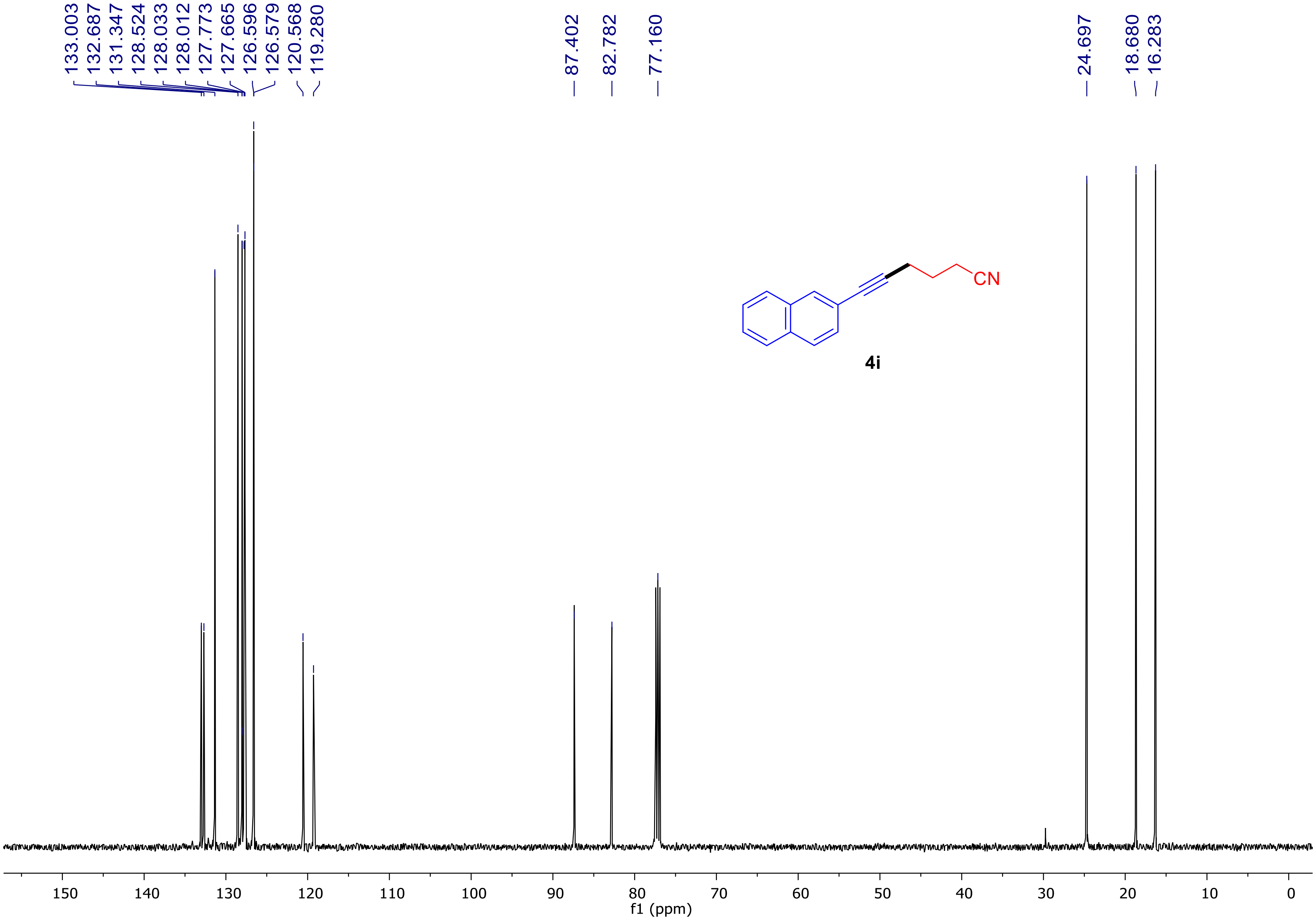

Supplementary Figure 74. ^{13}C NMR spectrum of 4i

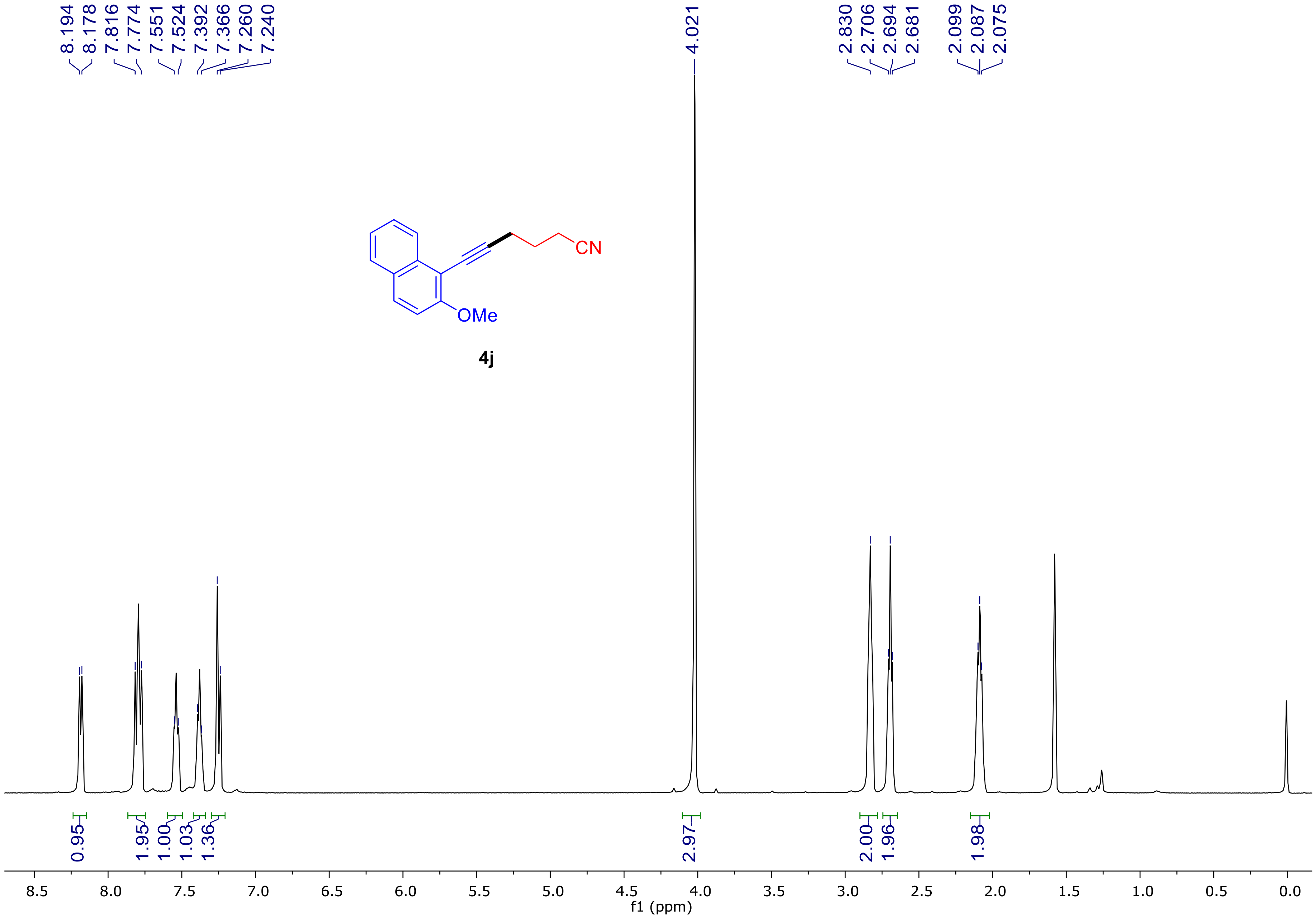

Supplementary Figure 75. ^{1}H NMR spectrum of 4j

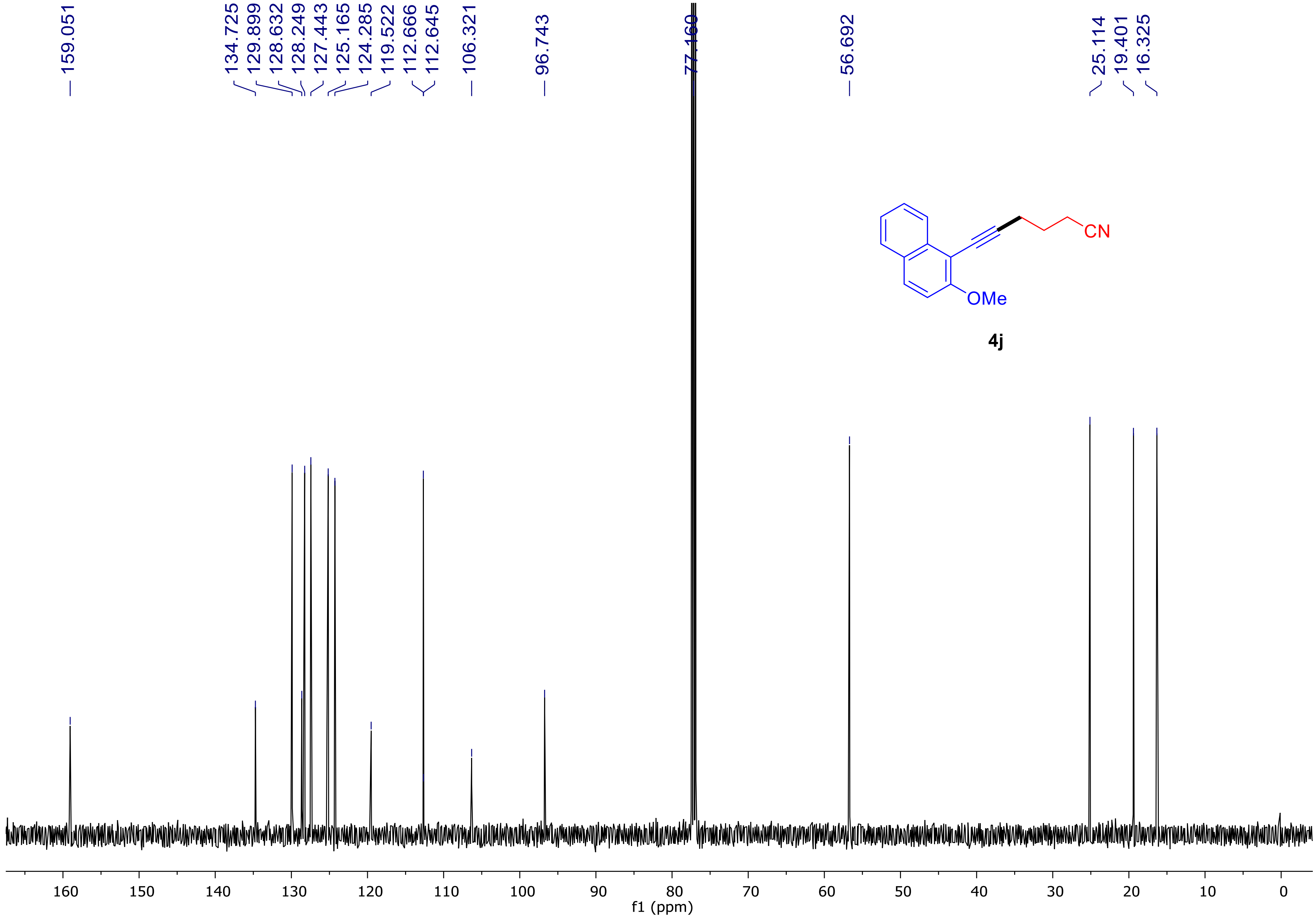

Supplementary Figure 76. ^{13}**C NMR spectrum of 4j**

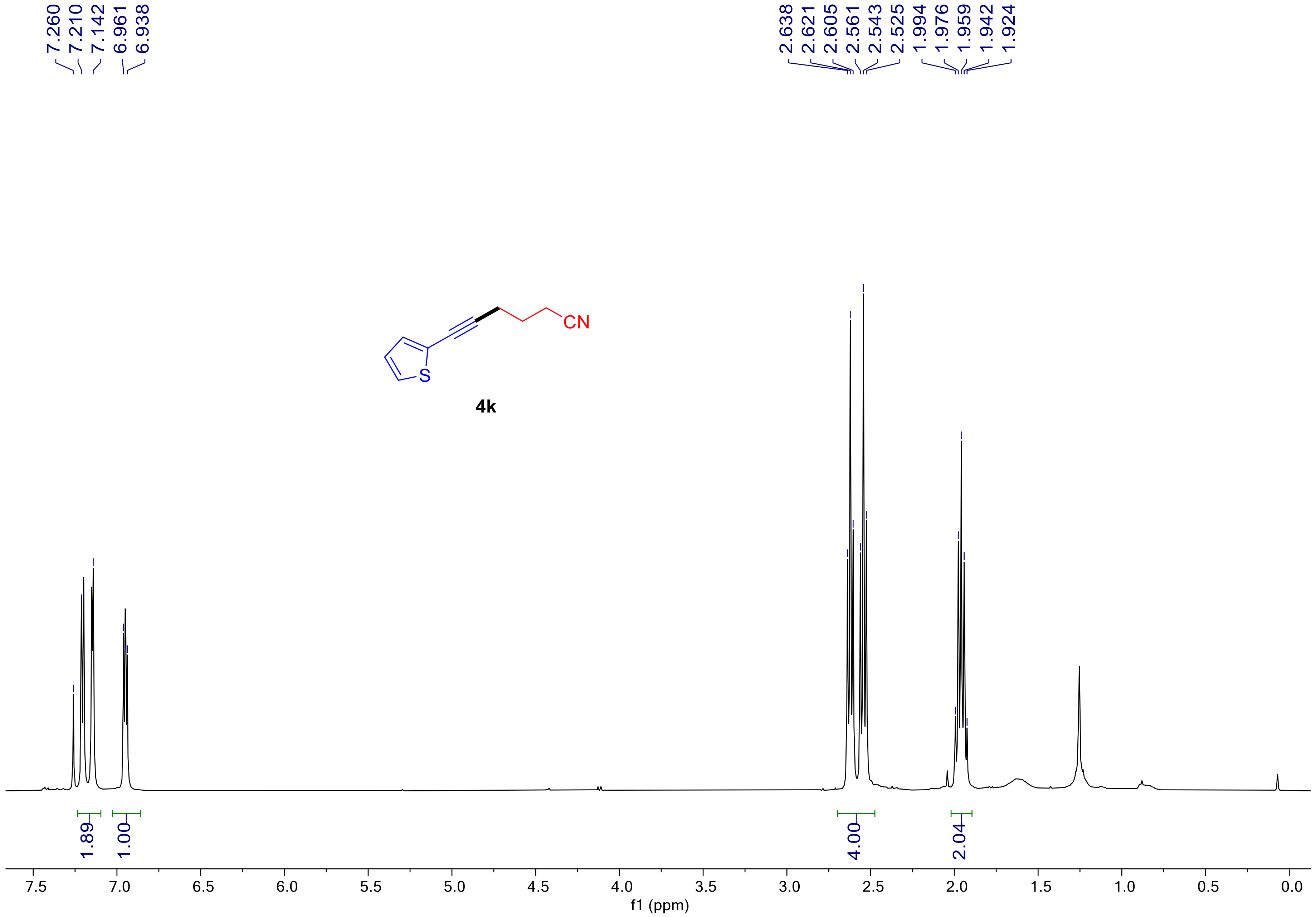

Supplementary Figure 77. ^{1}H NMR spectrum of 4k

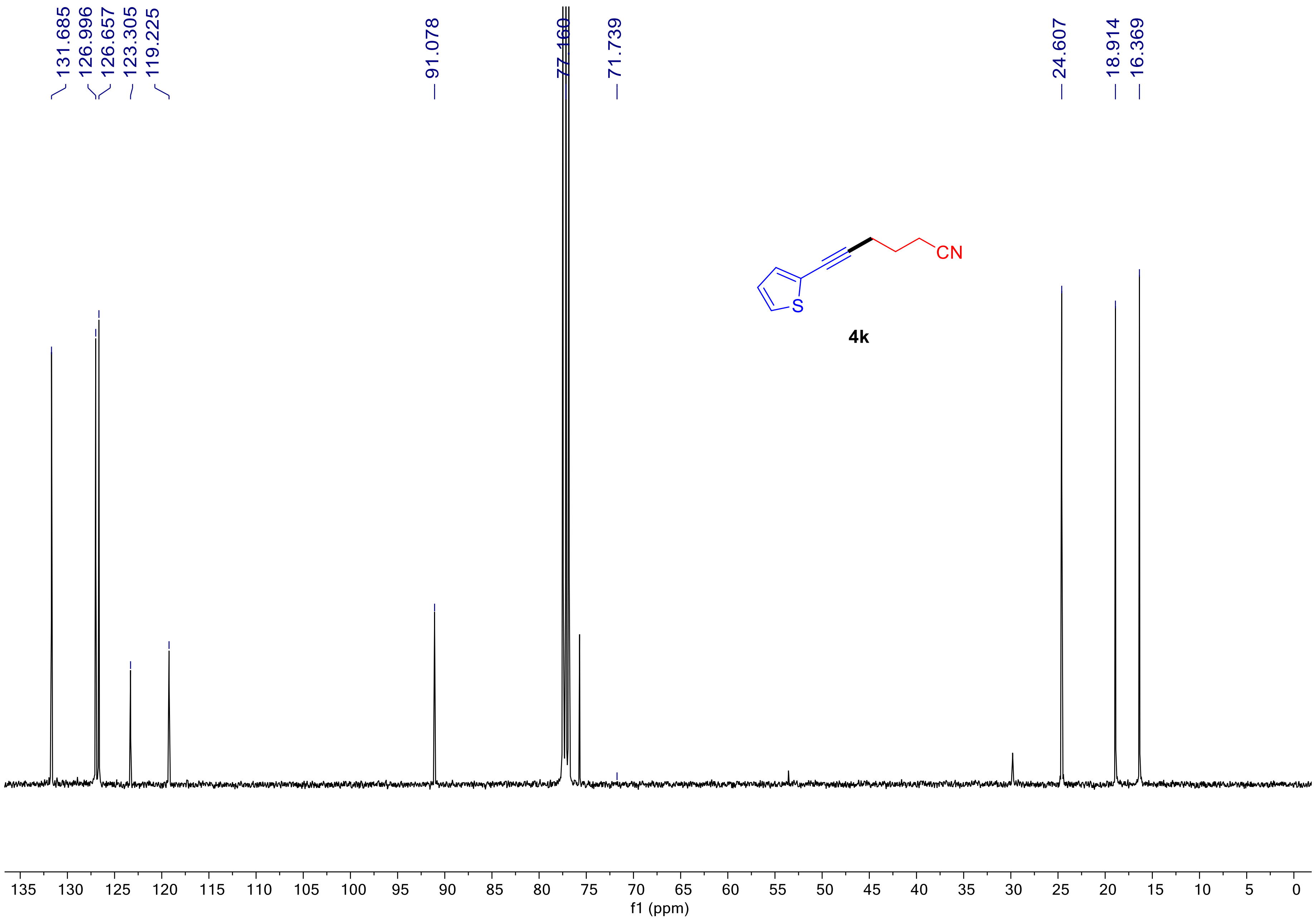

Supplementary Figure 78. ^{13}C NMR spectrum of 4k

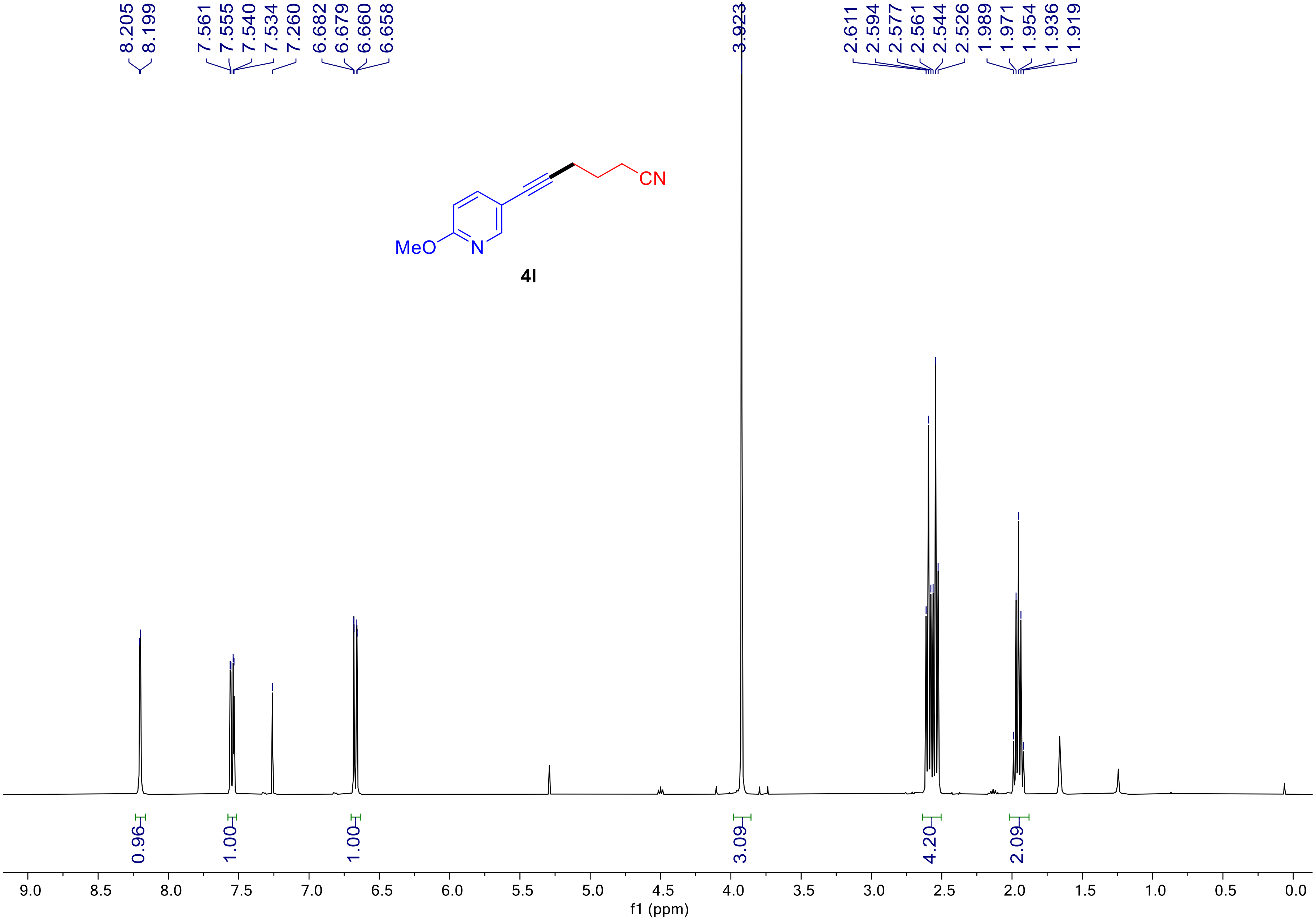

Supplementary Figure 79. ^{1}H NMR spectrum of 4l

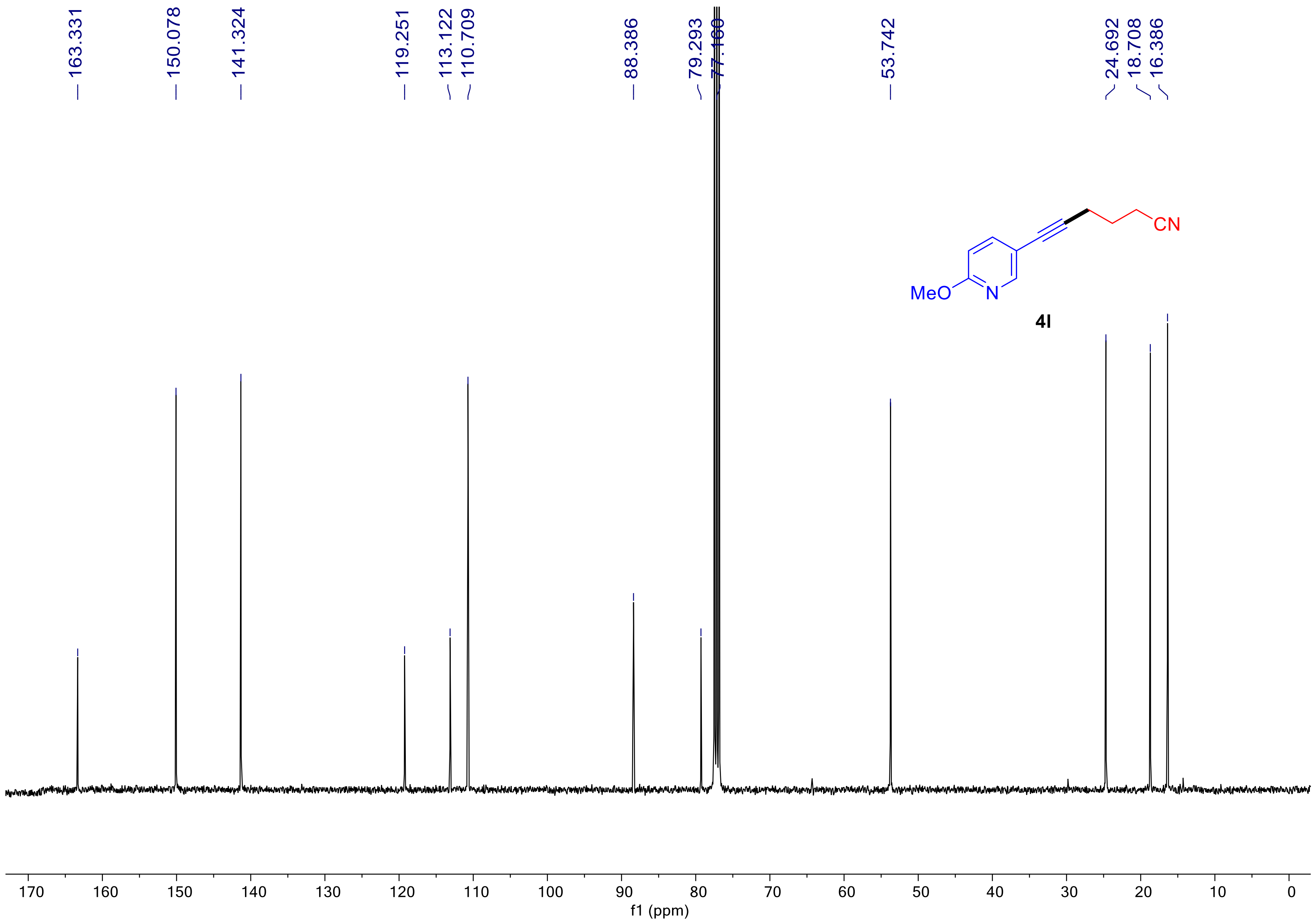

Supplementary Figure 80. ^{13}C NMR spectrum of 4l

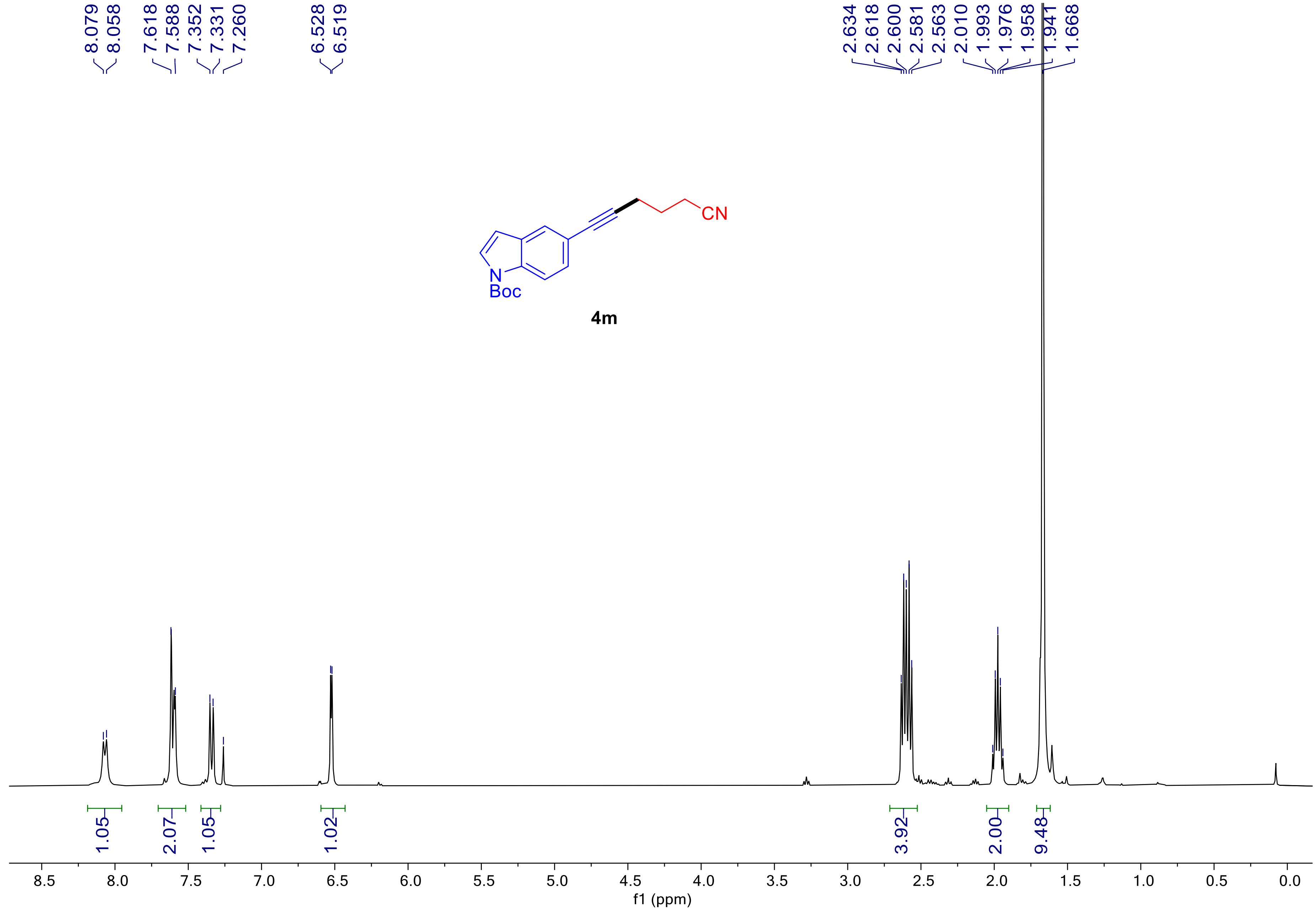

Supplementary Figure 81. ^{1}**H NMR spectrum of 4m**

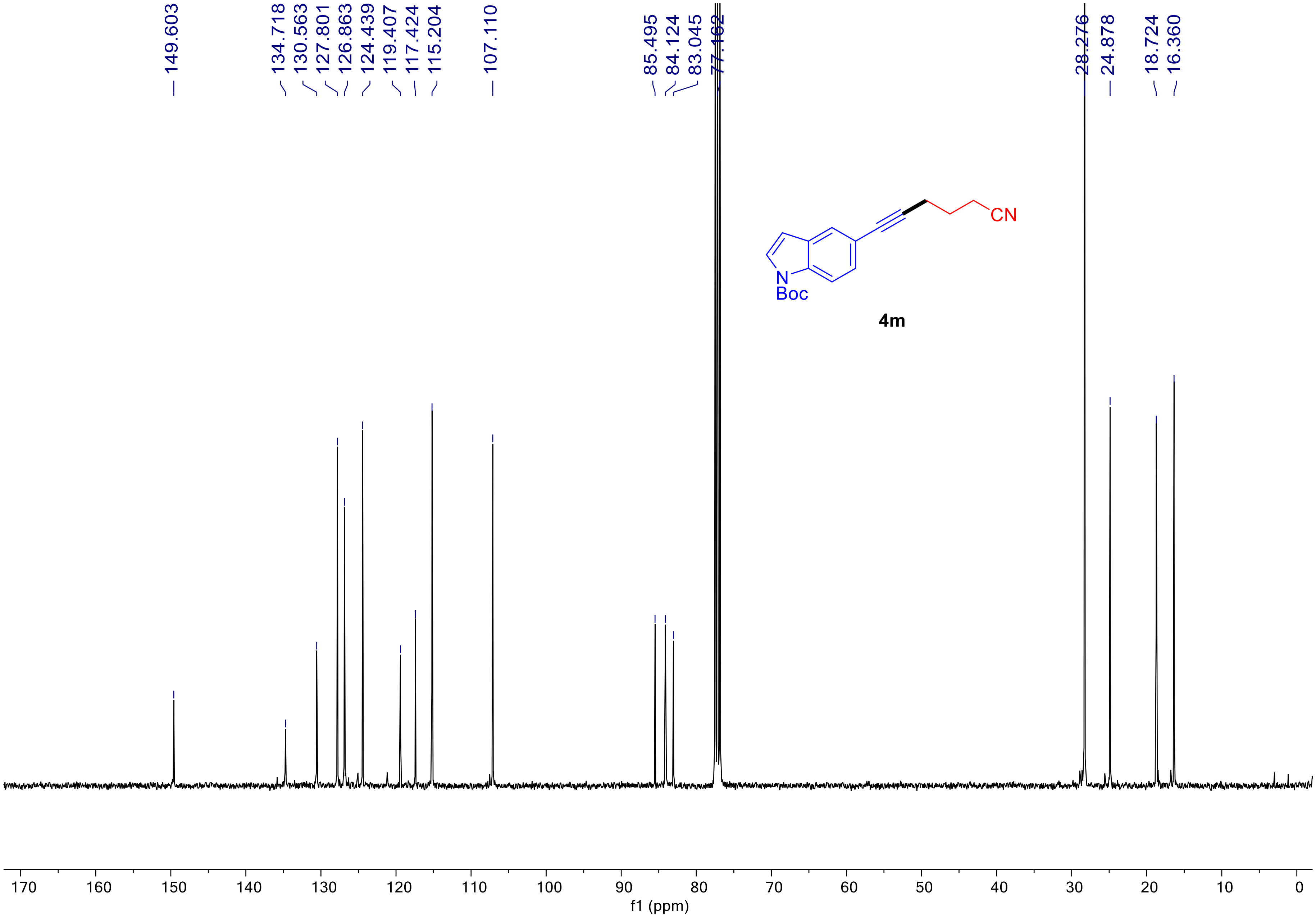

Supplementary Figure 82. ^{13}**C NMR spectrum of 4m**

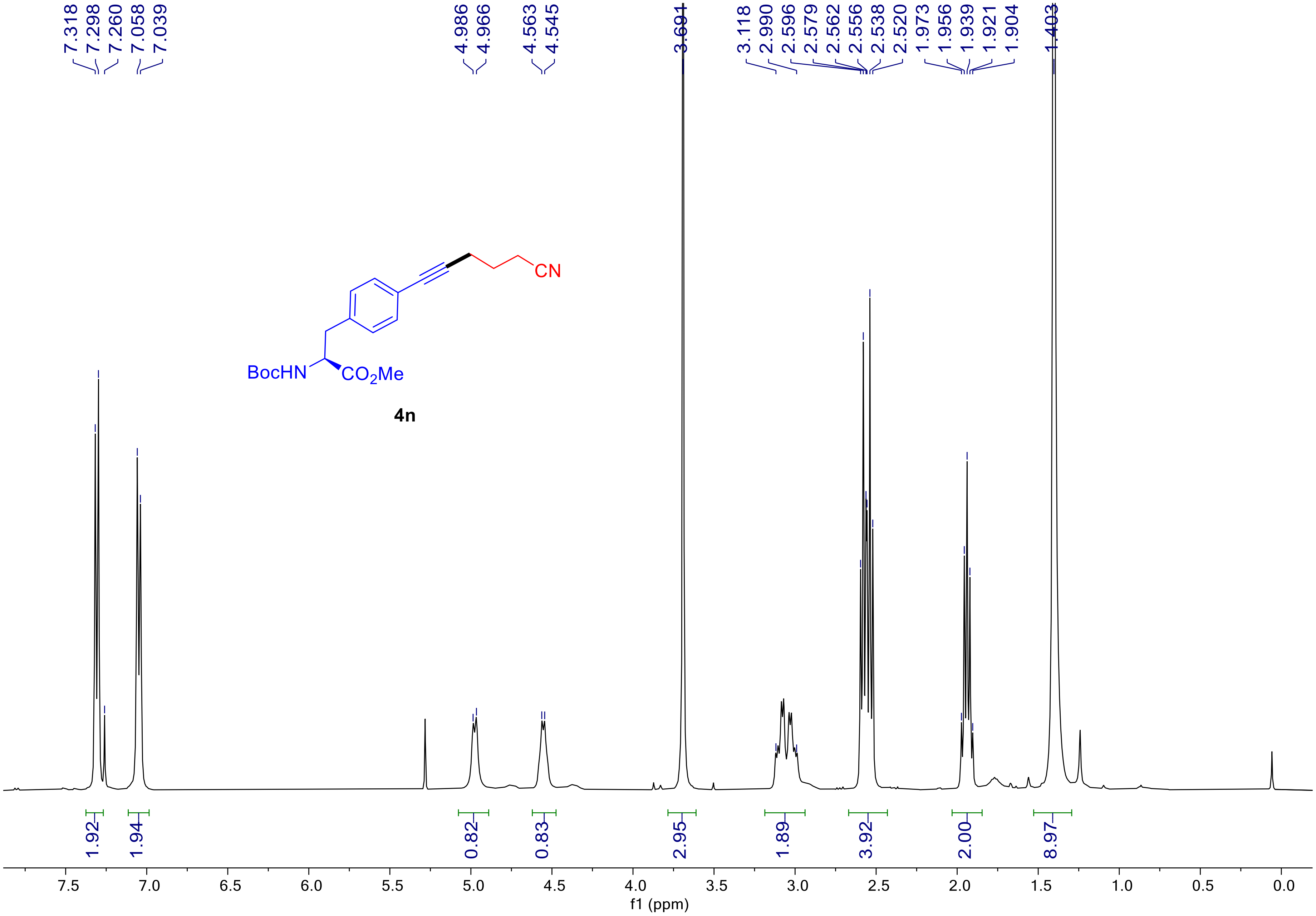

Supplementary Figure 83. ^{1}H NMR spectrum of 4n

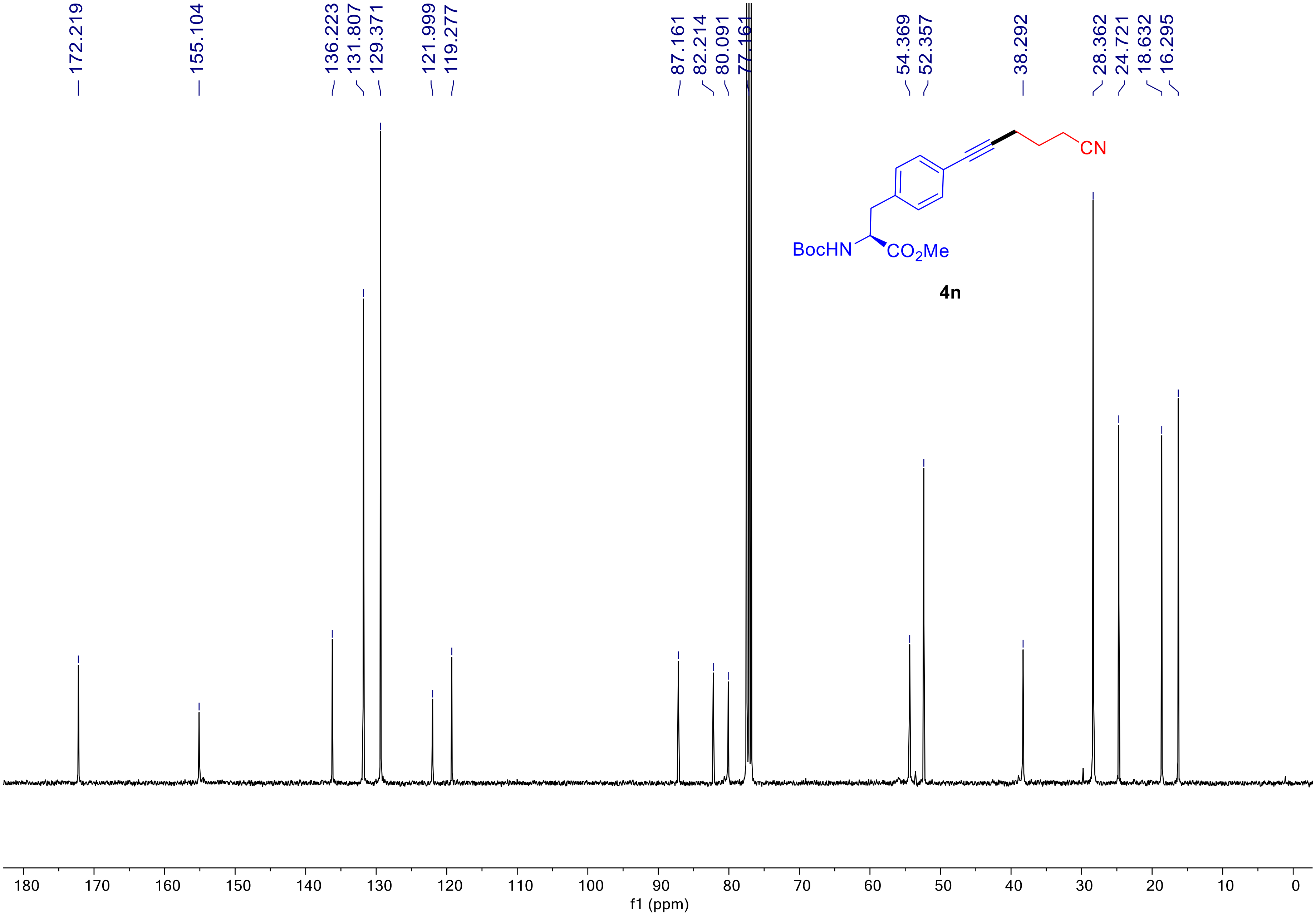

Supplementary Figure 84. ^{13}C NMR spectrum of 4n

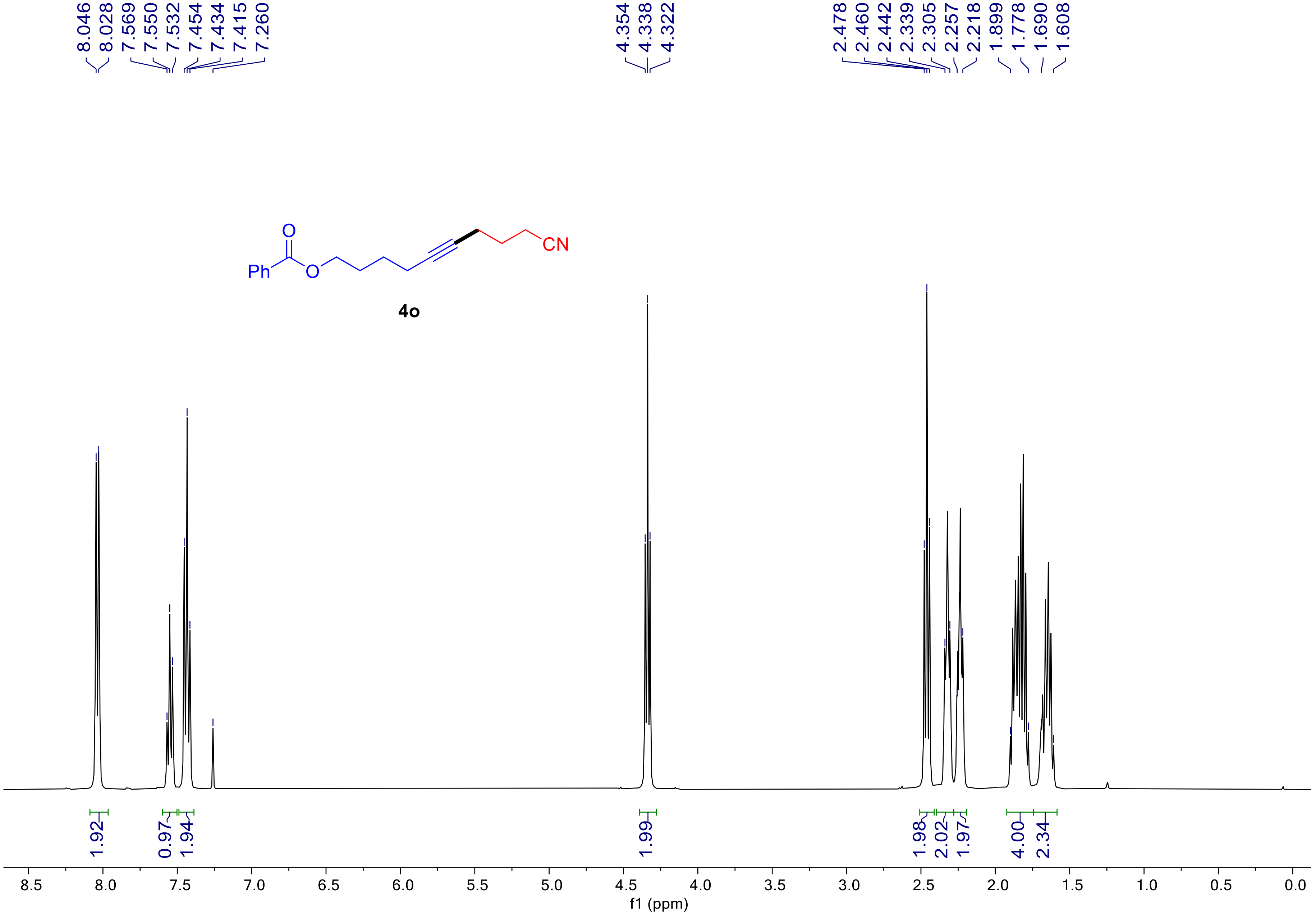

Supplementary Figure 85. ^{1}**H NMR spectrum of 4o**

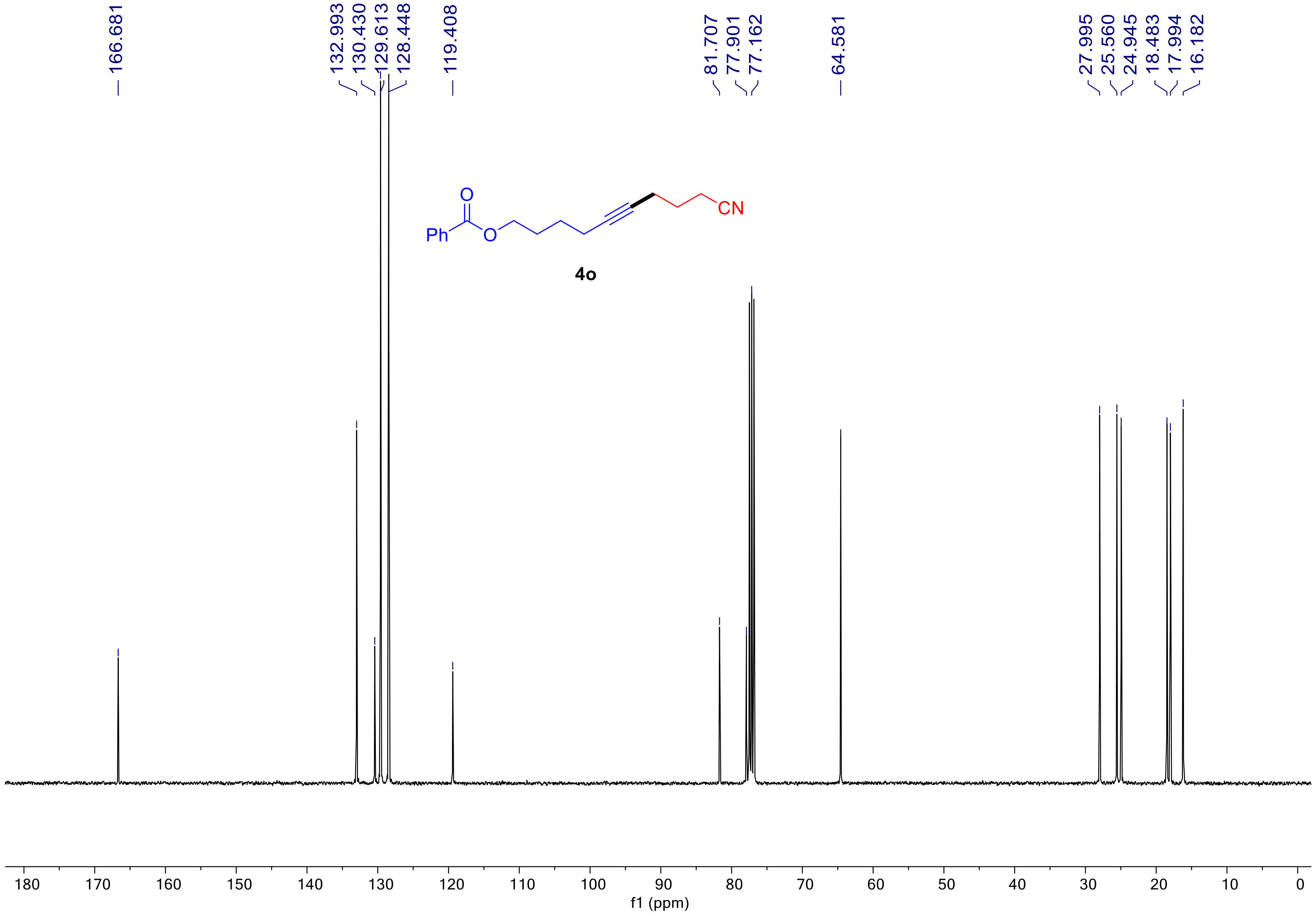

Supplementary Figure 86. ^{13}**C NMR spectrum of 4o**

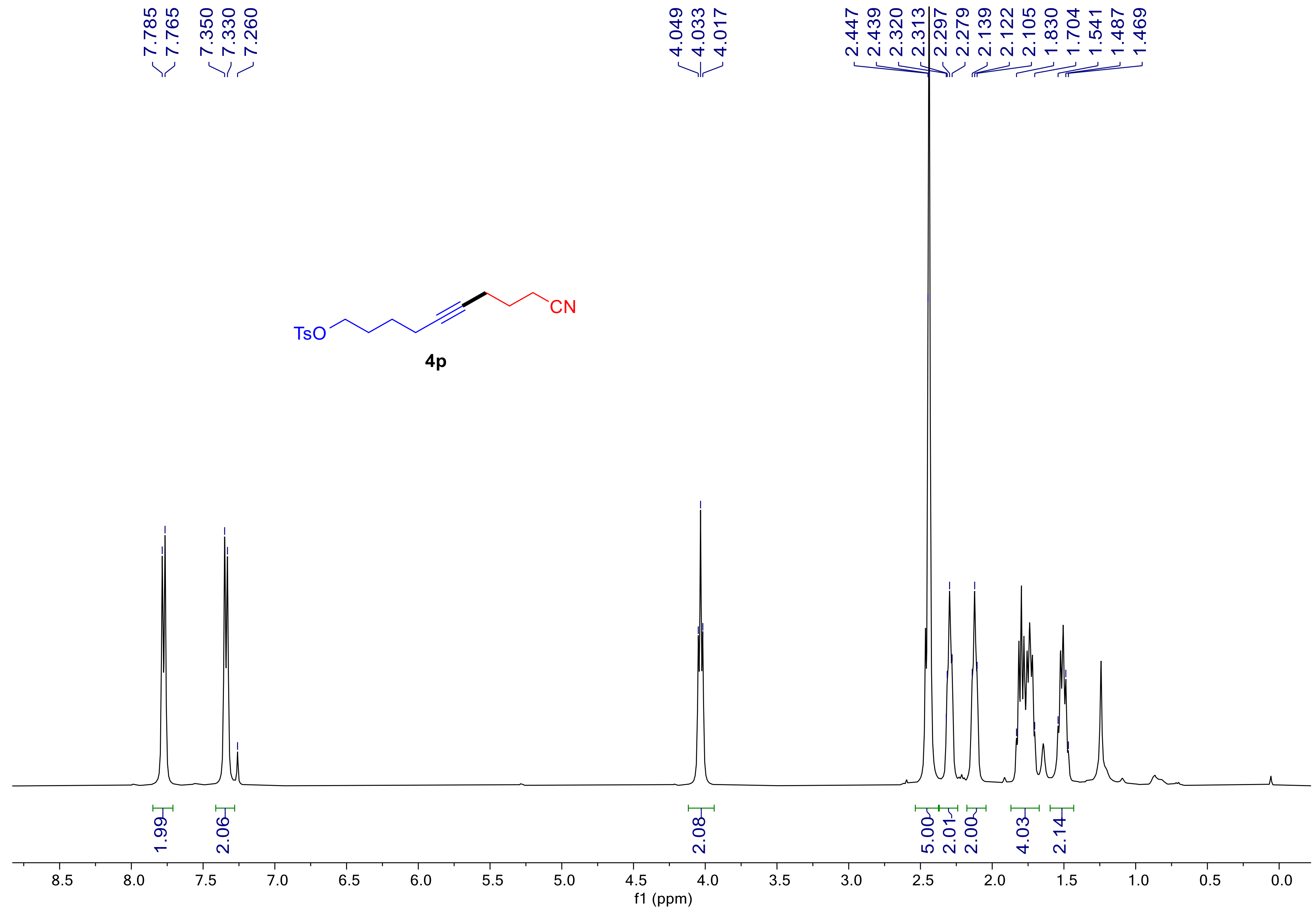

Supplementary Figure 87. ^{1}H NMR spectrum of 4p

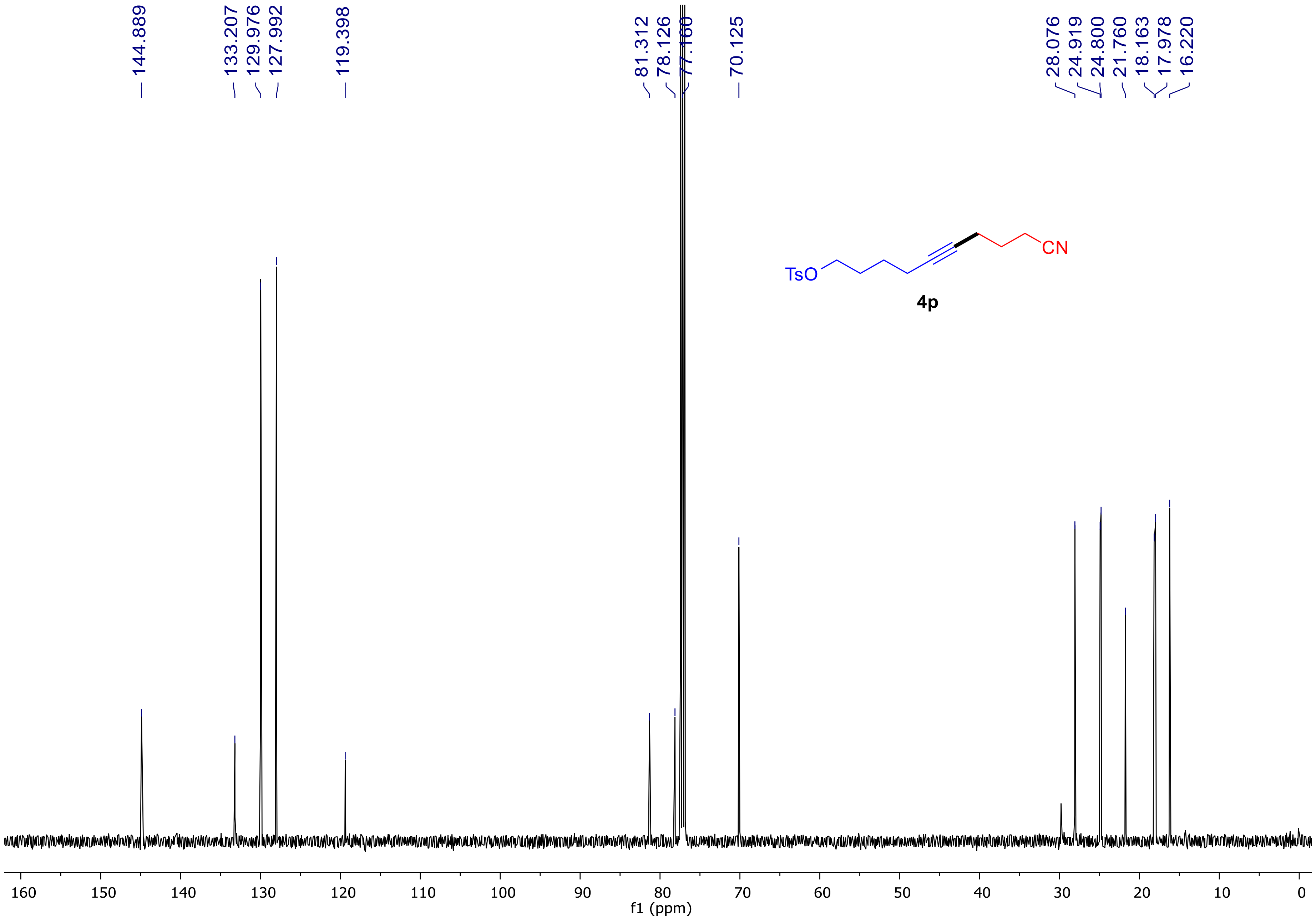

Supplementary Figure 88. ^{13}**C NMR spectrum of 4p**

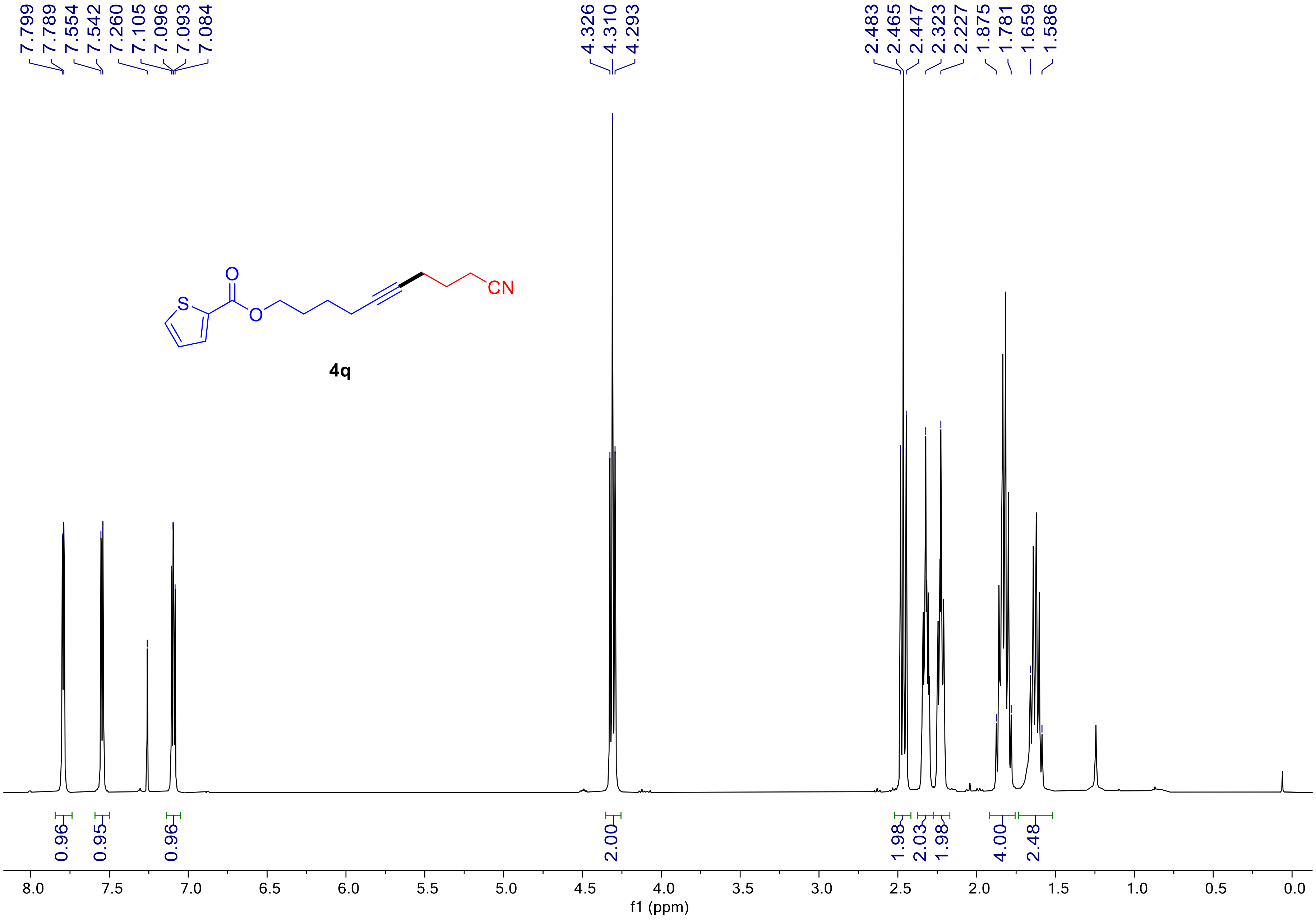

Supplementary Figure 89. ^{1}H NMR spectrum of 4q

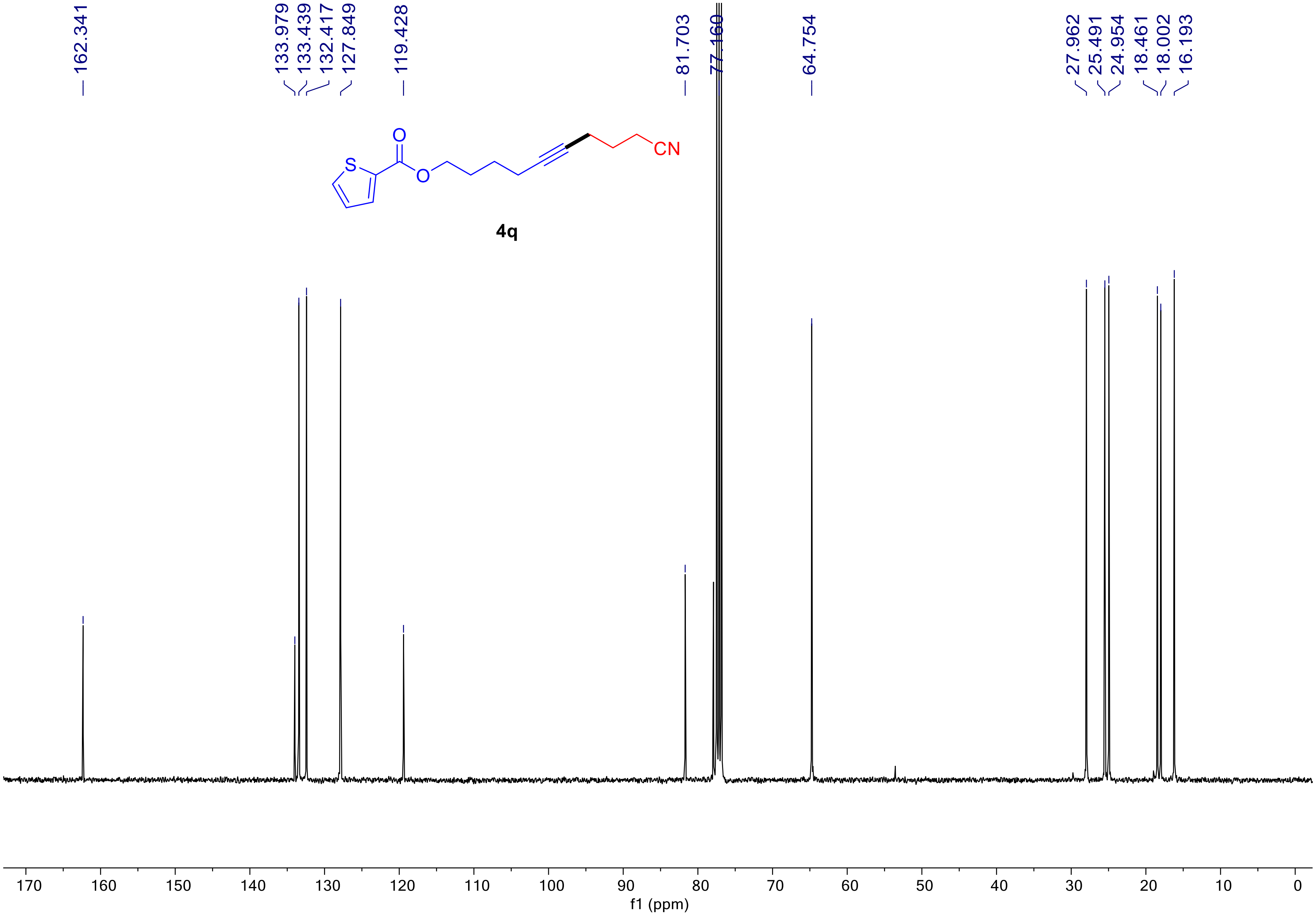

Supplementary Figure 90. ^{13}C NMR spectrum of 4q

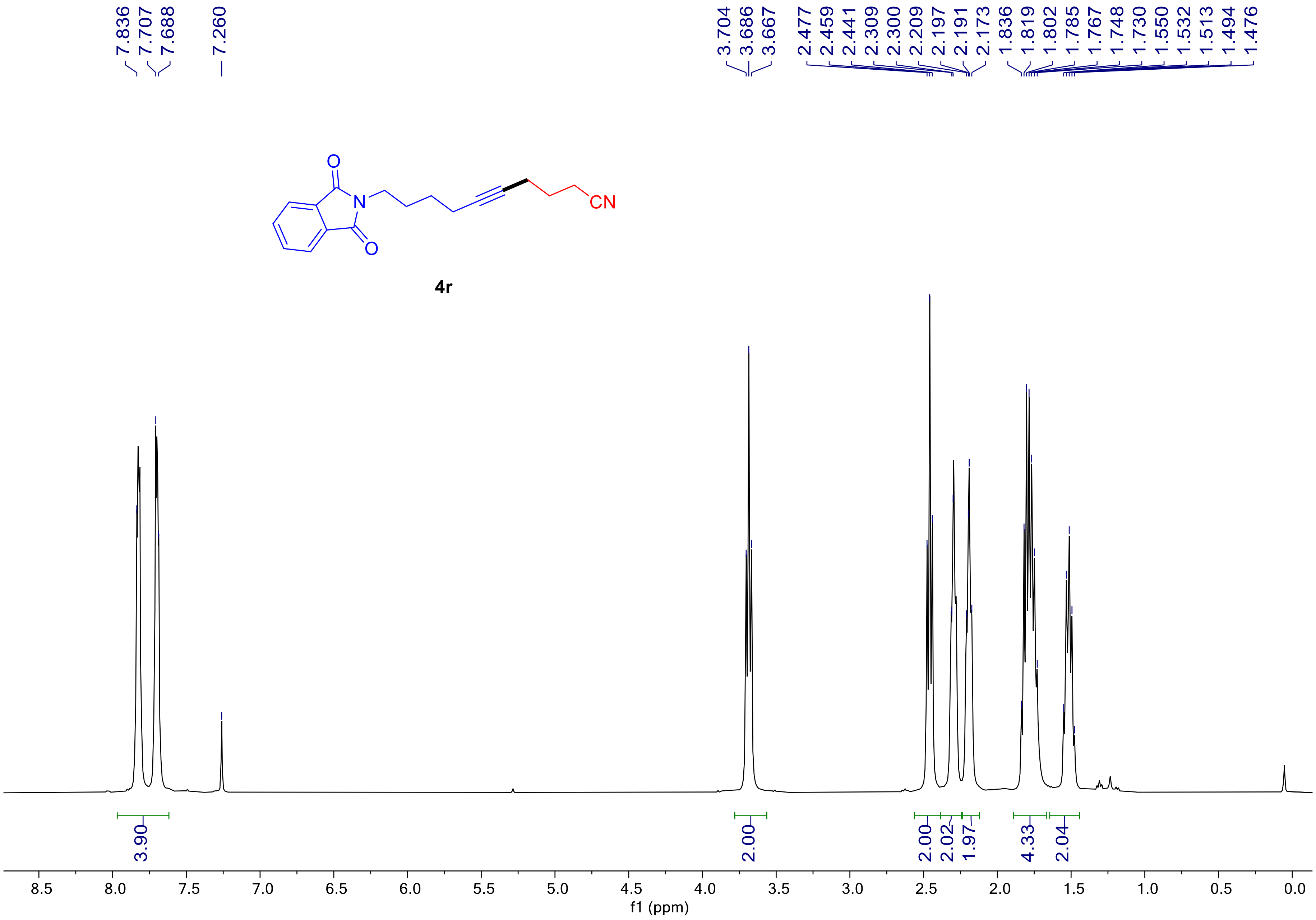

Supplementary Figure 91. ^{1}H NMR spectrum of 4r

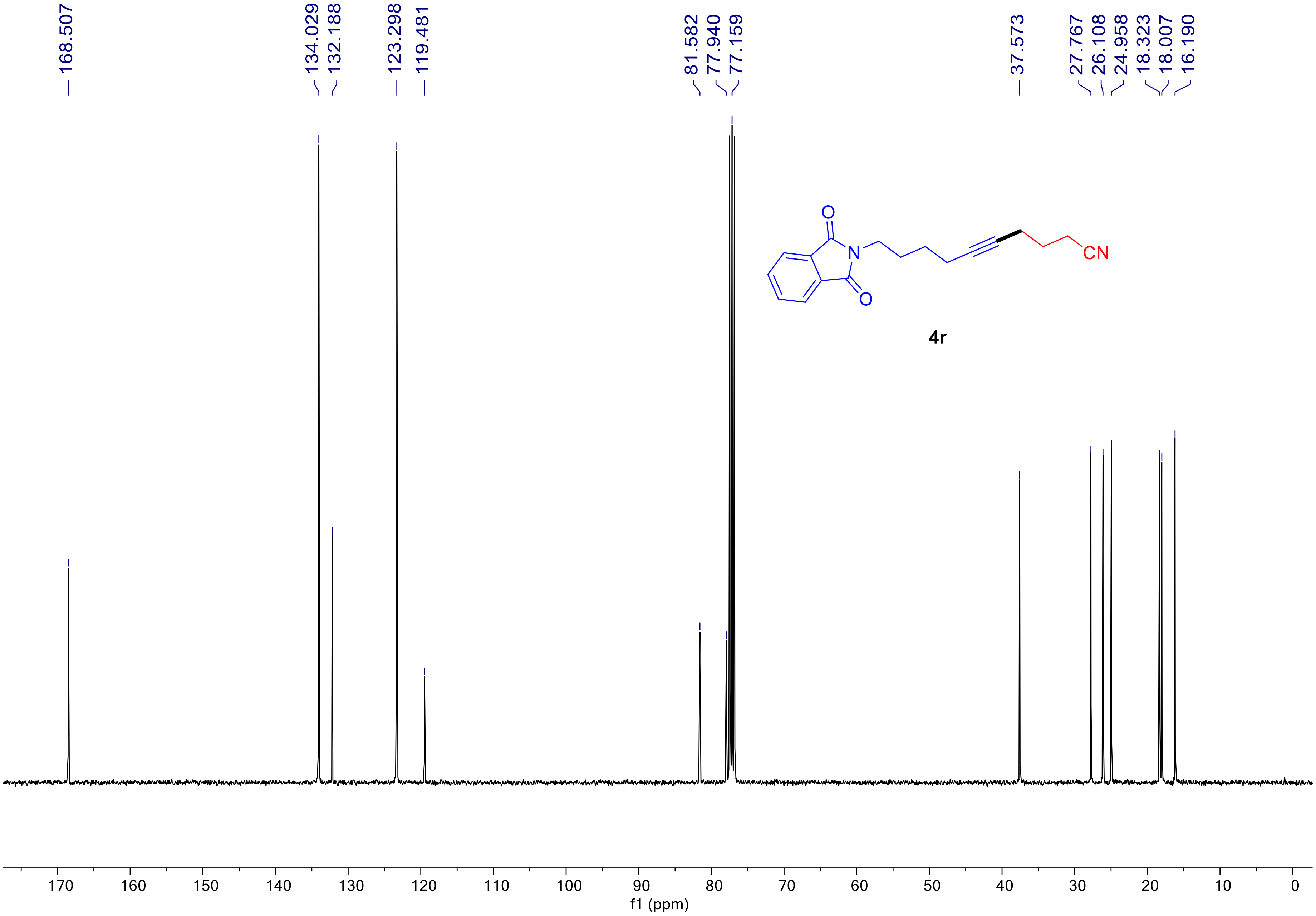

Supplementary Figure 92. ^{13}C NMR spectrum of 4r

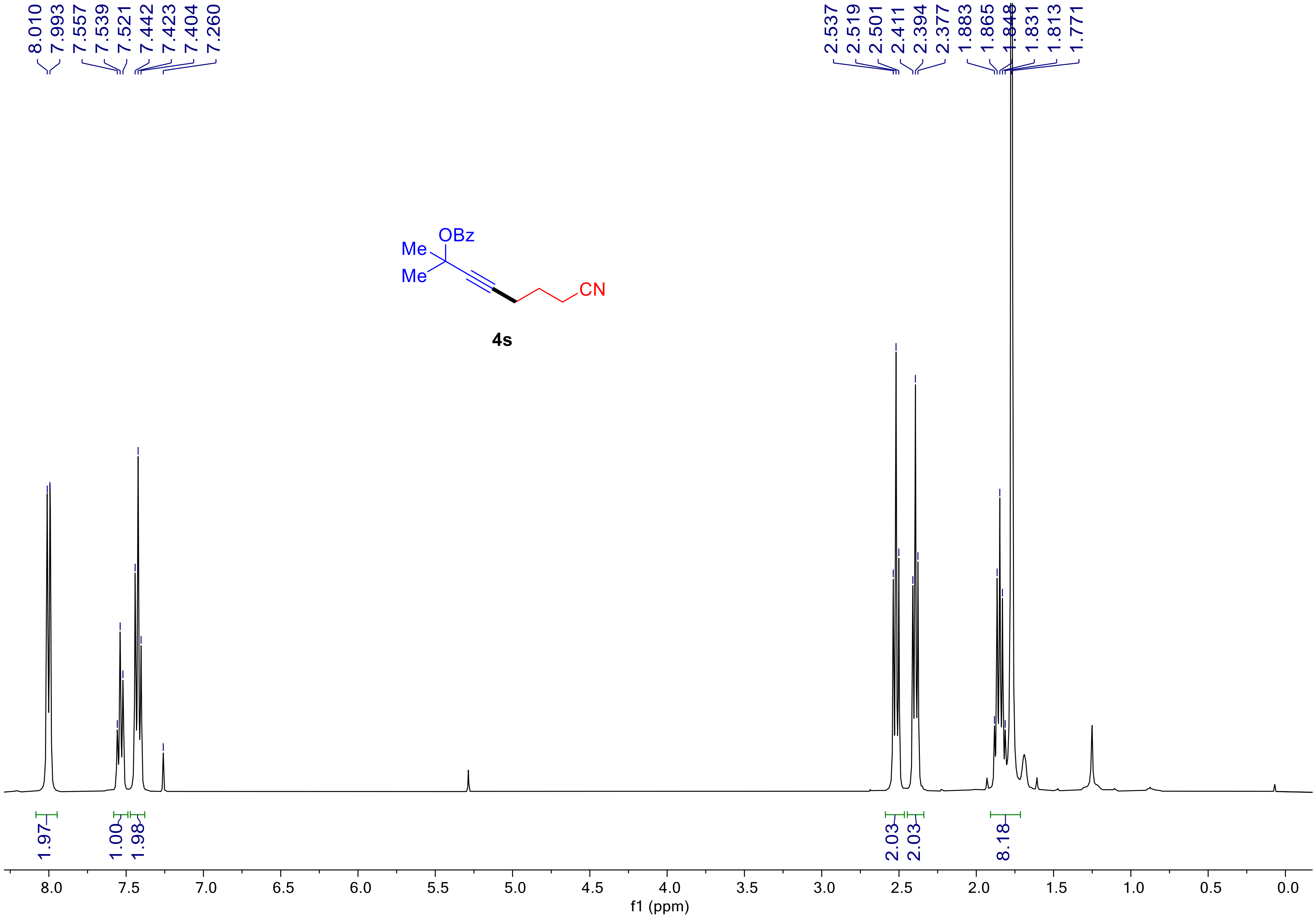

Supplementary Figure 93. ^{1}H NMR spectrum of 4s

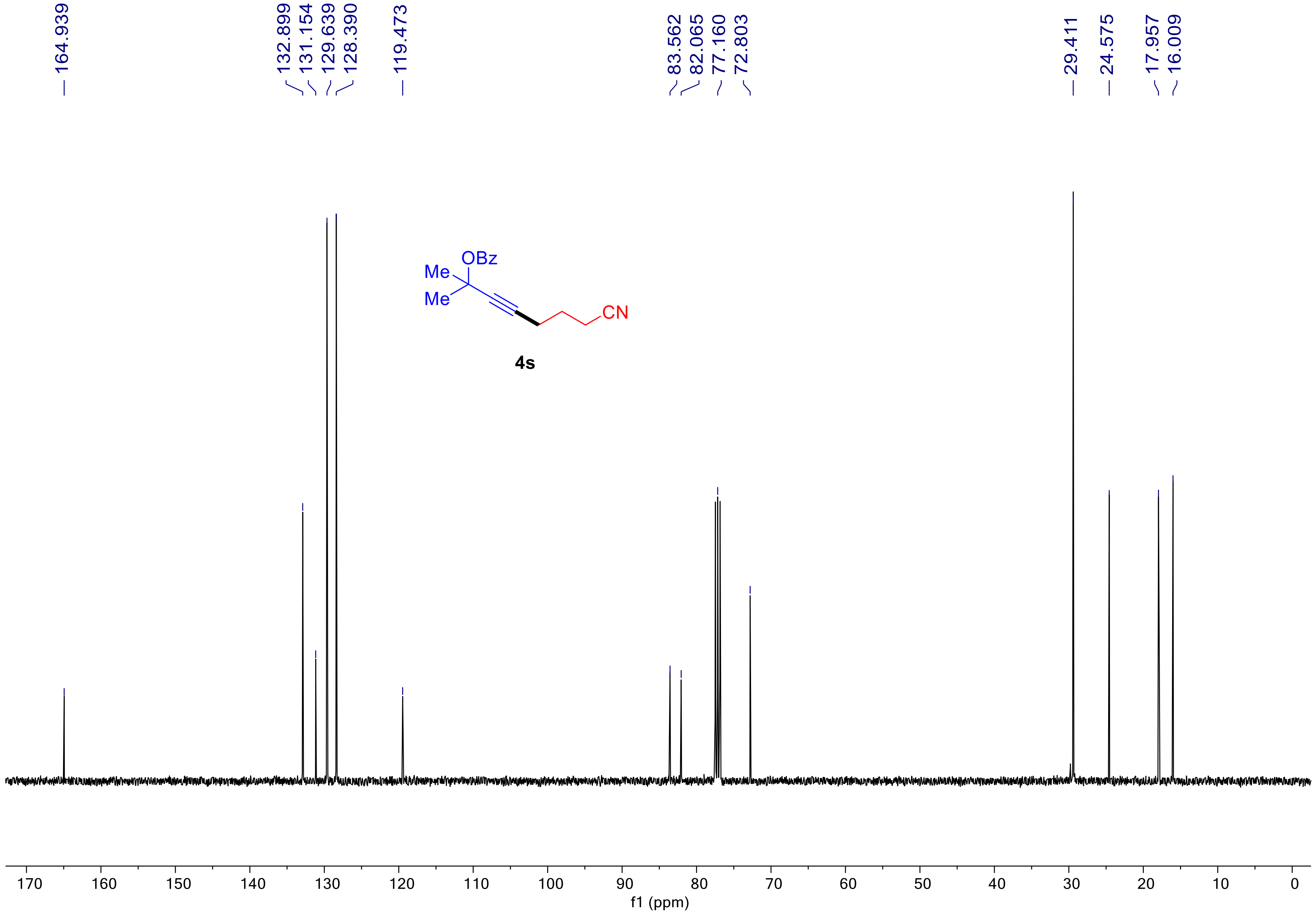

Supplementary Figure 94. ^{13}C NMR spectrum of 4s

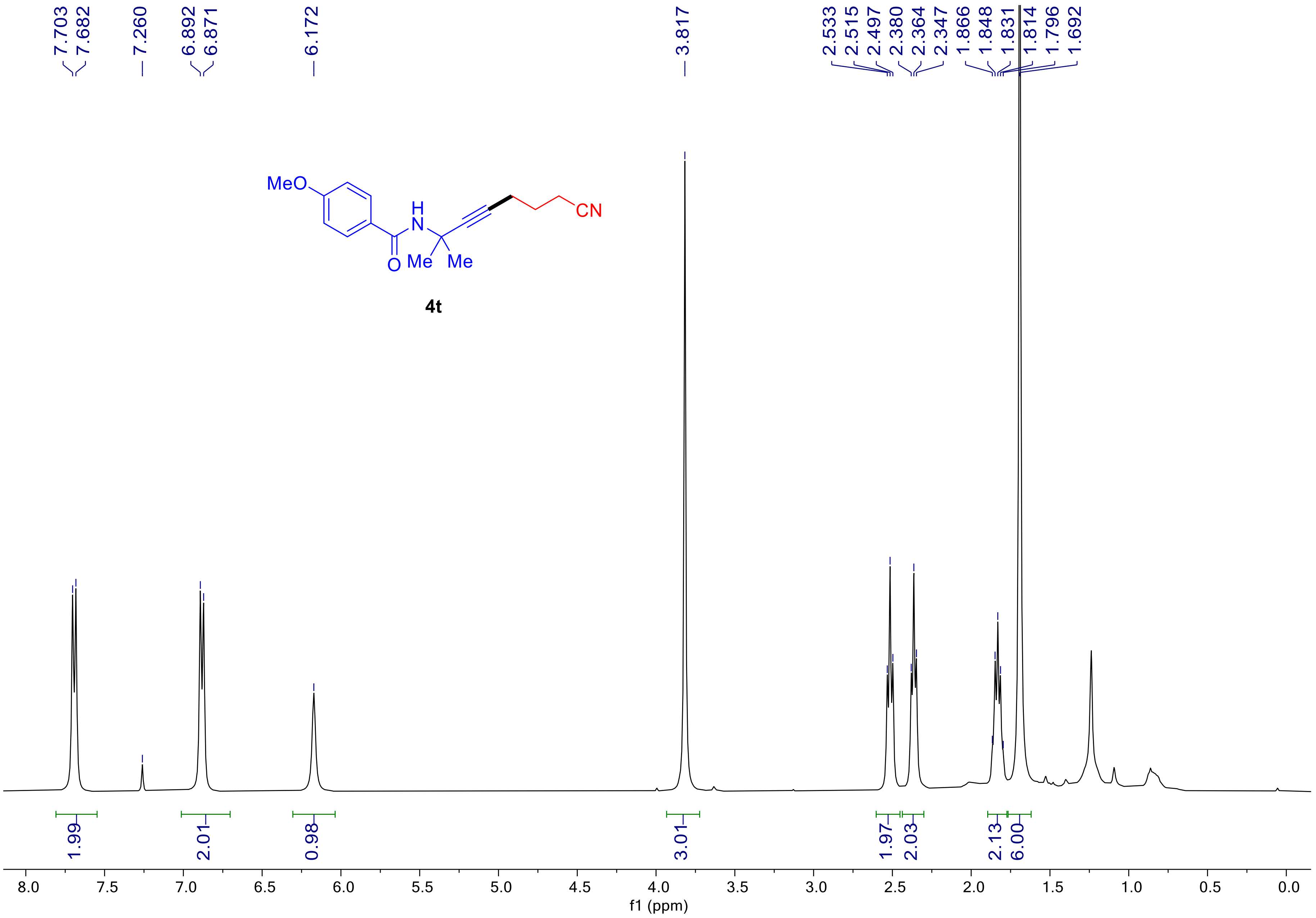

Supplementary Figure 95. ^{1}H NMR spectrum of 4t

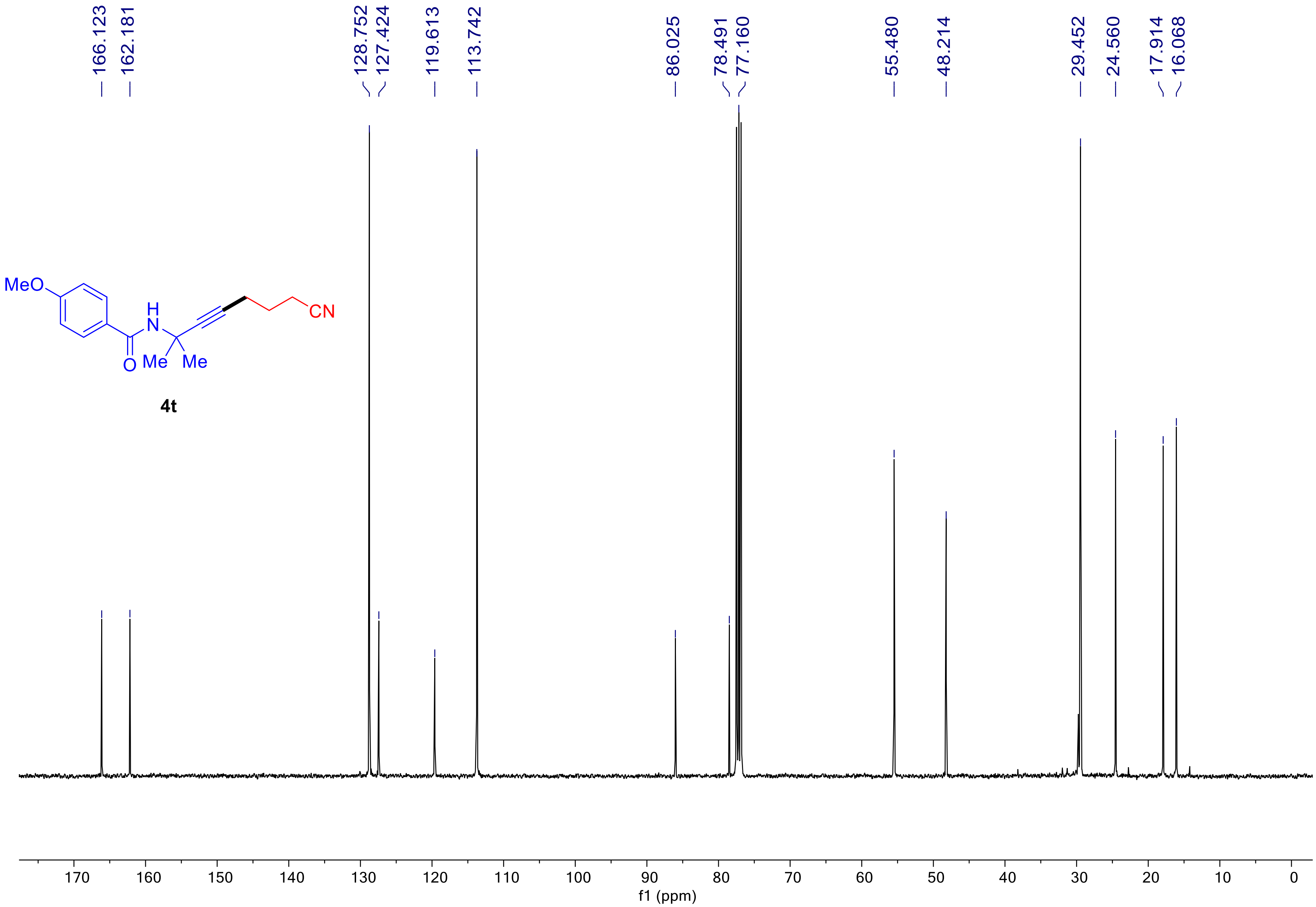

Supplementary Figure 96. ^{13}**C NMR spectrum of 4t**

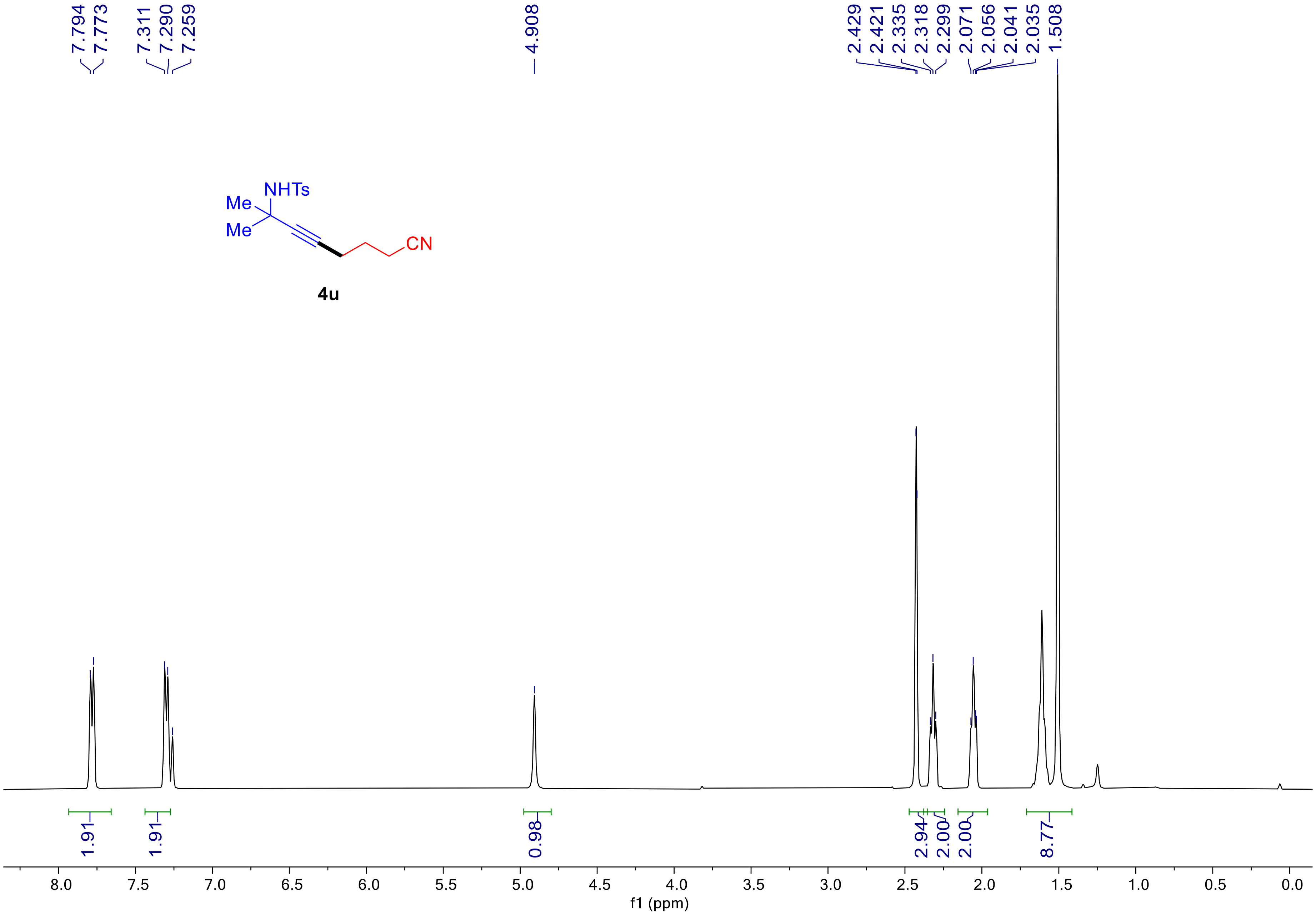

Supplementary Figure 97. ^{1}H NMR spectrum of 4u

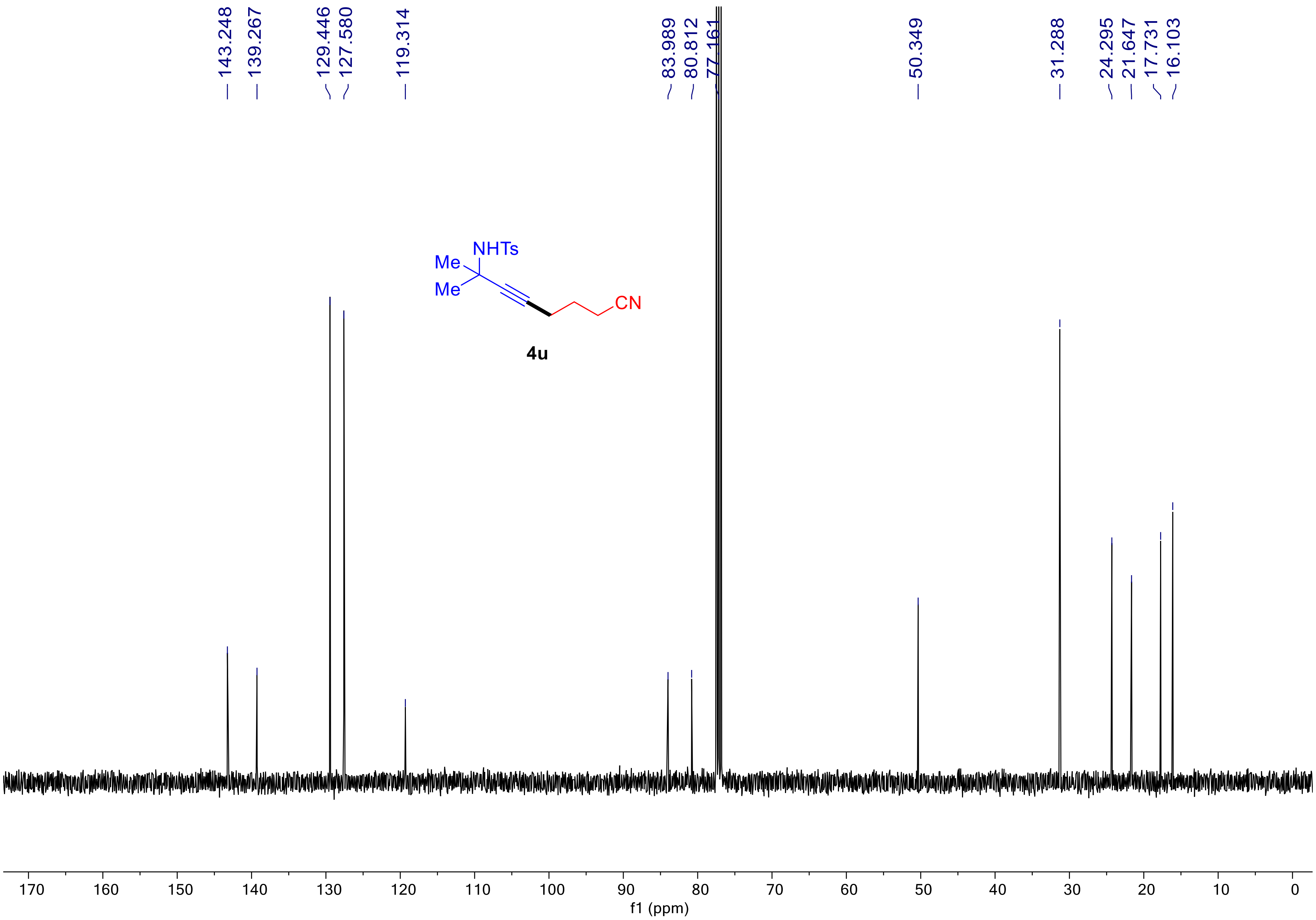

Supplementary Figure 98. ^{13}C NMR spectrum of 4u

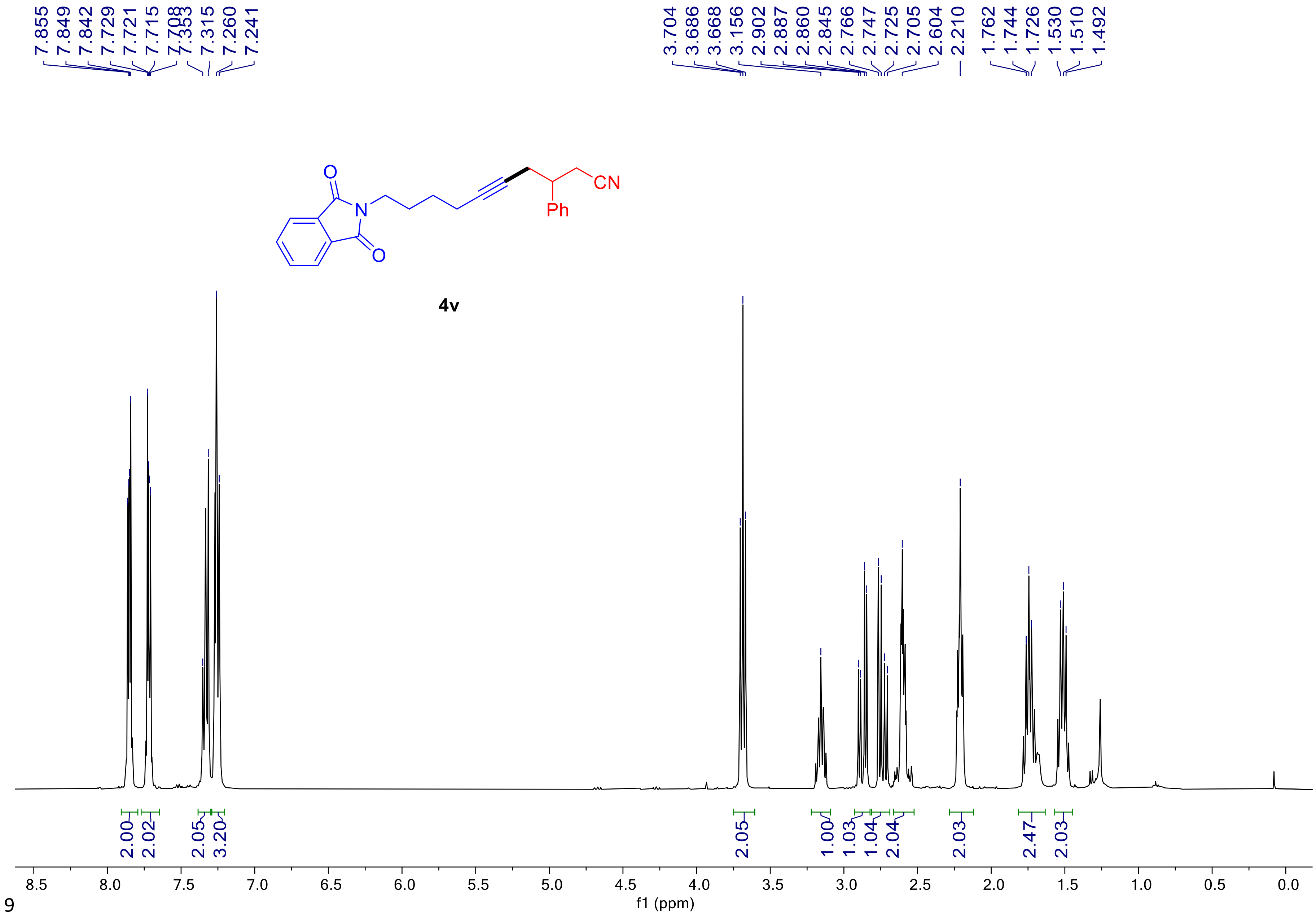

Supplementary Figure 99. ^{1}H NMR spectrum of 4v

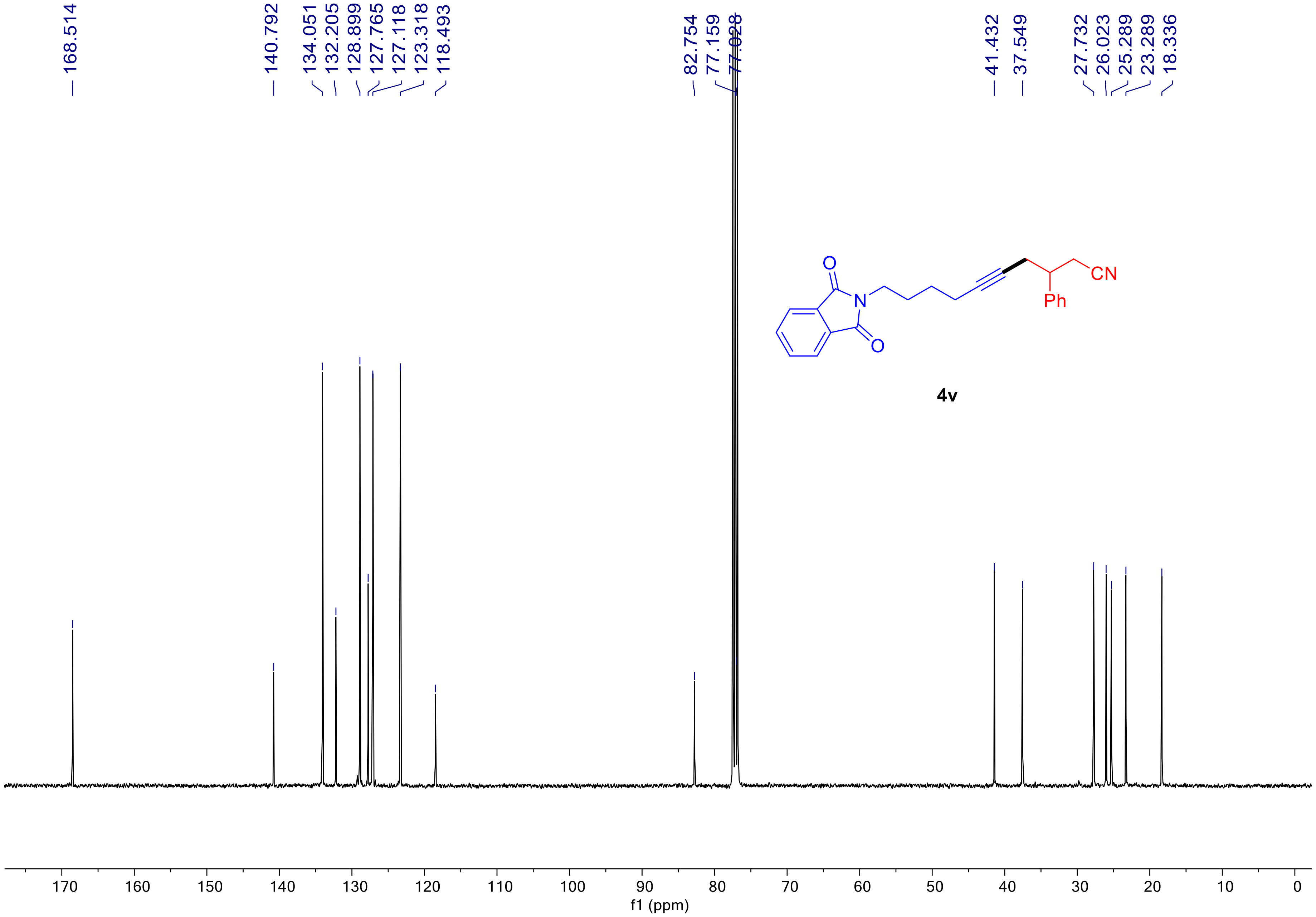

Supplementary Figure 100. ^{13}C NMR spectrum of 4v

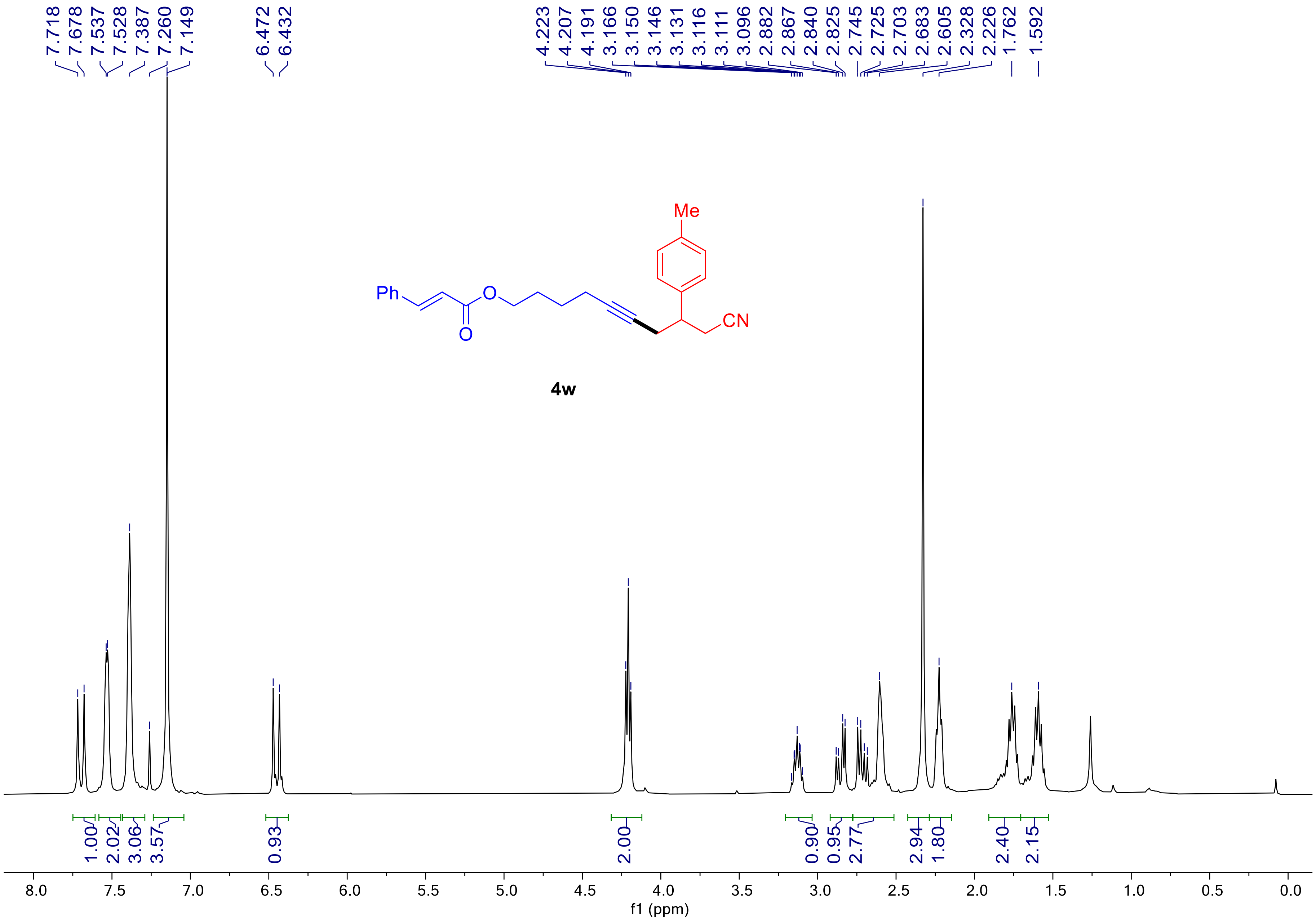

Supplementary Figure 101. ^{1}H NMR spectrum of 4w

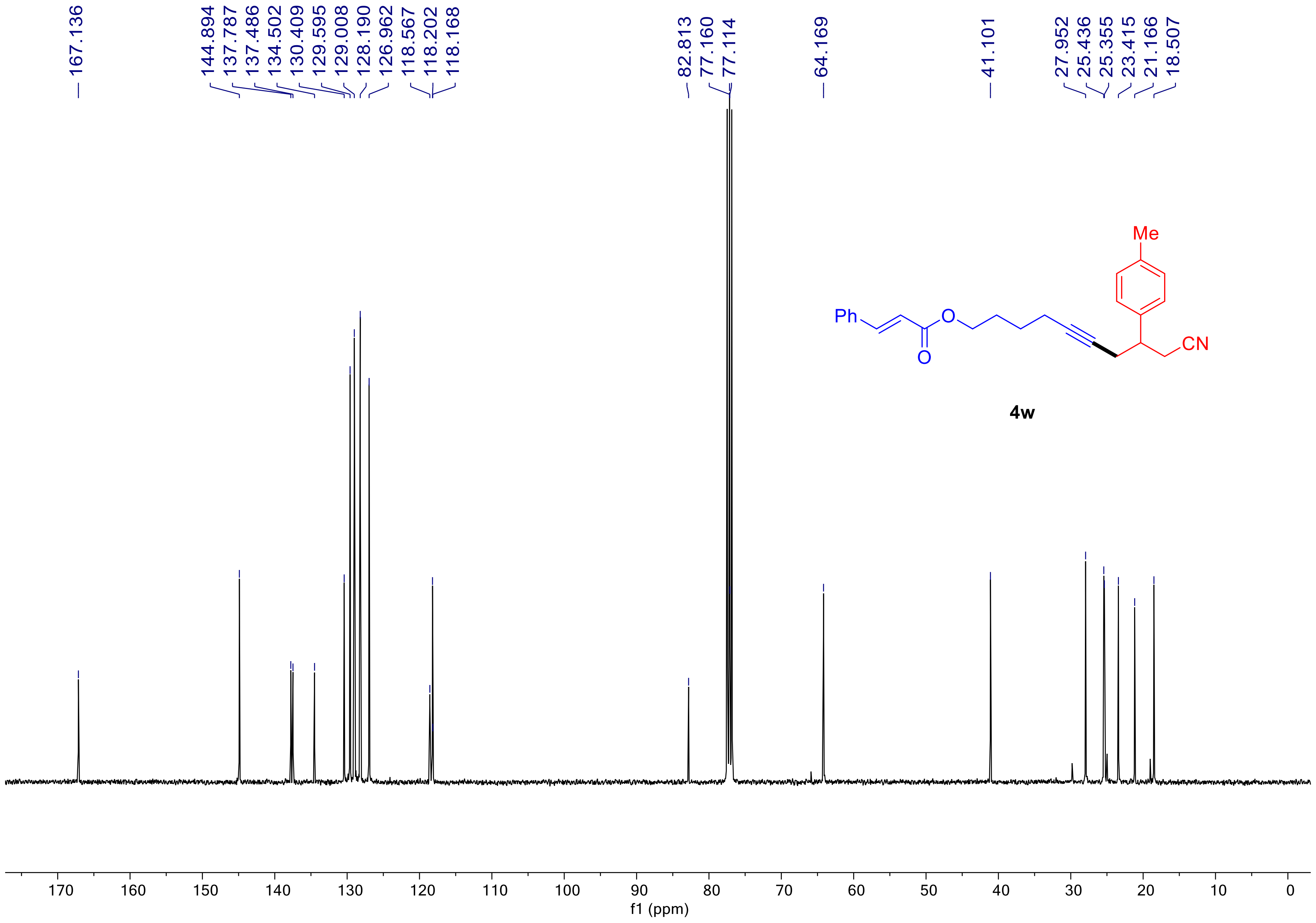

Supplementary Figure 102. ^{13}C NMR spectrum of 4w

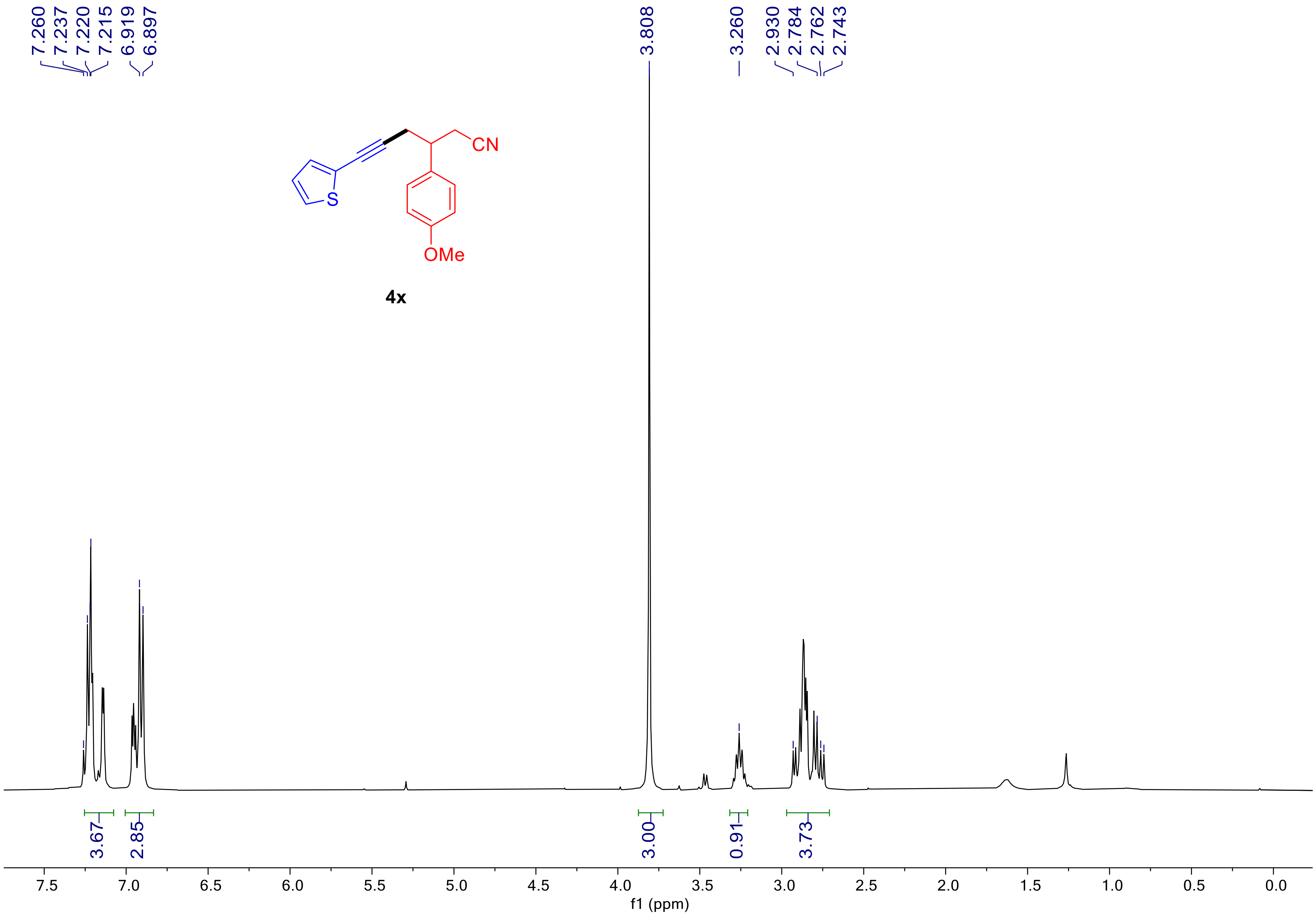

Supplementary Figure 103. ^{1}H NMR spectrum of 4x

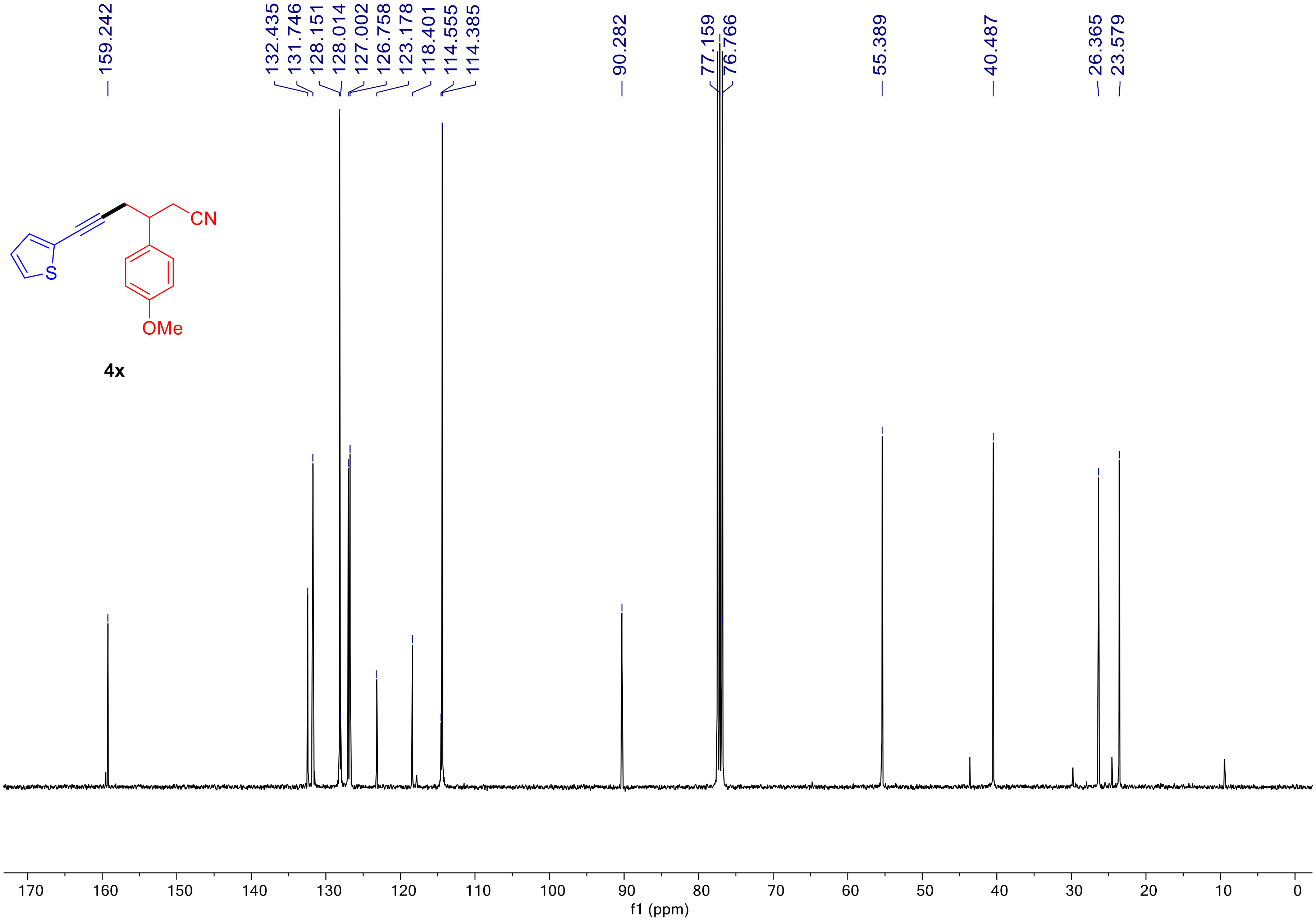

Supplementary Figure 104. ^{13}**C NMR spectrum of 4x**

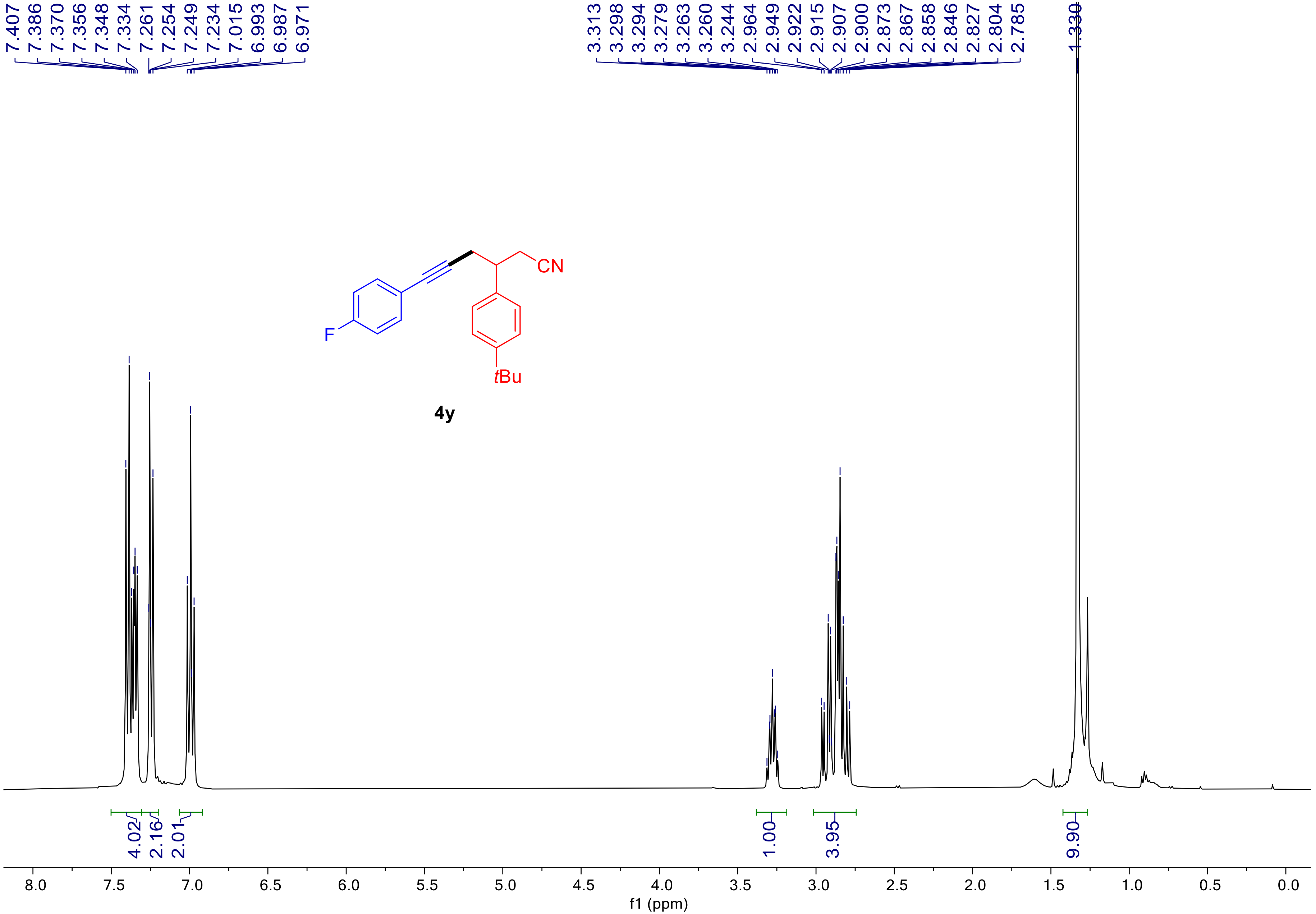

Supplementary Figure 105. ^{1}H NMR spectrum of 4y

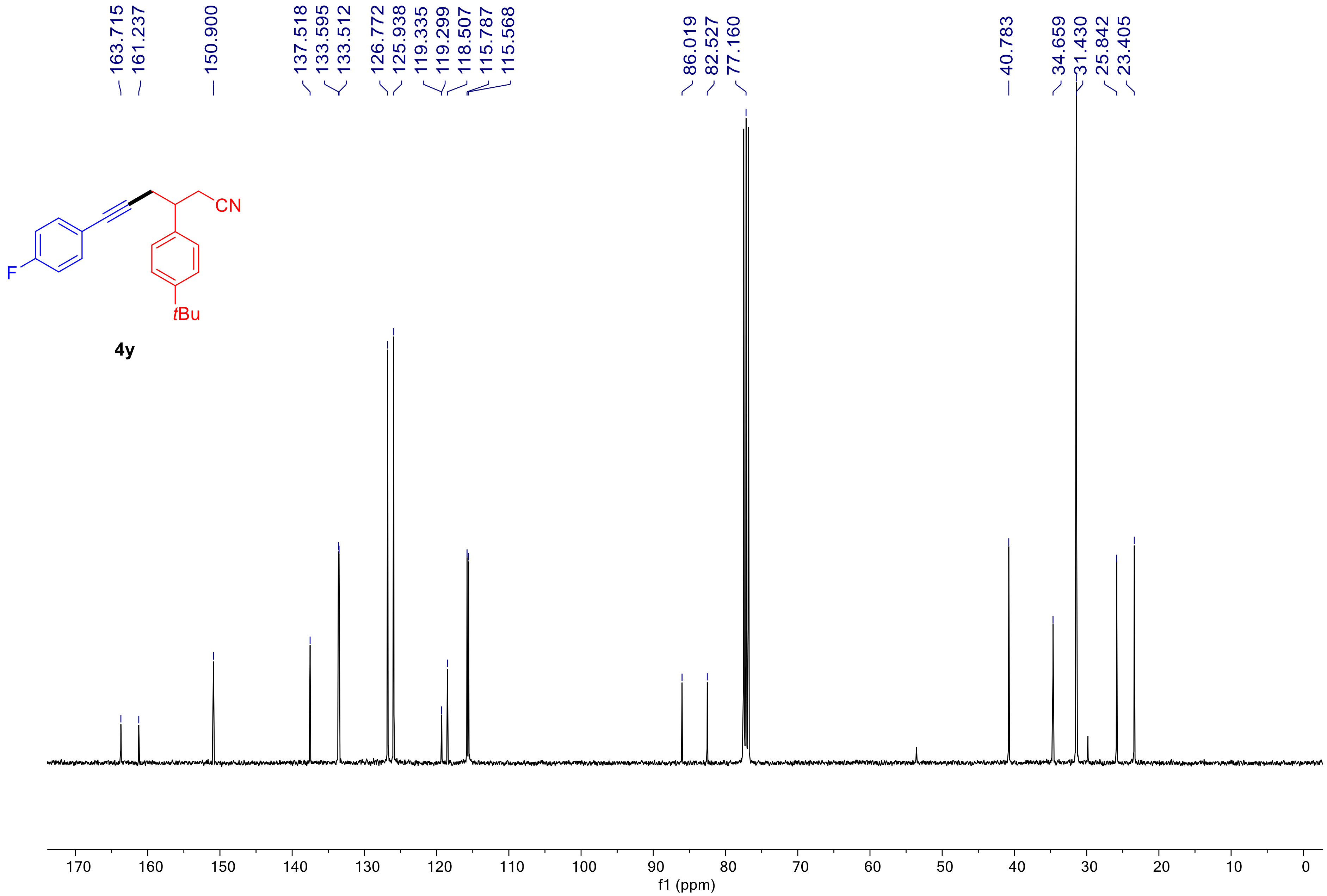

Supplementary Figure 106. ^{13}C NMR spectrum of 4y

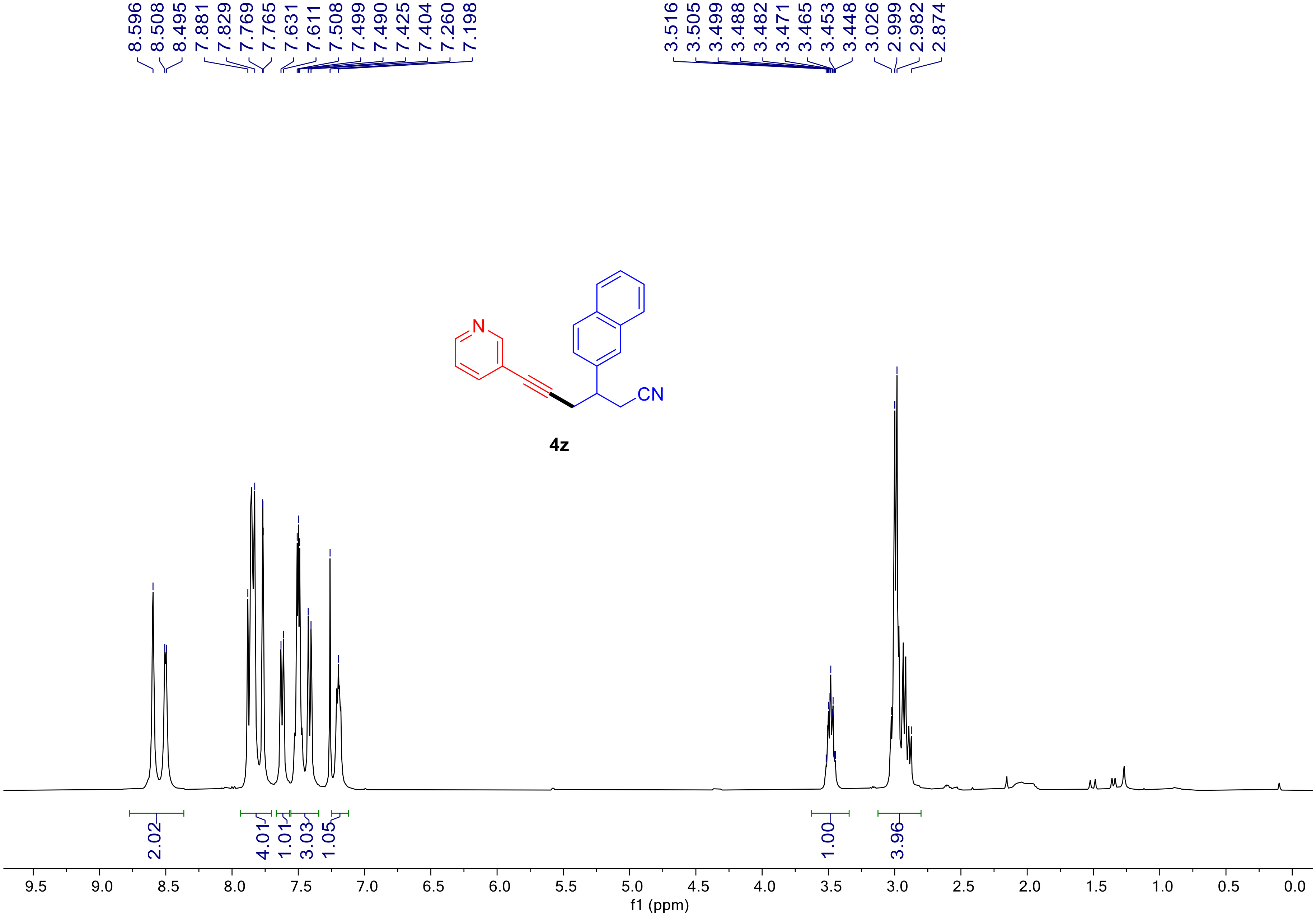

Supplementary Figure 107. ^{1}**H NMR spectrum of 4z**

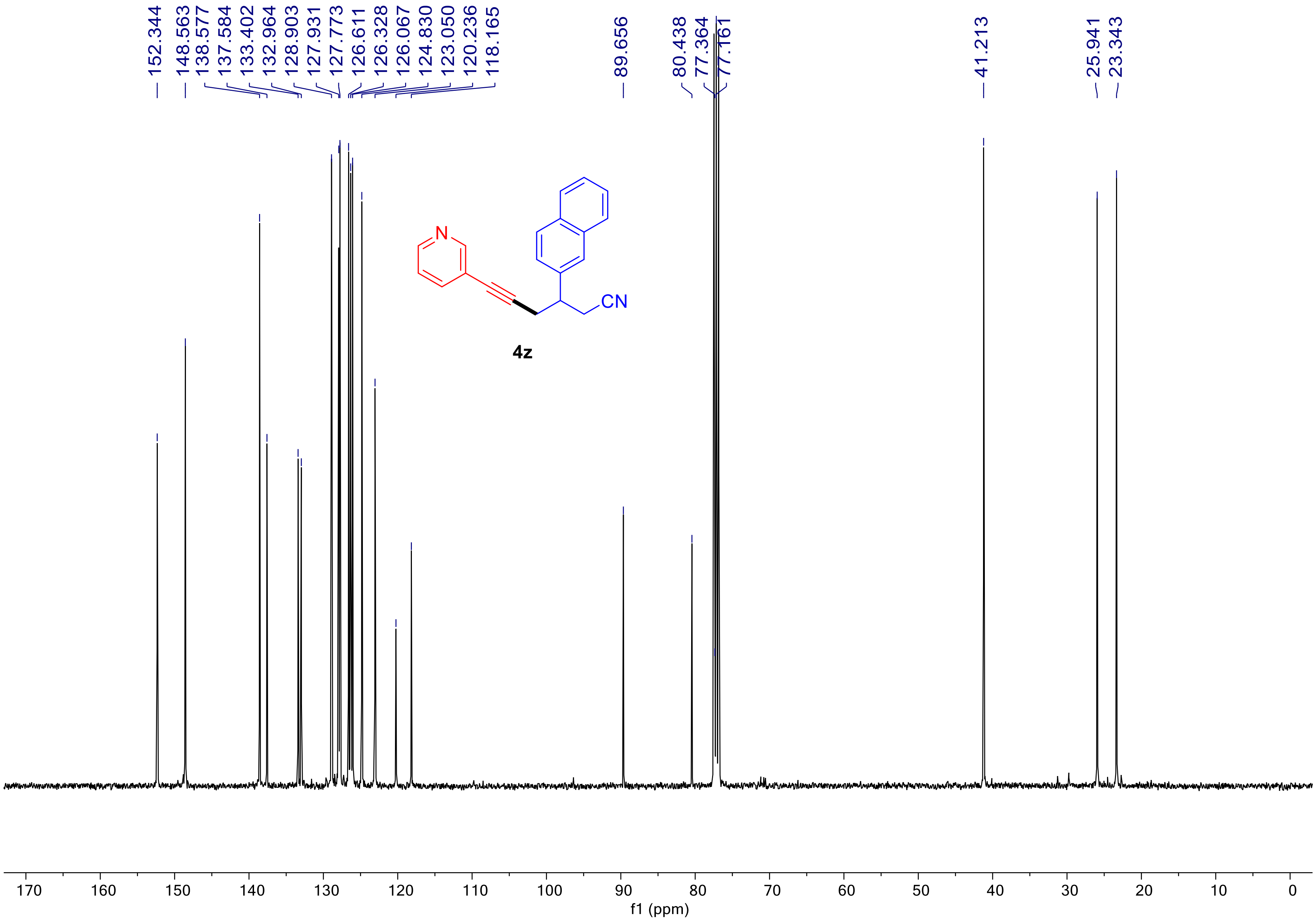

Supplementary Figure 108. ^{13}C NMR spectrum of 4z

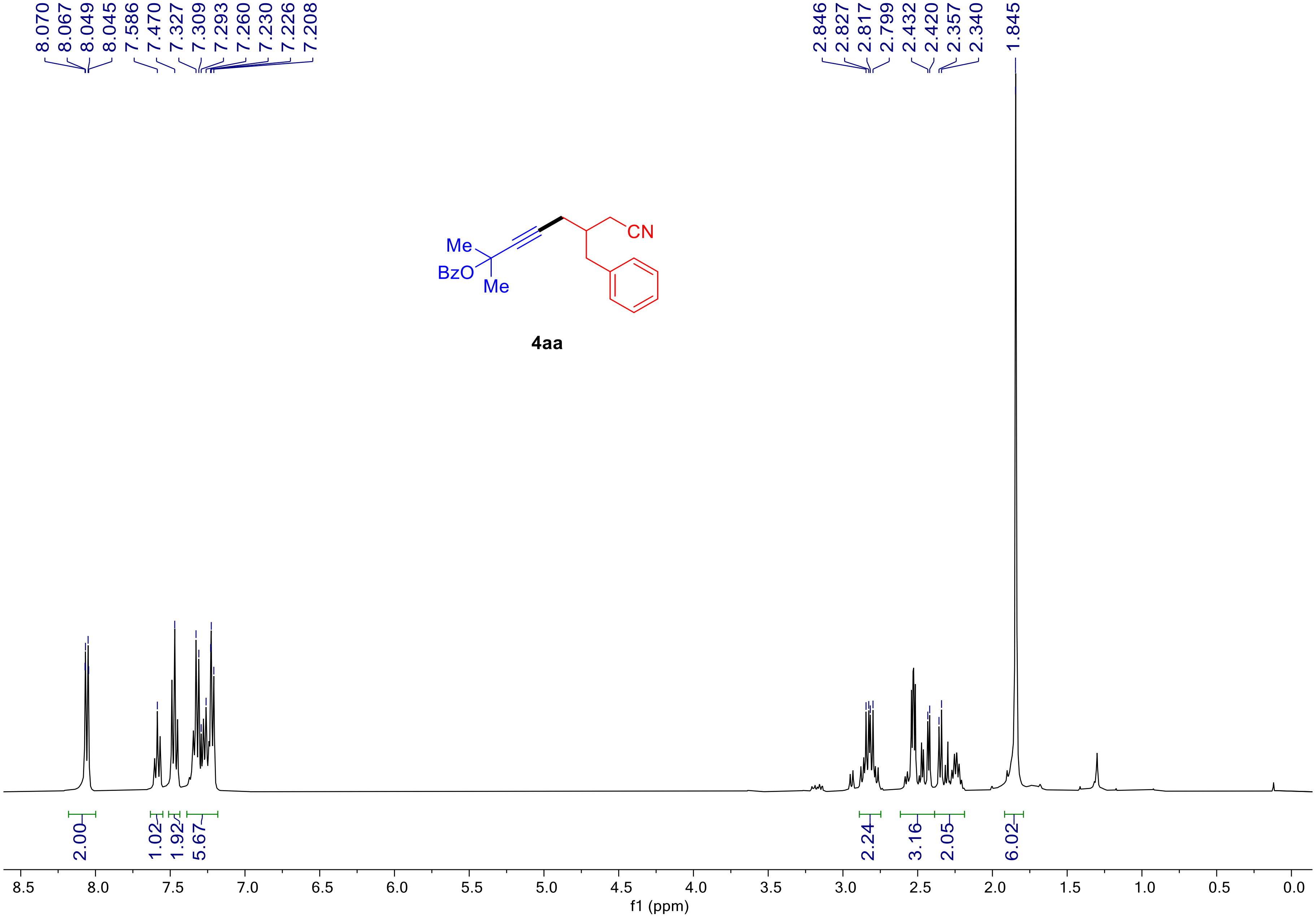

Supplementary Figure 109. ^{1}H NMR spectrum of 4aa

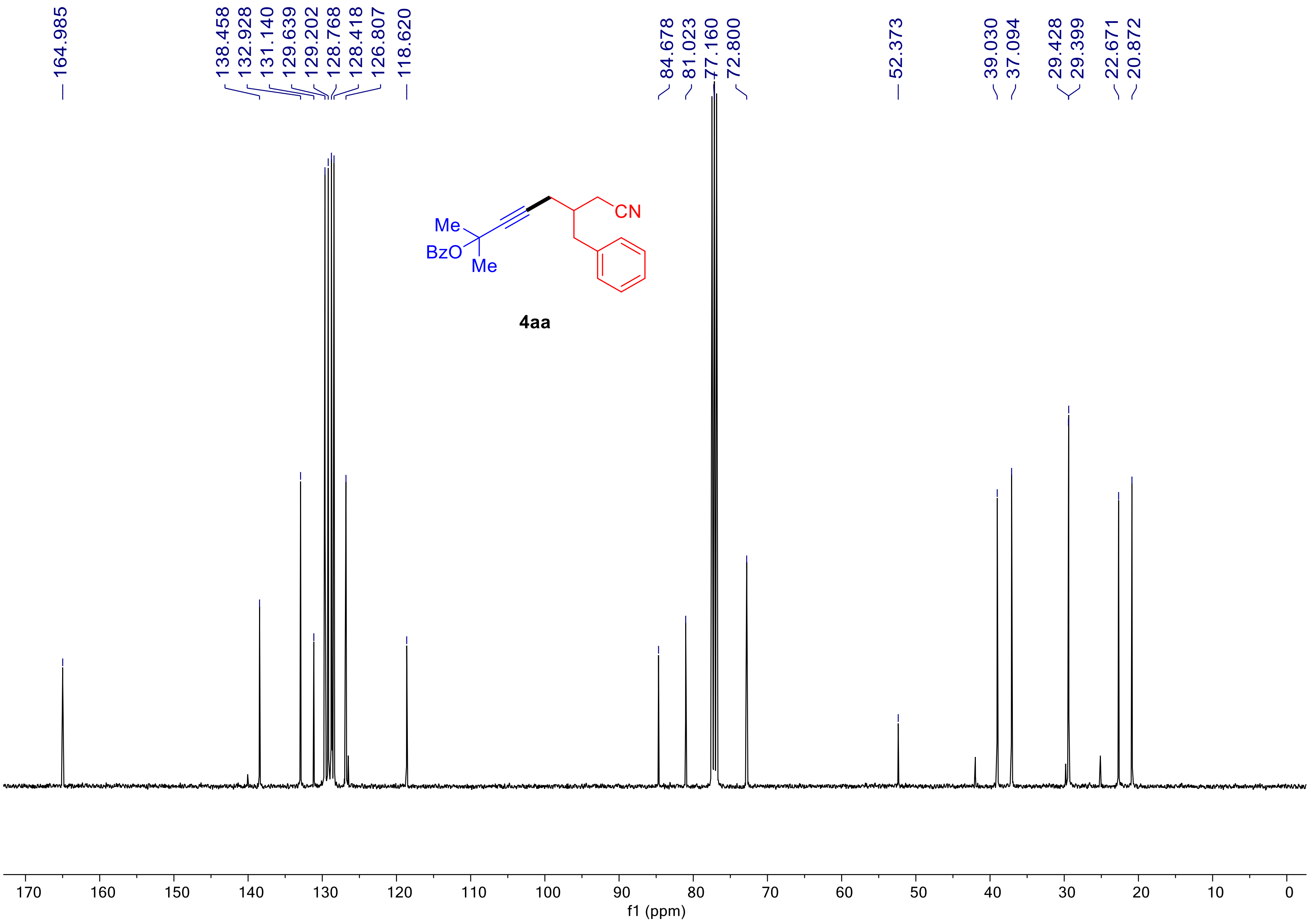

Supplementary Figure 110. ^{13}**C NMR spectrum of 4aa**

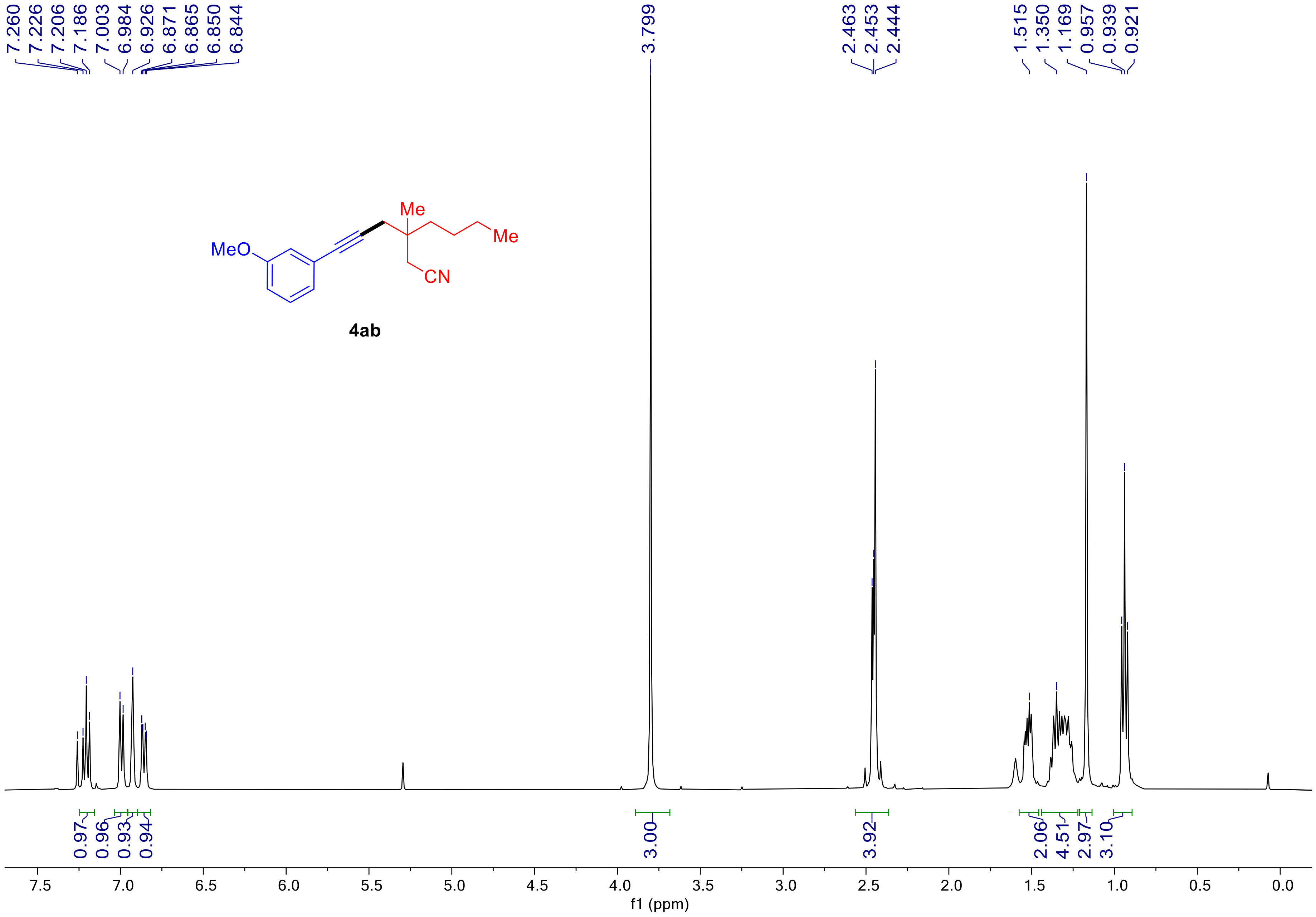

Supplementary Figure 111. ^{1}H NMR spectrum of 4ab

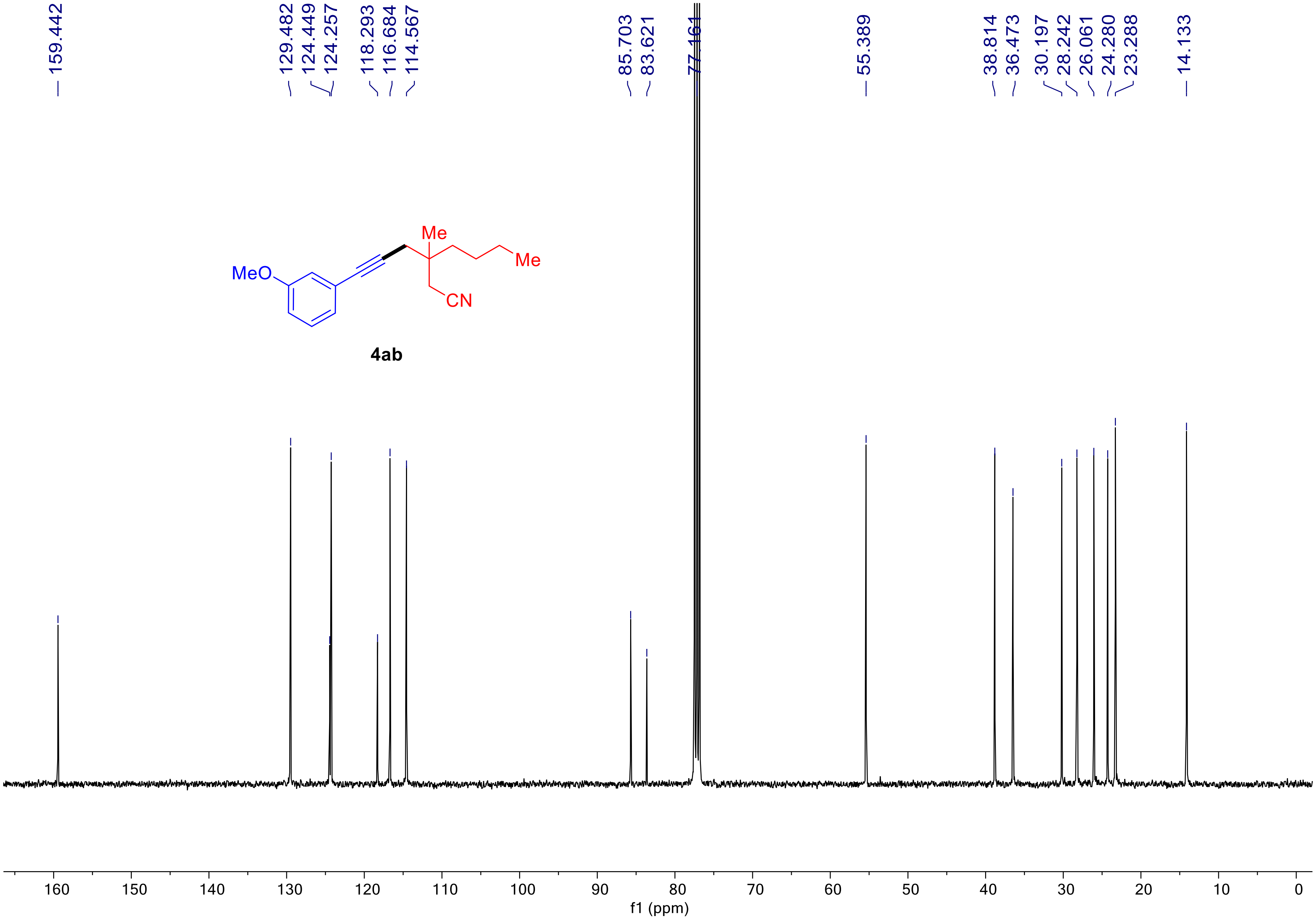

Supplementary Figure 112. ^{13}C NMR spectrum of 4ab

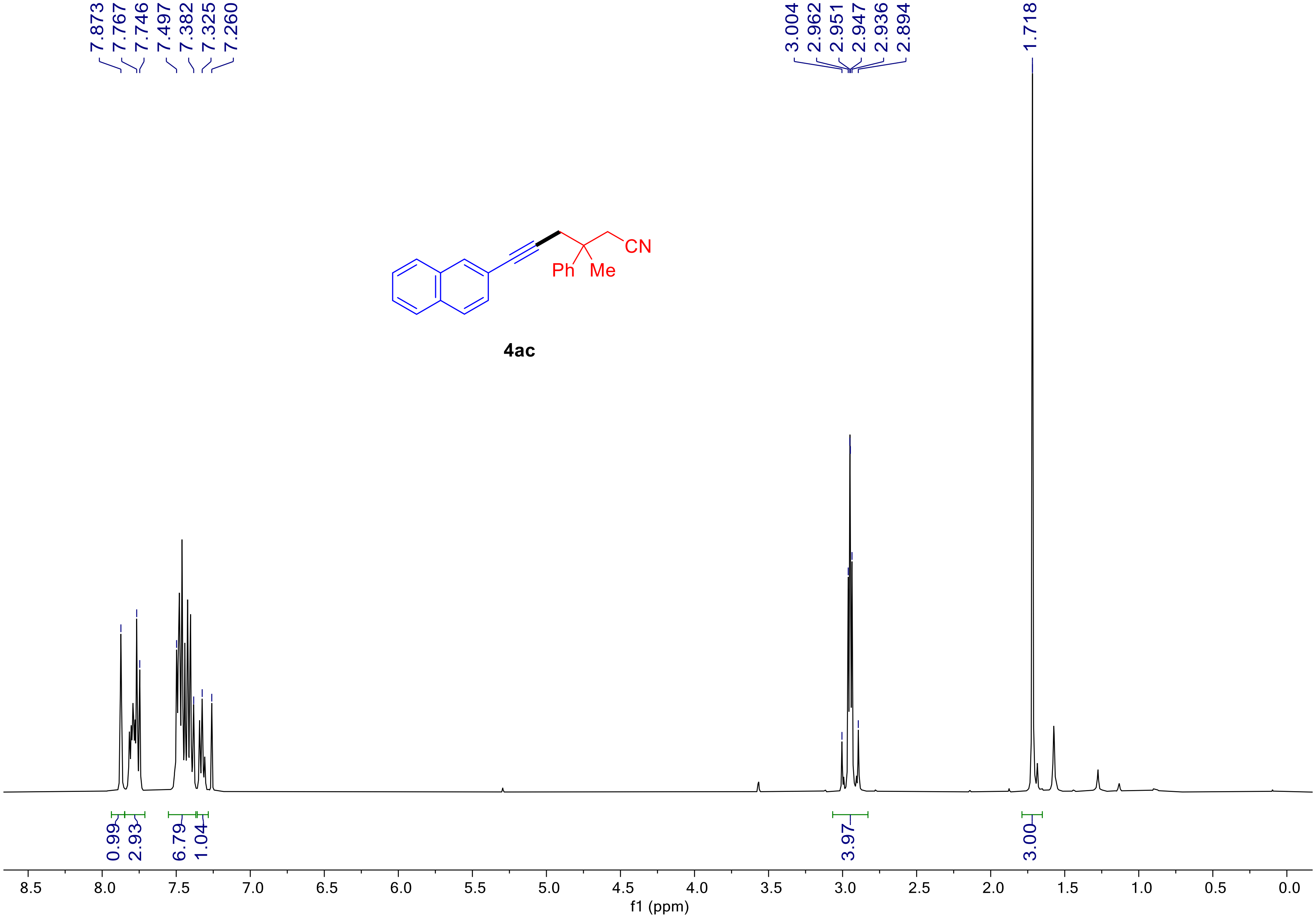

Supplementary Figure 113. ^{1}H NMR spectrum of 4ac

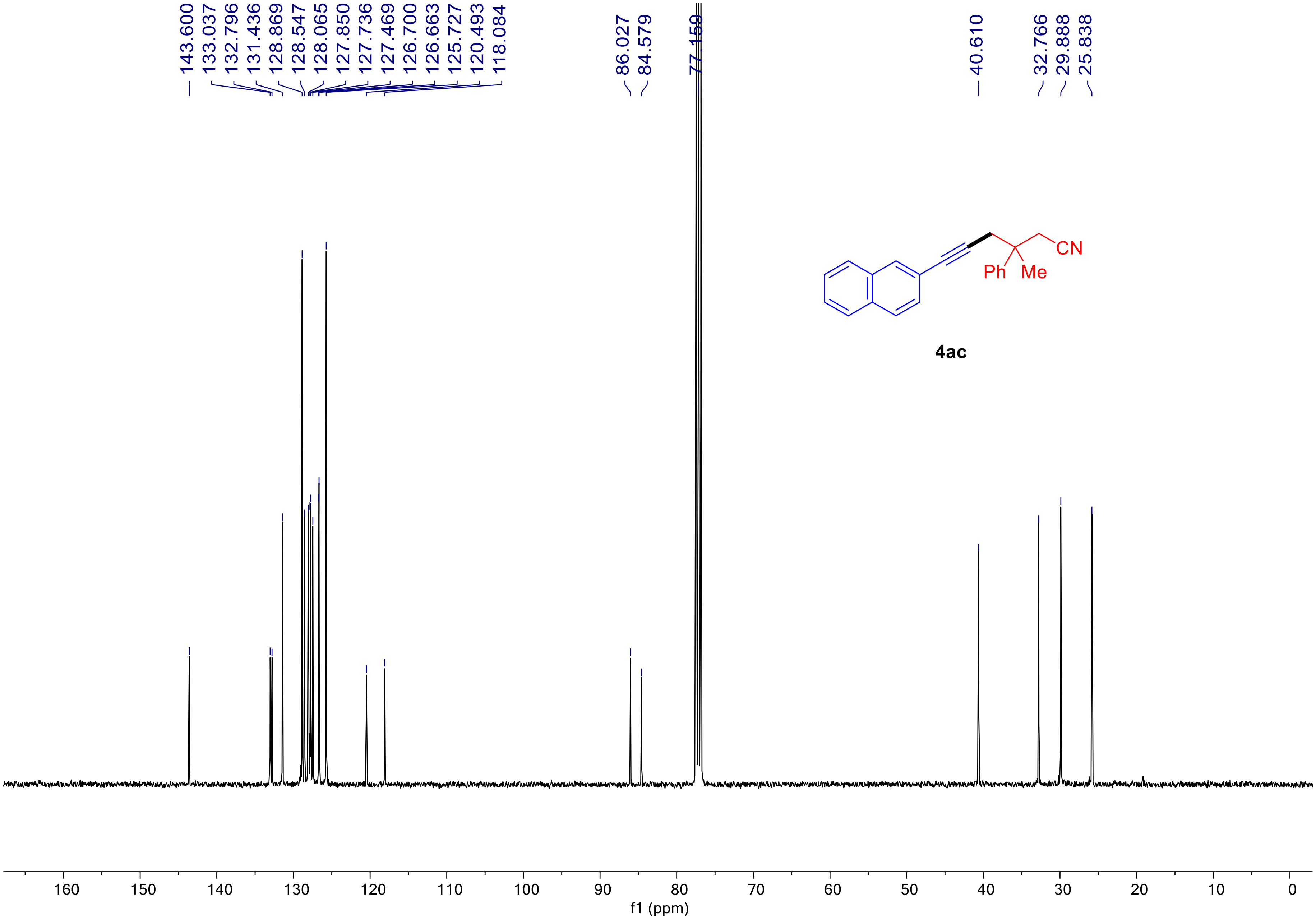

Supplementary Figure 114. ^{13}C NMR spectrum of 4ac

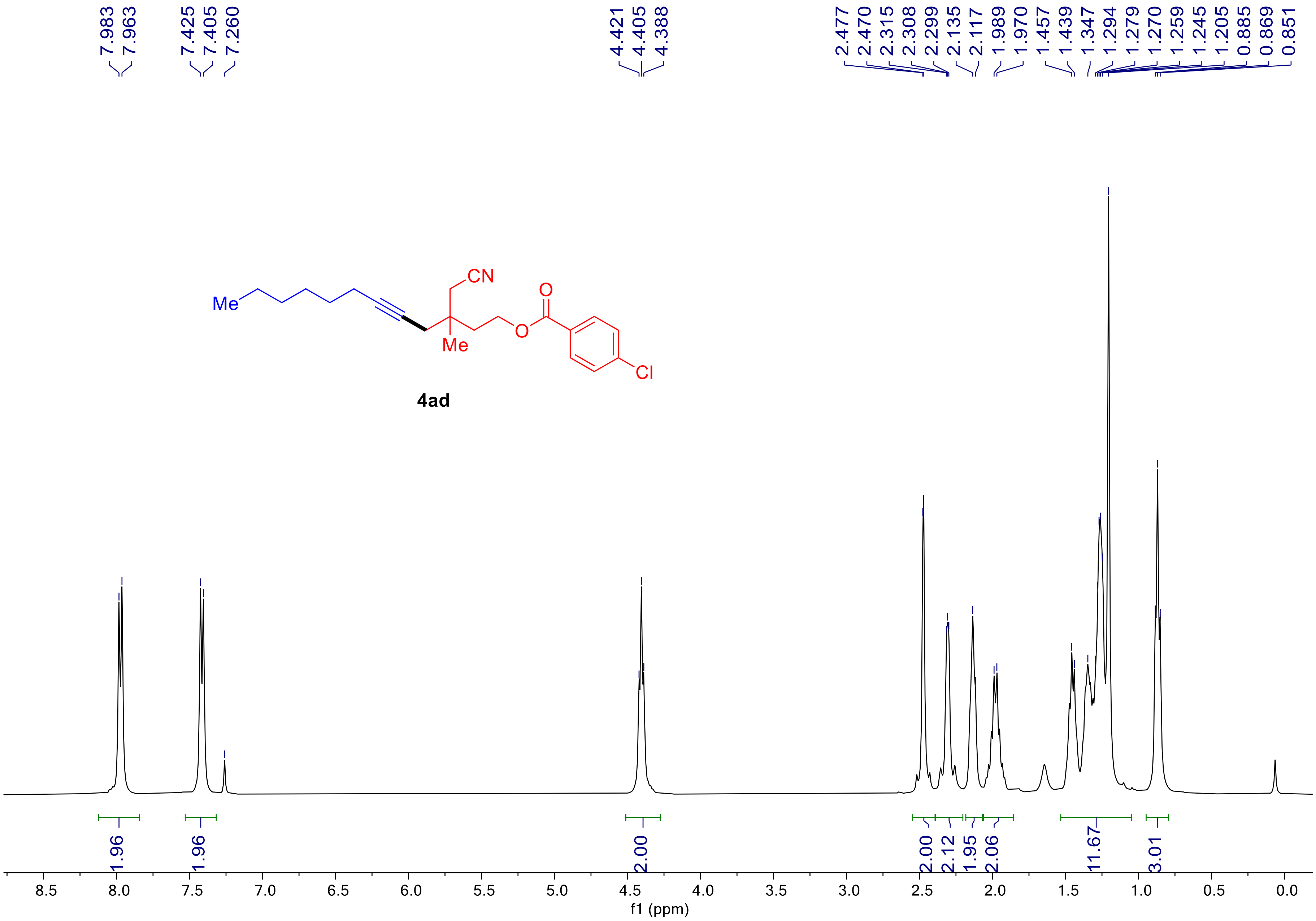

Supplementary Figure 115. ^{1}H NMR spectrum of 4ad

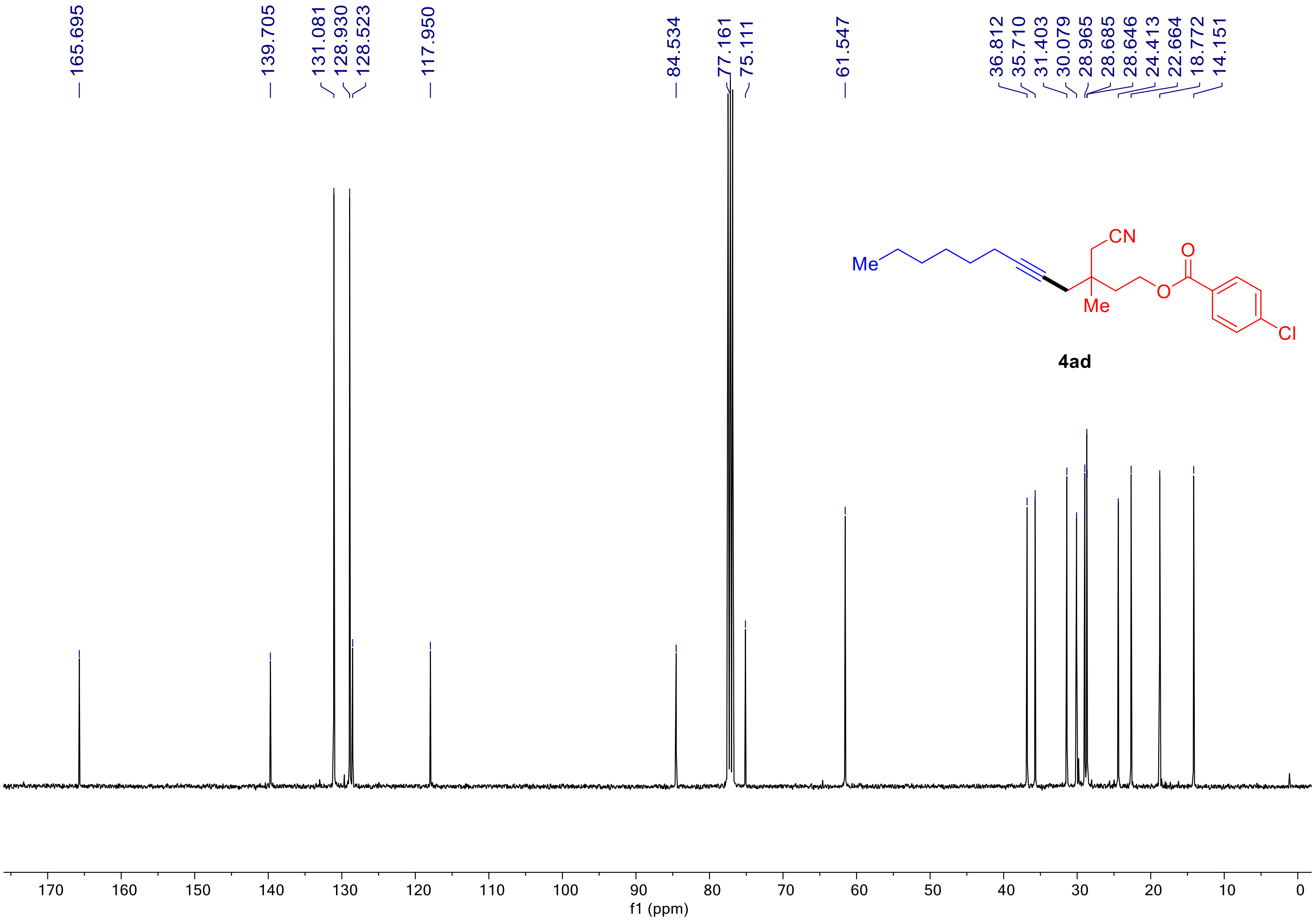

Supplementary Figure 116. ^{13}C NMR spectrum of 4ad

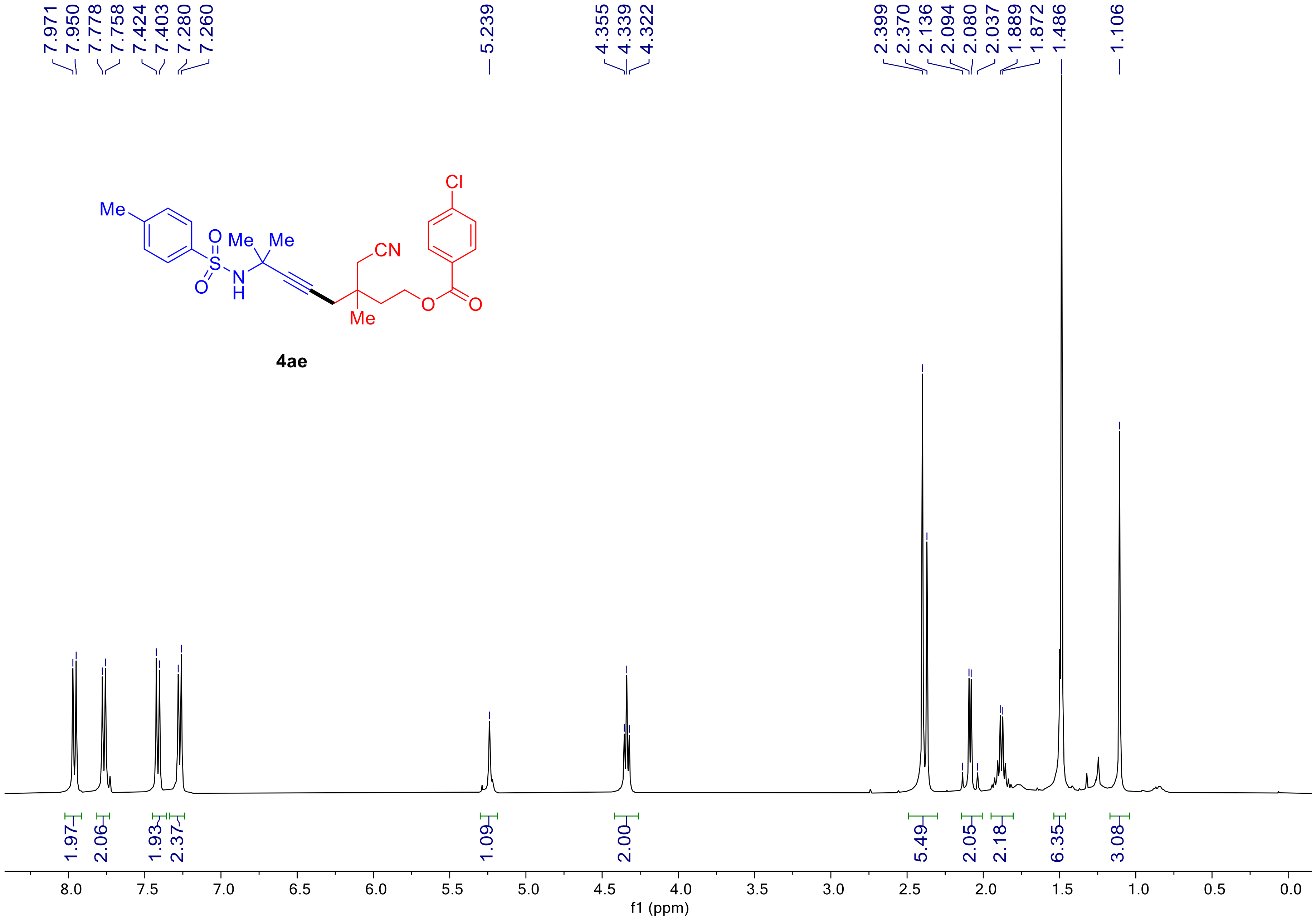

Supplementary Figure 117. ^{1}H NMR spectrum of 4ae

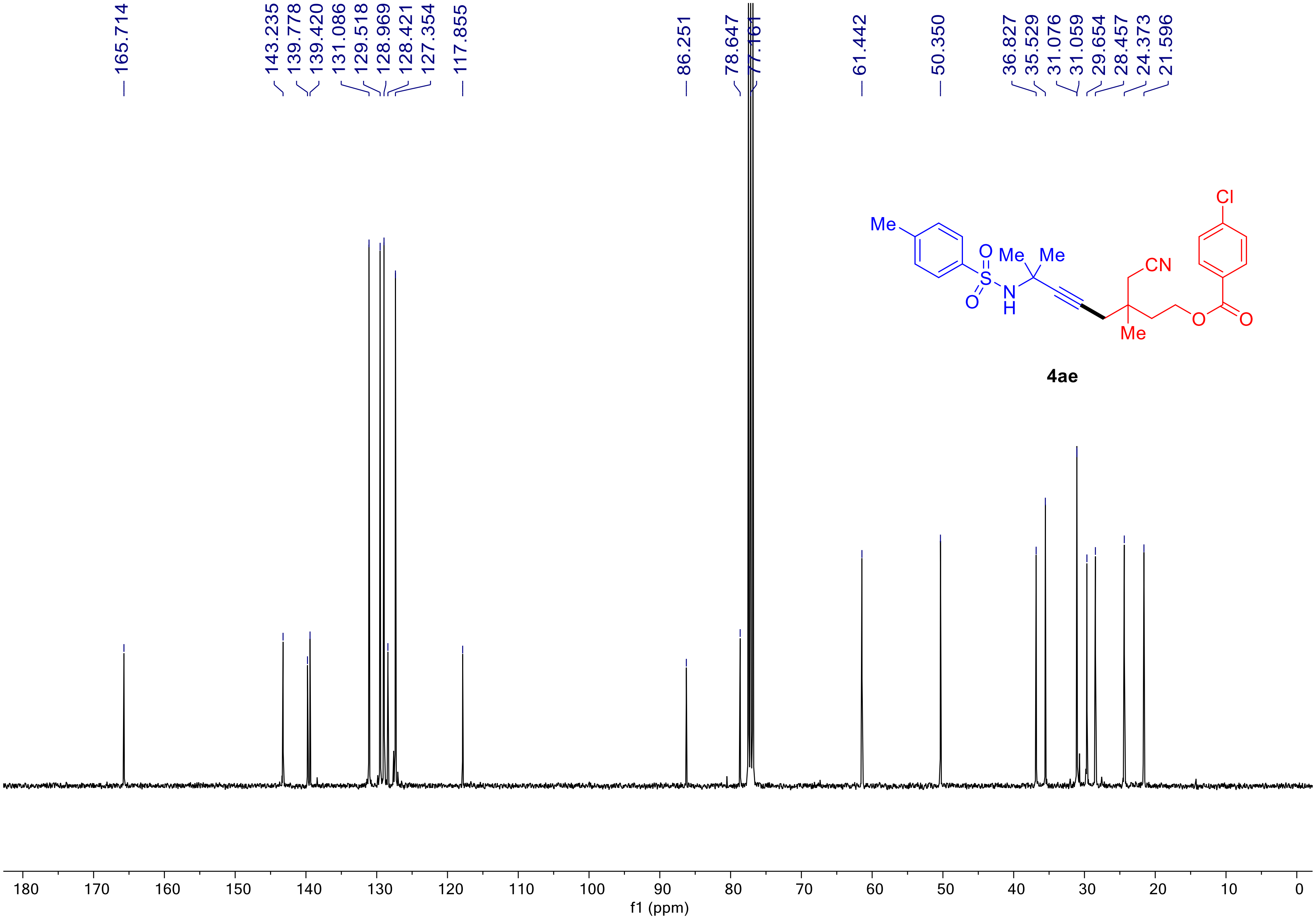

Supplementary Figure 118. ^{13}**C NMR spectrum of 4ae**

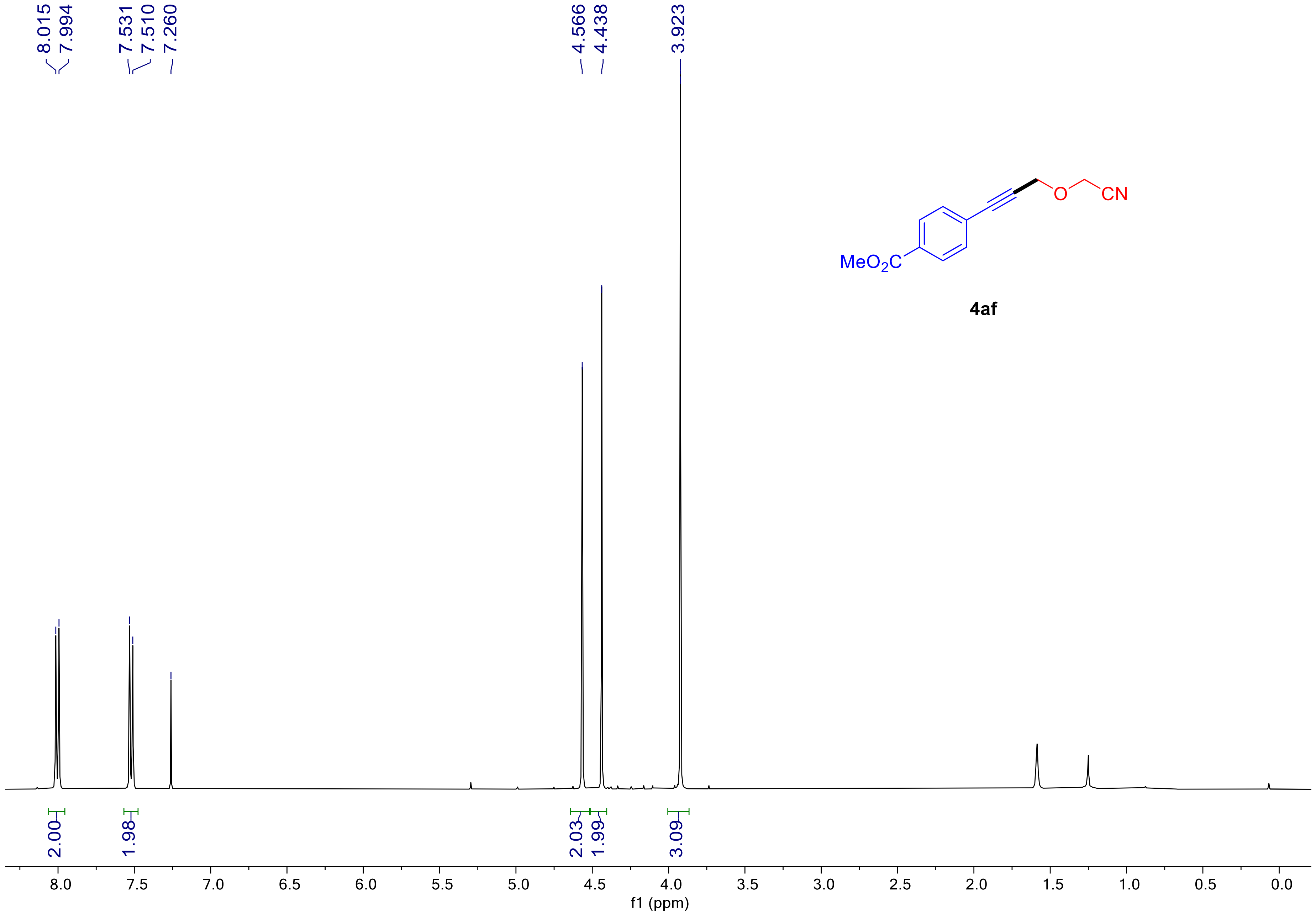

Supplementary Figure 119. ^{1}H NMR spectrum of 4af

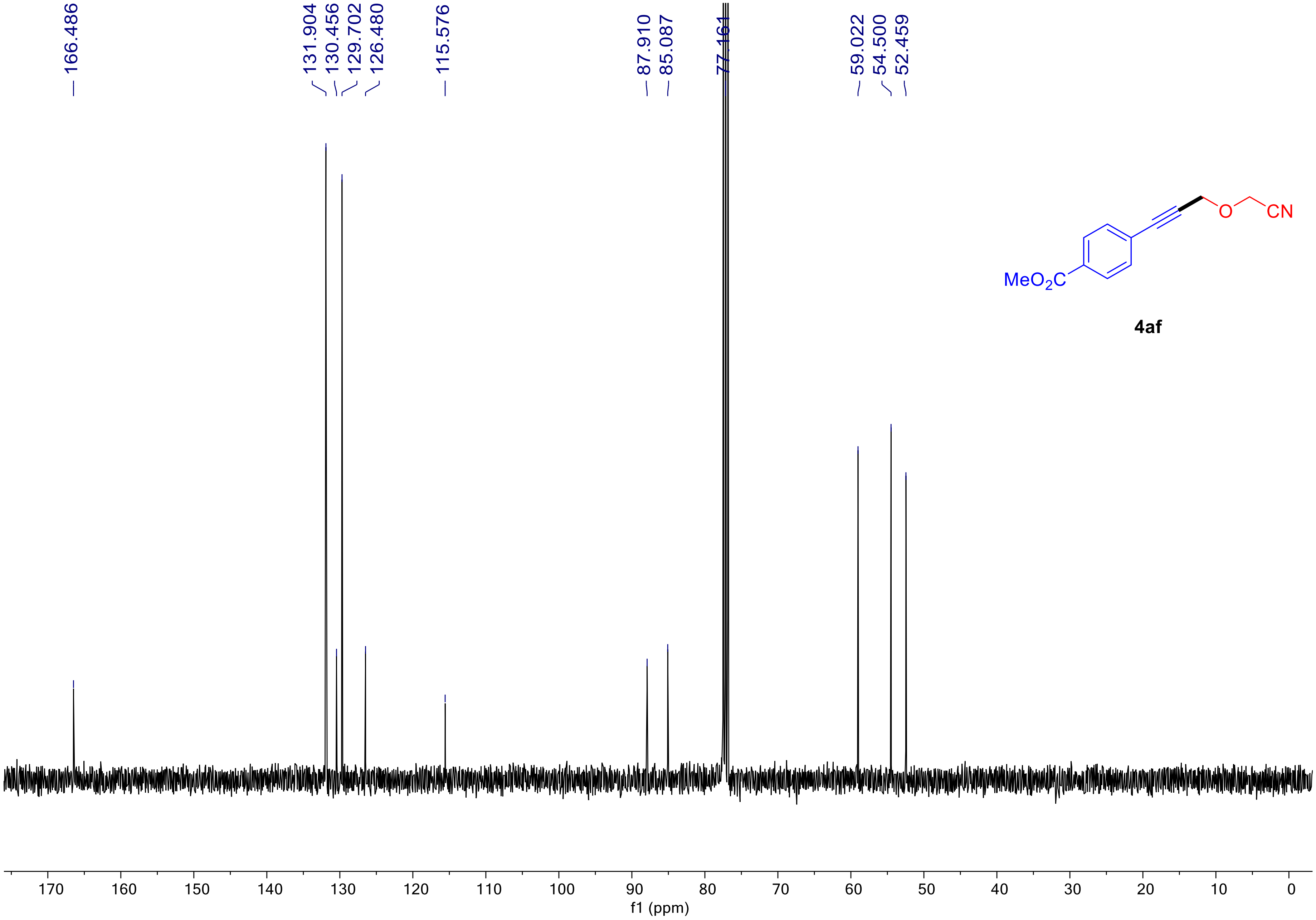

Supplementary Figure 120. ^{13}C NMR spectrum of 4af

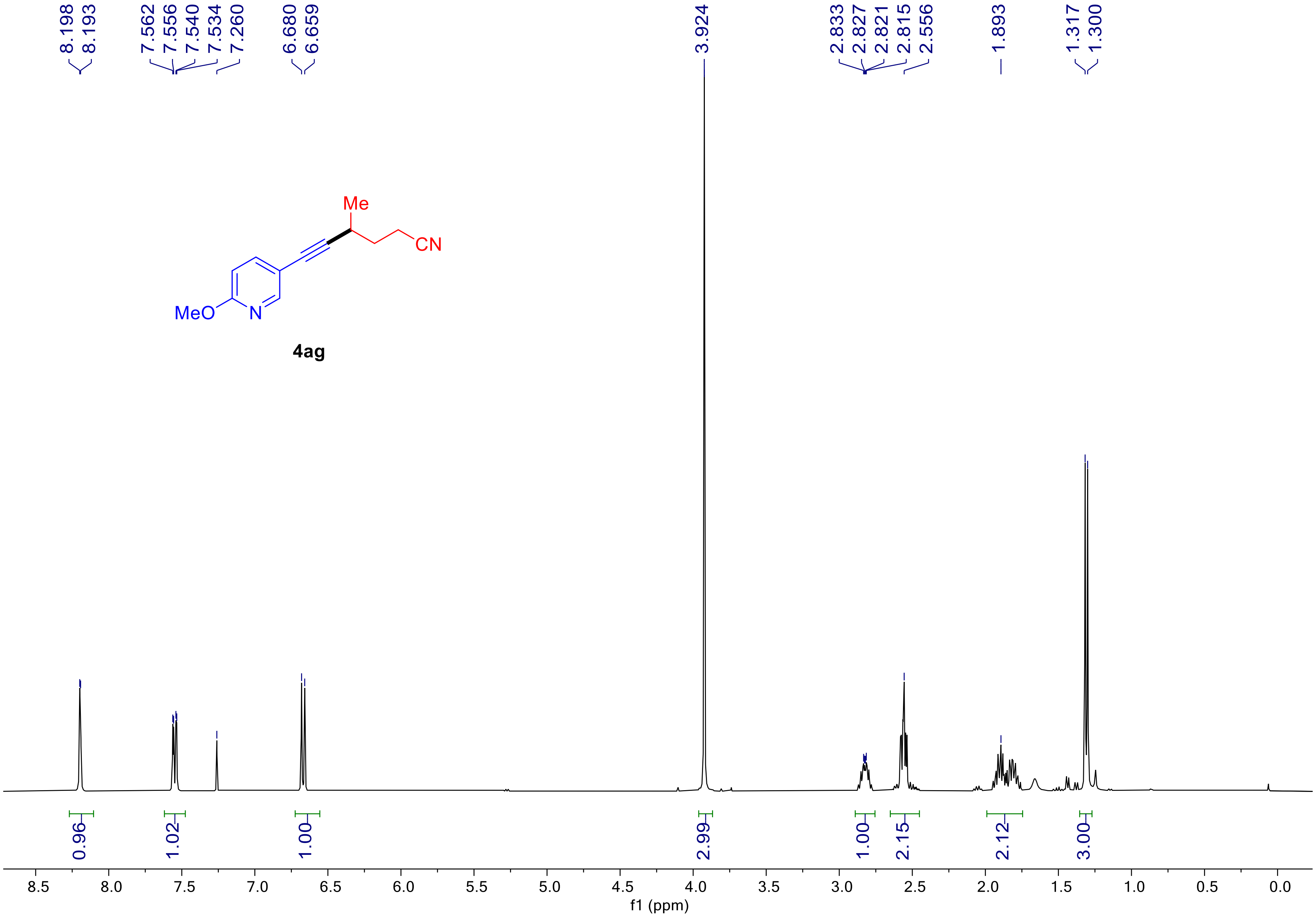

Supplementary Figure 121. ^{1}H NMR spectrum of 4ag

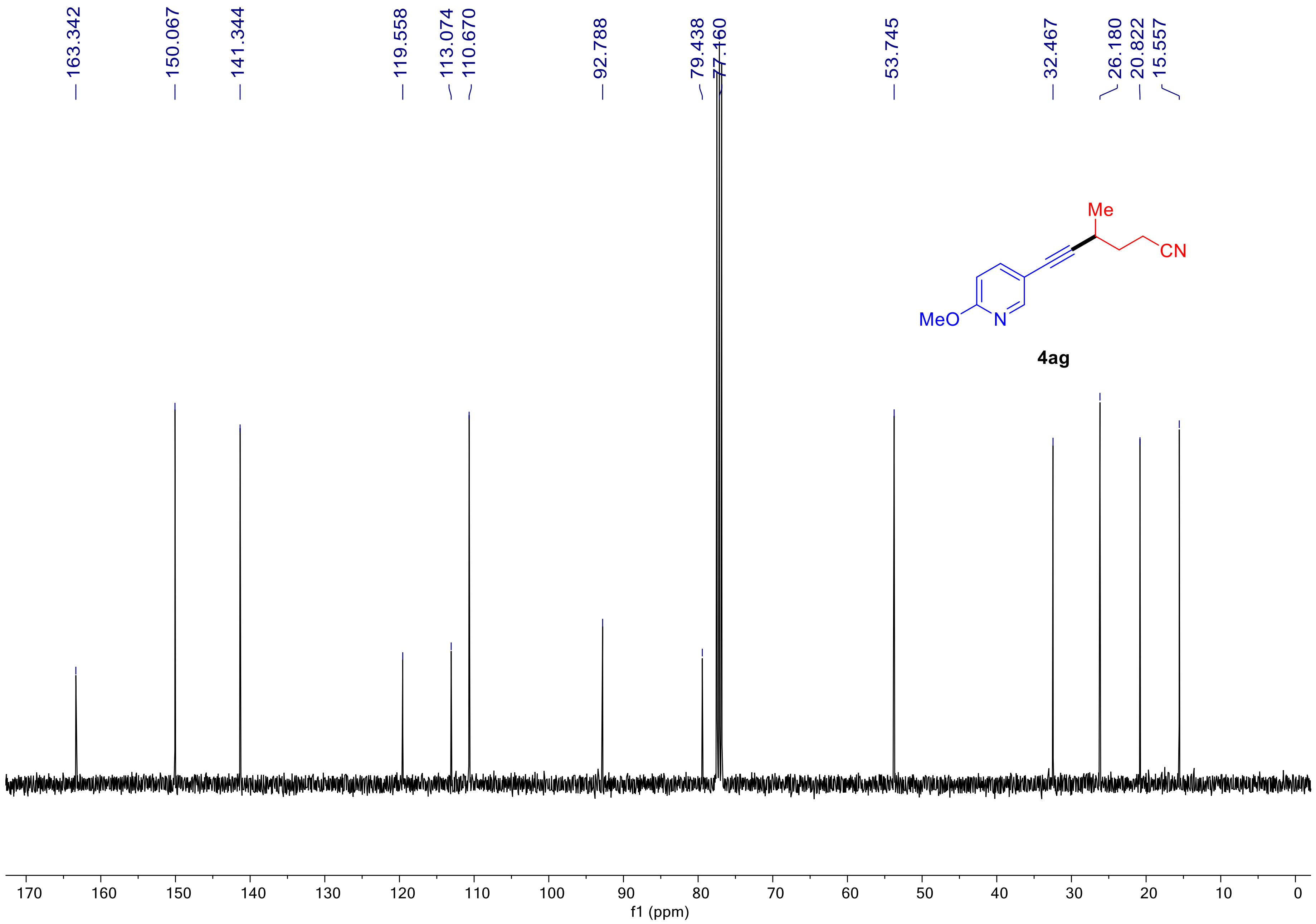

Supplementary Figure 122. ^{13}C NMR spectrum of 4ag

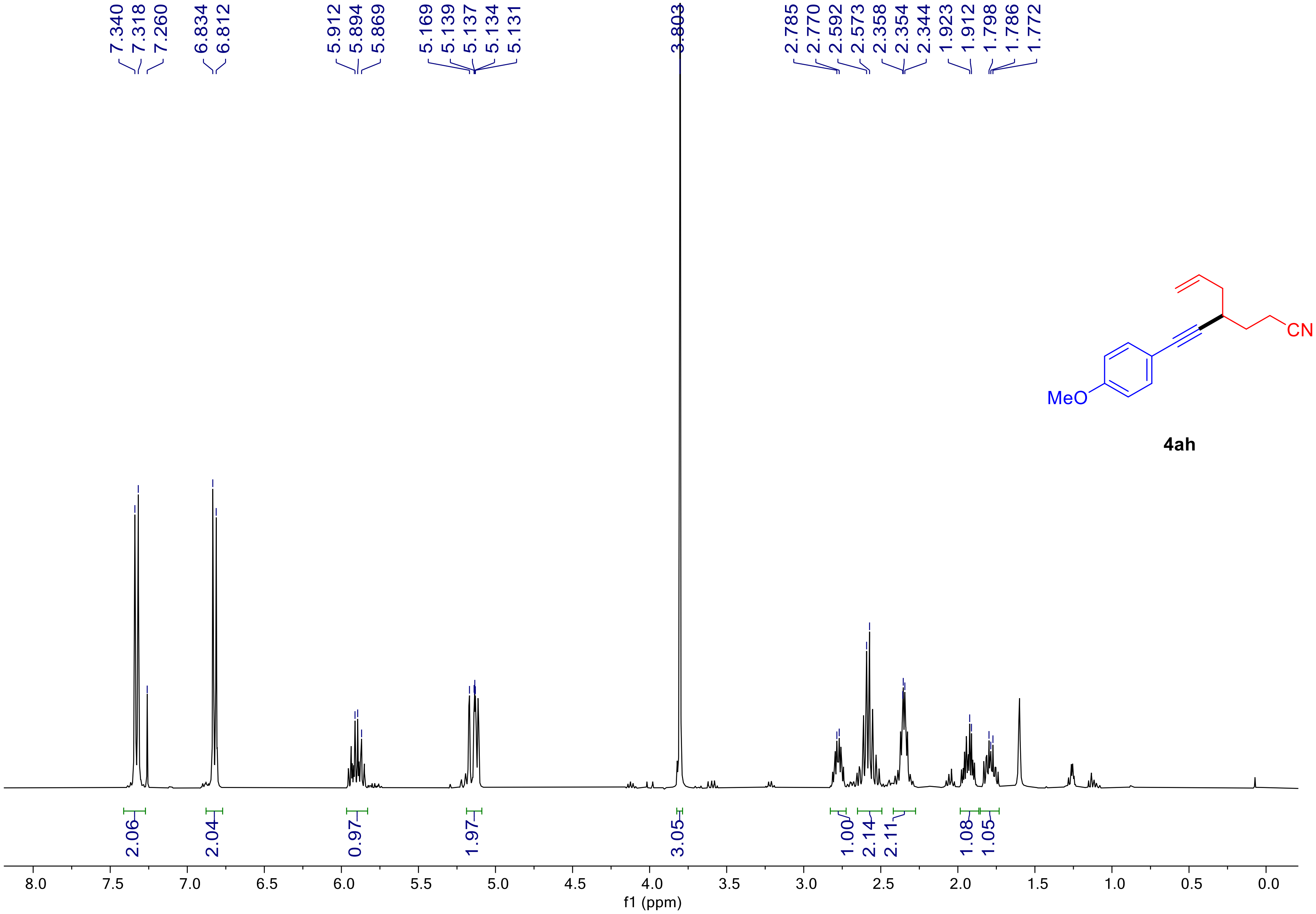

Supplementary Figure 123. ^{1}H NMR spectrum of 4ah

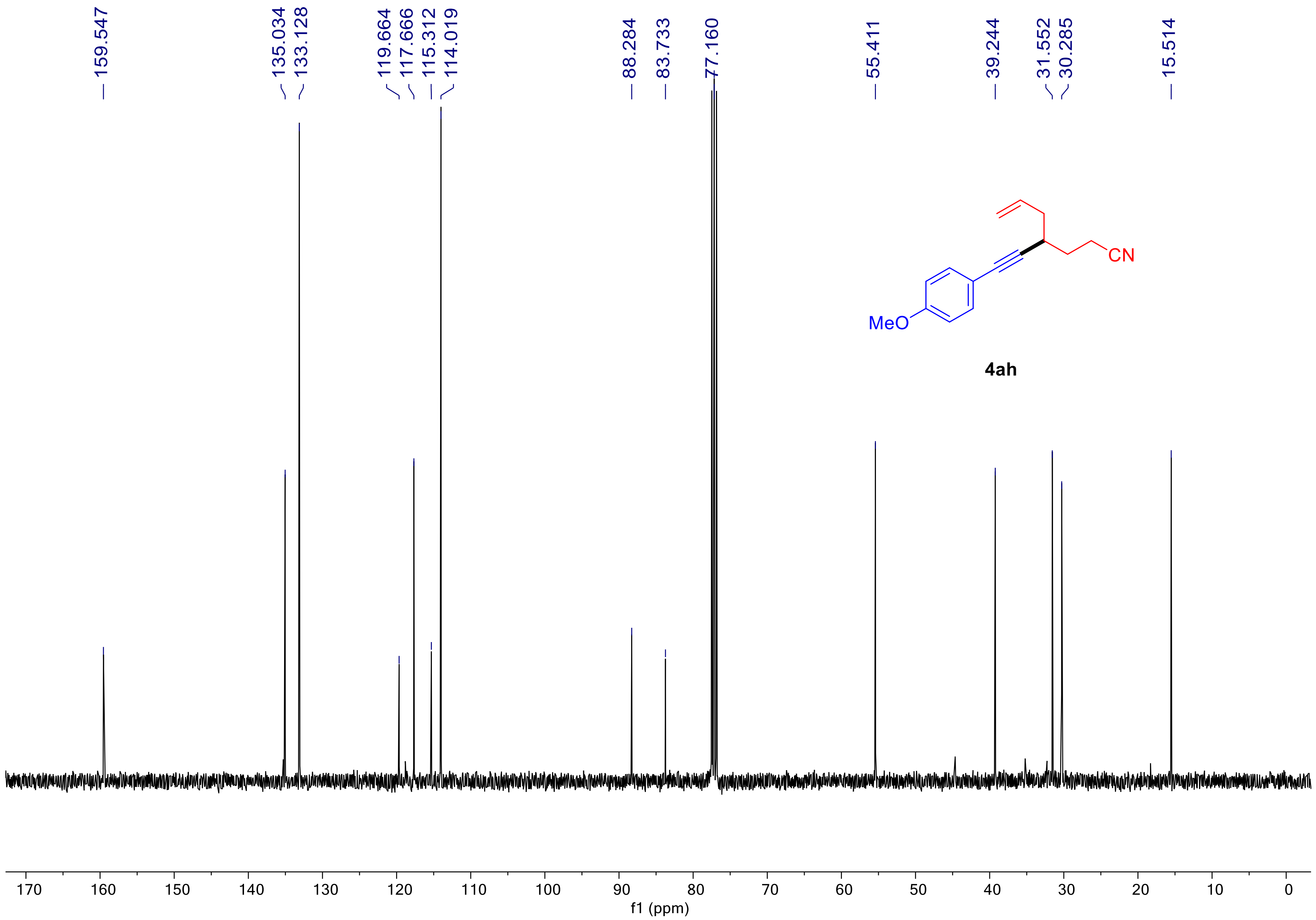

Supplementary Figure 124. ^{13}C NMR spectrum of 4ah

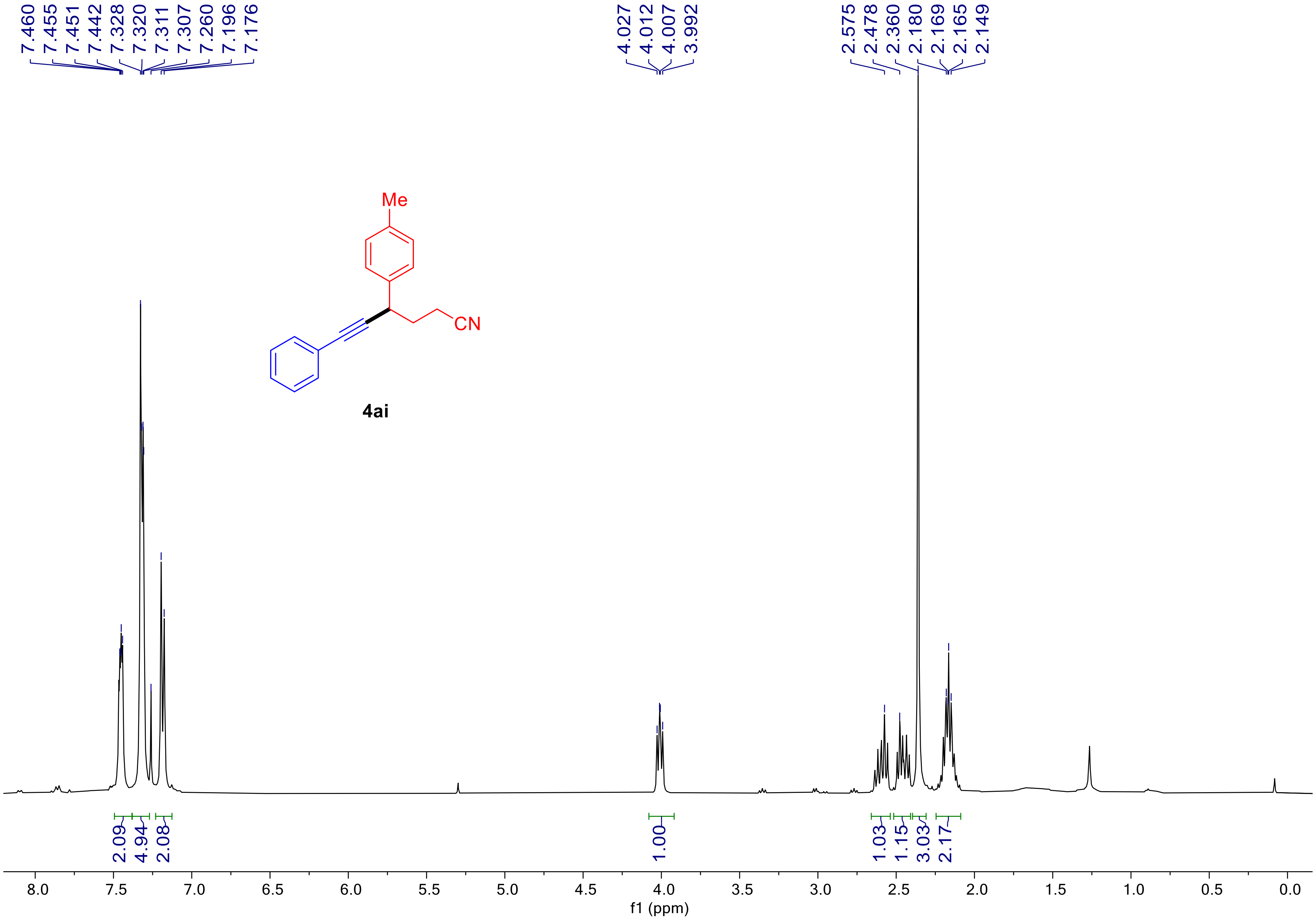

Supplementary Figure 125. ^{1}H NMR spectrum of 4ai

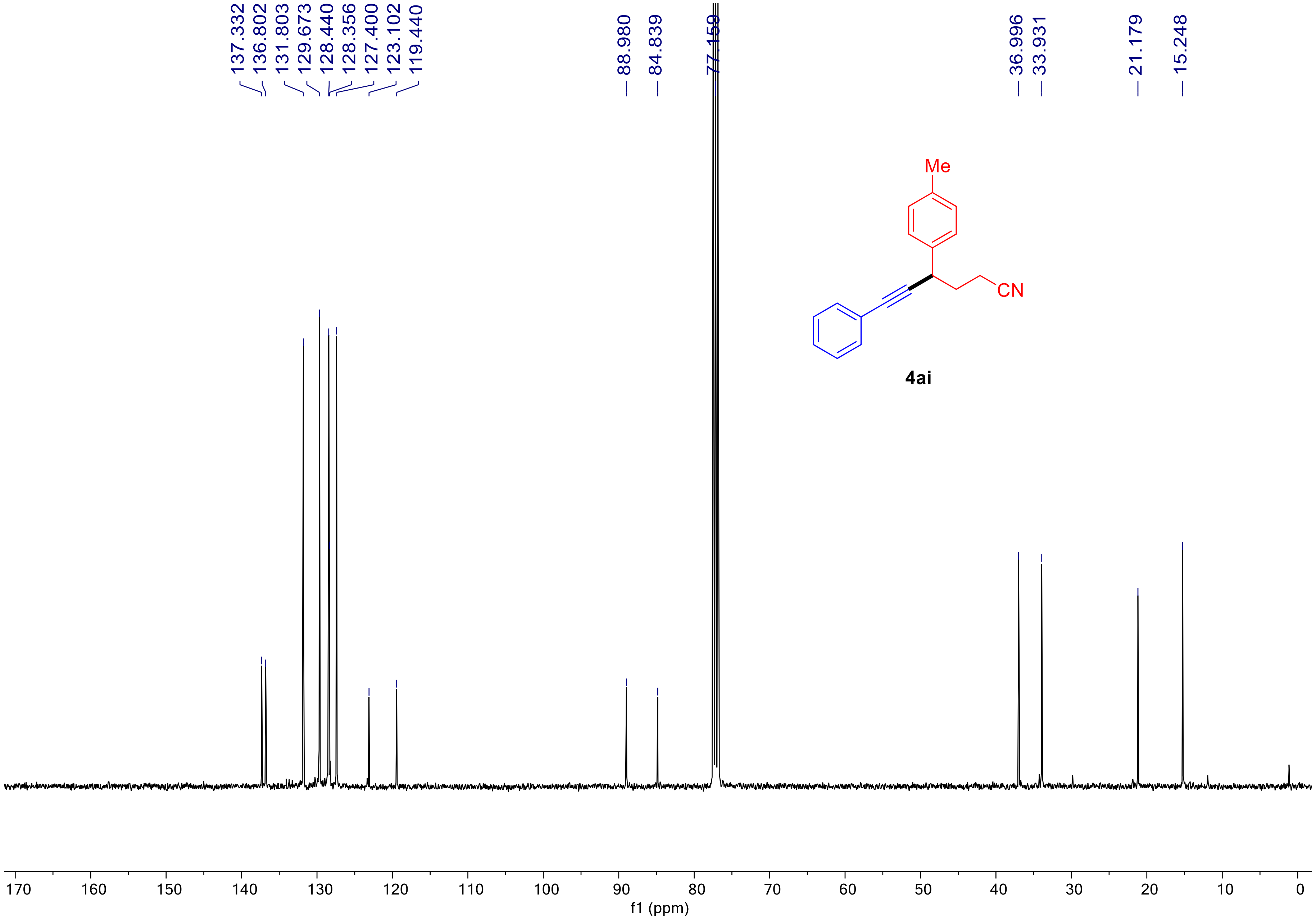

Supplementary Figure 126. ^{13}C NMR spectrum of 4ai

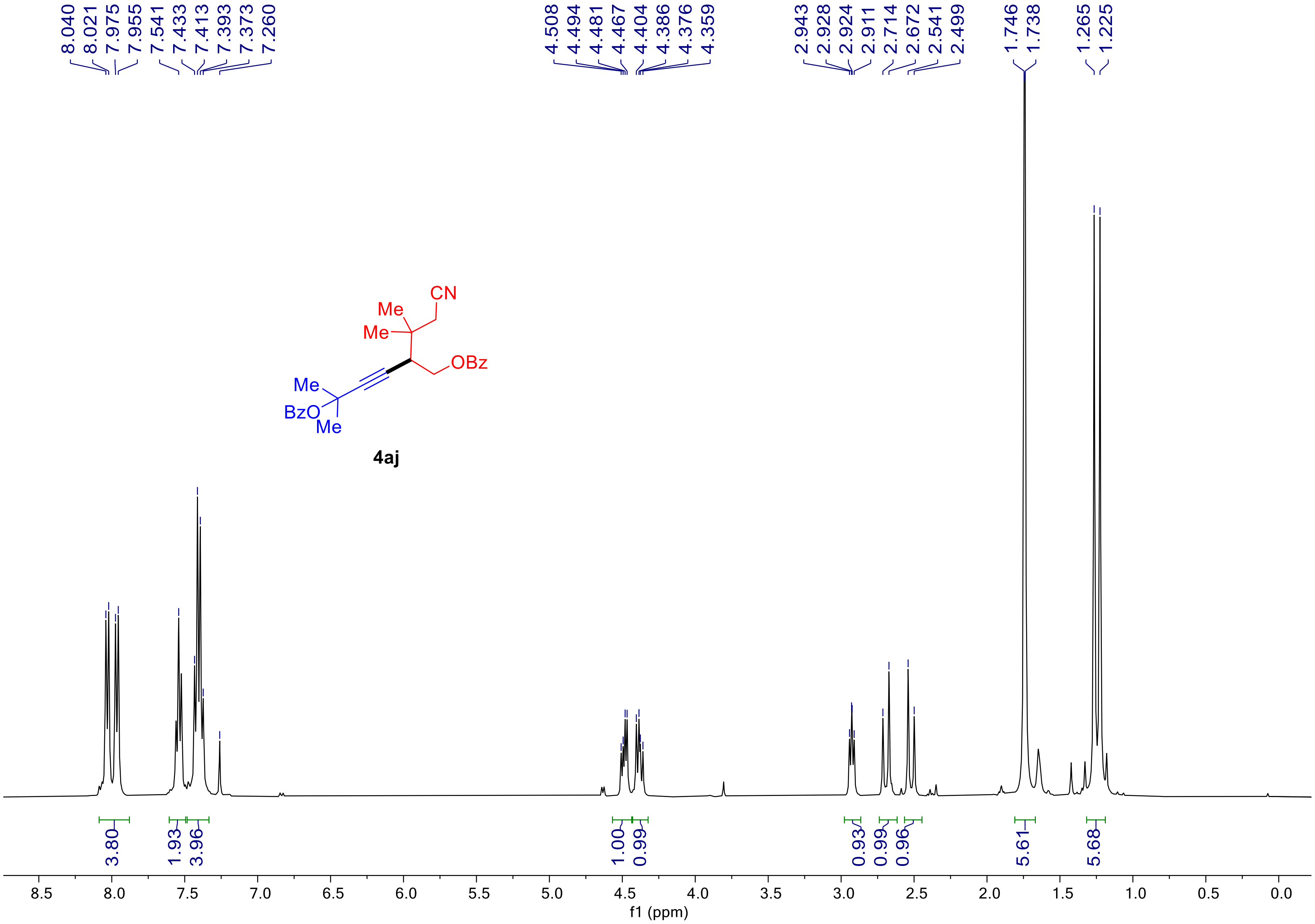

Supplementary Figure 127. ^{1}H NMR spectrum of 4aj

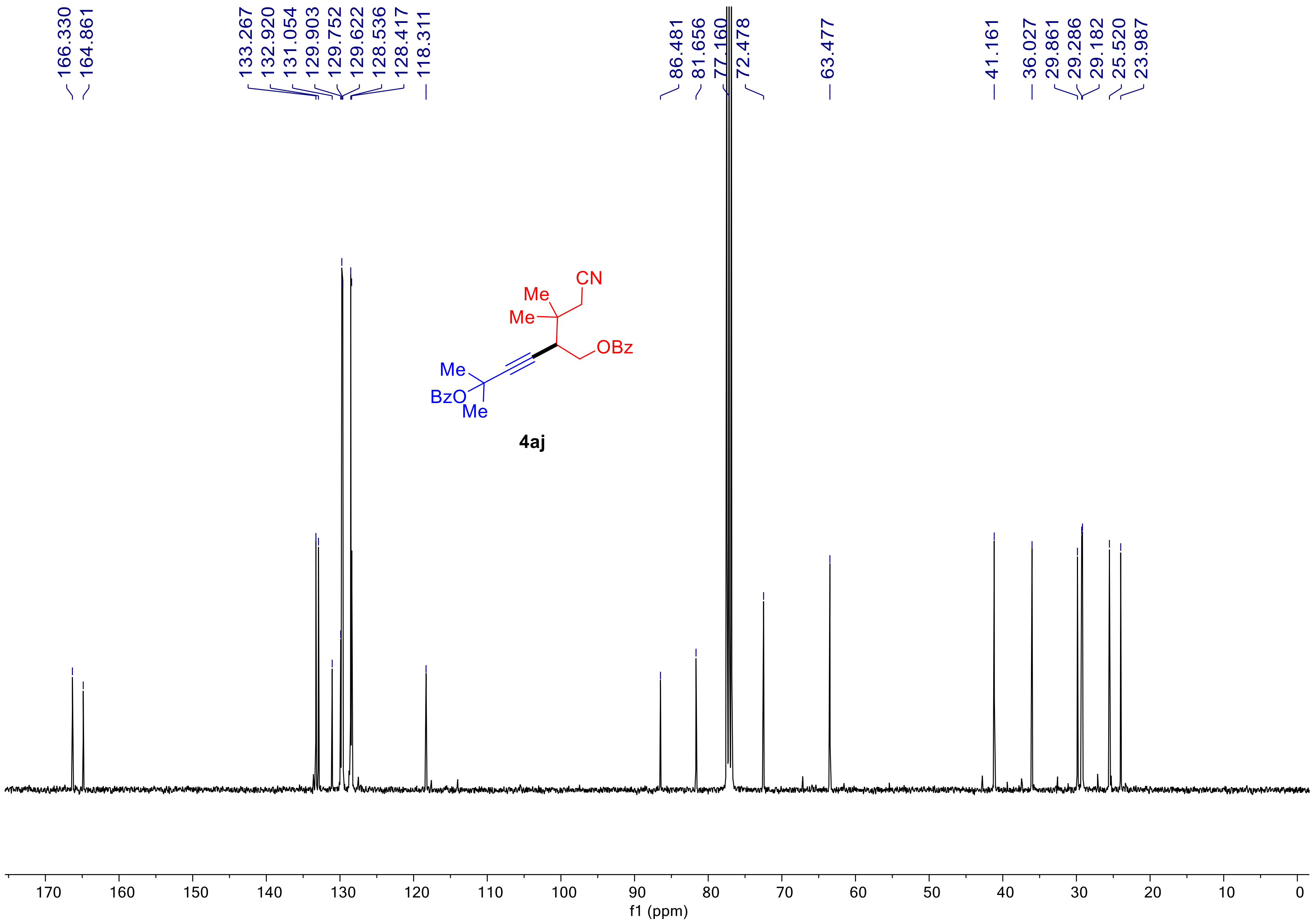

Supplementary Figure 128. ^{13}**C NMR spectrum of 4aj**

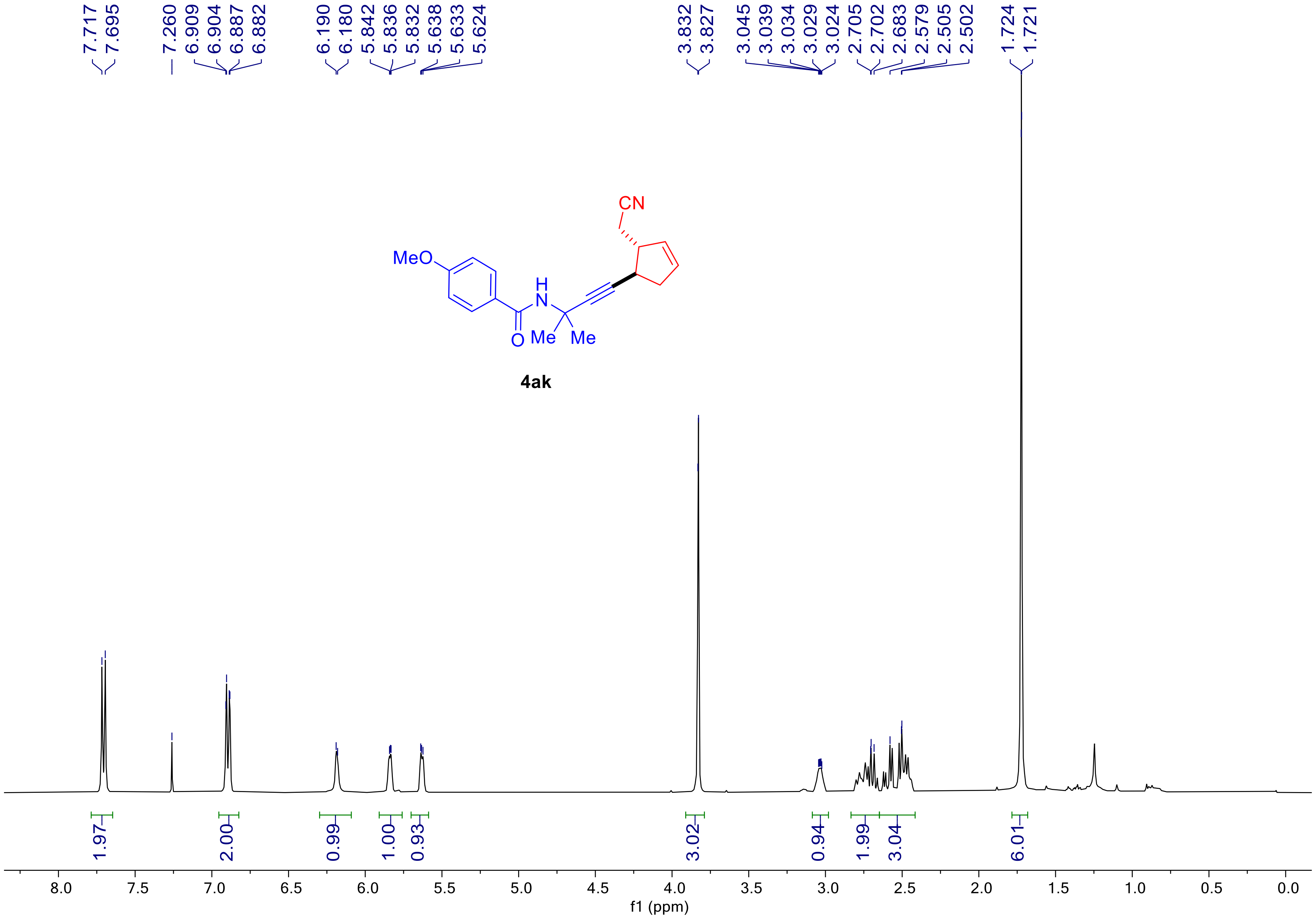

Supplementary Figure 129. ^{1}H NMR spectrum of 4ak

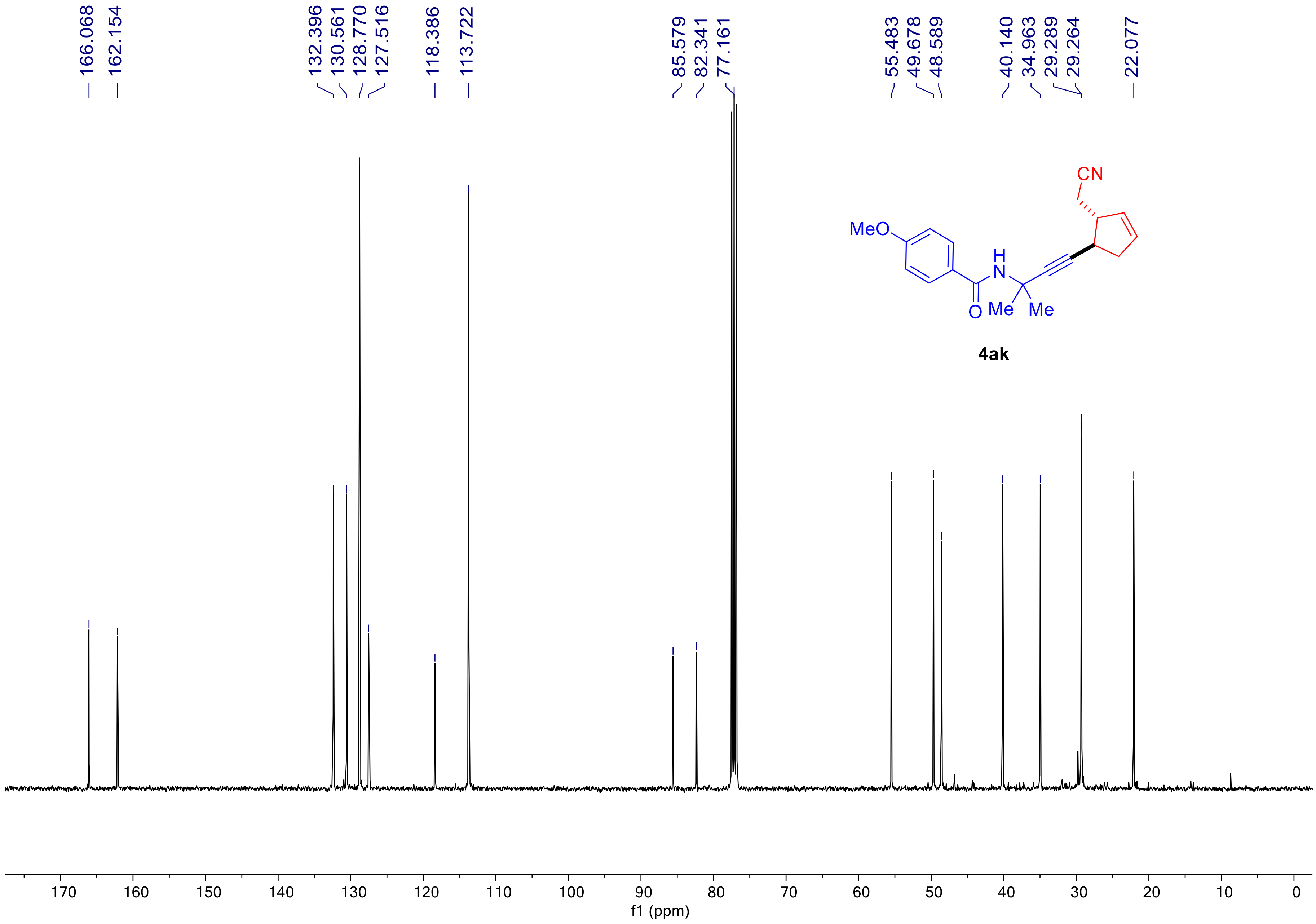

Supplementary Figure 130. ^{13}C NMR spectrum of 4ak

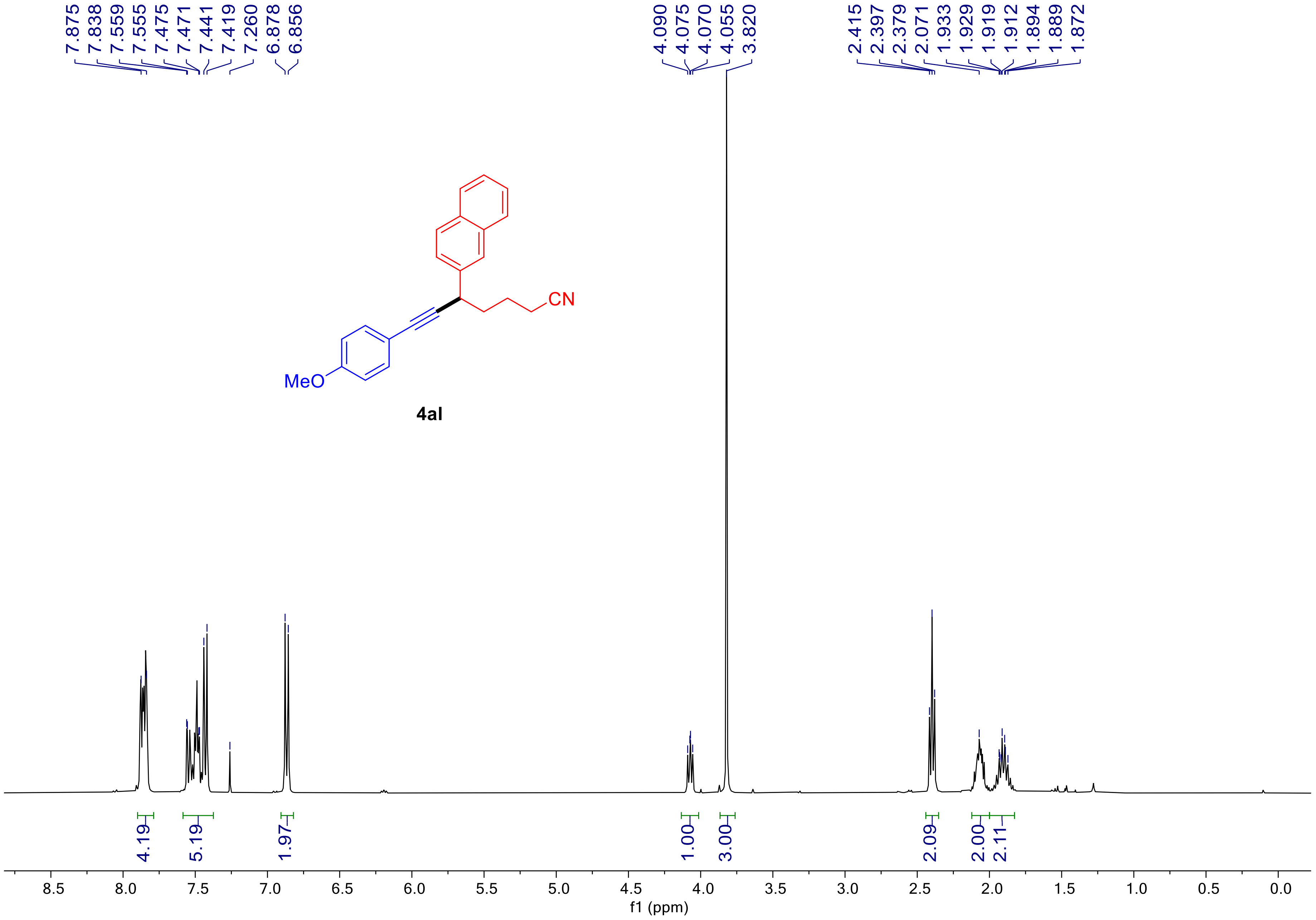

Supplementary Figure 131. ^{1}H NMR spectrum of 4al

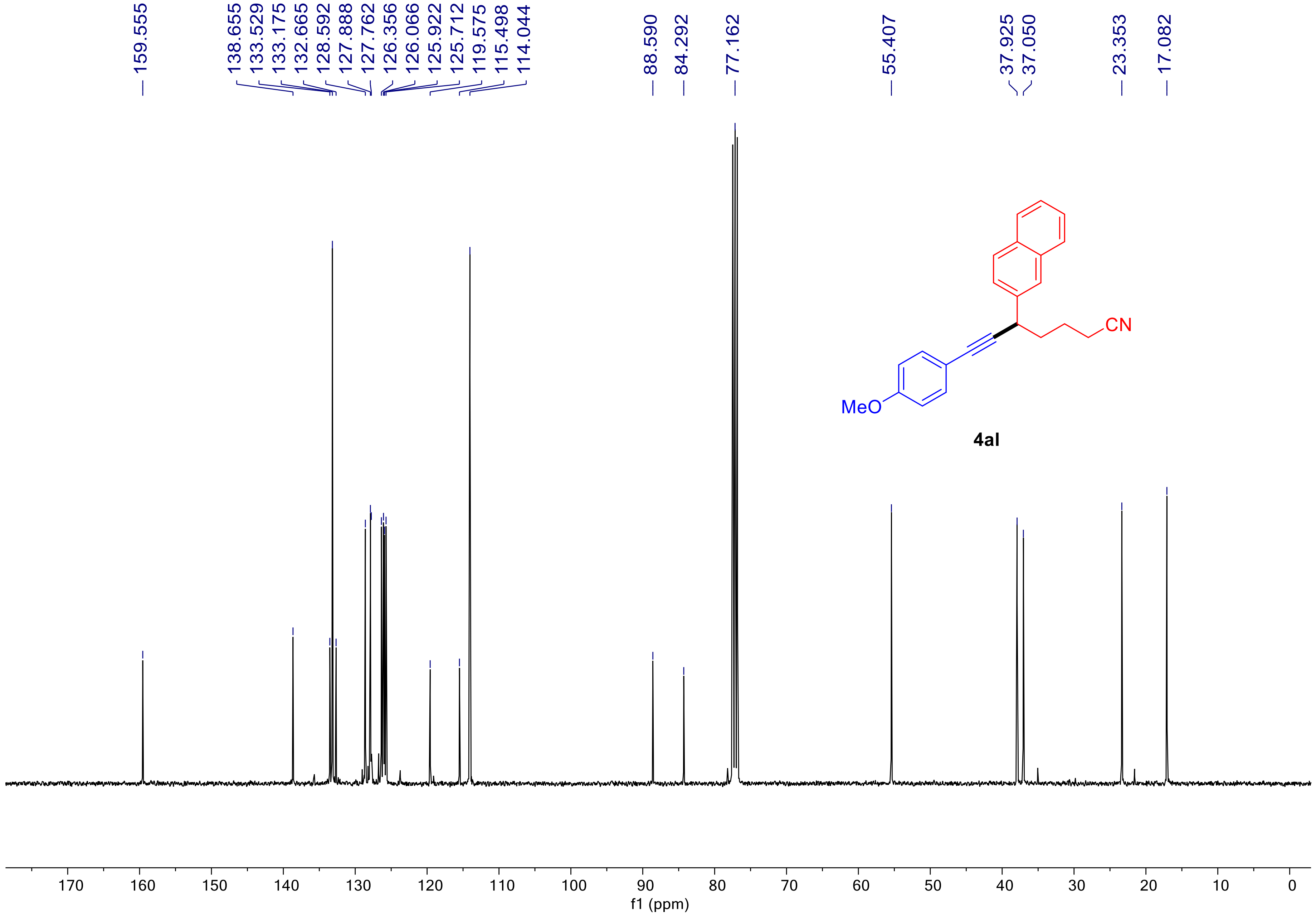

Supplementary Figure 132. ^{13}C NMR spectrum of 4al

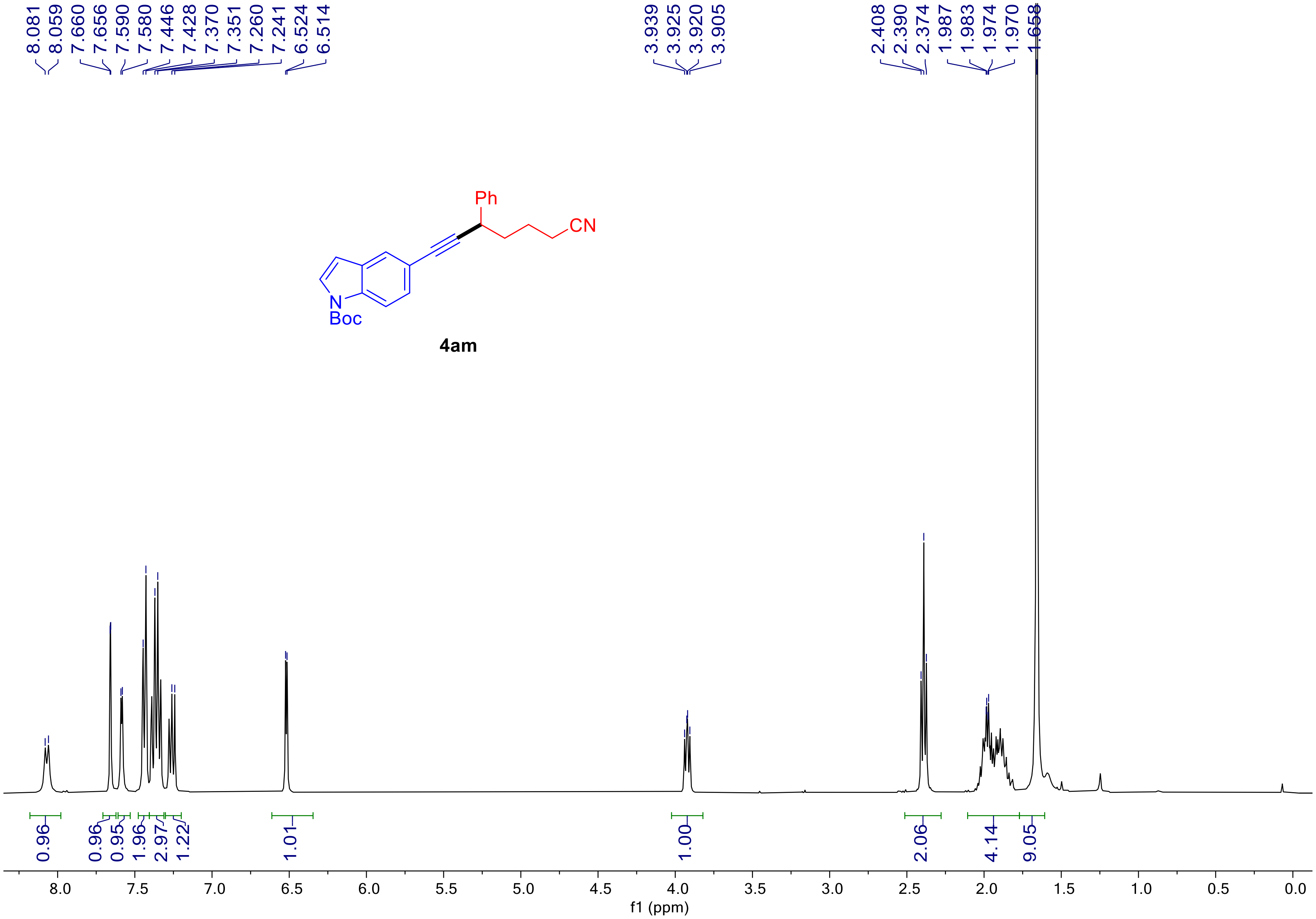

Supplementary Figure 133. ^{1}H NMR spectrum of 4am

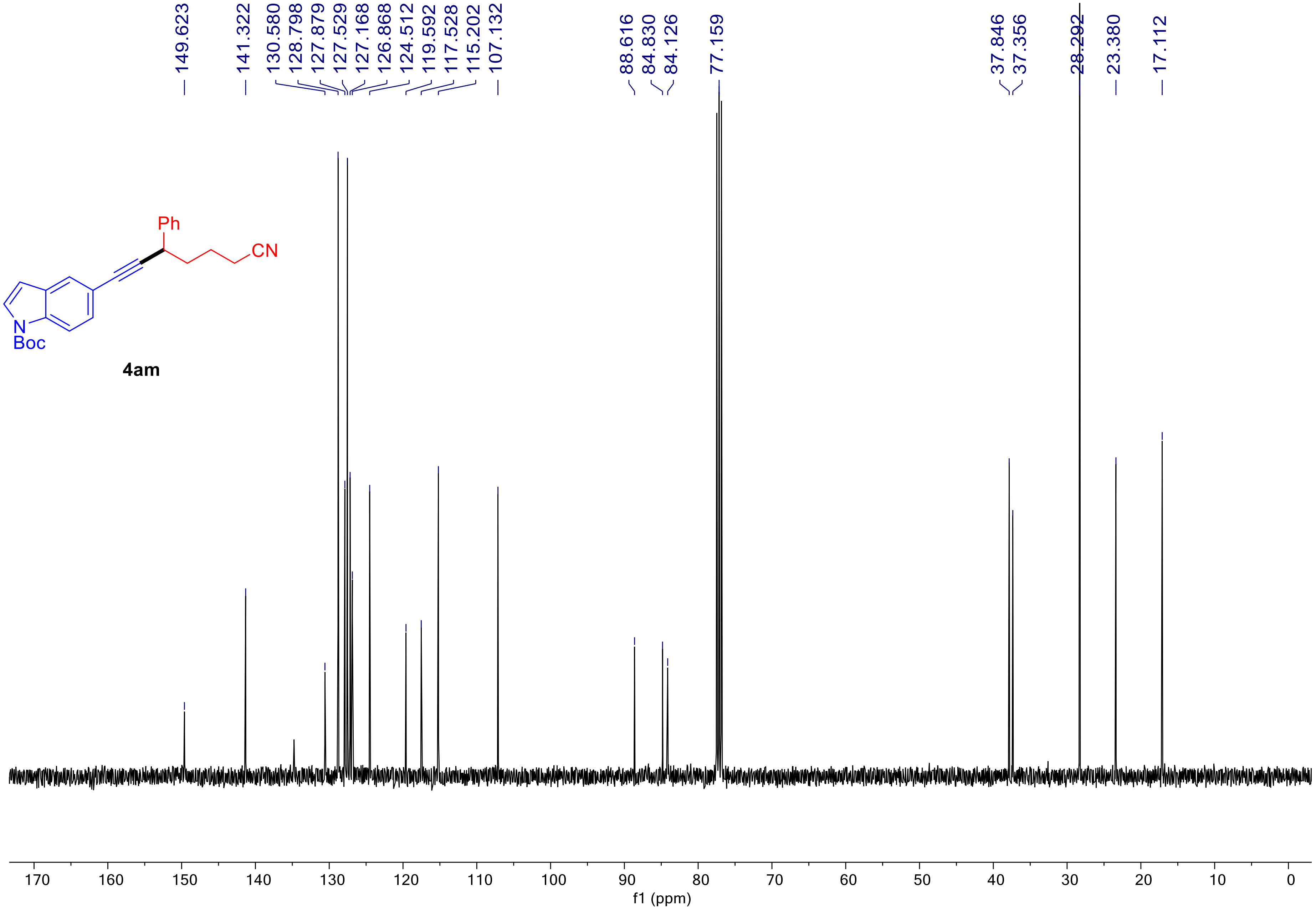

Supplementary Figure 134. ^{13}**C NMR spectrum of 4am**

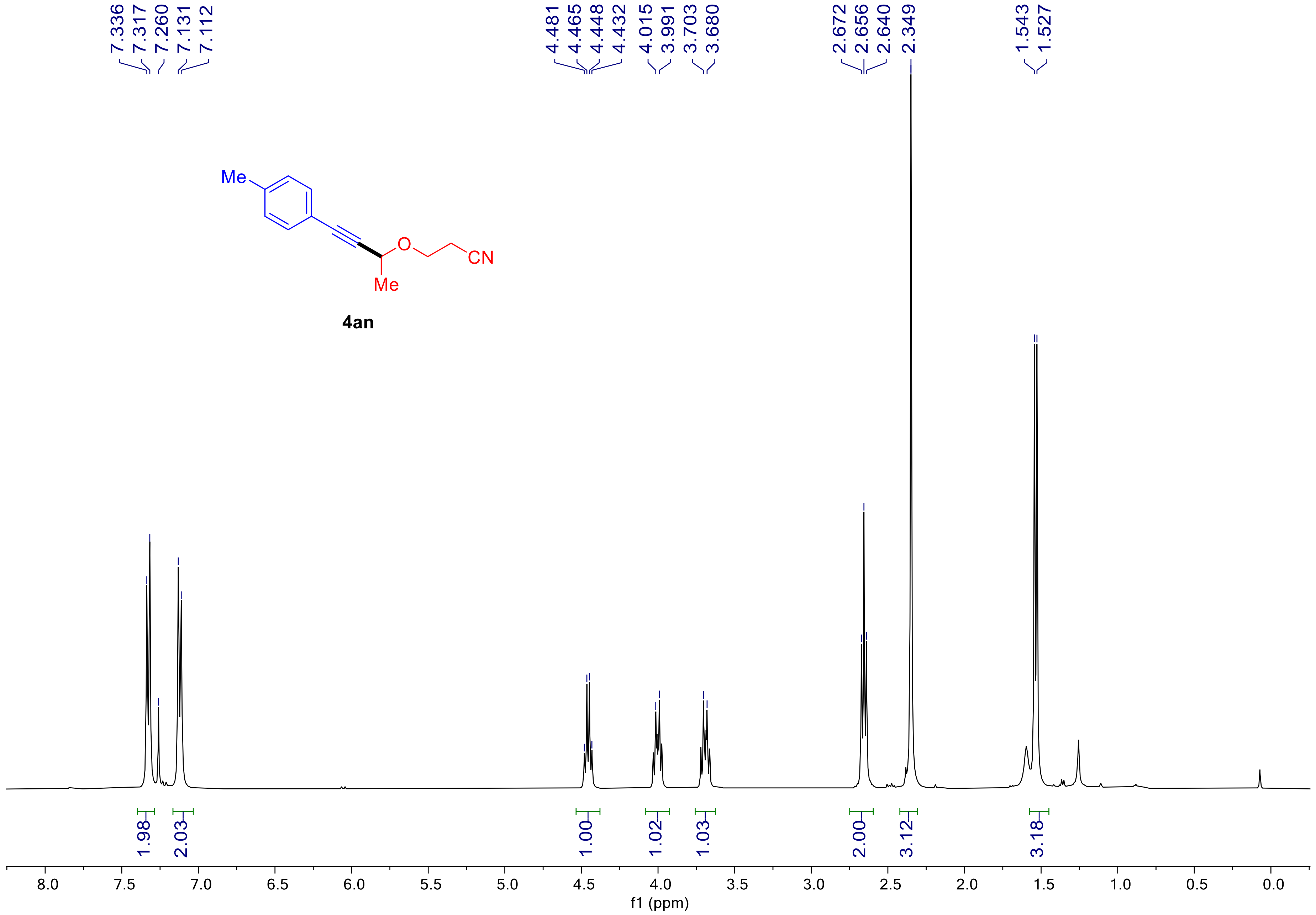

Supplementary Figure 135. ^{1}H NMR spectrum of 4an

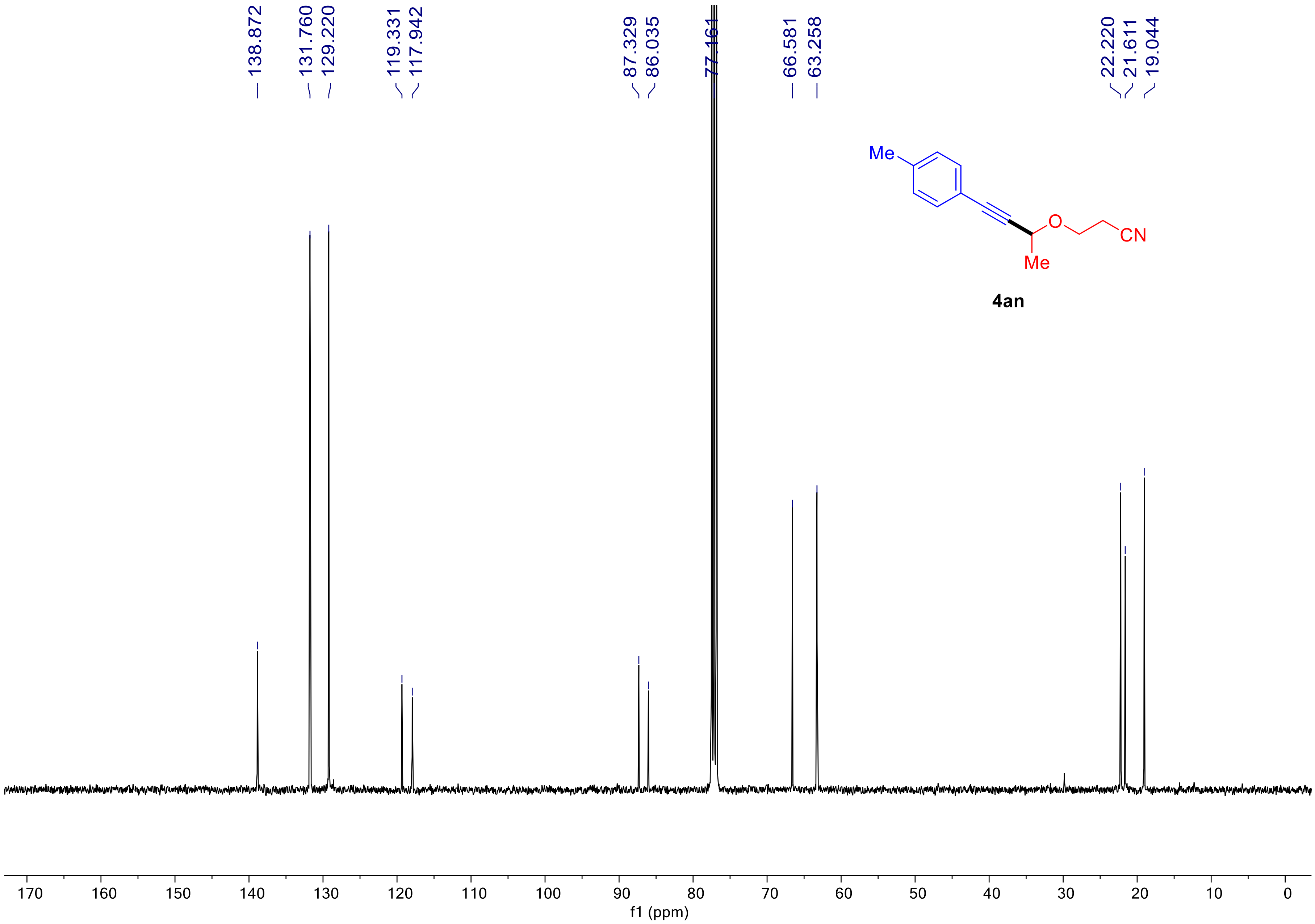

Supplementary Figure 136. ^{13}C NMR spectrum of 4an

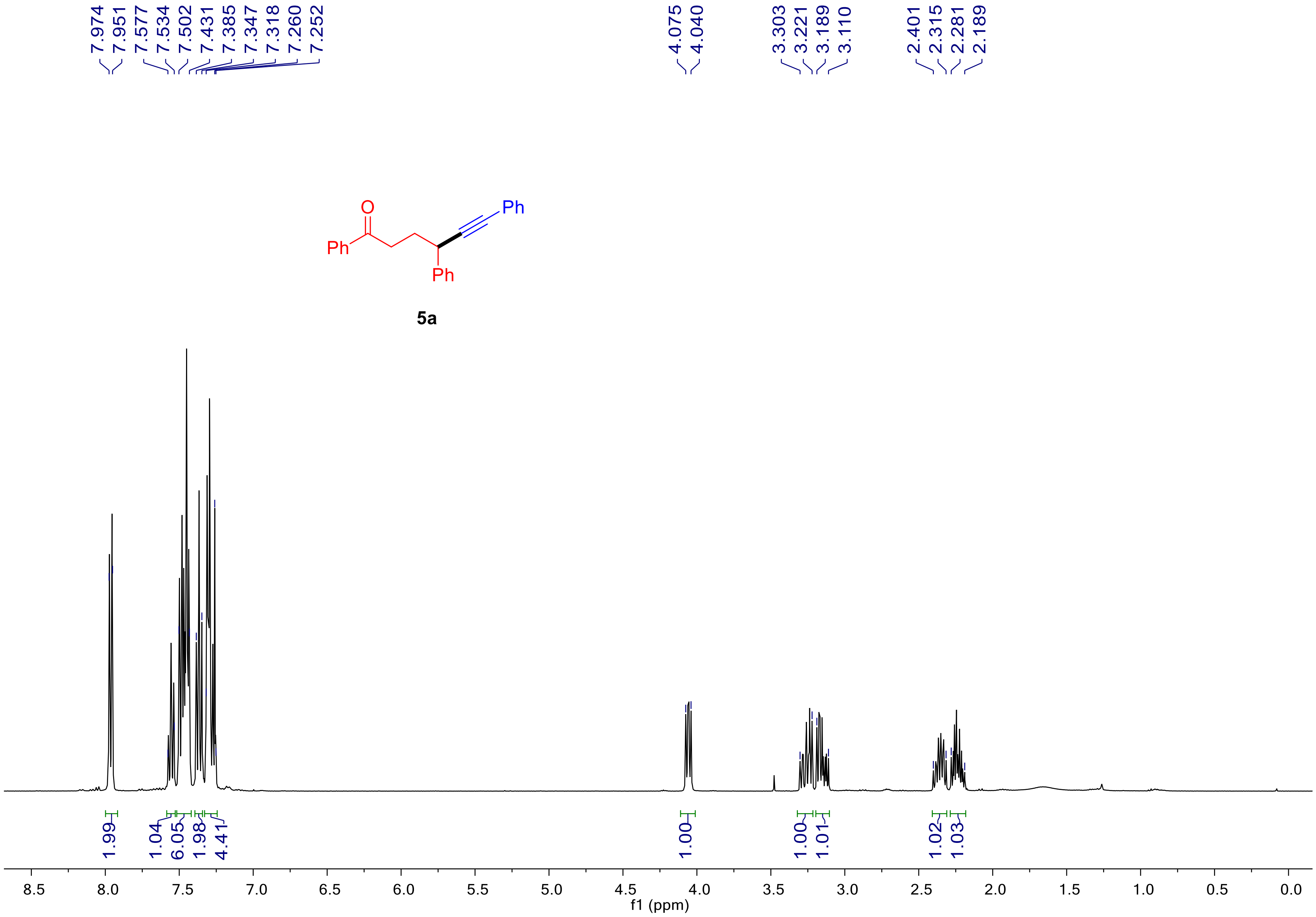

Supplementary Figure 137. ¹H NMR spectrum of 5a

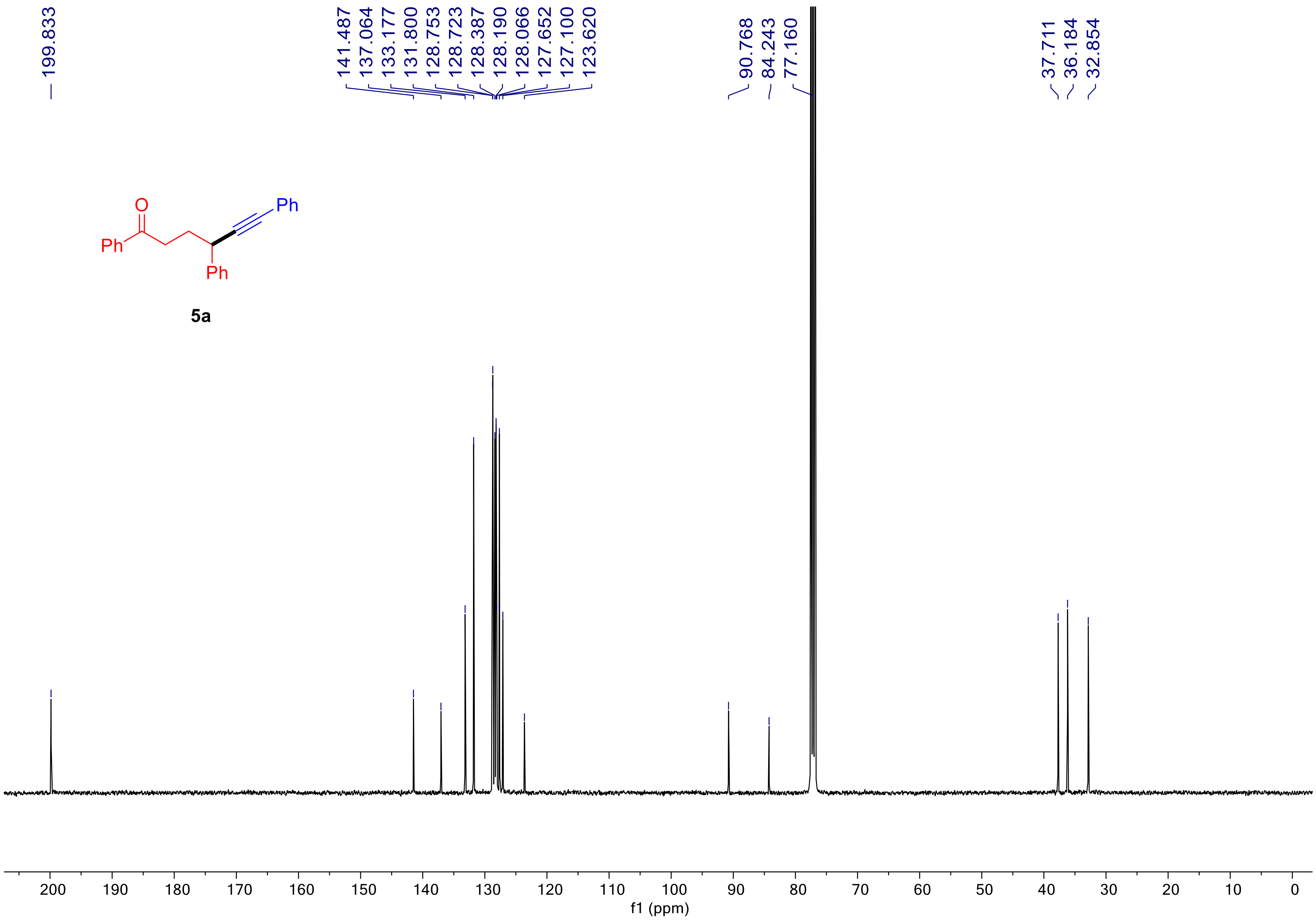

Supplementary Figure 138. ^{13}C NMR spectrum of 5a

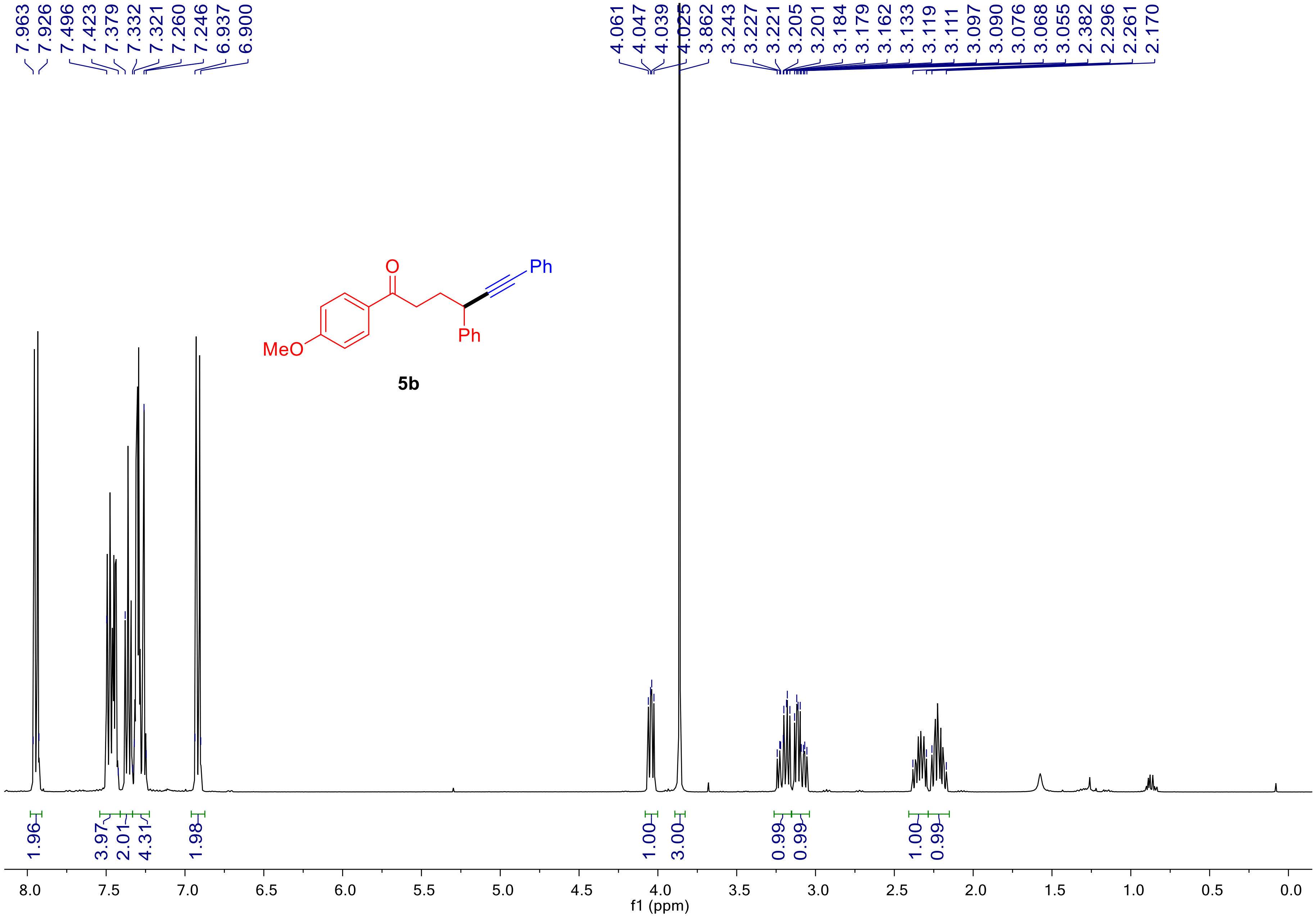

Supplementary Figure 139. ^{1}H NMR spectrum of 5b

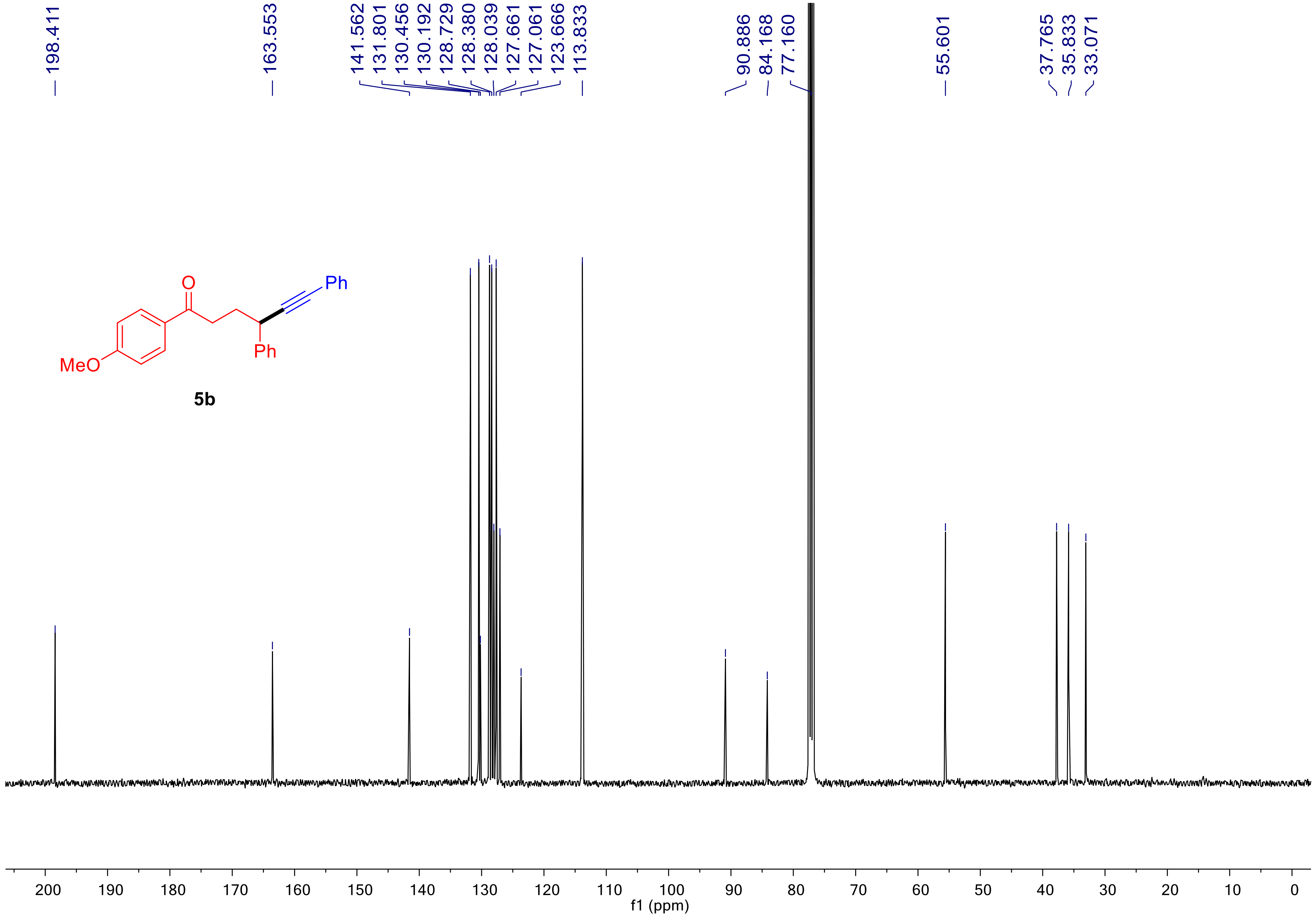

Supplementary Figure 140. ^{13}**C NMR spectrum of 5b**

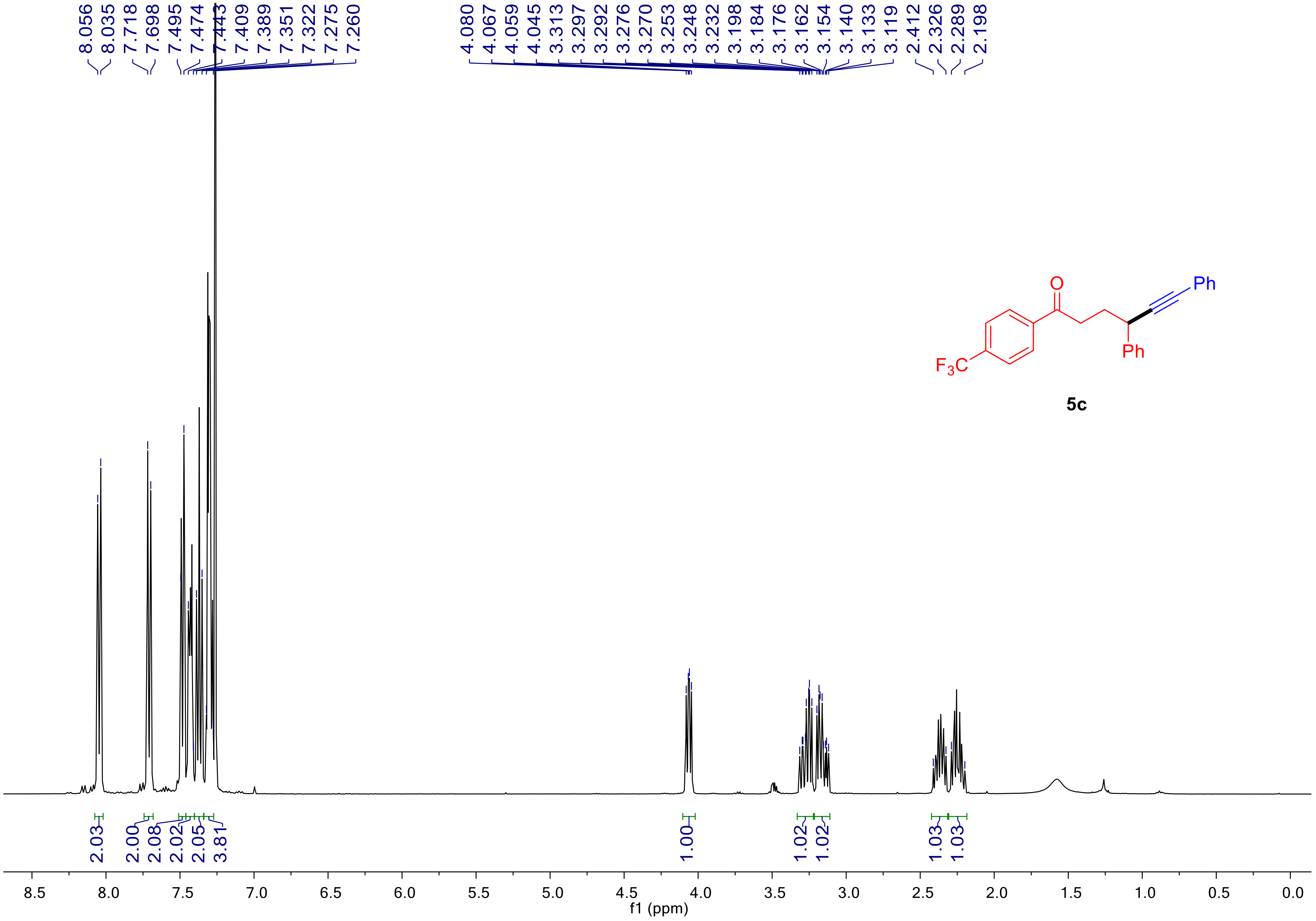

Supplementary Figure 141. ^{1}H NMR spectrum of 5c

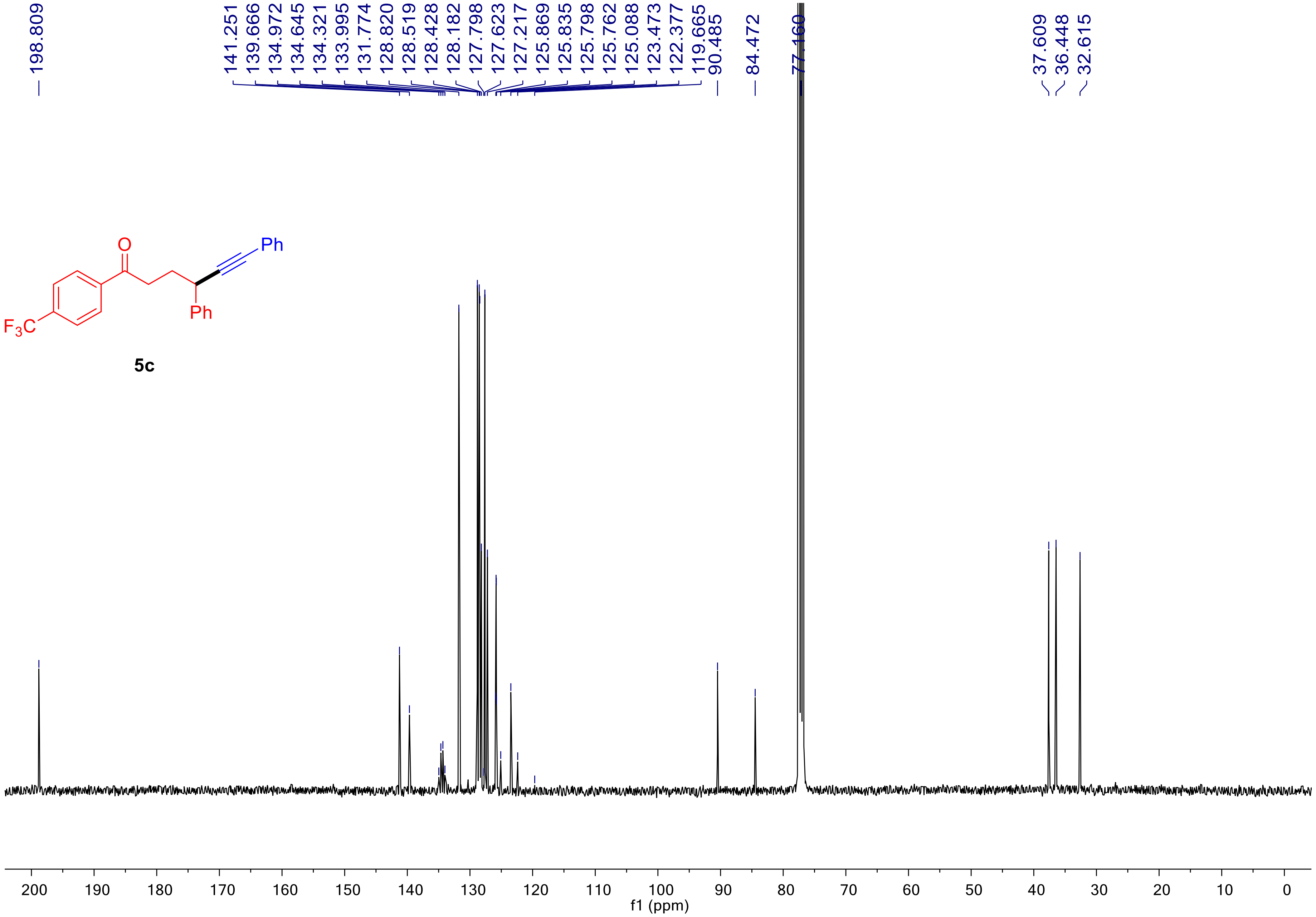

Supplementary Figure 142. ^{13}C NMR spectrum of 5c

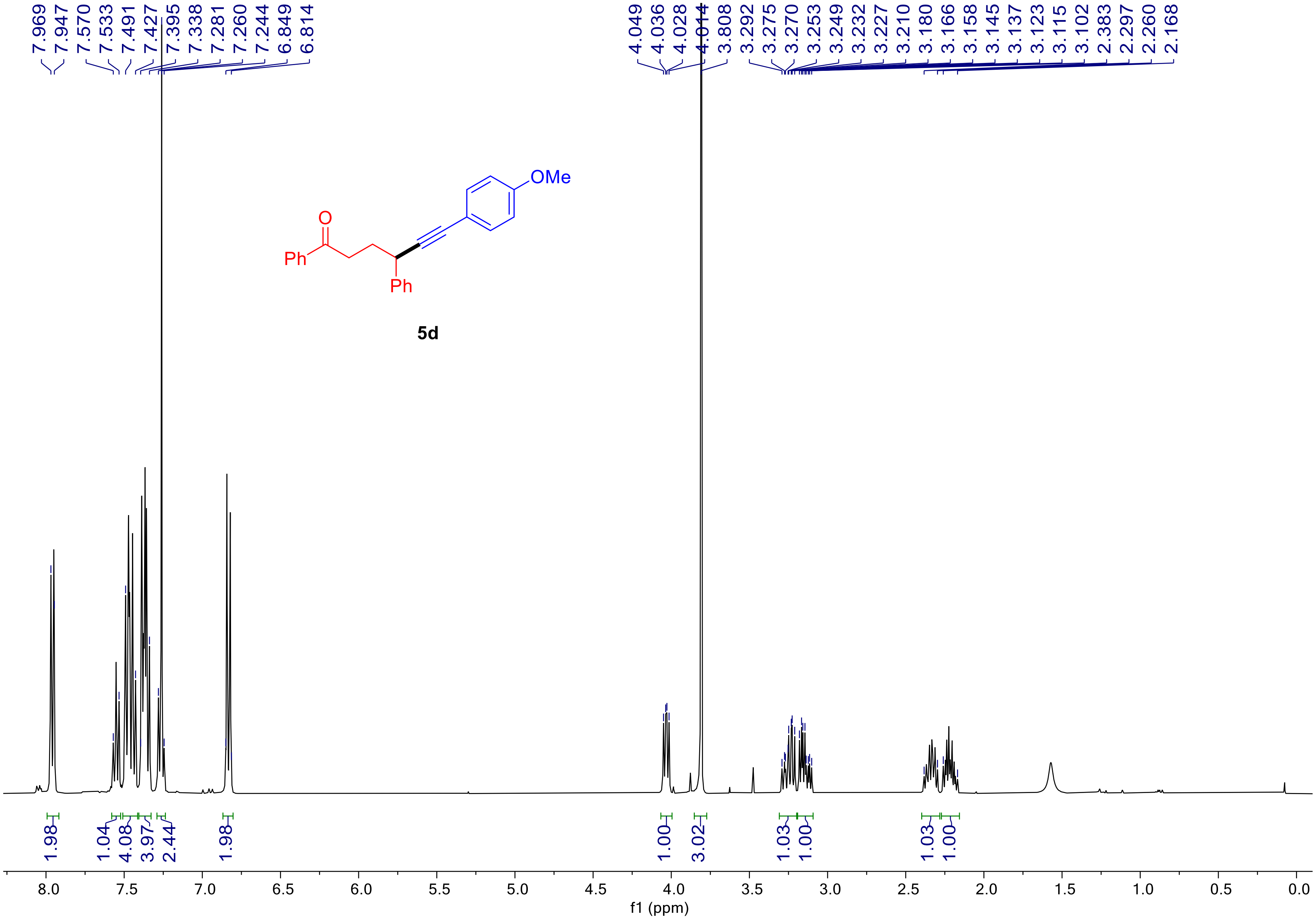

Supplementary Figure 143. ^{1}H NMR spectrum of 5d

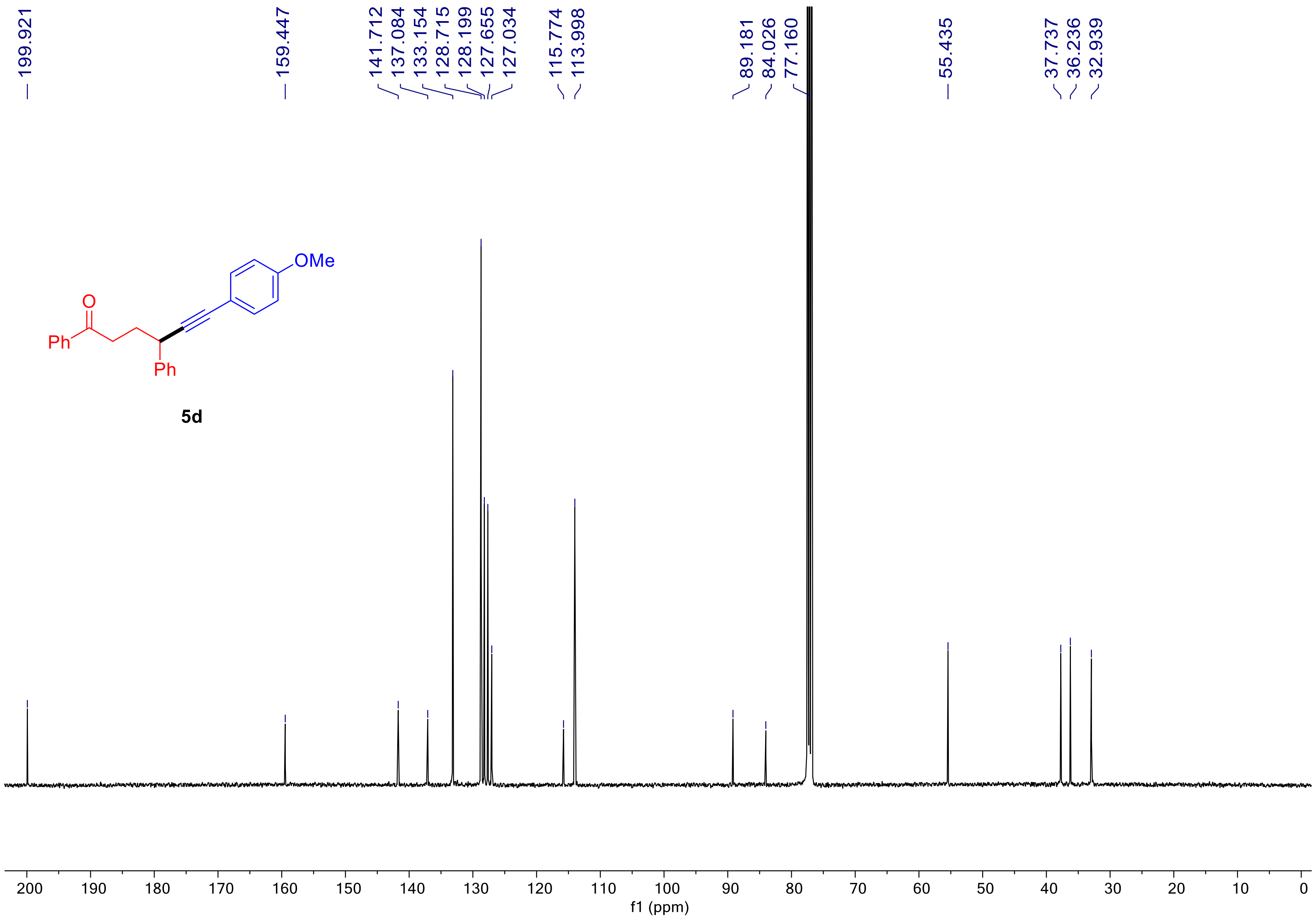

Supplementary Figure 144. ^{13}C NMR spectrum of 5d

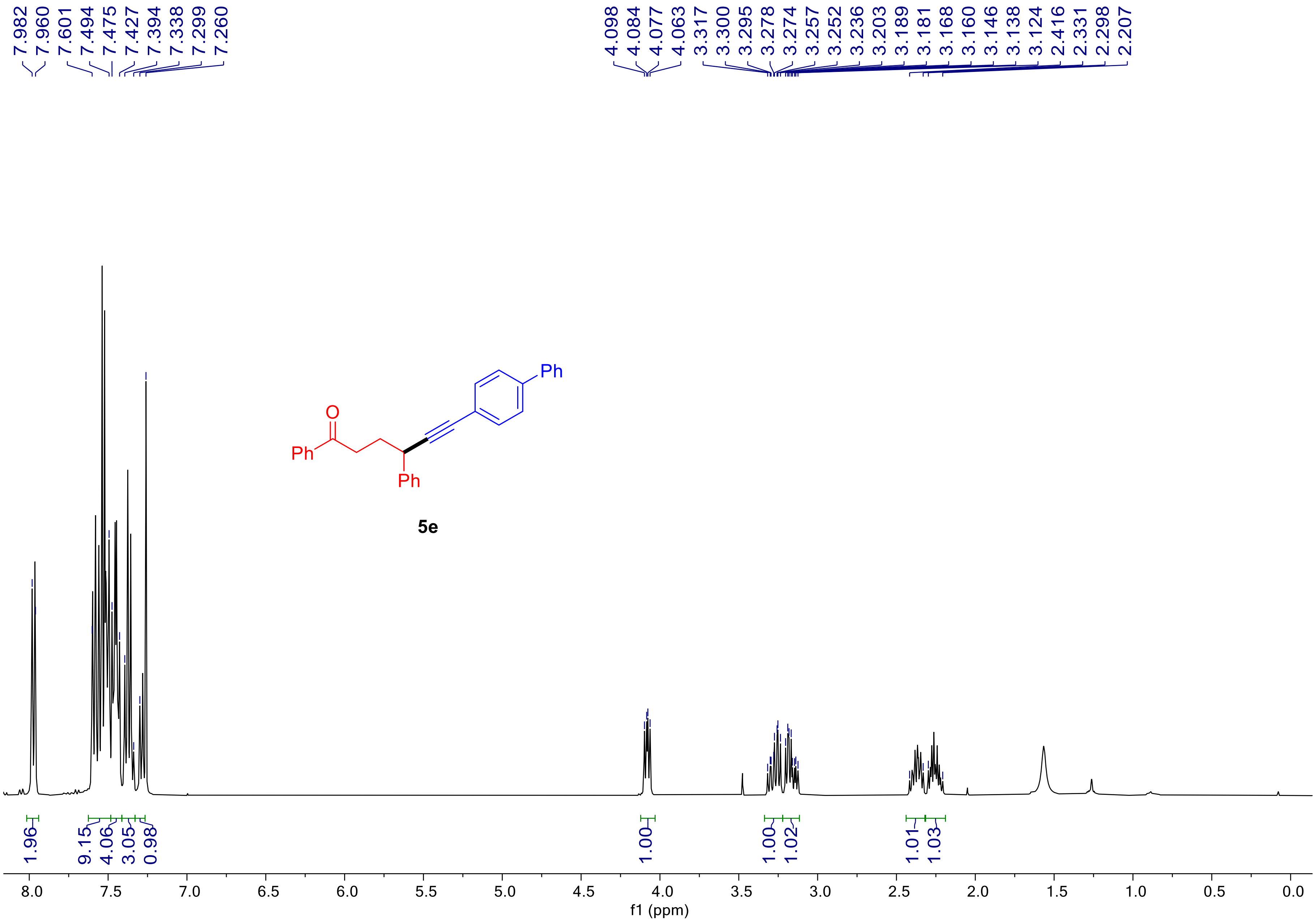

Supplementary Figure 145. ^{1}H NMR spectrum of 5e

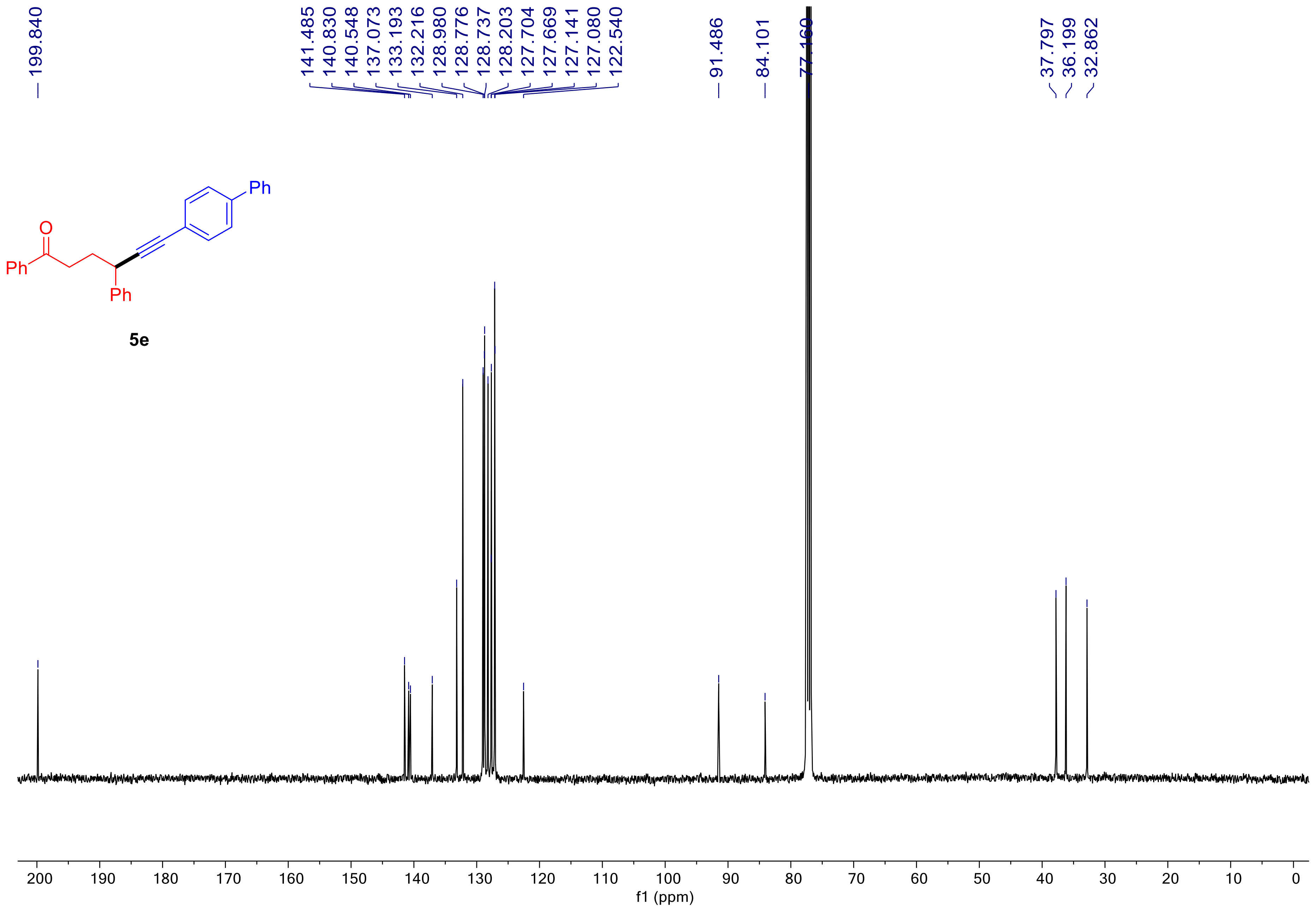

Supplementary Figure 146. ^{13}C NMR spectrum of 5e

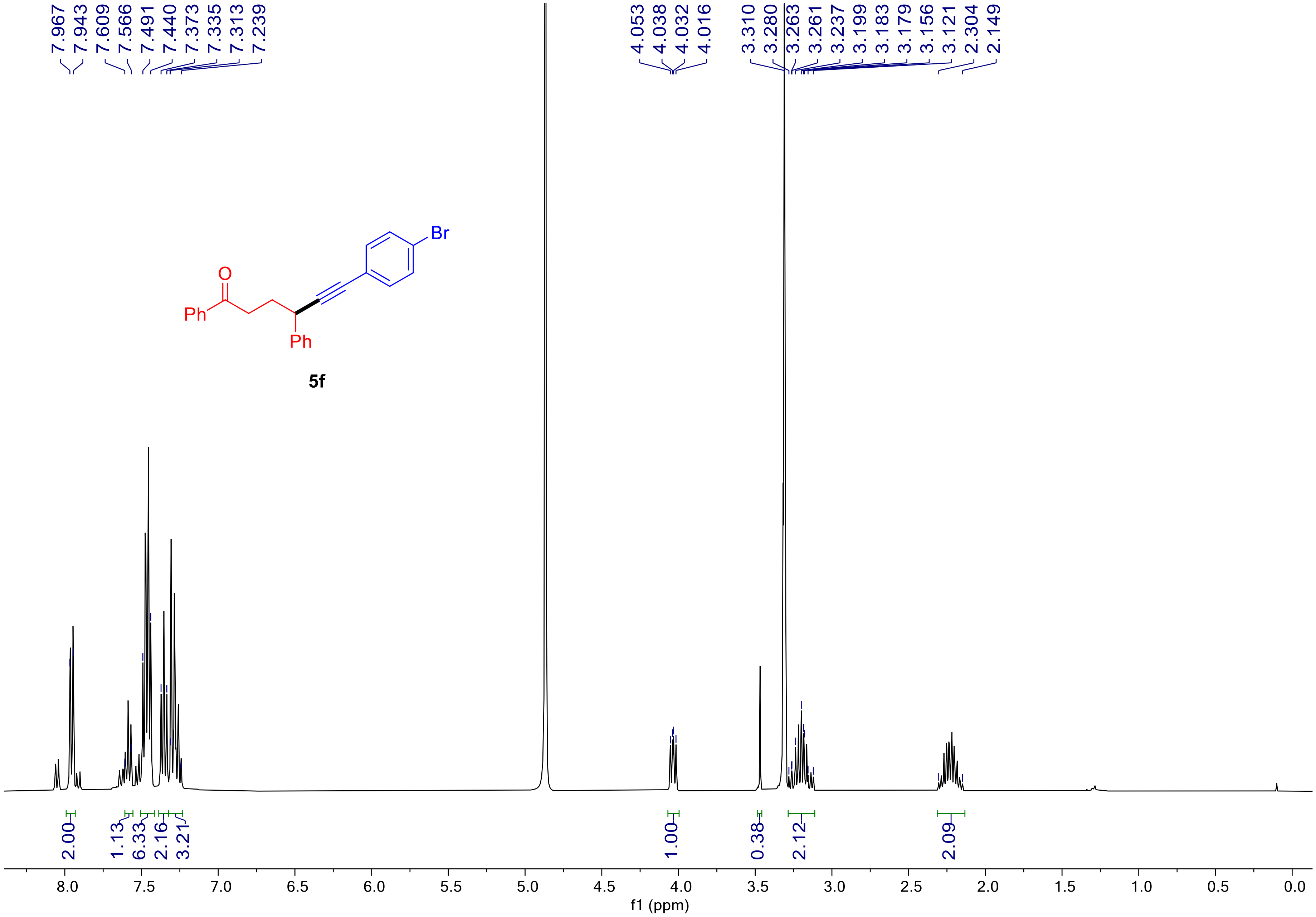

Supplementary Figure 147. ^{1}H NMR spectrum of 5f

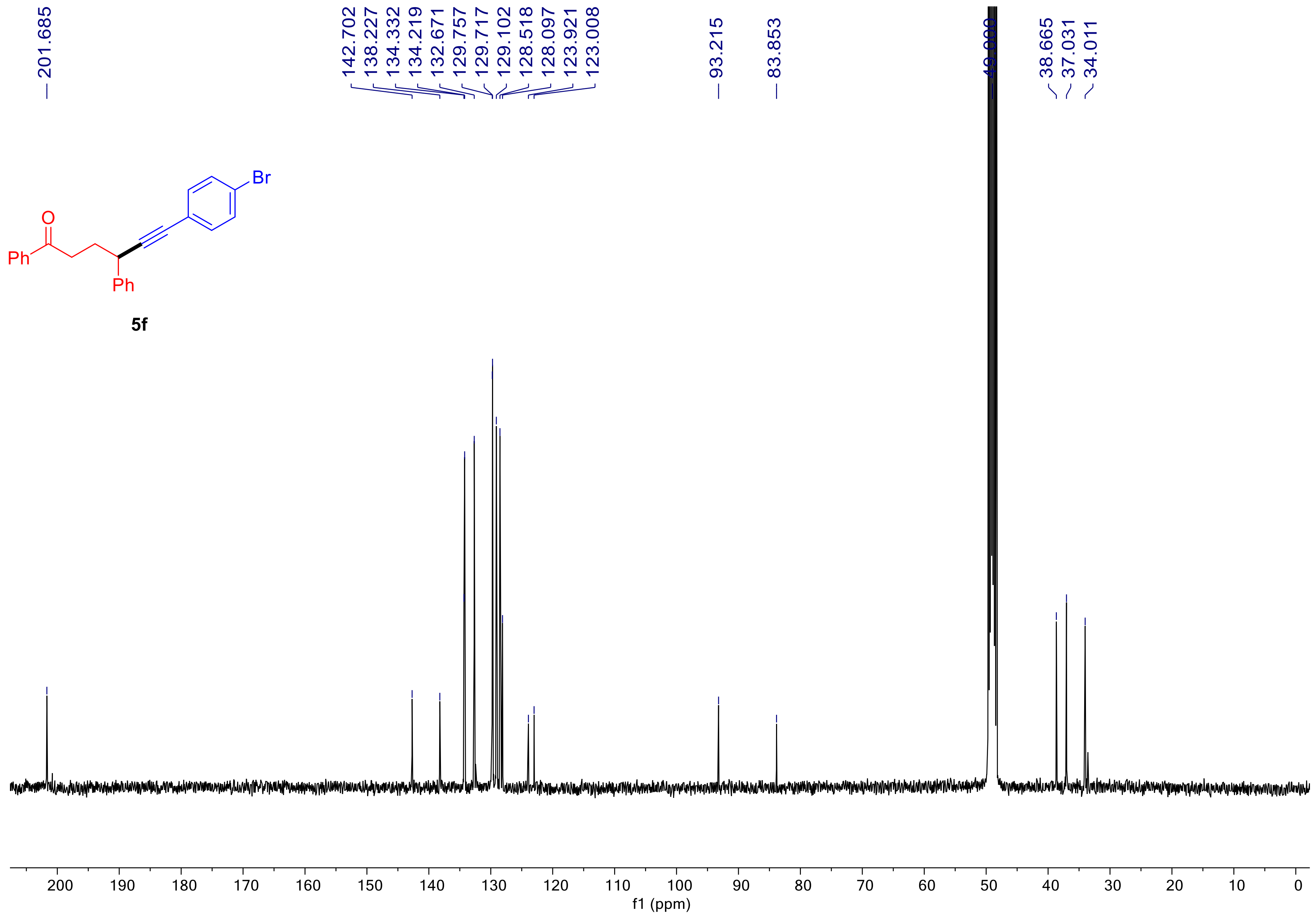

Supplementary Figure 148. ^{13}C NMR spectrum of 5f

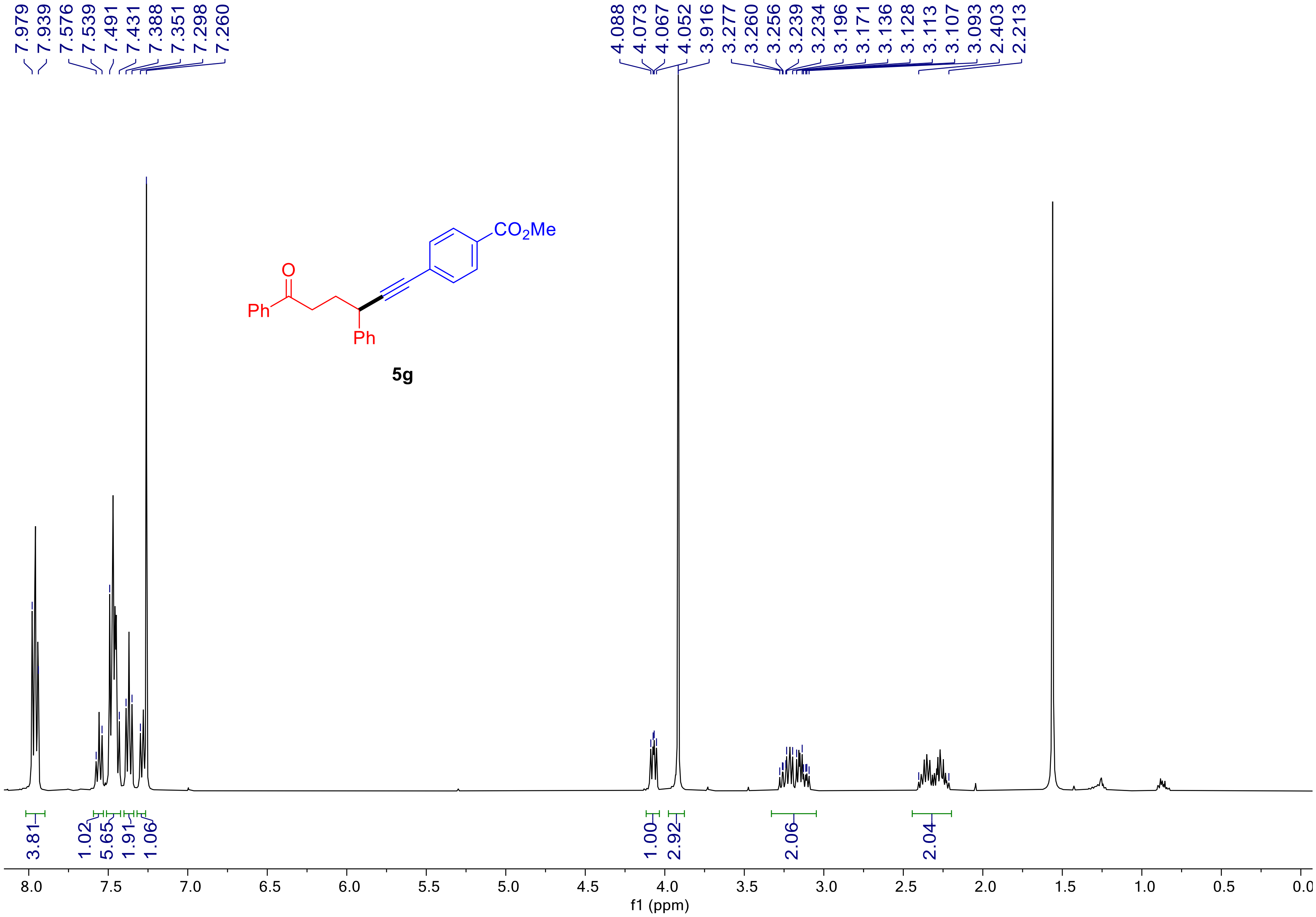

Supplementary Figure 149. ^{1}H NMR spectrum of 5g

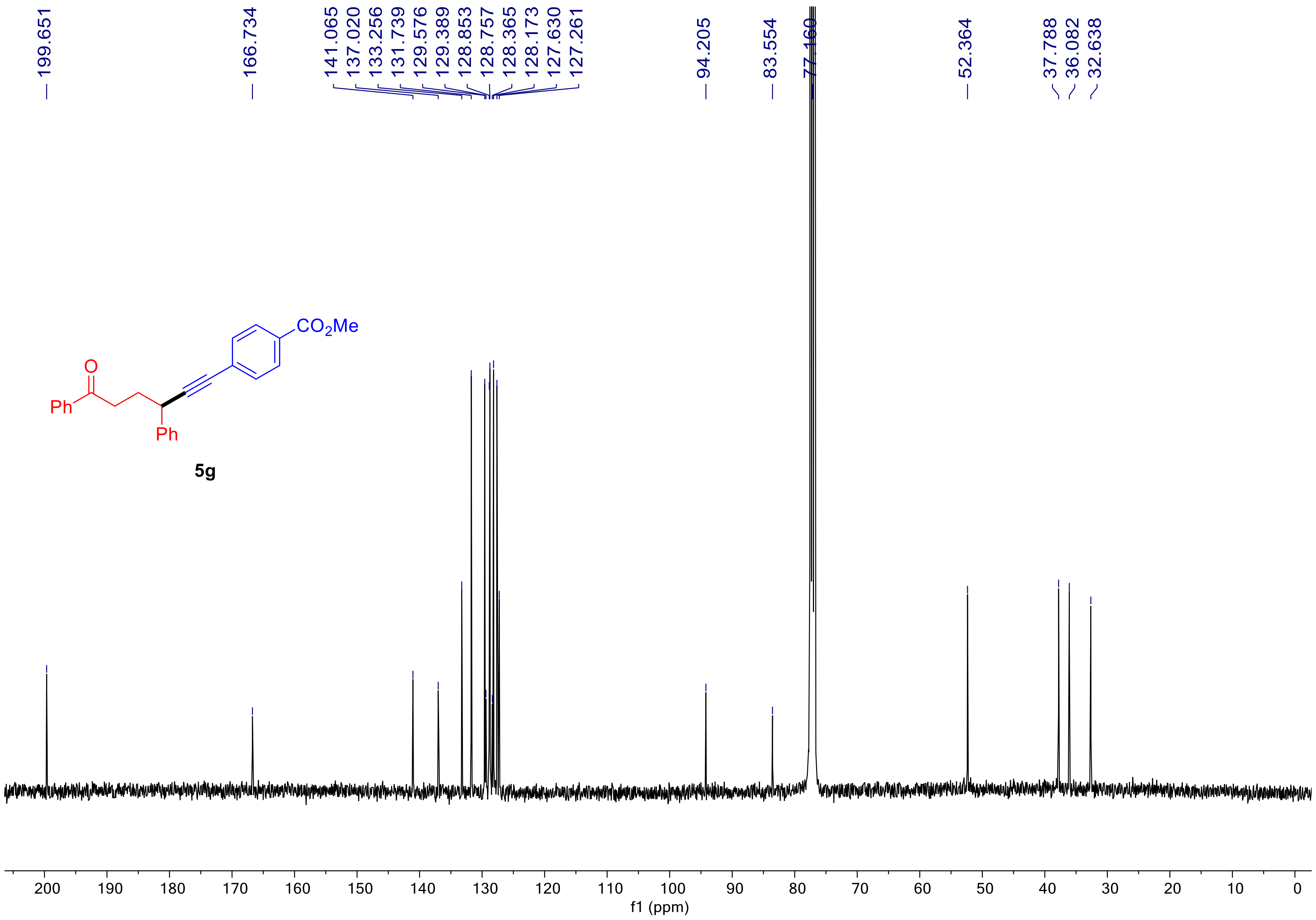

Supplementary Figure 150. ^{13}C NMR spectrum of 5g

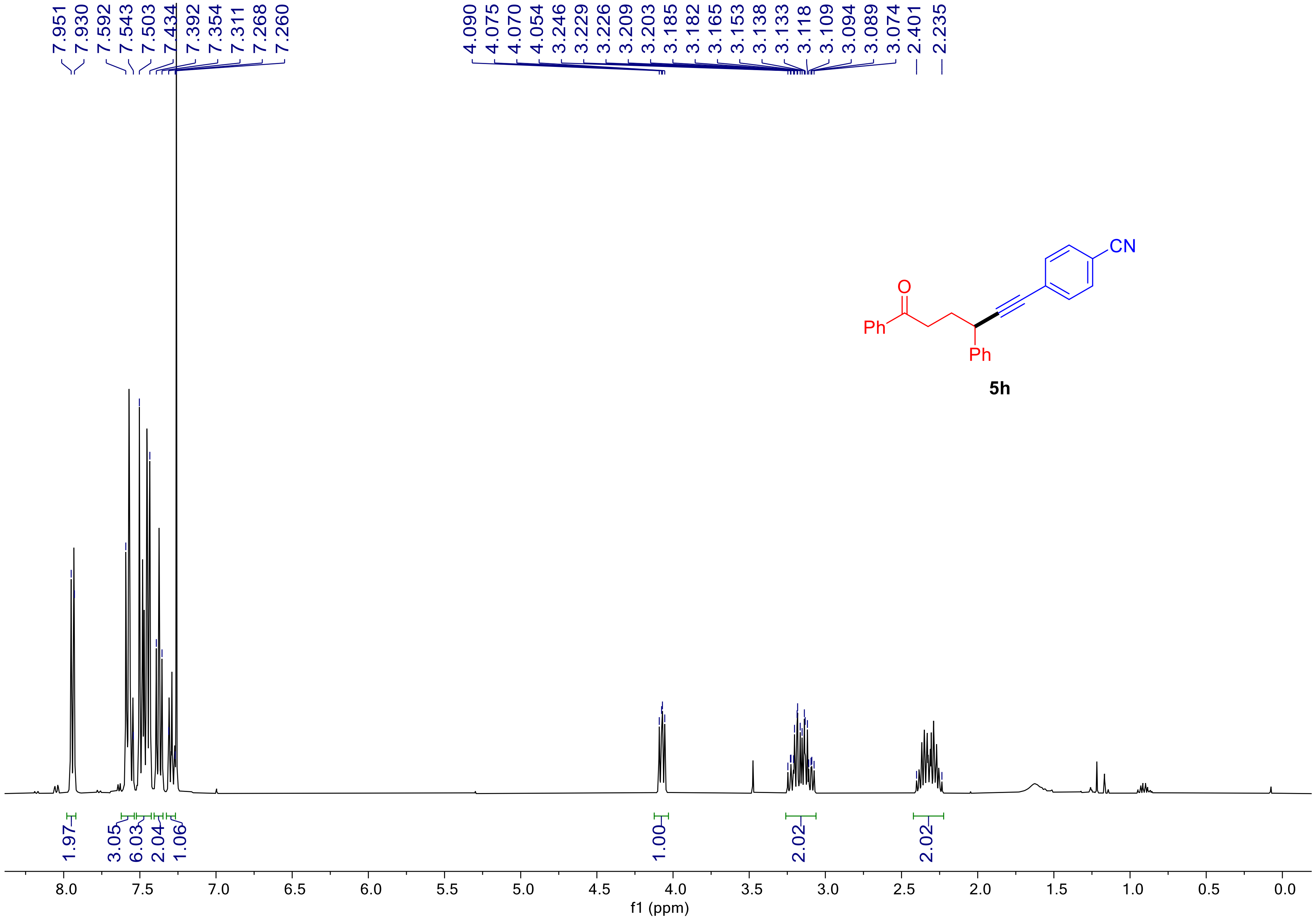

5h

Supplementary Figure 151. ^{1}H NMR spectrum of 5h

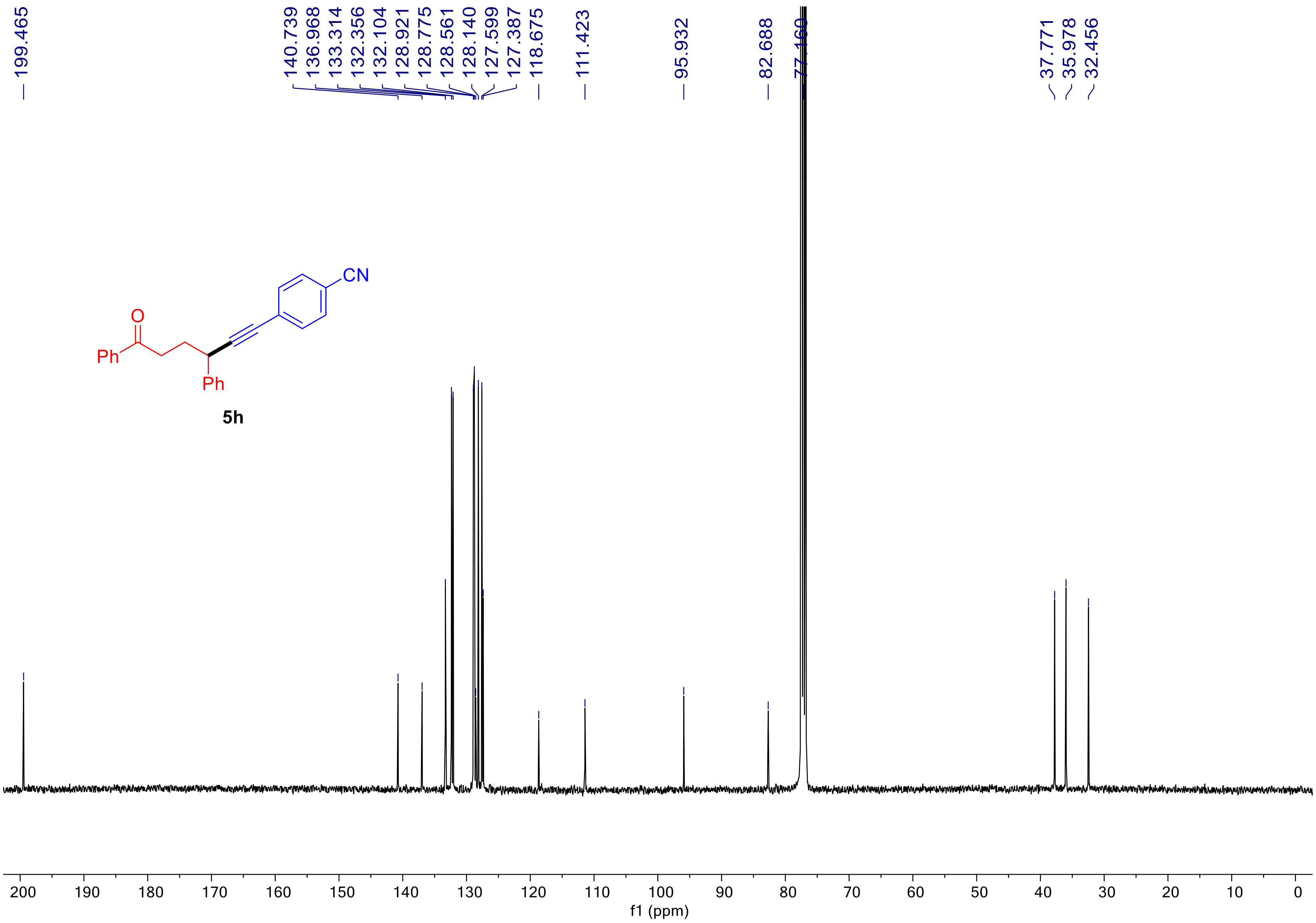

Supplementary Figure 152. ^{13}C NMR spectrum of 5h

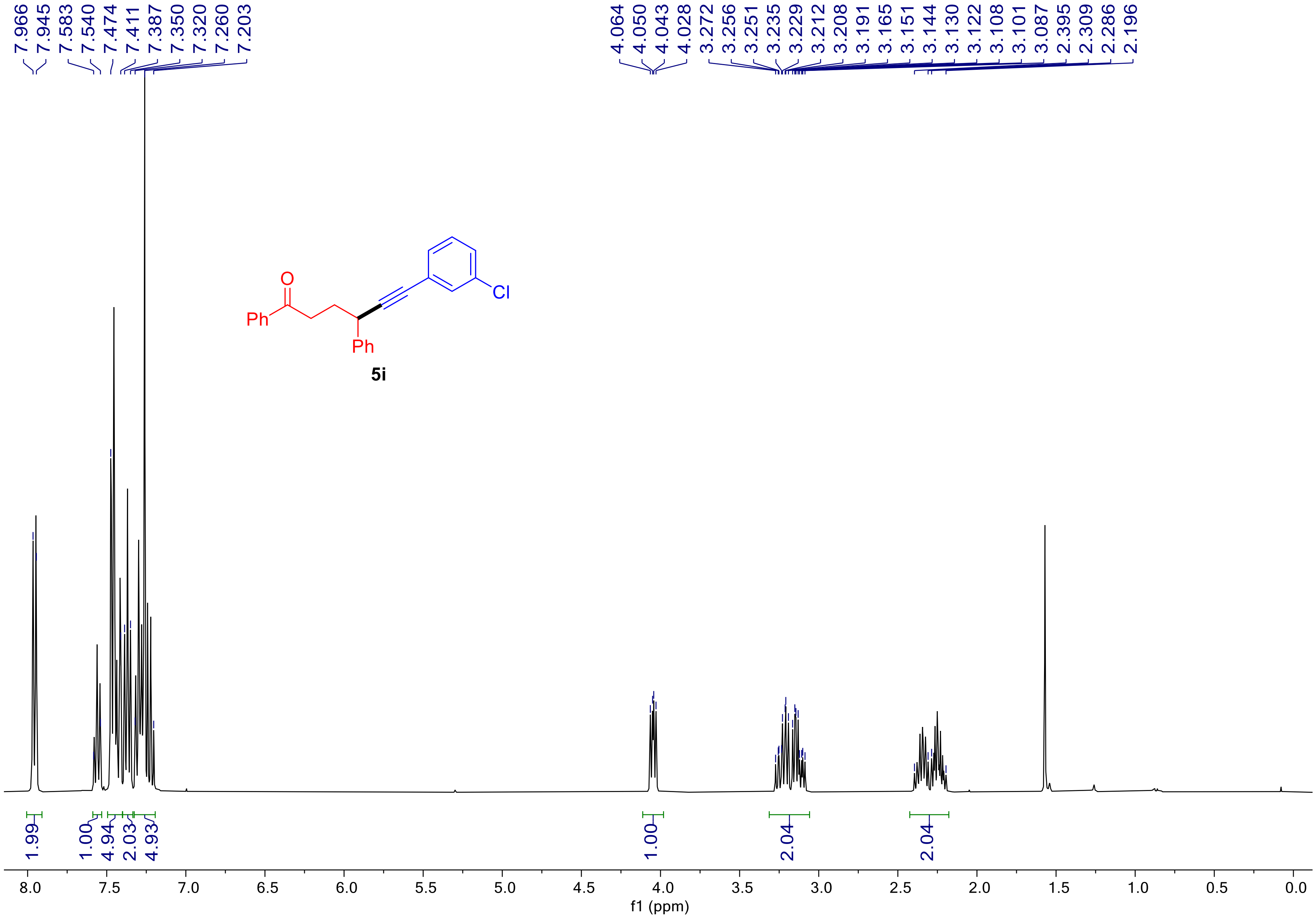

Supplementary Figure 153. ^{1}H NMR spectrum of 5i

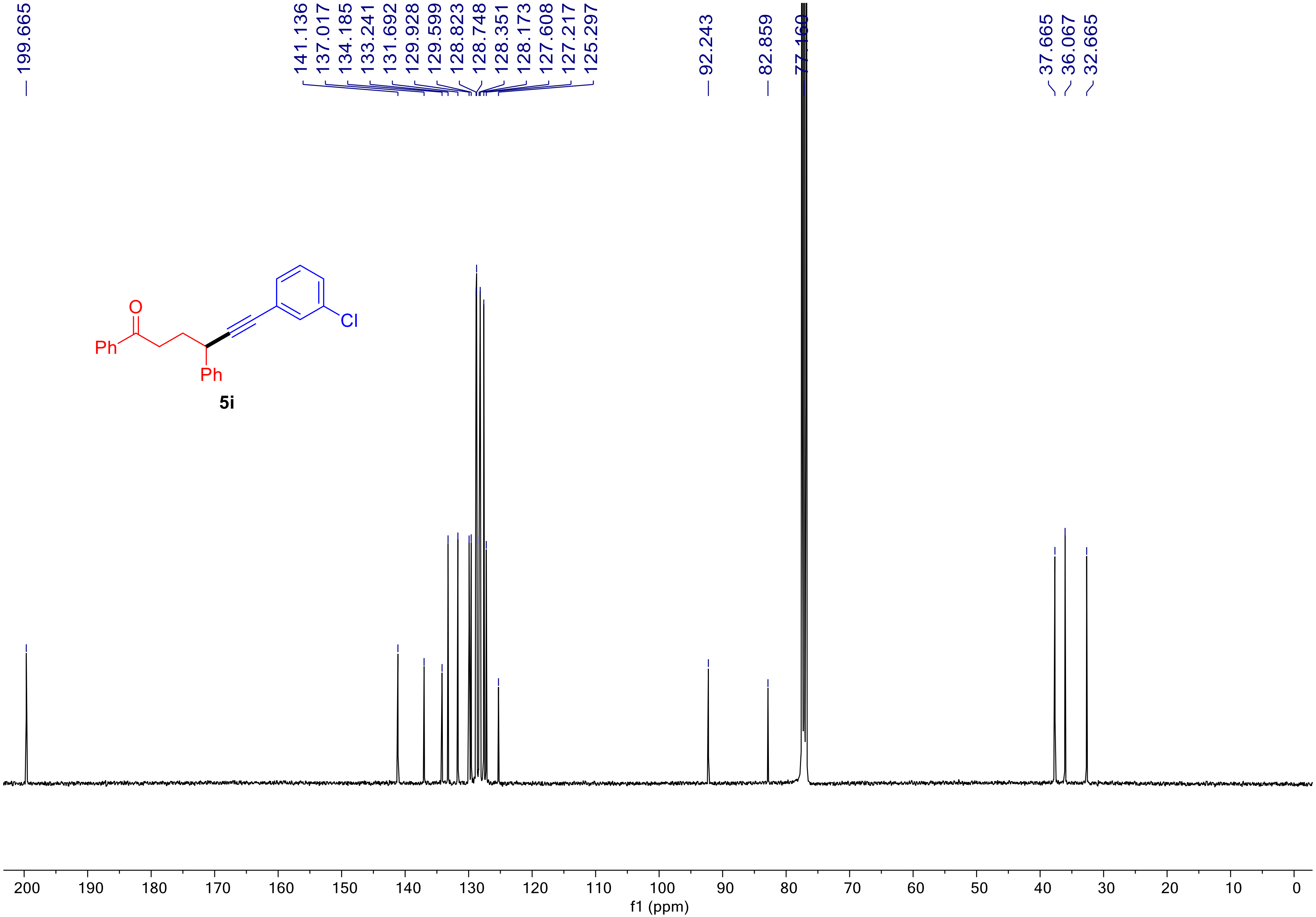

Supplementary Figure 154. ^{13}C NMR spectrum of 5i

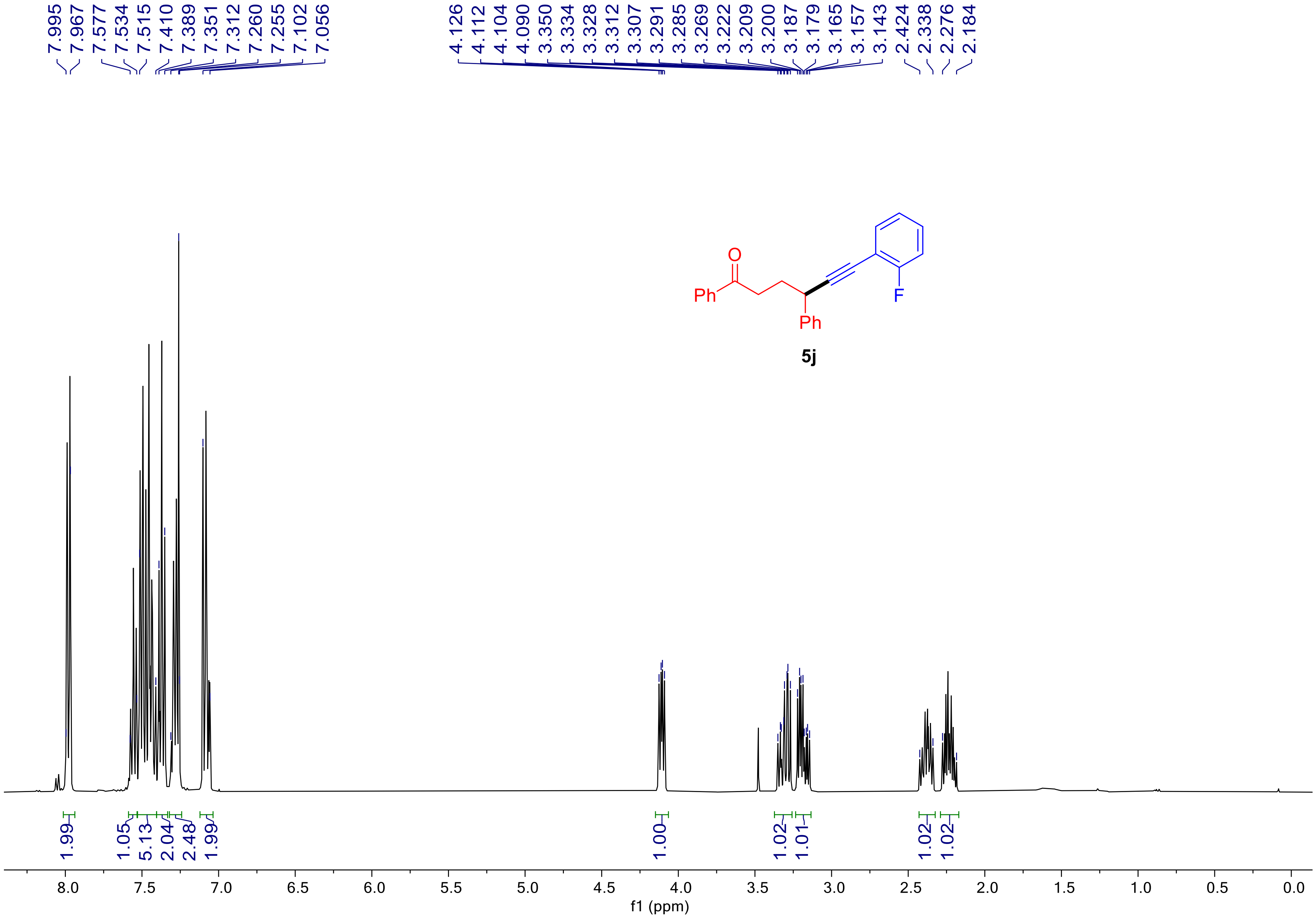

Supplementary Figure 155. ^{1}H NMR spectrum of 5j

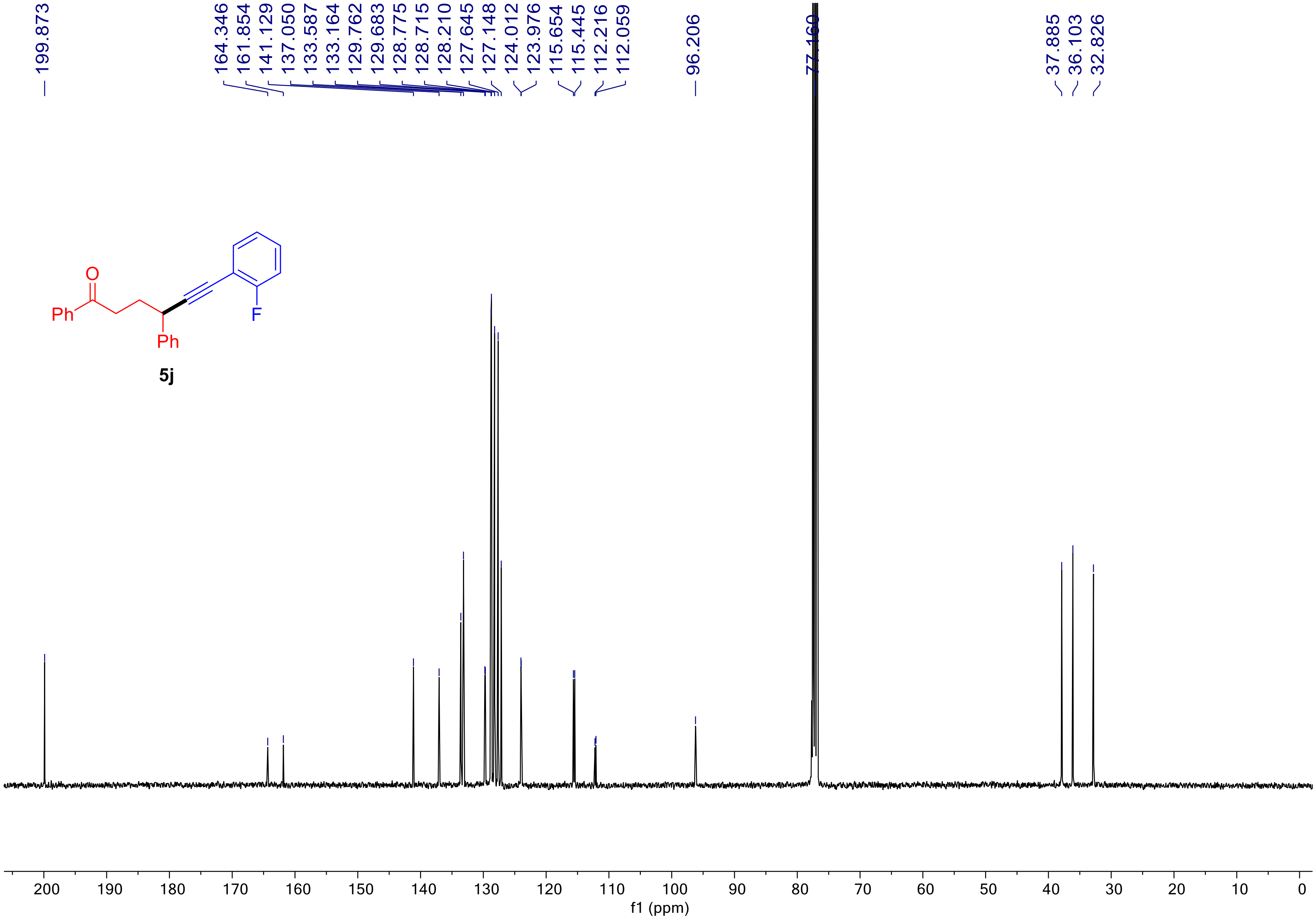

Supplementary Figure 156. ^{13}C NMR spectrum of 5j

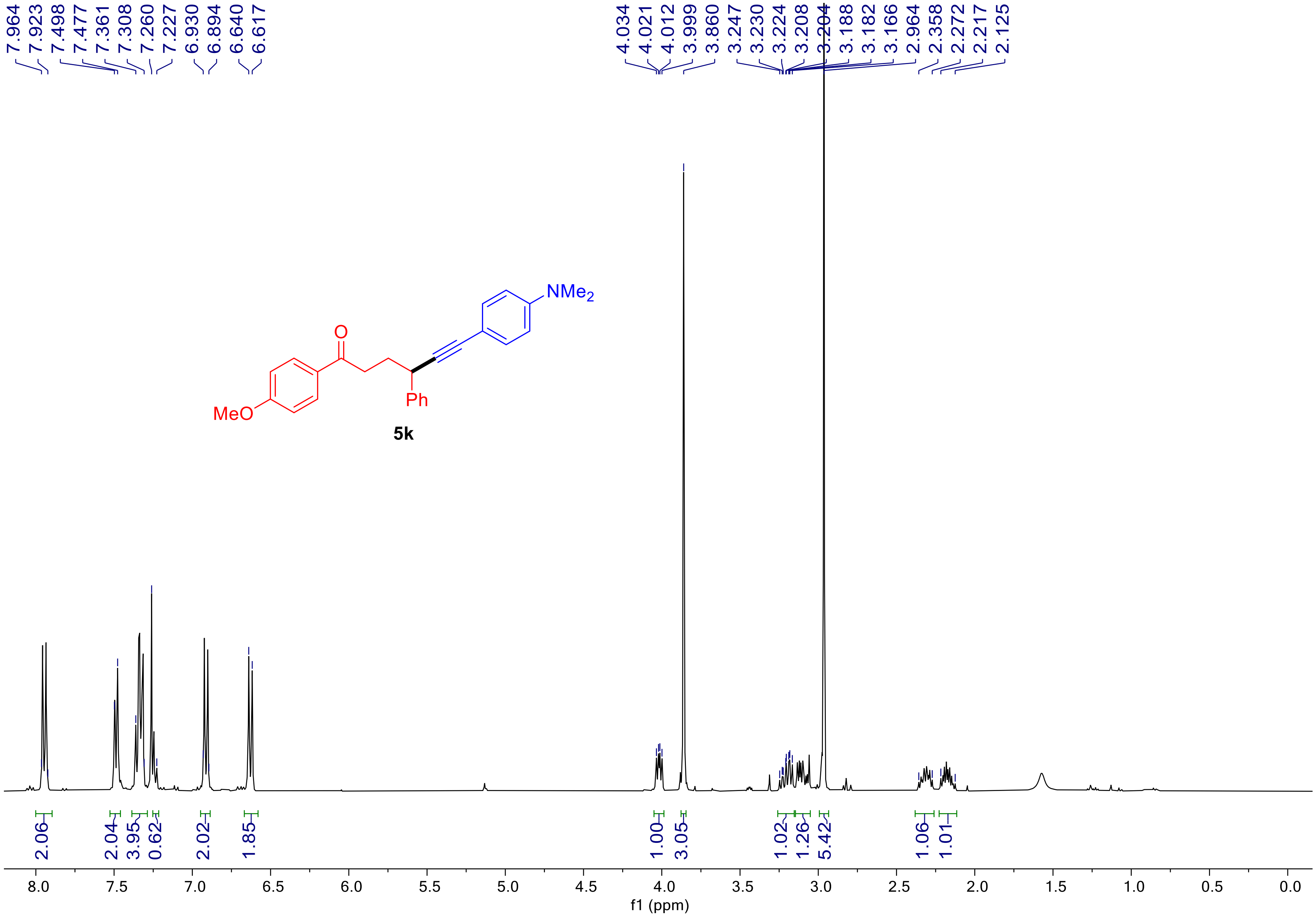

Supplementary Figure 157. ^{1}H NMR spectrum of 5k

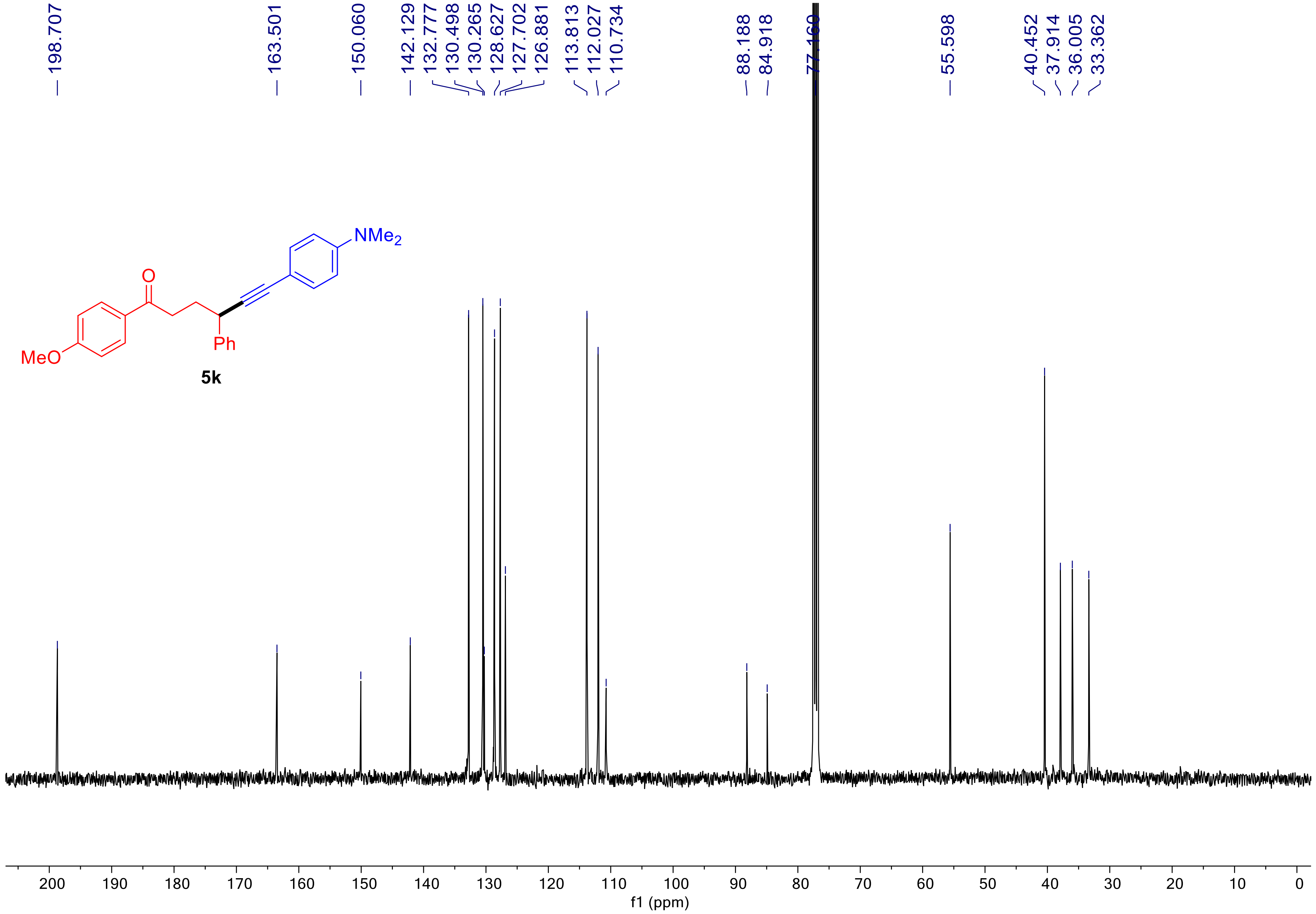

Supplementary Figure 158. ^{13}C NMR spectrum of 5k

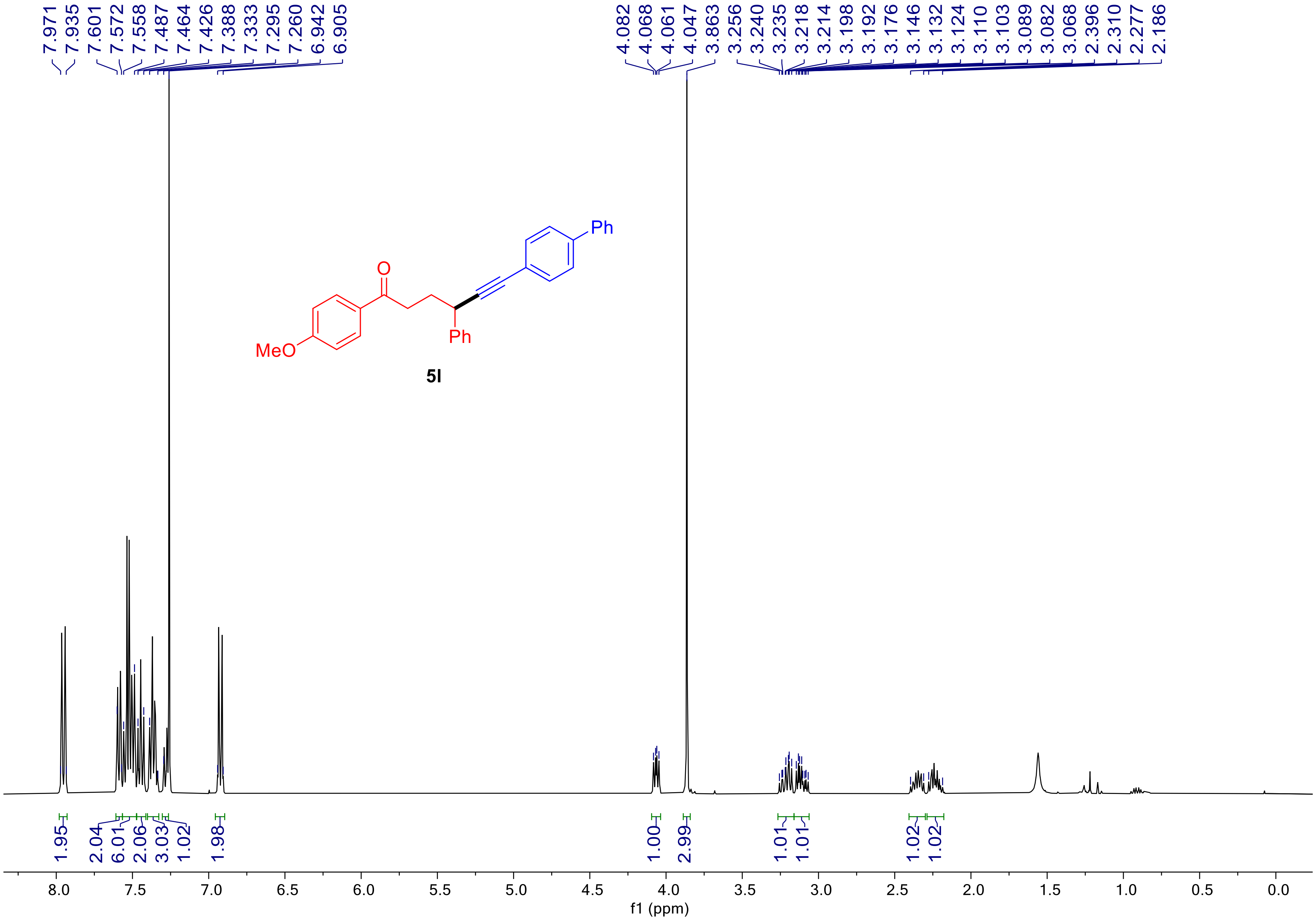

Supplementary Figure 159. ^{1}H NMR spectrum of 5l

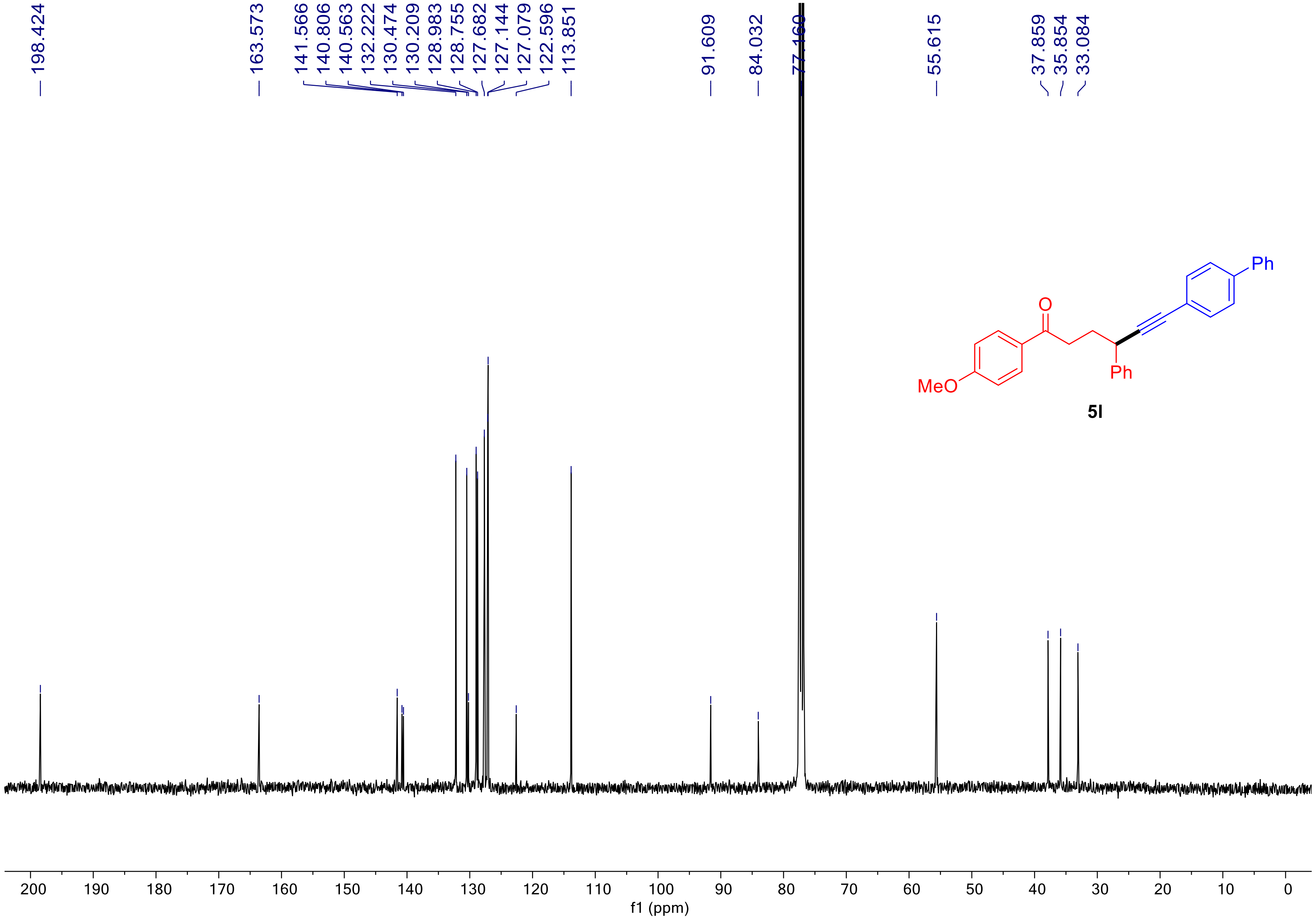

Supplementary Figure 160. ^{13}C NMR spectrum of 5l

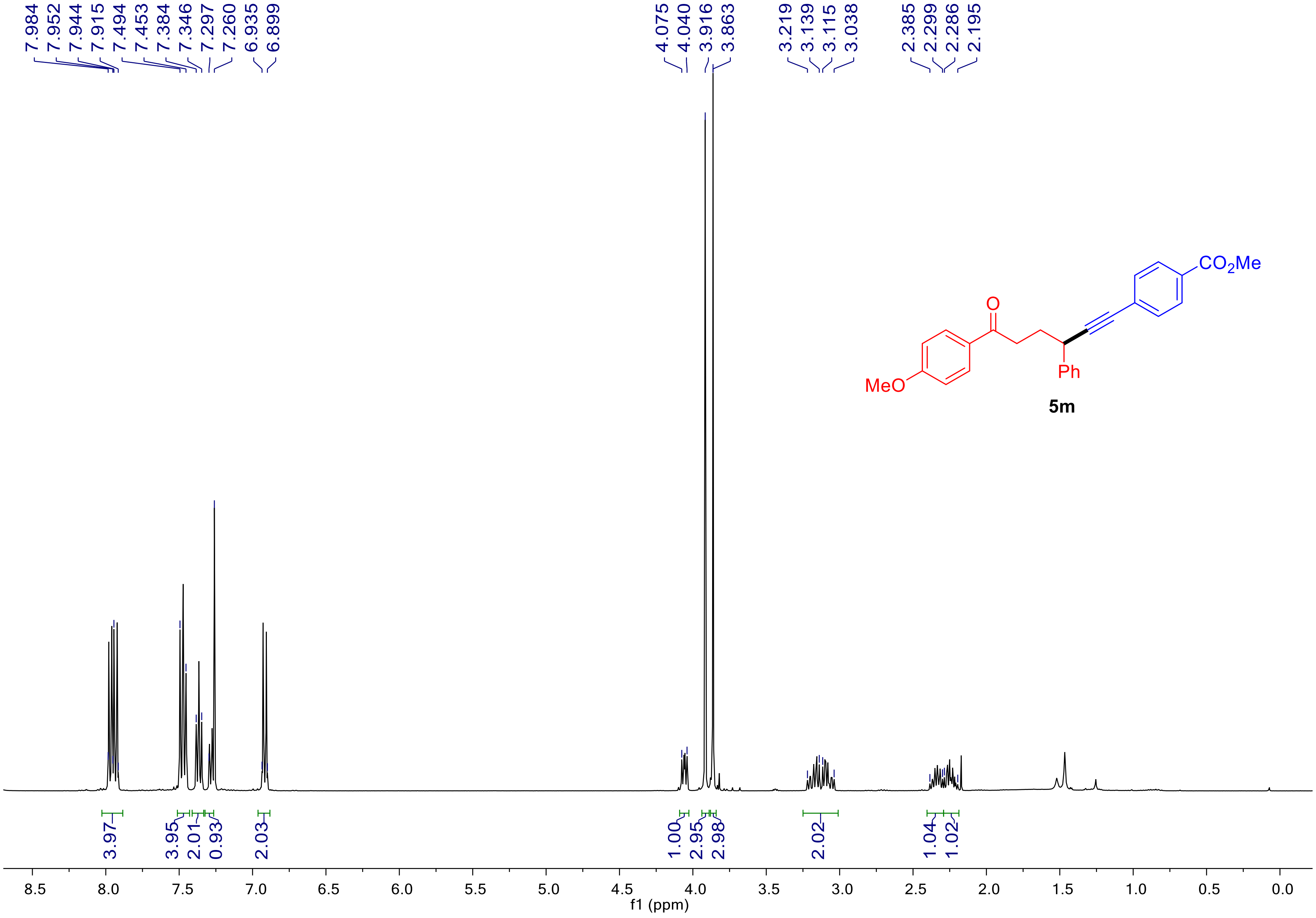

Supplementary Figure 161. ^{1}H NMR spectrum of 5m

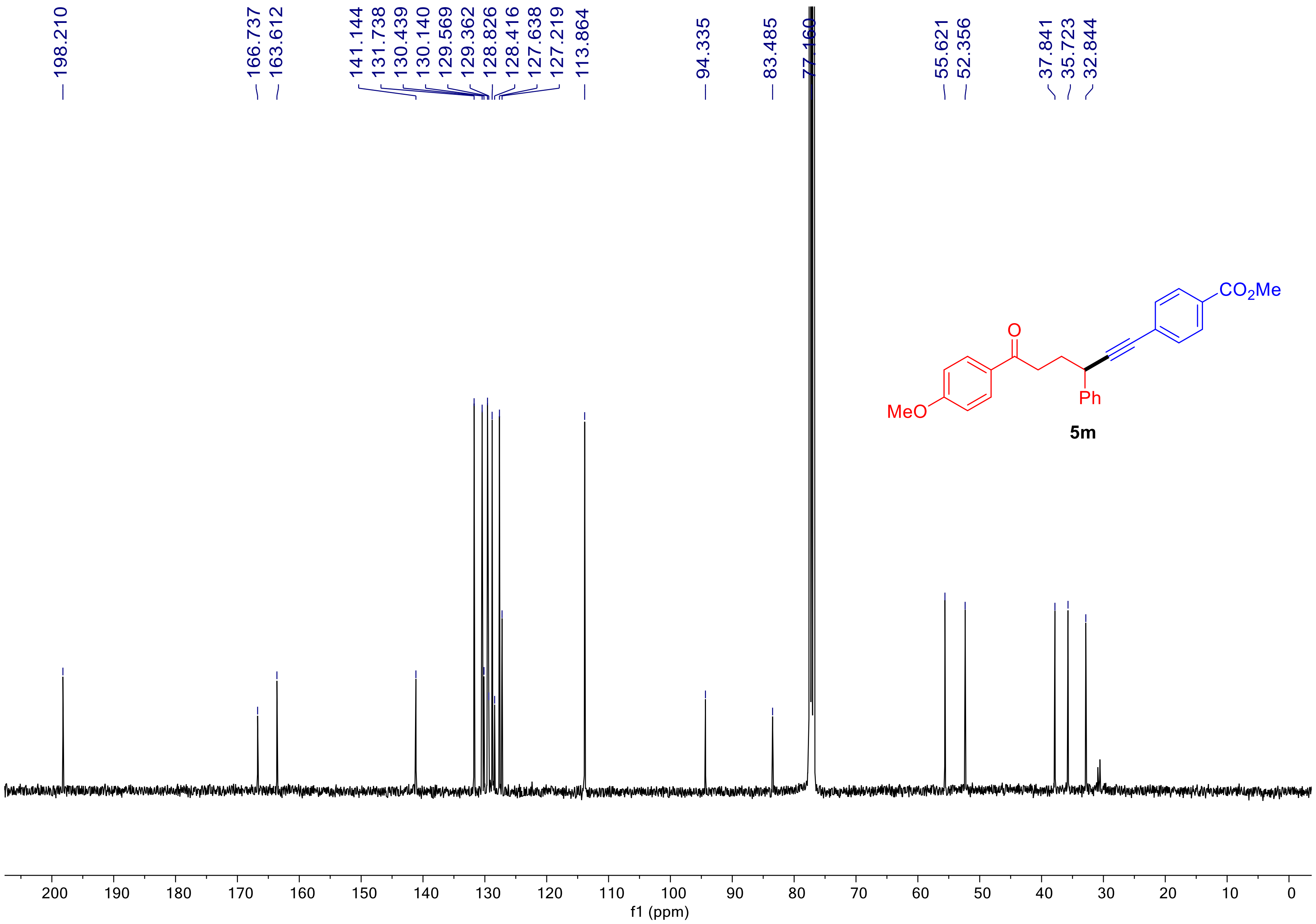

Supplementary Figure 162. ^{13}C NMR spectrum of 5m

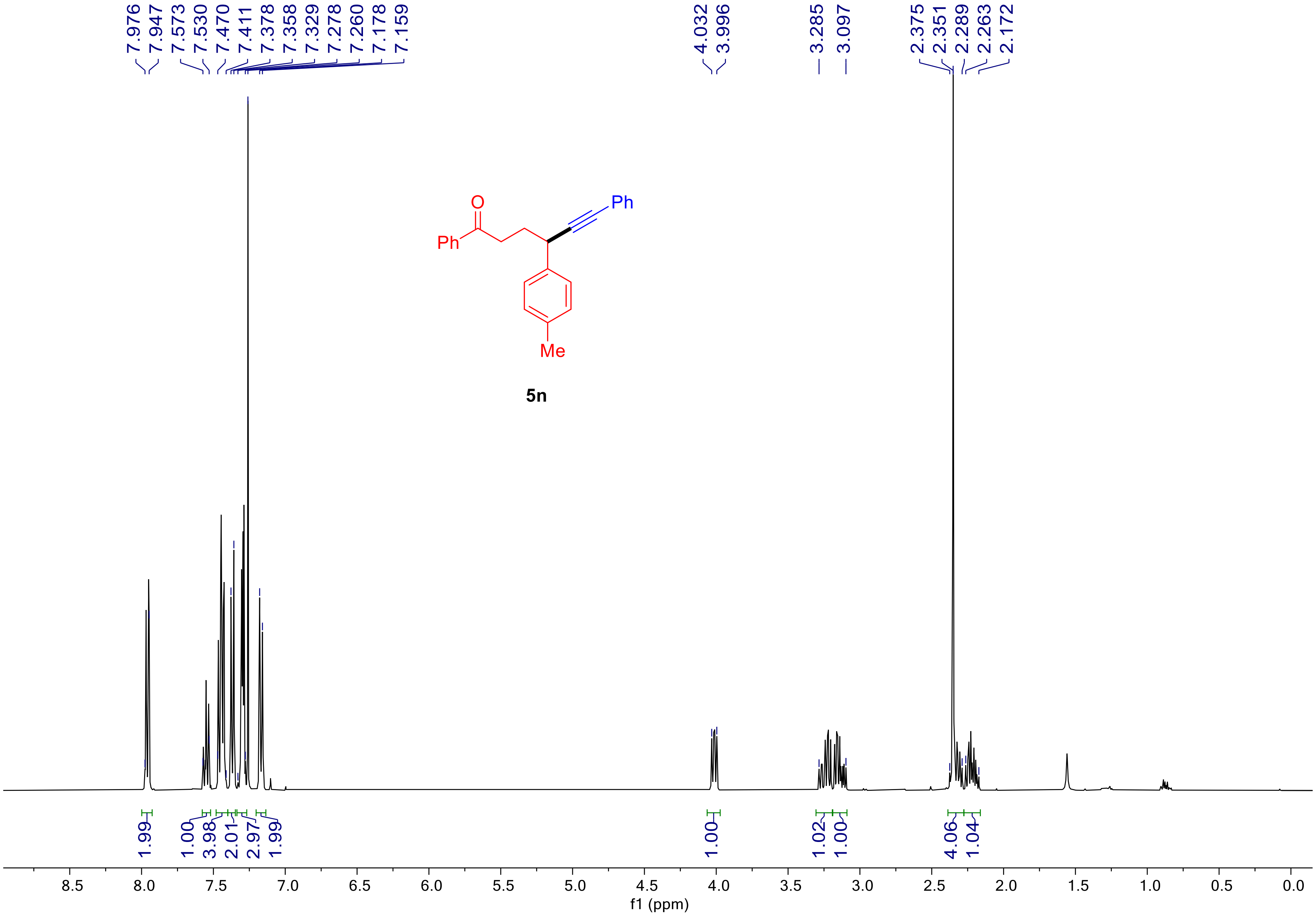

Supplementary Figure 163. ^{1}H NMR spectrum of 5n

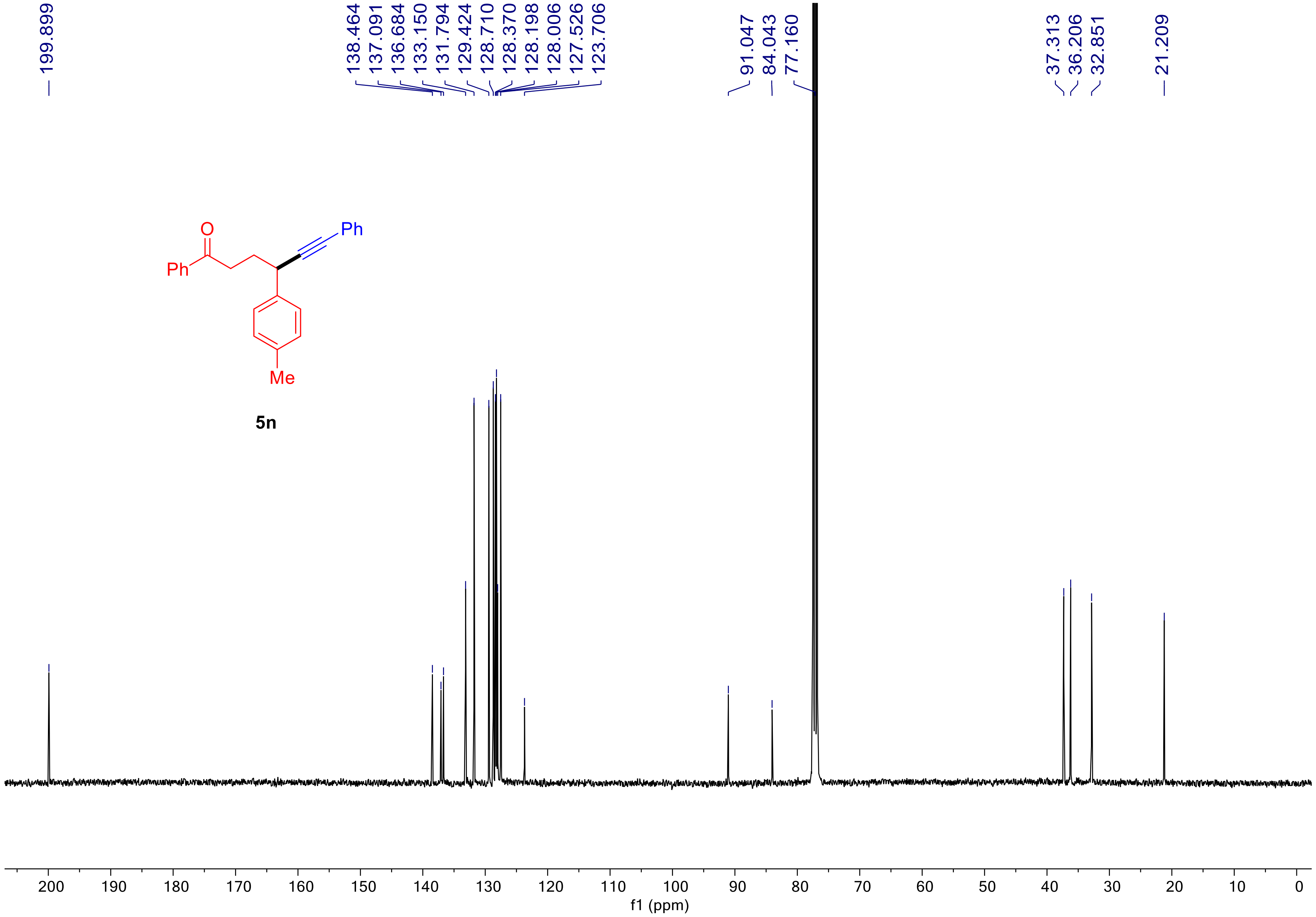

Supplementary Figure 164. ^{13}**C NMR spectrum of 5n**

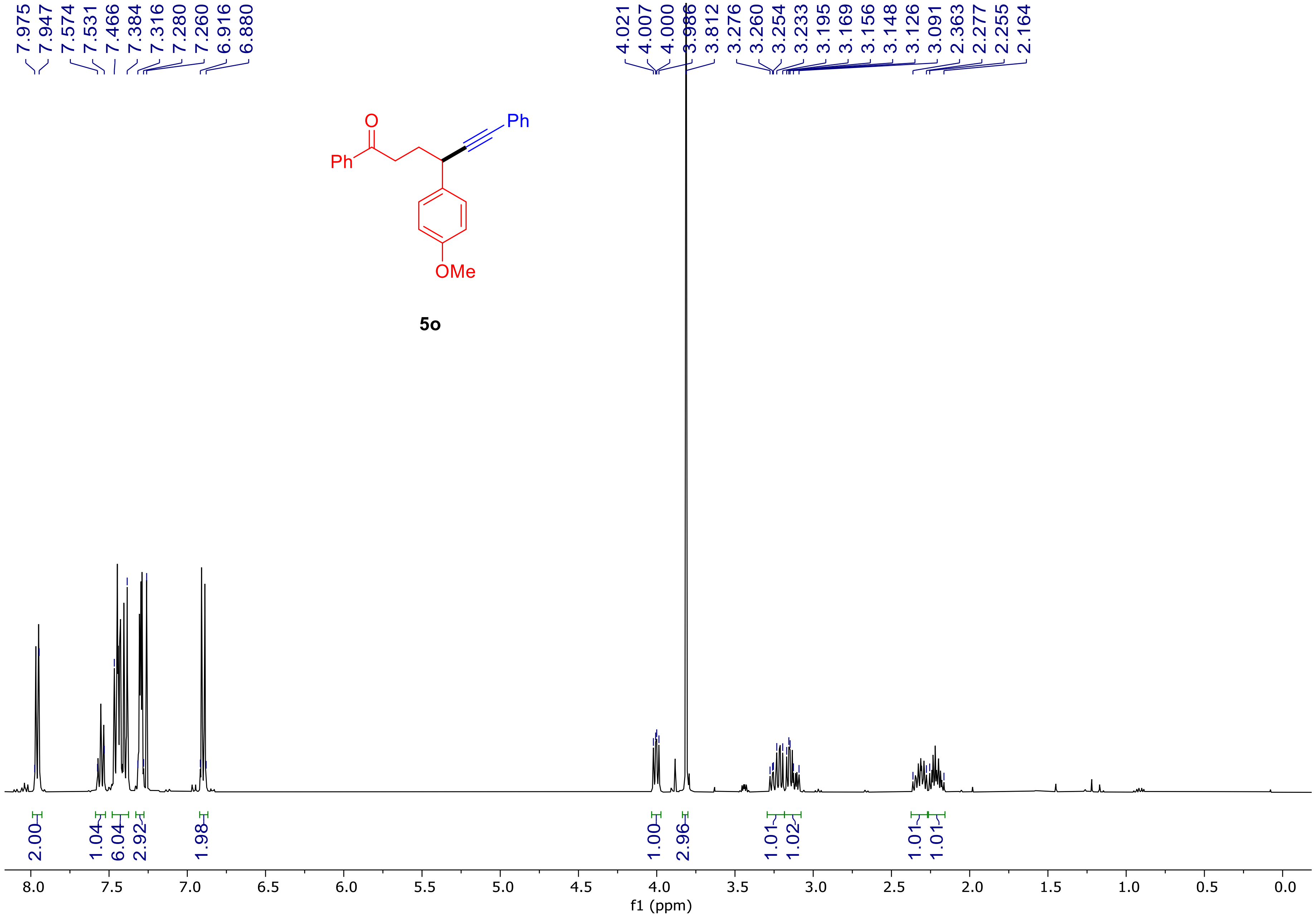

Supplementary Figure 165. ^{1}H NMR spectrum of 5o

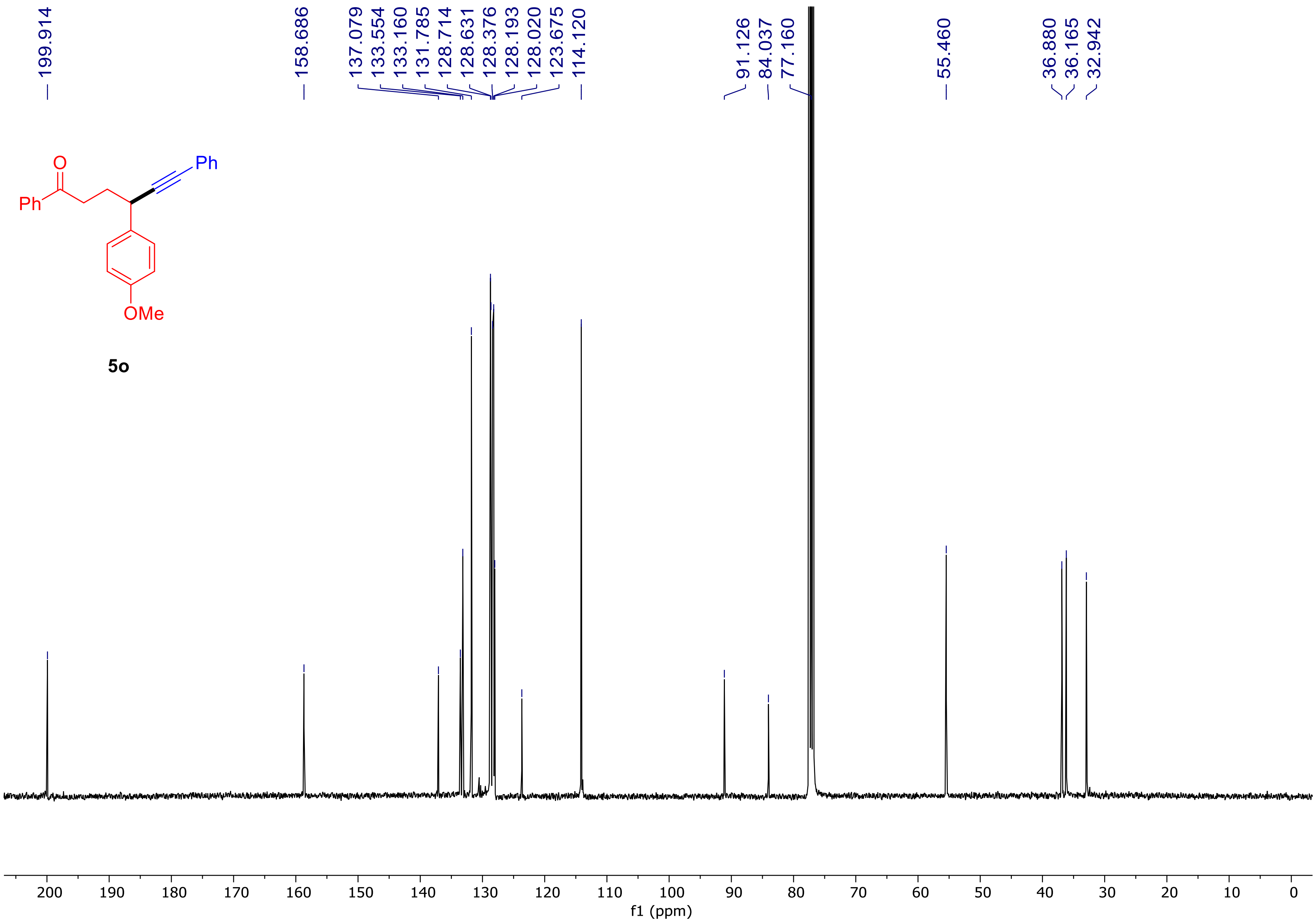

Supplementary Figure 166. ^{13}C **NMR spectrum of 5o**

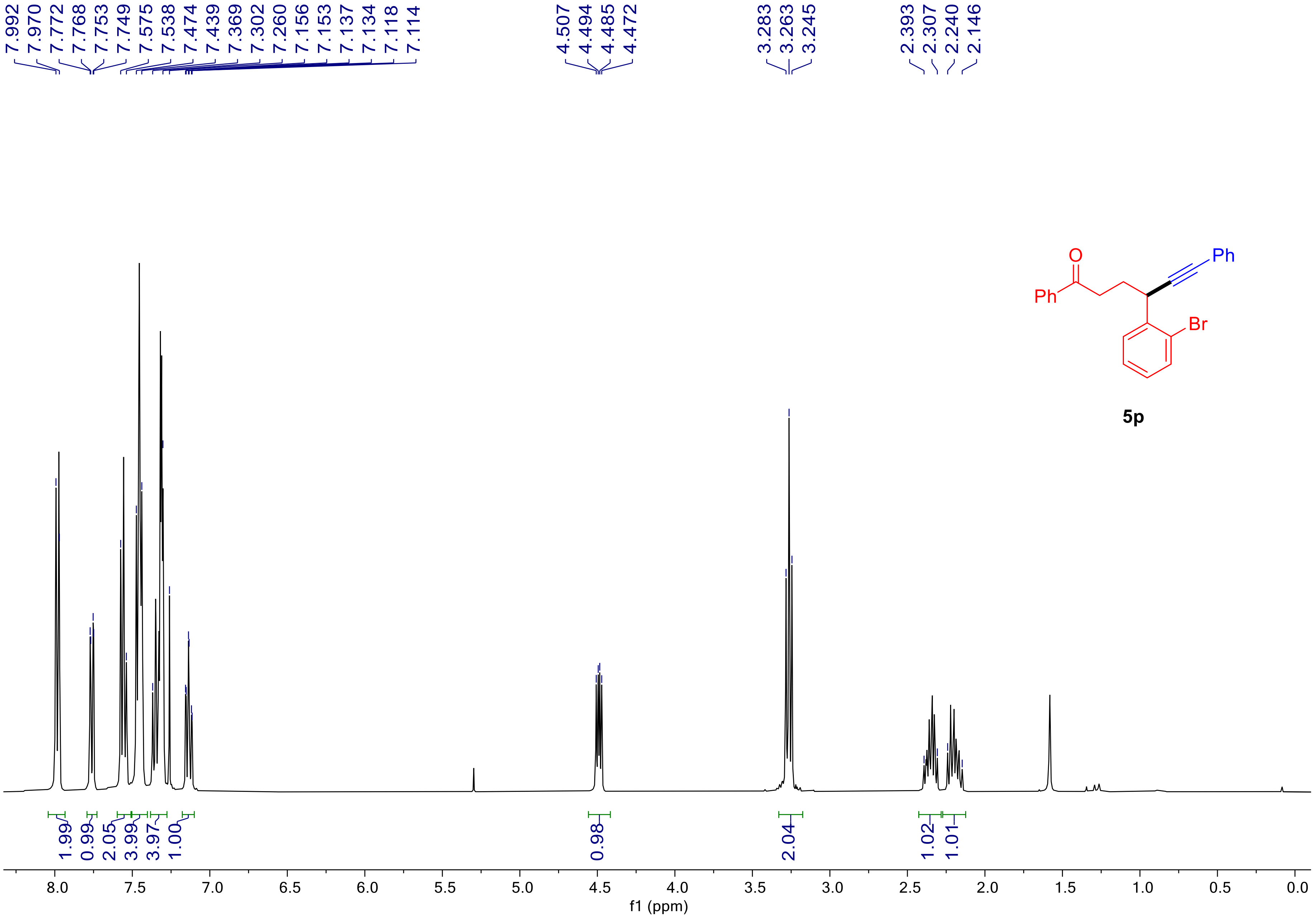

Supplementary Figure 167. ^{1}H NMR spectrum of 5p

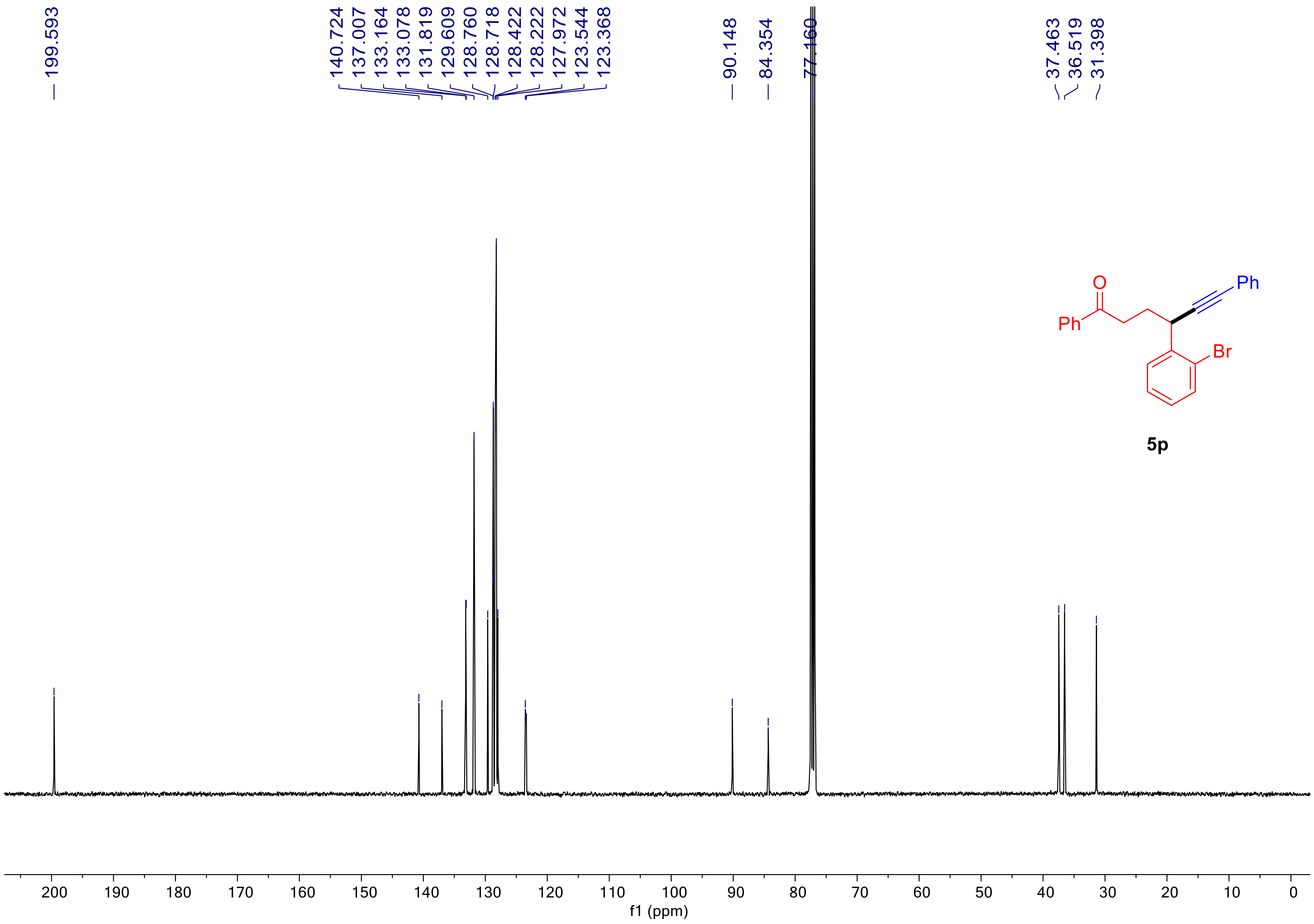

Supplementary Figure 168. ^{13}**C NMR spectrum of 5p**

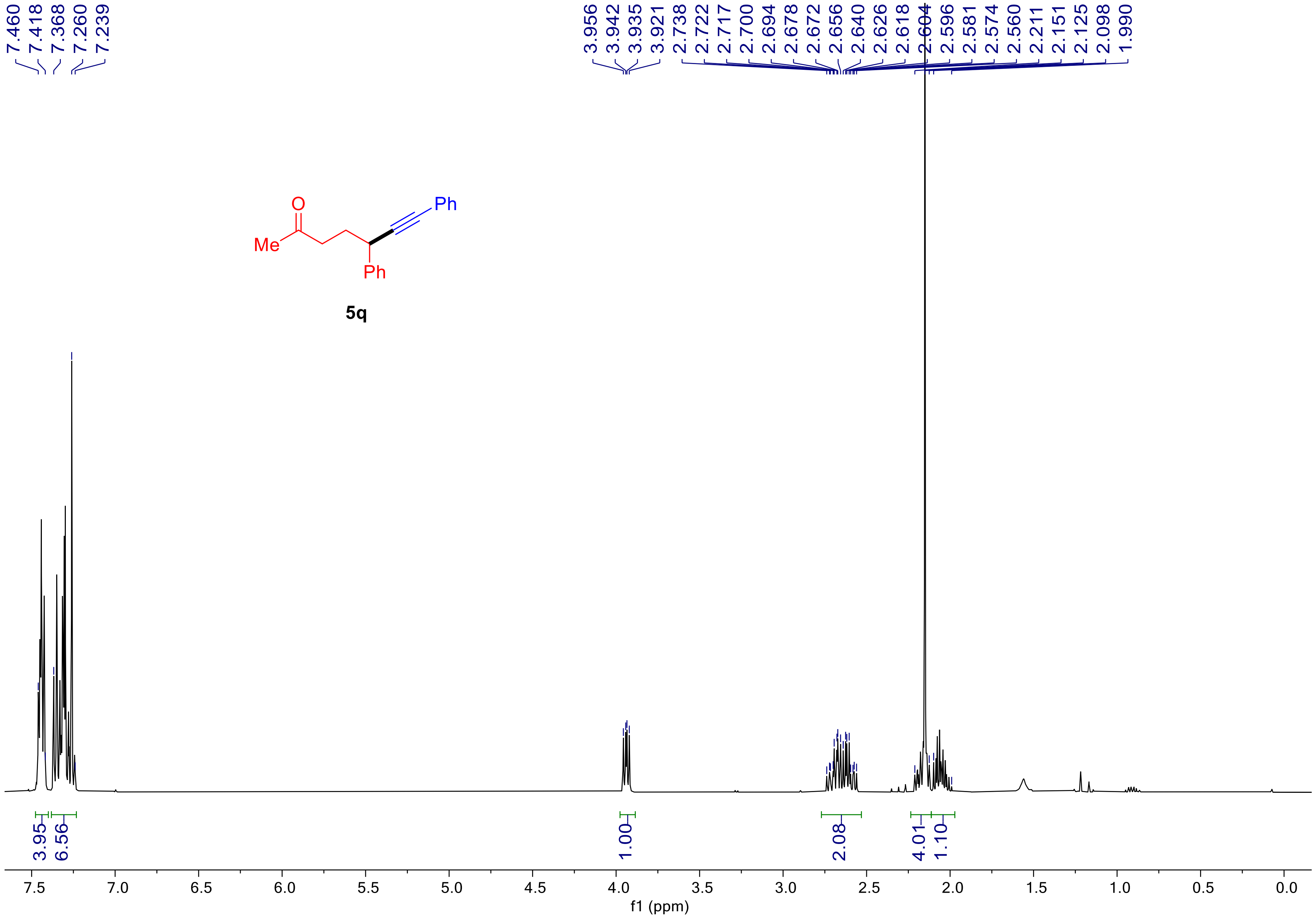

Supplementary Figure 169. ^{1}H NMR spectrum of 5q

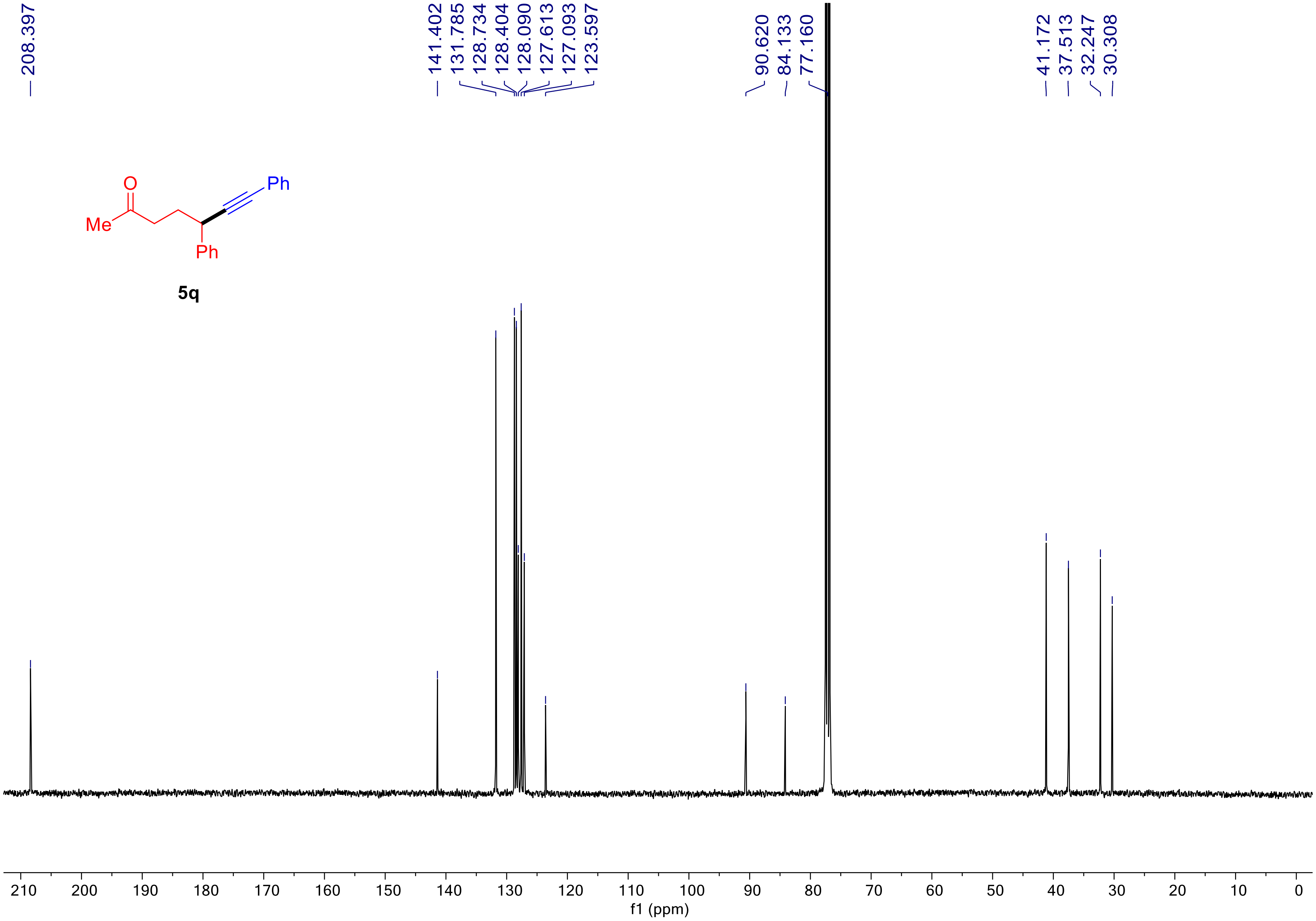

Supplementary Figure 170. ^{13}C NMR spectrum of 5q

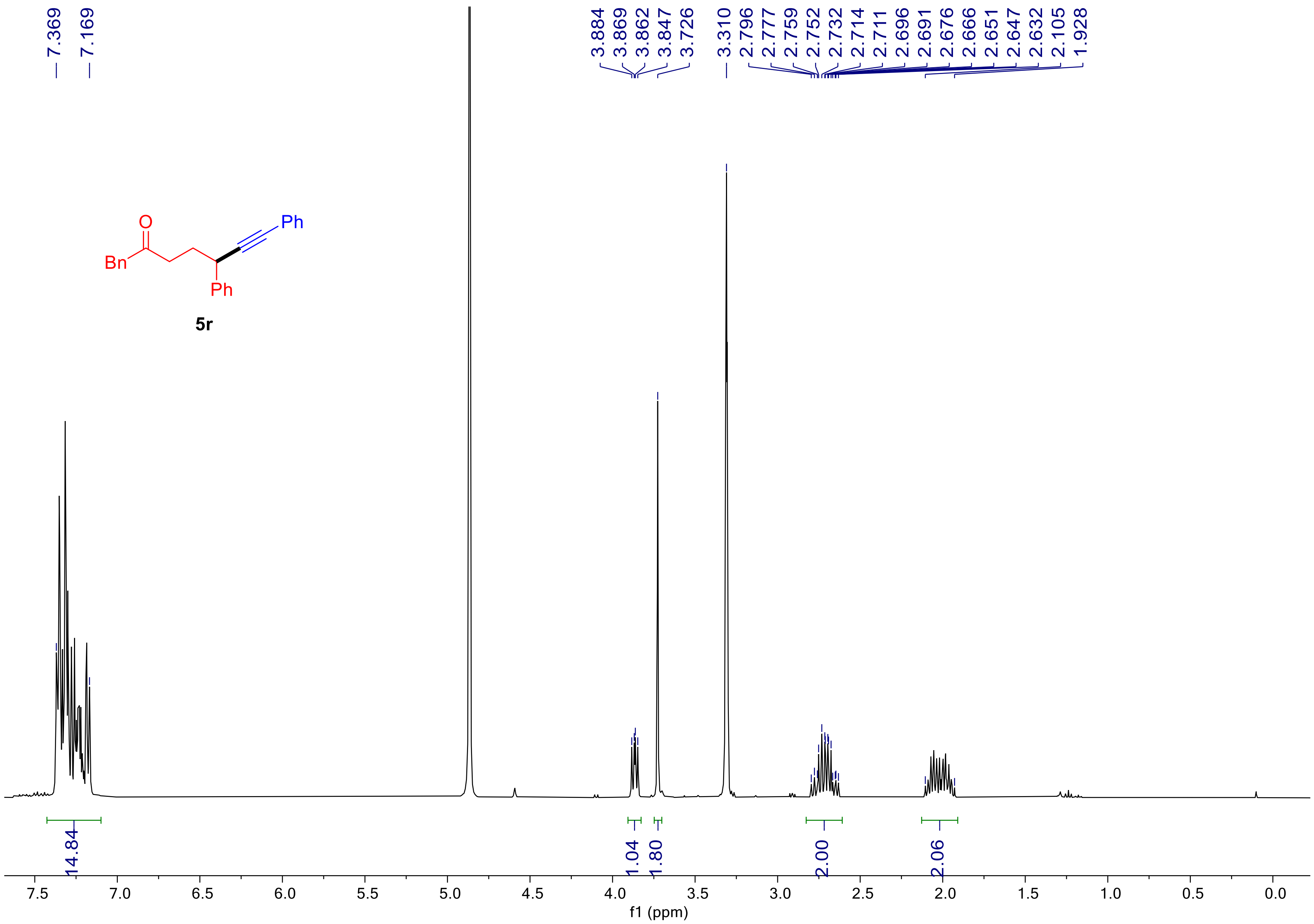

Supplementary Figure 171. ^{1}H NMR spectrum of 5r

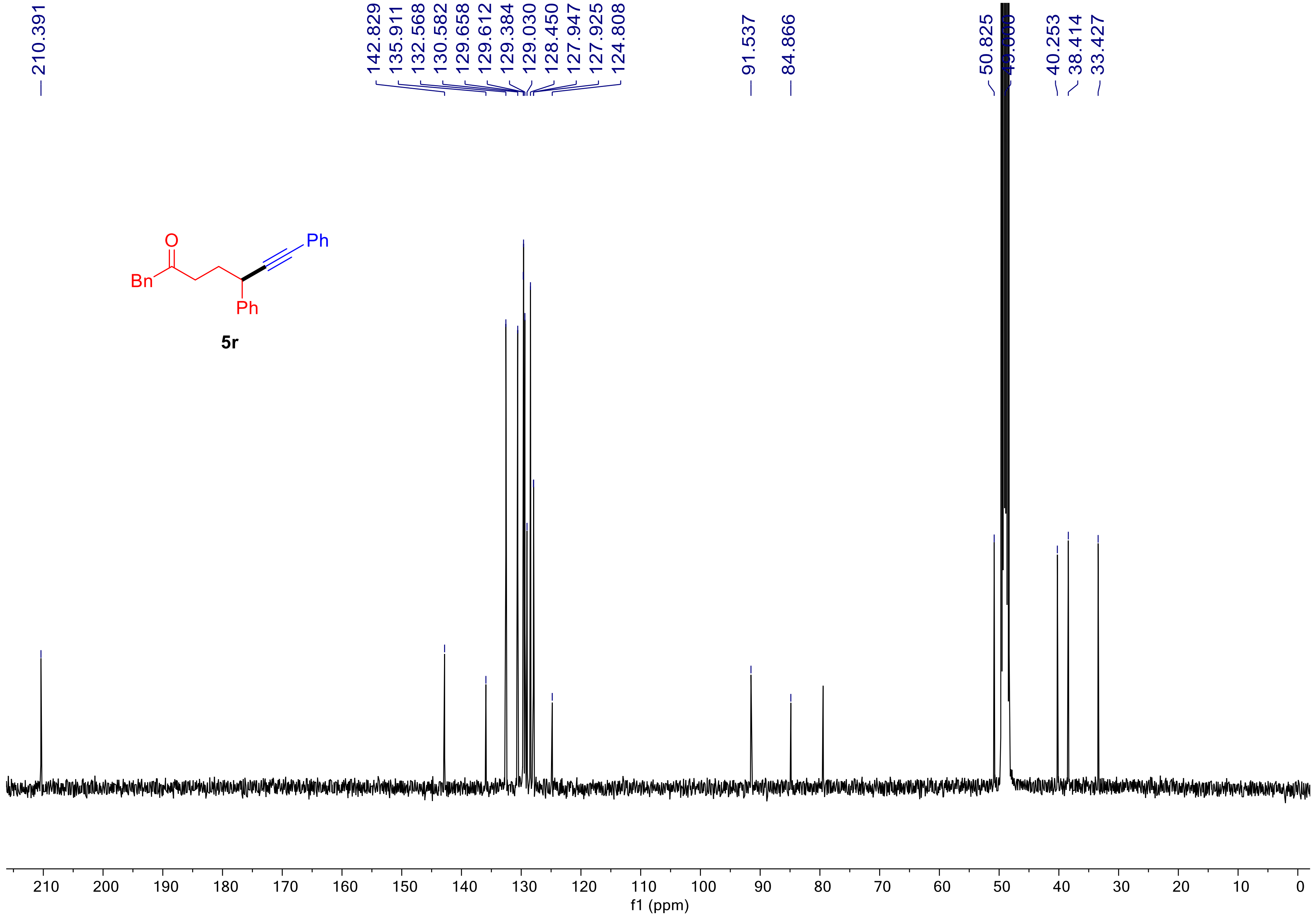

Supplementary Figure 172. ^{13}**C NMR spectrum of 5r**

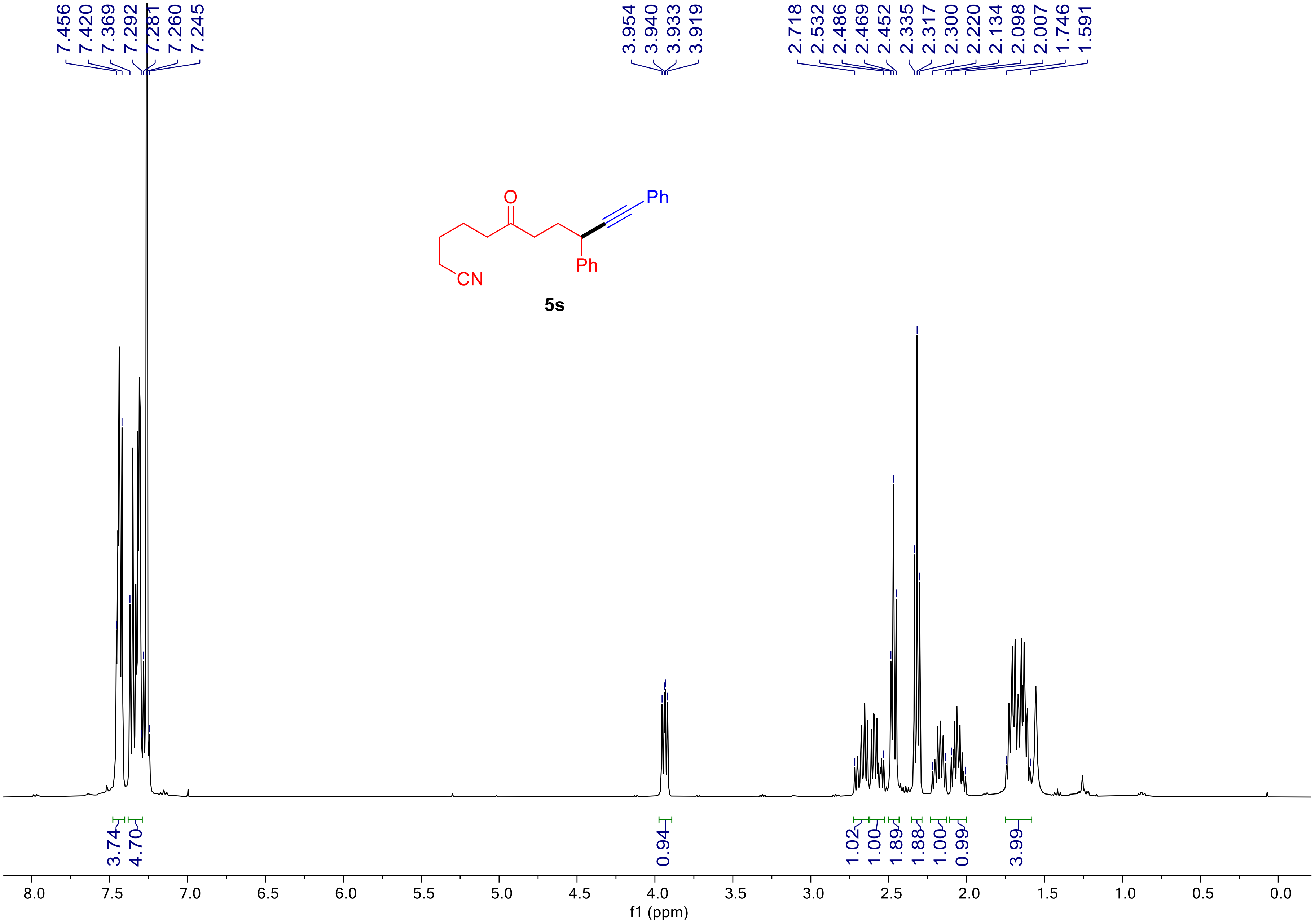

Supplementary Figure 173. ^{1}H NMR spectrum of 5s

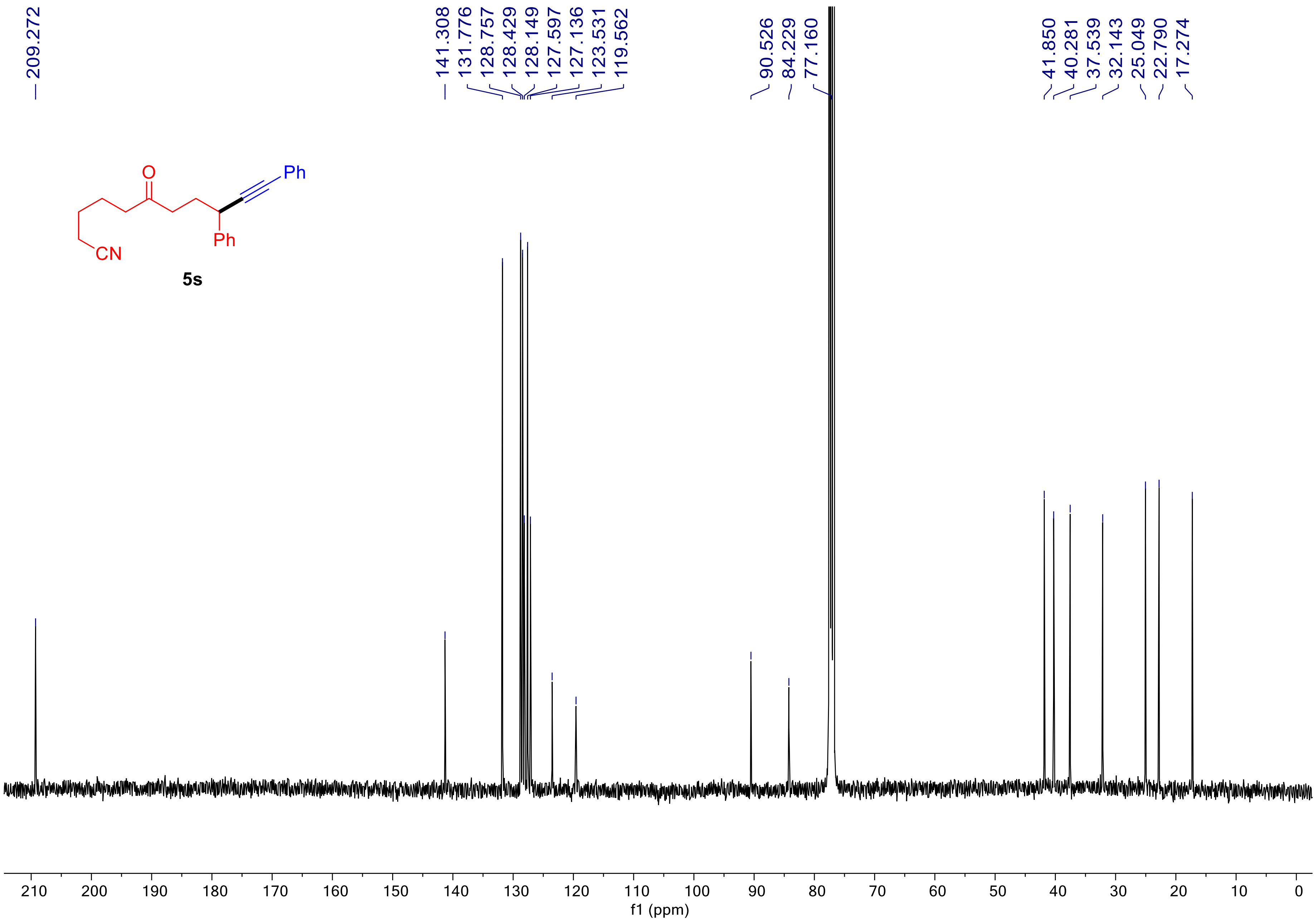

Supplementary Figure 174. ^{13}C NMR spectrum of 5s

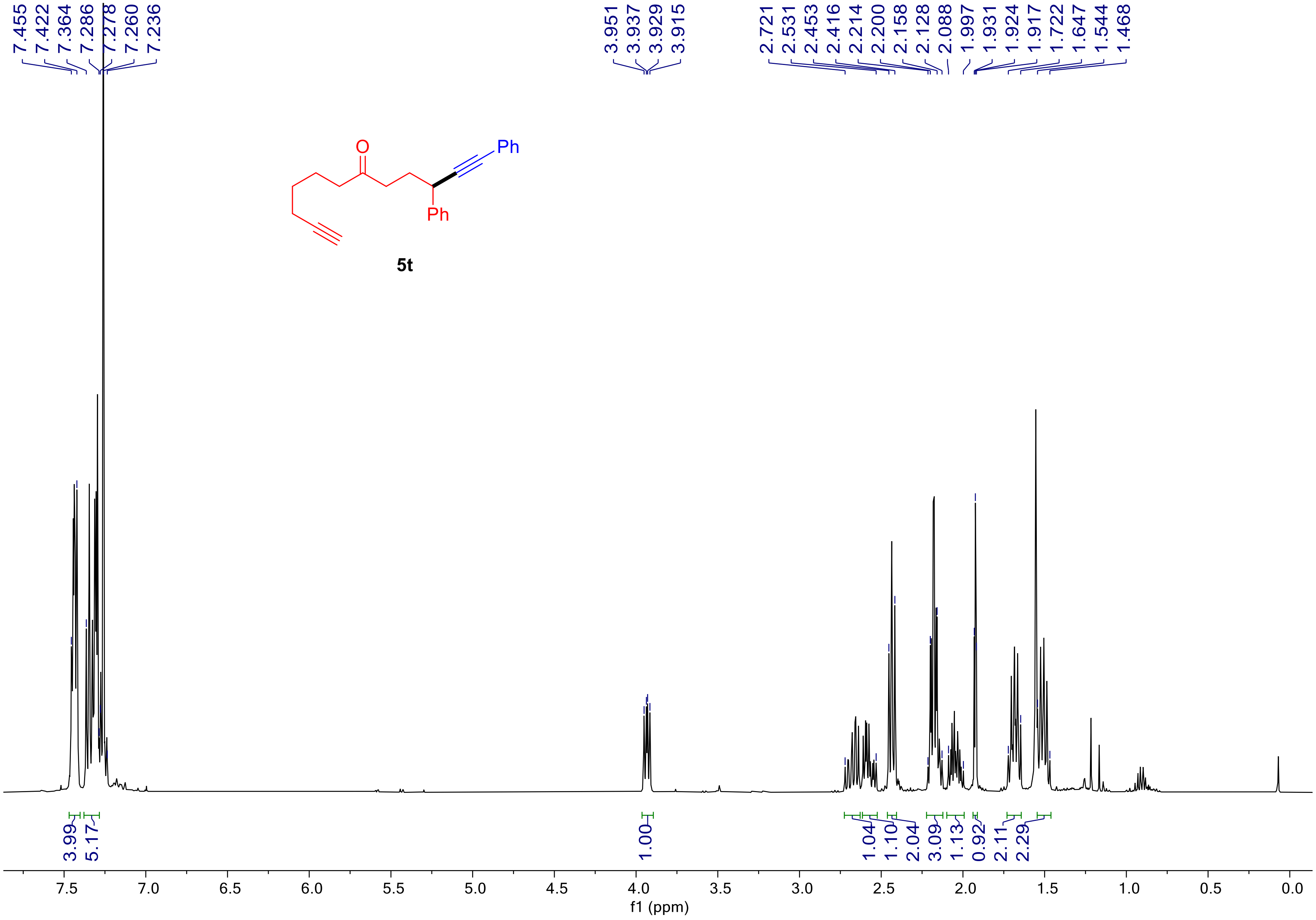

Supplementary Figure 175. ^{1}H NMR spectrum of 5t

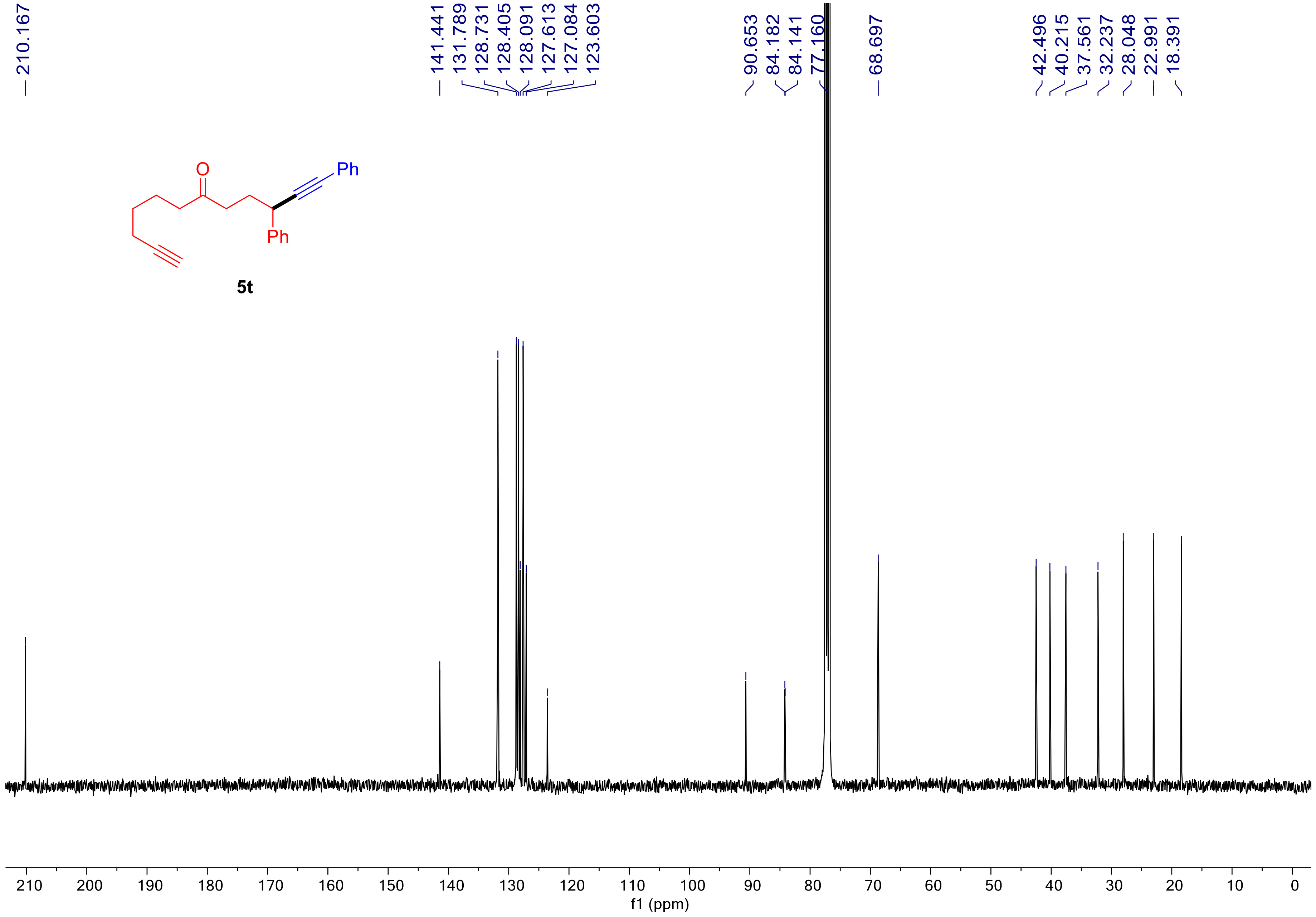

Supplementary Figure 176. ^{13}C NMR spectrum of 5t

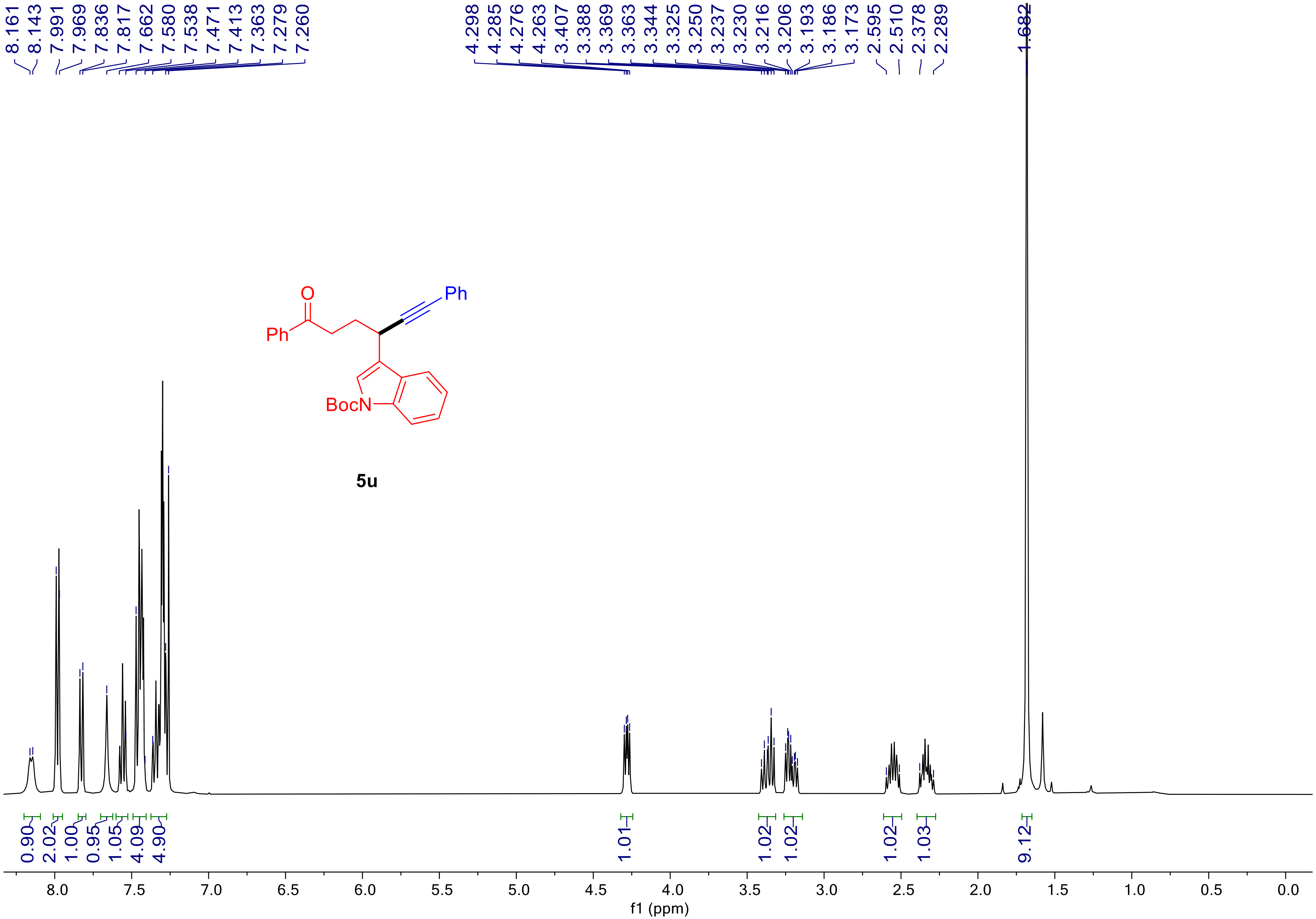

Supplementary Figure 177. ^{1}H NMR spectrum of 5u

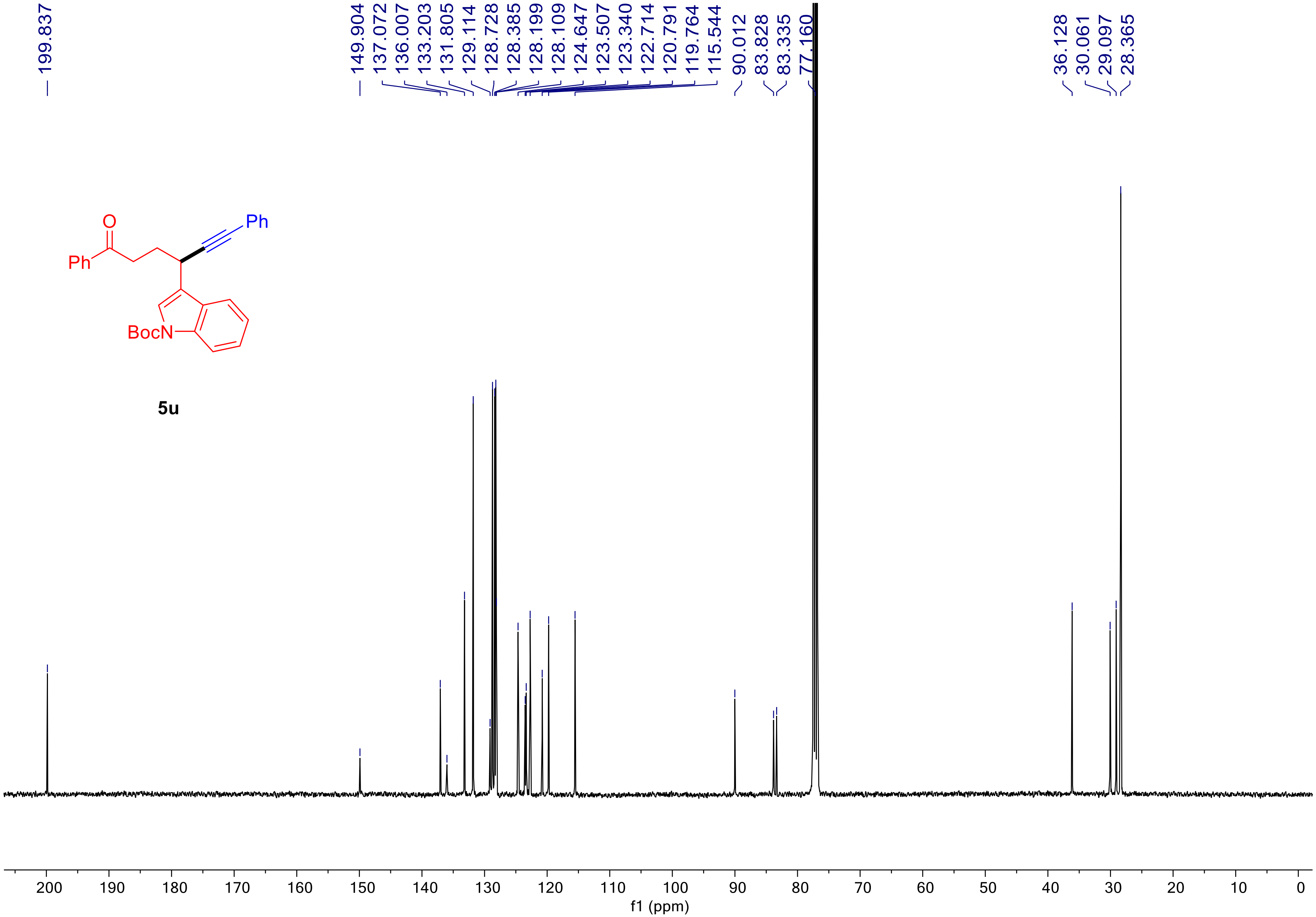

Supplementary Figure 178. ^{13}**C NMR spectrum of 5u**

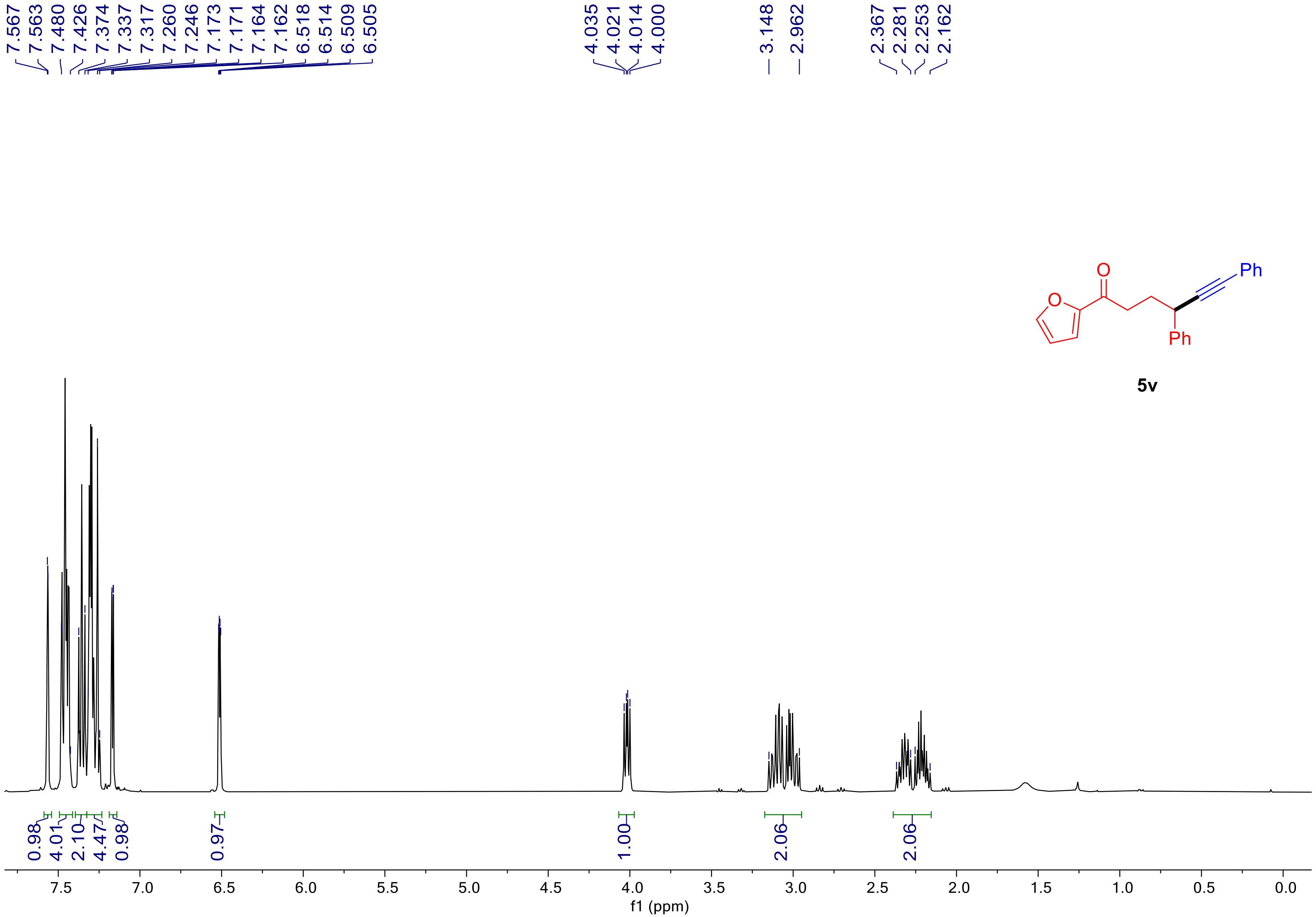

Supplementary Figure 179. ^{1}H NMR spectrum of 5v

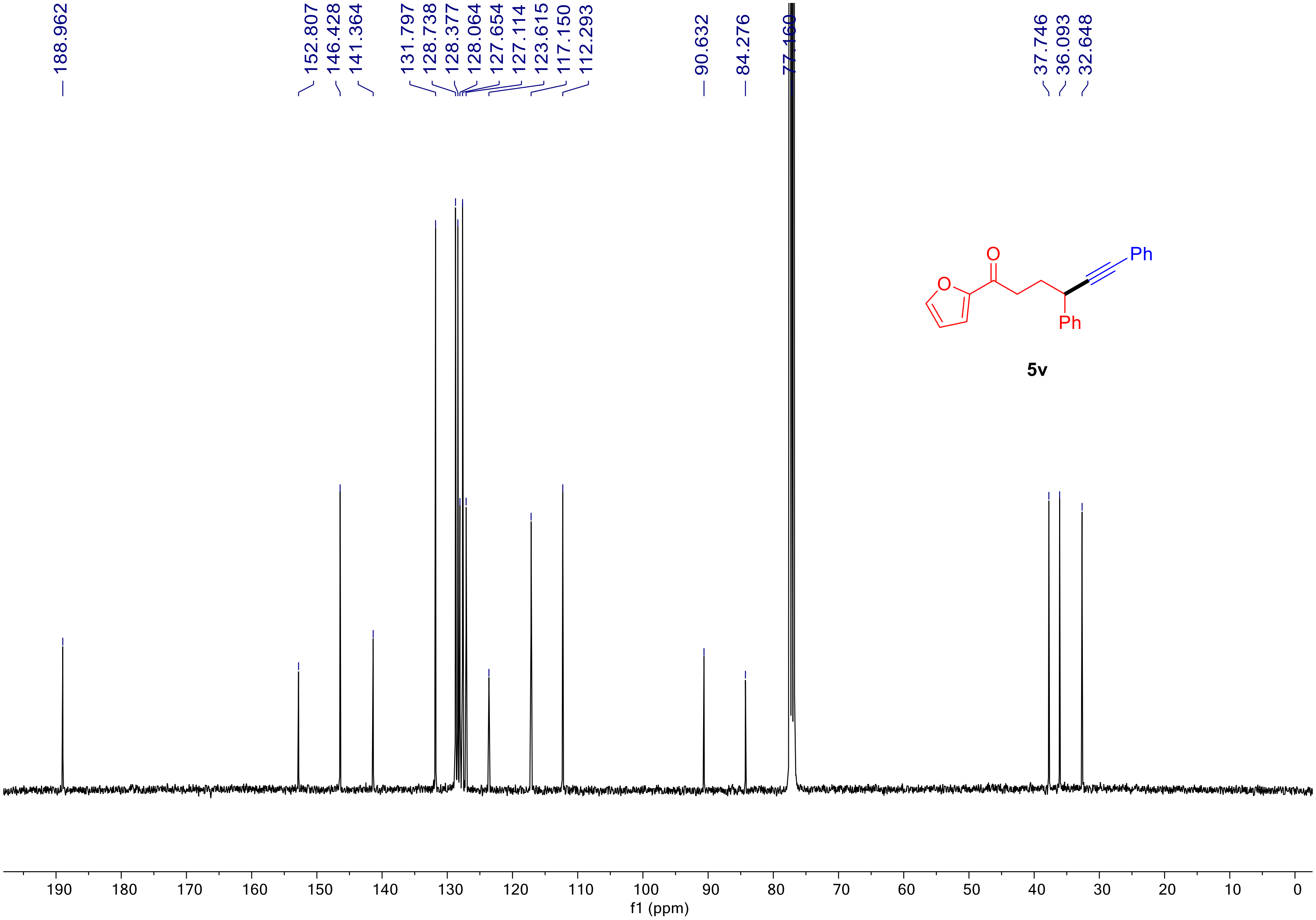

Supplementary Figure 180. ^{13}C **NMR spectrum of 5v**

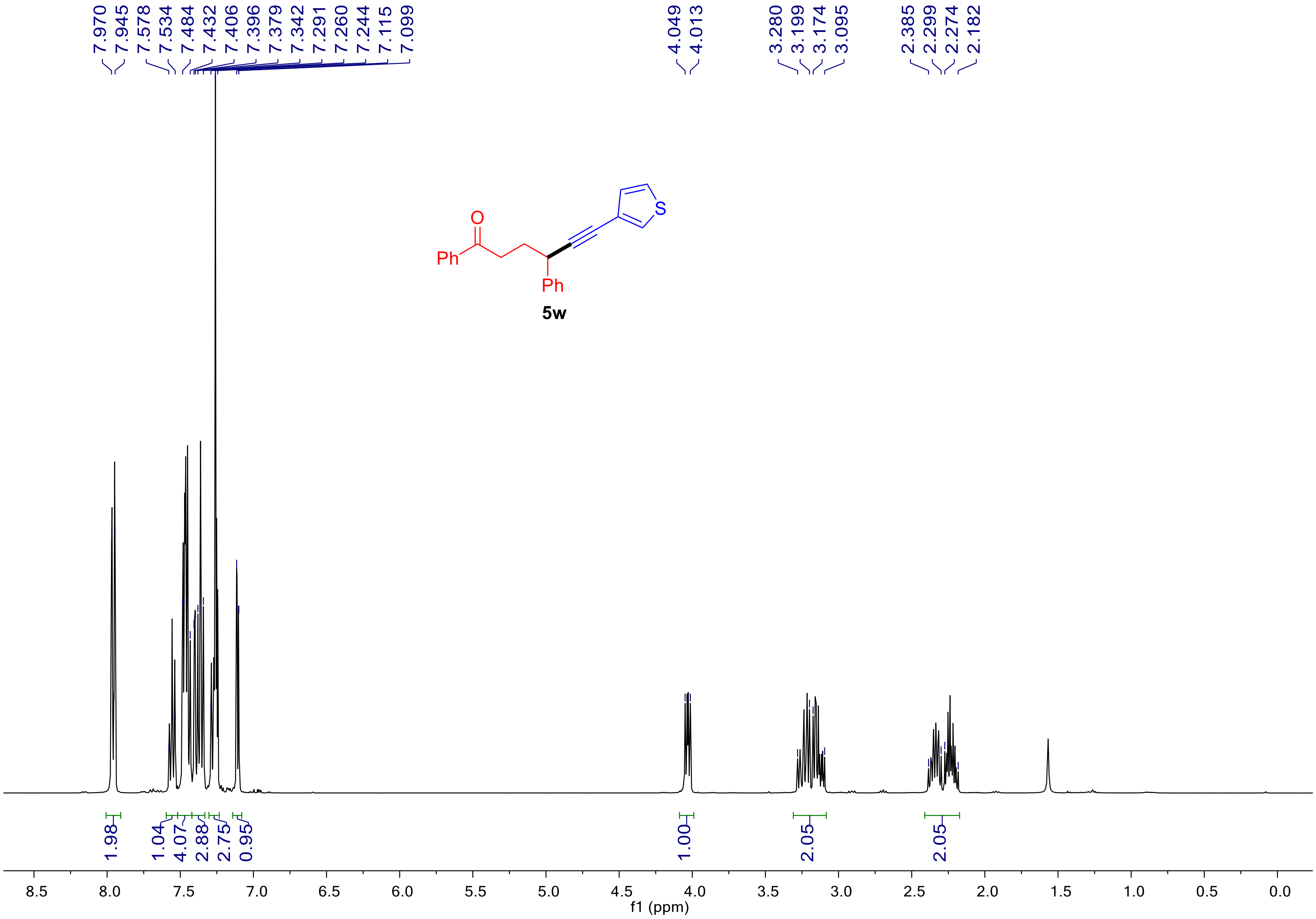

Supplementary Figure 181. ^{1}H NMR spectrum of 5w

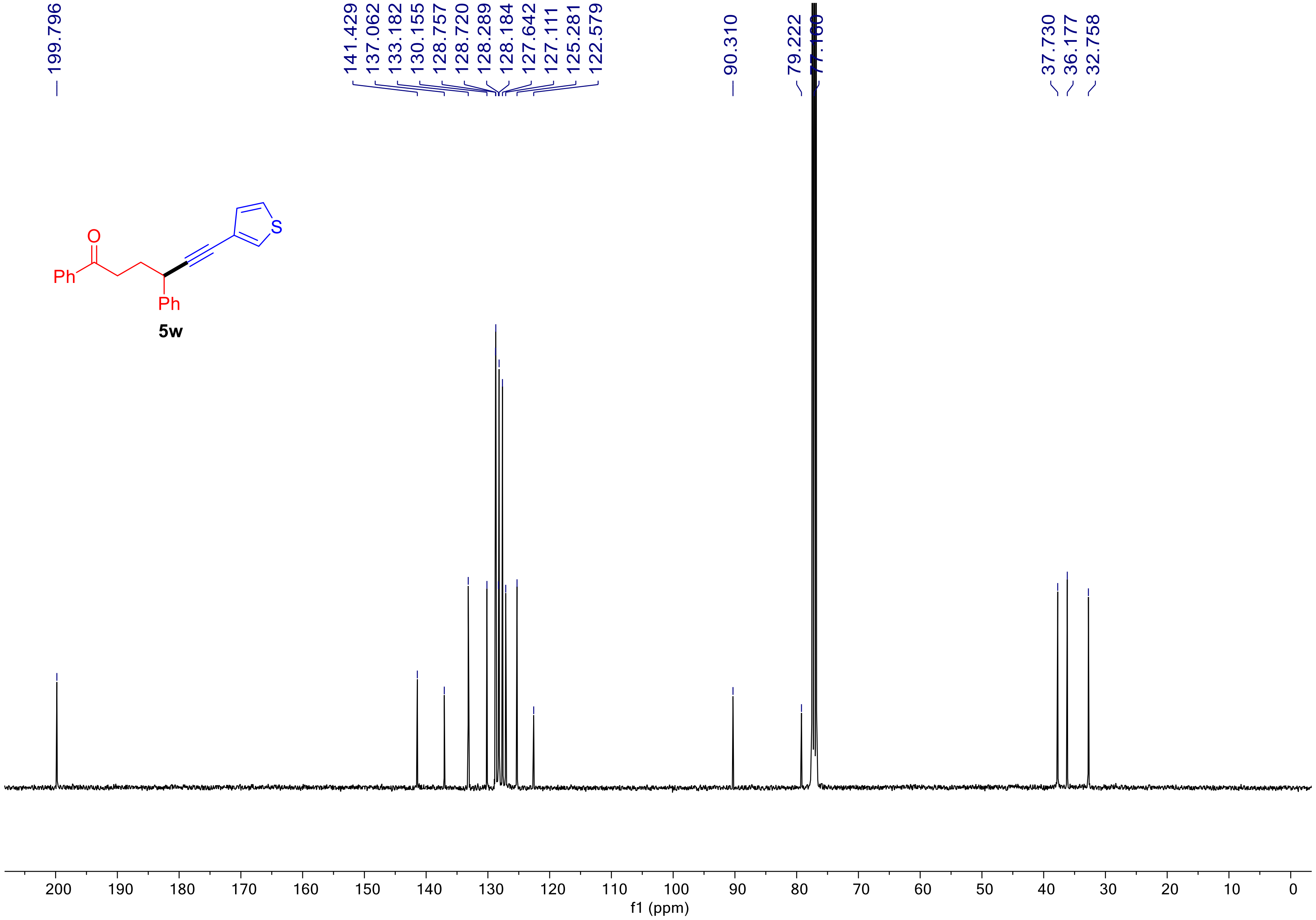

Supplementary Figure 182. ^{13}**C NMR spectrum of 5w**

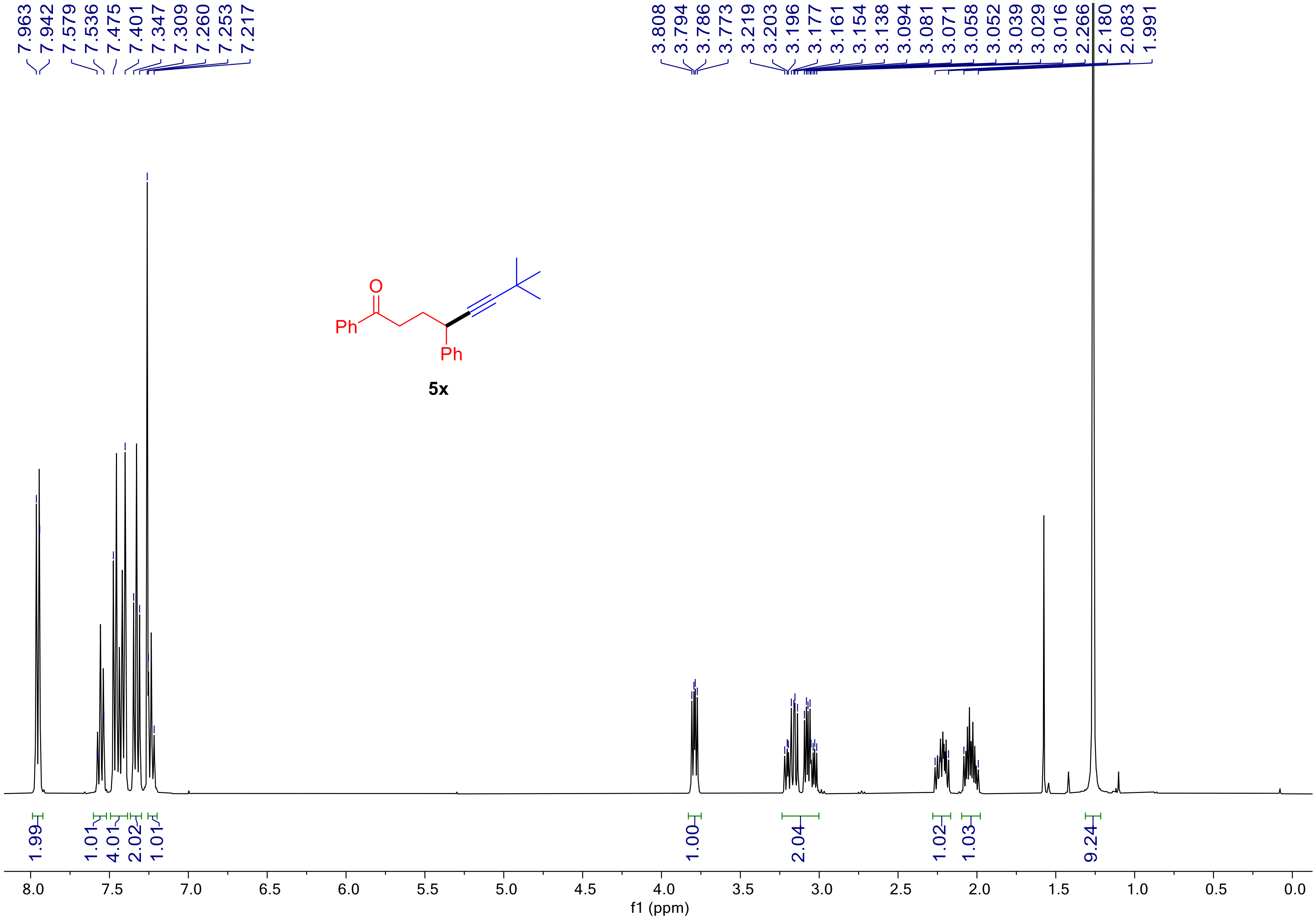

5x

Supplementary Figure 183. ^{1}H NMR spectrum of 5x

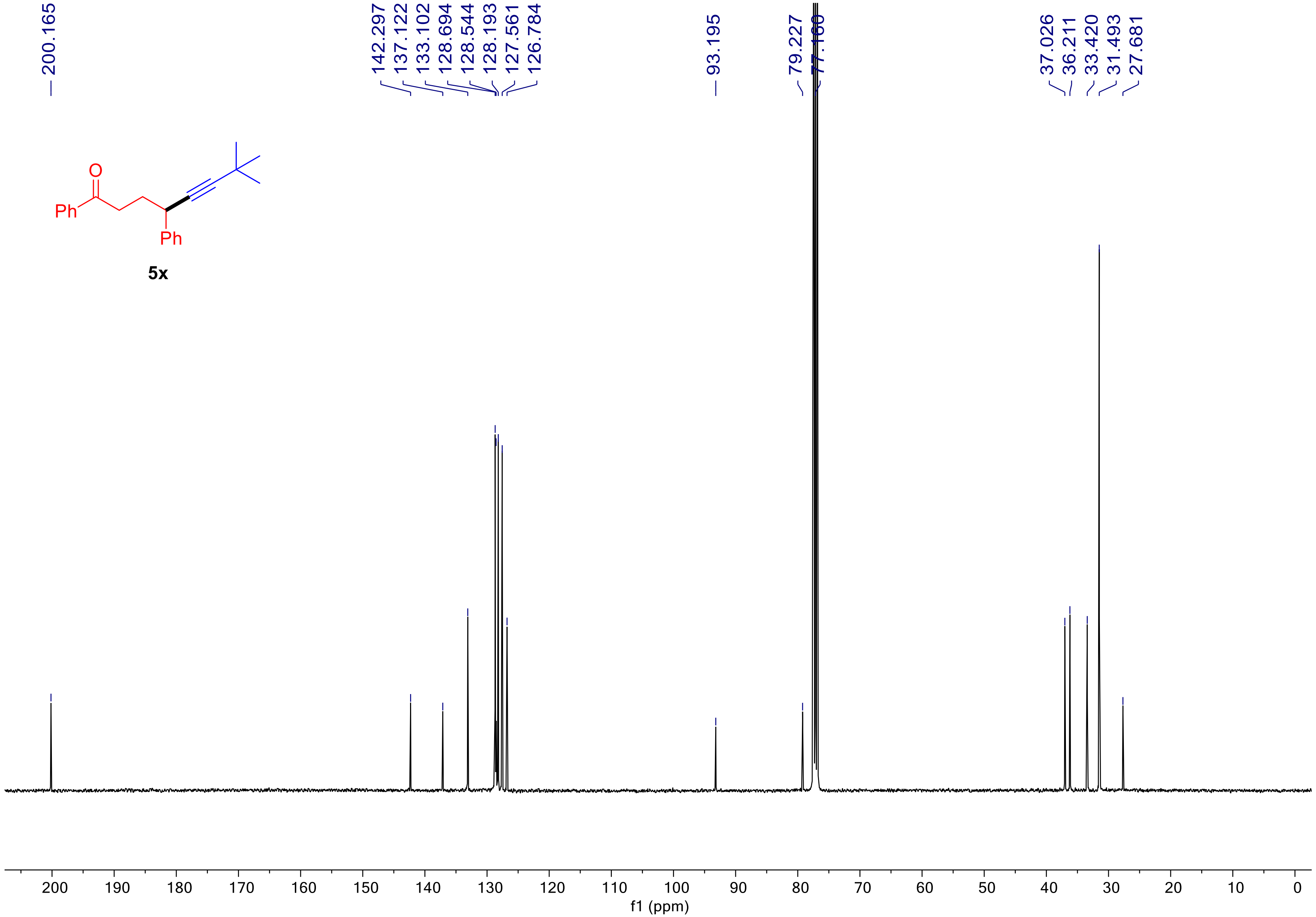

Supplementary Figure 184. ^{13}**C NMR spectrum of 5x**

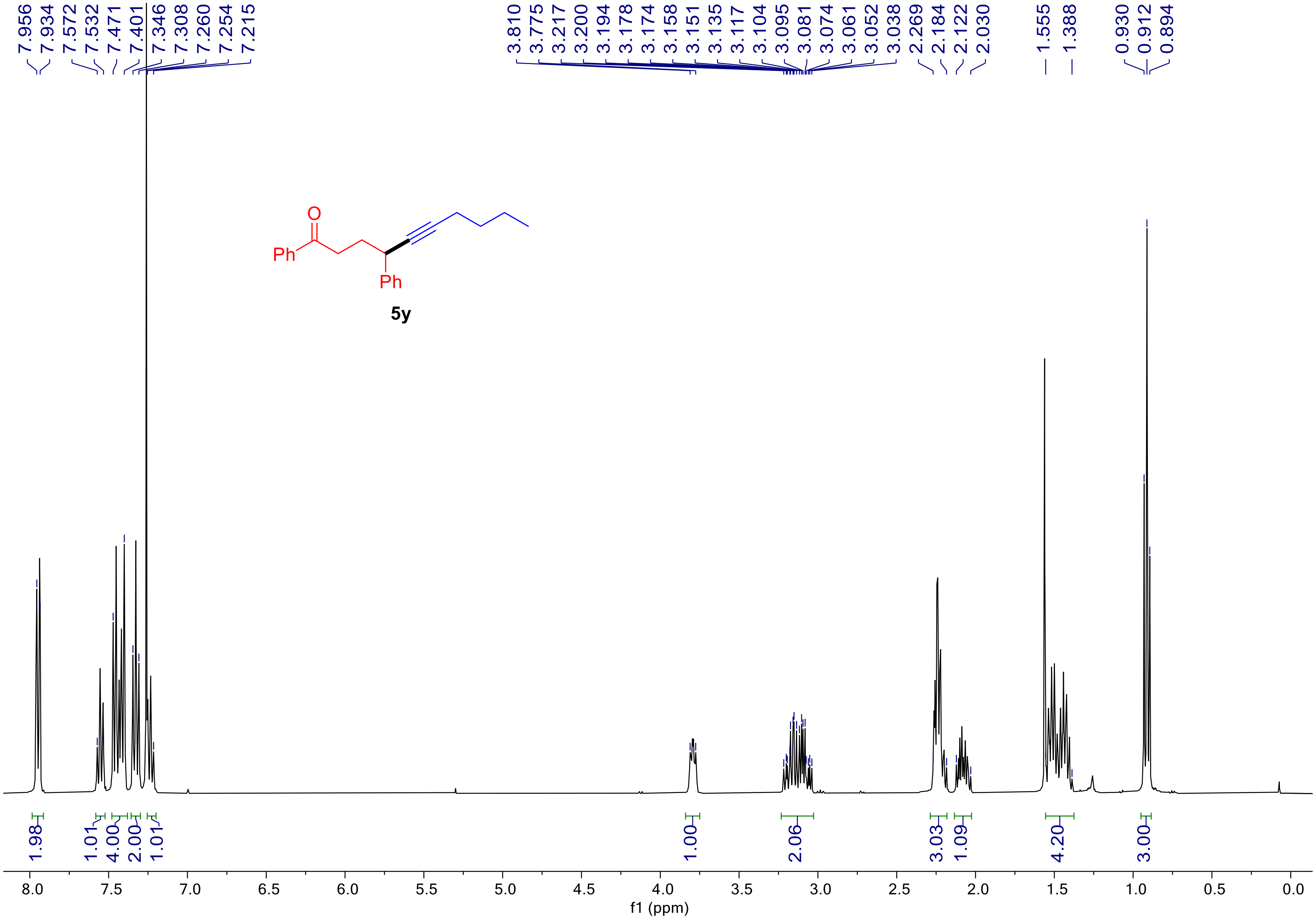

Supplementary Figure 185. ^{1}H NMR spectrum of 5y

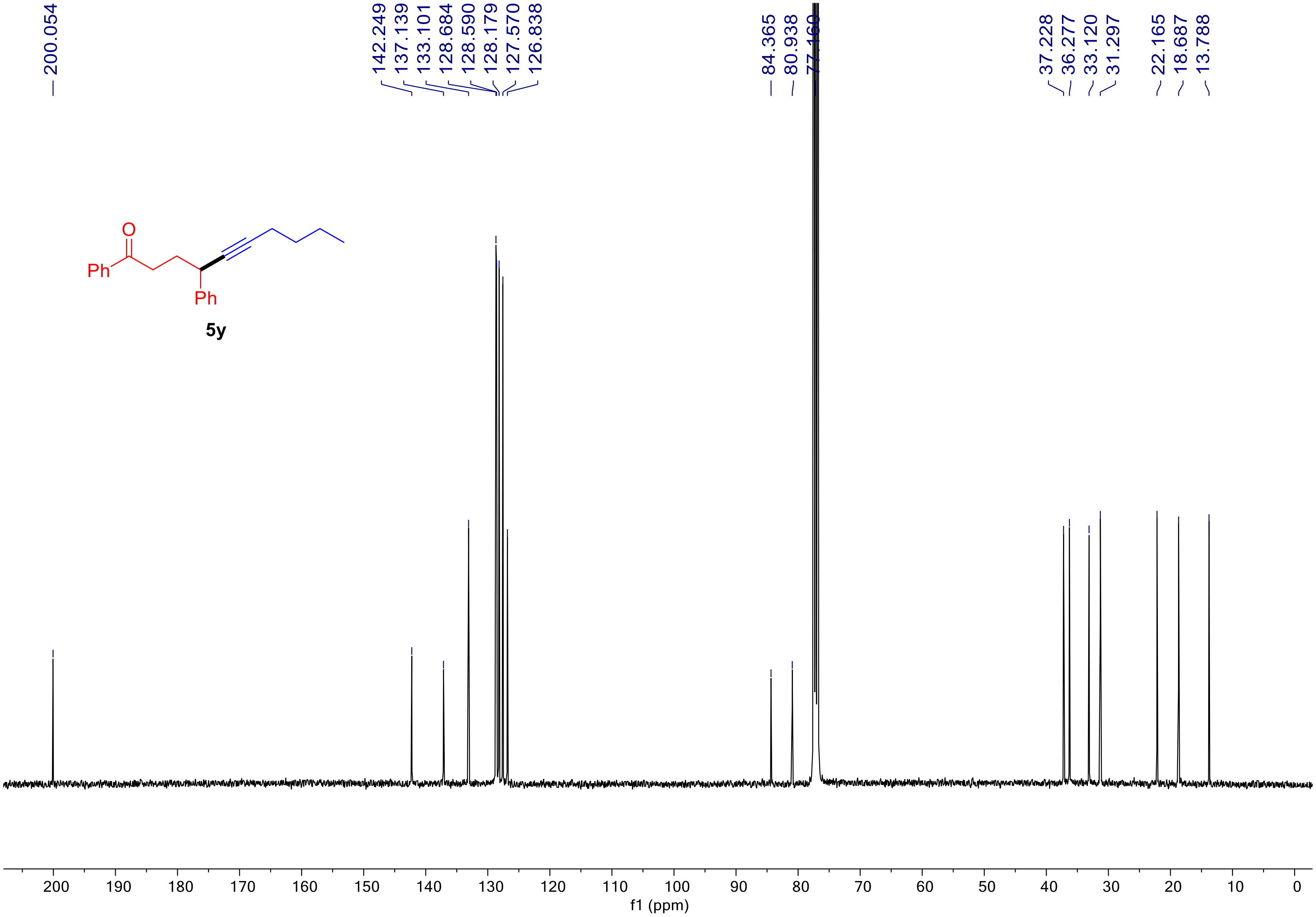

Supplementary Figure 186. ^{13}C NMR spectrum of 5y

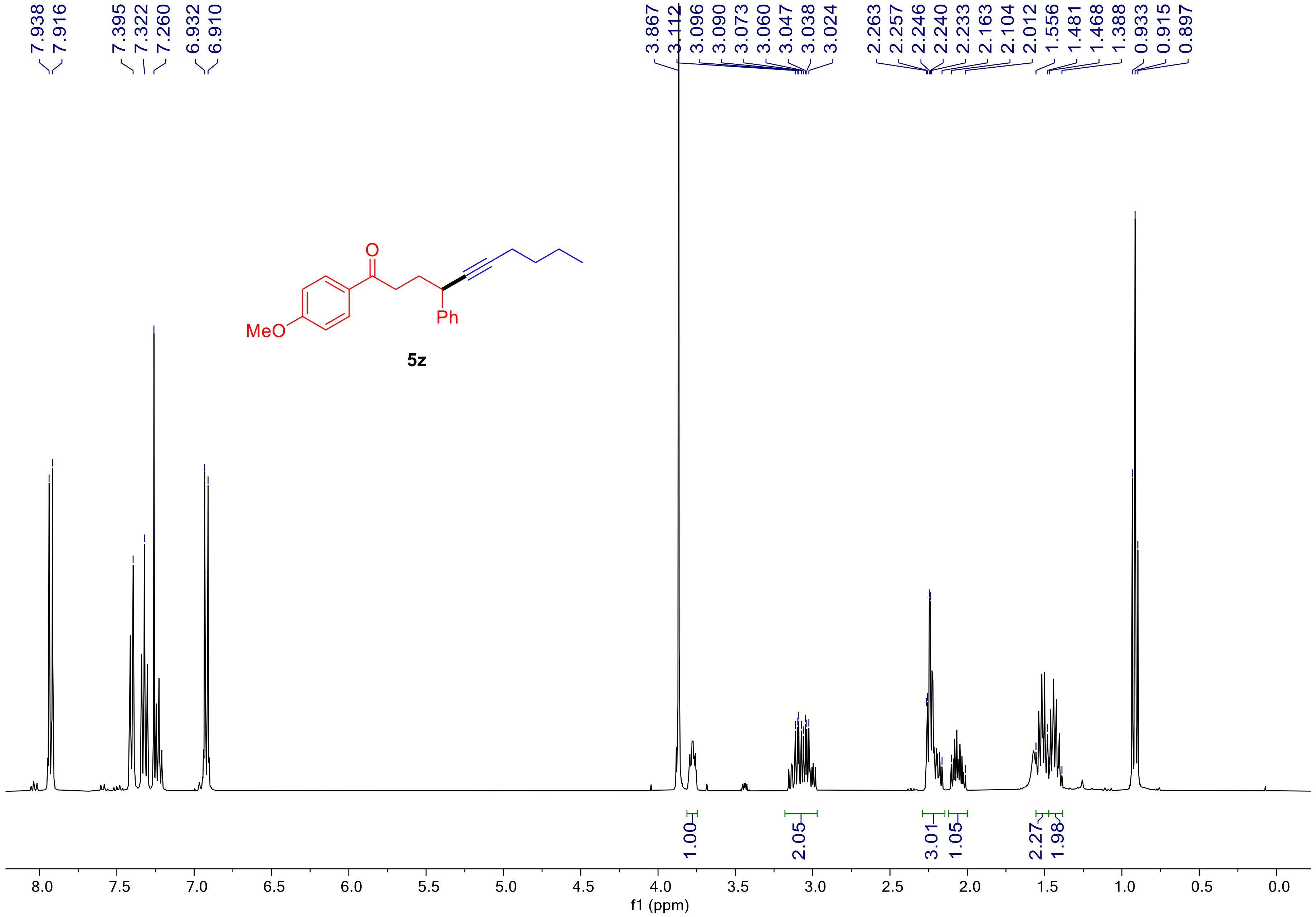

Supplementary Figure 187. ^{1}H NMR spectrum of 5z

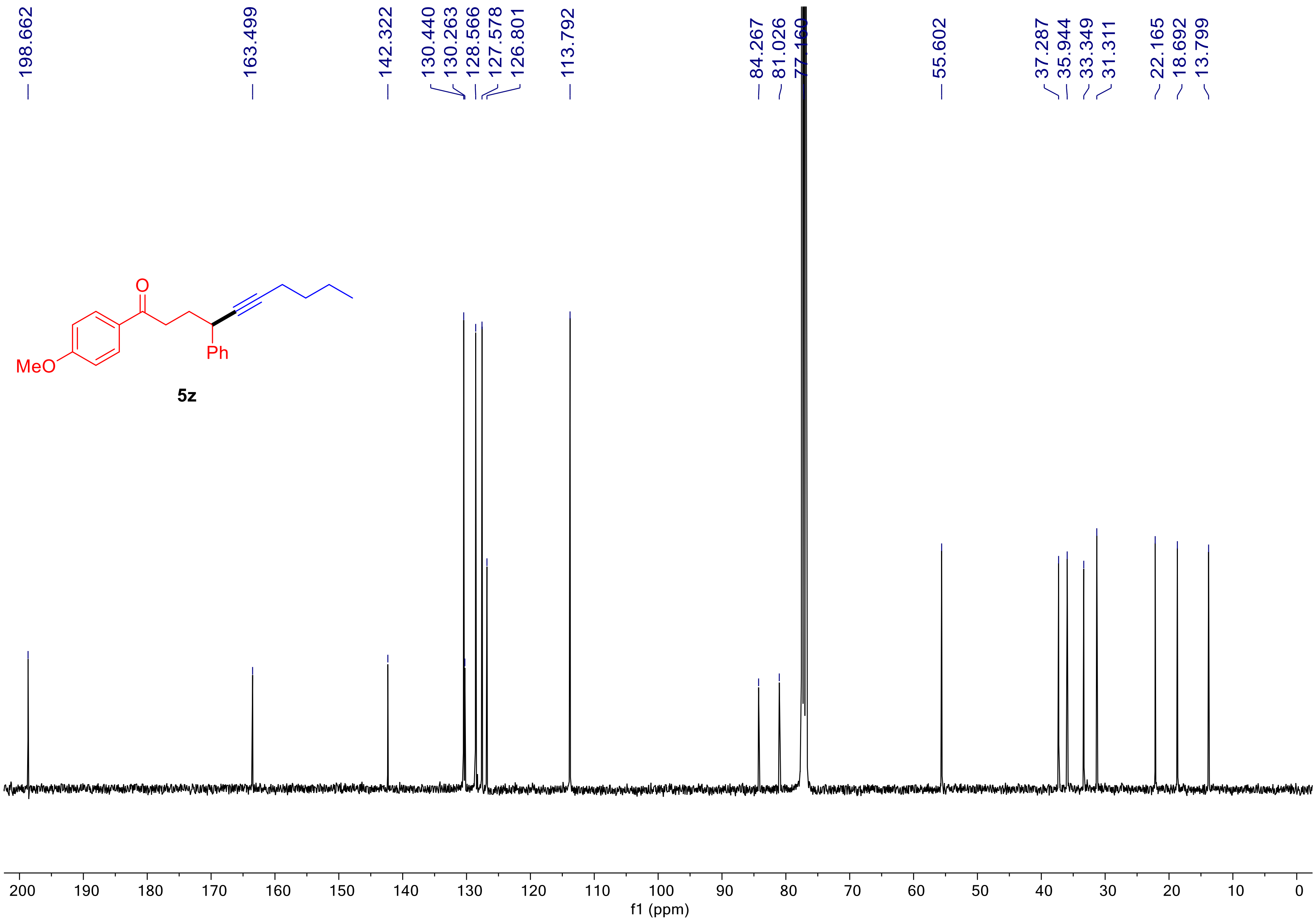

Supplementary Figure 188. ^{13}C NMR spectrum of 5z

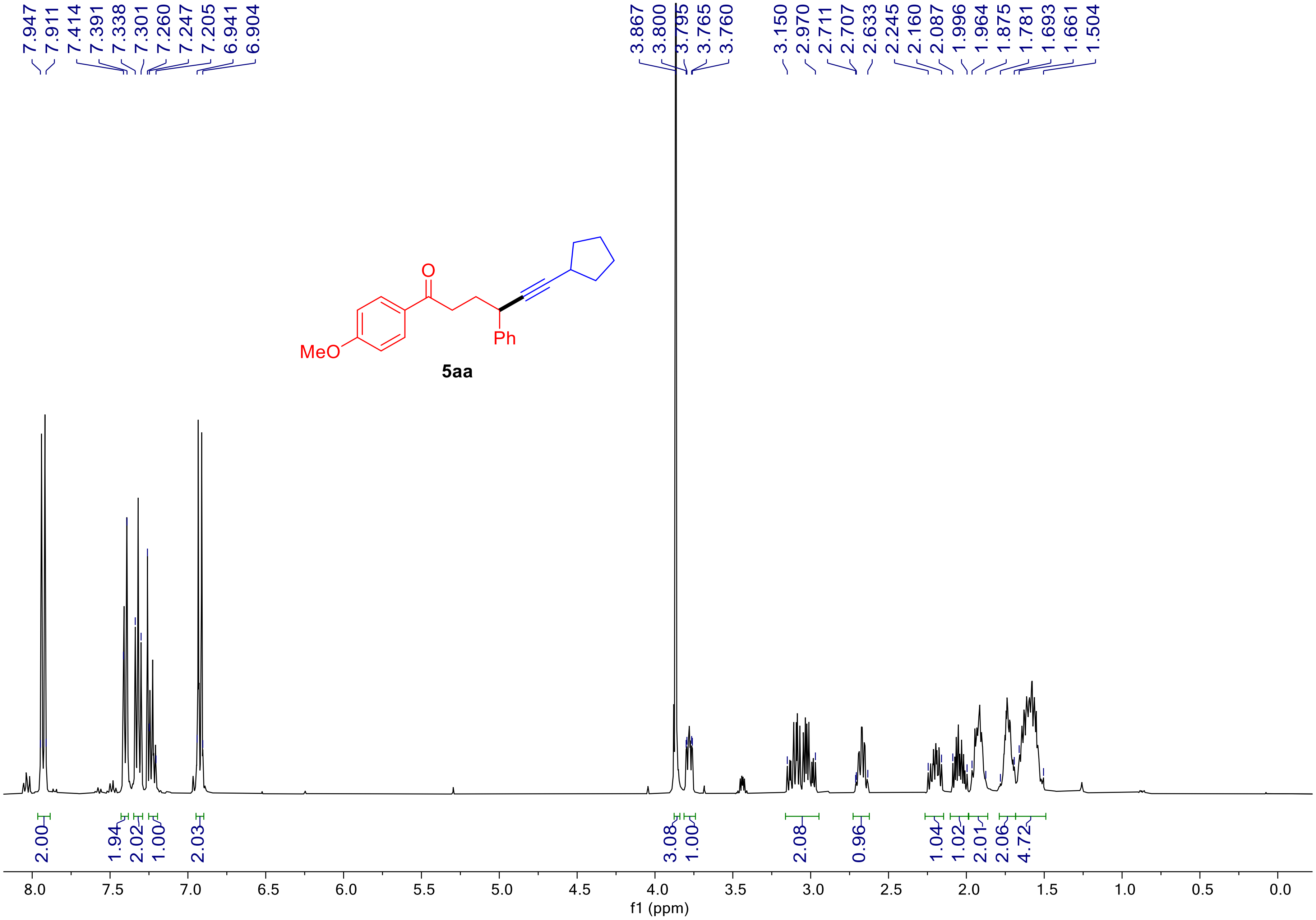

Supplementary Figure 189. ^{1}H NMR spectrum of 5aa

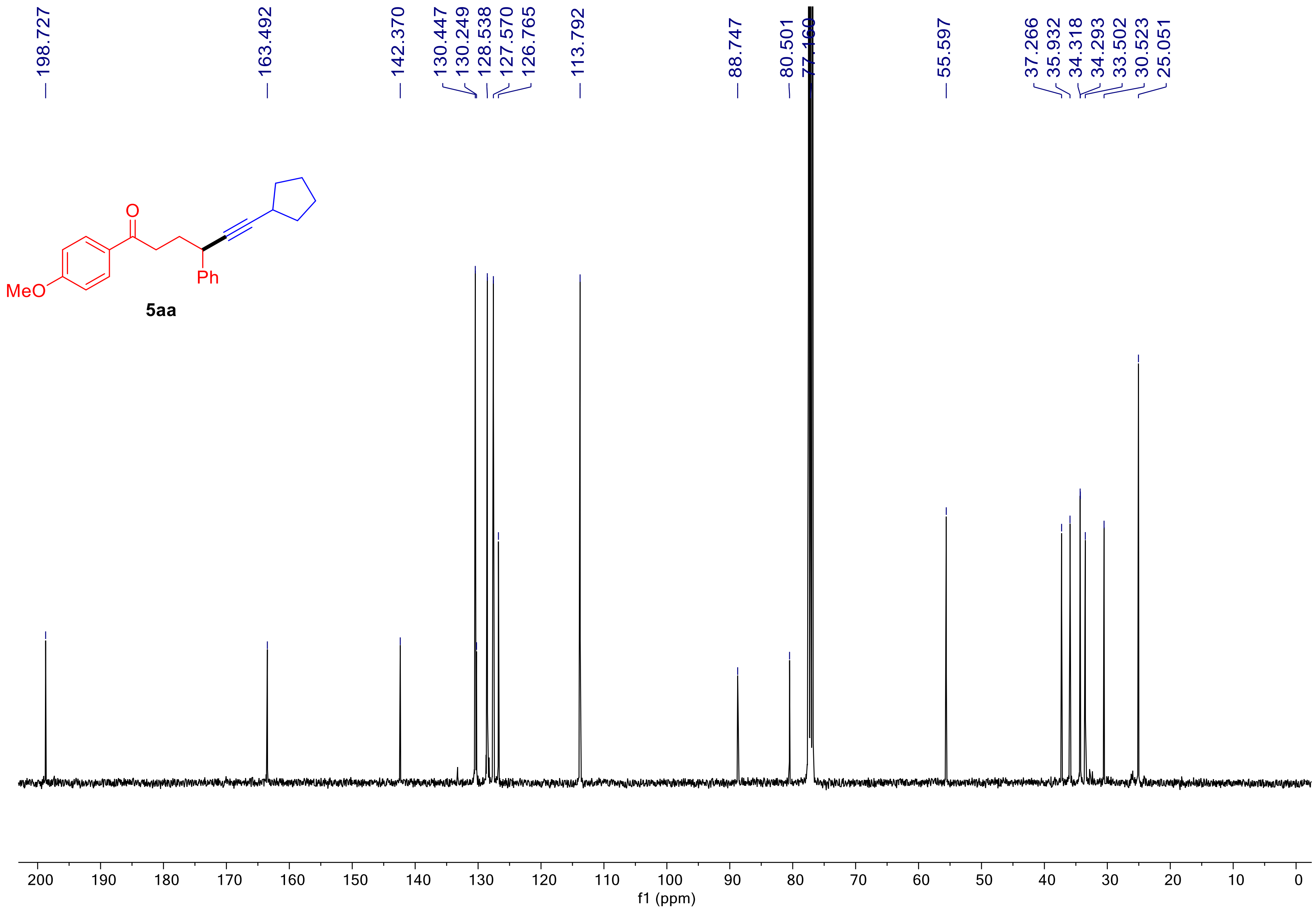

Supplementary Figure 190. ^{13}C NMR spectrum of 5aa

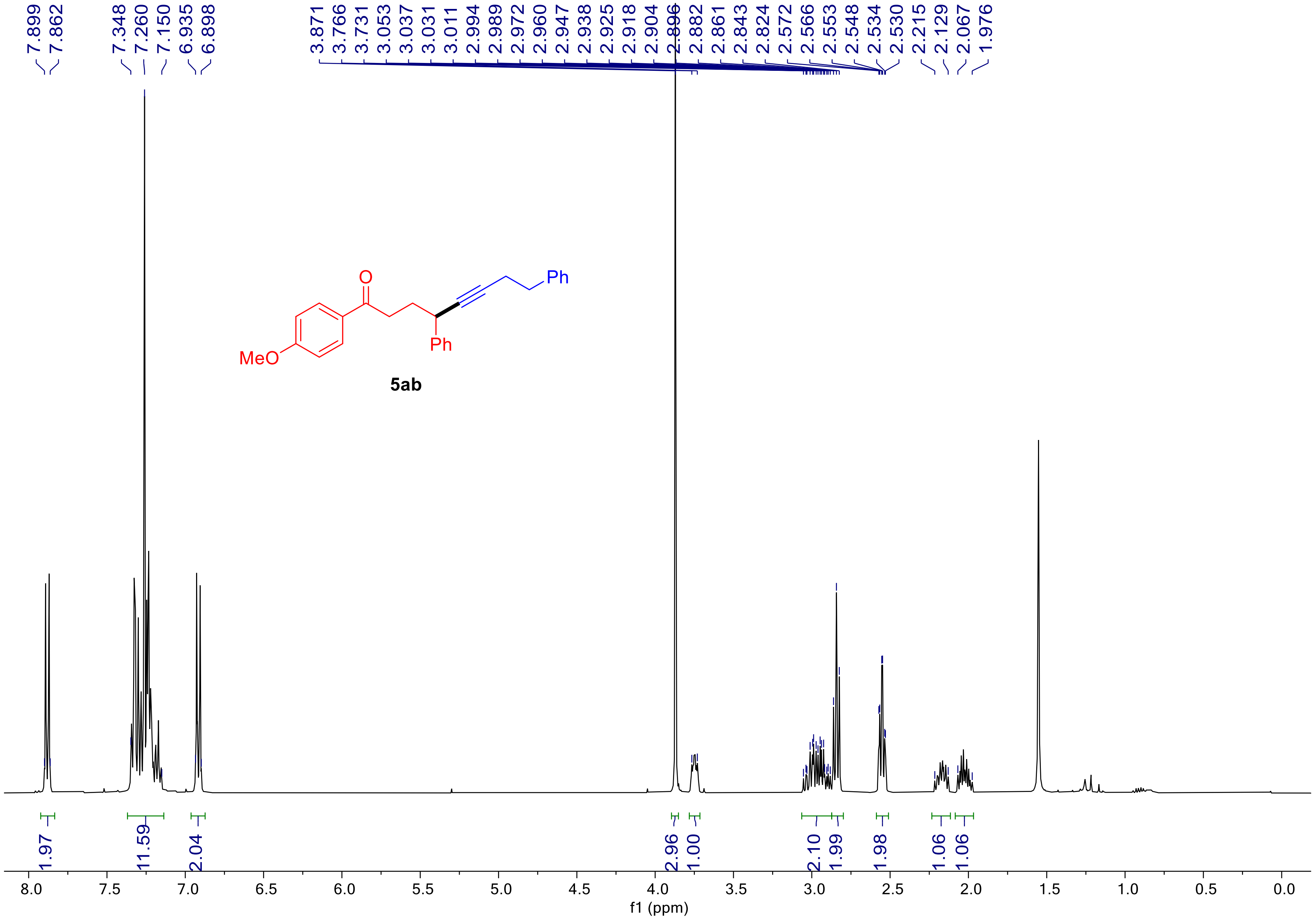

Supplementary Figure 191. ^{1}H NMR spectrum of 5ab

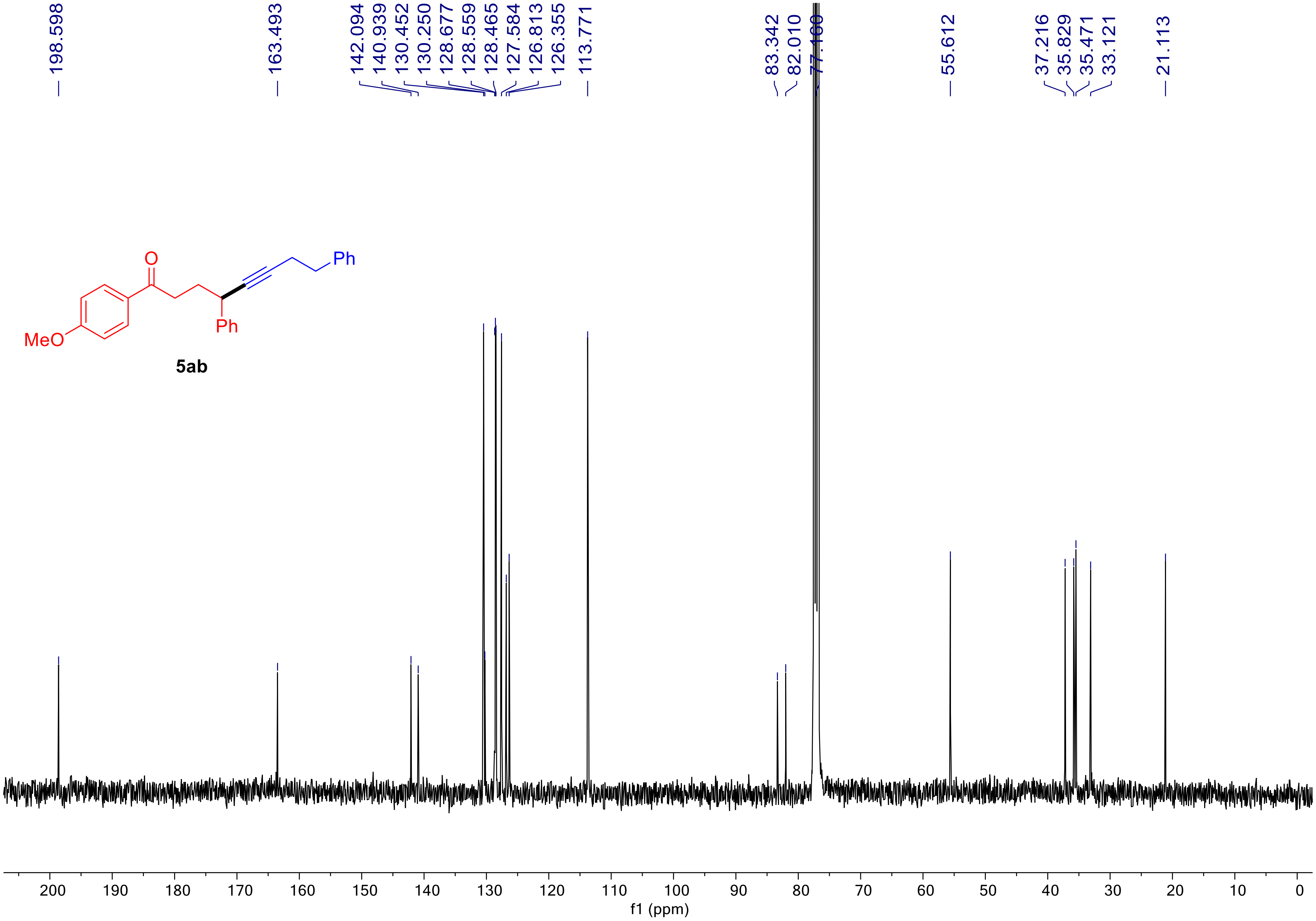

Supplementary Figure 192. ^{13}C NMR spectrum of 5ab

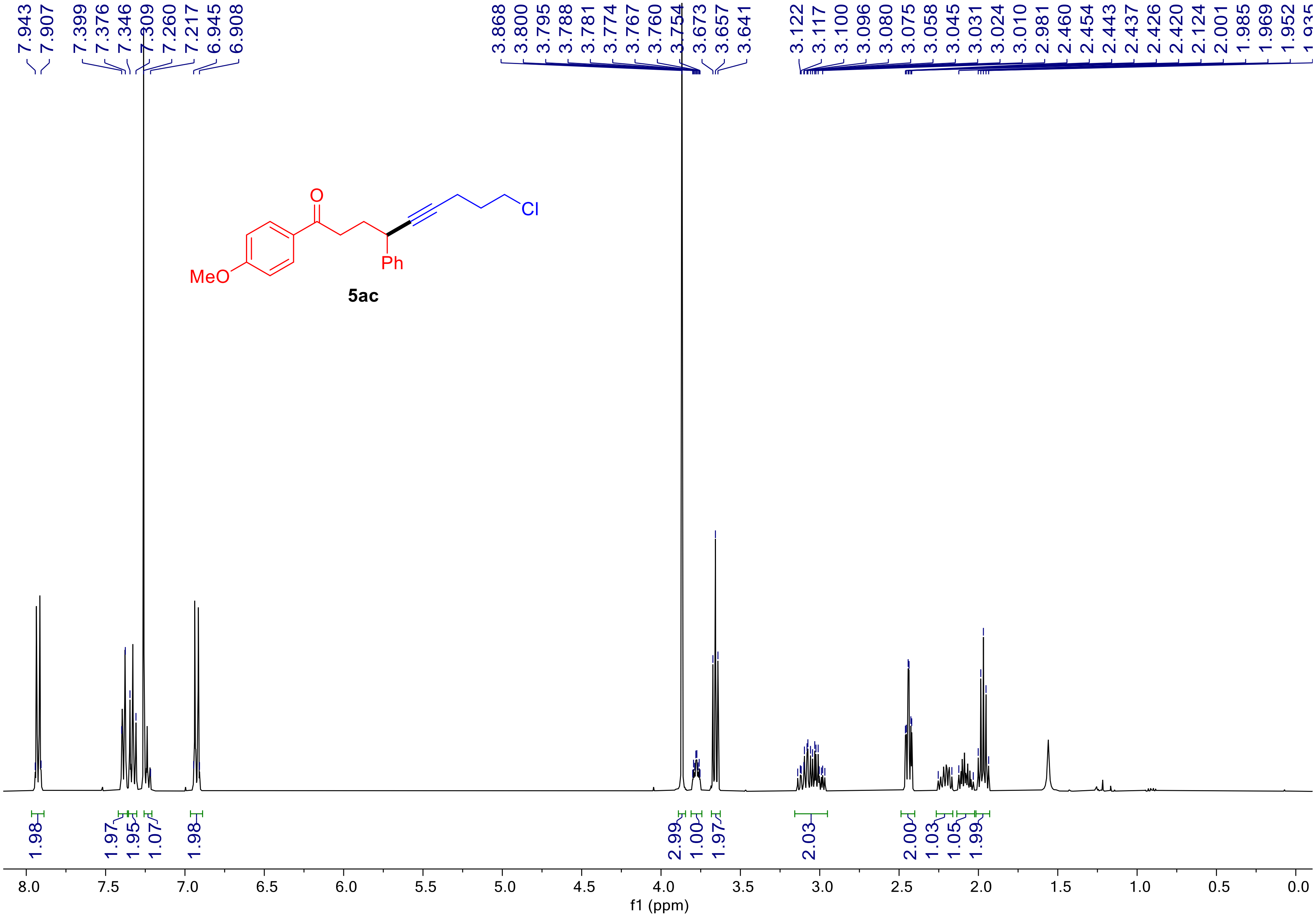

Supplementary Figure 193. ^{1}H NMR spectrum of 5ac

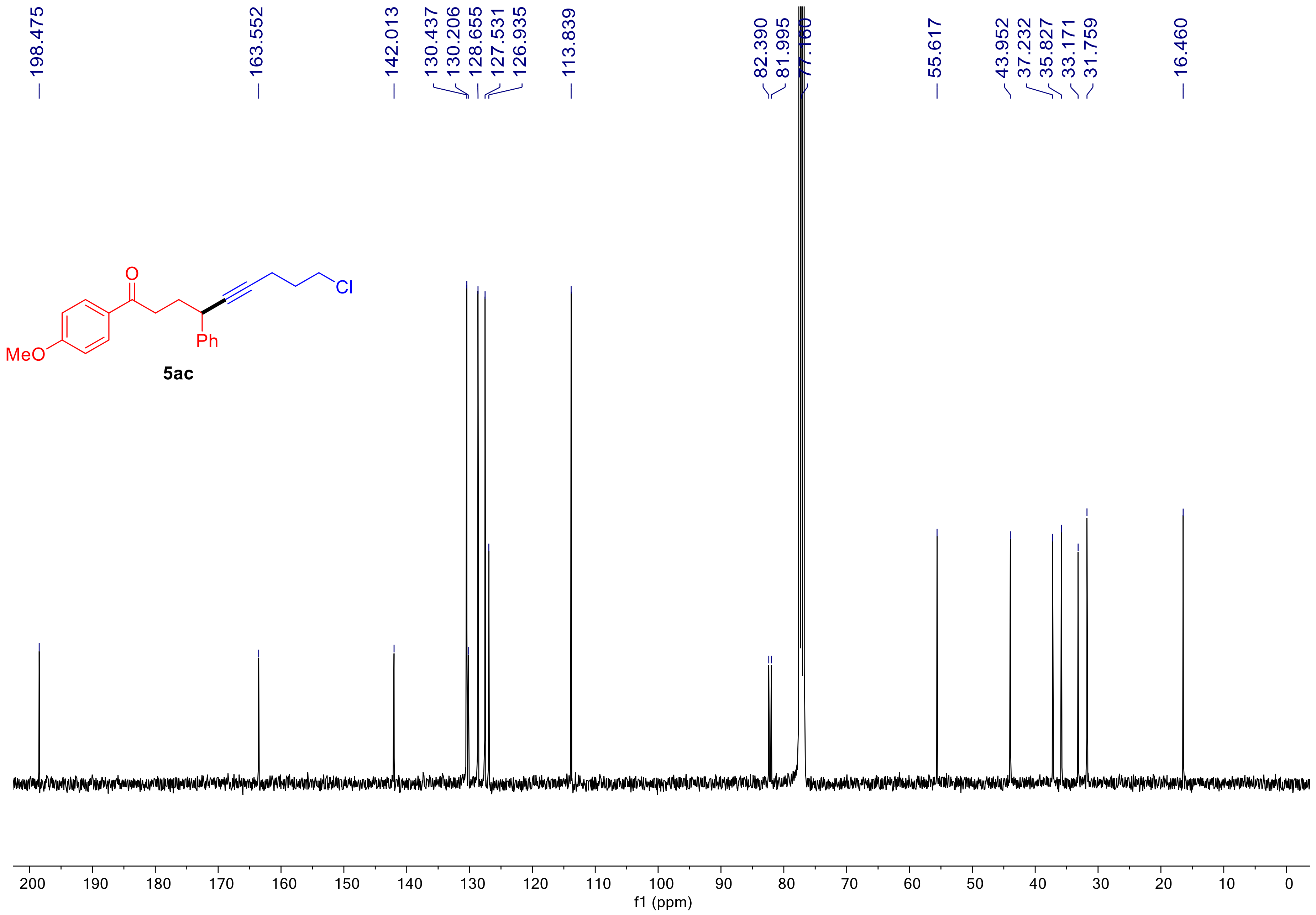

Supplementary Figure 194. ^{13}C NMR spectrum of 5ac

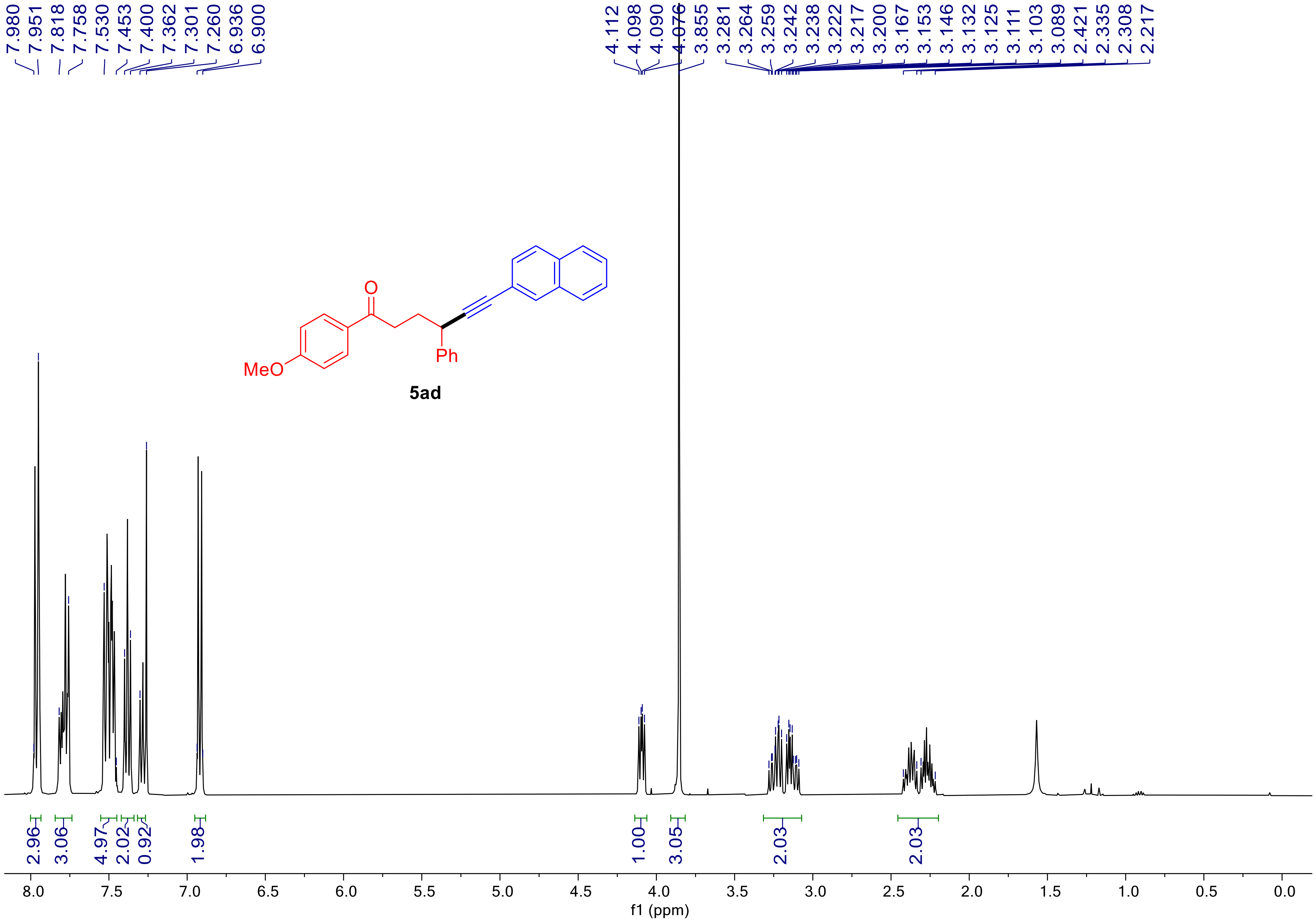

Supplementary Figure 195. ^{1}H NMR spectrum of 5ad

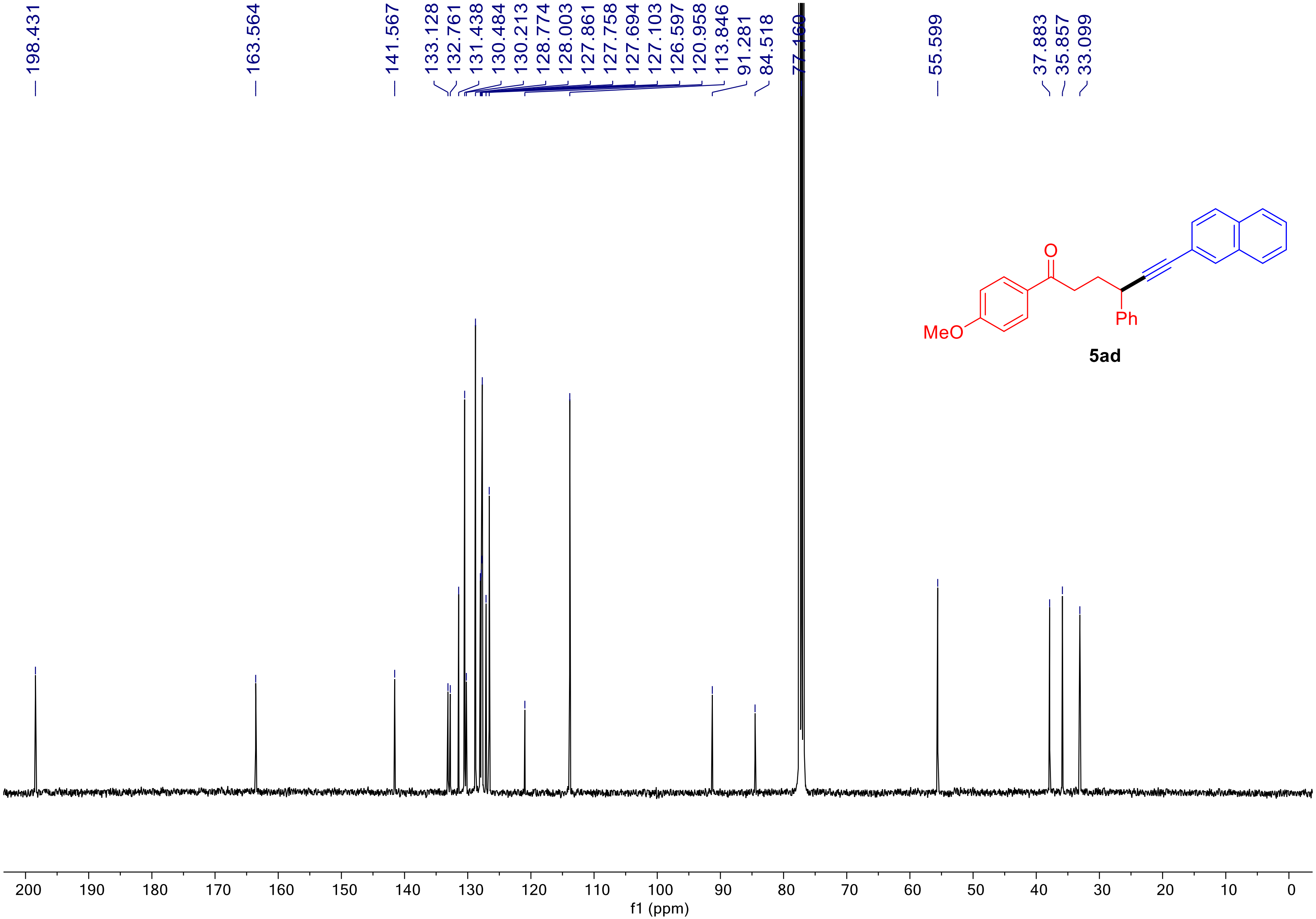

Supplementary Figure 196. ^{13}C NMR spectrum of 5ad

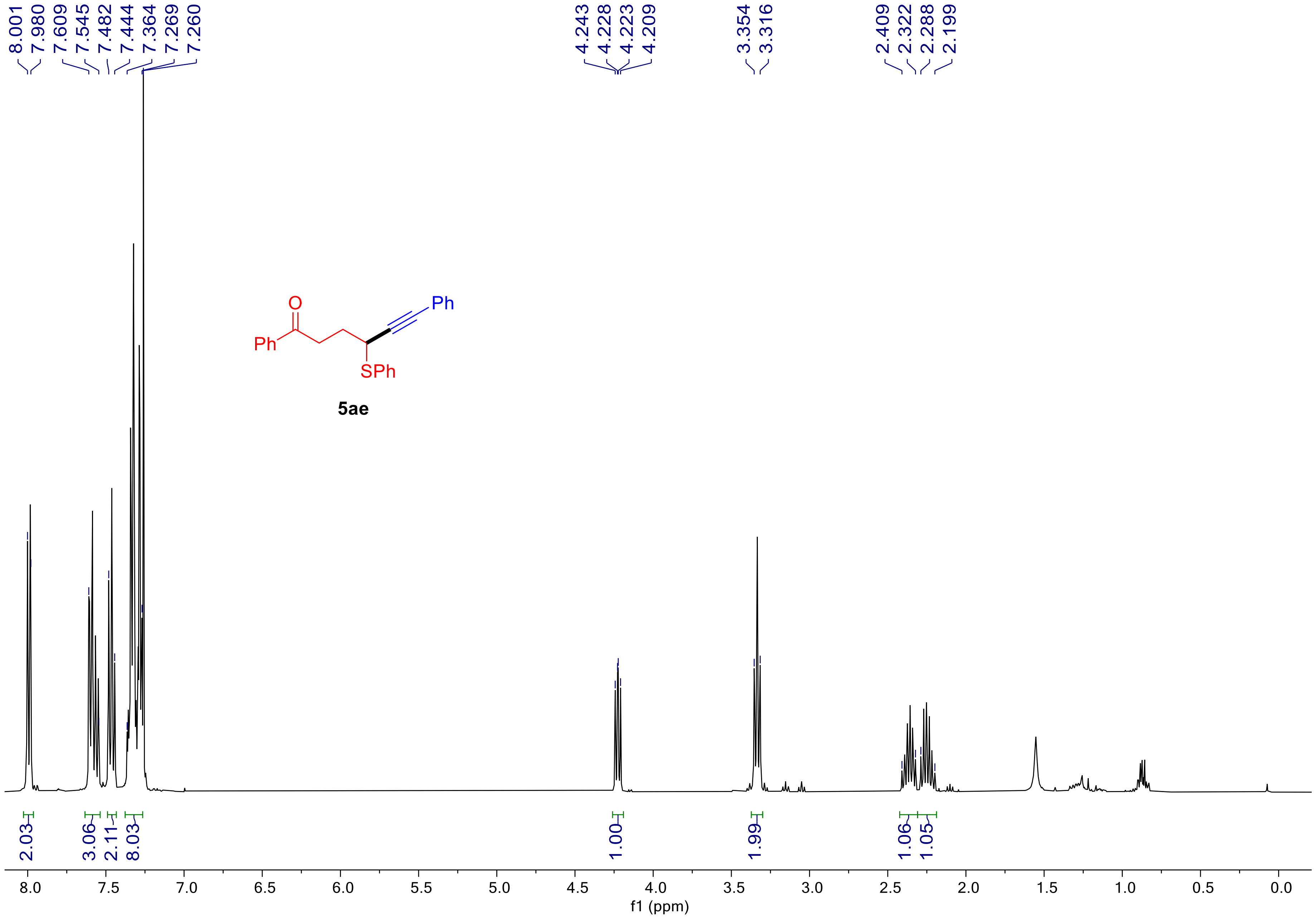

Supplementary Figure 197. ^{1}H NMR spectrum of 5ae

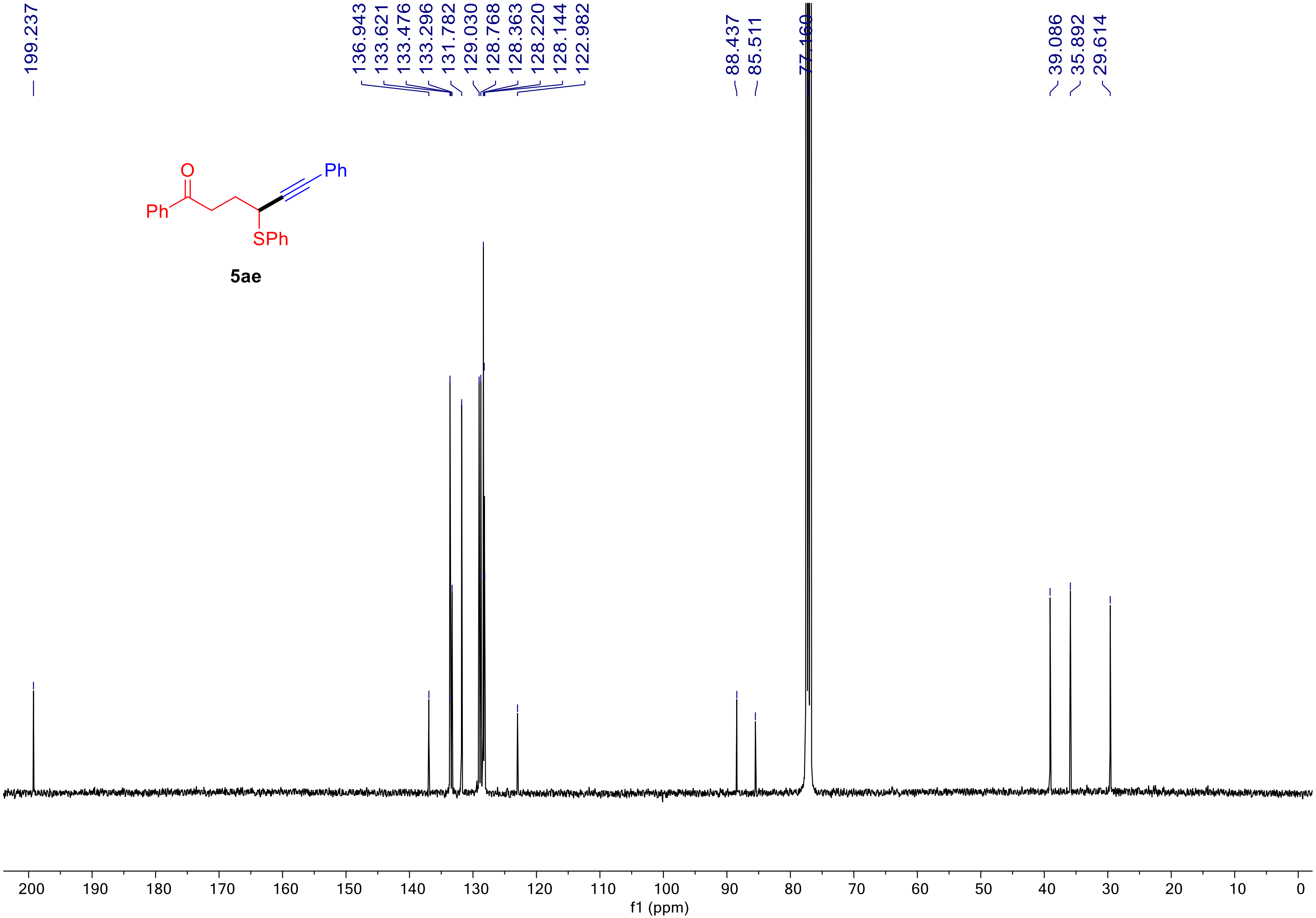

Supplementary Figure 198. ^{13}C NMR spectrum of 5ae

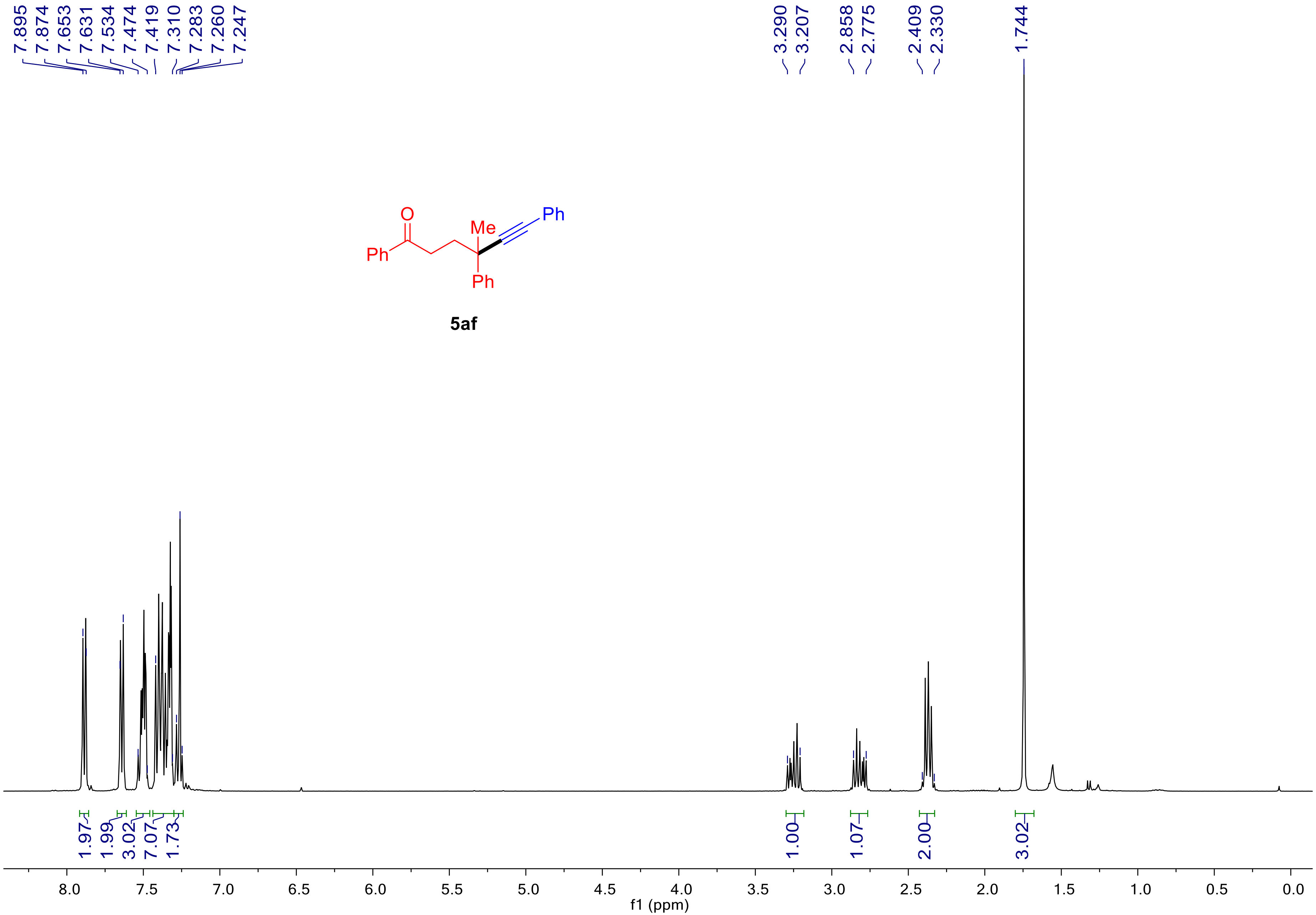

Supplementary Figure 199. ^{1}H NMR spectrum of 5af

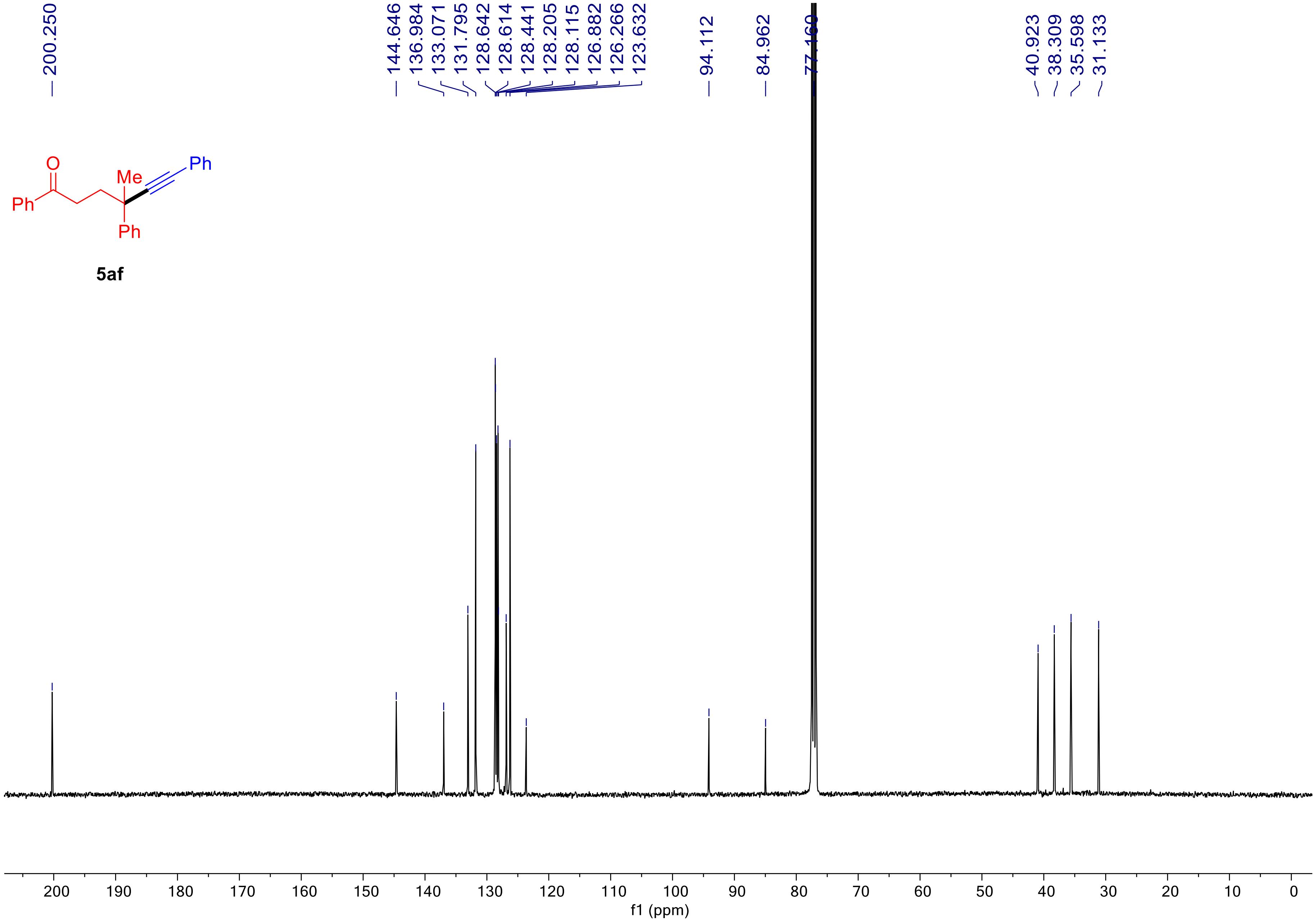

Supplementary Figure 200. ^{13}C **NMR spectrum of 5af**

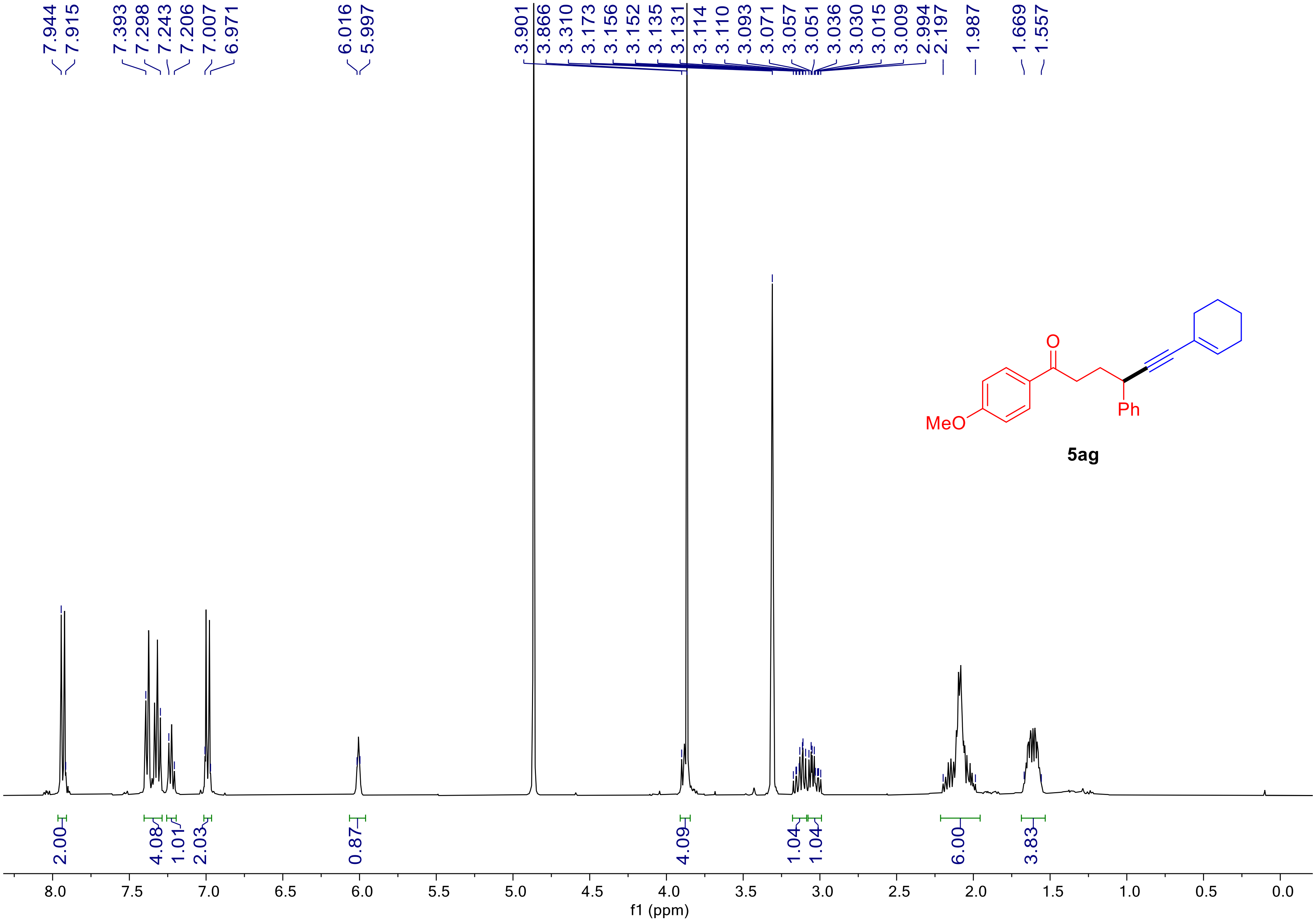

Supplementary Figure 201. ^{1}H NMR spectrum of 5ag

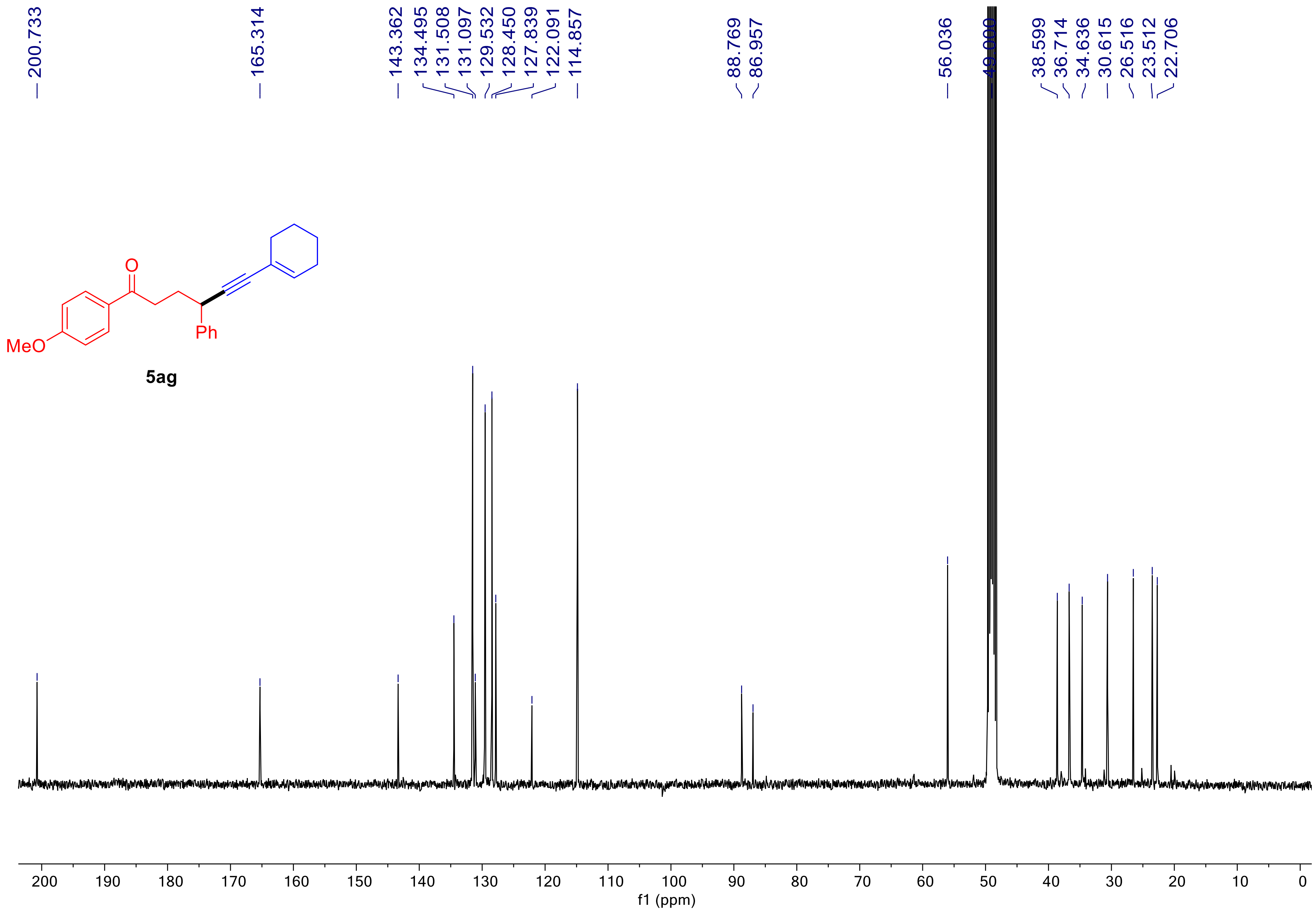

Supplementary Figure 202. ^{13}C NMR spectrum of 5ag

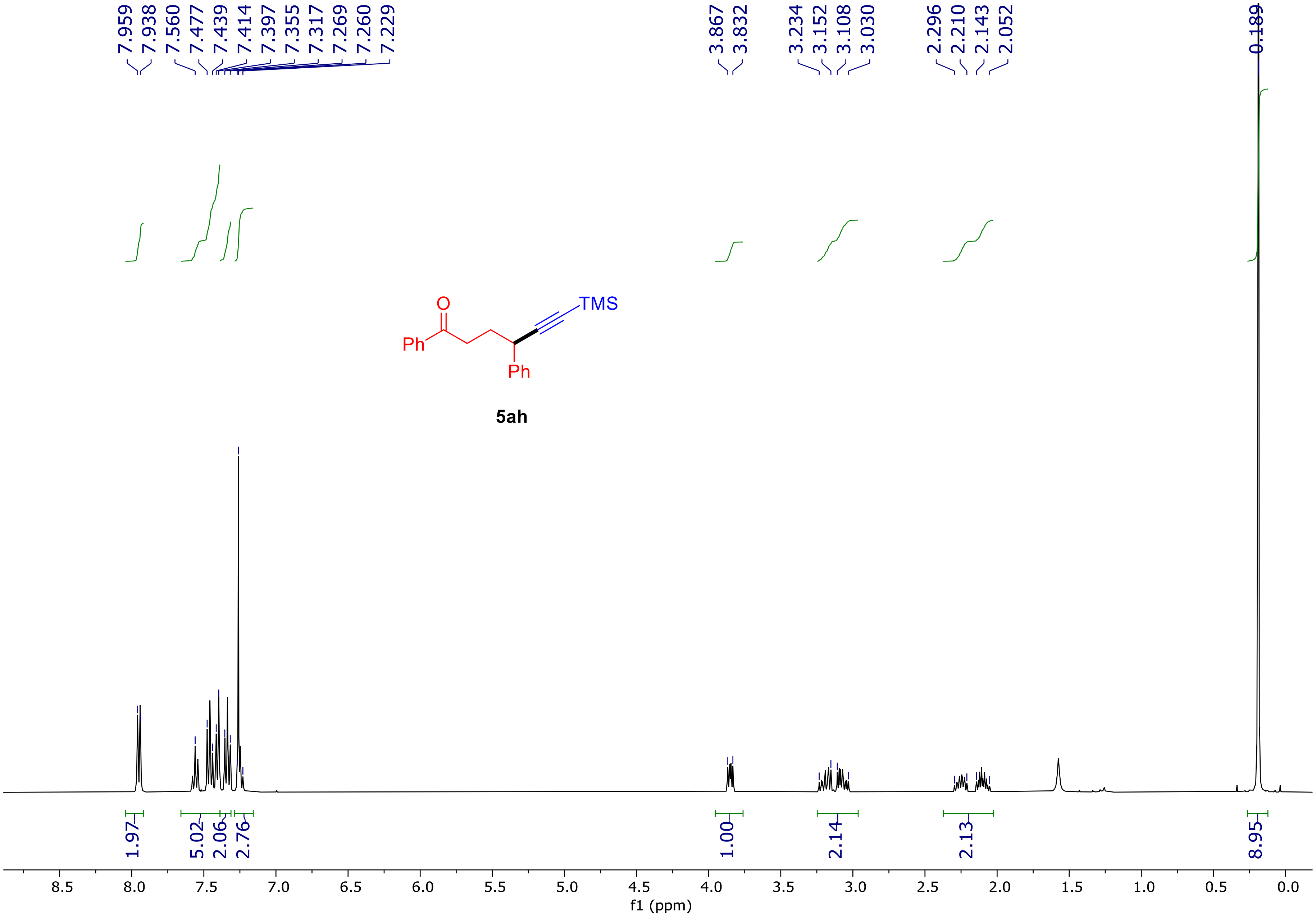

Supplementary Figure 203. ^{1}H NMR spectrum of 5ah

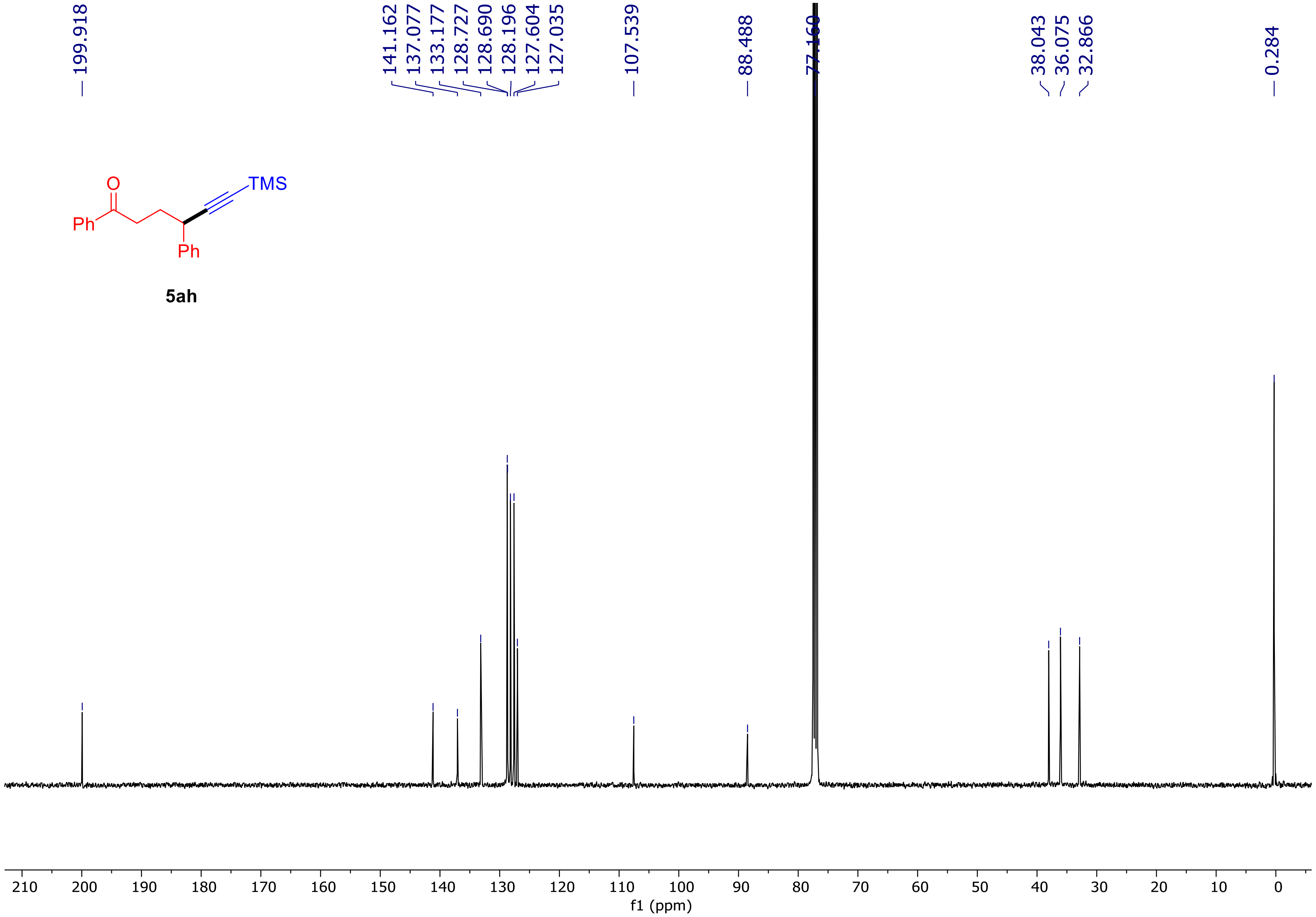

Supplementary Figure 204. ^{13}C NMR spectrum of 5ah

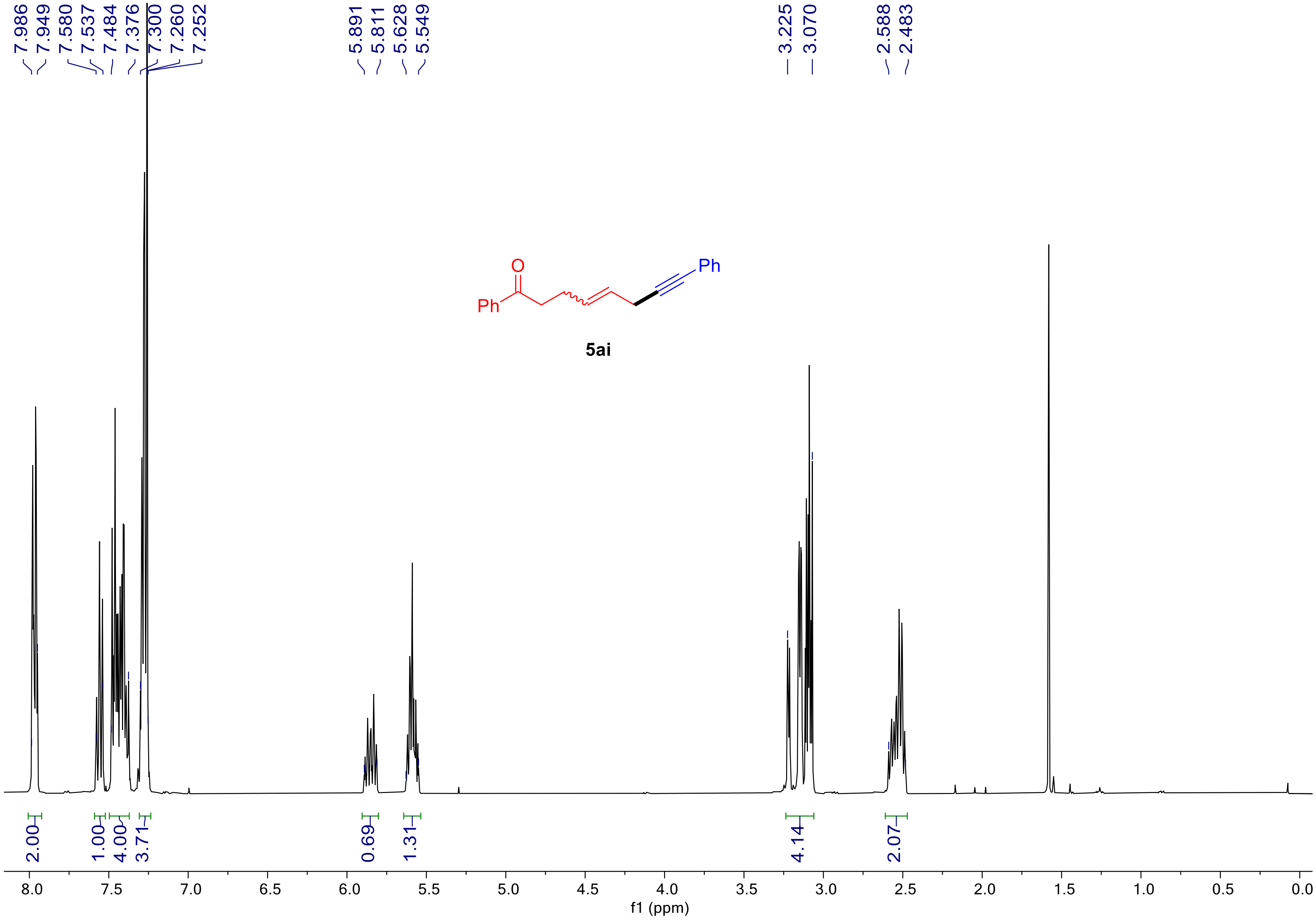

Supplementary Figure 205. ^{1}H NMR spectrum of 5ai

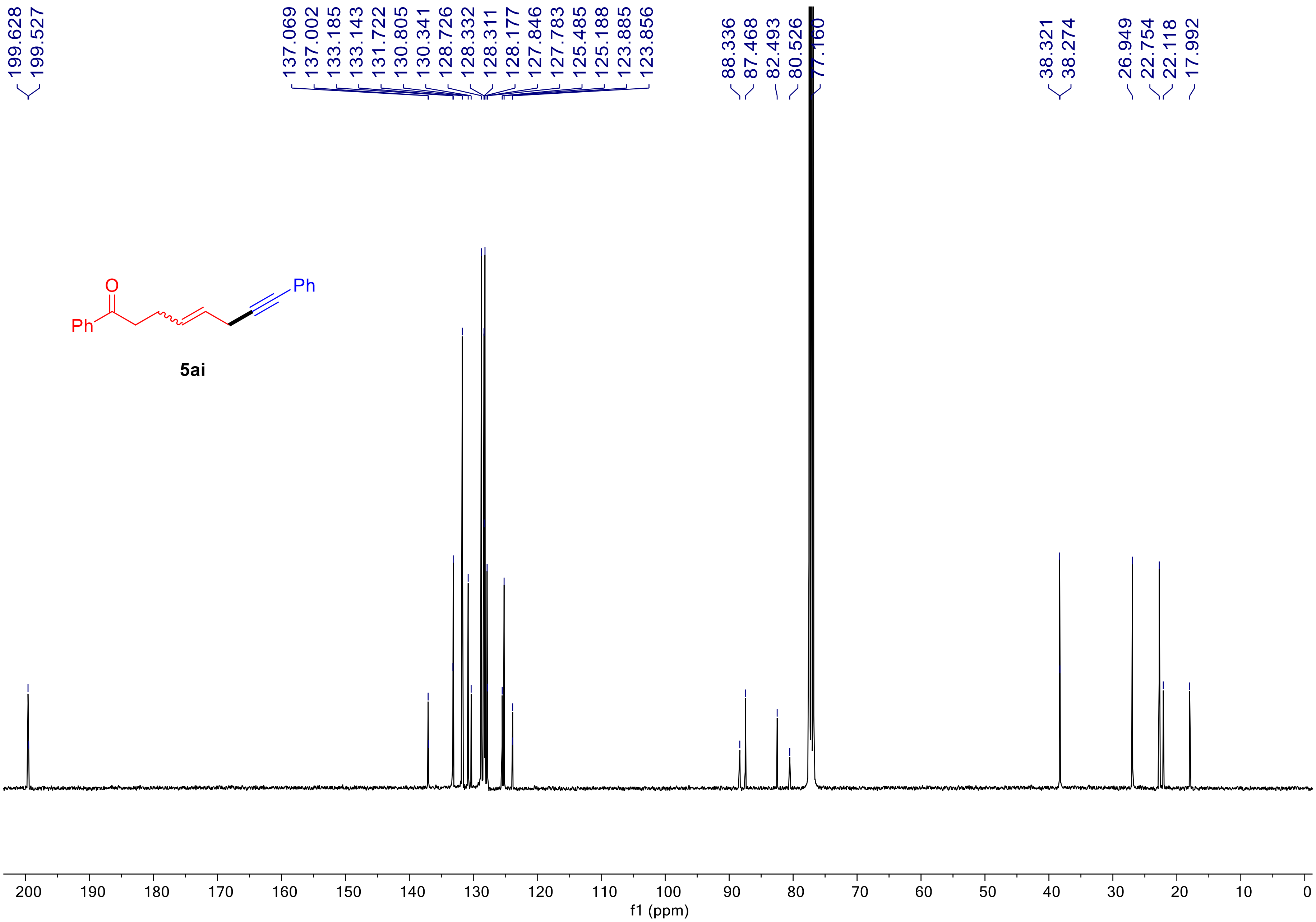

Supplementary Figure 206. ^{13}C NMR spectrum of 5ai

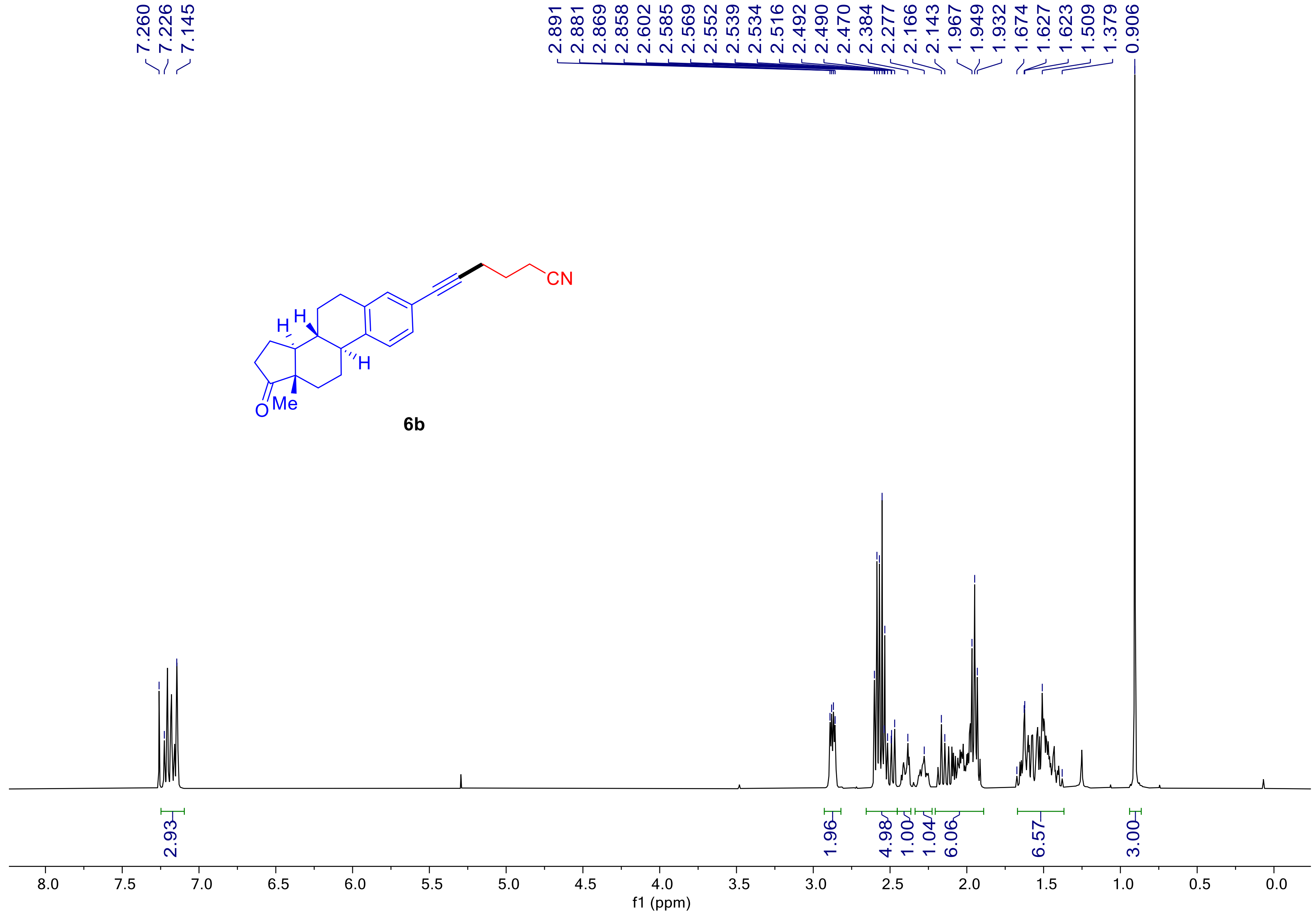

Supplementary Figure 207. ^{1}H NMR spectrum of 6b

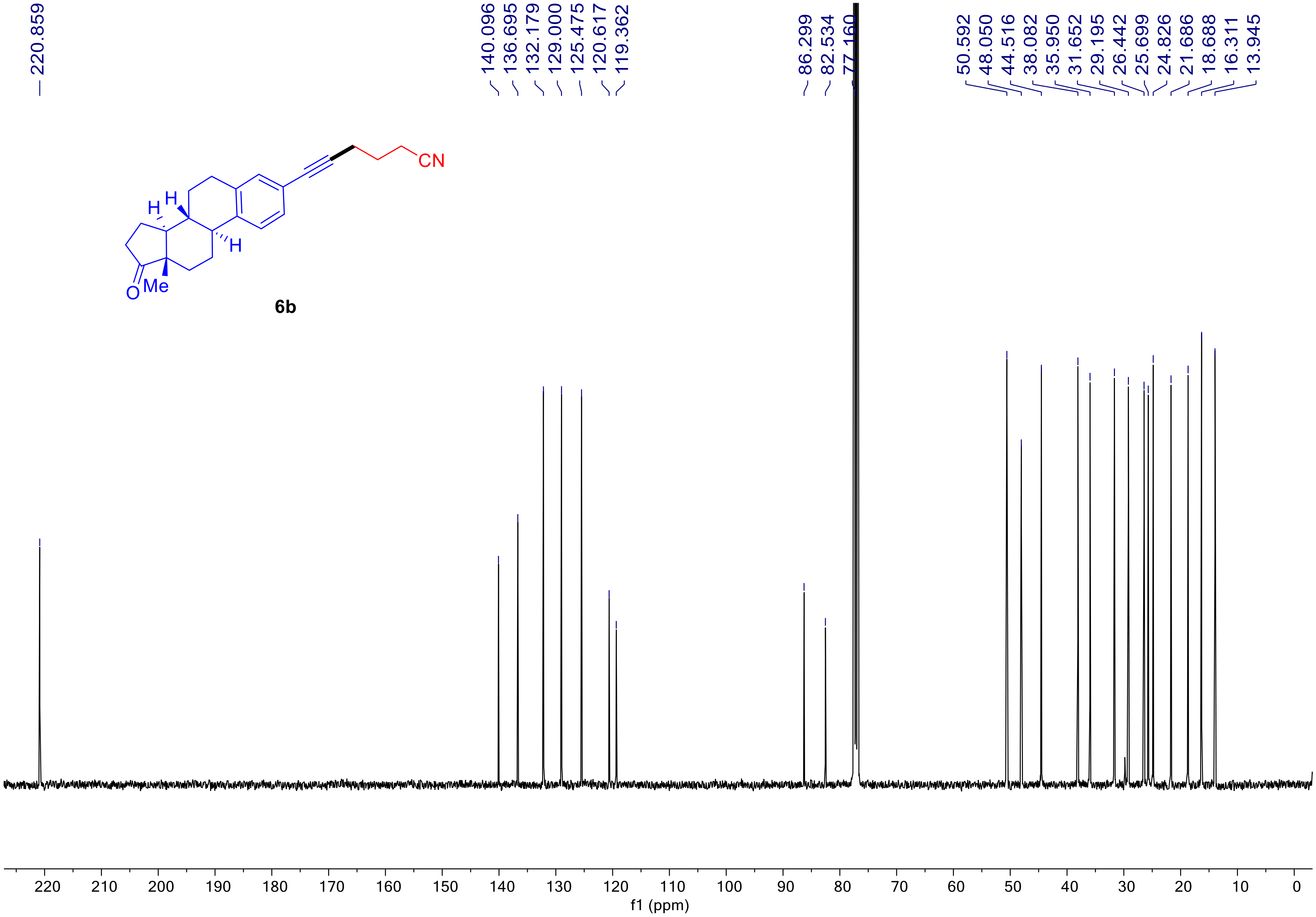

Supplementary Figure 208. ^{13}C NMR spectrum of 6b

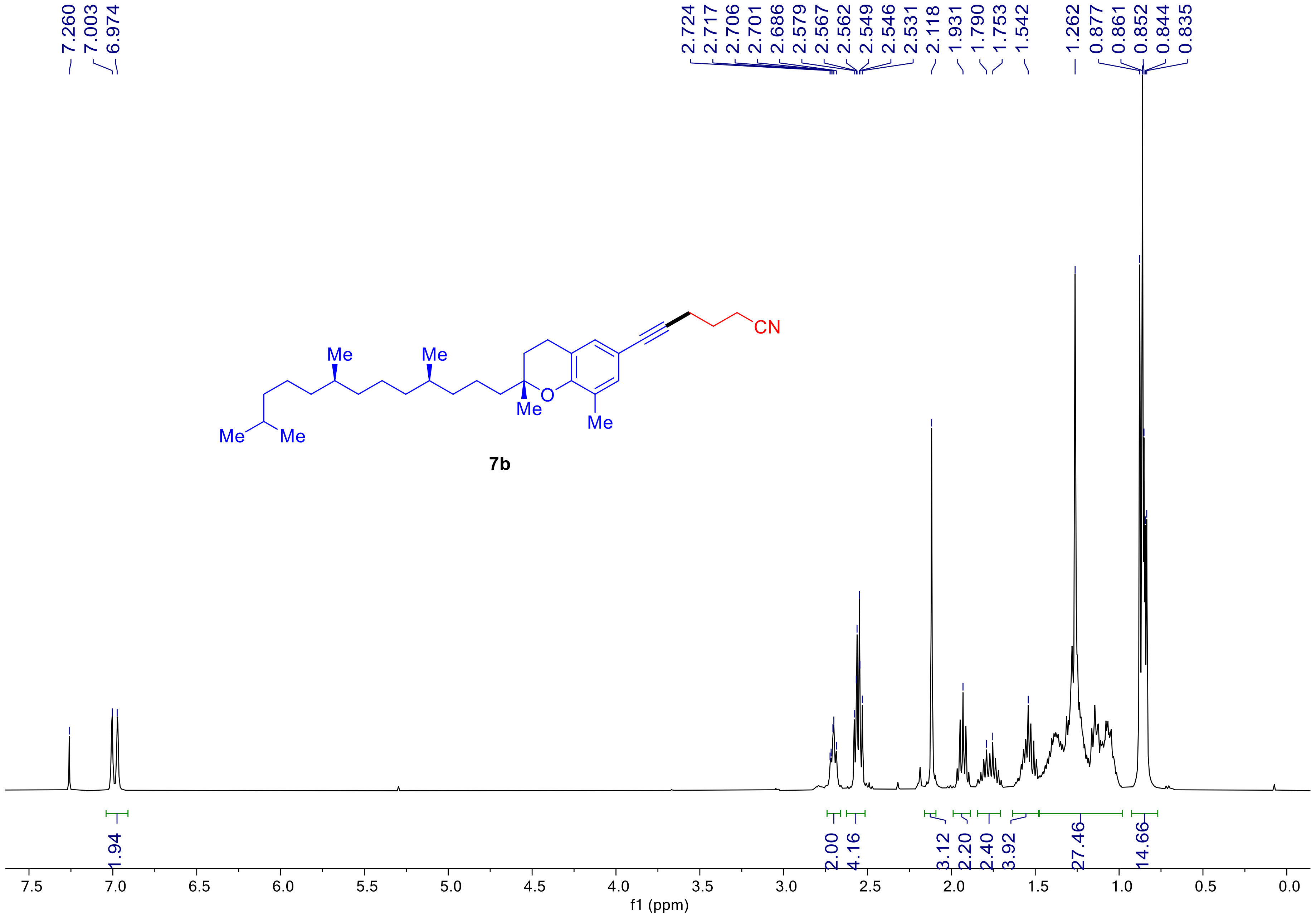

Supplementary Figure 209. ^{1}H NMR spectrum of 7b

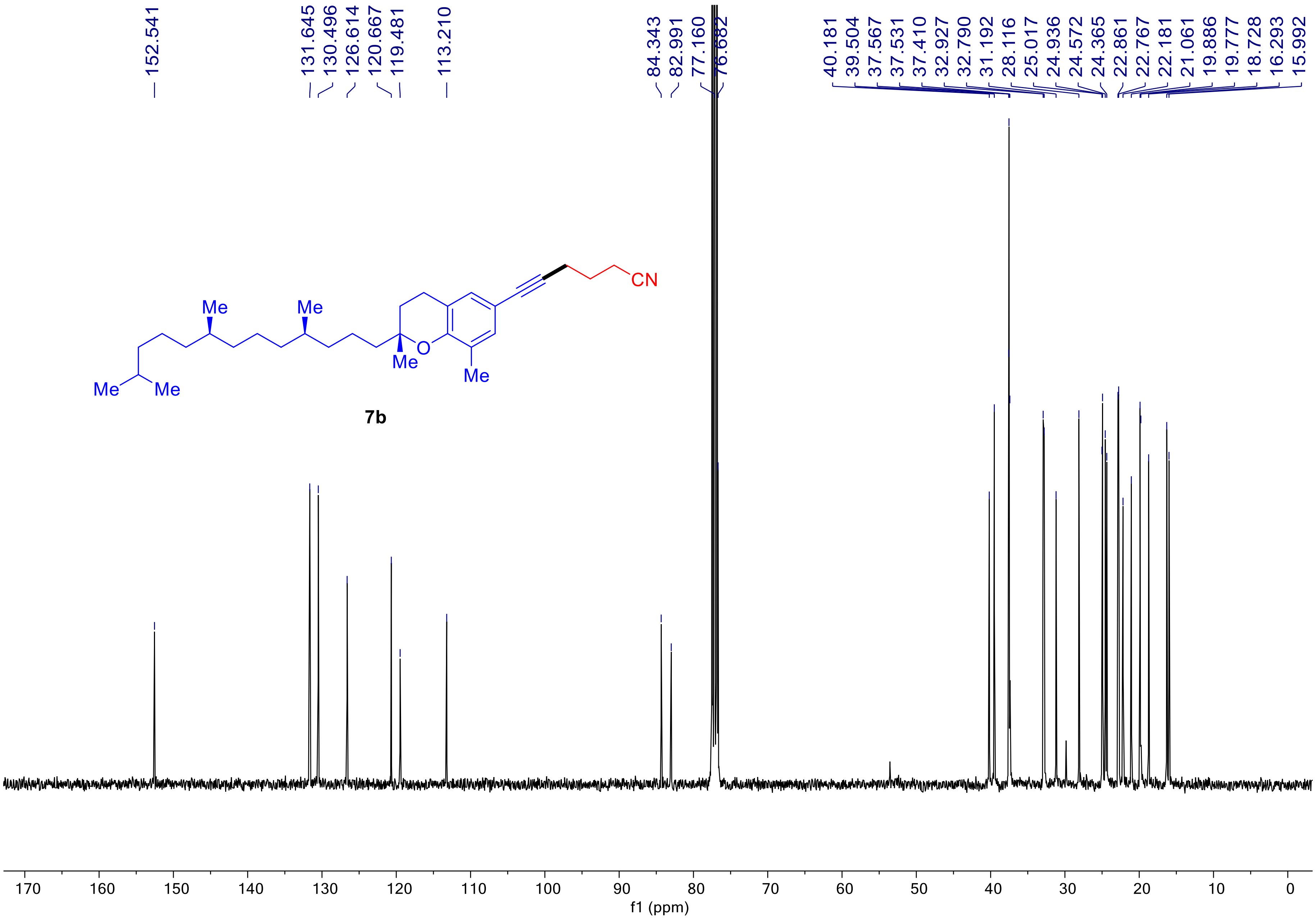

Supplementary Figure 210. ^{13}C NMR spectrum of 7b

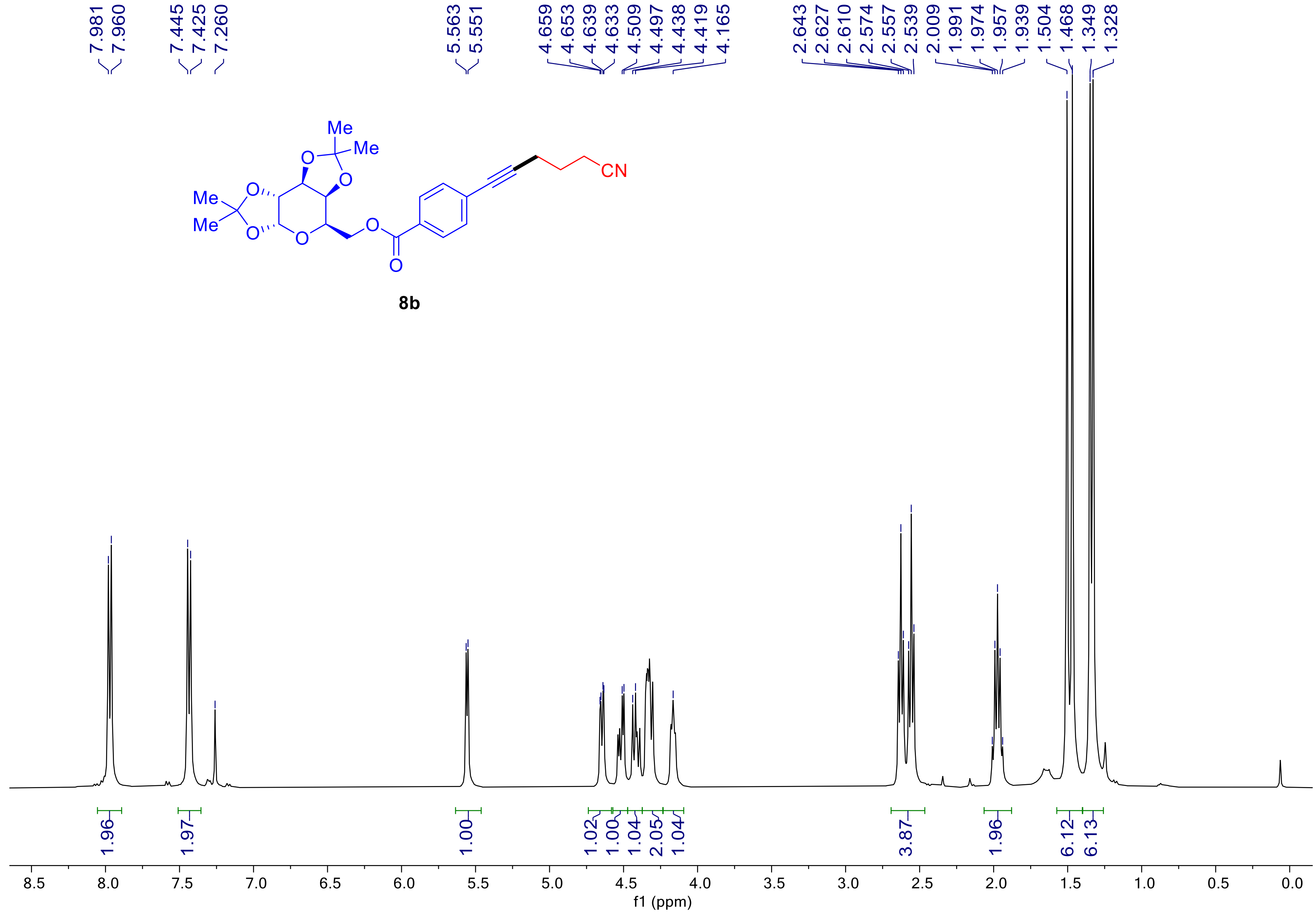

Supplementary Figure 211. ^{1}**H NMR spectrum of 8b**

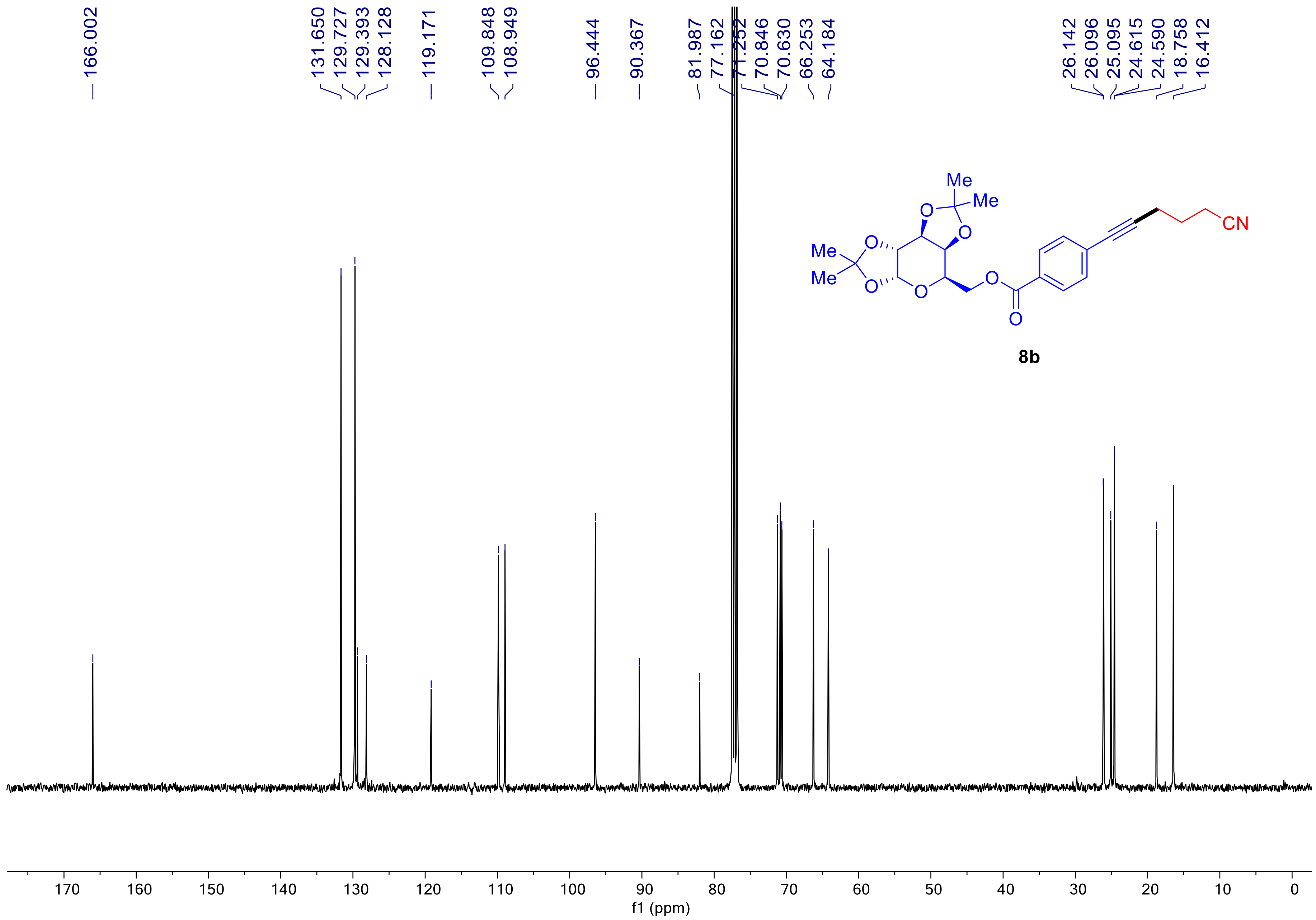

Supplementary Figure 212. ^{13}C NMR spectrum of 8b

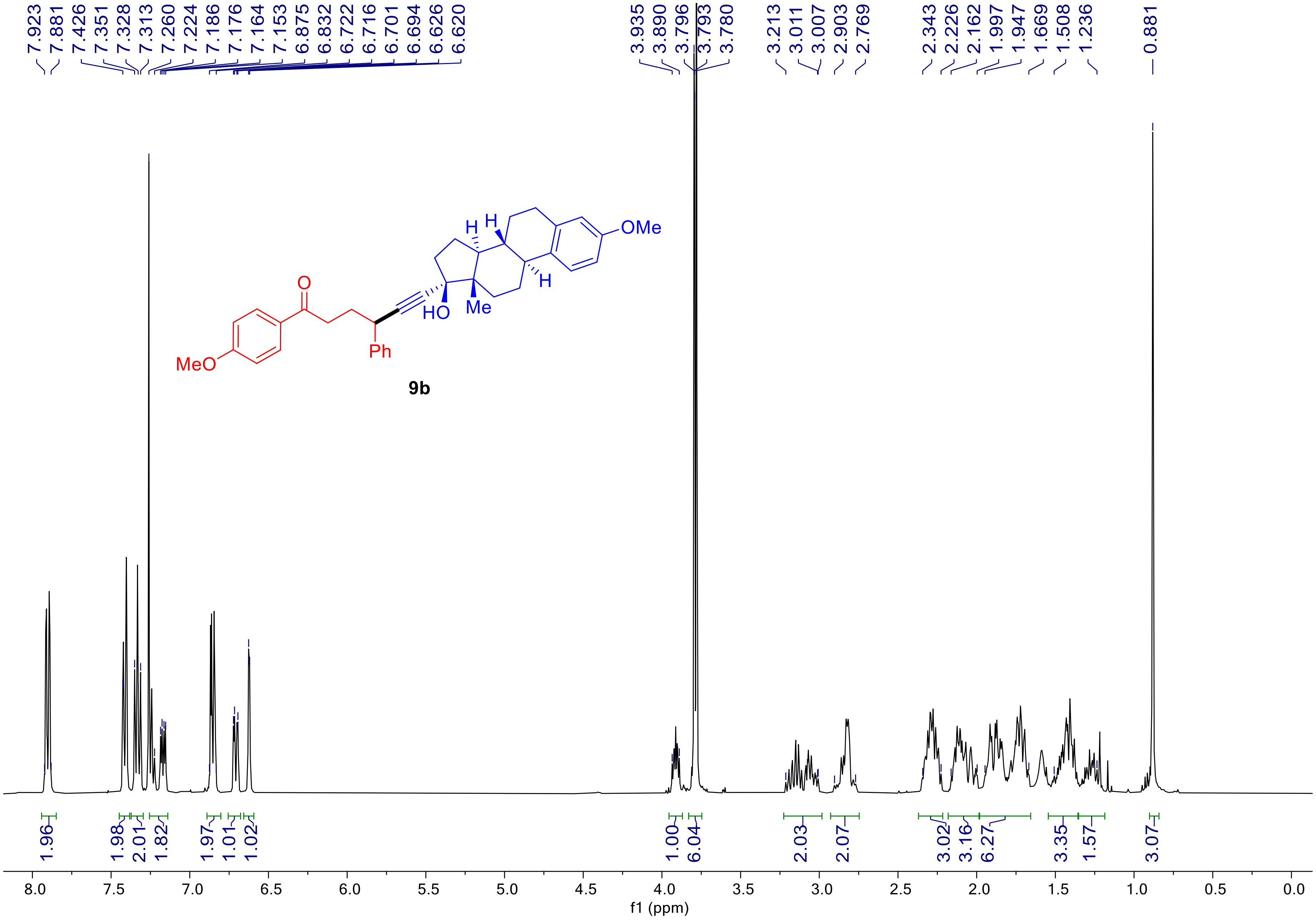

Supplementary Figure 213. ^{1}H NMR spectrum of 9b

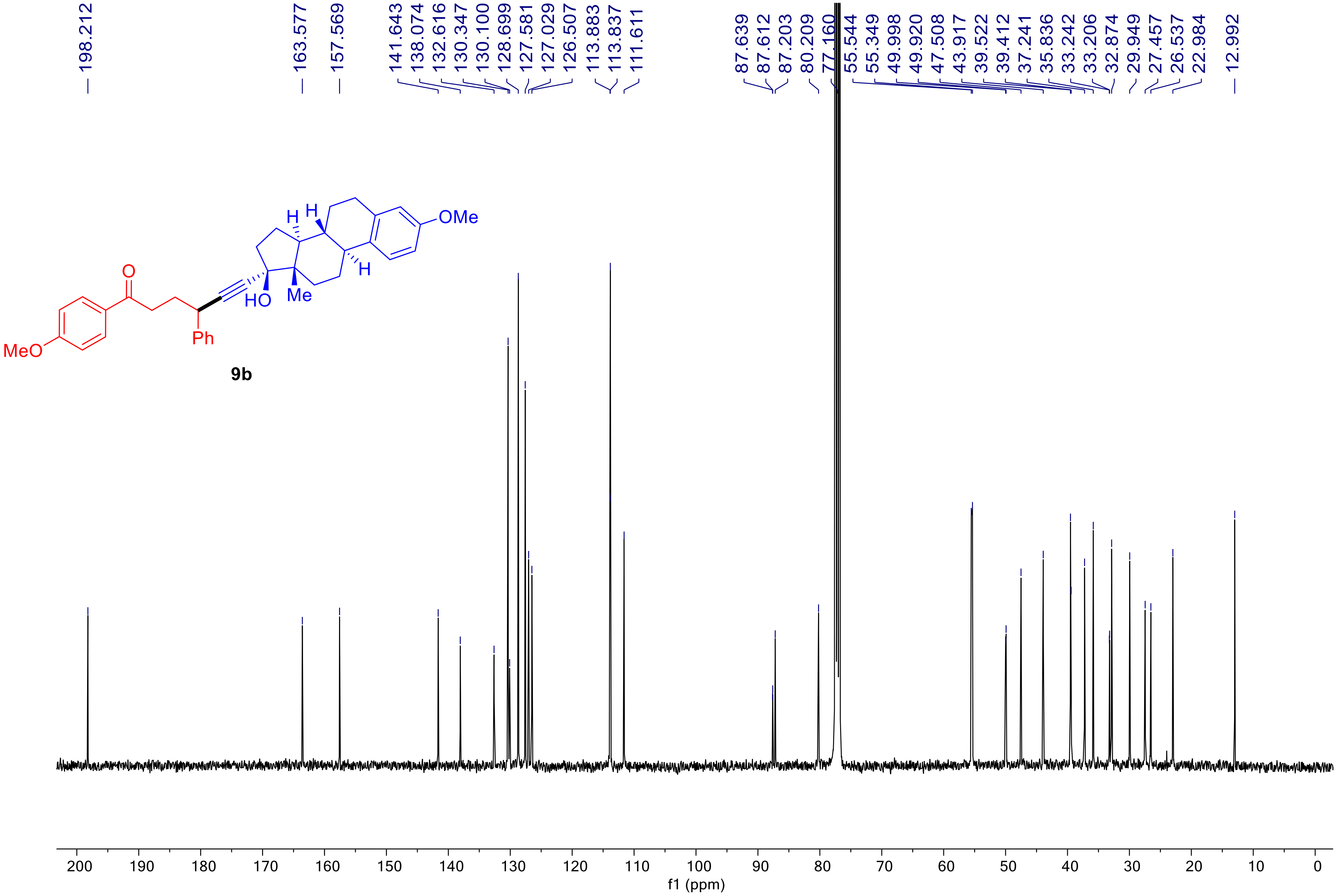

Supplementary Figure 214. ^{13}**C NMR spectrum of 9b**

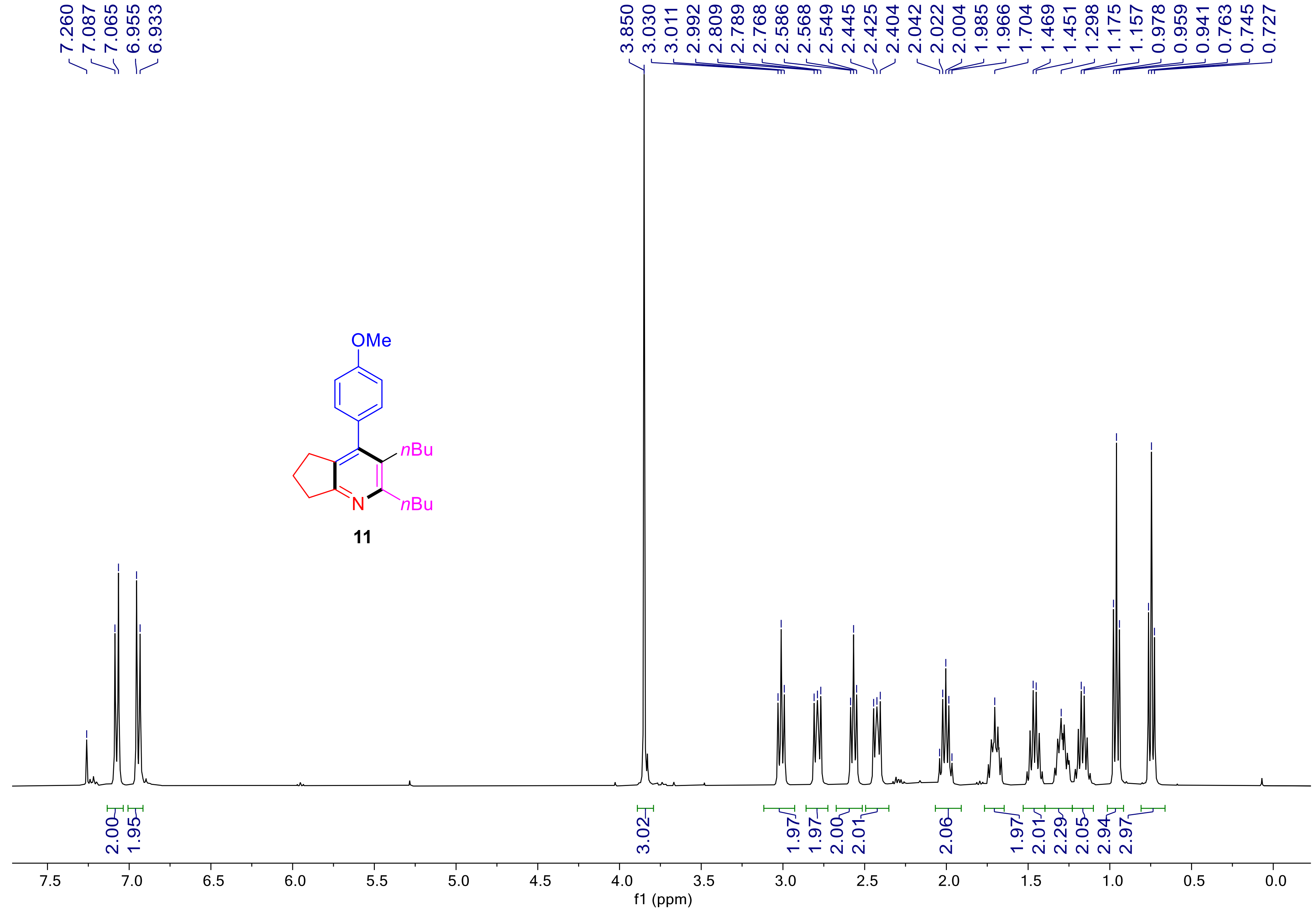

Supplementary Figure 215. ^{1}H NMR spectrum of 11

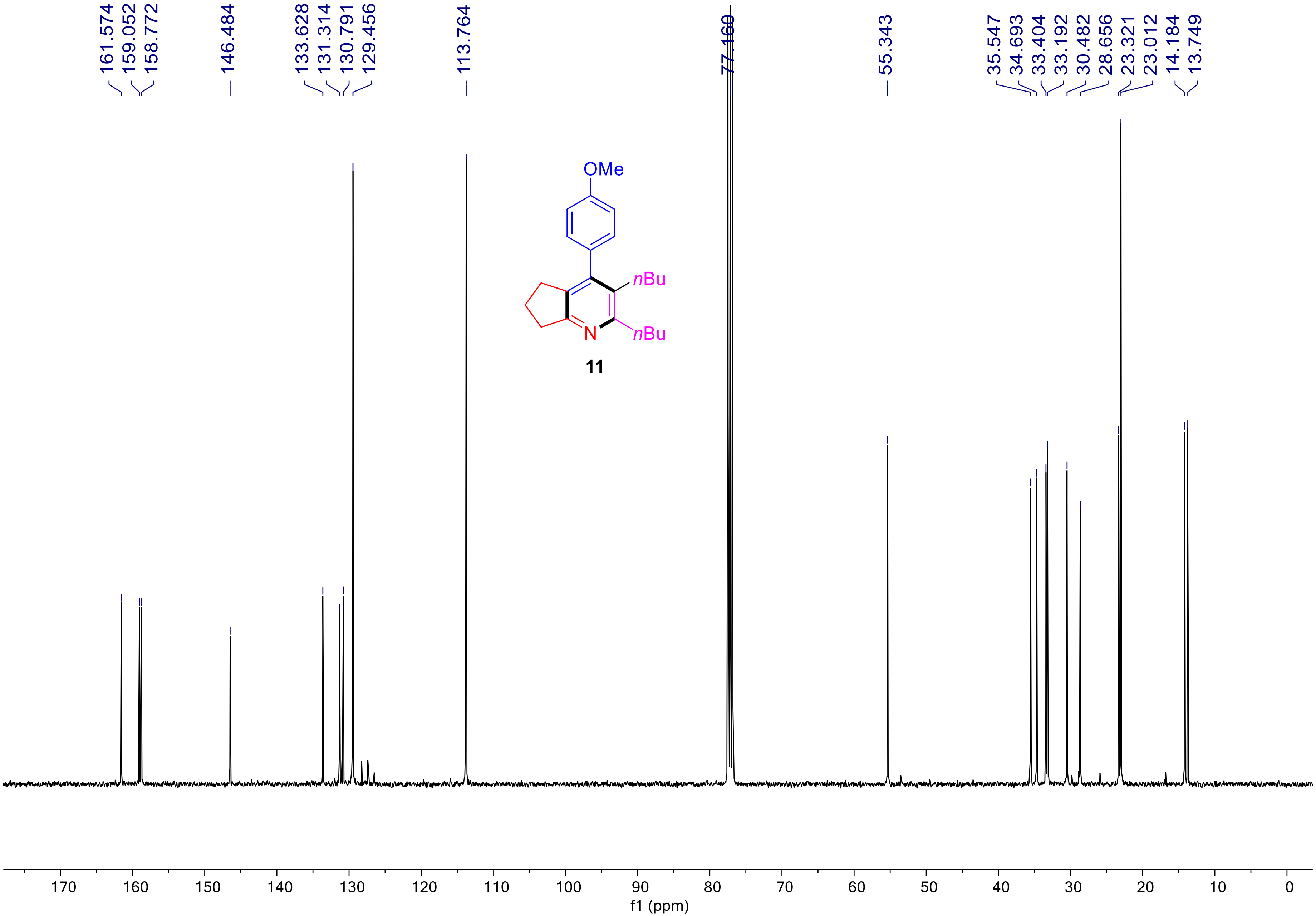

Supplementary Figure 216. ^{13}**C NMR spectrum of 11**

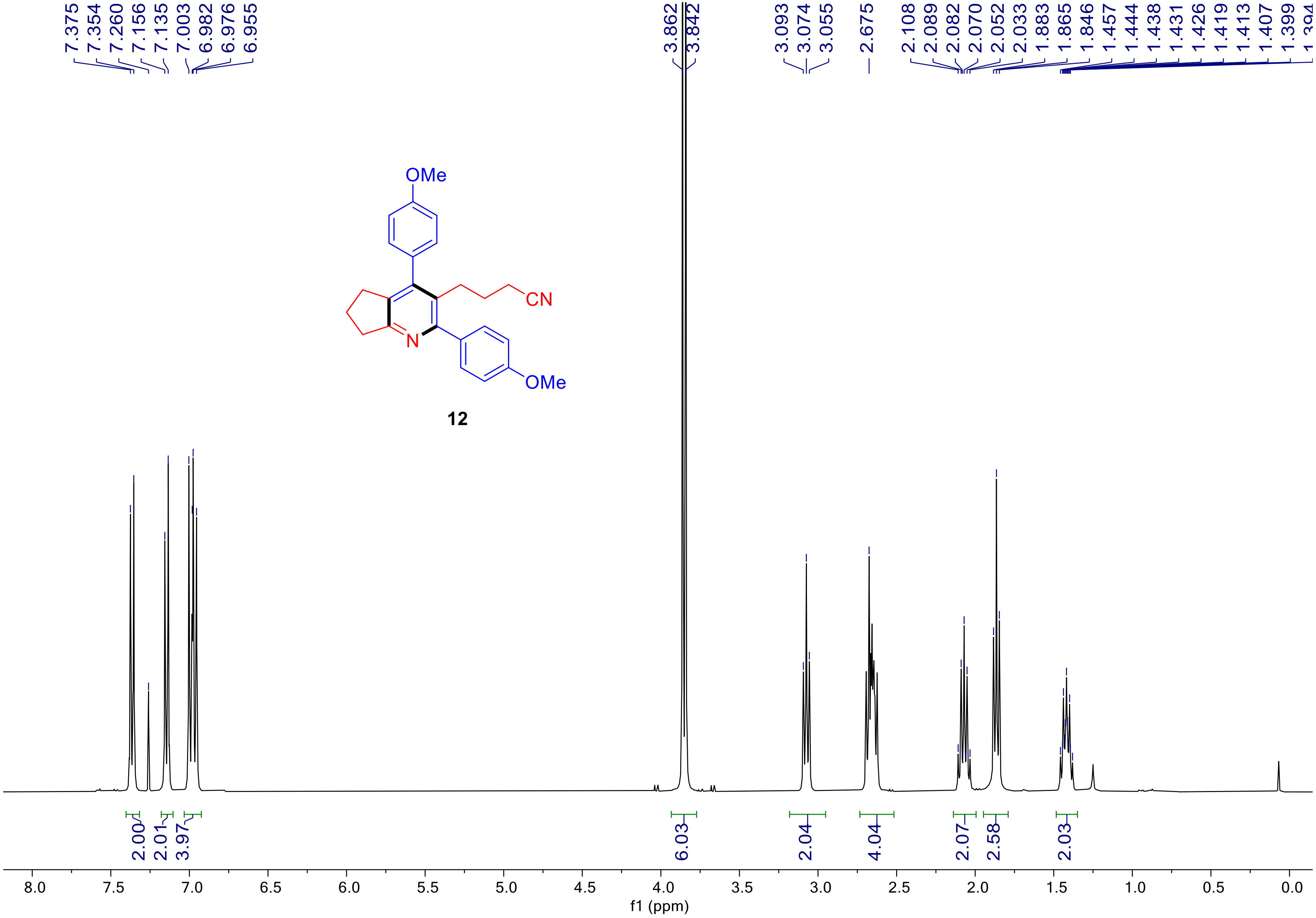

Supplementary Figure 217. ^{1}**H NMR spectrum of 12**

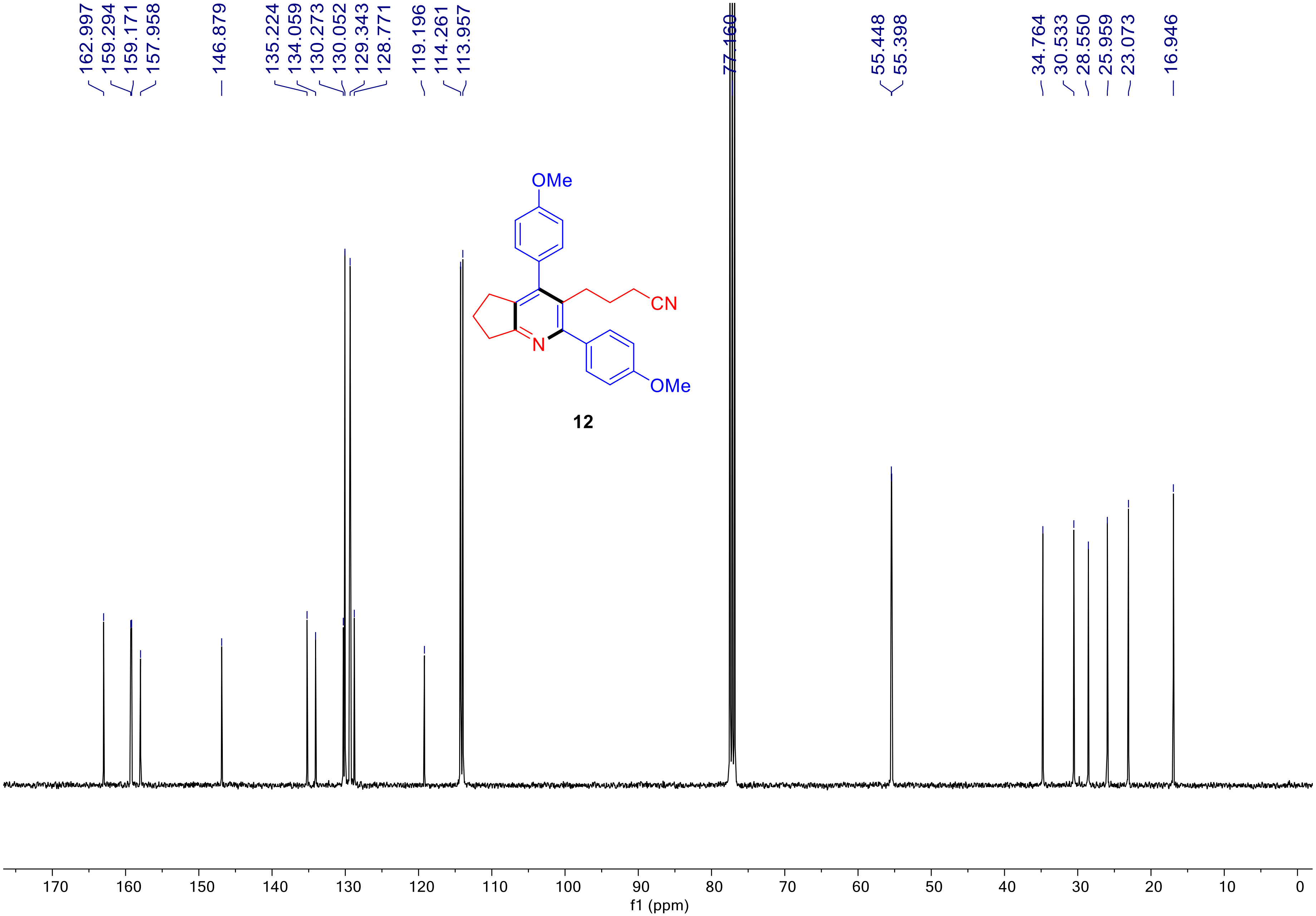

Supplementary Figure 218. ^{13}C NMR spectrum of 12

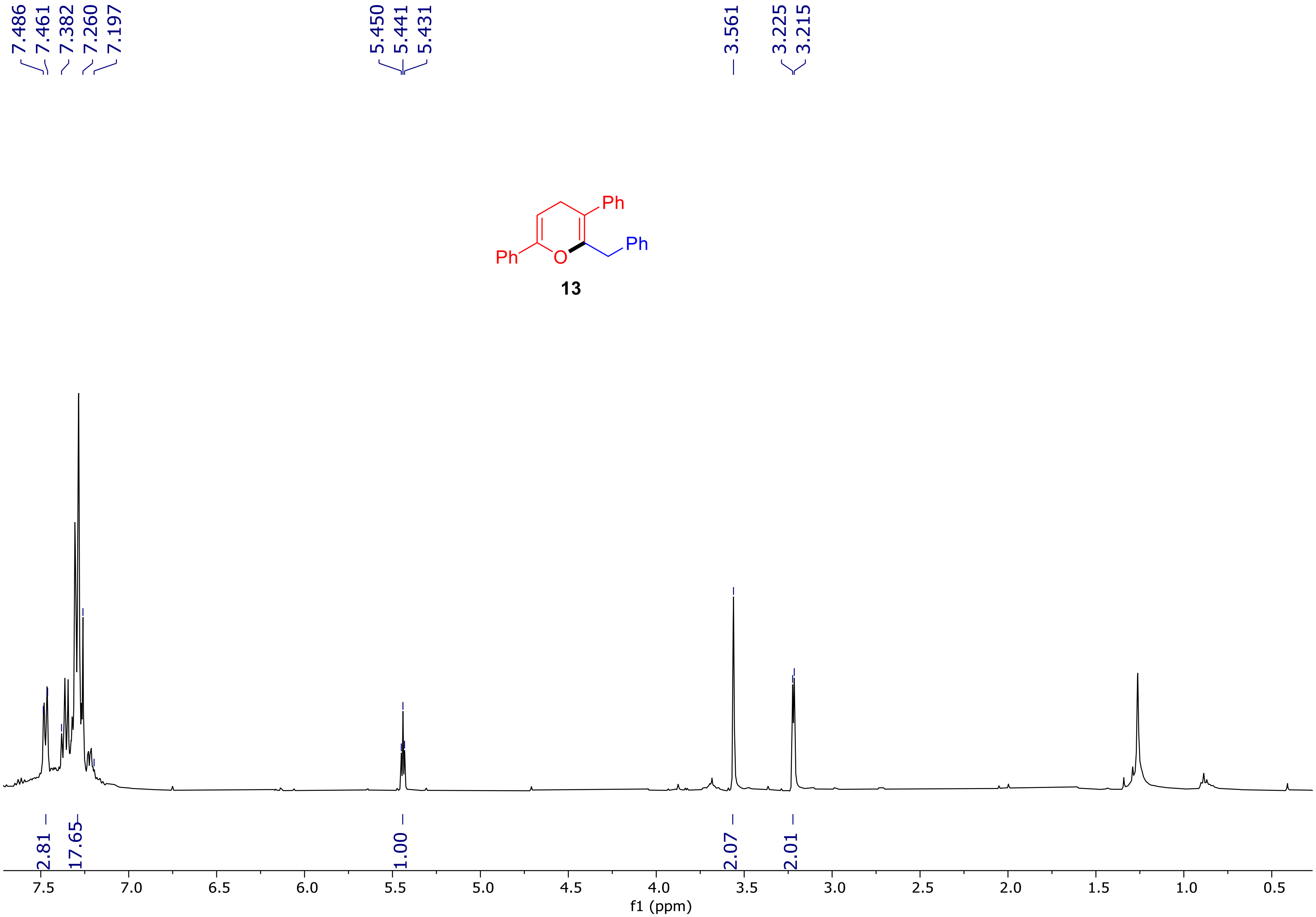

Supplementary Figure 219. ^{1}H NMR spectrum of 13

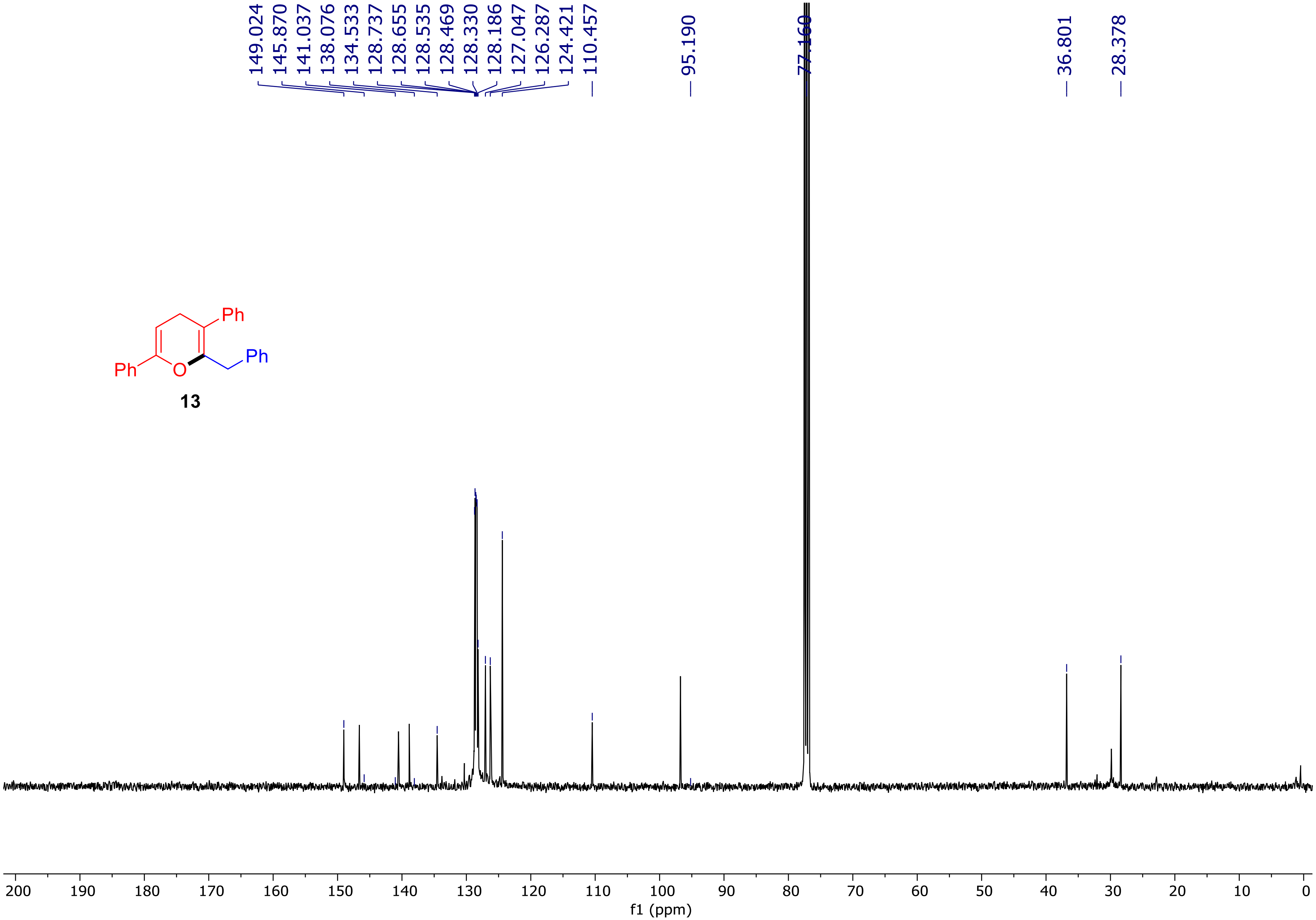

Supplementary Figure 220. ^{13}C NMR spectrum of 13

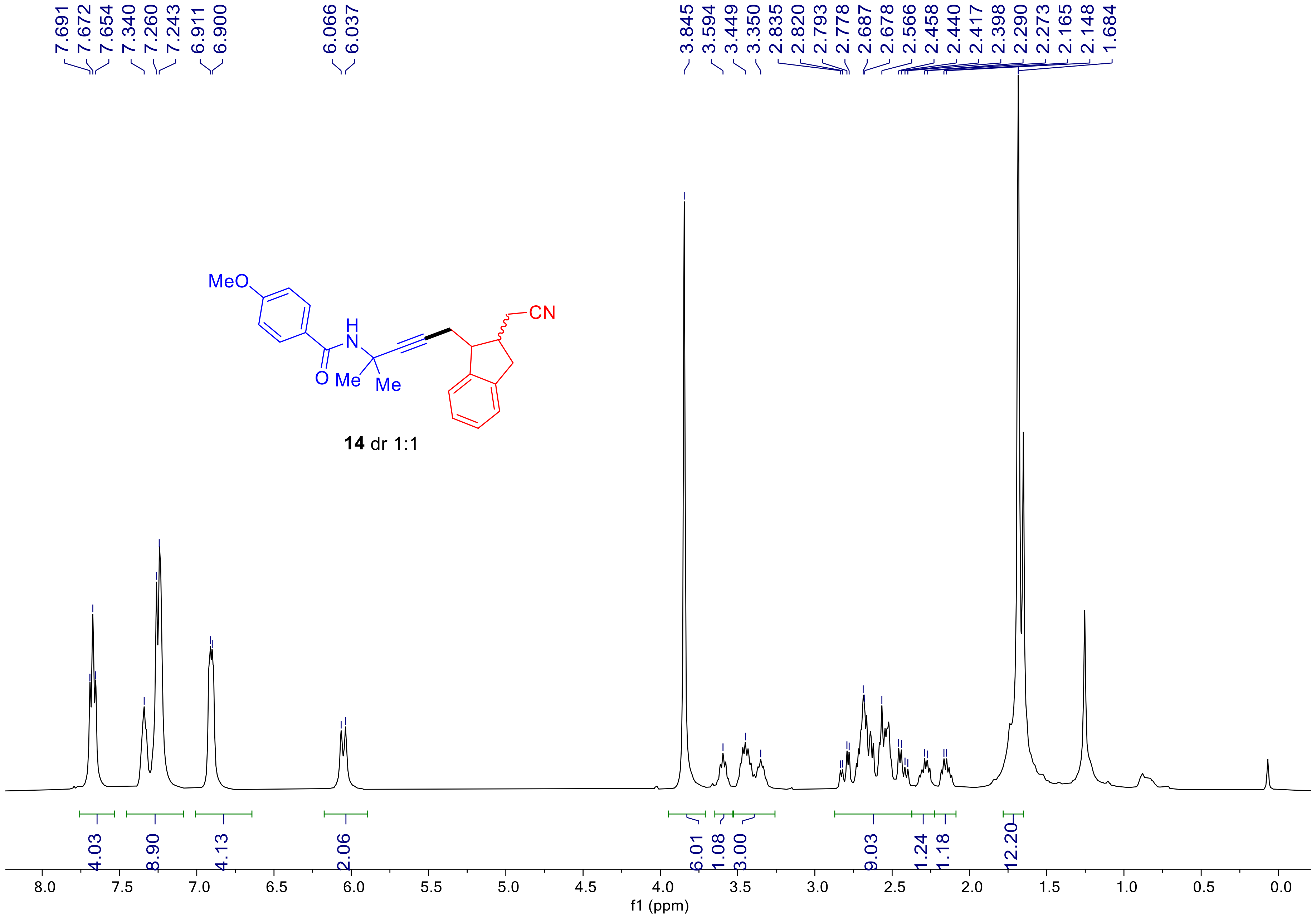

Supplementary Figure 221. ¹H NMR spectrum of 14

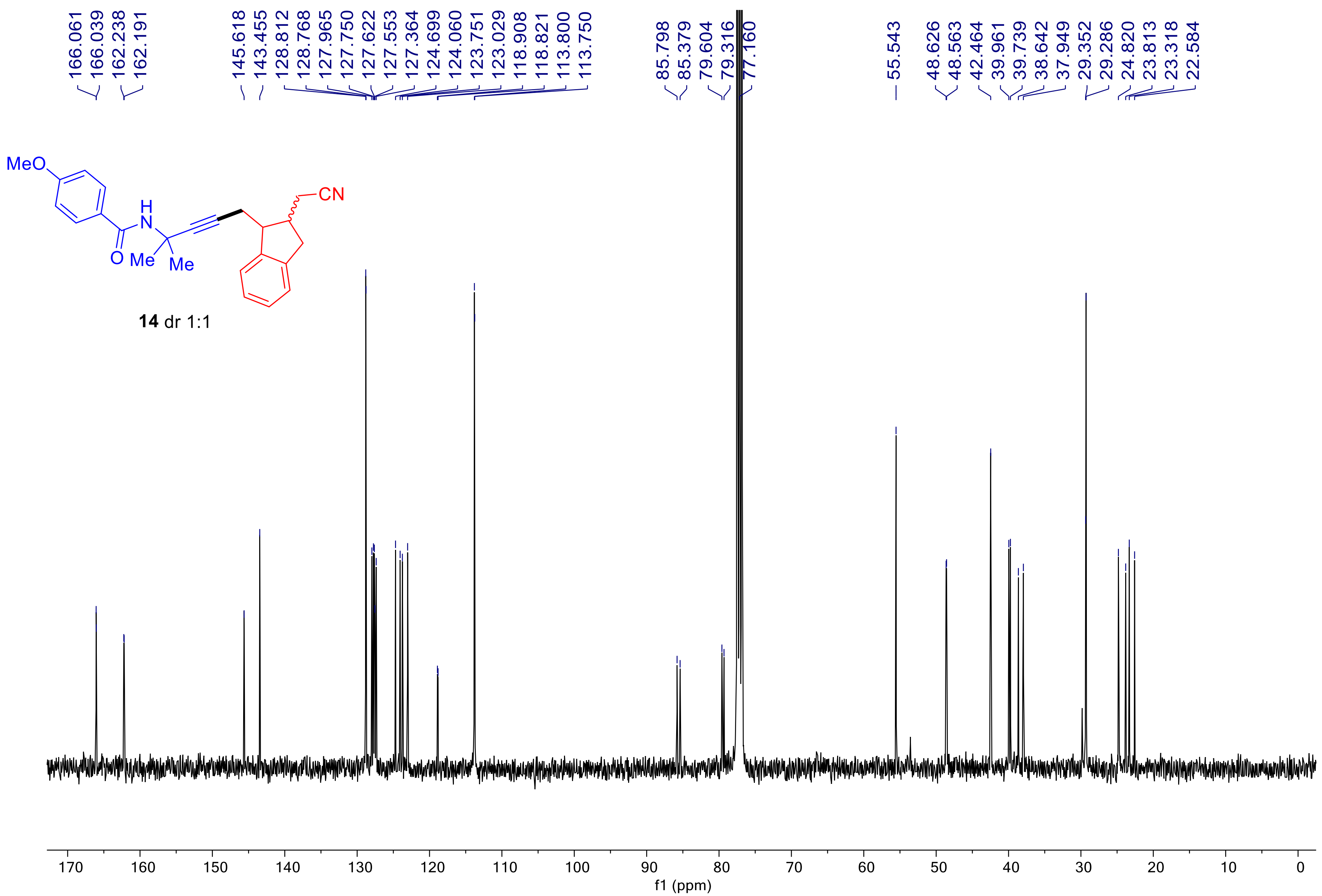

Supplementary Figure 222. ^{13}**C NMR spectrum of 14**

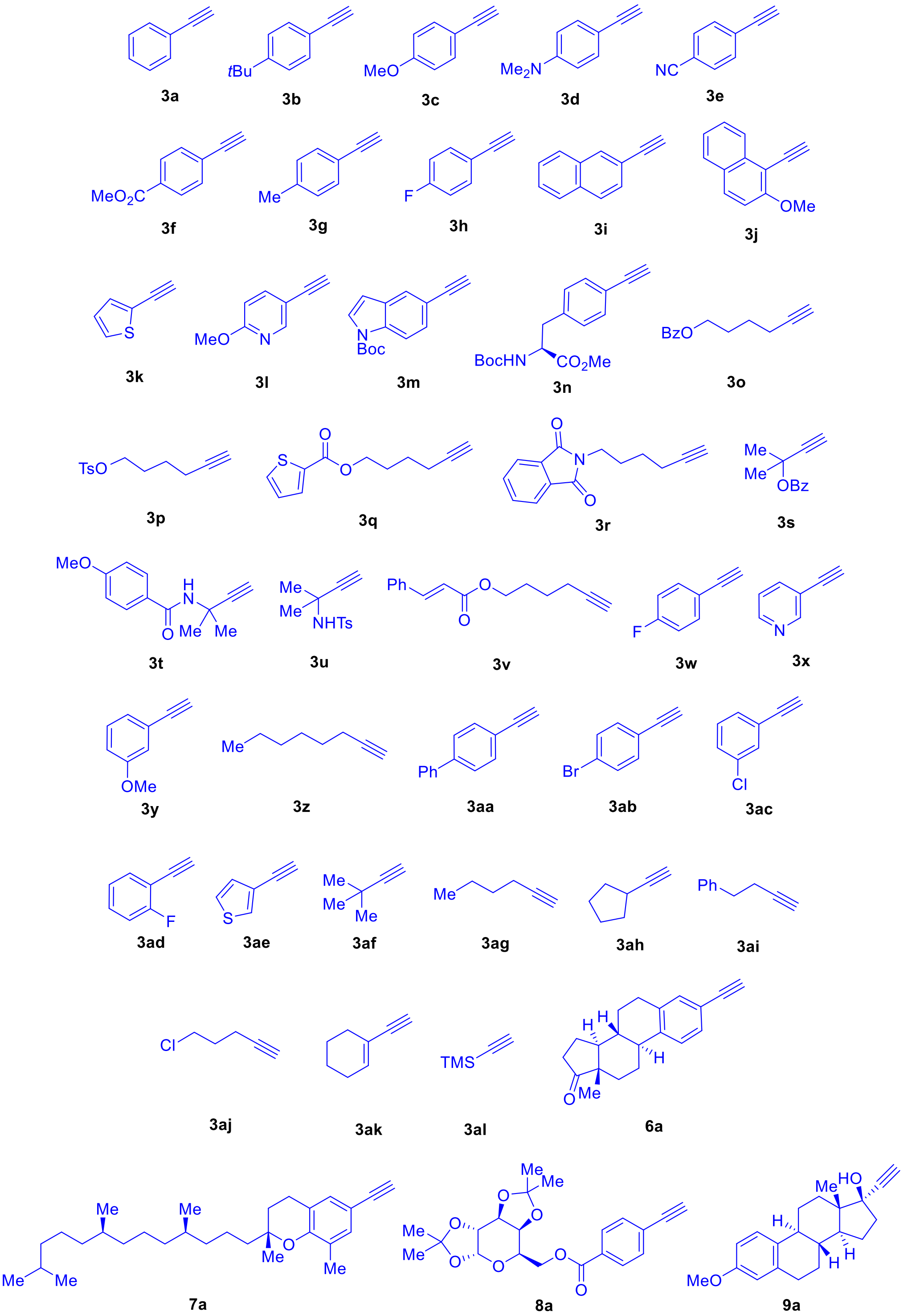

Supplementary Figure 223. List of alkynes used in the reactions

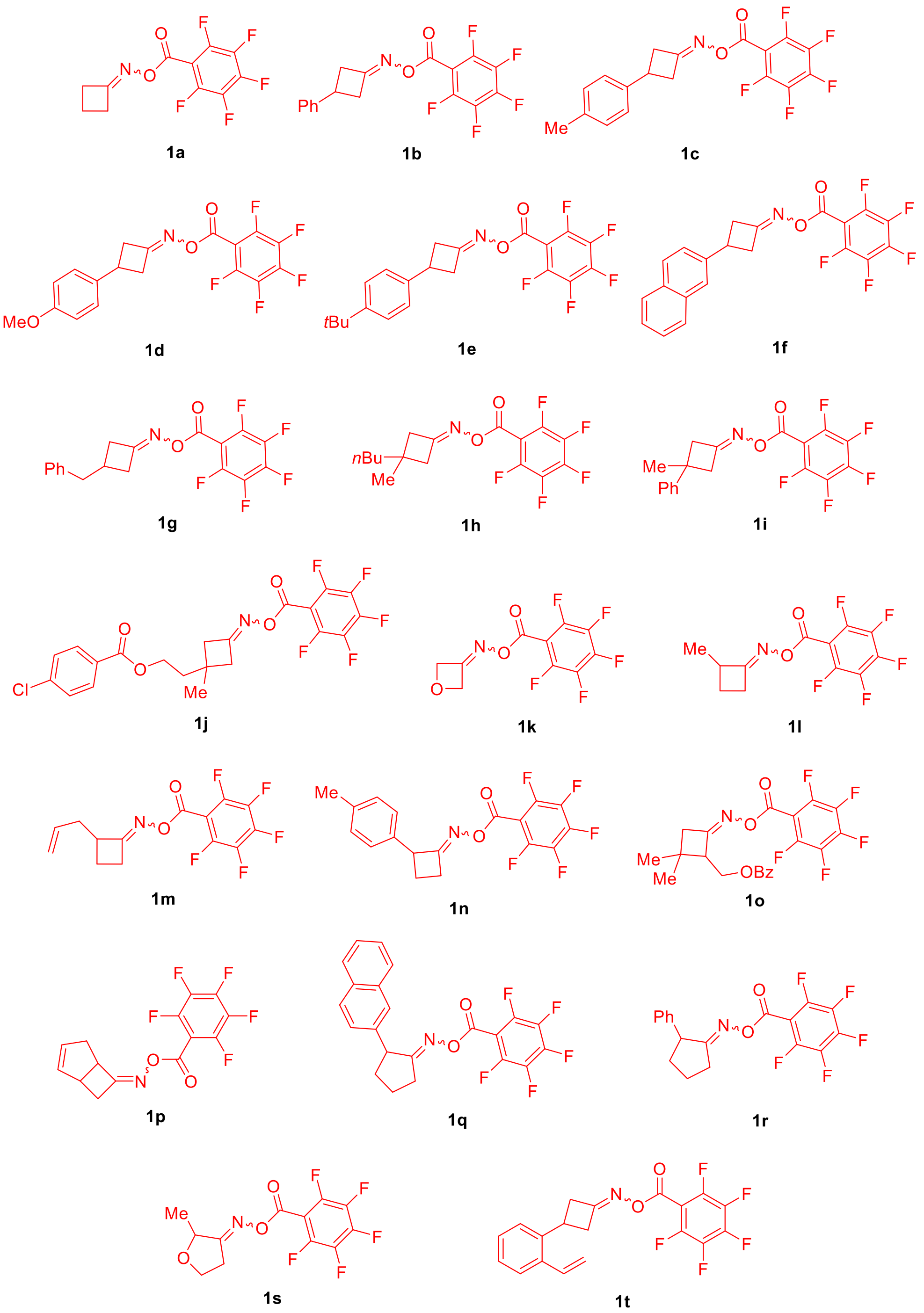

Supplementary Figure 224. List of Oxime Esters used in the Copper-Catalyzed Fragmentation/Alkynylation Protocol

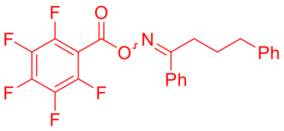

2a

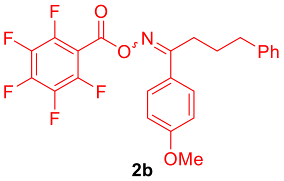

2b

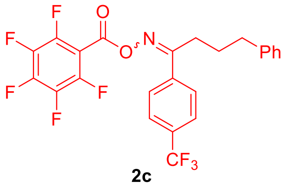

2c

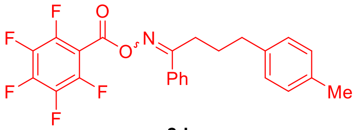

2d

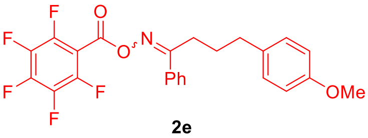

2e

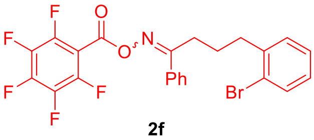

2f

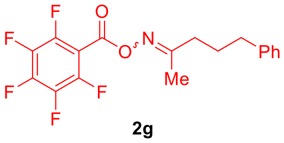

2g

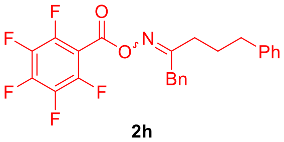

2h

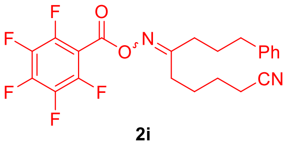

2i

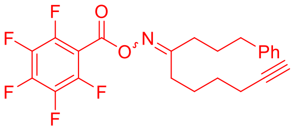

2j

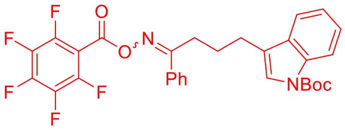

2k

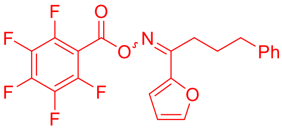

2l

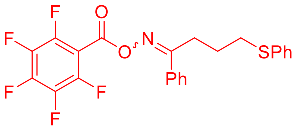

2m

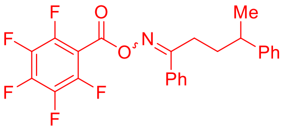

2n

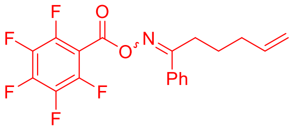

2o

Supplementary Figure 225. List of Oxime Esters used in the Copper-Catalyzed 1,5-HAT/Alkynylation Protocol

Supplementary Tables

Supplementary Table 1. Copper-catalyzed Radical Relay Fragmentation/ Alkynylation of Oxime Esters: **Ligand Effect Screening**

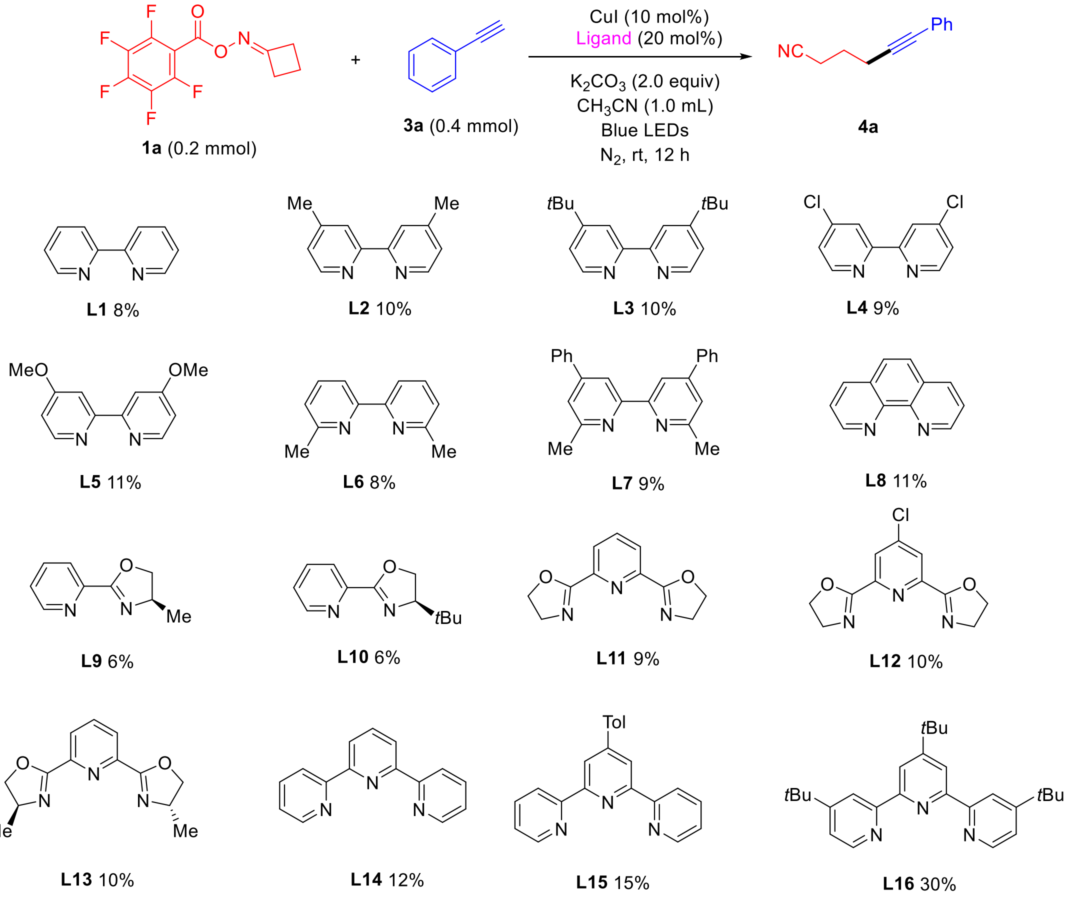

Yields were determined by ^{1}H NMR spectroscopy with 1,3,5-trimethoxybenzene as internal standard.

***L16** was chosen for the further optimization under photocatalytic conditions.

Supplementary Table 2. Copper-catalyzed Radical Relay Fragmentation/ Alkynylation of Oxime Esters: **Solvent Effect Screening**

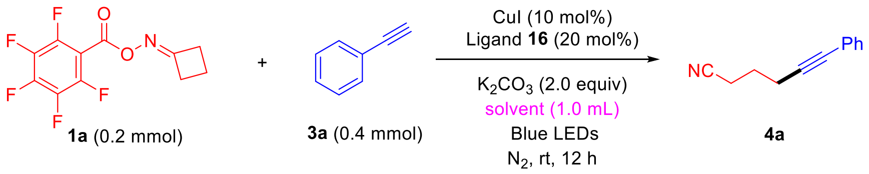

entry	Solvent	^{1}H NMR yield[a]
1	PhH	12%
2	PhMe	13%
3	$PhCF_3$	44%
4	DCM	0%
5	CH_3CN	30%
6	CH_3COCH_3	32%
7	DMF	34%
8	MeOH	0%
9	1,4-dioxane	23%
10	THF	22%

a: with 1,3,5-trimethoxybenzene as internal standard

Supplementary Table 3. Copper-catalyzed Radical Relay Fragmentation/ Alkynylation of Oxime Esters: **Base Effect Screening**

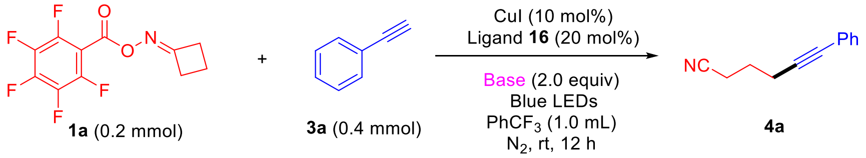

entry	Base	^{1}H NMR yield[a]
1	$NaHCO_3$	0%
2	$KHCO_3$	0%
3	Li_2CO_3	0%
4	Na_2CO_3	0%
5	K_2CO_3	44%
6	Cs_2CO_3	24%
7	*t*BuOLi	8%
8	*t*BuONa	10%
9	*t*BuOK	10%
10	CsF	0%
11	NaOAc	0%
12	KOAc	0%
13	Et_3N	0%
14	DMAP	0%

a: with 1,3,5-trimethoxybenzene as internal standard

Supplementary Table 4. Copper-catalyzed Radical Relay Fragmentation/ Alkynylation of Oxime Esters: **Catalyst Effect Screening**

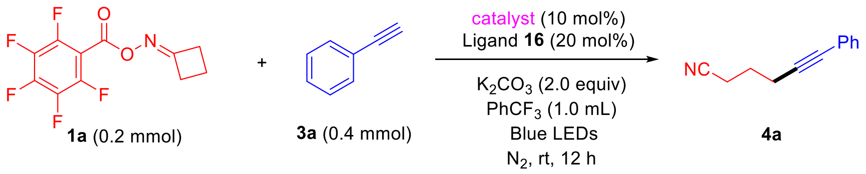

entry	Catalyst	^{1}H NMR yield[a]
1	$Cu(OTf)_2$	27%
2	$CuPF_6(CH_3CN)_2$	20%
3	CuI	44%
4	$Cu(OAc)_2$	0%
5	Copper(II) trifluoroacetylacetonate	0%
6	CuF_2	0%
7	$CuCl_2$	15%
8	$CuSO_4$	0%
9	$Cu_2(OH)_2CO_3$	0%
10	Copper (II) 2-ethylhexanoate	0%

a: with 1,3,5-trimethoxybenzene as internal standard

Note: The reaction was further optimized by increasing the temperature without blue LEDs.

Supplementary Table 5. Copper-catalyzed Radical Relay Fragmentation/ Alkynylation of Oxime Esters: **Solvent and Temperature Effect**

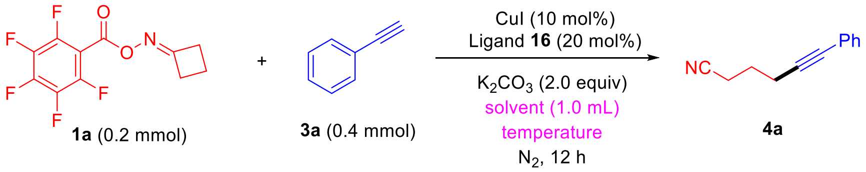

entry	Solvent	Temperature	1H NMR yield[a]
1	CH_3CN	rt	20%
2	CH_3CN	60 °C	76%
3	CH_3CN	70 °C	75%
4	$PhCF_3$	rt	15%
5	$PhCF_3$	60 °C	30%

a: with 1,3,5-trimethoxybenzene as internal standard

Supplementary Table 6. Copper-catalyzed Radical Relay Fragmentation/ Alkynylation of Oxime Esters: **Catalyst/Ligand Effect Screening**

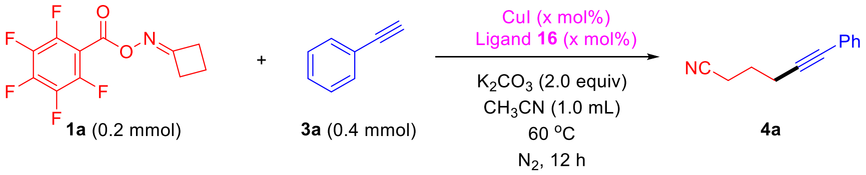

entry	CuI (x mol%)	Ligand **16** (x mol%)	1H NMR yield[a]
1	10 mol%	20 mol%	76%
2	10 mol%	10 mol%	72%
3	20 mol%	20 mol%	50%

a: with 1,3,5-trimethoxybenzene as internal standard

Supplementary Table 7. Copper-catalyzed Radical Relay Fragmentation/ Alkynylation of Oxime Esters: **Alkyne Effect Screening**

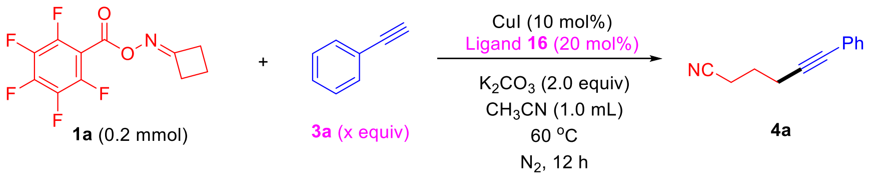

entry	**3a** (x equiv)	^{1}H NMR yield[a]
1	3 equiv	73%
**2**	**2 equiv**	**76%**

a: with 1,3,5-trimethoxybenzene as internal standard

Supplementary Table 8. Copper-catalyzed 1,5-HAT/Alkynylation of Oxime Esters: **Catalyst Effect Screening**

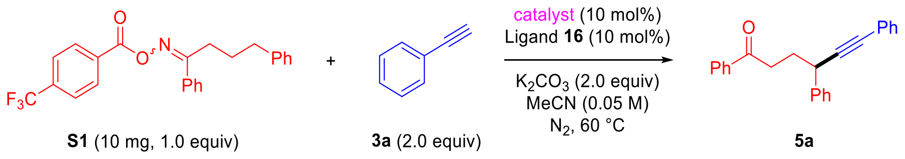

entry	Catalyst	^{1}H NMR yield[a]
1	CuI	traces
2	$Cu(OTf)_2$	16%[b]
3	$CuPF_6(MeCN)_4$	24%[b]
4	$CuCl_2$	traces
5	$Cu(OAc)_2$	<15%
6	$Cu(acac)_2$	traces
7	Cu(II) trifluoroacetylacetonate	<15%
8	CuF_2	0%
9	$CuSO_4$	0%
10	Cu(II) 2-ethylhexanoate	0%
11	$CuBr_2$	<15%
12	CuCl	<15%
13	$Cu(BF_4)_2 \cdot 6H_2O$	17%[b]
14	NHC-Cu	<15%
15	$(CuOTf)_2 \cdot C_6H_6$ complex	24%[b]
16	CuBr	<10%
17	CuOAc	<10%

a: with 1,3,5-trimethoxybenzene as internal standard

b: isolated yield

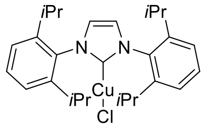

NHC-Cu

Supplementary Table 9. Copper-catalyzed 1,5-HAT/Alkynylation of Oxime Esters: **Ligand Effect Screening**

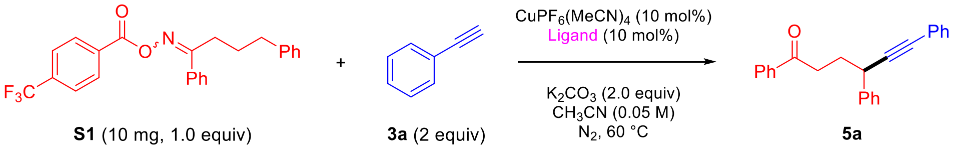

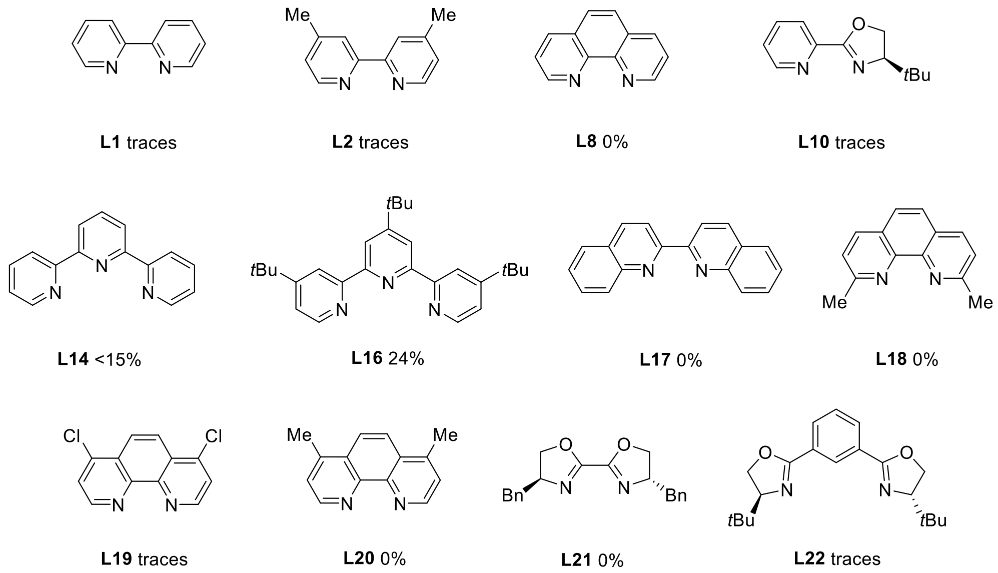

Yields were determined by ^{1}H NMR spectroscopy with 1,3,5-trimethoxybenzene as internal standard.

***L16** was chosen for the further optimization

Supplementary Table 10. Copper-catalyzed 1,5-HAT/Alkynylation of Oxime Esters: **Solvent Effect Screening**

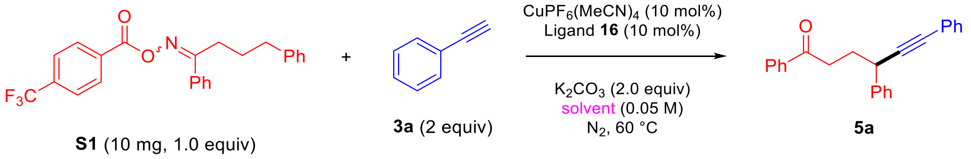

entry	Solvent	^{1}H NMR yield[a]
1	CH_3CN	24%[b]
2	DMF	0%
3	*t*BuOH	traces
4	$PhCH_3$	<15%
5	$PhCF_3$	0%
6	DCE	20%
7	THF	0%
8	PhH	<15%
9	PhCl	<15%
10	CH_3COCH_3	0%

a: with 1,3,5-trimethoxybenzene as internal standard

b: isolated yield

Supplementary Table 11. Copper-catalyzed 1,5-HAT/Alkynylation of Oxime Esters: **Base Effect Screening**

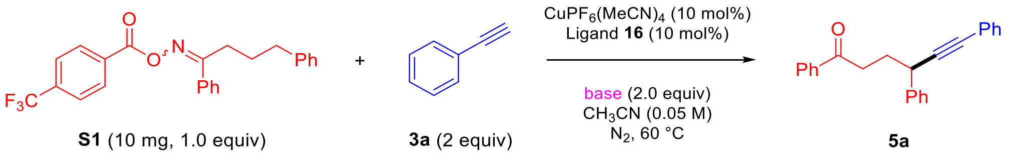

entry	Base	^{1}H NMR yield[a]
1	K_2CO_3	24%[b]
2	Li_2CO_3	0%
3	Na_2CO_3	0%
4	Cs_2CO_3	<15%
5	*t*BuONa	0%
6	*t*BuOK	0%

a: with 1,3,5-trimethoxybenzene as internal standard

b: isolated yield

Supplementary Table 12. Copper-catalyzed 1,5-HAT/Alkynylation of Oxime Esters: **Leaving Group Effect Screening**

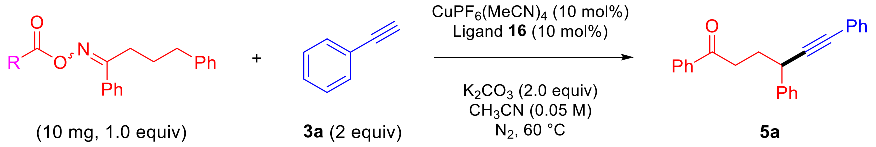

entry	R	^{1}H NMR yield[a]
1	4-$F_3CC_6H_4$ (**S1**)	24%[b]
2	*t*Bu (**S2**)	<10%
3	C_6F_5 (**2a**)	47%[b]
4	C_6F_5 (**2a**)	25%[c]
5	C_6F_5 (**2a**)	59%[b,d]
6	3,5-$(F_3C)_2C_6H_3$ (**S3**)	<10%
6	4-NCC_6H_4 (**S4**)	<10%

a: with 1,3,5-trimethoxybenzene as internal standard

b: isolated yield

c: 0.1 M

d: $(CuOTf)_2 \cdot C_6H_6$ complex as catalyst

Supplementary Table 13. Copper-catalyzed 1,5-HAT/Alkynylation of Oxime Esters: **Catalyst/Ligand and Temperature Effect Screening**

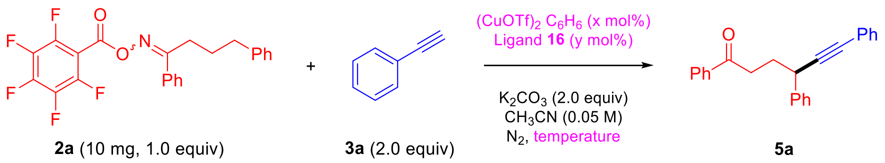

entry	Temperature	x mol% / y mol%	^{1}H NMR yield[a]
1	60 °C	10 mol% / 10 mol%	59%
2	45 °C	10 mol% / 10 mol%	35%
3	45 °C	5 mol% / 10 mol%	59%[b]
4	60 °C	5 mol% / 10 mol%	47%
5	rt	5 mol% / 10 mol%	19%
6	45 °C	2.5 mol% / 5 mol%	62%[b]

a: with 1,3,5-trimethoxybenzene as internal standard

b: isolated yield

Supplementary Table 14. Copper-catalyzed 1,5-HAT/Alkynylation of Oxime Esters: **Additional Tuning 1**

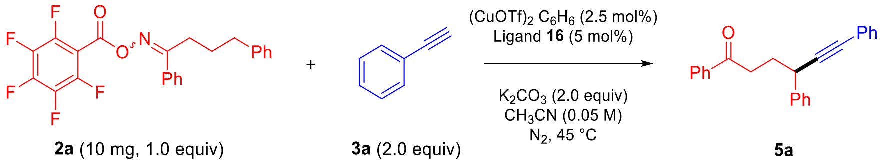

entry	Variation	^{1}H NMR yield[a]
1	None	62%[b]
2	3 equiv base / 3 equiv alkyne	60%
3	Na_2CO_3 as base	35%
4	Organic bases such as DIPEA, Et$_3$N, pyrrolidine, DBU	<15%
5	DCM	36%
6	DCE	58%
7	5 mol% catalyst<br>10 mol% ligand<br>DCE as solvent	71% (68%[b])

a: with 1,3,5-trimethoxybenzene as internal standard

b: isolated yield

Supplementary Table 15. Copper-catalyzed 1,5-HAT/Alkynylation of Oxime Esters: **Additional Tuning 2**

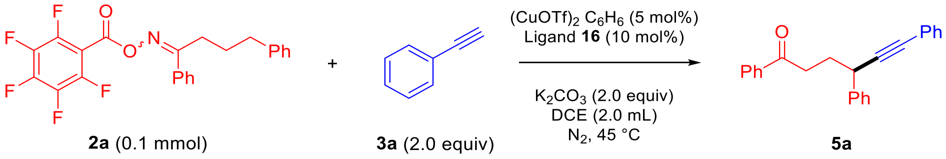

entry	Variation	^{1}H NMR yield[a]
1	None	71% (68%[b])
2	DCE:CH$_3$CN (2:1) as solvent	70%
**3**	**dry DCE**	**76%[b]**

a: with 1,3,5-trimethoxybenzene as internal standard

b: isolated yield

Supplementary Methods

General Information

NMR spectra were recorded on AV2 400 Bruker spectrometers. Chemical shifts are given in ppm. The spectra are calibrated to the residual ^{1}H and ^{13}C signals of the solvents. Multiplicities are abbreviated as follows: singlet (s), doublet (d), triplet (t), quartet (q), doublet-doublet (dd), quintet (quint), multiplet (m) and broad (*br*). Infrared spectra were recorded on a JASCO FT/IR-4100 spectrometer. High-resolution electrospray ionization and electronic impact mass spectrometry was performed on a Finnigan MAT 900 (Thermo Finnigan, San Jose, CA; USA) double focusing magnetic sector mass spectrometer.

Materials and Methods

Unless otherwise stated, starting materials were purchased from Aldrich and/or Fluka. Solvents were used directly without further purification. Chromatography (TLC) using Merck TLC silica gel 60 F254. Compounds were visualized by UV light at 254 nm and by dipping the plates in an ethanolic vanillin/sulfuric acid solution or an aqueous potassium permanganate solution followed by heating. Flash column chromatography was performed over silica gel (230-400 mesh).

General Procedure A for the Copper-catalyzed Radical Relay Fragmentation/Alkynylation of Oxime Esters

Alkynes (0.4 mmol, 2.0 equiv), K_2CO_3 (0.4 mmol, 2.0 equiv) and CuI (0.02 mmol, 10 mol%), ligand (0.04 mmol, 20 mol%), *N*-O oxime esters (0.2 mmol, 1.0 equiv) were placed in a dry Schlenk-tube. The reaction vessel was evacuated and filled up with nitrogen three times, then CH_3CN (1 mL) was added at rt. The reaction mixture was stirred at 60 °C for 12 h. The resulting mixture was extracted with DCM (3 x 15 mL) and the combined organic layers were dried over anhydrous $MgSO_4$. After removal of the solvent under reduced pressure, the crude product was purified by column chromatography on silica gel eluting with ether/petroleum ether to give the corresponding products.

General Procedure B for the Copper-catalyzed 1,5-HAT/Alkynylation of Oxime Esters

A suspension of the corresponding oxime ester (0.1 mmol, 1 equiv), K_2CO_3 (0.2 mmol, 2 equiv), ligand (10 mol%, 0.1 equiv) and $(CuOTf)_2 \cdot C_6H_6$ (5 mol%, 0.05 equiv) in DCE (0.05 M) was deoxygenated by freeze-pump-thaw cycles. Alkyne was added consecutively by using a syringe (if liquid, otherwise it was weighed alongside the other reactants). The reaction mixture was stirred at 45 °C until the complete consumption of

the starting material (monitored by TLC). The reaction mixture was poured into a saturated $NaHCO_3$ solution and extracted with EtOAc (3 x 15 mL). The combined organic layers were dried over Na_2SO_4 and evaporated to dryness under reduced pressure. The residue was purified by flash column chromatography on silica gel to afford the corresponding alkynylated ketone.

Note: All the *N*-O oxime esters were synthesized according to the reported procedures.[1]

Characterization Data of the New Substrates
Oxime Esters

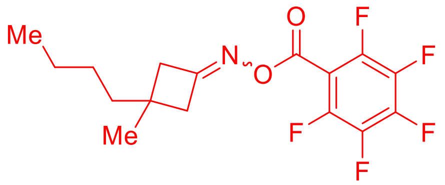

It was obtained as a mixture of 2 isomers ≈ 5:1.

3-Butyl-3-methylcyclobutan-1-one *O*-perfluorobenzoyl oxime (1h). ^{1}H NMR (400 MHz, $CDCl_3$) δ 2.85 - 2.65 (m, 4H), 1.54 - 1.50 (m, 2H), 1.39 - 1.24 (m, 4H), 1.23 (s, 3H), 0.91 (t, *J* = 6.8 Hz, 3H); ^{13}C NMR (100 MHz, $CDCl_3$) δ *(Peaks correspond only to the major isomer)* 168.7, 156.8, 145.5 (m), 143.6 (m), 137.8 (m), 107.2 (m), 43.8, 43.3, 41.0, 33.7, 27.1, 25.5, 23.1, 14.1; IR (film): ν (cm^{-1}) 2930, 1720, 1490, 1320, 1215, 1085, 864, 761; ^{19}F NMR (376 MHz, $CDCl_3$) δ -137.2 (m), -148.1 (m), -160.1 (m); HR-MS (ESI) *m/z* calcd for $C_{16}H_{17}F_5NO_2$ [M+H$^+$]: 350.1174; found 350.1177.

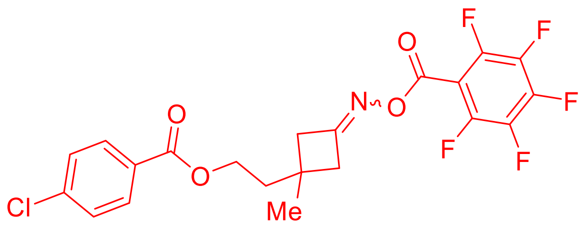

2-(1-Methyl-3-(((perfluorobenzoyl)oxy)imino)cyclobutyl)ethyl 4-chlorobenzoate (1j). ^{1}H NMR (400 MHz, $CDCl_3$) δ 7.95 - 7.93 (m, 2H), 7.43 - 7.40 (m, 2H), 4.42 (t, *J* = 6.8 Hz, 2H), 3.05 - 3.00 (m, 1H), 2.99 - 2.94 (m, 1H), 2.89 - 2.80 (m, 2H), 2.09 - 2.05 (m, 2H), 1.36 (s, 3H); ^{13}C NMR (100 MHz, $CDCl_3$) δ 167.7, 165.7, 156.6, 145.5 (m), 144.8 (m), 143.5 (m), 142.3 (m), 139.7, 139.1 (m), 137.8 (m), 131.0, 128.9, 128.5, 106.9 (m), 62.2, 44.3, 43.9, 39.2, 32.3, 25.4; ^{19}F NMR (376 MHz, $CDCl_3$) δ -137.0 (m), -147.6 (m), -159.8 (m); IR (film): ν (cm^{-1}) 2931, 1660, 1518, 1479, 1375, 1224, 1150, 1046, 876, 745; HR-MS (ESI) *m/z* calcd for $C_{21}H_{16}ClF_5NO_4$ [M+H$^+$]: 476.0683; found 476.0692.

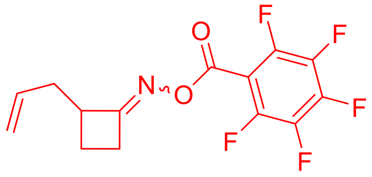

2-Allylcyclobutan-1-one *O*-perfluorobenzoyl oxime (1m). ^{1}H NMR (400 MHz, $CDCl_3$) δ 5.88 - 5.78 (m, 1H), 5.15 - 5.09 (m, 2H), 3.53 - 3.45 (m, 1H), 3.06 - 2.90 (m, 2H), 2.62 - 2.55 (m, 1H), 2.46 - 2.39 (m, 1H), 2.27 - 2.17 (m, 1H), 1.87 - 1.78 (m, 1H); ^{13}C NMR (100 MHz, $CDCl_3$) δ 173.8, 156.8, 145.5 (m), 143.5 (m), 137.9 (m), 134.4, 117.4, 107.2 (m), 44.7, 36.1, 29.5, 20.2; ^{19}F NMR (376 MHz, $CDCl_3$) δ -137.2 (m), -148.1 (m), -160.0 (m); IR (film): ν (cm^{-1}) 1758, 1501, 1327, 1210, 1096, 998, 847, 702; HR-MS (ESI) *m/z* calcd for $C_{14}H_{11}F_5NO_2$ [$M+H^+$]: 320.0704; found 320.0709.

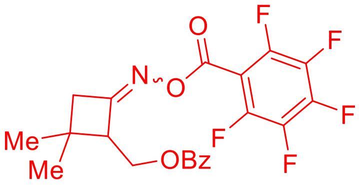

(2,2-Dimethyl-4-(((perfluorobenzoyl)oxy)imino)cyclobutyl)methyl benzoate (1o). ^{1}H NMR (400 MHz, $CDCl_3$) δ 8.04 - 8.02 (m, 2H), 7.60 - 7.56 (m, 1H), 7.48 - 7.44 (m, 2H), 4.69 (dd, J = 11.6, 5,6 Hz, 1H), 4.54 (dd, J = 11.6, 8.8 Hz, 1H), 3.53 - 3.49 (m, 1H), 2.82 (dd, J = 17.2, 2.0 Hz, 1H), 2.76 (dd, J = 17.2, 2.0 Hz, 1H), 1.39 (s, 3H), 1.28 (s, 3H); ^{13}C NMR (100 MHz, $CDCl_3$) δ 168.0, 166.2, 156.8, 145.5 (m), 143.5 (m), 137.9 (m), 133.4, 129.9, 129.8, 128.6, 106.9 (m), 61.3, 52.9, 44.2, 33.7, 29.9, 22.8; ^{19}F NMR (376 MHz, $CDCl_3$) δ -137.3 (m), -147.9 (m), -159.9 (m); IR (film): ν (cm^{-1}) 2961, 1760, 1725, 1501, 1325, 1270, 1183, 1001, 854, 732; HR-MS (ESI) *m/z* calcd for $C_{39}H_{62}NO_2$ [$M+H^+$]: 576.4775; found 576.4783.

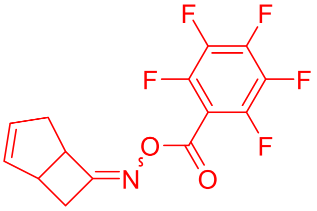

It was obtained as a mixture of isomers ≈ 5:1.

Bicyclo[3.2.0]hept-2-en-6-one *O*-perfluorobenzoyl oxime (1p). ^{1}H NMR (400 MHz, $CDCl_3$) δ 5.87 - 5.80 (m, 2H), 3.96 - 3.82 (m, 1H), 3.47 - 3.41 (m, 1H), 3.36 - 3.27 (m, 0.4 H), 3.24 - 3.17 (m, 0.6 H), 2.85 - 2.79 (m, 1H), 2.78 - 2.45 (m, 2H); ^{13}C NMR (100 MHz, $CDCl_3$) δ *(Peaks correspond only to the major isomer)* 175.1, 156.8, 145.5 (m), 143.5 (m), 137.9 (m), 132.7, 132.1, 107.1 (m), 46.9, 40.8, 39.4, 37.7; IR (film): ν (cm-

^{1}) 2929, 1760, 1501, 1325, 1205, 1011, 931, 854, 728; ^{19}F NMR (376 MHz,$CDCl_3$) δ -137.1 (m), -147.9 (m), -160.0 (m); HR-MS (ESI) *m/z* calcd for $C_{14}H_9F_5NO_2$ [M+H$^+$]: 318.0548; found 318.0555.

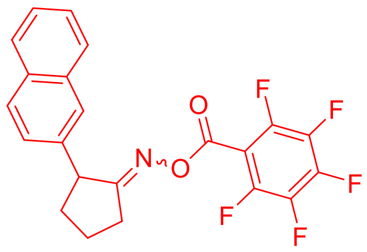

2-(Naphthalen-2-yl)cyclopentan-1-one *O*-perfluorobenzoyl oxime (1q). ^{1}H NMR (400 MHz, $CDCl_3$) δ 7.85 - 7.80 (m, 3H), 7.72 (s, 1H), 7.50 - 7.44 (m, 2H), 7.41 (dd, *J* = 8.8, 2.0 Hz, 1H), 4.20 - 4.16 (m, 1H), 2.97 - 2.89 (m, 1H), 2.83 - 2.74 (m, 1H), 2.44 - 2.36 (m, 1H), 2.21 - 2.12 (m, 1H), 2.10 - 2.02 (m, 1H), 1.95 - 1.86 (m, 1H); ^{13}C NMR (100 MHz, $CDCl_3$) δ 179.1, 156.8, 145.5 (m), 143.5 (m), 137.9 (m), 137.1, 133.5, 132.6, 128.6, 127.9, 127.7, 126.6, 126.3, 126.1, 126.0, 107.2 (m), 49.7, 34.7, 30.6, 22.7; ^{19}F NMR (376MHz, $CDCl_3$) δ -137.2 (m), -148.1 (m), -160.0 (m); IR (film): ν (cm^{-1}) 2160, 1763, 1501, 1328, 1212, 1006, 862, 712; HR-MS (ESI) *m/z* calcd for $C_{22}H_{14}F_5NNaO_2$ [M+Na$^+$]: 442.0837; found 442.0835.

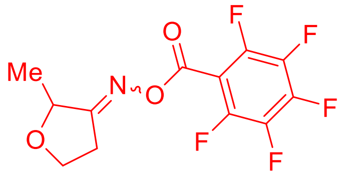

2-Methyldihydrofuran-3(2*H*)-one *O*-perfluorobenzoyl oxime (1s). ^{1}H NMR (400 MHz, $CDCl_3$) δ 4.54 (q, *J* = 6.4 Hz, 1H), 4.21 - 4.16 (m, 1H), 3.93 - 3.87 (m, 1H), 2.98 - 2.83 (m, 2H), 1.51 (d, *J* = 6.4 Hz, 3H); ^{13}C NMR (100 MHz, $CDCl_3$) δ 175.2, 156.4, 145.6 (m), 143.7 (m), 137.9 (m), 106.8 (m), 75.1, 65.6, 30.6, 18.0; ^{19}F NMR (376MHz, $CDCl_3$) δ -136.8 (m), -146.9 (m), -159.6 (m); IR (film): ν (cm^{-1}) 2931, 1660, 1518, 1479, 1375, 1224, 1150, 1046, 876, 745; HR-MS (ESI) *m/z* calcd for $C_{39}H_{62}NO_2$ [M+H$^+$]: 576.4775; found 576.4783.

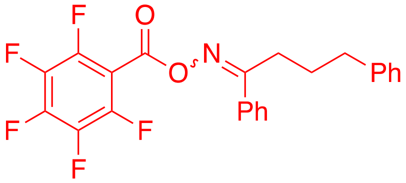

This compound was prepared using 1,4-diphenylbutan-1-one (2.92 mmol) as starting material and purified by column chromatography on silica gel (petroleum ether/EtOAc 97:3).

1,4-Diphenylbutan-1-one *O*-perfluorobenzoyl oxime (2a). White solid, 97% (1.23 g). Mp: 88 - 90 °C; ^{1}H NMR (400 MHz, $CDCl_3$) δ 7.70 - 7.68 (m, 2H), 7.51 - 7.46 (m, 1H), 7.44 - 7.40 (m, 2H), 7.25 - 7.22 (m, 2H), 7.18 - 7.12 (m, 3H), 2.93 - 2.89 (m, 2H), 2.69 (t, J = 7.6 Hz, 2H), 1.96 - 1.88 (m, 2H); ^{13}C NMR (100 MHz, $CDCl_3$) δ *(Peaks correspond only to the major isomer)* 168.8, 141.0, 133.1, 131.2, 129.0 (2), 128.5 (2), 128.5 (2), 127.5 (2), 126.2, 35.7, 28.4, 28.3 (*peaks corresponding to the perfluorinated benzoyl ester moiety were not resolvable due to their anticipated weak intensity*); ^{19}F NMR (376 MHz, $CDCl_3$) δ -137.1 (m), -147.7 (m), -159.7 (m); IR (film): ν (cm^{-1}) 2932, 1766, 1652, 1496, 1452, 1417, 1323, 1184, 1084, 1003; HR-MS (ESI) *m/z* calcd for $C_{23}H_{16}F_5NO_2Na$ [M+Na$^+$]: 456.0999; found 456.0994.

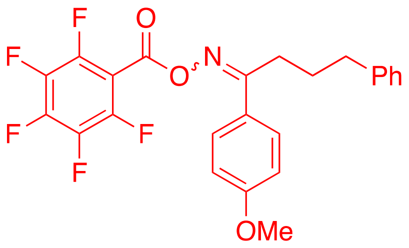

This compound was prepared using 1-(4-methoxyphenyl)-4-phenylbutan-1-one (7.29 mmol) as starting material, purified by column chromatography on silica gel (petroleum ether/EtOAc 96:4) and obtained as a mixture of isomers ≈ 9:1.

1-(4-Methoxyphenyl)-4-phenylbutan-1-one *O*-perfluorobenzoyl oxime (2b). White solid, 84%, 2.82 g. Mp: 96 - 98 °C; ^{1}H NMR (400 MHz, $CDCl_3$) δ 7.68 - 7.64 (m, 1.8H), 7.30 - 7.22 (m, 2.2H), 7.20 - 7.12 (m, 3H), 6.94 - 6.90 (m, 2H), 3.85 (s, 2.7H), 3.84 (s, 0.3H), 2.89 - 2.85 (m, 1.8H), 2.79 - 2.75 (m, 0.2H), 2.71 - 2.65 (m, 2H), 1.95 - 1.79 (m, 2H); ^{13}C NMR (100 MHz, $CDCl_3$) δ *(Peaks correspond only to the major isomer)* 168.0, 162.1, 141.1, 129.1 (2), 128.5 (2), 128.5 (2), 126.2, 125.2, 114.3 (2), 55.6, 35.7, 28.5, 28.0 (*peaks corresponding to the perfluorinated benzoyl ester moiety were not resolvable due to their anticipated weak intensity*); ^{19}F NMR (376 MHz, $CDCl_3$) δ -137.3 (m), -147.9 (m), -159.9 (m); IR (film): ν (cm^{-1}) 2933, 1759, 1651, 1604, 1496, 1456, 1415, 1327, 1252, 1196, 1182; HR-MS (ESI) *m/z* calcd for $C_{24}H_{18}F_5NNaO_3$ [M+Na$^+$]: 486.1099; found 486.1103.

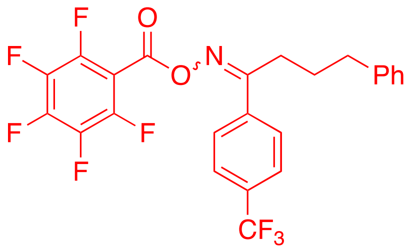

This compound was prepared using 4-phenyl-1-(4-(trifluoromethyl)phenyl)butan-1-one (1.83 mmol) as starting material, purified by column chromatography on silica gel

(petroleum ether/EtOAc 97:3).

4-Phenyl-1-(4-(trifluoromethyl)phenyl)butan-1-one *O*-perfluorobenzoyl oxime (2c). White solid, 87%, 806 mg. Mp: 83 - 85 °C; ^{1}H NMR (400 MHz, $CDCl_3$) δ 7.80 - 7.78 (m, 2H), 7.68 - 7.66 (m, 2H), 7.26 - 7.23 (m, 2H), 7.19 - 7.15 (m, 1H), 7.13 - 7.11 (m, 2H), 2.94 - 2.90 (m, 2H), 2.70 (t, J = 7.6 Hz, 2H), 1.95 - 1.88 (m, 2H); ^{13}C NMR (100 MHz, $CDCl_3$) δ 167.5, 140.7, 136.7, 133.0 (q, J = 32.5 Hz), 128.6 (2), 128.5 (2), 128.0 (2), 126.4, 125.9 (q, J = 3.7 Hz) (2H), 123.8 (q, J = 270.8 Hz), 35.6, 28.2 (2) (*peaks corresponding to the perfluorinated benzoyl ester moiety were not resolvable due to their anticipated weak intensity*); ^{19}F NMR (376 MHz, $CDCl_3$) δ -63.0 (s), -136.9 (m), -147.1 (m), -159.5 (m); IR (film): ν (cm^{-1}) 2949, 1759, 1652, 1525, 1500, 1320, 1193, 1173, 1134, 1071, 1058; HR-MS (ESI) *m/z* calcd for $C_{24}H_{15}F_8NNaO_2$ [M+Na$^+$]: 524.0867; found 524.0871.

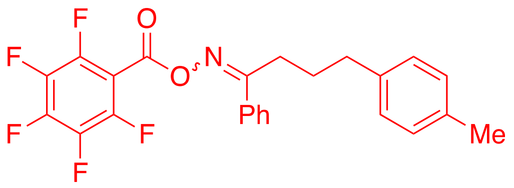

This compound was prepared using 1-phenyl-4-(*p*-tolyl)butan-1-one (0.47 mmol) as starting material, purified by column chromatography on silica gel (petroleum ether/EtOAc 98:2) and obtained as a mixture of isomers ≈ 7:1.

1-Phenyl-4-(*p*-tolyl)butan-1-one *O*-perfluorobenzoyl oxime (2d). White solid, 84%, 177 mg. Mp: 102 - 104 °C; ^{1}H NMR (400 MHz, $CDCl_3$) δ 7.71 - 7.68 (m, 1.75H), 7.50 - 7.40 (m, 3H), 7.29 - 7.27 (m, 0.25H), 7.08 - 7.00 (m, 4H), 2.92 - 2.88 (m, 1.75H), 2.79 - 2.75 (m, 0.25H), 2.65 (t, J = 7.6 Hz, 2H), 2.31 (s, 0.4H), 2.29 (s, 2.6H), 1.94 - 1.79 (m, 2H); ^{13}C NMR (100 MHz, $CDCl_3$) δ *(Peaks correspond only to the major isomer)* 168.9, 137.9, 135.7, 133.2, 131.2, 129.1 (2), 128.9 (2), 128.4 (2), 127.6 (2), 35.3, 28.4, 28.4, 21.1 (*peaks corresponding to the perfluorinated benzoyl ester moiety were not resolvable due to their anticipated weak intensity*); ^{19}F NMR (376 MHz, $CDCl_3$) δ -137.1 (m), -147.7 (m), -159.8 (m); IR (film): ν (cm^{-1}) 2931, 2023, 1763, 1653, 1500, 1417, 1323, 1192, 1093, 1000; HR-MS (ESI) *m/z* calcd for $C_{24}H_{18}F_5NNaO_2$ [M+Na$^+$]: 470.1150; found 470.1156.

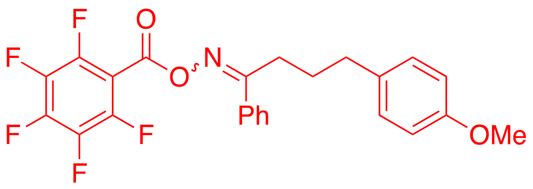

This compound was prepared using 4-(4-methoxyphenyl)-1-phenylbutan-1-one (0.34 mmol) as starting material, purified by column chromatography on silica gel (petroleum

ether/EtOAc 96:4) and obtained as a mixture of isomers ≈ 5:1.

4-(4-Methoxyphenyl)-1-phenylbutan-1-one *O*-perfluorobenzoyl oxime (2e). White solid, quantitative, 157 mg. Mp: 68 - 70 °C; ^{1}H NMR (400 MHz, $CDCl_3$) δ 7.71 - 7.68 (m, 1.66H), 7.52 - 7.40 (m, 3H), 7.29 - 7.26 (m, 0.33H), 7.05 - 7.00 (m, 2H), 6.82 - 6.74 (m, 2H), 3.78 (s, 0.5H), 3.76 (s, 2.5H), 2.91 - 2.87 (m, 1.66H), 2.78 - 2.74 (m, 0.33H), 2.63 (t, J = 7.2 Hz, 2H), 1.92 - 1.77 (m, 2H); ^{13}C NMR (100 MHz, $CDCl_3$) δ *(Peaks correspond only to the major isomer)* 168.9, 158.1, 133.2, 133.0, 131.2, 129.4 (2), 129.0 (2), 127.6 (2), 113.8 (2), 55.3, 34.7, 28.5, 28.2 (*peaks corresponding to the perfluorinated benzoyl ester moiety were not resolvable due to their anticipated weak intensity*); ^{19}F NMR (376 MHz, $CDCl_3$) δ -137.2 (m), -147.7 (m), -159.9 (m); IR (film): ν (cm^{-1}) 2941, 1763, 1653, 1611, 1499, 1442, 1418, 1322, 1246, 1192, 1038; HR-MS (ESI) *m/z* calcd for $C_{24}H_{18}F_5NNaO_3$ [M+Na$^+$]: 486.1099; found 486.1096.

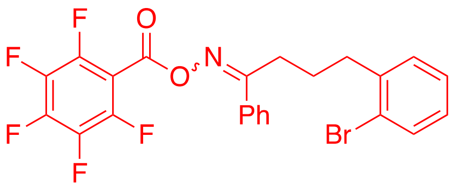

This compound was prepared using 4-(2-bromophenyl)-1-phenylbutan-1-one (0.52 mmol) as starting material, purified by column chromatography on silica gel (petroleum ether/EtOAc 98:2).

4-(2-Bromophenyl)-1-phenylbutan-1-one *O*-perfluorobenzoyl oxime (2f). White solid, 94%, 251 mg. Mp: 80 - 82°C; ^{1}H NMR (400 MHz, $CDCl_3$) δ 7.75 - 7.72 (m, 2H), 7.52 - 7.41 (m, 4H), 7.21 - 7.14 (m, 2H), 7.04 (ddd, J = 7.6, 6.8, 2.0 Hz, 1H), 2.98 - 2.94 (m, 2H), 2.83 - 2.80 (m, 2H); ^{13}C NMR (100 MHz, $CDCl_3$) δ 168.5, 140.3, 133.2, 133.0, 131.3, 130.5, 129.0 (2), 128.0, 127.6 (3), 124.5, 36.1, 28.5, 27.0; (*peaks corresponding to the perfluorinated benzoyl ester moiety were not resolvable due to their anticipated weak intensity*); ^{19}F NMR (376 MHz, $CDCl_3$) δ -136.9 (m), -147.6 (m), -159.7 (m); IR (film): ν (cm^{-1}) 2964, 1751, 1649, 1521, 1496, 1469, 1413, 1324, 1198, 1003; HR-MS (ESI) *m/z* calcd for $C_{23}H_{15}{}^{79}BrF_5NNaO_2$ [M+Na$^+$]: 534.0099; found 534.0099.

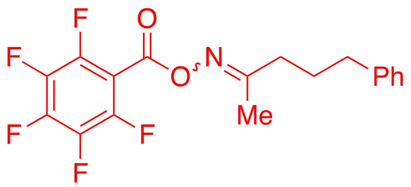

This compound was prepared using 5-phenylpentan-2-one (0.57 mmol) as starting material, purified by column chromatography on silica gel (petroleum ether/EtOAc 97:3) and obtained as a mixture of isomers ≈ 7:3.

5-Phenylpentan-2-one *O*-perfluorobenzoyl oxime (2g). Colorless oil, 93%, 197 mg. ^{1}H NMR (400 MHz, $CDCl_3$) δ 7.31 - 7.14 (m, 5H), 2.69 (t, *J* = 7.6 Hz, 1.4H), 2.66 (t, *J* = 7.6 Hz, 0.6H), 2.51 - 2.47 (m, 0.6H), 2.46 - 2.42 (m, 1.4H), 2.09 (s, 0.9H), 2.02 (s, 2.1H), 1.99 - 1.84 (m, 2H); ^{13}C NMR (100 MHz, $CDCl_3$) δ *(Major isomer)* 169.0, 141.3, 128.6 (4), 126.2, 35.4, 35.4, 27.9, 16.0; *(minor isomer)* 169.6, 140.9, 128.5 (2), 128.4 (2), 126.3, 35.7, 30.8, 27.4, 20.1; (*peaks corresponding to the perfluorinated benzoyl ester moiety were not resolvable due to their anticipated weak intensity*); ^{19}F NMR (376 MHz, $CDCl_3$) δ -137.3 (m), -148.0 (m), -160.0 (m); IR (film): ν (cm^{-1}) 2929, 1759, 1651, 1522, 1496, 1325, 1194, 1092, 997; HR-MS (ESI) *m/z* calcd for $C_{18}H_{14}F_5NNaO_2$ [M+Na^+]: 394.0837; found 394.0839.

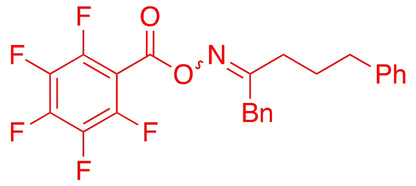

This compound was prepared using 1,5-diphenylpentan-2-one (0.35 mmol) as starting material, purified by column chromatography on silica gel (petroleum ether/EtOAc 96:4) and obtained as a mixture of isomers ≈ 5:4.

1,5-Diphenylpentan-2-one *O*-perfluorobenzoyl oxime (2h). Colorless oil, 94%, 149 mg. ^{1}H NMR (400 MHz, $CDCl_3$) δ 7.35 - 7.03 (m, 10H), 3.80 (s, 1.12H), 3.67 (s, 0.88H), 2.62 (t, *J* = 7.6 Hz, 1.12H), 2.55 (t, *J* = 7.6 Hz, 0.88H), 2.39 - 2.32 (m, 2H), 1.94 - 1.87 (m, 1.12H), 1.77 - 1.69 (m, 0.88H); ^{13}C NMR (100 MHz, $CDCl_3$) δ 170.9, 169.7, 141.3, 140.9, 134.8, 134.4, 129.4 (2), 129.1 (2), 129.0 (2), 129.0 (2), 128.6 (2), 128.5 (2), 128.5 (2), 128.5 (2), 127.6, 127.4, 126.2, 126.2, 40.4, 35.9, 35.7, 35.4, 33.3, 28.9, 27.9, 27.6 (*peaks corresponding to the perfluorinated benzoyl ester moiety were not resolvable due to their anticipated weak intensity*); ^{19}F NMR (376 MHz, $CDCl_3$) δ -137.2 (m), -147.8 (m), -159.8 (m); IR (film): ν (cm^{-1}) 2929, 1757, 1651, 1523, 1496, 1454, 1421, 1324, 1194, 1092; HR-MS (ESI) *m/z* calcd for $C_{24}H_{18}F_5NNaO_2$ [M+Na^+]: 447.1150; found 447.1148.

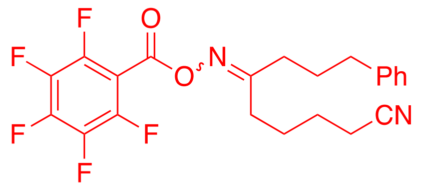

This compound was prepared using 6-oxo-9-phenylnonanenitrile (0.49 mmol) as starting material, purified by column chromatography on silica gel (petroleum ether/EtOAc 9:1) and obtained as a mixture of isomers ≈ 1:1.

6-(((Perfluorobenzoyl)oxy)imino)-9-phenylnonanenitrile (2i). Light yellow oil, 87%, 184 mg. ^{1}H NMR (400 MHz, $CDCl_3$) δ 7.31 - 7.13 (m, 10H), 2.71 (t, J = 7.6 Hz, 2H), 2.67 (t, J = 7.6 Hz, 2H), 2.46 - 2.34 (m, 12H), 2.00 - 1.83 (m, 4H), 1.82 - 1.66 (m, 8H); ^{13}C NMR (100 MHz, $CDCl_3$) δ 171.1, 170.8, 141.2, 140.8, 128.6 (4), 128.5 (2), 128.5 (2), 126.3, 126.3, 119.4, 119.1, 35.8, 35.4, 33.7, 33.4, 29.6, 29.2, 27.9, 27.7, 25.3, 25.0, 24.9 (2), 17.1, 17.0; (*Peaks corresponding to the perfluorinated benzoyl ester moiety were not resolvable due to their anticipated weak intensity*); ^{19}F NMR (376 MHz, $CDCl_3$) δ -137.3 (m), -148.0 (m), -160.0 (m); IR (film): ν (cm^{-1}) 2939, 2160, 1755, 1523, 1496, 1456, 1421, 1325, 1194, 1092; HR-MS (ESI) *m/z* calcd for $C_{22}H_{19}F_5KN_2O_2$ [M+K$^+$]: 477.0998; found 477.0997.

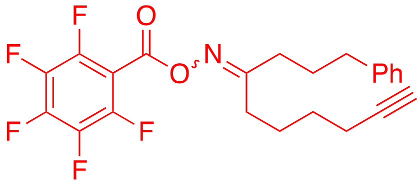

This compound was prepared using 1-phenyldec-9-yn-4-one (0.74 mmol) as starting material, purified by column chromatography on silica gel (petroleum ether/EtOAc 97:3) and obtained as a mixture of isomers ≈ 1:1.

1-Phenyldec-9-yn-4-one *O*-perfluorobenzoyl oxime (2j). Light yellow semisolid, 81%, 263 mg). ^{1}H NMR (400 MHz, $CDCl_3$) δ 7.31 - 7.13 (m, 10H), 2.70 (t, J = 7.6 Hz, 2H), 2.66 (t, J = 7.6 Hz, 2H), 2.47 - 2.39 (m, 8H), 2.23 (td, J = 7.2, 2.8 Hz, 2H), 2.20 (td, J = 6.8, 2.4 Hz, 2H), 2.00 - 1.83 (m, 6H), 1.76 - 1.50 (m, 8H); ^{13}C NMR (100 MHz, $CDCl_3$) δ 171.9, 171.7, 141.3, 141.0, 128.6 (2), 128.6 (2), 128.5 (2), 128.5 (2), 126.3, 126.2, 83.9, 83.6, 68.9 (2), 35.8, 35.5, 33.7, 33.7, 29.5, 29.4, 28.3, 28.0, 27.9, 27.7, 25.2, 25.0, 18.2, 18.1 (*peaks corresponding to the perfluorinated benzoyl ester moiety were not resolvable due to their anticipated weak intensity*); ^{19}F NMR (376 MHz, $CDCl_3$) δ -137.2 (m), -147.9 (m), -159.8 (m); IR (film): ν (cm^{-1}) 2941, 1757, 1651, 1522, 1496, 1325, 1194, 1090, 951; HR-MS (ESI) *m/z* calcd for $C_{23}H_{20}F_5NNaO_2$ [M+Na$^+$]: 460.1306; found 460.1338.

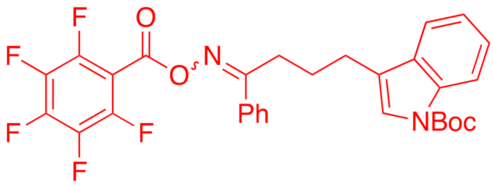

This compound was prepared using tert-butyl 3-(4-oxo-4-phenylbutyl)-1*H*-indole-1-carboxylate (1.15 mmol) as starting material, purified by column chromatography on silica gel (petroleum ether/EtOAc 98:2) and obtained as a mixture of isomers ≈ 9:1.

***tert*-Butyl 3-(4-(((perfluorobenzoyl)oxy)imino)-4-phenylbutyl)-1*H*-indole-1-**

carbo-xylate (2k). Colorless viscous oil, 63%, 414 mg. ^{1}H NMR (400 MHz, $CDCl_3$) δ 8.11 - 8.03 (*br* m, 1H), 7.74 - 7.71 (m, 1.8H), 7.51 - 7.46 (m, 0.9H), 7.44 - 7.24 (m, 5.3H), 7.22 - 7.14 (m, 1H), 3.02 - 2.98 (m, 1.8H), 2.86 - 2.83 (m, 0.2H), 2.79 - 2.74 (m, 2H), 2.07 - 1.91 (m, 2H), 1.67 (s, 9H); ^{13}C NMR (100 MHz, $CDCl_3$) δ *(Peaks correspond only to the major isomer)* 168.6, 149.9, 135.5, 133.1, 131.3, 130.4, 129.0 (2), 127.6 (2), 124.4, 122.8, 122.4, 119.6, 118.9, 115.3, 83.8, 28.4, 28.3 (3), 26.0, 24.7 (*peaks corresponding to the perfluorinated benzoyl ester moiety were not resolvable due to their anticipated weak intensity*); ^{19}F NMR (376 MHz, $CDCl_3$) δ -137.4 (m), -147.6 (m), -159.7 (m); IR (film): ν (cm^{-1}) 2979, 2933, 1763, 1728, 1498, 1452, 1371, 1324, 1254, 1190, 1155, 1090, 999; HR-MS (ESI) *m/z* calcd for $C_{30}H_{25}F_5N_2NaO_4$ [M+Na$^+$]: 595.1627; found 525.1622.

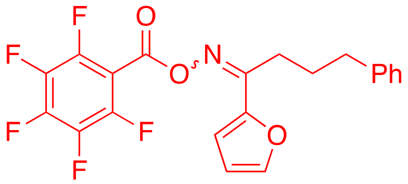

This compound was prepared using 1-(furan-2-yl)-4-phenylbutan-1-one (0.41 mmol) as starting material, purified by column chromatography on silica gel (petroleum ether/EtOAc 96:4) and obtained as a mixture of isomers ≈ 3:2.

1-(Furan-2-yl)-4-phenylbutan-1-one *O*-perfluorobenzoyl oxime (2l). White semisolid, 96%, 165 mg. Mp: 103 - 105 °C; ^{1}H NMR (400 MHz, $CDCl_3$) δ 7.58 (dd, *J* = 1.6, 0.8 Hz, 0.6H), 7.55 (dd, *J* = 1.6, 0.4 Hz, 0.4H), 7.30 - 7.14 (m, 5.4H), 6.88 (dd, *J* = 3.6, 0.4 Hz, 0.6H), 6.56 (dd, *J* = 3.6, 1.6 Hz, 0.4H), 6.51 (dd, *J* = 3.6, 1.6 Hz, 0.6H), 2.90 - 2.86 (m, 0.8H), 2.82 - 2.78 (m, 1.2H), 2.77 - 2.70 (m, 2H), 2.09 - 1.94 (m, 2H); ^{13}C NMR (100 MHz, $CDCl_3$) δ 159.8, 155.5, 147.2, 145.8, 144.8, 143.9, 141.6, 141.0, 128.6 (2), 128.5 (6), 126.3, 126.1, 120.9, 114.7, 112.9, 112.1, 35.7, 35.6, 31.7, 29.4, 28.7, 27.6 (*peaks corresponding to the perfluorinated benzoyl ester moiety were not resolvable due to their anticipated weak intensity*); ^{19}F NMR (376 MHz, $CDCl_3$) δ -137.0 (m), -147.5 (m), -159.7 (m); IR (film): ν (cm^{-1}) 2929, 1762, 1653, 1600, 1523, 1498, 1324, 1192, 1092, 1000; HR-MS (ESI) *m/z* calcd for $C_{21}H_{14}F_5NNaO$ [M+Na$^+$]: 446.0786; found 446.0784.

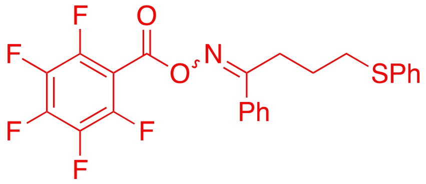

This compound was prepared using 1-phenyl-4-(phenylthio)butan-1-one (0.37 mmol) as starting material, purified by column chromatography on silica gel (petroleum ether/EtOAc 97:3) and obtained as a mixture of isomers ≈ 7:1.

1-Phenyl-4-(phenylthio)butan-1-one *O*-perfluorobenzoyl oxime (2m). White solid, 70%, 123 mg. Mp: 86 - 88 °C; ^{1}H NMR (400 MHz, $CDCl_3$) δ 7.74 - 7.71 (m, 1.75H), 7.52 - 7.40 (m, 3H), 7.30 - 7.14 (m, 5.25H), 3.08 - 3.04 (m, 1.75H), 2.99 (t, J = 7.2 Hz, 0.25H), 2.96 (t, J = 6.8 Hz, 1.75H), 2.92 - 2.88 (m, 0.25), 1.96 - 1.86 (m, 2H); ^{13}C NMR (100 MHz, $CDCl_3$) δ *(Peaks correspond only to the major isomer)* 168.0, 135.7, 132.9, 131.4, 129.5 (2), 129.1 (2), 129.0 (2), 127.6 (2), 126.4, 33.5, 27.7, 26.2 (*peaks corresponding to the perfluorinated benzoyl ester moiety were not resolvable due to their anticipated weak intensity*); ^{19}F NMR (376 MHz, $CDCl_3$) δ -137.0 (m), -147.4 (m), -159.6 (m); IR (film): ν (cm^{-1}) 1760, 1652, 1523, 1496, 1439, 1417, 1325, 1200, 1099, 1069, 1000; HR-MS (ESI) *m/z* calcd for $C_{23}H_{16}F_5NNaO_2S$ [M+Na$^+$]: 488.0714; found 488.0717.

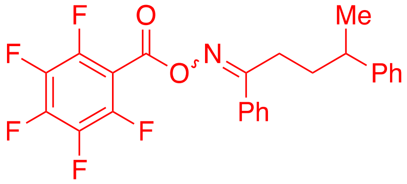

This compound was prepared using 1,4-diphenylpentan-1-one (0.57 mmol) as starting material, purified by column chromatography on silica gel (petroleum ether/EtOAc 98:2) and obtained as a mixture of isomers ≈ 4:1.

1,4-Diphenylpentan-1-one *O*-perfluorobenzoyl oxime (2n). White solid, 97%, 249 mg. Mp: 76 - 78°C; ^{1}H NMR (400 MHz, $CDCl_3$) δ 7.65 - 7.62 (m, 1.6H), 7.49 - 7.45 (m, 0.8H), 2.42 - 7.38 (m, 2H), 7.30 - 7.17 (m, 3.2H), 7.16 - 7.12 (m, 2.4H), 2.86 - 2.55 (m, 3H), 1.90 - 1.78 (m, 2H), 1.26 (d, J = 6.8 Hz, 3H); ^{13}C NMR (100 MHz, $CDCl_3$) δ *(Peaks correspond only to the major isomer)* 168.9, 145.9, 133.1, 131.2, 128.9 (2), 128.5 (2), 127.5 (2), 127.1 (2), 126.4, 40.4, 35.0, 27.2, 22.4 (*peaks corresponding to the perfluorinated benzoyl ester moiety were not resolvable due to their anticipated weak intensity*); ^{19}F NMR (376 MHz, $CDCl_3$) δ -137.1 (m), -147.8 (m), -159.8 (m); IR (film): ν (cm^{-1}) 2966, 1768, 1651, 1522, 1496, 1444, 1417, 1323, 1194, 1092; HR-MS (ESI) *m/z* calcd for $C_{24}H_{18}F_5NNaO_2$ [M+Na$^+$]: 470.1150; found 470.1156.

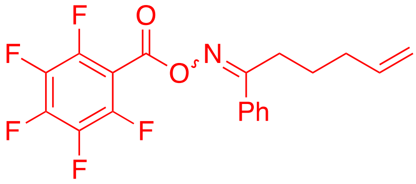

This compound was prepared using 1-phenylhex-5-en-1-one (0.56 mmol) as starting material, purified by column chromatography on silica gel (petroleum ether/EtOAc 98:2) and obtained as a mixture of isomers ≈ 9:1.

1-Phenylhex-5-en-1-one *O*-perfluorobenzoyl oxime (2o). Colorless oil, 95%, 205 mg.

^{1}H NMR (400 MHz, $CDCl_3$) δ 7.78 - 7.73 (m, 1.8H), 7.54 - 7.43 (m, 3H), 7.34 - 7.32 (m, 0.2H), 5.83 - 5.72 (m, 1H), 5.06 - 4.99 (m, 2H), 2.95 - 2.91 (m, 1.8H), 2.80 - 2.76 (m, 0.2H), 2.19 - 2.13 (m, 2H), 1.76 - 1.62 (m, 2H); ^{13}C NMR (100 MHz, $CDCl_3$) δ *(Peaks correspond only to the major isomer)* 168.9, 137.4, 133.3, 131.2, 129.0 (2), 127.6 (2), 115.9, 33.7, 28.4, 26.1 (*peaks corresponding to the perfluorinated benzoyl ester moiety were not resolvable due to their anticipated weak intensity*); ^{19}F NMR (376 MHz, $CDCl_3$) δ -137.1 (m), -147.6 (m), -159.8 (m); IR (film): ν (cm^{-1}) 3007, 2945, 1766, 1651, 1523, 1496, 1419, 1326, 1198, 1094, 999; HR-MS (ESI) *m/z* calcd for $C_{19}H_{14}F_5NNaO_2$ [M+Na$^+$]: 406.0837; found 406.0831.

Alkynyl nitriles

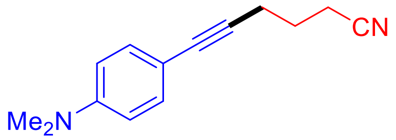

This compound was prepared following general procedure A using oxime ester **1a** (0.2 mmol) as starting material and purified by column chromatography on silica gel.

6-(4-(Dimethylamino)phenyl)hex-5-ynenitrile (4d).Yellow oil, 73%, 30.9 mg. ^{1}H NMR (400 MHz, $CDCl_3$) δ 7.27 (d, *J* = 8.0 Hz, 2H), 6.62 (d, *J* = 8.0 Hz, 2H), 2.97 (s, 6H), 2.60 - 2.55 (m, 4H), 1.98 - 1.91 (m, 2H); ^{13}C NMR (100 MHz, $CDCl_3$) δ 150.1, 132.7, 119.5, 112.0, 110.2, 84.4, 83.2, 40.4, 25.0, 18.8, 16.3; IR (film): ν (cm^{-1}) 2930, 1670, 1610, 1517, 1352, 1188, 1057, 947, 821, 717; HR-MS (ESI) *m/z* calcd for $C_{14}H_{17}N_2$ [M+H$^+$]: 213.1386; found 213.1388.

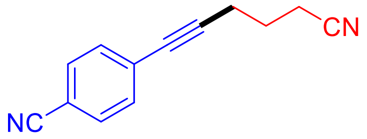

This compound was prepared following general procedure A using oxime ester **1a** (0.2 mmol) as starting material and purified by column chromatography on silica gel.

4-(5-Cyanopent-1-yn-1-yl)benzonitrile (4e). Yellow oil, 76%, 29.5 mg. ^{1}H NMR (400 MHz, $CDCl_3$) δ 7.59 - 7.57 (m, 2H), 7.47 – 7.45 (m, 2H), 2.63 (t, *J* = 6.8 Hz, 2H), 2.55 (t, *J* = 7.2 Hz, 2H), 1.97 (quint, *J* = 7.2 Hz, 2H); ^{13}C NMR (100 MHz, $CDCl_3$) δ 132.3, 132.1, 128.3, 119.0, 118.6, 111.5, 92.0, 81.1, 24.4, 18.7, 16.4; IR (film): ν (cm^{-1}) 2921, 2224, 1604, 1505, 1428, 1267, 1190, 1064, 852; HR-MS (ESI) *m/z* calcd for $C_{13}H_{11}N_2$ [M+H$^+$]: 195.0917; found 195.0915.

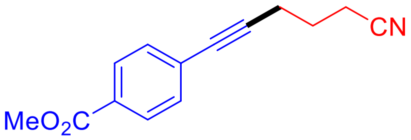

This compound was prepared following general procedure A using oxime ester **1a** (0.2 mmol) as starting material and purified by column chromatography on silica gel.

Methyl 4-(5-cyanopent-1-yn-1-yl)benzoate (4f). Yellow oil, 71%, 32.2 mg. ^{1}H NMR (400 MHz, $CDCl_3$) δ 7.97 – 7.94 (m, 2H), 7.45 - 7.43 (m, 2H), 3.90 (s, 3H), 2.62 (t, *J* = 6.8 Hz, 2H), 2.55 (t, *J* = 7.2 Hz, 2H), 1.96 (quint, *J* = 6.8 Hz, 2H); ^{13}C NMR (100 MHz, $CDCl_3$) δ 166.6, 131.6, 129.6, 129.4, 128.0, 119.1, 90.3, 81.9, 52.3, 24.6, 18.7, 16.4; IR (film): ν (cm^{-1}) 2950, 1720, 1604, 1435, 1275, 1103, 967, 863; HR-MS (ESI) *m/z* calcd for $C_{14}H_{14}NO_2$ [M+H$^+$]: 228.1019; found 228.1017.

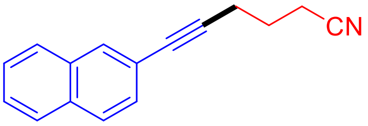

This compound was prepared following general procedure A using oxime ester **1a** (0.2 mmol) as starting material and purified by column chromatography on silica gel.

6-(Naphthalen-2-yl)hex-5-ynenitrile (4i). Yellow oil, 79%, 34.7 mg. ^{1}H NMR (400 MHz, $CDCl_3$) δ 7.94 (s, 1H), 7.80 - 7.77 (m, 3H), 7.50 - 7.46 (m, 3H), 2.66 - 2.63 (m, 2H), 2.58- 2.56 (m, 2H), 2.01 - 1.95 (m, 2H); ^{13}C NMR (100 MHz, $CDCl_3$) δ 133.0, 132.7, 131.3, 128.5, 128.0, 127.8, 127.7, 126.6, 126.6, 120.6, 119.3, 87.4, 82.8, 24.7, 18.7, 16.3; IR (film): ν (cm^{-1}) 2932, 2246, 1593, 1499, 1274, 1018, 897; HR-MS (EI) *m/z* calcd for $C_{16}H_{13}N$ [M$^+$]: 219.1043; found 219.1049.

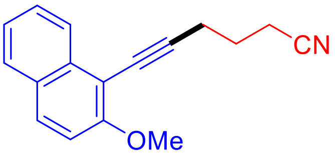

This compound was prepared following general procedure A using oxime ester **1a** (0.2 mmol) as starting material and purified by column chromatography on silica gel.

6-(2-Methoxynaphthalen-1-yl)hex-5-ynenitrile (4j). Yellow oil, 67%, 33.4 mg. ^{1}H NMR (400 MHz, $CDCl_3$) δ 8.19 (d, *J* = 6.4 Hz, 1H), 7.82 - 7.77 (m, 2H), 7.55 - 7.52 (m, 1H), 7.39 - 7.37 (m, 1H), 7.26 - 7.24 (m, 1H), 4.02 (s, 3H), 2.85 - 2.81 (m, 2H), 2.71 - 2.68 (m, 2H), 2.12 - 2.06 (m, 2H); ^{13}C NMR (100 MHz, $CDCl_3$) δ 159.1, 134.7, 129.9, 128.6, 128.2, 127.4, 125.2, 124.3, 119.5, 112.7, 106.3, 96.7, 76.9, 56.7, 25.1, 19.4, 16.3; IR (film): ν (cm^{-1}) 2924, 2163, 1812, 1593, 1385, 1270, 1073, 805; HR-MS

(ESI) *m/z* calcd for $C_{17}H_{16}NO$ [M+H$^+$]: 250.1226; found 250.1223.

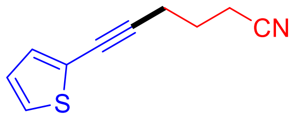

This compound was prepared following general procedure A using oxime ester **1a** (0.2 mmol) as starting material and purified by column chromatography on silica gel.

6-(Thiophen-2-yl)hex-5-ynenitrile (4k). Yellow oil, 79%, 27.7 mg. ^{1}H NMR (400 MHz, $CDCl_3$) δ 7.21 (d, J = 5.2 Hz, 1H), 7.16 (d, J = 3.2 Hz, 1H), 6.96 (dd, J = 4.8, 4.0 Hz, 1H), 2.63 (t, J = 6.4 Hz, 2H), 2.55 (t, J = 7.2 Hz, 2H), 2.00 - 1.93 (m, 2H); ^{13}C NMR (100 MHz, $CDCl_3$) δ 131.7, 127.0, 126.7, 123.3, 119.2, 91.1, 75.7, 24.6, 18.9, 16.4; IR (film): ν (cm^{-1}) 2925, 2157, 1790, 1424, 1194, 1046, 849, 701; HR-MS (ESI) *m/z* calcd for $C_{10}H_{10}NS$[M+H$^+$]: 176.0528; found 176.0524.

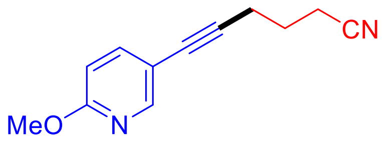

This compound was prepared following general procedure A using oxime ester **1a** (0.2 mmol) as starting material and purified by column chromatography on silica gel.
6-(6-Methoxypyridin-3-yl)hex-5-ynenitrile (4l). Yellow oil, 65%, 26.0 mg. ^{1}H NMR (400 MHz, $CDCl_3$) δ 8.21 (d, J = 2.4 Hz, 1H), 7.55 (dd, J = 8.8, 2.4 Hz, 1H), 6.67 (dd, J = 8.8, 0.4 Hz, 1H), 3.93 (s, 3H), 2.60 (t, J = 6.8 Hz, 2H), 2.55 (t, J = 7.2 Hz, 2H), 1.99 - 1.92 (m, 2H); ^{13}C NMR (100 MHz, $CDCl_3$) δ 163.3, 150.1, 141.3, 119.3, 113.1, 110.7, 88.4, 79.3, 53.7, 24.7, 18.7, 16.4; IR (film): ν (cm^{-1}) 2946, 2163, 1600, 1495, 1364, 1287, 1030, 832; HR-MS (ESI) *m/z* calcd for $C_{12}H_{13}N_2O$ [M+H$^+$]: 201.1022; found 201.1023.

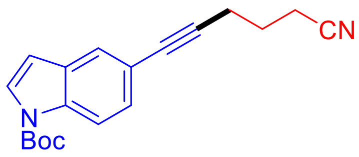

This compound was prepared following general procedure A using oxime ester **1a** (0.2 mmol) as starting material and purified by column chromatography on silica gel.

***tert*-Butyl 5-(5-cyanopent-1-yn-1-yl)-1*H*-indole-1-carboxylate (4m)**. Yellow oil, 70%, 43.4 mg. ^{1}H NMR (400 MHz, $CDCl_3$) δ 8.07 (d, J = 8.0 Hz, 1H), 7.62 (s, 1H), 7.59 (d, J = 3.6 Hz, 1H), 7.34 (d, J = 8.4 Hz, 1H), 6.53 (d, J = 3.6 Hz, 1H), 2.62 (t, J = 6.8 Hz, 2H), 2.59 (t, J = 7.6 Hz, 2H), 2.01 - 1.95 (m, 2H), 1.67 (s, 9H); ^{13}C NMR (100 MHz, $CDCl_3$) δ 149.6, 134.7, 130.6, 127.8, 126.9, 124.4, 119.4, 117.4, 115.2, 107.1,

85.5, 84.1, 83.0, 28.3, 24.9, 18.7, 16.4; IR (film): ν (cm^{-1}) 2970, 1736, 1462, 1364, 1270, 1161, 1002, 909, 843; HR-MS (ESI) *m/z* calcd for $C_{19}H_{20}N_2NaO_2$ [M+Na$^+$]: 331.1417; found 331.1415.

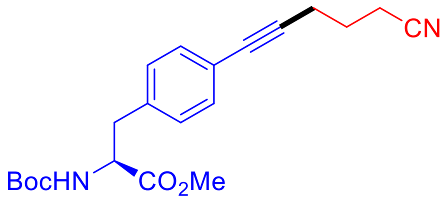

This compound was prepared following general procedure A using oxime ester **1a** (0.2 mmol) as starting material and purified by column chromatography on silica gel.

Methyl (*S*)-2-((*tert*-butoxycarbonyl)amino)-3-(4-(5-cyanopent-1-yn-1-yl)phenyl) propanoate (4n). Yellow oil, 60%, 43.4 mg. ^{1}H NMR (400 MHz, $CDCl_3$) δ 7.31(d, *J* = 8.0 Hz, 2H), 7.05 (d, *J* = 7.6 Hz, 2H), 4.98 (d, *J* = 7.2 Hz, 1H), 4.57 - 4.55 (m, 1H), 3.70 (s, 3H), 3.12 - 2.99 (m, 2H), 2.58 (t, *J* = 6.8 Hz, 2H), 2.54 (t, *J* = 7.2 Hz, 2H), 1.98 - 1.91 (m, 2H), 1.41 (s, 9H); ^{13}C NMR (100 MHz, $CDCl_3$) δ 172.2, 155.1, 136.2, 131.8, 129.4, 122.0, 119.3, 87.2, 82.2, 80.1, 54.4, 52.3, 38.3, 28.4, 24.7, 18.6, 16.3; IR (film): ν (cm^{-1}) 2960, 1719, 1518, 1364, 1250, 1158, 1027, 852; HR-MS (ESI) *m/z* calcd for $C_{21}H_{26}N_2NaO_4$ [M+Na$^+$]: 393.1785; found 393.1785.

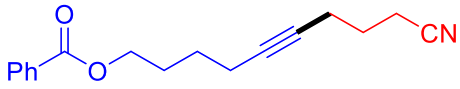

This compound was prepared following general procedure A using oxime ester **1a** (0.2 mmol) as starting material and purified by column chromatography on silica gel.

9-Cyanonon-5-yn-1-yl benzoate (4o). Yellow oil, 69%, 37.1 mg. ^{1}H NMR (400 MHz, $CDCl_3$) δ 8.04 (d, *J* = 7.2 Hz, 2H), 7.56 (t, *J* = 7.2 Hz,1H), 7.44 (t, *J* = 7.6 Hz,2H), 4.34 (t, *J* = 6.4 Hz,2H), 2.47 (t, *J* = 7.2 Hz,2H), 2.34 - 2.31 (m, 2H), 2.26 - 2.22 (m, 2H), 1.90 - 1.78 (m, 4H), 1.70 - 1.61 (m, 2H); ^{13}C NMR (100 MHz, $CDCl_3$) δ 166.7, 133.0, 130.4, 129.6, 128.5, 119.4, 81.7, 77.9, 64.6, 28.0, 25.5, 24.9, 18.5, 18.0, 16.2; IR (film): ν (cm^{-1}) 2940, 1734, 1435, 1275, 1120, 1021, 940, 812, 720; HR-MS (ESI) *m/z* calcd for $C_{17}H_{19}NNaO_2$ [M+Na$^+$]: 292.1308; found 292.1316.

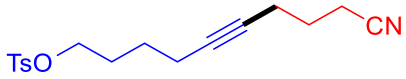

This compound was prepared following general procedure A using oxime ester **1a** (0.2 mmol) as starting material and purified by column chromatography on silica gel.

9-Cyanonon-5-yn-1-yl 4-methylbenzenesulfonate (4p). Yellow oil, 68%, 43.4 mg. ^{1}H

NMR (400 MHz, $CDCl_3$) δ 7.78 (d, *J* = 8.0 Hz,2H), 7.34 (d, *J* = 8.0 Hz, 2H), 4.04 (t, *J* = 6.8 Hz, 2H), 2.47 - 2.44 (m, 2H), 2.44 (s,3H), 2.30 (t, *J* = 6.4 Hz, 2H), 2.13 (t, *J* = 6.4 Hz, 2H), 1.84 - 1.71 (m, 4H), 1.55 - 1.49 (m, 2H); ^{13}C NMR (100 MHz, $CDCl_3$) δ 144.9, 133.2, 130.0, 128.0, 119.4, 81.3, 78.1, 70.1, 28.1, 24.9, 24.8, 21.8, 18.2, 18.0, 16.2; IR (film): ν (cm^{-1}) 2935, 1457, 1358, 1172, 1013, 925, 816, 730; HR-MS (ESI) *m/z* calcd for $C_{17}H_{22}NO_3S$ [M+H$^+$]: 320.1315; found 320.1320.

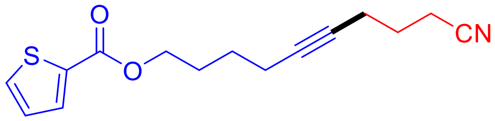

This compound was prepared following general procedure A using oxime ester **1a** (0.2 mmol) as starting material and purified by column chromatography on silica gel.

9-Cyanonon-5-yn-1-yl thiophene-2-carboxylate (4q).Yellow oil, 71%, 39.1 mg. ^1H NMR (400 MHz, $CDCl_3$) δ 7.80 (dd, *J* = 3.6, 0.8 Hz, 1H), 7.55 (dd, *J* = 4.8, 0.8 Hz, 1H), 7.10 (dd, *J* = 4.8, 4.4 Hz, 1H), 4.31 (t, *J* = 6.4 Hz, 2H), 2.47 (t, *J* = 7.2 Hz, 2H), 2.35 - 2.30 (m, 2H), 2.26 - 2.21 (m, 2H), 1.88 - 1.79 (m, 4H), 1.68 - 1.59 (m, 2H); ^{13}C NMR (100 MHz, $CDCl_3$) δ 162.4, 134.0, 133.4, 132.4, 127.9, 119.4, 81.7, 77.9, 64.8, 28.0, 25.5, 24.9, 18.5, 18.0, 16.2; IR (film): ν (cm^{-1}) 2930, 1703, 1419, 1260, 1096, 909, 832, 760; HR-MS (ESI) *m/z* calcd for $C_{15}H_{17}NNaO_2S$ [M+Na$^+$]: 298.0872; found 298.0873.

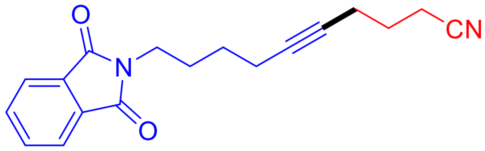

This compound was prepared following general procedure A using oxime ester **1a** (0.2 mmol) as starting material and purified by column chromatography on silica gel.

10-(1,3-Dioxoisoindolin-2-yl)dec-5-ynenitrile (4r). Yellow oil, 70%, 41.2 mg. ^1H NMR (400 MHz, $CDCl_3$) δ 7.84 - 7.82 (m, 2H), 7.71 - 7.69 (m, 2H), 3.69 (t, *J* = 7.2 Hz, 2H), 2.46 (t, *J* = 6.8 Hz, 2H), 2.32 - 2.29 (m, 2H), 2.21 - 2.18 (m, 2H), 1.84 - 1.74 (m, 4H), 1.55 - 1.48 (m, 2H); ^{13}C NMR (100 MHz, $CDCl_3$) δ 168.5, 134.0, 132.2, 123.3, 119.5, 81.6, 77.9, 37.6, 27.8, 26.1, 25.0, 18.3, 18.0, 16.2; IR (film): ν (cm^{-1}) 2940, 1768, 1702, 1392, 1196, 1032, 923, 787; HR-MS (ESI) *m/z* calcd for $C_{18}H_{19}N_2O_2$ [M+H$^+$]: 295.1441; found295.1445.

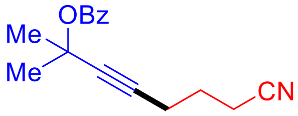

This compound was prepared following general procedure A using oxime ester **1a** (0.2

mmol) as starting material and purified by column chromatography on silica gel.

7-Cyano-2-methylhept-3-yn-2-yl benzoate (4s). Yellow oil, 75%, 38.5 mg. ^{1}H NMR (400 MHz, $CDCl_3$) δ 7.81(d, *J* = 7.2 Hz, 2H), 7.54 (t, *J* = 7.2 Hz, 1H), 7.43 (t, *J* = 8.0 Hz, 2H), 2.52 (t, *J* = 7.2 Hz, 2H), 2.40 (t, *J* = 6.8 Hz, 2H), 1.89 - 1.82 (m, 2H), 1.78 (s, 6H); ^{13}C NMR (100 MHz, $CDCl_3$) δ 164.9, 132.9, 131.2, 129.6, 128.4, 119.5, 83.6, 82.1, 72.8, 29.4, 24.6, 17.9, 16.0; IR (film): ν (cm^{-1}) 2936, 1720, 1456, 1282, 1106, 1068, 914, 838, 712; HR-MS (ESI) *m/z* calcd for $C_{16}H_{17}NNaO_2$ [M+Na$^+$]: 278.1151; found 278.1152.

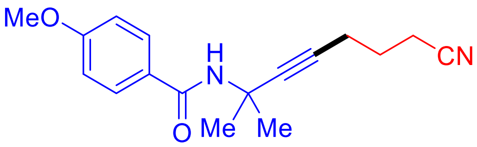

This compound was prepared following general procedure A using oxime ester **1a** (0.2 mmol) as starting material and purified by column chromatography on silica gel.

***N*-(7-Cyano-2-methylhept-3-yn-2-yl)-4-methoxybenzamide (4t)**. Yellow oil, 74%, 42.0 mg. ^{1}H NMR (400 MHz, $CDCl_3$) δ 7.70 (d, *J* = 8.4 Hz, 2H), 6.89 (d, *J* = 8.4 Hz, 2H), 6.18 (*br* s, 1H), 3.82 (s, 3H), 2.52 (t, *J* = 6.8 Hz, 2H), 2.37 (t, *J* = 6.8 Hz, 2H), 1.87 - 1.80 (m, 2H), 1.70(s, 6H); ^{13}C NMR (100 MHz, $CDCl_3$) δ 166.1, 162.2, 128.8, 127.4, 119.6, 113.7, 86.0, 78.5, 55.5, 48.2, 29.4, 24.5, 17.9, 16.1; IR (film): ν (cm^{-1}) 2931, 1642, 1502, 1292, 1253, 1183, 1027, 843, 763; HR-MS (ESI) *m/z* calcd for $C_{17}H_{20}N_2NaO_2$ [M+Na$^+$]: 307.1417; found 307.1421.

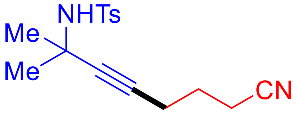

This compound was prepared following general procedure A using oxime ester **1a** (0.2 mmol) as starting material and purified by column chromatography on silica gel.

***N*-(7-Cyano-2-methylhept-3-yn-2-yl)-4-methylbenzenesulfonamide (4u)**. Yellow oil, 72%, 43.8 mg. ^{1}H NMR (400 MHz, $CDCl_3$) δ 7.79(d, *J* = 7.6 Hz, 2H), 7.30 (d, *J* = 7.6 Hz, 2H), 4.91 (*br* s, 1H), 2.43 (s, 3H), 2.32 (t, *J* = 6.8 Hz, 2H), 2.05 (t, *J* = 6.4 Hz, 2H), 1.62 - 1.58 (m, 2H), 1.51 (s, 6H); ^{13}C NMR (100 MHz, $CDCl_3$) δ 143.3, 139.3, 129.5, 127.6, 119.3, 84.0, 80.8, 50.3, 31.3, 24.3, 21.6, 17.7, 16.1; IR (film): ν (cm^{-1}) 2924, 1418, 1320, 1150, 1090, 986, 816,717; HR-MS (ESI) *m/z* calcd for $C_{16}H_{20}N_2NaO_2S$ [M+Na$^+$]: 327.1138; found 327.1145.

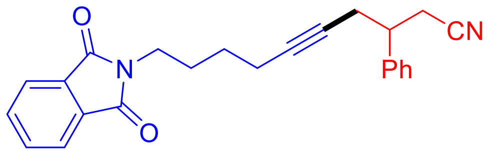

This compound was prepared following general procedure A using oxime ester **1b** (0.2 mmol) as starting material and purified by column chromatography on silica gel.

10-(1,3-Dioxoisoindolin-2-yl)-3-phenyldec-5-ynenitrile (4v). Yellow oil, 68%, 50.3 mg. ^{1}H NMR (400 MHz, $CDCl_3$) δ 7.88 - 7.84 (m, 2H), 7.74 - 7.70 (m, 2H), 7.36 - 7.32 (m, 2H), 7.27 - 7.25 (m, 3H), 3.69 (t, J = 7.2 Hz, 2H), 3.20 - 3.13 (m, 1H), 2.88 (dd, J = 166.8, 6.0 Hz, 1H), 2.74 (dd, J = 16.4, 8.0 Hz, 1H), 2.65 - 2.54 (m, 2H), 2.24 -2.19 (m, 2H), 1.79 - 1.71 (m, 2H), 1.55 - 1.48 (m, 2H); ^{13}C NMR (100 MHz, $CDCl_3$) δ 168.5, 140.8, 134.1, 132.2, 128.9, 127.8, 127.1, 123.3, 118.5, 82.8, 77.0, 41.4, 37.6, 27.7, 26.0, 25.3, 23.3, 18.3; IR (film): ν (cm^{-1}) 2931, 1703, 1397, 1189, 1035, 914, 767; HR-MS (ESI) *m/z* calcd for $C_{24}H_{23}N_2O_2$ [M+H$^+$]: 371.1754; found 371.1755.

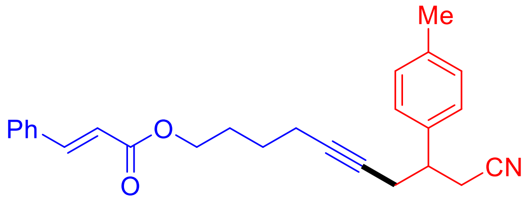

This compound was prepared following general procedure A using oxime ester **1c** (0.2 mmol) as starting material and purified by column chromatography on silica gel.

9-Cyano-8-(*p*-tolyl)non-5-yn-1-yl cinnamate (4w). Yellow oil, 51%, 39.3 mg. ^{1}H NMR (400 MHz, $CDCl_3$) δ 7.70 (d, J = 16.0 Hz, 1H), 7.54 - 7.53 (m, 2H), 7.40 - 7.39 (m, 3H), 7.10 - 7.05 (m, 4H), 6.45 (d, J = 16.0 Hz, 1H), 4.21 (t, J = 6.4 Hz, 2H), 3.17 - 3.10 (m, 1H), 2.86 (dd, J = 12.8, 6.0 Hz, 1H), 2.72 (dd, J = 12.8, 8.0 Hz, 1H), 2.61 - 2.59 (m, 2H), 2.33 (s, 3H), 2.25 - 2.22 (m, 2H), 1.80 - 1.73 (m, 2H), 1.62 - 1.58 (m, 2H); ^{13}C NMR (100 MHz, $CDCl_3$) δ 167.2, 144.9, 137.8, 137.5, 134.5, 130.4, 129.6, 129.0, 128.2, 127.0, 118.6, 118.2, 82.8, 77.1, 64.2, 41.1, 27.9, 25.43, 25.35, 23.4, 21.2, 18.5; IR (film): ν (cm^{-1}) 2930, 2360, 1709, 1637, 1517, 1457, 1315, 1166, 1035, 767; HR-MS (ESI) *m/z* calcd for $C_{26}H_{27}NNaO_2$ [M+Na$^+$]: 408.1934; found 408.1926.

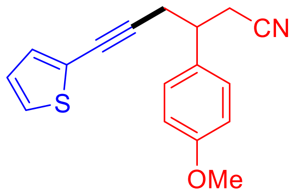

This compound was prepared following general procedure A using oxime ester **1d** (0.2 mmol) as starting material and purified by column chromatography on silica gel.

3-(4-Methoxyphenyl)-6-(thiophen-2-yl)hex-5-ynenitrile (4x). Yellow oil, 70%, 39.3 mg. ^{1}H NMR (400 MHz, $CDCl_3$) δ 7.24 - 7.14 (m, 4H), 6.97 - 6.90 (m, 3H), 3.81 (s, 3H), 3.30 - 3.23 (m, 1H), 2.94 - 2.85 (m, 3H), 2.78 (dd, *J* = 8.0, 7.6 Hz, 1H); ^{13}C NMR (100 MHz, $CDCl_3$) δ 159.3, 132.4, 131.8, 128.2, 127.0, 126.8, 123.2, 118.4, 114.4, 90.3, 76.8, 55.4, 40.5, 26.4, 23.6; IR (film): ν (cm^{-1}) 2920, 1616, 1518, 1429, 1248, 1183, 1029, 832, 712; HR-MS (ESI) *m/z* calcd for $C_{17}H_{16}NOS$ [M+H$^+$]: 282.0947; found 282.0950.

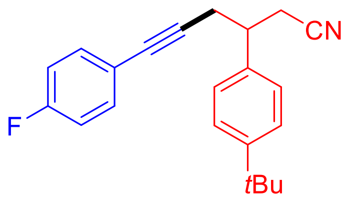

This compound was prepared following general procedure A using oxime ester **1e** (0.2 mmol) as starting material and purified by column chromatography on silica gel.

3-(4-(*tert*-Butyl)phenyl)-6-(4-fluorophenyl)hex-5-ynenitrile (4y). Yellow oil, 65%, 41.5 mg. ^{1}H NMR (400 MHz, $CDCl_3$) δ 7.40 (d, *J* = 8.0 Hz, 2H), 7.39 - 7.34 (m, 2H), 7.25 (d, *J* = 8.0 Hz, 2H), 7.02 - 6.98 (m, 2H), 3.32 - 3.25 (m, 1H), 2.94 (dd, *J* = 12.8, 6.0 Hz, 1H), 2.88 - 2.79 (m, 3H), 1.35 (s, 9H); ^{13}C NMR (100 MHz, $CDCl_3$) δ 162.5 (d, *J* = 250.5 Hz), 150.9, 137.5, 133.6 (d, *J* = 8.1 Hz), 126.8, 125.9, 119.3 (d, *J* = 3.7 Hz), 118.5, 115.7 (d, *J* = 22.1 Hz), 86.0, 82.5, 40.8, 34.7, 31.4, 25.8, 23.4; IR (film): ν (cm^{-1}) 2956, 1506, 1364, 1221, 1162, 1013, 925, 838; HR-MS (ESI) *m/z* calcd for $C_{22}H_{23}FN$ [M+H$^+$]: 320.1809; found320.1810.

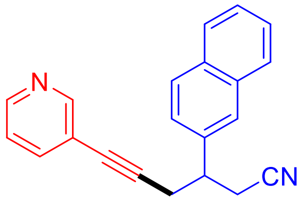

This compound was prepared following general procedure A using oxime ester **1f** (0.2 mmol) as starting material and purified by column chromatography on silica gel.

3-(Naphthalen-2-yl)-6-(pyridin-3-yl)hex-5-ynenitrile (4z). Yellow oil, 73%, 43.2 mg. ^{1}H NMR (400 MHz, $CDCl_3$) δ 8.60 (s, 1H), 8.50 (d, *J* = 3.6 Hz, 1H), 7.89 - 7.83 (m, 3H), 7.77 (s, 1H), 7.63 (d, *J* = 7.6 Hz, 1H), 7.53 - 7.48 (m, 2H), 7.43 - 7.41 (m, 1H), 7.22 - 7.18 (m, 1H), 3.52 - 3.45 (m, 1H), 3.04 - 2.97 (m, 3H), 2.95 - 2.88 (m, 1H); ^{13}C NMR (100 MHz, $CDCl_3$) δ 152.4, 148.6, 138.6, 137.6, 133.4, 133.0, 128.9, 127.9, 127.8, 126.6, 126.3, 126.1, 124.8, 123.1, 120.2, 118.2, 89.7, 80.4, 41.2, 25.9, 23.3; IR (film): ν (cm^{-1}) 2940, 2261, 1478, 1413, 1270, 1183, 1018, 821, 750; HR-MS (ESI) *m/z* calcd for $C_{21}H_{17}N_2$ [M+H$^+$]: 297.1386; found 297.1381.

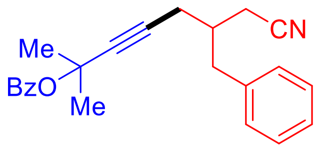

This compound was prepared following general procedure A using oxime ester **1g** (0.2 mmol) as starting material and purified by column chromatography on silica gel.

6-Benzyl-7-cyano-2-methylhept-3-yn-2-yl benzoate (4aa). Yellow oil, 68%, 46.9 mg. ^{1}H NMR (400 MHz, $CDCl_3$) δ 8.07 - 8.05 (m, 2H), 7.61 - 7.57 (m, 1H), 7.47 (t, J = 8.0 Hz, 2H), 7.35 - 7.21 (m, 5H), 2.89 - 2.79 (m, 2H), 2.59 -2.48 (m, 2H), 2.45 (dd, J = 16.8, 4.8 Hz, 1H), 2.33 (dd, J = 16.8, 6.8 Hz, 1H), 2.28 - 2.21 (m, 1H), 1.85 (s, 3H), 1.85 (s, 3H); ^{13}C NMR (100 MHz, $CDCl_3$) δ 165.0, 138.5, 132.9, 131.1, 129.6, 129.2, 128.8, 128.4, 126.8, 118.6, 84.7, 81.0, 72.8, 39.0, 37.1, 29.4, 29.4, 22.7, 20.9; IR (film): ν (cm^{-1}) 2921, 2020, 1720, 1451, 1282, 1006, 938, 834, 757; HR-MS (ESI) *m/z* calcd for $C_{23}H_{23}NNaO_2$ [M+Na$^+$]: 368.1621; found 368.1621.

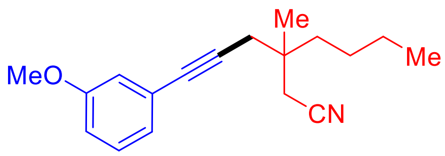

This compound was prepared following general procedure A using oxime ester **1h** (0.2 mmol) as starting material and purified by column chromatography on silica gel.

3-(3-(3-Methoxyphenyl)prop-2-yn-1-yl)-3-methylheptanenitrile (4ab). Yellow oil, 60%, 32.3 mg. ^{1}H NMR (400 MHz, $CDCl_3$) δ 7.21 (t, J = 8.0 Hz,1H), 7.00 (d, J = 7.6 Hz,1H), 6.93 (s, 1H), 6.86 (dd, J = 8.0, 2.0 Hz, 1H), 3.80 (s, 3H), 2.51 - 2.42 (m, 4H), 1.55 - 1.50 (m, 2H), 1.39 - 1.26 (m, 4H), 1.17 (s, 3H), 0.94 (t, J = 6.8 Hz, 3H); ^{13}C NMR (100 MHz, $CDCl_3$) δ 159.4, 129.5, 124.5, 124.3, 118.3, 116.7, 114.6, 85.7, 83.6, 55.4, 38.8, 36.5, 30.2, 28.2, 26.1, 24.3, 23.3, 14.1; IR (film): ν (cm^{-1}) 2935, 1600, 1462, 1423, 1287, 1168, 1046, 860, 783; HR-MS (ESI) *m/z* calcd for $C_{18}H_{24}NO$ [M+H$^+$]: 270.1852; found 270.1855.

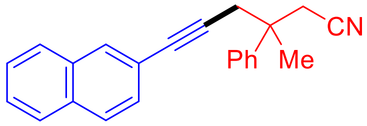

This compound was prepared following general procedure A using oxime ester **1i** (0.2 mmol) as starting material and purified by column chromatography on silica gel.

3-Methyl-6-(naphthalen-2-yl)-3-phenylhex-5-ynenitrile (4ac). Yellow oil, 64%, 39.9 mg. ^{1}H NMR (400 MHz, $CDCl_3$) δ 7.88 (s, 1H), 7.83 - 7.75 (m, 3H), 7.50 - 7.39 (m,

7H), 7.33 (t, J = 6.8 Hz, 1H), 3.01 - 2.90 (m, 4H), 1.72 (s, 3H); ^{13}C NMR (100 MHz, $CDCl_3$) δ 143.6, 133.0, 132.8, 131.4, 128.9, 128.6, 128.1, 127.9, 127.7, 127.5, 126.7, 126.7, 125.7, 120.5, 118.1, 86.0, 84.6, 40.6, 32.8, 29.9, 25.8; IR (film): ν (cm^{-1}) 2972, 2246, 1599, 1499, 1267, 1024, 891; HR-MS (ESI) *m/z* calcd for $C_{23}H_{20}N$ [M+H$^+$]: 310.1590; found 310.1603.

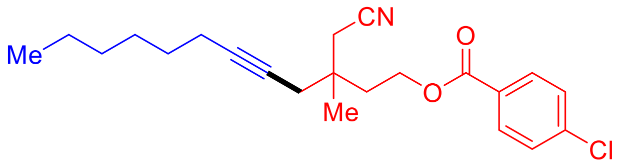

This compound was prepared following general procedure A using oxime ester **1j** (0.2 mmol) as starting material and purified by column chromatography on silica gel.

3-(Cyanomethyl)-3-methyldodec-5-yn-1-yl 4-chlorobenzoate (4ad). Yellow oil, 64%, 48.2 mg. ^{1}H NMR (400 MHz, $CDCl_3$) δ 7.98 (d, J = 8.0 Hz, 2H), 7.42 (d, J = 8.0 Hz, 2H), 4.41 (t, J = 6.0 Hz, 2H), 2.52 - 2.43 (m, 2H), 2.36 - 2.26 (m, 2H), 2.18 - 2.10 (m, 2H), 2.03 - 1.92 (m, 2H), 1.48 - 1.43 (m, 2H), 1.35 - 1.27 (m, 6H), 1.21 (s, 3H), 0.87 (t, J = 6.0 Hz, 3H); ^{13}C NMR (100 MHz, $CDCl_3$) δ 165.7, 139.7, 131.1, 128.9, 128.5, 118.0, 84.5, 75.1, 61.5, 36.8, 35.7, 31.4, 30.1, 29.0, 28.7, 28.6, 24.4, 22.7, 18.8, 14.1; IR (film): ν (cm^{-1}) 2935, 1724, 1593, 1468, 1277, 1103, 1016, 852, 760; HR-MS (ESI) *m/z* calcd for $C_{22}H_{28}ClNNaO_2$ [M+Na$^+$]: 396.1701; found 396.1701.

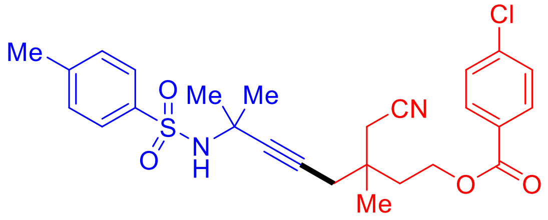

This compound was prepared following general procedure A using oxime ester **1j** (0.2 mmol) as starting material and purified by column chromatography on silica gel.

3-(Cyanomethyl)-3,7-dimethyl-7-((4-methylphenyl)sulfonamido)oct-5-yn-1-yl 4-chlorobenzoate (4ae). Yellow oil, 61%, 61.0 mg. ^{1}H NMR (400 MHz, $CDCl_3$) δ 7.97 (d, J = 8.4 Hz, 2H), 7.77 (d, J = 8.4 Hz, 2H), 7.42 (d, J = 8.4 Hz, 2H), 7.28 (d, J = 8.0 Hz, 2H), 5.25 (*br* s, 1H), 4.34 (t, J = 6.8 Hz, 2H), 2.40 (s, 3H), 2.38 (s, 2H), 2.12 (d, J = 16.9 Hz, 1H), 2.06 (d, J = 16.9 Hz, 1H), 1.93 - 1.84 (m, 2H), 1.49 (s, 6H), 1.11 (s, 3H); ^{13}C NMR (100 MHz, $CDCl_3$) δ 165.7, 143.2, 139.8, 139.4, 131.1, 129.5, 129.0, 128.4, 127.4, 117.9, 86.3, 78.6, 61.4, 50.4, 36.8, 35.5, 31.1, 29.6, 28.4, 24.4, 21.6; IR (film): ν (cm^{-1}) 2923, 1714, 1599, 1435, 1325, 1270, 1101, 997, 865, 756; HR-MS (ESI) *m/z* calcd for $C_{26}H_{29}ClN_2NaO_4S$ [M+Na$^+$]: 523.1429; found 523.1432.

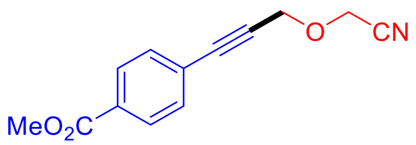

This compound was prepared following general procedure A using oxime ester **1k** (0.2 mmol) as starting material and purified by column chromatography on silica gel.

Methyl 4-(3-(cyanomethoxy)prop-1-yn-1-yl)benzoate (4af). Yellow oil, 62%, 28.4 mg. ^{1}H NMR (400 MHz, $CDCl_3$) δ 8.02 - 8.00 (m, 2H), 7.54 - 7.52 (m, 2H), 4.57 (s, 2H), 4.44 (s, 2H), 3.93 (s, 3H); ^{13}C NMR (100 MHz, $CDCl_3$) δ 166.5, 131.9, 130.5, 129.7, 126.5, 115.6, 87.9, 85.1, 59.0, 54.5, 52.5; IR (film): ν (cm^{-1}) 2931, 1660, 1518, 1479, 1375, 1224, 1150, 1046, 876, 745; HR-MS (ESI) *m/z* calcd for $C_{13}H_{12}NO_3$ [M+H^{+}]: 230.0812; found 230.0806.

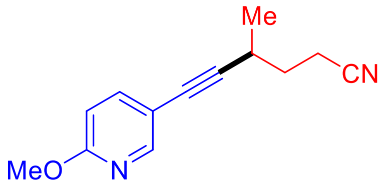

This compound was prepared following general procedure A using oxime ester **1l** (0.2 mmol) as starting material and purified by column chromatography on silica gel.

6-(6-Methoxypyridin-3-yl)-4-methylhex-5-ynenitrile (4ag). Yellow oil, 69%, 29.5 mg. ^{1}H NMR (400 MHz, $CDCl_3$) δ 8.20 (d, *J* = 2.0 Hz, 1H), 7.56 (dd, *J* = 8.4, 2.0 Hz, 1H), 6.67 (d, *J* = 8.8 Hz, 1H), 3.93 (s, 3H), 2.84 - 2.82 (m, 1H), 2.59 - 2.54 (m, 2H), 1.92 - 1.80 (m, 2H), 1.31 (d, *J* = 6.8 Hz, 3H); ^{13}C NMR (100 MHz, $CDCl_3$) δ 163.4, 150.1, 141.4, 119.6, 113.1, 110.7, 92.8, 79.4, 53.7, 32.5, 26.2, 20.8, 15.6; IR (film): ν (cm^{-1}) 2940, 1600, 1489, 1369, 1287, 1019, 1046, 827, 717; HR-MS (ESI) *m/z* calcd for $C_{13}H_{15}N_2O$ [M+H^{+}]: 215.1179; found 215.1174.

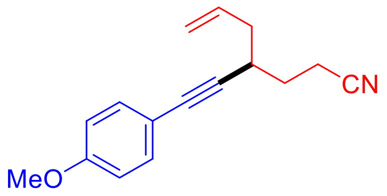

This compound was prepared following general procedure A using oxime ester **1m** (0.2 mmol) as starting material and purified by column chromatography on silica gel.

4-((4-Methoxyphenyl)ethynyl)hept-6-enenitrile (4ah). Yellow oil, 74%, 35.4 mg. ^{1}H NMR (400 MHz, $CDCl_3$) δ 7.35 - 7.32 (m, 2H), 6.85 - 6.81 (m, 2H), 5.96 - 5.86 (m, 1H), 5.18 - 5.12 (m, 2H), 3.81 (s, 3H), 2.82 - 2. 75 (m, 1H), 2.62 - 2.54 (m, 2H), 2.38 -

2.33 (m, 2H), 1.98 - 1.90 (m, 1H), 1.84 - 1.74 (m, 1H); ^{13}C NMR (100 MHz, $CDCl_3$) δ 159.6, 135.0, 133.1, 119.7, 117.7, 115.3, 114.0, 88.3, 83.7, 55.4, 39.2, 31.6, 30.3, 15.5; IR (film): ν (cm^{-1}) 2939, 1610, 1512, 1446, 1294, 1179, 1027, 912, 814; HR-MS (ESI) *m/z* calcd for $C_{16}H_{18}NO$ [M+H$^+$]: 240.1383; found 240.1386.

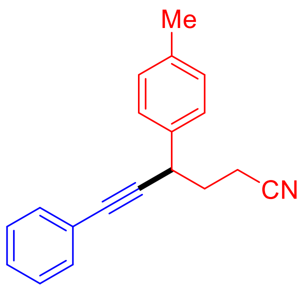

This compound was prepared following general procedure A using oxime ester **1n** (0.2 mmol) as starting material and purified by column chromatography on silica gel.

6-Phenyl-4-(*p*-tolyl)hex-5-ynenitrile (4ai). Yellow oil, 57%, 29.5 mg.^{1}H NMR (400 MHz, $CDCl_3$) δ 7.47 - 7.45 (m, 2H), 7.33 - 7.31 (m, 5H), 7.19 (d, *J* = 7.6 Hz, 2H), 4.02 (t, *J* = 7.6 Hz, 1H), 2.60 (dt, *J* = 16.8, 8.0 Hz, 1H), 2.46 (dt, *J* = 17.2, 7.2 Hz, 1H), 2.37 (s, 3H), 2.22 - 2.13 (m, 2H); ^{13}C NMR (100 MHz, $CDCl_3$) δ 136.3, 135.8, 130.8, 128.7, 127.5, 127.4, 126.4, 122.1, 118.4, 88.0, 83.8, 36.0, 32.9, 20.2, 14.2; IR (film): ν (cm^{-1}) 2945, 1517, 1484, 1365, 1014, 816, 752; HR-MS (ESI) *m/z* calcd for $C_{19}H_{18}N$ [M+H$^+$]: 260.1434; found 260.1428.

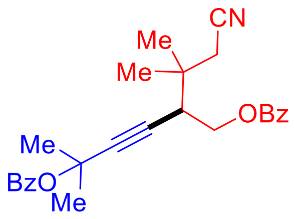

This compound was prepared following general procedure A using oxime ester **1o** (0.2 mmol) as starting material and purified by column chromatography on silica gel.

2-(1-Cano-2-methylpropan-2-yl)-5-methylhex-3-yne-1,5-diyl dibenzoate (4aj). Yellow oil, 60%, 50.0 mg. ^{1}H NMR (400 MHz, $CDCl_3$) δ 8.04 (d, *J* = 7.6 Hz, 2H), 7.97 (d, *J* = 7.6 Hz, 2H), 7.55 (t, *J* = 7.2 Hz, 2H), 7.42 (t, *J* = 8.0 Hz, 2H), 7.40 (t, *J* = 8.0 Hz, 2H), 4.49 (dd, *J* = 11.2, 5.6 Hz, 1H), 4.39 (dd, *J* = 11.2, 7.6 Hz, 1H), 2.93 (t, *J* = 6.4 Hz, 1H), 2.70 (d, *J* = 16.4 Hz, 1H), 2.52 (d, *J* = 16.4 Hz, 1H), 1.75 (s, 3H), 1.74 (s, 3H), 1.27 (s, 3H), 1.23 (s, 3H); ^{13}C NMR (100 MHz, $CDCl_3$) δ 166.3, 164.9, 133.3, 132.9, 131.1, 129.9, 129.8, 129.6, 128.5, 128.4, 118.3, 86.5, 81.7, 72.5, 63.5, 41.2, 36.0, 29.9, 29.3, 29.2, 25.5, 24.0; IR (film): ν (cm^{-1}) 2960, 1720, 1386, 1277, 1107, 1024, 854; HR-MS (ESI) *m/z* calcd for $C_{26}H_{27}NNaO_4$ [M+Na$^+$]: 440.1832; found 440.1836.

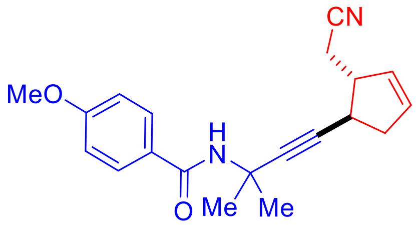

This compound was prepared following general procedure A using oxime ester **1p** (0.2 mmol) as starting material and purified by column chromatography on silica gel.

***N*-(4-((1*R*,2*R*)-2-(Cyanomethyl)cyclopent-3-en-1-yl)-2-methylbut-3-yn-2-yl)-4-methoxybenzamide (4ak)**. Yellow oil, 76%, 48.9 mg. ^{1}H NMR (400 MHz, $CDCl_3$) δ 7.72 - 7.70 (m, 2H), 6.91- 6.89 (m, 2H), 6.21 (*br* s, 1H), 5.85 - 5.83 (m, 1H), 5.64 - 5.63 (m, 1H), 3.83 (s, 3H), 3.04 - 3.02 (m, 1H), 2.80 - 2.74 (m, 1H), 2.72 - 2.66 (m, 1H), 2.59 (dd, *J* = 16.8, 6.0 Hz, 1H), 2.52 - 2.45 (m, 2H), 1.72 (s, 6H); ^{13}C NMR (100 MHz, $CDCl_3$) δ 166.1, 162.2, 132.4, 130.6, 128.8, 127.5, 118.4, 113.7, 85.6, 82.3, 55.5, 49.7, 48.6, 40.1, 35.0, 29.3, 29.3, 22.1; IR (film): ν (cm^{-1}) 2930, 1643, 1501, 1303, 1254, 1030, 953, 843; HR-MS (ESI) *m/z* calcd for $C_{20}H_{23}N_2O_2$ [M+H$^+$]: 323.1754; found 323.1750.

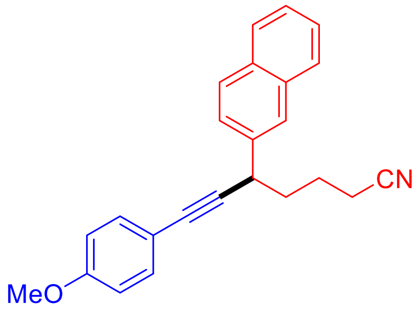

This compound was prepared following general procedure A using oxime ester **1q** (0.2 mmol) as starting material and purified by column chromatography on silica gel.

7-(4-Methoxyphenyl)-5-(naphthalen-2-yl)hept-6-ynenitrile (4al). Yellow oil, 57%, 38.6 mg. ^{1}H NMR (400 MHz, $CDCl_3$) δ 7.88 (s, 1H), 7.87 - 7.84 (m, 3H), 7.55 (dd, *J* = 8.4, 1.6 Hz, 1H), 7.53 - 7.48 (m, 2H), 7.45 - 7.42 (m, 2H), 6.89 - 6.85 (m, 2H), 4.08 (dd, *J* = 8.0, 7.6 Hz, 1H), 3.83 (s, 3H), 2.40 (t, *J* = 7.2 Hz, 2H), 2.09 - 2.04 (m, 2H), 1.94 - 1.88 (m, 2H); ^{13}C NMR (100 MHz, $CDCl_3$) δ 159.6, 138.7, 133.5, 133.2, 132.7, 128.6, 127.9, 127.8, 126.4, 126.1, 125.9, 125.7, 119.6, 115.5, 88.6, 84.3, 55.4, 37.9, 37.0, 23.3, 17.1; IR (film): ν (cm^{-1}) 2935, 1605, 1511, 1256, 1172, 1054, 909, 832, 734; HR-MS (ESI) *m/z* calcd for $C_{22}H_{21}NNaO$ [M+Na$^+$]: 576.4775; found 576.4783.

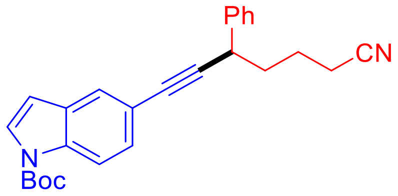

This compound was prepared following general procedure A using oxime ester **1r** (0.2 mmol) as starting material and purified by column chromatography on silica gel.

***tert*-Butyl (*R*)-5-(6-cyano-3-phenylhex-1-yn-1-yl)-1*H*-indole-1-carboxylate (4am)**. Yellow oil, 50%, 39.8 mg. ^{1}H NMR (400 MHz, $CDCl_3$) δ 8.07 (d, J = 8.4 Hz, 1H), 7.66 (s, 1H), 7.59 (d, J = 3.2 Hz, 1H), 7.44 (d, J = 7.2 Hz, 2H), 7.40 - 7.34 (m, 3H), 7.28 - 7.25 (m, 1H), 6.53 (d, J = 6.8 Hz, 1H), 3.93 (dd, J = 7.2, 6.0 Hz, 1H), 2.40 (t, J = 7.2 Hz, 2H), 2.01 - 1.86 (m, 4H), 1.66 (s, 9H); ^{13}C NMR (100 MHz, $CDCl_3$) δ 149.6, 141.3, 134.8, 130.6, 128.8, 127.9, 127.5, 127.2, 126.9, 124.5, 119.6, 117.5, 115.2, 107.1, 88.6, 84.8, 84.1, 37.8, 37.4, 28.3, 23.4, 17.1; IR (film): ν (cm^{-1}) 2940, 1730, 1467, 1369, 1232, 1158, 1002, 832,750; HR-MS (ESI) *m/z* calcd for $C_{26}H_{27}N_2O_2$ [M+Na$^+$]: 399.2067; found 399.2065.

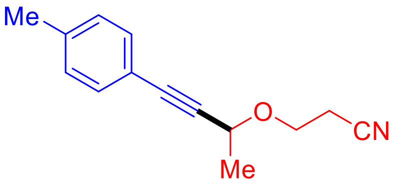

This compound was prepared following general procedure A using oxime ester **1s** (0.2 mmol) as starting material and purified by column chromatography on silica gel.

3-((4-(*p*-Tolyl)but-3-yn-2-yl)oxy)propanenitrile (4an). Yellow oil, 67%, 28.5 mg. ^{1}H NMR (400 MHz, $CDCl_3$) δ 7.33 (d, J = 8.0 Hz, 2H), 7.12 (d, J = 7.6 Hz, 2H), 4.46 (q, J = 6.4 Hz, 1H), 4.04 - 3.98 (m, 1H), 4.73 - 3.67 (m, 1H), 2.66 (t, J = 6.4 Hz, 2H), 2.35 (s, 3H), 1.54 (d, J = 6.4 Hz, 3H); ^{13}C NMR (100 MHz, $CDCl_3$) δ 138.9, 131.8, 129.2, 119.3, 117.9, 87.3, 86.0, 66.6, 63.3, 22.2, 21.6, 19.0; IR (film): ν (cm^{-1}) 2931, 1511, 1440, 1106, 942, 821, 723; HR-MS (ESI) *m/z* calcd for $C_{14}H_{16}NO$ [M+H$^+$]: 214.1226; found 214.1222.

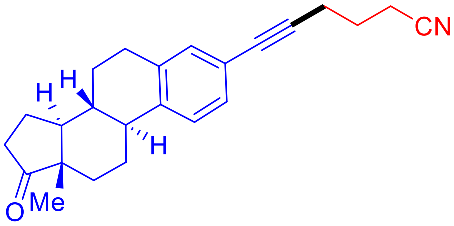

This compound was prepared following general procedure A using oxime ester **1a** (0.2 mmol) and alkyne **6a** (0.4 mmol) as starting materials and purified by column

chromatography on silica gel.

6-((8*R*,9*S*,13*S*,14*S*)-13-Methyl-17-oxo-7,8,9,11,12,13,14,15,16,17-decahydro-6*H*-cyclopenta[a]phenanthren-3-yl)hex-5-ynenitrile (6b). Yellow oil, 72%, 49.7 mg. ^{1}H NMR (400 MHz, $CDCl_3$) δ 7.22 (d, *J* = 8.0 Hz, 1H), 7.17 (d, *J* = 8.0 Hz, 1H), 7.15 (s, 1H), 2.90 - 2.86 (m, 2H), 2.59 (t, *J* = 6.8 Hz, 2H), 2.56 (t, *J* = 6.8 Hz, 2H), 2.54 - 2.48 (m, 1H), 2.43 - 2.38 (m, 1H), 2.32 - 2.26 (m, 1H), 2.19 - 1.92 (m, 6H), 1.68 - 1.38 (m, 6H), 0.91 (s, 3H); ^{13}C NMR (100 MHz, $CDCl_3$) δ 220.8, 140.1, 136.7, 132.2, 129.0, 125.5, 120.6, 119.4, 86.3, 82.5, 50.6, 48.0, 44.5, 38.1, 35.9, 31.6, 29.2, 26.4, 25.7, 24.8, 21.7, 18.7, 16.3, 13.9; IR (film): ν (cm^{-1}) 2930, 2240, 1760, 1484, 1336, 1259, 1051, 903; HR-MS (ESI) *m/z* calcd for $C_{24}H_{28}NO$ [M+H$^+$]: 346.2165; found 346.2172.

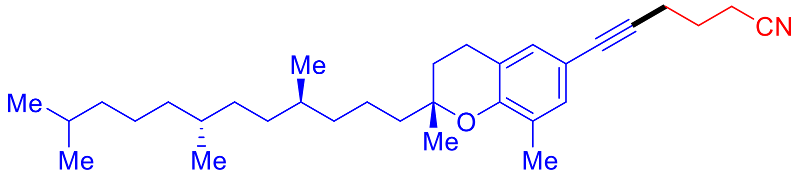

This compound was prepared following general procedure A using oxime ester **1a** (0.2 mmol) and alkyne **7a** (0.4 mmol) as starting materials and purified by column chromatography on silica gel.

6-((*R*)-2,8-Dimethyl-2-((4*R*,8*S*)-4,8,11-trimethyldodecyl)chroman-6-yl)hex-5-ynenitrile (7b). Yellow oil, 70%, 64.8 mg. ^{1}H NMR (400 MHz, $CDCl_3$) δ 7.01 (s, 1H), 6.98 (s, 1H), 2.72 - 2.69 (m, 2H), 2.57 (t, *J* = 6.8 Hz, 2H), 2.55 (t, *J* = 6.8 Hz, 2H), 2.12 (s, 3H), 1.97 - 1.90 (m, 2H), 1.83 - 1.72 (m, 2H), 1.57 - 1.05 (m, 21H), 0.87 (d, *J* = 6.4 Hz, 6H), 0.87 (s, 3H), 0.86 (d, *J* = 6.8 Hz, 3H), 0.85 (d, *J* = 6.8 Hz, 3H); ^{13}C NMR (100 MHz, $CDCl_3$) δ 152.5, 131.6, 130.5, 126.6, 120.7, 119.5, 113.2, 84.3, 83.0, 76.7, 40.2, 39.5, 37.6, 37.5, 37.4, 32.9, 32.8, 31.2, 28.1, 25.0, 24.9, 24.6, 24.4, 22.9, 22.8, 21.2, 21.1, 19.9, 19.8, 18.7, 16.3, 16.0; IR (film): ν (cm^{-1}) 2935, 1467, 1309, 1238, 1167, 942, 882, 732; HR-MS (ESI) *m/z* calcd for $C_{33}H_{51}NO$ [M+H$^+$]: 477.3965; found 477.3985.

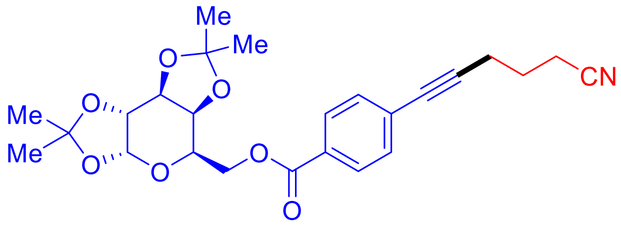

This compound was prepared following general procedure A using oxime ester **1a** (0.2 mmol) and sugar derivative alkyne **8a** (0.4 mmol) as starting materials and purified by column chromatography on silica gel.

3-(Naphthalen-2-yl)-6-(pyridin-3-yl)hex-5-ynenitrile (8b). Yellow oil, 55%, 50.1 mg.

^{1}H NMR (400 MHz, $CDCl_3$) δ 7.98 (d, J = 8.4 Hz, 2H), 7.44 (d, J = 8.0 Hz, 2H), 5.57 (d, J = 4.8 Hz, 1H), 4.65 (dd, J = 7.6, 2.0 Hz, 1H), 4.52 (dd, J = 7.6, 4.8 Hz, 1H), 4.42 (dd, J = 7.6, 4.8 Hz, 1H), 4.35 - 4.33 (m, 1H), 4.32 (d, J = 8.8 Hz, 1H), 4.18 - 4.16 (m, 1H), 2.63 (t, J = 6.8 Hz, 2H), 2.56 (t, J = 7.2 Hz, 2H), 2.01 - 1.94 (m, 2H), 1.51 (s, 3H), 1.47 (s, 3H), 1.35 (s, 3H), 1.33 (s, 3H); ^{13}C NMR (100 MHz, $CDCl_3$) δ 166.0, 131.7, 129.7, 129.4, 128.1, 119.2, 109.8, 109.0, 96.4, 90.4, 82.0, 71.2, 70.8, 70.6, 66.2, 64.2, 26.1, 26.1, 25.1, 24.6, 24.6, 18.7, 16.4; IR (film): ν (cm^{-1}) 2987, 1716, 1378, 1267, 1173, 1002, 902; HR-MS (ESI) *m/z* calcd for $C_{25}H_{29}NNaO_7$ [$M+Na^+$]: 478.1836; found 478.1838.

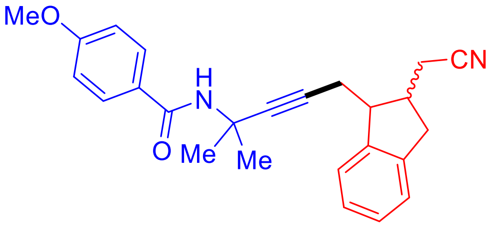

dr 1:1

This compound was prepared following general procedure A using oxime ester **1t** (0.2 mmol) as starting material and purified by column chromatography on silica gel.

***N*-(5-(2-(Cyanomethyl)-2,3-dihydro-1*H*-inden-1-yl)-2-methylpent-3-yn-2-yl)-4-methoxybenzamide (14)**. Yellow oil, 57%, 44.0 mg. ^{1}H NMR (400 MHz, $CDCl_3$) δ 7.70- 7.66 (m, 2H), 7.34 - 7.33 (m, 1H), 7.26- 7.25 (m, 3H), 6.92 - 6.90 (m, 2H), 6.07 (*br* s, 0.5H), 6.04 (*br* s, 0.5H), 3.85 (s, 3H), 3.62 - 3.58 (m, 0.5H), 3.49 - 3.42 (m, 1H), 3.37 - 3.31 (m, 0.5H), 2.81 (dd, J = 16.4, 5.2 Hz, 0.5H), 2.74 - 2.63 (m, 2H), 2.59 - 2.51 (m, 1.5H), 2.43 (dd, J = 16.4, 7.2 Hz, 0.5H), 2.33 - 2.26 (m, 0.5H), 2.19 - 2.12 (m, 0.5H), 1.69 (s, 0.5H), 1.69 (s, 3H), 1.65 (s, 3H); ^{13}C NMR (100 MHz, $CDCl_3$) δ 166.1, 166.1, 162.3, 162.2, 145.6, 145.6, 143.5, 128.8, 128.8, 128.0, 127.8, 127.6, 127.6, 127.5, 127.4, 124.7, 124.1, 123.8, 123.0, 118.9, 118.8, 113.8, 113.8, 85.8, 85.4, 79.6, 79.3, 55.5, 48.6, 48.6, 42.5, 40.0, 39.7, 38.6, 37.9, 29.4, 29.3, 24.8, 23.8, 23.3, 22.6; IR (film): ν (cm^{-1}) 2922, 1643, 1507, 1254, 1177, 1030, 843, 762; HR-MS (ESI) *m/z* calcd for $C_{25}H_{26}N_2NaO_2$ [$M+H^+$]: 409.1886; found 409.1893.

Note: The relative configuration could be determined by analogy with compound **13f** in Waser's publication (see the respective SI file: page S50).[2]

Alkynyl ketones

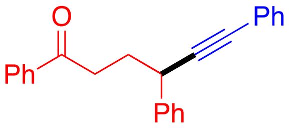

This compound was prepared following general procedure B using oxime ester **2a** (0.1 mmol) as starting material and purified by column chromatography on silica gel (petroleum ether/EtOAc 98:2).

1,4,6-Triphenylhex-5-yn-1-one (5a). Colorless oil, 76%, 24.8 mg. ^{1}H NMR (400 MHz, $CDCl_3$) δ 7.97 - 7.95 (m, 2H), 7.58 - 7.53 (m, 1H), 7.50 - 7.43 (m, 6H), 7.39 - 7.35 (m, 2H), 7.32 - 7.25 (m, 4H), 4.06 (dd, *J* = 8.4, 5.6 Hz, 1H), 3.26 (ddd, *J* = 17.2, 8.8, 6.8 Hz, 1H), 3.15 (ddd, *J* = 17.2, 8.8, 5.6 Hz, 1H), 2.40 - 2.32 (m, 1H), 2.28 - 2.19 (m, 1H); ^{13}C NMR (100 MHz, $CDCl_3$) δ 199.8, 141.5, 137.1, 133.2, 131.8 (2), 128.8 (2), 128.7 (2), 128.4 (2), 128.2 (2), 128.1, 127.7 (2), 127.1, 123.6, 90.8, 84.2, 37.7, 36.2, 32.9; IR (film): ν (cm^{-1}) 2924, 2854, 1732, 1684, 1599, 1491, 1448, 1365, 1230, 1176; HR-MS (ESI) *m/z* calcd for $C_{24}H_{21}O$ [M+H$^+$]: 325.1587; found 325.1585.

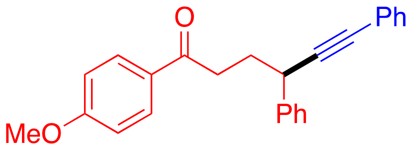

This compound was prepared following general procedure B using oxime ester **2b** (0.1 mmol) as starting material and purified by column chromatography on silica gel (petroleum ether/EtOAc 96:4).

1-(4-Methoxyphenyl)-4,6-diphenylhex-5-yn-1-one (5b). Yellow solid, 78%, 27.8 mg. Mp: 65 - 67 °C; ^{1}H NMR (400 MHz, $CDCl_3$) δ 7.96 - 7.93 (m, 2H), 7.50 - 7.42 (m, 4H), 7.38 - 7.33 (m, 2H), 7.32 -7.25 (m, 4H), 6.94 - 6.90 (m, 2H), 4.04 (dd, *J* = 8.4, 5.6 Hz, 1H), 3.86 (s, 3H), 3.20 (ddd, *J* = 17.6, 8.4, 6.4 Hz, 1H), 3.09 (ddd, *J* = 17.6, 8.8, 5.6 Hz, 1H), 2.38 - 2.30 (m, 1H), 2.26 - 2.17 (m, 1H); ^{13}C NMR (100 MHz, $CDCl_3$) δ 198.4, 163.6, 141.6, 131.8 (2), 130.5 (2), 130.2, 128.7 (2), 128.4 (2), 128.0, 127.7 (2), 127.1, 123.7, 113.8 (2), 90.9, 84.2, 55.6, 37.8, 35.8, 33.1; IR (film): ν (cm^{-1}) 2935, 1667, 1595, 1508, 1454, 1369, 1312, 1244, 1207, 1168, 1114, 1027; HR-MS (ESI) *m/z* calcd for $C_{25}H_{22}NaO_2$ [M+Na$^+$]: 377.1512; found 377.1514.

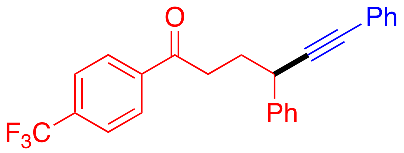

This compound was prepared following general procedure B using oxime ester **2c** (0.1 mmol) as starting material and purified by column chromatography on silica gel (petroleum ether/EtOAc 97:3).

4,6-Diphenyl-1-(4-(trifluoromethyl)phenyl)hex-5-yn-1-one (5c). Colorless oil, 78%, 30.4 mg. ^{1}H NMR (400 MHz, $CDCl_3$) δ 8.05 (d, *J* = 8.4 Hz, 2H), 7.71 (d, *J* = 8.0 Hz, 2H), 7.50 - 7.47 (m, 2H), 7.44 - 7.41 (m, 2H), 7.39 - 7.35 (m, 2H), 7.32 - 7.28 (m, 4H),

4.06 (dd, J = 8.4, 5.2 Hz, 1H), 3.27 (ddd, J = 17.2, 8.4, 6.4 Hz, 1H), 3.16 (ddd, J = 17.2, 8.8, 5.6 Hz, 1H), 2.41 - 2.33 (m, 1H), 2.29 - 2.20 (m, 1H); ^{13}C NMR (100 MHz, $CDCl_3$) δ 198.8, 141.3, 139.7, 134.5 (q, J = 32.4 Hz), 131.8 (2), 128.8 (2), 128.5 (2), 128.4 (2), 128.2, 127.6 (2), 127.2, 125.8 (q, J = 3.7 Hz) (2), 123.7 (q, J = 271.1 Hz), 123.5, 90.5, 84.5, 37.6, 36.4, 32.6; IR (film): ν (cm^{-1}) 3062, 2160, 1689, 1599, 1491, 1450, 1410, 1323, 1228, 1167, 1128, 1066, 1014; HR-MS (ESI) *m/z* calcd for $C_{25}H_{20}F_3O$ [M+H$^+$]: 393.1461; found 393.1460.

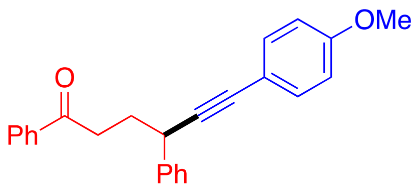

This compound was prepared following general procedure B using oxime ester **2a** (0.1 mmol) as starting material and purified by column chromatography on silica gel (petroleum ether/EtOAc 97:3).

6-(4-Methoxyphenyl)-1,4-diphenylhex-5-yn-1-one (5d). Light yellow oil, 62%, 22.0 mg. ^{1}H NMR (400 MHz, $CDCl_3$) δ 7.97 - 7.95 (m, 2H), 7.57 - 7.53 (m, 1H), 7.49 - 7.43 (m, 4H), 7.40 - 7.34 (m, 4H), 7.28 - 7.24 (m, 1H), 6.85 - 6.81 (m, 2H), 4.03 (dd, J = 8.4, 5.2 Hz, 1H), 3.81 (s, 3H), 3.25 (ddd, J = 17.2, 8.8, 6.8 Hz, 1H), 3.14 (ddd, J = 17.2, 8.8, 5.6 Hz, 1H), 2.38 - 2.29 (m, 1H), 2.26 - 2.17 (m, 1H); ^{13}C NMR (100 MHz, $CDCl_3$) δ 199.9, 159.4, 141.7, 137.1, 133.2 (3), 128.7 (4), 128.2 (2), 127.7 (2), 127.0, 115.8, 114.0 (2), 89.2, 84.0, 55.4, 37.7, 36.2, 32.9; IR (film): ν (cm^{-1}) 2931, 1682, 1601, 1508, 1448, 1363, 1288, 1246, 1173, 1105, 1028; HR-MS (ESI) *m/z* calcd for $C_{25}H_{22}AgO_2$ [M+Ag$^+$]: 461.0665; found 461.0676.

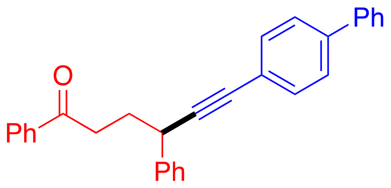

This compound was prepared following general procedure B using oxime ester **2a** (0.1 mmol) as starting material and purified by column chromatography on silica gel (petroleum ether/EtOAc 98:2).

6-([1,1'-Biphenyl]-4-yl)-1,4-diphenylhex-5-yn-1-one (5e). Yellow solid, 58%, 23.4 mg. Mp: 105 - 107 °C. ^{1}H NMR (400 MHz, $CDCl_3$) δ 7.98 - 7.96 (m, 2H), 7.60 - 7.49 (m, 9H), 7.48 - 7.43 (m, 4H), 7.39 - 7.34 (m, 3H), 7.30 - 7.26 (m, 1H), 4.08 (dd, J = 7.6, 5.6 Hz, 1H), 3.28 (ddd, J = 17.2, 8.8, 6.8 Hz, 1H), 3.16 (ddd, J = 17.2, 8.8, 5.6 Hz, 1H), 2.42 - 2.33 (m, 1H), 2.30 - 2.21 (m, 1H); ^{13}C NMR (100 MHz, $CDCl_3$) δ 199.8, 141.5, 140.8, 140.5, 137.1, 133.2, 132.2 (2), 129.0 (2), 128.8 (2), 128.7 (2), 128.2 (2), 127.7,

127.7 (2), 127.1 (3), 127.1 (2), 122.5, 91.5, 84.1, 37.8, 36.2, 32.9; IR (film): ν (cm^{-1}) 2951, 1678, 1597, 1487, 1448, 1367, 1323, 1263, 1205, 1003; HR-MS (ESI) *m/z* calcd for $C_{30}H_{24}NaO$ [M+Na$^+$]: 423.1719; found 423.1713.

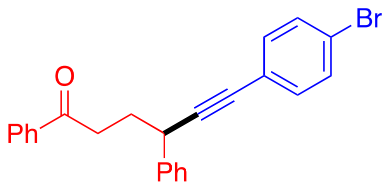

This compound was prepared following general procedure B using oxime ester **2a** (0.1 mmol) as starting material and purified by column chromatography on silica gel (petroleum ether/EtOAc 98:2).

6-(4-Bromophenyl)-1,4-diphenylhex-5-yn-1-one (5f). Colorless oil, 76%, 30.7 mg. ^{1}H NMR (400 MHz, CD_3OD) δ 7.97 - 7.94 (m, 2H), 7.61 - 7.57 (m, 1H), 7.49 - 7.44 (m, 6H), 7.37 - 7.34 (m, 2H), 7.31 - 7.24 (m, 3H), 4.04 (dd, *J* = 8.4, 6.0 Hz, 1H), 3.24 (ddd, *J* = 17.2, 7.6, 6.8 Hz, 1H), 3.16 (ddd, *J* = 17.2, 8.0, 6.4 Hz, 1H), 2.30 - 2.15 (m, 2H); ^{13}C NMR (100 MHz, CD_3OD) δ 201.7, 142.7, 138.2, 134.3, 134.2 (2), 132.7 (2), 129.8 (2), 129.7 (2), 129.1 (2), 128.5 (2), 128.1, 123.9, 123.0, 93.2, 83.9, 38.7, 37.0, 34.0; IR (film): ν (cm^{-1}) 3060, 3030, 1682, 1597, 1487, 1450, 1363, 1228, 1201, 1070, 1009; HR-MS (ESI) *m/z* calcd for $C_{24}H_{19}{}^{79}BrNaO$ [M+Na$^+$]: 425.0511; found 425.0510.

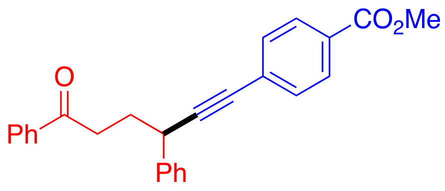

This compound was prepared following general procedure B using oxime ester **2a** (0.1 mmol) as starting material and purified by column chromatography on silica gel (petroleum ether/EtOAc 96:4).

Methyl 4-(6-oxo-3,6-diphenylhex-1-yn-1-yl)benzoate (5g). Yellow semisolid, 48%, 18.3 mg. ^{1}H NMR (400 MHz, $CDCl_3$) δ 7.98 - 7.94 (m, 4H), 7.58 - 7.54 (m, 1H), 7.49 - 7.43 (m, 6H), 7.39 - 7.35 (m, 2H), 7.30 - 7.26 (m, 1H), 4.07 (dd, *J* = 8.4, 6.0 Hz, 1H), 3.92 (s, 3H), 3.24 (ddd, *J* = 17.2, 8.4, 6.8 Hz, 1H), 3.13 (ddd, *J* = 17.6, 8.0, 5.6 Hz, 1H), 2.40 - 2.21 (m, 2H); ^{13}C NMR (100 MHz, $CDCl_3$) δ 199.7, 166.7, 141.1, 137.0, 133.3, 131.7 (2), 129.6 (2), 129.4, 128.9 (2), 128.8 (2), 128.4, 128.2 (2), 127.6 (2), 127.3, 94.2, 83.6, 52.4, 37.8, 36.1, 32.6; IR (film): ν (cm^{-1}) 2927, 1720, 1682, 1603, 1448, 1435, 1275, 1176, 1107; HR-MS (ESI) *m/z* calcd for $C_{26}H_{22}NaO_3$ [M+Na$^+$]: 405.1461; found 405.1457.

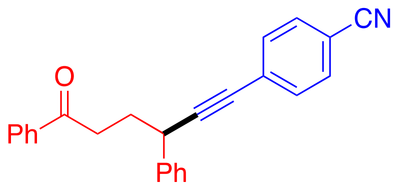

This compound was prepared following general procedure B using oxime ester **2a** (0.1 mmol) as starting material and purified by column chromatography on silica gel (petroleum ether/EtOAc 96:4).

4-(6-Oxo-3,6-diphenylhex-1-yn-1-yl)benzonitrile (5h). Yellow oil, 63%, 22.0 mg. ^{1}H NMR (400 MHz, $CDCl_3$) δ 7.95 - 7.93 (m, 2H), 7.59 - 7.54 (m, 3H), 7.50 - 7.43 (m, 6H), 7.39 - 7.35 (m, 2H), 7.31 - 7.27 (m, 1H), 4.07 (dd, J = 8.0, 6.0 Hz, 1H), 3.21 (ddd, J = 17.2, 8.0, 6.8 Hz, 1H), 3.11 (ddd, J = 17.2, 8.0, 6.0 Hz, 1H), 2.40 - 2.24 (m, 2H); ^{13}C NMR (100 MHz, $CDCl_3$) δ 199.5, 140.7, 137.0, 133.3, 132.4 (2), 132.1 (2), 128.9 (2), 128.8 (2), 128.6, 128.1 (2), 127.6 (2), 127.4, 118.7, 111.4, 95.9, 82.7, 37.8, 36.0, 32.5; IR (film): ν (cm^{-1}) 2927, 2225, 2158, 1682, 1601, 1581, 1496, 1450, 1404, 1363, 1228, 1201, 1178; HR-MS (ESI) *m/z* calcd for $C_{25}H_{19}AgNO$ [M+Ag^{+}]: 456.0512; found 456.0508.

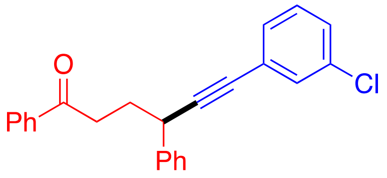

This compound was prepared following general procedure B using oxime ester **2a** (0.1 mmol) as starting material and purified by column chromatography on silica gel (petroleum ether/EtOAc 98:2).

6-(3-Chlorophenyl)-1,4-diphenylhex-5-yn-1-one (5i). Yellow oil, 67%, 24 mg. ^{1}H NMR (400 MHz, $CDCl_3$) δ 7.97 - 7.95 (m, 2H), 7.58 - 7.54 (m, 1H), 7.47 - 7.41 (m, 5H), 7.39 - 7.35 (m, 2H), 7.32 - 7.20 (m, 4H), 4.05 (dd, J = 8.4, 5.6 Hz, 1H), 3.23 (ddd, J = 17.2, 8.4, 6.4 Hz, 1H), 3.13 (ddd, J = 17.2, 8.4, 5.6 Hz, 1H), 2.40 - 2.31 (m, 1H), 2.29 - 2.20 (m, 1H); ^{13}C NMR (100 MHz, $CDCl_3$) δ 199.7, 141.1, 137.0, 134.2, 133.2, 131.7, 129.9, 129.6, 128.8 (2), 128.7 (2), 128.4, 128.2 (2), 127.6 (2), 127.2, 125.3, 92.2, 82.9, 37.7, 36.1, 32.7; IR (film): ν (cm^{-1}) 2925, 1682, 1595, 1562, 1493, 1473, 1448, 1408, 1363, 1228, 1203, 1178, 1095, 1076; HR-MS (ESI) *m/z* calcd for $C_{24}H_{19}AgClO$ [M+Ag^{+}]: 465.0170; found 465.0168.

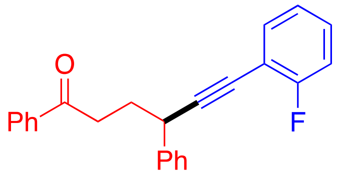

This compound was prepared following general procedure B using oxime ester **2a** (0.1 mmol) as starting material and purified by column chromatography on silica gel (petroleum ether/EtOAc 98:2).

6-(2-Fluorophenyl)-1,4-diphenylhex-5-yn-1-one (5j). Yellow oil, 67%, 23.1 mg. ^{1}H NMR (400 MHz, $CDCl_3$) δ 8.00 - 7.97 (m, 2H), 7.58 - 7.53 (m, 1H), 7.52 - 7.41 (m, 5H), 7.39 - 7.35 (m, 2H), 7.31 - 7.26 (m, 2H), 7.10 - 7.06 (m, 2H), 4.11 (dd, *J* = 8.8, 5.6 Hz, 1H), 3.31 (ddd, *J* = 17.2, 8.8, 6.4 Hz, 1H), 3.18 (ddd, *J* = 17.2, 8.8, 5.2 Hz, 1H), 2.42 - 2.34 (m, 1H), 2.28 - 2.18 (m, 1H); ^{13}C NMR (100 MHz, $CDCl_3$) δ 199.9, 163.1 (d, *J* = 249.2 Hz), 141.1, 137.1, 133.6 (d, *J* = 1.4 Hz), 133.2, 129.7 (d, *J* = 7.9 Hz), 128.8 (2), 128.7 (2), 128.2 (2), 127.6 (2), 127.1, 124.0 (d, *J* = 3.6 Hz), 115.6 (d, *J* = 20.9 Hz), 112.1 (d, *J* = 15.7 Hz), 96.2 (d, *J* = 3.4 Hz), 77.7, 37.9, 36.1, 32.8; IR (film): ν (cm^{-1}) 2931, 1682, 1599, 1579, 1491, 1448, 1365, 1250, 1215, 1103, 1078; HR-MS (ESI) *m/z* calcd for $C_{24}H_{19}AgFO$ [$M+Ag^{+}$]: 449.0465; found 449.0473.

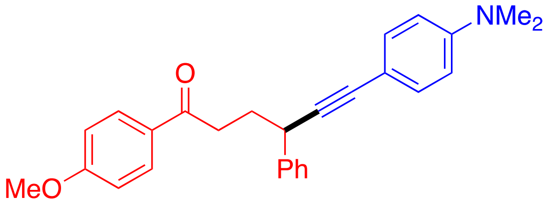

This compound was prepared following general procedure B using oxime ester **2b** (0.1 mmol) as starting material and purified by column chromatography on silica gel (petroleum ether/EtOAc 9:1).

6-(4-(Dimethylamino)phenyl)-1-(4-methoxyphenyl)-4-phenylhex-5-yn-1-one (5k). Dark yellow oil, 58%, 23 mg. ^{1}H NMR (400 MHz, $CDCl_3$) δ 7.96 - 7.92 (m, 2H), 7.50 - 7.48 (m, 2H), 7.36 - 7.31 (m, 4H), 7.26 - 7.23 (m, 1H), 6.93 - 6.89 (m, 2H), 6.64 - 6.62 (m, 2H), 4.02 (dd, *J* = 8.8, 5.2 Hz, 1H), 3.86 (s, 3H), 3.21 (ddd, *J* = 17.2, 9.2, 6.8 Hz, 1H), 3.13 - 3.06 (m, 1H), 2.96 (s, 6H), 2.36 - 2.27 (m, 1H), 2.22 - 2.13 (m, 1H); ^{13}C NMR (100 MHz, $CDCl_3$) δ 198.7, 163.5, 150.1, 142.1, 132.8 (2), 130.5 (2), 130.3, 128.6 (2), 127.7 (2), 126.9, 113.8 (2), 112.0 (2), 110.7, 88.2, 84.9, 55.6, 40.5 (2), 37.9, 36.0, 33.4; IR (film): ν (cm^{-1}) 2931, 1673, 1599, 1576, 1520, 1511, 1451, 1361, 1309, 1256, 1238, 1169, 1028; HR-MS (ESI) *m/z* calcd for $C_{27}H_{28}NO_2$ [$M+H^{+}$]: 398.2115; found 398.2139.

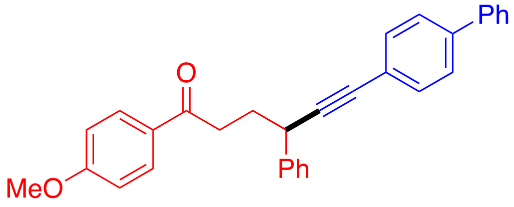

This compound was prepared following general procedure B using oxime ester **2b** (0.1 mmol) as starting material and purified by column chromatography on silica gel (petroleum ether/EtOAc 95:5).

6-([1,1'-Biphenyl]-4-yl)-1-(4-methoxyphenyl)-4-phenylhex-5-yn-1-one (5l). Yellow solid, 71%, 30.7 mg. Mp: 133 - 135 °C; ^{1}H NMR (400 MHz, $CDCl_3$) δ 7.97 - 7.94 (m, 2H), 7.60 - 7.57 (m, 2H), 7.56 - 7.49 (m, 6H), 7.46 - 7.43 (m, 2H), 7.39 - 7.33 (m, 3H), 7.30 - 7.26 (m, 1H), 6.94 - 6.91 (m, 2H), 4.06 (dd, *J* = 8.4, 5.6 Hz, 1H), 3.86 (s, 3H), 3.22 (ddd, *J* = 17.2, 8.4, 6.4 Hz, 1H), 3.11 (ddd, *J* = 17.2, 8.8, 5.6 Hz, 1H), 2.40 - 2.31 (m, 1H), 2.28 - 2.19 (m, 1H); ^{13}C NMR (100 MHz, $CDCl_3$) δ 198.4, 163.6, 141.6, 140.8, 140.6, 132.2 (2), 130.5 (2), 130.2, 129.0 (2), 128.8 (2), 127.7 (3), 127.1 (2), 127.1 (3), 122.6, 113.9 (2), 91.6, 84.0, 55.6, 37.9, 35.9, 33.1; IR (film): ν (cm^{-1}) 2960, 1672, 1597, 1508, 1486, 1368, 1310, 1258, 1246, 1205, 1167, 1113, 1027; HR-MS (ESI) *m/z* calcd for $C_{31}H_{26}NaO_2$ [M+Na$^+$]: 453.1825; found 453.1826.

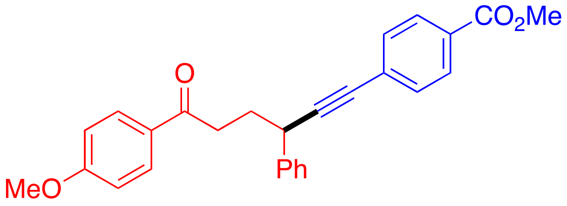

This compound was prepared following general procedure B using oxime ester **2b** (0.1 mmol) as starting material and purified by column chromatography on silica gel (petroleum ether/EtOAc 98:2).

Methyl 4-(6-(4-methoxyphenyl)-6-oxo-3-phenylhex-1-yn-1-yl)benzoate (5m). Yellow solid, 76%, 31.5 mg. Mp: 91 - 93 °C; ^{1}H NMR (400 MHz, $CDCl_3$) δ 7.98 - 7.95 (m, 2H), 7.94 - 7.92 (m, 2H), 7.49 - 7.45 (m, 4H), 7.38 - 7.35 (m, 2H), 7.30 - 7.26 (m, 1H), 6.94 - 6.90 (m, 2H), 4.06 (dd, *J* = 8.4, 5.6 Hz, 1H), 3.92 (s, 3H), 3.86 (s, 3H), 3.18 (ddd, *J* = 16.8, 8.4, 6.4 Hz, 1H), 3.08 (ddd, *J* = 16.8, 8.4, 5.6 Hz, 1H), 2.39 - 2.30 (m, 1H), 2.29 - 2.20 (m, 1H); ^{13}C NMR (100 MHz, $CDCl_3$) δ 198.2, 166.7, 163.6, 141.1, 131.7 (2), 130.4 (2), 130.1, 129.6 (2), 129.4, 128.8 (2), 128.4, 127.6 (2), 127.2, 113.9 (2), 94.3, 83.5, 55.6, 52.4, 37.8, 35.7, 32.8; IR (film): ν (cm^{-1}) 2953, 1724, 1667, 1595, 1508, 1453, 1434, 1370, 1310, 1272, 1257, 1243, 1208, 1168, 1108; HR-MS (ESI) *m/z* calcd for $C_{27}H_{24}AgO_4$ [M+Ag$^+$]: 519.0720; found 519.0708.

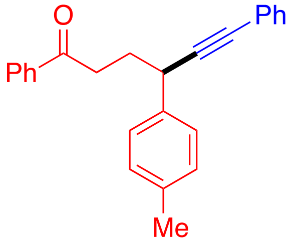

This compound was prepared following general procedure B using oxime ester **2d** (0.1 mmol) as starting material and purified by column chromatography on silica gel (petroleum ether/EtOAc 98:2).

1,6-Diphenyl-4-(*p*-tolyl)hex-5-yn-1-one (5n). Yellow oil, 60%, 20.3 mg. ^{1}H NMR (400 MHz, $CDCl_3$) δ 7.98 - 7.95 (m, 2H), 7.57 - 7.53 (m, 1H), 7.47 - 7.41 (m, 4H), 7.38 - 7.36 (m, 2H), 7.33 - 7.28 (m, 3H), 7.18 - 7.16 (m, 2H), 4.01 (dd, J = 8.8, 6.0 Hz, 1H), 3.24 (ddd, J = 17.6, 8.8, 6.8 Hz, 1H), 3.14 (ddd, J = 17.2, 8.8, 5.6 Hz, 1H), 2.38 - 2.29 (m, 1H), 2.35 (s, 3H), 2.26 - 2.17 (m, 1H); ^{13}C NMR (100 MHz, $CDCl_3$) δ 199.9, 138.5, 137.1, 136.7, 133.2, 131.8 (2), 129.4 (2), 128.7 (2), 128.4 (2), 128.2 (2), 128.0, 127.5 (2), 123.7, 91.0, 84.0, 37.3, 36.2, 32.9, 21.2; IR (film): ν (cm^{-1}) 2924, 1682, 1597, 1579, 1512, 1491, 1446, 1363, 1228, 1201, 1178; HR-MS (ESI) *m/z* calcd for $C_{25}H_{22}NaO$ [M+Na$^+$]: 361.1563; found 361.1556.

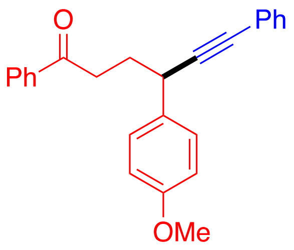

This compound was prepared following general procedure B using oxime ester **2e** (0.1 mmol) as starting material and purified by column chromatography on silica gel (petroleum ether/EtOAc 97:3).

4-(4-Methoxyphenyl)-1,6-diphenylhex-5-yn-1-one (5o). Pale yellow oil, 68%, 24.0 mg. ^{1}H NMR (400 MHz, $CDCl_3$) δ 7.98 - 7.95 (m, 2H), 7.57 - 7.53 (m, 1H), 7.47 - 7.38 (m, 6H), 7.32 - 7.28 (m, 3H), 6.92 - 6.88 (m, 2H), 4.00 (dd, J = 8.4, 5.6 Hz, 1H), 3.81 (s, 3H), 3.24 (ddd, J = 17.2, 8.8, 6.4 Hz, 1H), 3.13 (ddd, J = 17.2, 8.4, 5.2 Hz, 1H), 2.36 - 2.28 (m, 1H), 2.26 - 2.16 (m, 1H); ^{13}C NMR (100 MHz, $CDCl_3$) δ 199.9, 158.7, 137.1, 133.6, 133.2, 131.8 (2), 128.7 (2), 128.6 (2), 128.4 (2), 128.2 (2), 128.0, 123.7, 114.1 (2), 91.1, 84.0, 55.5, 36.9, 36.2, 32.9; IR (film): ν (cm^{-1}) 2931, 1682, 1599, 1510, 1446, 1363, 1302, 1248, 1176, 1032; HR-MS (ESI) *m/z* calcd for $C_{25}H_{23}O_2$ [M+H$^+$]: 355.1693; found 355.1682.

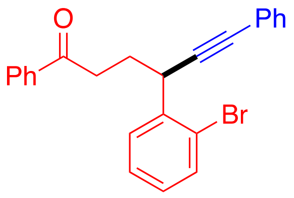

This compound was prepared following general procedure B using oxime ester **2f** (0.1 mmol) as starting material and purified by column chromatography on silica gel (petroleum ether/toluene 1:1).

4-(2-Bromophenyl)-1,6-diphenylhex-5-yn-1-one (5p). Light yellow oil, 58%, 23.3 mg. ^{1}H NMR (400 MHz, $CDCl_3$) δ 7.99 - 7.97 (m, 2H), 7.76 (dd, *J* = 7.6, 1.6 Hz, 1H), 7.58 - 7.54 (m, 2H), 7.47 - 7.44 (m, 4H), 7.37 - 7.30 (m, 4H), 7.14 (td, *J* = 7.6, 1.2 Hz, 1H), 4.49 (dd, *J* = 8.8, 5.2 Hz, 1H), 3.26 (t, *J* = 8.0 Hz, 2H), 2.39 - 2.31 (m, 1H), 2.24 - 2.15 (m, 1H); ^{13}C NMR (100 MHz, $CDCl_3$) δ 199.6, 140.7, 137.0, 133.2, 133.1, 131.8 (2), 129.6, 128.8, 128.7 (2), 128.4 (2), 128.2 (3), 128.0, 123.5, 123.4, 90.1, 84.4, 37.5, 36.5, 31.4; IR (film): ν (cm^{-1}) 3059, 2933, 1684, 1597, 1489, 1469, 1442, 1365, 1230, 1201, 1024; HR-MS (ESI) *m/z* calcd for $C_{24}H_{19}{}^{79}BrNaO$ [M+Na^{+}]: 425.0511; found 425.0513.

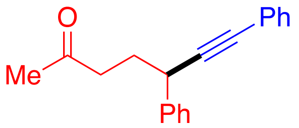

This compound was prepared following general procedure B using oxime ester **2g** (0.1 mmol) as starting material and purified by column chromatography on silica gel (petroleum ether/EtOAc 96:4).

5,7-Diphenylhept-6-yn-2-one (5q). Colorless oil, 62%, 16.3 mg. ^{1}H NMR (400 MHz, $CDCl_3$) δ 7.46 - 7.42 (m, 4H), 7.37 - 7.24 (m, 6H), 3.94 (dd, *J* = 8.4, 5.6 Hz, 1H), 2.70 (ddd, *J* = 17.6, 8.4, 6.4 Hz, 1H), 2.60 (ddd, *J* = 17.6, 8.8, 5.6 Hz, 1H), 2.21 - 2.13 (m, 1H), 2.15 (s, 3H), 2.10 - 1.99 (m, 1H); ^{13}C NMR (100 MHz, $CDCl_3$) δ 208.4, 141.4, 131.8 (2), 128.7 (2), 128.4 (2), 128.1, 127.6 (2), 127.1, 123.6, 90.6, 84.1, 41.2, 37.5, 32.2, 30.3; IR (film): ν (cm^{-1}) 2968, 2925, 1674, 1597, 1577, 1491, 1446, 1375, 1358, 1294, 1223, 1205; HR-MS (ESI) *m/z* calcd for $C_{25}H_{22}AgO$ [M+Ag^{+}]: 445.0716; found 445.0721.

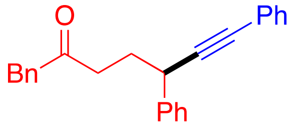

This compound was prepared following general procedure B using oxime ester **2h** (0.1 mmol) as starting material and purified by column chromatography on silica gel (petroleum ether/EtOAc 97:3).

1,5,7-Triphenylhept-6-yn-2-one (5r). Colorless oil, 43%, 14.4 mg. ^{1}H NMR (400 MHz, CD_3OD) δ 7.37 - 7.17 (m, 15H), 3.87 (dd, *J* = 8.8, 6.0 Hz, 1H), 3.73 (s, 2H), 2.76 (m, 1H), 2.67 (ddd, *J* = 18.0, 8.0, 6.0 Hz, 1H), 2.11 - 1.93 (m, 2H); ^{13}C NMR (100 MHz, CD_3OD) δ 210.4, 142.8, 135.9, 132.6 (2), 130.6 (2), 129.7 (2), 129.6 (2), 129.4 (2), 129.0, 128.5 (2), 127.9, 127.9, 124.8, 91.5, 84.9, 50.8, 40.3, 38.4, 33.4; IR (film): ν (cm^{-1}) 2927, 1712, 1689, 1599, 1493, 1450, 1363, 1317, 1070, 1026; HR-MS (ESI) *m/z* calcd for $C_{25}H_{22}NaO$ [M+Na^{+}]: 361.1563; found 361.1563.

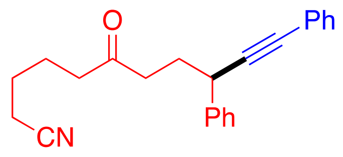

This compound was prepared following general procedure B using oxime ester **2i** (0.1 mmol) as starting material and purified by column chromatography on silica gel (petroleum ether/acetone 9:1).

6-Oxo-9,11-diphenylundec-10-ynenitrile (5s). Colorless oil, 68%, 24.0 mg. ^{1}H NMR (400 MHz, $CDCl_3$) δ 7.46 - 7.42 (m, 4H), 7.37 - 7.29 (m, 5H), 7.28 - 7.25 (m, 1H), 3.94 (dd, J = 8.4, 5.6 Hz, 1H), 2.68 (ddd, J = 17.6, 8.4, 6.8 Hz, 1H), 2.57 (ddd, J = 17.6, 8.4, 5.6 Hz, 1H), 2.47 (t, J = 6.8 Hz, 2H), 2.32 (t, J = 7.2 Hz, 2H), 2.22 - 2.13 (m, 1H), 2.10 - 2.01 (m, 1H), 1.75 - 1.59 (m, 4H); ^{13}C NMR (100 MHz, $CDCl_3$) δ 209.3, 141.3, 131.8 (2), 128.8 (2), 128.4 (2), 128.2, 127.6 (2), 127.1, 123.5, 119.6, 90.5, 84.2, 41.9, 40.3, 37.5, 32.1, 25.1, 22.8, 17.3; IR (film): ν (cm^{-1}) 2931, 1711, 1491, 1450, 1367, 1103, 1026; HR-MS (ESI) *m/z* calcd for $C_{23}H_{23}NNaO$ [M+Na^{+}]: 352.1672; found 352.1663.

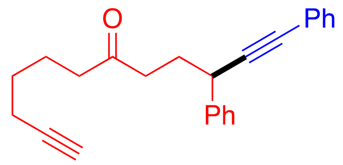

This compound was prepared following general procedure B using oxime ester **2j** (0.1 mmol) as starting material and purified by column chromatography on silica gel (petroleum ether/EtOAc 97:3).

1,3-Diphenyldodeca-1,11-diyn-6-one (5t). Light yellow oil, 50%, 16.3 mg. ^{1}H NMR (400 MHz, $CDCl_3$) δ 7.46 - 7.42 (m, 4H), 7.36 - 7.27 (m, 5H), 7.28 - 7.24 (m, 1H), 3.93 (dd, J = 8.8, 5.6 Hz, 1H), 2.68 (ddd, J = 17.6, 8.4, 6.8 Hz, 1H), 2.57 (ddd, J = 17.6, 8.4, 5.6 Hz, 1H), 2.45 - 2.42 (m, 2H), 2.21 - 2.13 (m, 3H), 2.09 - 2.00 (m, 1H), 1.92 (t, J = 2.8 Hz, 1H), 1.72 - 1.65 (m, 2H), 1.54 - 1.47 (m, 2H); ^{13}C NMR (100 MHz, $CDCl_3$) δ 210.2, 141.4, 131.8 (2), 128.7 (2), 128.4 (2), 128.1, 127.6 (2), 127.1, 123.6, 90.7, 84.2, 84.1, 68.7, 42.5, 40.2, 37.6, 32.2, 28.0, 23.0, 18.4; IR (film): ν (cm^{-1}) 3298, 2933, 2158, 1711, 1491, 1450, 1367, 1101; HR-MS (ESI) *m/z* calcd for $C_{24}H_{24}NaO$ [M+Na^{+}]: 351.1719; found 351.1713.

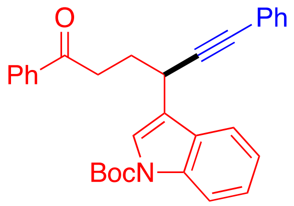

This compound was prepared following general procedure B using oxime ester **2k** (0.1 mmol) as starting material and purified by column chromatography on silica gel (toluene/petroleum ether 95:5).

***tert*-Butyl 3-(6-oxo-1,6-diphenylhex-1-yn-3-yl)-1*H*-indole-1-carboxylate (5u)**. Dark yellow oil, 73%, 33.7 mg. ^{1}H NMR (400 MHz, $CDCl_3$) δ 8.15 (*br* d, *J* = 7.2 Hz, 1H), 7.99 - 7.97 (m, 2H), 7.82 (d, *J* = 7.6 Hz, 1H), 7.66 (*br* s, 1H), 7.58 - 7.54 (m, 1H), 7.47 - 7.41 (m, 4H), 7.36 - 7.28 (m, 5H), 4.28 (dd, *J* = 8.8, 5.2 Hz, 1H), 3.37 (dt, *J* = 17.6, 7.6 Hz, 1H), 3.21 (ddd, *J* = 17.6, 8.0, 5.2 Hz, 1H), 2.60 - 2.51 (m, 1H), 2.38 - 2.29 (m, 1H), 1.68 (s, 9H); ^{13}C NMR (100 MHz, $CDCl_3$) δ 199.8, 149.9, 137.1, 136.0, 133.2, 131.8 (2), 129.1, 128.7 (2), 128.4 (2), 128.2 (2), 128.1, 124.6, 123.5, 123.3, 122.7, 120.8, 119.8, 115.5, 90.0, 83.8, 83.3, 36.1, 30.1, 29.1, 28.4 (3); IR (film): ν (cm^{-1}) 2976, 1730, 1682, 1452, 1367, 1254, 1153, 1086; HR-MS (ESI) *m/z* calcd for $C_{31}H_{29}NNaO_3$ [M+Na$^+$]: 486.2040; found 486.2042.

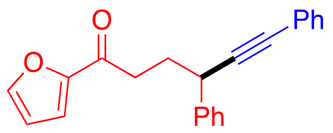

This compound was prepared following general procedure B using oxime ester **2l** (0.1 mmol) as starting material and purified by column chromatography on silica gel (petroleum ether/EtOAc 97:3).

1-(Furan-2-yl)-4,6-diphenylhex-5-yn-1-one (5v). Colorless oil, 76%, 24.0 mg. ^{1}H NMR (400 MHz, $CDCl_3$) δ 7.57 - 7.56 (m, 1H), 7.48 - 7.43 (m, 4H), 7.37 - 7.34 (m, 2H), 7.32 - 7.25 (m, 4H), 7.17 (dd, *J* = 3.6, 0.8 Hz, 1H), 6.51 (dd, *J* = 3.6, 1.6 Hz, 1H), 4.02 (dd, *J* = 8.4, 5.6 Hz, 1H), 3.11 (ddd, *J* = 16.4, 8.4, 6.4 Hz, 1H), 3.00 (ddd, *J* = 16.8, 8.8, 5.6 Hz, 1H), 2.37 - 2.28 (m, 1H), 2.25 - 2.16 (m, 1H); ^{13}C NMR (100 MHz, $CDCl_3$) δ 189.0, 152.8, 146.4, 141.4, 131.8 (2), 128.7 (2), 128.4 (2), 128.1, 127.7 (2), 127.1, 123.6, 117.2, 112.3, 90.6, 84.3, 37.7, 36.1, 32.6; IR (film): ν (cm^{-1}) 2933, 2023, 1674, 1568, 1491, 1468, 1394, 1255, 1159, 1024; HR-MS (ESI) *m/z* calcd for $C_{22}H_{19}O_2$ [M+H$^+$]: 315.1380; found 315.1369.

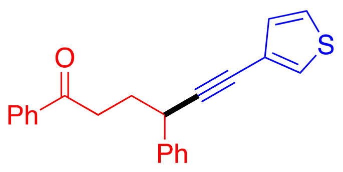

This compound was prepared following general procedure B using oxime ester **2a** (0.1 mmol) as starting material and purified by column chromatography on silica gel (petroleum ether/EtOAc 98:2).

1,4-Diphenyl-6-(thiophen-3-yl)hex-5-yn-1-one (5w). Yellow solid, 70%, 23.0 mg.

Mp: 96 - 98 °C; ^{1}H NMR (400 MHz, $CDCl_3$) δ 7.97 - 7.95 (m, 2H), 7.58 - 7.53 (m, 1H), 7.48 - 7.43 (m, 4H), 7.40 (dd, J = 2.8, 0.8 Hz, 1H), 7.38 - 7.34 (m, 2H), 7.29 - 7.24 (m, 2H), 7.11 (dd, J = 5.2, 1.2 Hz, 1H), 4.03 (dd, J = 8.8, 5.6 Hz, 1H), 3.24 (ddd, J = 17.2, 8.8, 6.4 Hz, 1H), 3.13 (ddd, J = 17.2, 8.8, 5.6 Hz, 1H), 2.34 - 2.30 (m, 1H), 2.27 - 2.18 (m, 1H); ^{13}C NMR (100 MHz, $CDCl_3$) δ 199.8, 141.4, 137.1, 133.2, 130.2, 128.8 (2), 128.7 (2), 128.3, 128.2 (2), 127.6 (2), 127.1, 125.3, 122.6, 90.3, 79.2, 37.7, 36.2, 32.8; IR (film): ν (cm^{-1}) 2954, 16076, 1595, 1491, 1448, 1365, 1263, 1205, 1184, 1074, 1005; HR-MS (ESI) *m/z* calcd for $C_{22}H_{18}NaOS_3$ [M+Na$^+$]: 353.0971; found 353.0968.

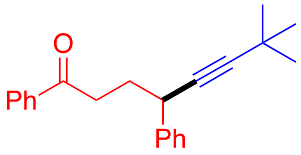

This compound was prepared following general procedure B using oxime ester **2a** (0.1 mmol) as starting material and purified by column chromatography on silica gel (petroleum ether/EtOAc 99:1).

7,7-Dimethyl-1,4-diphenyloct-5-yn-1-one (5x). Colorless oil, 59%, 18.1 mg. ^{1}H NMR (400 MHz, $CDCl_3$) δ 7.96 - 7.94 (m, 2H), 7.58 - 7.54 (m, 1H), 7.48 - 7.40 (m, 4H), 7.35 - 7.31 (m, 2H), 7.25 - 7.22 (m, 1H), 3.79 (dd, J = 8.8, 5.6 Hz, 1H), 3.18 (ddd, J = 16.8, 9.2, 6.4 Hz, 1H), 3.06 (ddd, J = 16.8, 9.2, 5.2 Hz, 1H), 2.27 - 2.18 (m, 1H), 2.08 - 1.99 (m, 1H); ^{13}C NMR (100 MHz, $CDCl_3$) δ 200.2, 142.3, 137.1, 133.1, 128.7 (2), 128.5 (2), 128.2 (2), 127.6 (2), 126.8, 93.2, 79.2, 37.0, 36.2, 33.4, 31.5 (3), 27.7; IR (film): ν (cm^{-1}) 2968, 1682, 1597, 1581, 1477, 1450, 1363, 1228, 1201, 1159, 1030; HR-MS (ESI) *m/z* calcd for $C_{22}H_{24}AgO$ [M+Ag$^+$]: 411.0873; found 411.0874.

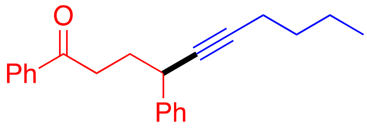

This compound was prepared following general procedure B using oxime ester **2a** (0.1 mmol) as starting material and purified by column chromatography on silica gel (petroleum ether/EtOAc 99:1).

1,4-Diphenyldec-5-yn-1-one (5y). Yellow oil, 61%, 18.5 mg. ^{1}H NMR (400 MHz, $CDCl_3$) δ 7.96 - 7.93 (m, 2H), 7.57 - 7.53 (m, 1H), 7.47 - 7.40 (m, 4H), 7.35 - 7.31 (m, 2H), 7.25 - 7.22 (m, 1H), 3.81 - 3.78 (m, 1H), 3.18 (ddd, J = 17.2, 9.2, 6.8 Hz, 1H), 3.08 (ddd, J = 17.2, 8.8, 5.2 Hz, 1H), 2.27 - 2.18 (m, 3H), 2.12 - 2.03 (m, 1H), 1.56 - 1.39 (m, 4H), 0.91 (t, J = 7.2 Hz, 3H); ^{13}C NMR (100 MHz, $CDCl_3$) δ 200.1, 142.2, 137.1, 133.1, 128.7 (2), 128.6 (2), 128.2 (2), 127.6 (2), 126.8, 84.4, 80.9, 37.2, 36.2, 33.1, 31.3, 22.2, 18.7, 13.8; IR (film): ν (cm^{-1}) 2956, 2931, 2871, 1707, 1682, 1597, 1448, 1363, 1225, 1178, 989; HR-MS (ESI) *m/z* calcd for $C_{22}H_{24}AgO$ [M+Ag$^+$]:

411.0873; found 411.0873.

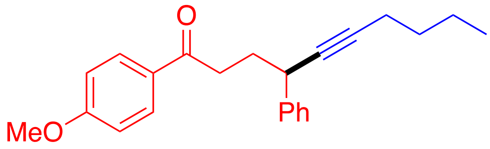

This compound was prepared following general procedure B using oxime ester **2b** (0.1 mmol) as starting material and purified by column chromatography on silica gel (petroleum ether/acetone 97:3).

1-(4-Methoxyphenyl)-4-phenyldec-5-yn-1-one (5z). Yellow oil, 77%, 25.6 mg. ^{1}H NMR (400 MHz, $CDCl_3$) δ 7.95 - 7.91 (m, 2H), 7.42 - 7.40 (m, 2H), 7.34 - 7.30 (m, 2H), 7.25 - 7.21 (m, 1H), 6.94 - 6.90 (m, 2H), 3.87 (s, 3H), 3.78 (m, 1H), 3.11 (ddd, *J* = 16.8, 8.8, 6.4 Hz, 1H), 3.02 (ddd, *J* = 16.8, 8.8, 5.2 Hz, 1H), 2.24 (td, *J* = 6.8, 2.4 Hz, 2H), 2.23 - 2.16 (m, 1H), 2.10 - 2.01 (m, 1H), 1.56 - 1.48 (m, 2H), 1.47 - 1.39 (m, 2H), 0.92 (t, *J* = 7.2 Hz, 3H); ^{13}C NMR (100 MHz, $CDCl_3$) δ 198.7, 163.5, 142.3, 130.4 (2), 130.3, 128.6 (2), 127.6 (2), 126.8, 113.8 (2), 84.3, 81.0, 55.6, 37.3, 35.9, 33.3, 31.3, 22.2, 18.7, 13.8; IR (film): ν (cm^{-1}) 2956, 2931, 1676, 1599, 1576, 1510, 1452, 1361, 1309, 1254, 1169, 1028; HR-MS (ESI) *m/z* calcd for $C_{23}H_{26}AgO_2$ [M+Ag$^+$]: 441.0978; found 441.0989.

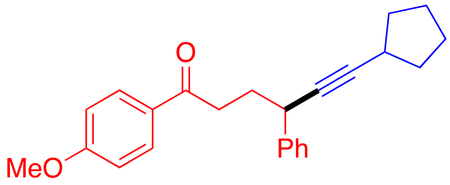

This compound was prepared following general procedure B using oxime ester **2b** (0.1 mmol) as starting material and purified by column chromatography on silica gel (toluene/petroleum ether 95:5).

6-Cyclopentyl-1-(4-methoxyphenyl)-4-phenylhex-5-yn-1-one (5aa). Light yellow oil, 67%, 23.7 mg. ^{1}H NMR (400 MHz, $CDCl_3$) δ 7.95 - 7.91 (m, 2H), 7.41 - 7.39 (m, 2H), 7.34 - 7.30 (m, 2H), 7.25 - 7.21 (m, 1H), 6.94 - 6.90 (m, 2H), 3.87 (s, 3H), 3.78 (ddd, *J* = 8.0, 5.6, 2.0 Hz, 1H), 3.11 (ddd, *J* = 16.4, 8.8, 6.4 Hz, 1H), 3.01 (ddd, *J* = 16.4, 8.8, 5.2 Hz, 1H), 2.67 (pd, *J* = 7.6, 2.0 Hz, 1H), 2.25 - 2.16 (m, 1H), 2.09 - 2.00 (m, 1H), 1.96 - 1.88 (m, 2H), 1.78 - 1.69 (m, 2H), 1.66 - 1.50 (m, 4H); ^{13}C NMR (100 MHz, $CDCl_3$) δ 198.7, 163.5, 142.4, 130.5 (2), 130.2, 128.5 (2), 127.6 (2), 126.8, 113.8 (2), 88.7, 80.5, 55.6, 37.3, 35.9, 34.3, 34.3, 33.5, 30.5, 25.1 (2); IR (film): ν (cm^{-1}) 2956, 2158, 1674, 1599, 1512, 1450, 1309, 1255, 1171; HR-MS (ESI) *m/z* calcd for $C_{24}H_{26}NaO_2$ [M+Na$^+$]: 369.1825; found 369.1818.

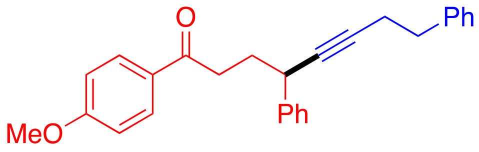

This compound was prepared following general procedure B using oxime ester **2b** (0.1 mmol) as starting material and purified by column chromatography on silica gel (petroleum ether/EtOAc 96:4).

1-(4-Methoxyphenyl)-4,8-diphenyloct-5-yn-1-one (5ab). Yellow oil, 73%, 28.0 mg. ^{1}H NMR (400 MHz, $CDCl_3$) δ 7.90 - 7.86 (m, 2H), 7.35 - 7.15 (m, 10H), 6.94 - 6.90 (m, 2H), 3.87 (s, 3H), 3.77 - 3.73 (m, 1H), 3.01 (ddd, J = 16.8, 8.8, 6.4 Hz, 1H), 2.92 (ddd, J = 16.8, 8.8, 5.2 Hz, 1H), 2.84 (t, J = 7.2 Hz, 2H), 2.55 (td, J = 7.6, 2.4 Hz, 2H), 2.22 - 2.13 (m, 1H), 2.07 - 1.98 (m, 1H); ^{13}C NMR (100 MHz, $CDCl_3$) δ 198.6, 163.5, 142.1, 140.9, 130.5 (2), 130.3, 128.7 (2), 128.6 (2), 128.5 (2), 127.6 (2), 126.8, 126.4, 113.8 (2), 83.3, 82.0, 55.6, 37.2, 35.8, 35.5, 33.1, 21.1; IR (film): ν (cm^{-1}) 2931, 1674, 1599, 1508, 1452, 1309, 1255, 1169; HR-MS (ESI) *m/z* calcd for $C_{27}H_{27}O_2$ [$M+H^+$]: 383.2006; found 383.2002.

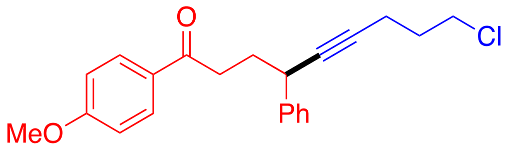

This compound was prepared following general procedure B using oxime ester **2b** (0.1 mmol) as starting material and purified by column chromatography on silica gel (petroleum ether/EtOAc 95:5).

9-Chloro-1-(4-methoxyphenyl)-4-phenylnon-5-yn-1-one (5ac). Yellow oil, 66%, 23.3 mg. ^{1}H NMR (400 MHz, $CDCl_3$) δ 7.94 - 7.91 (m, 2H), 7.40 - 7.38 (m, 2H), 7.35 - 7.31 (m, 2H), 7.26 - 7.22 (m, 1H), 6.95 - 6.91 (m, 2H), 3.87 (s, 3H), 3.78 (ddd, J = 10.4, 4.8, 2.0 Hz, 1H), 3.66 (t, J = 6.4 Hz, 2H), 3.10 (ddd, J = 17.2, 8.8, 6.8 Hz, 1H), 3.01 (ddd, J = 17.2, 8.4, 5.6 Hz, 1H), 2.44 (td, J = 6.8, 2.4 Hz, 2H), 2.25 - 2.17 (m, 1H), 2.12 - 2.03 (m, 1H), 1.97 (quint, J = 6.4 Hz, 2H); ^{13}C NMR (100 MHz, $CDCl_3$) δ 198.5, 163.6, 142.0, 130.4 (2), 130.2, 128.7 (2), 127.5 (2), 126.9, 113.8 (2), 82.4, 82.0, 55.6, 44.0, 37.2, 35.8, 33.1, 31.8, 16.5; IR (film): ν (cm^{-1}) 2927, 1674, 1599, 1496, 1450, 1417, 1363, 1309, 1254, 1171, 1028; HR-MS (ESI) *m/z* calcd for $C_{22}H_{23}ClNaO_2$ [$M+Na^+$]: 377.1279; found 377.1278.

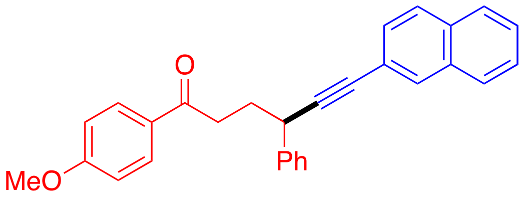

This compound was prepared following general procedure B using oxime ester **2b** (0.1 mmol) as starting material and purified by column chromatography on silica gel (petroleum ether/EtOAc 95:5).

1-(4-Methoxyphenyl)-6-(naphthalen-2-yl)-4-phenylhex-5-yn-1-one (5ad). Yellow solid, 68%, 27.4 mg. Mp: 69 - 71 °C; ^{1}H NMR (400 MHz, $CDCl_3$) δ 7.98 - 7.95 (m, 3H), 7.82 - 7.76 (m, 3H), 7.53 - 7.45 (m, 5H), 7.40 - 7.36 (m, 2H), 7.30 - 7.26 (m, 1H), 6.94 - 6.90 (m, 2H), 4.09 (dd, J = 8.8, 5.6 Hz, 1H), 3.86 (s, 3H), 3.24 (ddd, J = 17.2, 8.8, 6.8 Hz, 1H), 3.13 (ddd, J = 17.2, 8.4, 5.6 Hz, 1H), 2.42 - 2.34 (m, 1H), 2.31 - 2.22 (m, 1H); ^{13}C NMR (100 MHz, $CDCl_3$) δ 198.4, 163.6, 141.6, 133.1, 132.8, 131.4, 130.5 (2), 130.2, 128.8 (3), 128.0, 127.9, 127.8, 127.7 (2), 127.1, 126.6 (2), 121.0, 113.8 (2), 91.3, 84.5, 55.6, 37.9, 35.9, 33.1; IR (film): ν (cm^{-1}) 2954, 1668, 1599, 1576, 1508, 1450, 1417, 1361, 1304, 1257, 1240, 1203, 1173, 1028; HR-MS (ESI) *m/z* calcd for $C_{29}H_{24}AgO_2$ [M+Ag$^+$]: 511.0822; found 511.0816.

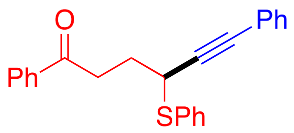

This compound was prepared following general procedure B using oxime ester **2m** (0.1 mmol) as starting material and purified by column chromatography on silica gel (petroleum ether/EtOAc 97:3).

1,6-Diphenyl-4-(phenylthio)hex-5-yn-1-one (5ae). Colorless oil, 46%, 16.4 mg. ^{1}H NMR (400 MHz, $CDCl_3$) δ 8.00 - 7.98 (m, 2H), 7.61 - 7.55 (m, 3H), 7.48 - 7.44 (m, 2H), 7.36 - 7.27 (m, 8H), 4.23 (dd, J = 7.6, 6.0 Hz, 1H), 3.40 - 3.27 (m, 2H), 2.41 - 2.32 (m, 1H), 2.29 - 2.20 (m, 1H); ^{13}C NMR (100 MHz, $CDCl_3$) δ 199.2, 136.9, 133.6 (2), 133.5, 133.3, 131.8 (2), 129.0 (2), 128.8 (2), 128.4 (3), 128.2 (2), 128.1, 123.0, 88.4, 85.5, 39.1, 35.9, 29.6; IR (film): ν (cm^{-1}) 3059, 1684, 1597, 1581, 1489, 1442, 1363, 1215, 1180, 1068, 1024, 997; HR-MS (ESI) *m/z* calcd for $C_{24}H_{20}NaOS$ [M+Na$^+$]: 379.1127; found 379.1117.

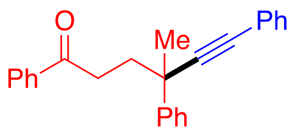

This compound was prepared following general procedure B using oxime ester **2n** (0.1 mmol) as starting material and purified by column chromatography on silica gel (petroleum ether/EtOAc 97:3).

4-Methyl-1,4,6-triphenylhex-5-yn-1-one (5af). Yellow solid, 45% 15.2 mg. Mp: 87 - 89 °C; ^{1}H NMR (400 MHz, $CDCl_3$) δ 7.89 - 7.87 (m, 2H), 7.65 - 7.63 (m, 2H), 7.53 -

7.47 (m, 3H), 7.42 - 7.31 (m, 7H), 7.28 - 7.25 (m, 1H), 3.24 (ddd, *J* = 16.8, 9.6, 6.4 Hz, 1H), 2.86 - 2.78 (m, 1H), 2.41 - 2.33 (m, 2H), 1.74 (s, 3H); ^{13}C NMR (100 MHz, $CDCl_3$) δ 200.3, 144.6, 137.0, 133.1, 131.8 (2), 128.6 (2), 128.6 (2), 128.4 (2), 128.2 (2), 128.1, 126.9, 126.3 (2), 123.6, 94.1, 85.0, 40.9, 38.3, 35.6, 31.1; IR (film): ν (cm^{-1}) 2968, 2925, 1674, 1597, 1577, 1491, 1446, 1375, 1358, 1294, 1223, 1205; HR-MS (ESI) *m/z* calcd for $C_{25}H_{22}AgO$ [M+Ag$^+$]: 445.0716; found 445.0721.

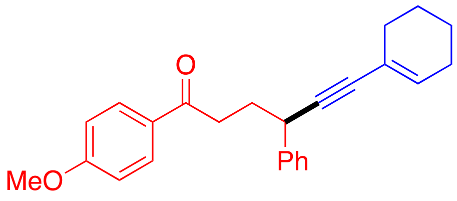

This compound was prepared following general procedure B using oxime ester **2b** (0.1 mmol) as starting material and purified by column chromatography on silica gel (toluene/petroleum ether 95:5).

6-(Cyclohex-1-en-1-yl)-1-(4-methoxyphenyl)-4-phenylhex-5-yn-1-one (5ag). Light yellow oil, 76%, 27.2 mg. ^{1}H NMR (400 MHz, CD_3OD) δ 7.94 - 7.92 (m, 2H), 7.39 - 7.30 (m, 4H), 7.24 - 7.21 (m, 1H), 7.01 - 6.97 (m, 2H), 6.02 - 6.00 (m, 1H), 3.90 - 3.87 (m, 1H), 3.87 (s, 3H), 3.13 (ddd, *J* = 16.8, 8.4, 6.8 Hz, 1H), 3.03 (ddd, *J* = 16.8, 8.0, 5.6 Hz, 1H), 2.20 - 1.99 (m, 6H), 1.67 - 1.56 (m, 4H); ^{13}C NMR (100 MHz, CD_3OD) δ 200.7, 165.3, 143.4, 134.5, 131.5 (2), 131.1, 129.5 (2), 128.5 (2), 127.8, 122.1, 114.9 (2), 88.8, 87.0, 56.0, 38.6, 36.7, 34.6, 30.6, 26.5, 23.5, 22.7; IR (film): ν (cm^{-1}) 2933, 1672, 1599, 1309, 1254, 1169; HR-MS (ESI) *m/z* calcd for $C_{25}H_{26}AgO_2$ [M+Ag$^+$]: 465.0978; found 465.0985.

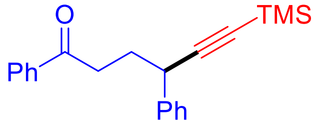

This compound was prepared following general procedure B using oxime ester **2a** (0.1 mmol) as starting material and purified by column chromatography on silica gel (petroleum ether/EtOAc 99:1).

1,4-Diphenyl-6-(trimethylsilyl)hex-5-yn-1-one (5ah). Colorless oil, 56%, 18.0 mg. ^{1}H NMR (400 MHz, $CDCl_3$) δ 7.96 – 7.94 (m, 2H), 7.58 – 7.54 (m, 1H), 7.48 – 7.44 (m, 2H), 7.42 – 7.40 (m, 2H), 7.36 – 7.32 (m, 2H), 7.27 – 7.23 (m, 1H), 3.85 (dd, J = 8.8, 5.6 Hz, 1H), 3.19 (ddd, *J* = 17.2, 9.2, 6.4 Hz, 1H), 3.07 (ddd, *J* = 17.2, 8.8, 5.6 Hz, 1H), 2.30 – 2.21 (m, 1H), 2.14 – 2.05 (m, 1H), 0.19 (s, 9H); ^{13}C NMR (100 MHz, $CDCl_3$) δ 199.9, 141.2, 137.1, 133.2, 128.7 (2), 128.7 (2), 128.2 (2), 127.6 (2), 127.0, 107.5, 88.5, 38.0, 36.1, 32.9, 0.28 (3); IR (film): ν (cm^{-1}) 2958, 2171, 1684, 1450, 1248, 1232; HR-MS (ESI) *m/z* calcd for $C_{21}H_{24}NaOSi$ [M+Na$^+$]: 343.1489; found 343.1487.

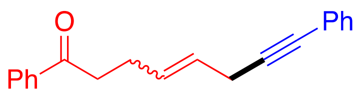

This compound was prepared following general procedure B using oxime ester **2o** (0.1 mmol) as starting material and purified by column chromatography on silica gel (petroleum ether/EtOAc 98:2).

1,8-Diphenyloct-4-en-7-yn-1-one (5ai). Pale yellow oil, 75%, 20.5 mg. ^{1}H NMR (400 MHz, $CDCl_3$) δ (*Mixture of isomers in a ratio of 1:0.7*) 8.00 - 7.95 (m, 2H), 7.58 - 7.54 (m, 1H), 7.48 - 7.38 (m, 4H), 7.30 - 7.25 (m, 3H), 5.89 - 5.81 (m, 0.7H), 5.63 - 5.55 (m, 1.3H), 3.23-3.07 (m, 4H), 2.59 - 2.48 (m, 2H); ^{13}C NMR (100 MHz, $CDCl_3$) δ (*Major isomer*) 199.6, 137.1, 133.1, 131.7 (2), 130.8, 128.7 (2), 128.3 (2), 128.2 (2), 127.8, 125.2, 123.9, 87.5, 82.5, 38.3, 26.9, 22.8; (*Minor isomer*) 199.5, 137.0, 133.2, 131.7 (2), 130.3, 128.7 (2), 128.3 (2), 128.2 (2), 127.8, 125.5, 123.9, 88.3, 80.5, 38.3, 22.1, 18.0; IR (film): ν (cm^{-1}) 2914, 1684, 1597, 1579, 1489, 1446, 1412, 1361, 1236, 1203, 1070; HR-MS (ESI) *m/z* calcd for $C_{20}H_{18}AgO$ [M+Ag^{+}]: 381.0403; found 381.0414.

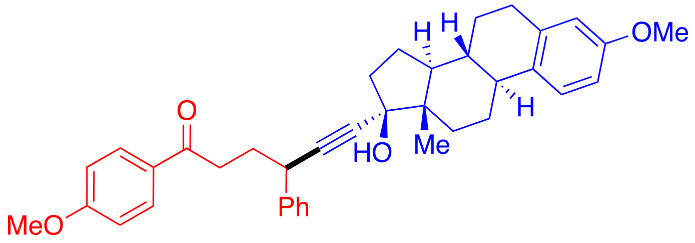

This compound was prepared following general procedure B using oxime ester **2b** (0.1 mmol) and mestranol derivative (0.2 mmol) as starting materials, purified by column chromatography on silica gel (petroleum ether/EtOAc 8:2) and obtained as a mixture 1:1 of diastereoisomers.

6-((8*R*,9*S*,13*S*,14*S*,17*S*)-17-Hydroxy-3-methoxy-13-methyl-7,8,9,11,12,13,14,15,16,17-decahydro-6*H*-cyclopenta[*a*]phenanthren-17-yl)-1-(4-methoxyphenyl)-4-phenyl-hex-5-yn-1-one (9b). Light yellow oil, 72%, 40.5 mg. ^{1}H NMR (400 MHz, $CDCl_3$) δ 7.92 - 7.88 (m, 4H), 7.42 - 7.40 (m, 4H), 7.35 - 7.31 (m, 4H), 7.26 - 7.22 (m, 2H), 7.17 (dd, J = 8.8, 3.6 Hz, 2H), 6.88 - 6.83 (m, 4H), 6.71 (dd, J = 8.4, 2.8 Hz, 2H), 6.62 (d, J = 2.8 Hz, 2H), 3.93 - 3.88 (m, 2H), 3.80 (s, 3H), 3.79 (s, 3H), 3.78 (s, 6H), 3.21 - 3.12 (m, 2H), 3.09 - 3.01 (m, 2H), 2.90 - 2.77 (m, 4H), 2.34 - 2.23 (m, 6H), 2.17 - 2.00 (m, 6H), 1.95 - 1.67 (m, 12H), 1.55 - 1.35 (m, 6H), 1.34 - 1.20 (m, 2H), 0.88 (s, 6H); ^{13}C NMR (100 MHz, $CDCl_3$) δ 198.2 (2), 163.6 (2), 157.6 (2), 141.6 (2), 138.1 (2), 132.6, 132.6, 130.4 (4), 130.1, 130.1, 128.7 (4), 127.6 (4), 127.0 (2), 126.5 (2), 113.9 (2), 113.8 (4), 111.6 (2), 87.6, 87.6, 87.2 (2), 80.2, 80.2, 55.5 (2), 55.3 (2), 50.0, 49.9, 47.5 (2), 43.9 (2), 39.5 (2), 39.4 (2), 37.2 (2), 35.8 (2), 33.2, 33.2, 32.9 (2), 29.9 (2), 27.5 (2), 26.5 (2), 23.0 (2), 13.0 (2); IR (film): ν (cm^{-1}) 3464, 2930, 1672, 1600, 1577, 1498, 1452, 1363, 1310, 1242, 1170, 1030; HR-MS (ESI) *m/z* calcd for

$C_{38}H_{42}AgO_4$ [M+Ag$^+$]: 669.2129; found 669.21231.

Mechanistic Study (Radical Inhibition Experiments)

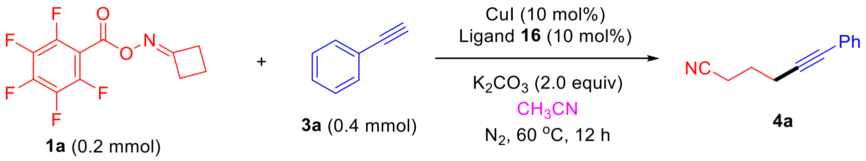

Additive	4a, yield (%)[a]
None	76%
BHT (3 equiv)	10%
TEMPO (1 equiv) or (3 equiv)	0%

Optimized conditions: Oxime ester **1a** (0.2 mmol, 1 equiv), phenylacetylene **3a** (0.4 mmol, 2.0 equiv), K_2CO_3 (0.4 mmol, 2 equiv), CuI (0.02 mmol, 10 mol%), ligand **16** (0.04 mmol, 20 mol%), CH_3CN (1 mL); [a] ^{1}H NMR yield based on **4a**, with 1,3,5-trimethoxybenzene as internal standard.

Derivatization of 6-(4-methoxyphenyl)hex-5-ynenitrile (4c) and 1,4,6-triphenylhex-5-yn-1-one (5a)

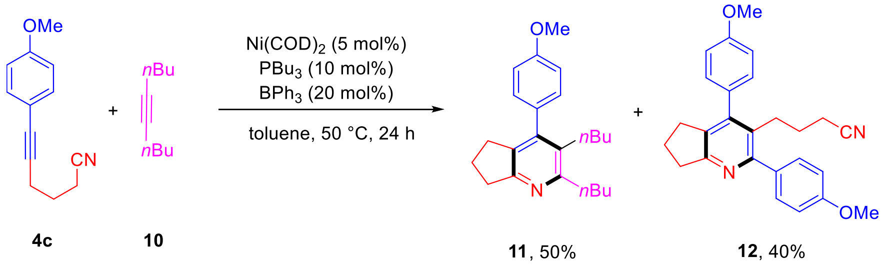

This reaction was performed following Liu's procedure[3].

In a nitrogen-filled glove box, to a screw-cap vial were added $Ni(COD)_2$ (4.1 mg, 0.015 mmol), PBu_3 (7.5 µL, 0.03 mmol), BPh_3 (14.5 mg, 0.06 mmol) and toluene (1.0 mL). The resulting solution was stirred at room temperature for 10 - 20 min. Then 6-(4-methoxyphenyl)hex-5-ynenitrile (**4c**, 80 mg, 0.4 mmol) and dec-5-yne (54 µL, 0.3 mmol) were added. The vial was taken outside the glove box and stirred at 50 °C for 24 h. The resulting mixture was filtered through a plug of silica gel. The solvent was evaporated under the reduced pressure and the residue was purified by flash column chromatography on silica gel (petroleum ether/ethyl acetate 10/1 to 3/1) to afford **11** as a yellow oil in 50% yield (33.7 mg) and **12** as a white solid in 40% yield (31.8 mg).

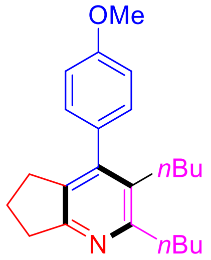

2,3-Dibutyl-4-(4-methoxyphenyl)-6,7-dihydro-5*H*-cyclopenta[*b*]pyridine (11). Yellow oil, 50%, 33.7 mg. ^{1}H NMR (400 MHz, $CDCl_3$) δ 7.08 (d, *J* = 8.8 Hz, 2H), 6.95 (d, *J* = 8.8 Hz, 2H), 3.85 (s, 3H), 3.02 (t, *J* = 7.6 Hz, 2H), 2.81 - 2.77 (m, 2H), 2.57 (t, *J* = 7.2 Hz, 2H), 2.45 - 2.41 (m, 2H), 2.05 - 1.97 (m, 2H), 1.75 - 1.67 (m, 2H), 1.51 - 1.42 (m, 2H), 1.34 - 1.26 (m, 2H), 1.22 - 1.13 (m, 2H), 0.96 (t, *J* = 7.6 Hz, 3H), 0.75 (t, *J* = 7.2 Hz, 3H); ^{13}C NMR (100 MHz, $CDCl_3$) δ 161.6, 159.1, 158.8, 146.5, 133.6, 131.3, 130.8, 129.5, 113.8, 55.3, 35.5, 34.7, 33.4, 33.2, 30.5, 28.7, 23.3, 23.0, 14.2, 13.7; IR (film): ν (cm^{-1}) 2954, 1610, 1511, 1466, 1289, 1173, 1035, 836, 753; HR-MS (ESI) *m/z* calcd for $C_{23}H_{32}NO$ [$M+H^+$]: 338.2478; found 338.2475.

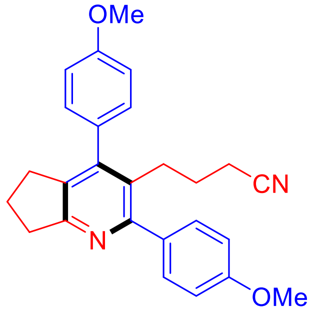

2,3-Dibutyl-4-(4-methoxyphenyl)-6,7-dihydro-5*H*-cyclopenta[*b*]pyridine (12). Yellow oil, 40%, 31.8 mg. ^{1}H NMR (400 MHz, $CDCl_3$) δ 7.39 - 7.35 (m, 2H), 7.17 - 7.13 (m, 2H), 7.02 - 6.95 (m, 4H), 3.87 (s, 3H), 3.85 (s, 3H), 3.08 (t, *J* = 7.6, 2H), 2.70 - 2.63 (m, 4H), 2.11 - 2.04 (m, 2H), 1.87 (t, *J* = 7.2 Hz, 2H), 1.46 - 1.39 (m, 2H); ^{13}C NMR (100 MHz, $CDCl_3$) δ 163.0, 159.3, 159.2, 158.0, 146.9, 135.2, 134.1, 130.3, 130.1, 129.3, 128.8, 119.2, 114.3, 114.0, 55.4, 55.4, 34.8, 30.5, 28.5, 25.9, 23.1, 16.9; IR (film): ν (cm^{-1}) 2963, 1610, 1512, 1243, 1035, 909, 832, 728; HR-MS (ESI) *m/z* calcd for $C_{26}H_{27}N_2O_2$ [$M+H^+$]: 399.2067; found 399.2065.

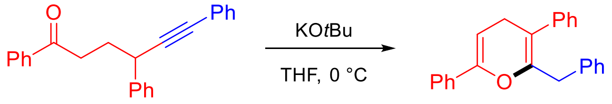

5a **13**, 85%

To a solution of alkyne **5a** (10 mg, 30.8 μmol) in THF (1.0 mL) was added a mixture of KO*t*Bu (4.2 mg, 37 μmol) in THF (0.5 mL) at 0 °C. The reaction was stirred for 1 h, then quenched with a saturated NH_4Cl solution and extracted with EtOAc (3 x 15 mL).

The combined organic layers were dried over Na_2SO_4 and evaporated to dryness under reduced pressure. The residue was pure enough to be fully characterized.

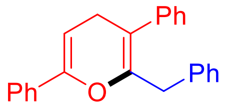

2-Benzyl-3,6-diphenyl-4*H*-pyran (13). Yellow oil, 85%, 8.5 mg. ^{1}H NMR (400 MHz, $CDCl_3$) δ 7.49 – 7.46 (m, 3H), 7.38 – 7.20 (m, 17H), 5.44 (t, J = 3.6 Hz, 1H), 3.56 (s, 2H), 3.22 (d, J = 4.0 Hz, 2H); ^{13}C NMR (100 MHz, $CDCl_3$) δ 149.0, 145.9, 141.0, 138.1, 134.5, 128.7 (2), 128.7 (2), 128.5 (2), 128.5 (2), 128.3 (2), 128.2, 127.0, 126.3, 124.4 (2), 110.5, 95.2, 36.8, 28.4; IR (film): ν (cm^{-1}) 2920, 1684, 1599, 1493, 1448, 1230, 1072; HR-MS (ESI) *m/z* calcd for [M$^+$]: 324.1509; found 324.1495.

Supplementary References

1. Zhao, B. & Shi, Z. Copper-catalyzed intermolecular Heck-like coupling of cyclobutanone oximes initiated by selective C-C bond cleavage. *Angew. Chem. Int. Ed.* **56**, 12727-12731 (2017).
2. Yu, X.-Y., Chen, J.-R., Wang, P.-Z., Yang, M.-N., Liang, D. & Xiao, W.-J. A visible-light-driven iminyl radical-mediated C-C single bond cleavage/radical addition cascade of oximes. *Angew. Chem. Int. Ed.* **57**, 738-743 (2018).
3. Le Vaillant, F., Garreau, M., Nocolai, S., Gryn'ova, G., Corminboeuf, C. & Waser, J. Fine-tuned organic photoredox catalysts for fragmentation-alkynylation cascades of cyclic oxime ethers. *Chem. Sci.* **9**, 5883-5889 (2018).
4. You, X., Xie, X., Wang, G., Xiong, M., Sun, R., Chen, H. & Liu, Y. Nickel-catalyzed [2+2+2] cycloaddition of alkyne-nitriles with alkynes assisted by Lewis acids: Efficient synthesis of fused pyridines. C*hem. Eur. J.* **22**, 16765 (2016).
